# Ring-Closing Metathesis Approaches towards the Total Synthesis of Rhizoxins

**DOI:** 10.3390/molecules25194527

**Published:** 2020-10-02

**Authors:** Marc Liniger, Christian M. Neuhaus, Karl-Heinz Altmann

**Affiliations:** ETH Zürich, Department of Chemistry and Applied Biosciences, Institute of Pharmaceutical Sciences, 8093 Zürich, Switzerland; marc.liniger@givaudan.com (M.L.); chr.neuhaus1@gmail.com (C.M.N.)

**Keywords:** macrocyclization, macrolactone, natural products, paterson aldol reaction, reductive decomplexation, rhizoxin, ring-closing alkyne metathesis, radical-induced double bond isomerization, ring-closing olefin metathesis, total synthesis

## Abstract

Efforts are described towards the total synthesis of the bacterial macrolide rhizoxin F, which is a potent tubulin assembly and cancer cell growth inhibitor. A significant amount of work was expanded on the construction of the rhizoxin core macrocycle by ring-closing olefin metathesis (RCM) between C(9) and C(10), either directly or by using relay substrates, but in no case was ring-closure achieved. Macrocycle formation was possible by ring-closing alkyne metathesis (RCAM) at the C(9)/C(10) site. The requisite diyne was obtained from advanced intermediates that had been prepared as part of the synthesis of the RCM substrates. While the direct conversion of the triple bond formed in the ring-closing step into the C(9)-C(10) *E* double bond of the rhizoxin macrocycle proved to be elusive, the corresponding Z isomer was accessible with high selectivity by reductive decomplexation of the biscobalt hexacarbonyl complex of the triple bond with ethylpiperidinium hypophosphite. Radical-induced double bond isomerization, full elaboration of the C(15) side chain, and directed epoxidation of the C(11)-C(12) double bond completed the total synthesis of rhizoxin F.

## 1. Introduction

**Figure 1:**
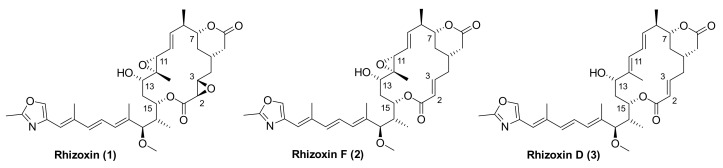
Molecular structures of rhizoxin (**1**), rhizoxin F (**2**), and rhizoxin D (**3**).

Rhizoxin (**1**) (Figure 1) is a 16-membered macrolide that was first isolated in 1984 from the plant pathogenic fungus *Rhizopus chinensis* by Iwasaki and co-workers [1] (for the elucidation of the absolute configuration of rhizoxin (**1**) by means of X-ray crystallography cf. [2]). Fungi of the genus *Rhizopus* are responsible for the rice seedling blight and rhizoxin (**1**) is the causative phytotoxin responsible for the disease [1].

Mechanistically, rhizoxin (**1**) binds to rice β-tubulin [3,4], which leads to inhibition of mitosis, cell cycle arrest, and, ultimately, death of the plant. *Rhizopus* sp. were believed to be the producers of rhizoxin (**1**) until 2005, when Partida-Martinez and Hertweck demonstrated that rhizoxin in *Rhizopus microsporus* is not produced by the fungus itself, but by endosymbiotic bacteria of the genus *Burkholderia* that were termed “*Burkholderia rhizoxina*” [5]. Partida-Martinez and Hertweck also located, cloned, and sequenced the entire gene locus that encodes rhizoxin biosynthesis in *Burkholderia rhizoxina* [6]. In addition, the endosymbiont was isolated in pure form and fermented on large scale, resulting in the recovery of a number of rhizoxin variants with highly potent biological activity, including WF-1360F (**2**) [7] (Figure 1), which we have termed rhizoxin F [8]. The isolation of rhizoxin F (**2**) had also been reported previously by Iwasaki et al. [9] and Kiyoto et al. [10] from *Rhizopus chinensis* and *Rhizopus* sp., respectively. In addition, rhizoxin variants have been isolated from fermentations of *Pseudomonas fluorescens* Pf-5 [11,12] (see also in [13]).

Rhizoxin (**1**) also binds to eukaryotic tubulin and prevents its assembly (“polymerization”) into microtubules [14,15]; in addition, preformed microtubules are depolymerized by the compound. As a consequence, rhizoxin (**1**) is a potent inhibitor of cancer cell growth in vitro with IC_50_ values in the single digit nM or even sub-nM range [11,16]. (For example, the IC_50_ value of 1 against the mouse P388 leukemia cell line is 0.16 nM [10].) Rhizoxin (**1**) had been suggested to bind to the β-subunit of the α/β-tubulin heterodimer at the same site as maytansine, and this binding site was proposed to be distinct from either the vinblastine or the colchicine site [14,15]. These hypotheses have recently been confirmed experimentally by means of X-ray crystallography on complexes of α/β-tubulin with rhizoxin F (**2**) and maytansine, respectively [8]. Importantly, compared to rhizoxin (**1**), monoepoxy-based rhizoxin F (**2**) has been reported to exhibit similar [10] or even significantly higher [8] in vitro antiproliferative activity.

Rhizoxin (**1**) has shown significant in vivo antitumor activity in animal models of cancer [11,16,17] and the compound has been evaluated in humans in a number of Phase I and Phase II clinical studies. However, the outcome of these trials was generally disappointing [18], although the reasons for the lack of clinical efficacy of rhizoxin (**1**) have not been elucidated in detail (based on public literature). It has been suggested that “further studies of this class of agents [rhizoxins] will depend on the identification of more efficacious analogs in preclinical models” [18].

The chemistry of rhizoxins has been explored extensively and as of today, 10 total syntheses of a naturally occurring rhizoxin have been reported in the literature. Out of these 10 syntheses, eight have targeted the epoxide-free rhizoxin variant rhizoxin D (**3**) (Figure 1) and only Ohno and co-workers have described the final conversion of 3 into rhizoxin [19]. In addition, we have reported the total synthesis of the monoepoxy variant rhizoxin F (**2**) [20]. Early work in the field (up to 2004) has been reviewed in detail in an excellent account by Hong and White [21] and shall not be discussed here, except to note that a recurring theme in the majority of these syntheses is the closure of the macrolactone ring through intramolecular Horner-Wittig-Emmons (HWE) reaction. Alternative approaches to ring-closure have been reported by Pattenden and co-workers (Stille coupling between C(10) and C(11)) [22] and by our own group (ring-closing alkyne metathesis (RCAM) between C(9) and C(10)) [20]. Most recently, Fürstner and co-workers have described an intriguing formal total synthesis of rhizoxin D (**3**) that included ring-closure by RCAM between C(11) and C(12) (!) [23]. The resulting triple bond was then efficiently transformed into the required trisubstituted *trans* double bond by *trans*-stannylation and subsequent C-methylation with excellent selectivity.

Our group has a long-standing interest in the chemistry, medicinal chemistry, and pharmacology of bioactive macrocyclic natural products. In this context, we were interested to develop a new synthetic approach towards rhizoxins that would be based on ring-closing olefin metathesis (RCM), which, somewhat surprisingly, had not been investigated as part of any of the previous total syntheses of a rhizoxin variant. The specific target to be pursued in our work was rhizoxin F (**2**); as alluded to above, this monoepoxy rhizoxin variant has been reported to be at least equipotent with rhizoxin (**1**) with regard to cancer cell proliferation in vitro. In a previous communication, we have reported the total synthesis of rhizoxin F (**2**) via RCAM [20]; in this communication, we also briefly touched upon our numerous futile attempts at RCM-based ring closure. In the current paper, we now disclose full details on these attempts and on the successful total synthesis of **2** based on macrocyclization by RCAM.

## 2. Results and Discussion

### 2.1. Synthetic Planning

**Scheme 1:**
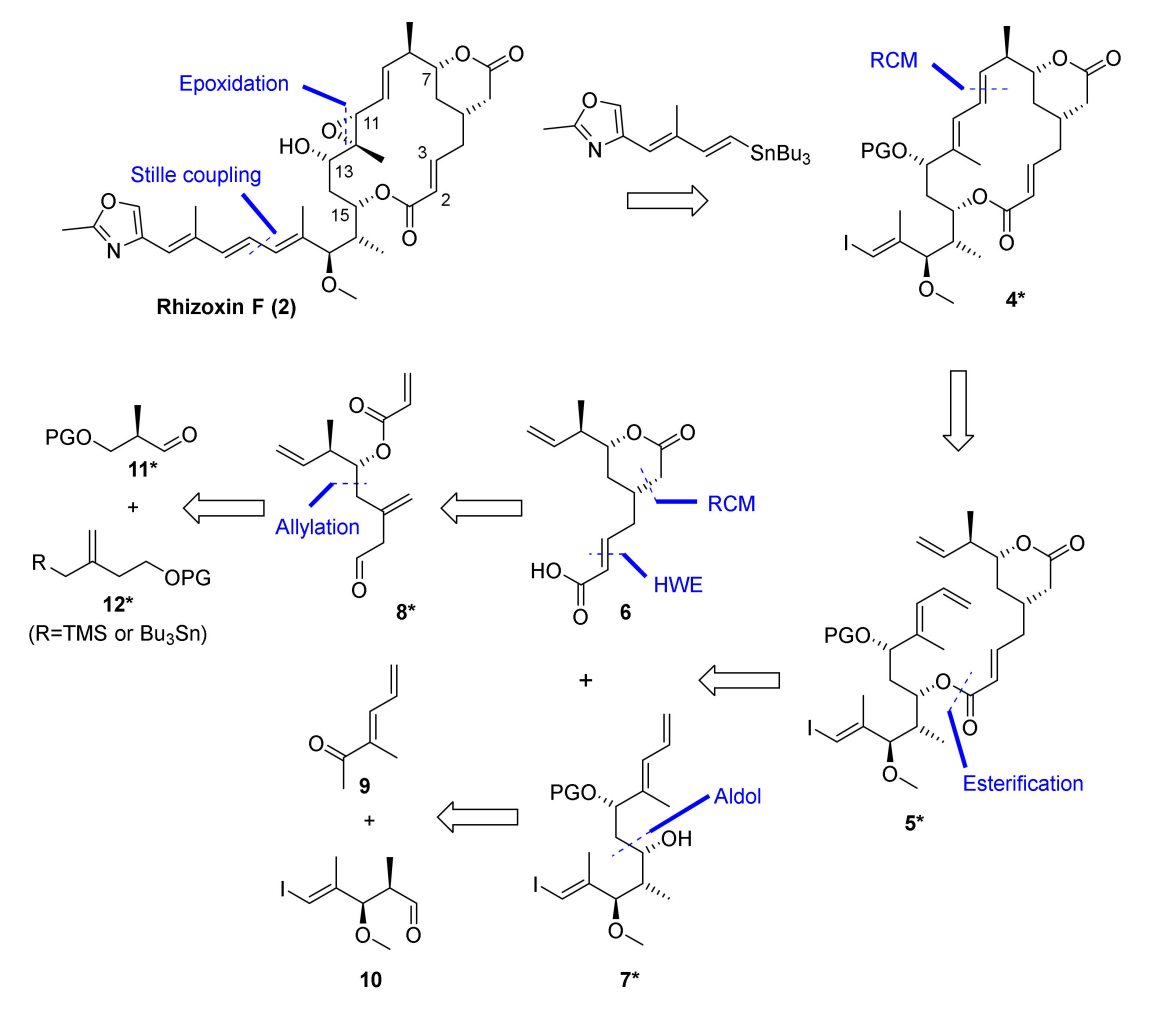
Retrosynthesis of rhizoxin F (**2**) based on ring-closing olefin metathesis (RCM) of diene 5*. PG, protecting group. The suffix “*” indicates that differently protected versions of the corresponding intermediates were contemplated at the planning stage.

Apart from the exploration of the feasibility of an RCM-based macrocyclization strategy, our total synthesis of **2** was also to provide a platform for the subsequent synthesis of side chain-modified rhizoxin F analogs for SAR studies. In this context, the intermediate formed in the ring-closure reaction was also meant to serve as a central node and point of diversification for the elaboration of differently modified side chains in a single step. One possible strategy for the synthesis of rhizoxin F (**2**) that would meet our predefined boundary conditions is outlined in Scheme 1. This strategy envisioned the late stage elaboration of the C15-side chain from macrocyclic vinyl iodide 4* by means of Stille coupling. Vinyl iodide 4* could also serve as a versatile common intermediate for the synthesis of differently modified side chain analogs by employing alternative vinyl stannanes as building blocks. Stille coupling was to be followed by deprotection of the C13 hydroxy group and directed epoxidation of the C(11)-C(12) double bond to deliver **2**; the feasibility of this latter transformation had already been demonstrated by Ohno and co-workers as part of their synthesis of rhizoxin (**1**), although for a differently decorated macrocycle [19].

Out the three possible diene precursors, our RCM-based disconnection of macrocycle 4* led to diene 5* as the most promising substrate, as it presents two unencumbered monosubstituted terminal double bonds as the sites of reaction. At the same time, 5* was envisaged to be accessible by the esterification of two fragments of similar size and structural complexity, i.e., acid **6** and alcohol 7*; therefore, this approach also offered the highest level of convergency for the total synthesis. The δ-lactone-containing carboxylic acid **6** was envisioned to be accessed by a diastereoselective allylation of aldehyde 11*, followed by RCM to close the δ-lactone ring. Alcohol 7* was planned to be obtained by the substrate-controlled 1,3-*anti* reduction of a β-hydroxy ketone precursor, which in turn was to be furnished by stereoselective aldol reaction between known aldehyde **10** and methyl ketone **11**, either under Mukaiyama [24,25] or Paterson [26] conditions. Acid **6** was envisioned to be accessed by two-carbon extension of aldehyde 8* via HWE olefination with an alkyl dialkylphosphono acetate; the former was envisaged to result from the stereoselective allylation of an aldehyde 11* with an allylsilane/allyl stannane 12* in a Hosomi-Sakurai- [27] or Keck-type [28] allylation reaction and subsequent functional group adjustment. 

### 2.2. Synthesis of Alcohol 7*

**Scheme 2:**
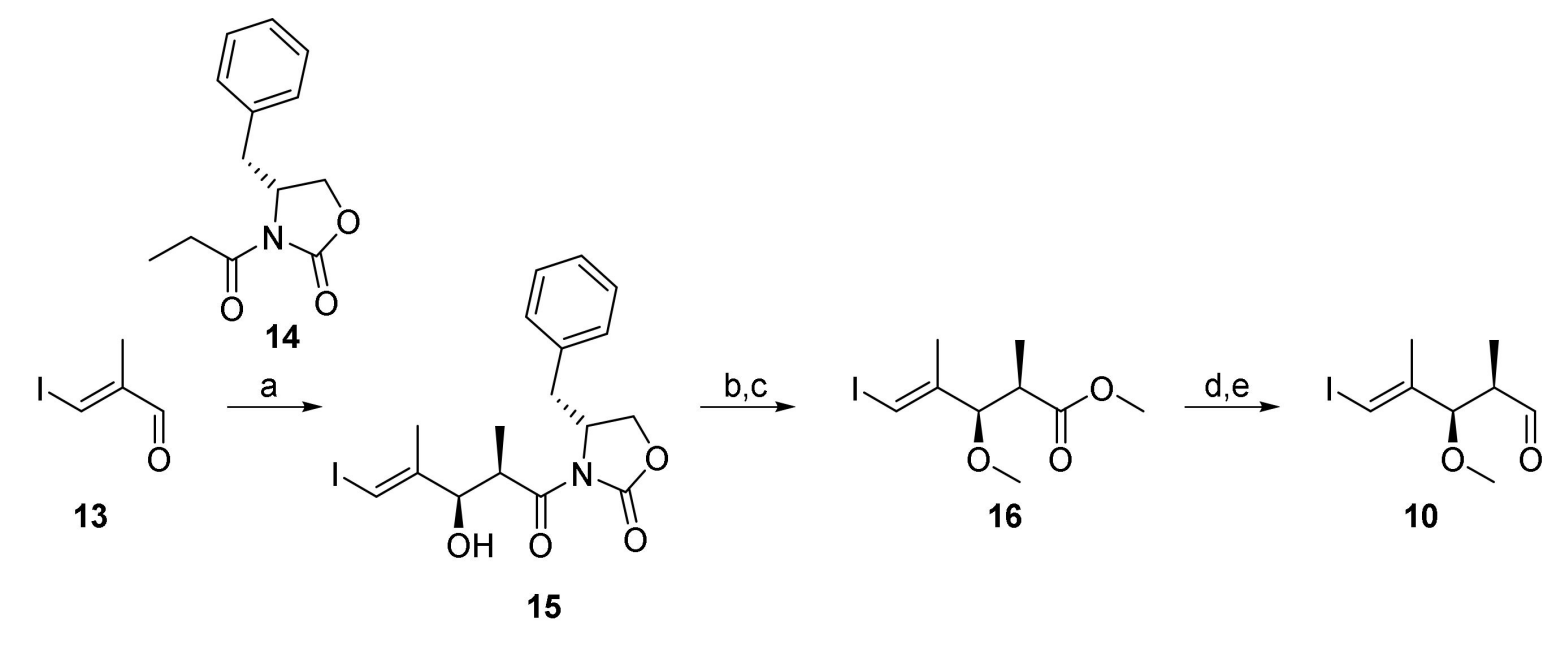
Reagents and conditions: (**a**) **14**, *n*Bu_2_BOTf, NEt_3_, CH_2_Cl_2_, 0 °C, then 13, −78 °C to 0 °C, 92%, single isomer; (**b**) NaOMe, CH_2_Cl_2_/MeOH (3:1), 0 °C, 95%; (**c**) NaH, MeI, THF/DMF (3:1), 0 °C, 94%; (**d**) DIBAL, CH_2_Cl_2_, −78 °C to 0 °C, 97%; (**e**) DMP, NaHCO_3_, 0 °C to rt. DIBAL, diisobutylaluminum hydride; DMP, Dess–Martin periodinane (1,1,1-tris(acetyloxy)-1,1-dihydro-1,2-benziodoxol-3-(1H)-one); Tf, trifluormethansulfonyl.

As illustrated in Scheme 2, the synthesis of aldehyde **10** as one of the building blocks for the construction of 7* was based on a high-yielding and highly stereoselective Evans aldol reaction between aldehyde **13** and *N*-propionyl-oxazolidinone **14** (92% yield of a **15** as a single isomer), as has been described previously by White and co-workers [29]; aldehyde **13** was obtained from diethyl methyl malonate according to Menche et al. [30]. Imide **15** was then cleanly converted into methyl ester **16** by treatment with sodium methoxide. The latter was elaborated into aldehyde **10** by methylation with NaH and MeI, followed by DIBAL reduction of the ester group (91% overall yield) and Dess-Martin periodinane (DMP) [31] oxidation of the ensuing primary alcohol. In order to prevent epimerization of **10** during chromatography, the crude aldehyde was directly used in the aldol step.

**Scheme 3:**
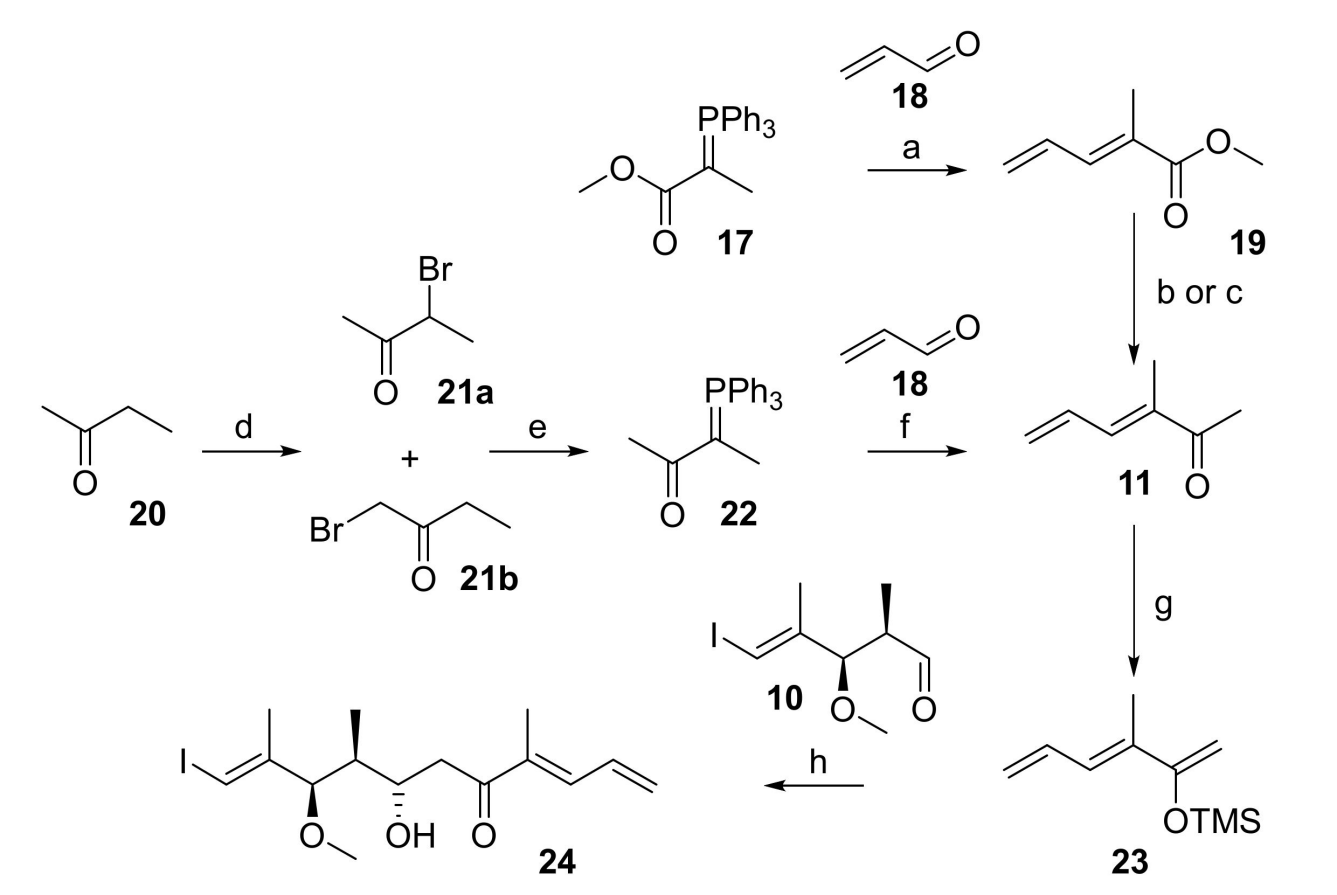
Reagents and conditions: (**a**) CH_2_Cl_2_, rt to rf, 70%; (**b**) Me(OMe)NH∙HCl, MeMgCl, THF, −15 °C to rt, 51%; (**c**) LiHMDS, MeMgCl, THF, −78 °C to +10 °C, 35%; (**d**) Br_2_, AcOH, H_2_O, 65 °C, 47%, 21a:21b = 11:1; (**e**) (**i**) PPh_3_, MeCN, 70 °C; (**ii**) NaOH, MeCN, rt, 79%; (**f**) CH_2_Cl_2_, rt to rf, 57%; (**g**) NEt_3_, TMSCl, NaI, CH_3_CN, 0 °C to 90 °C, 86%; (**h**) TiCl_4_, CH_2_Cl_2_, −78 °C, 12%, *dr* 4:1. LiHMDS, lithium bis(trimethylsilyl)amide; TMS, trimethylsilyl.

In initial experiments, methyl ketone **11** was prepared via the known ester **19** [32] (Scheme 3), which was obtained by Wittig reaction of phosphorane **17** with acrolein (**18**) in 70% yield. Ester **19** could be converted into **11** either by enolate trap Grignard addition or by one-pot Weinreb ketone synthesis [33]. However, for both approaches the yield of **11** was moderate at best and the reactions suffered from poor reproducibility. Alternatively, methyl ketone **11** was accessed more reliably from 2-butanone (**20**), which was α-brominated to yield an 11:1 mixture of bromides **21a** and **21b** in 47% yield (Scheme 3). Reaction of the mixture of **21a**/**21b** with PPh_3_ in acetonitrile gave a crude phosphorane **22** that reacted with acrolein (**18**) to furnish **11** as a single isomer in 57% yield. Notably, the treatment of **21a/b** with aqueous NaOH (which were the conditions used for the preparation of **17** from its phosphonium salt precursor in high yield) did not give any of the desired phosphorane **22**.

With aldehyde **10** and methyl ketone **11** in hand, the stage was set for the projected aldol reaction. To this end, ketone **11** was first transformed into its TMS-enol ether **23** as the necessary precursor for a Mukaiyama aldol reaction [24,25] (Scheme 3). The best conditions for the formation of **23** involved treatment of **11** with triethylamine, TMS-chloride, and sodium iodide in acetonitrile, a method developed by Cazeau et al. for the special purpose of forming TMS-enol ethers from α,β-unsaturated ketones and aldehydes [34]. In analogy to the work of Pattenden et al. [22], enol ether **23** was then submitted to reaction with aldehyde **10** in the presence of Me_2_AlCl; however, these conditions led to a mixture of at least three compounds, whose spectroscopic data could not be interpreted. If TiCl_4_ was used as a Lewis acid, the aldol product was indeed obtained, but only in 12% yield and with moderate diastereoselectivity (4:1).

**Scheme 4:**
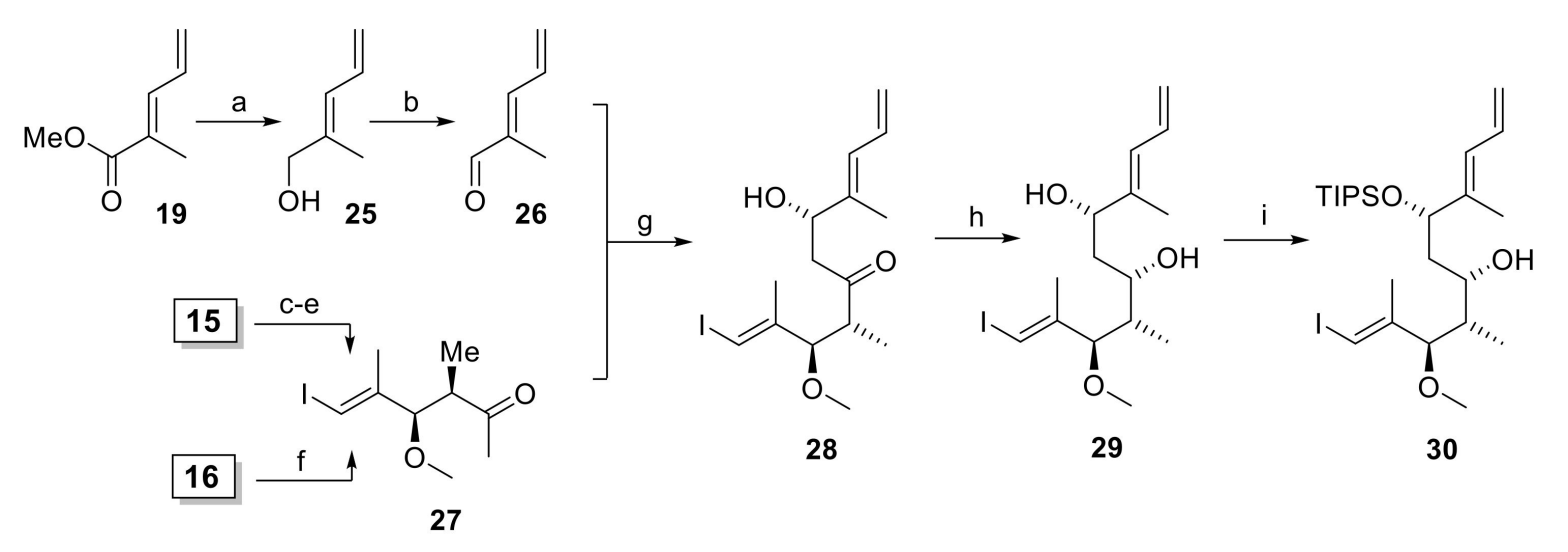
Reagents and conditions: (**a**) LiAlH_4_, Et_2_O, 0 °C; (**b**) activated MnO_2_, CH_2_Cl_2_, rt, 78% (2 steps); (**c**) Me(OMe)NH∙HCl, AlMe_3_, THF, 0 °C to rt, then **15**, −40 °C to rt, 96%; (**d**) NaH, MeI, THF/DMF (3:1), 0 °C, 95%; (**e**) MeMgBr, THF, −20 °C to −5 °C, 93%; (**f**) Me(OMe)NH∙HCl, MeMgCl, THF, −10 °C to rt, 72%; (**g**) (**i**) **27**, NEt_3_, (+)-DIPCl, CH_2_Cl_2_, −78 °C, then **26**, −78 °C to −18 °C; (**ii**) H_2_O_2_, phosphate buffer (pH 7), MeOH, 0 °C to rt, 58%, *dr* 14.8:1; (**h**) NMe_4_BH(OAc)_3_, CH_3_CN/AcOH (2:1), −40 °C to rt, 92%, single isomer; (**i**) TIPSOTf, 2,6-lutidine, 3 Å molecular sieves, CH_2_Cl_2_, −78 °C, 91%. DIPCl, B-chlorodiisopinocampheylborane; Tf, trifluormethansulfonyl; TIPS, triisopropylsilyl.

In light of the disappointing findings on the Mukaiyama aldol reaction, we turned our attention to the possible construction of the C15 stereocenter (rhizoxin numbering, see Scheme 1) by a Paterson aldol reaction [26] between aldehyde **26** and methyl ketone **27** (Scheme 4). The successful Paterson aldol reaction between **27** and a more complex aldehyde comprising the C(3)–C(13) segment of rhizoxin had been reported by White and co-workers as part of their synthesis of rhizoxin D [29].

Aldehyde **26** was readily accessible from ester **19** by reduction to the alcohol and subsequent oxidation of the latter with activated MnO_2_ (Scheme 4). Methyl ketone **27** could be prepared in 3 steps from aldol product **15** (*cf*. Scheme 2) as described by White [29] (with improved conditions for the methylation step) in 85% overall yield. Alternatively, ester **16** (cf. Scheme 2) could be converted into **27** by one-pot Weinreb amide ketone synthesis in 72% yield.

Satisfyingly, the reaction of the boron enolate of ketone **27** (formed with NEt_3_ and (+)-DIPCl in dichloromethane) with aldehyde **26** afforded β-hydroxy ketone **28** in 58% yield with a *dr* of 14.8:1. While the aldol product was inseparable from the isopinocampheol formed upon oxidative workup, the latter did not interfere with the subsequent reaction step and could then be easily removed by chromatography. Importantly, a control reaction with (−)-DIPCl proceeded without significant stereoselectivity (*dr* 1.3:1), thus indicating that the combination of α,β-chiral ketone **27** and (−)-DIPCl represents a mismatched case for the Paterson aldol reaction. Reduction of β-hydroxy ketone **28** with NMe_4_BH(OAc)_3_ [35] gave diol **29** in 92% yield as a single isomer (Scheme 4). While attempts at the regioselective TBS protection of the C(13)-OH group (rhizoxin numbering) returned only mixtures of regioisomers, treatment of **29** with TIPS-OTf and 2,6-lutidine at −78 °C gave silyl ether **30** in 91% yield as a single regioisomer.

### 2.3. Synthesis of Acid 6

**Scheme 5:**
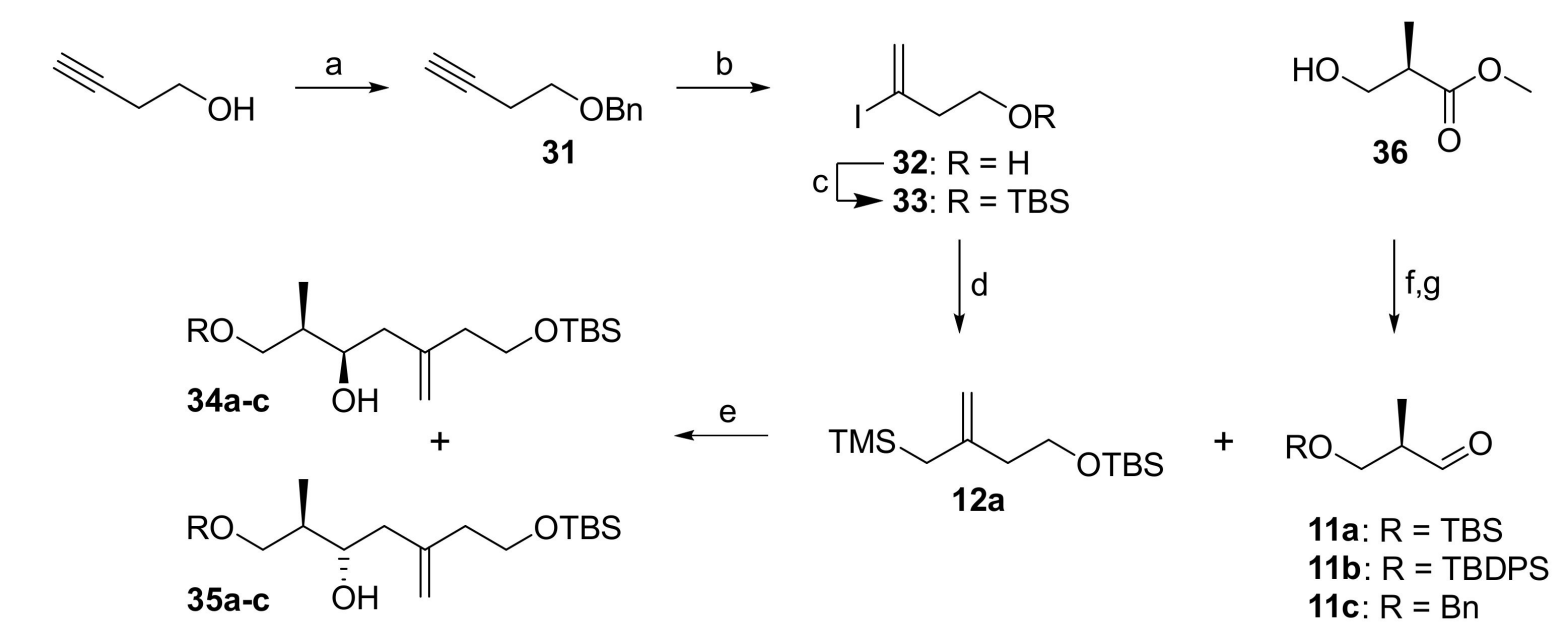
Reagents and conditions: (**a**) NaH, BnBr, TBAI, THF, 0 °C to rt, quant.; (**b**) TMSCl, NaI, H_2_O (0.6 equiv), MeCN, 0 °C to rt, 40%, 96% pure; (**c**) TBSCl, imidazole, DMF, rt, 98%; (**d**) TMSCH_2_MgCl, Pd(PPh_3_)_4_ (0.5 mol%), LiCl, Et_2_O, rt, 93%; (**e**) **34/35a** (R = TBS): (**i**) **12a** (2.1 equiv), 4 Å molecular sieves, SnCl_4_ (2.0 equiv), CH_2_Cl_2_, −78 °C; (**ii**) **11a** (1.0 equiv), CH_2_Cl_2_, –78 °C, 71% (2 steps, including step g)), **34a/35a** = 1.7:1; **34/35b** (R = TBDPS): **11b** (1.0 equiv), **12a** (2.2 equiv), TiCl_4_ (1.1 equiv), CH_2_Cl_2_, −78 °C; 37% (2 steps, including step g)), **34b**/**35b** = 1.74:1; **34**/**35c** (R = Bn): **11c** (1.0 equiv), **12a** (2.6 equiv), TiCl_4_ (1.4 equiv), CH_2_Cl_2_, −78 °C, 50% (2 steps, including step g)), **34c**/**35c** = 1:3.3; (**f**) a: TBSCl, imidazole, CH_2_Cl_2_, rt, 99%; b: TBDPSCl, imidazole, DMF, rt, 92%; c: benzyl 2,2,2-trichloroacetimidate, TfOH (cat.), CH_2_Cl_2_/cyclohexane (1:1), rt, 77%; (**g**) DIBAL (1.05 equiv), CH_2_Cl_2_, −78 °C, crude. TBAI, tetra-*n*-butylammonium iodide; TBS, *tert*-butyldimethylsilyl; TBDPS, *tert*-butyldiphenylsilyl; TMS, trimethylsilyl.

The synthesis of acid building block **6** at an early stage of our work involved exploration of the stereoselective allylation of an aldehyde **11** with allylsilane **12a** in a Hosomi-Sakurai reaction [27]. As depicted in Scheme 5, the preparation of allylsilane **12a** commenced with the benzylation of commercially available 3-butynol with BnBr/NaH, which delivered benzyl ether **31** in quantitative yield. Hydroiodination of **31** with in situ formed HI (from TMSCl, NaI and water) [36] in the presence of 0.6 equivalents of water at 0 °C led to concomitant loss of the benzyl group and gave the free alcohol **32** in 40% yield. These optimized hydroiodination conditions do not generate more than 0.6 equivalents of HI, thereby effectively preventing diiodination.

Hydroiodination was also possible with free butyn-3-ol, but the desired iodo alcohol **32** was only obtained in 22% isolated yield. Attempts to (re)protect **32** as a benzyl ether under basic conditions (BnBr, NaH) predominantly led to elimination and gave an inseparable mixture of the desired product and benzyl ether **31** in a combined yield of 41%. Attempts to conduct the benzylation under neutral (Dudley’s reagent [37]) or acidic conditions (benzyl trichloroacetimidate/catalytic acid [38]) resulted in complex reaction mixtures, from which the desired product could not be purified to homogeneity. In contrast, treatment of **32** with TBSCl cleanly furnished TBS-ether **33** in 98% isolated yield. Subsequent cross-coupling with TMS-methylmagnesium chloride either under Negishi [39] or Kumada [40,41] (Ni and Pd) conditions generally worked well. In particular, the use of Pd(PPh_3_)_4_ and LiCl gave excellent yields of allyl silane **12a** at very low catalyst loadings (down to 0.5 mol%) even on large scale. Allylsilane **12a** was found to be highly acid sensitive and prone to protodesilylation. Thus, the eluent for silica gel chromatography was buffered with 0.5% of NEt_3_ and deuterated benzene was employed as the NMR solvent instead of chloroform. These conditions effectively prevented degradation of **12a** during purification and analysis.

The Hosomi-Sakurai reaction between **12a** and aldehydes **11** was then investigated for a range of Lewis acids. As expected, the non-coordinating aldehydes **11a** and **11b** gave the desired Felkin-Anh (*syn*) products **34a** or **34b**, respectively, preferentially, but yields were generally low to moderate and even in the most favorable case (aldehyde **11a** in combination with BF_3_•Et_2_O) the *dr* did not exceed 4:1. As expected, benzyl-protected aldehyde **11c** reacted with chelation control, thus delivering the *anti* product **35c** in excess.

**Scheme 6:**
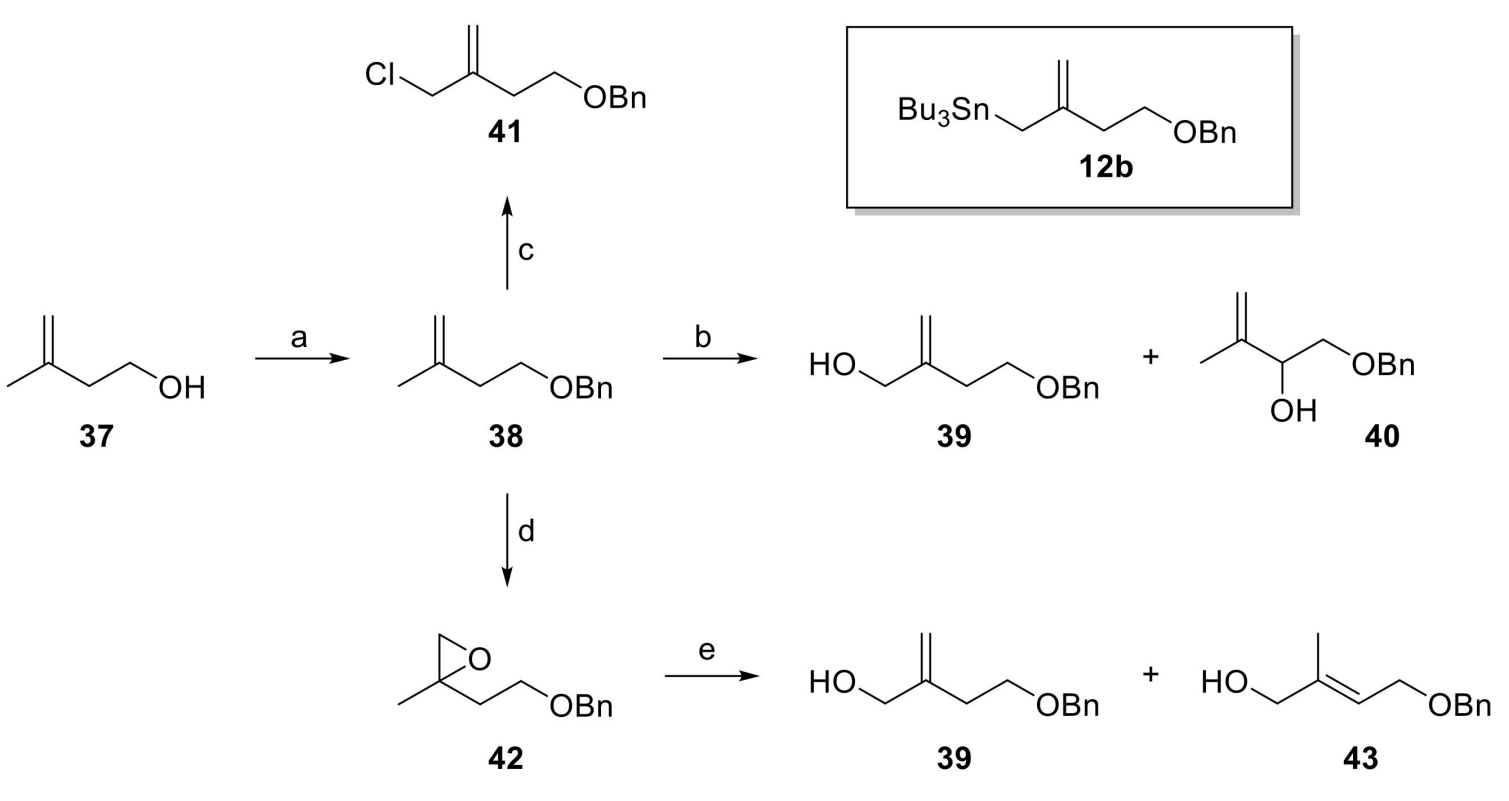
Reagents and conditions: (**a**) NaH, BnBr, TBAI, THF, 0 °C to rt, 97%; (**b**) (**i**) SeO_2_ (10 mol%), *t*BuOOH, CH_2_Cl_2_, rt; (**ii**) NaBH_4_, MeOH, 0 °C, **39**: 23%, 38% brsm, **40**: 28%; (**c**) CeCl_3_·7H_2_O, NaClO, CH_2_Cl_2_/H_2_O (1:1), rt, 58% + inseparable regioisomers; (**d**) *m*CPBA, CHCl_3_, rt, 95%; (**e**) TMPAlEt_2_, toluene, 0 °C, **39**: 48%, **43**: 16%. TBAI, tetra-*n*-butylammonium iodide; TMP = 2,2,6,6-tetramethylpiperidine. Brsm, based on recovered starting material.

Rather than trying to improve the selectivity of the Sakurai reaction by using chiral Lewis acid as, for example, developed by Yamamoto [42], we considered it more promising to assess the stereoselective Keck-type allylation [28] of aldehydes **11** with allyl stannane **12b**. Different precursors were envisioned for the preparation of **12b** by reaction with tributyltin chloride, including benzyl ether **38** (via the derived anion), allylic alcohol **39** (e.g., via the corresponding mesylate) or allylic chloride **41**; in principle, **38** should also serve as a precursor for **39** and **41** (Scheme 6). Benzyl ether **38** was readily accessible from 2-methylbut-1-en-4-ol (**37**) in excellent yield (97%) by benzylation with benzyl bromide under basic conditions. Unfortunately, however, deprotonation of **38** with *n*BuLi/TMEDA [43] or Schlosser’s base [44,45] followed by quenching of the resulting anion with tributyltin chloride failed to yield allyl stannane **12b**. Allylic chlorination [46] of **38** was non-selective and the desired isomer **41** could not be separated from isomeric impurities. In contrast, Riley oxidation [47] furnished a separable mixture of allylic alcohols **39** and **40**, from which the desired isomer **39** could isolated in 23% yield; **40** was obtained in 28% yield.

As an alternative to direct allylic hydroxylation, we also explored the regioselective β-elimination from *gem*-disubstituted epoxide **42** by means of a sterically hindered aluminium amide base, following an approach developed by Brückner [48]. However, while the treatment of the TBS-protected variant of **42** with TMPAlEt_2_ has been reported to yield the allylic alcohol corresponding to **39** in high yield as a single isomer [48], epoxide **42** furnished a ~3:1 mixture of allylic alcohols **39** and **43**, which were isolated in 48% and 16% yield, respectively. Thus, while allylic alcohol **39** could be accessed from **38** as a single isomer, the yield was far from satisfactory.

**Scheme 7:**
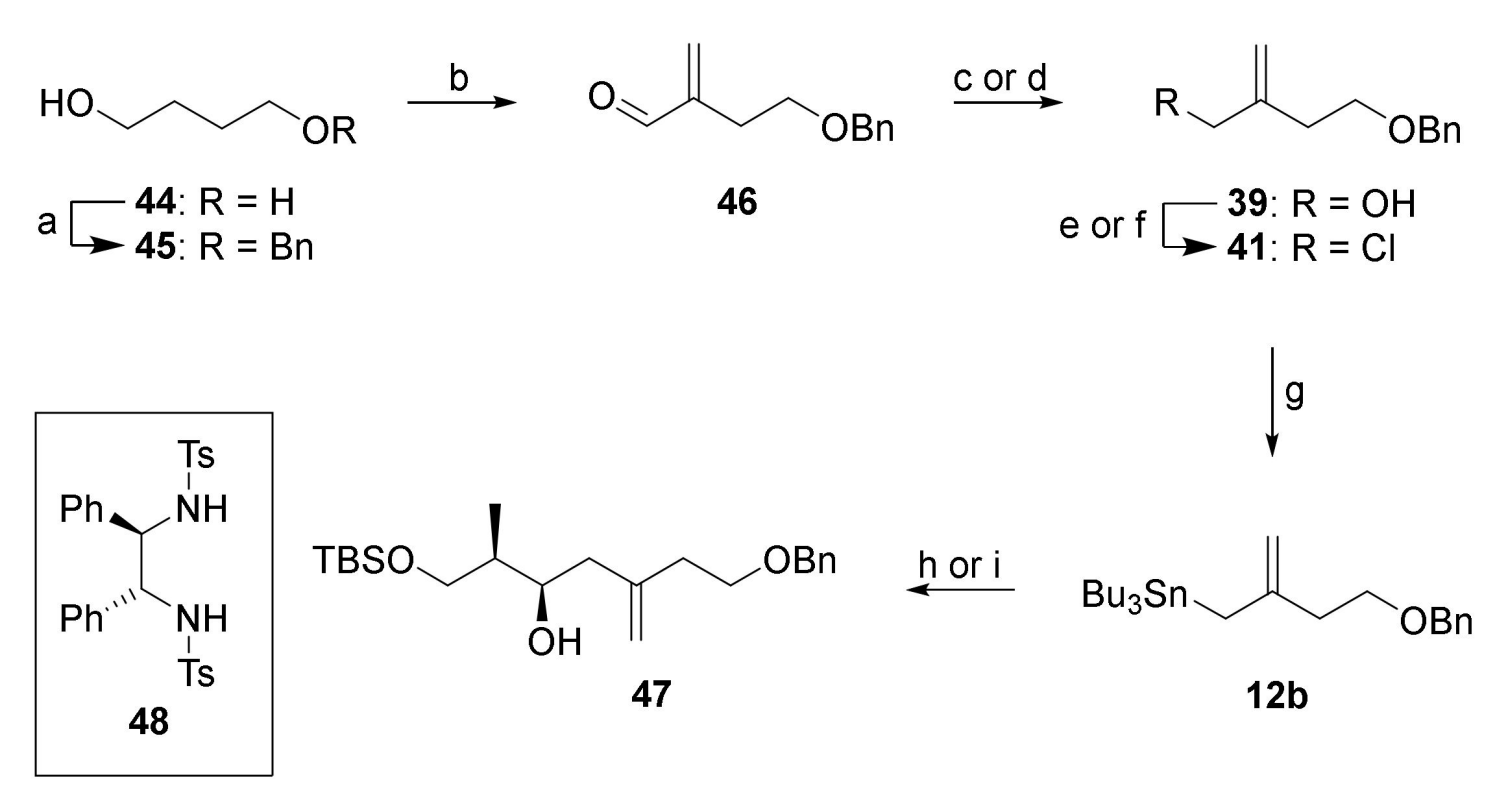
Reagents and conditions: (**a**) NaH, BnBr, THF, 0 °C to rt, 15 h, 97%; (**b**) (COCl)_2_, DMSO, NEt_3_, CH_2_Cl_2_, −78 °C to 0 °C, 2 h, then CH_2_NMe_2_Cl, DBU, rt, 22 h, 86%; (**c**) NaBH_4_, EtOH, 0 °C, 1 h, 94%; (**d**) LiAlH_4_, THF, 0 °C, 96%; (**e**) MsCl, 2,6-lutidine, LiCl, DMF, 0 °C to rt, 5 h, 88%; (**f**) CCl_4_, PPh_3_, MeCN, rt, 2 h, 95%; (**g**) Mg, Bu_3_SnCl, THF, ultrasound, 0 °C, 2h, then dibromoethane (cat.), refl., then rt, overnight, quant., 80% purity; (**h**) **11a**, BF_3_·OEt_2_, CH_2_Cl_2_, −78 °C, 1 h, 65% (*dr* 1.9:1); (**i**) (**i**) BBr_3_, ligand **48**, CH_2_Cl_2_, 0 °C to rt, 1 h, (**ii**) **12b**, 0 °C to rt, 16 h, (**iii**) **11a**, −78 °C, 2 h, 74% (2 steps) (*dr* 10:1). DBU, 1,8-diazabicyclo [5.4.0]undec-7-ene; Ms, mesyl.

In light of the difficulties to access **39** directly from **38**, an alternative route was developed from 1,4-butandiol (**44**) which delivered **39** with higher overall efficiency and without the need for isomer separation. As depicted in Scheme 7, monobenzylation of **44** followed by Swern oxidation furnished an aldehyde that was α-methylenated in situ (CH_2_NMe_2_Cl, DBU), employing a modification of a procedure originally developed by Ogasawara and co-workers [49]. The resulting α-methylene aldehyde **46** was obtained in 86% yield. Reduction of **46** with LiAlH_4_ gave alcohol **39**, which was submitted to Appel reaction to furnish allylic chloride **41**. In initial experiments, **46** was converted into **41** by NaBH_4_ reduction followed by Finkelstein reaction, but this approach proved to be inconvenient on larger scale, due to the use of DMF and EtOH in large quantities.

Conversion of chloride **41** into stannane **12b** was then achieved by Barbier-type reaction of **41**, Mg turnings, and tributyltin chloride with ultrasound irradiation (quantitative yield, 80% purity by ^1^H-NMR analysis) [50]. The purity of the material obtained after simple extractive work-up was sufficient for the next step.

The BF_3_•OEt_2_-mediated allylation of aldehyde **11a** with stannane **12b** gave alcohol **47** only with poor diastereoselectivity (*dr* 1.9:1). However, transmetalation of **12b** with bromoborane complex (prepared from BBr_3_ and bis-tosylated (*R,R*)-1,2-diphenylethylenediamine ((*R,R*)-DPEN) (**48**) [51] under conditions elaborated by Williams et al. [52] and subsequent reaction with **11a** at −78 °C furnished secondary alcohol **47** in very good yield and with high diastereoselectivity (74% over 2 steps, *dr* 10:1, 94% recovery of **48**). The configuration of the newly established stereocenter in **47** was confirmed by Mosher ester analysis [53].

**Scheme 8:**
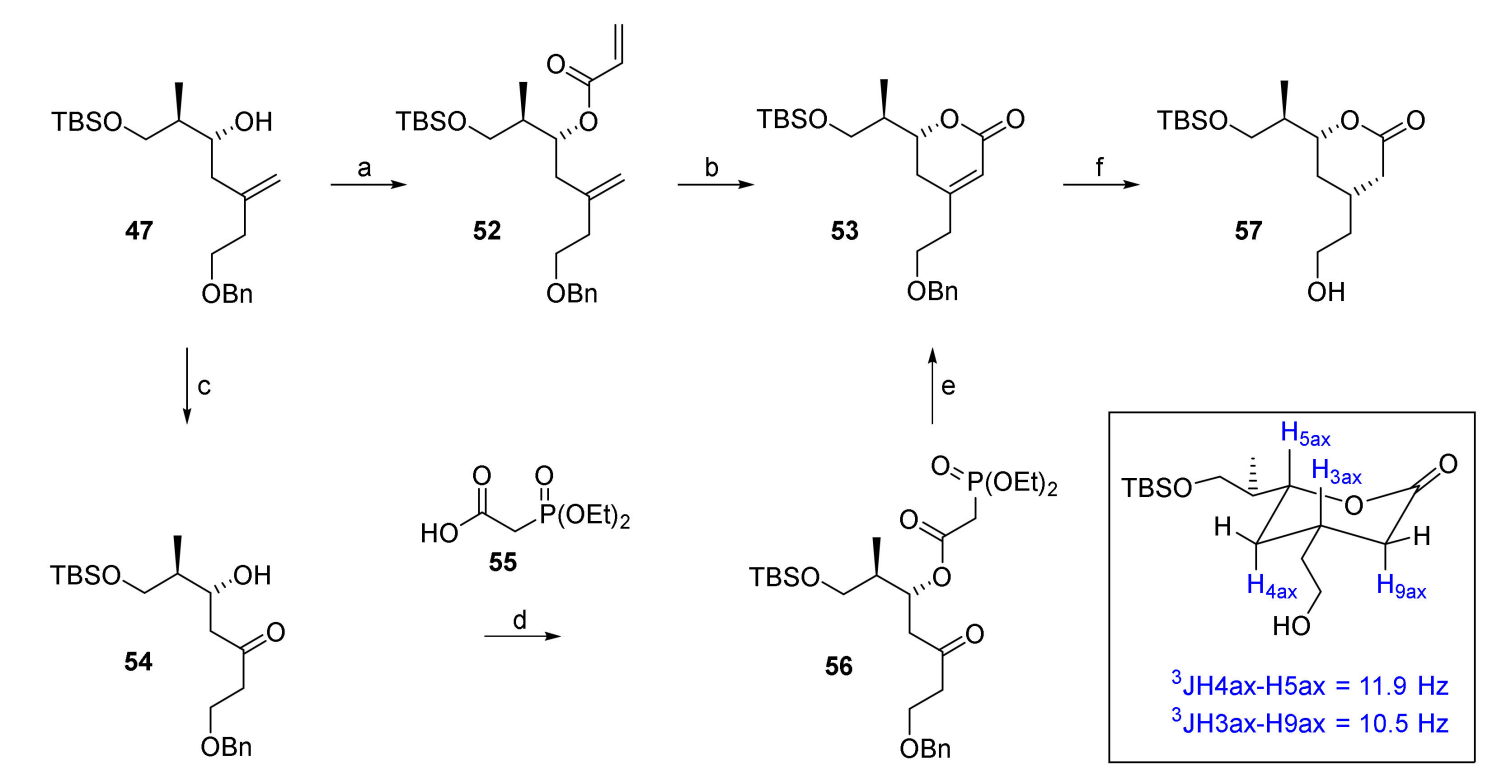
Reagents and conditions: (**a**) Acryloyl chloride, DIPEA, CH_2_Cl_2_, −50 °C, 85%; (**b**) Hoveyda-Grubbs 2nd generation catalyst (14 mol%), DCE, reflux, 89%; (**c**) OsO_4_ (cat.), NaIO_4_, 2,6-lutidine, dioxane/water, rt, 94%; (**d**) **55**, CME-CDI, DMAP, CH_2_Cl_2_, 0 °C to rt, 93%; (**e**) NaH, THF, 0 °C, 83%; (**f**) H_2_ (9 bar), Pd(OH)_2_/C (5 mol%), EtOAc, rt, 98%. CDE-CDI = *N*-cyclohexyl-*N*′-(β-[*N*-methyl-morpholino]ethyl) carbodiimide *p*-toluenesulfonate; DMAP, 4-(dimethylamino)pyridine.

Esterification of **47** with acryloyl chloride afforded diene **52** in high yield (85%) as a single isomer (Scheme 8). RCM of **52** with 10 mol% of Hoveyda-Grubbs 2nd generation catalyst in refluxing dichloroethane delivered dihydropyrone **53** in an excellent 89% yield on small scale (44 mg of **52**). However, the reaction was found to be rather unreliable on multigram scale, where it frequently did not proceed beyond 50% conversion, thus requiring multiple recyclings of the starting diene to achieve acceptable yields. Moreover, dimerization via the more reactive monosubstituted double bond started to compete with ring formation over time. Although this pathway could be partially suppressed by using higher dilution, this requirement imposed restrictions on scale-up. Therefore, an alternative three-step synthesis of **53** from **47** was elaborated that was based on an intramolecular HWE reaction for 6-membered ring formation, similar to what had been reported by Regan and co-workers as part of their synthesis of the C(1)–C(9) fragment of rhizoxin [54].

Thus, alkene **47** was subjected to Lemieux-Johnson oxidation, to furnish β-hydroxy ketone **54** in excellent 94% yield. In comparison, ozonolysis led to lower yields (76–84%) and the formation of inseparable side products (~5–10%). Steglich esterification of **54** with diethylphosphonoacetic acid (**55**) yielded phosphono ester **56**, which underwent intramolecular HWE reaction upon treatment with NaH, to yield the desired dihydropyrone **53** in 83% yield. The stereoselective hydrogenation of the double bond in **53** with Pearlman’s catalyst, according to the procedure reported by *Regan* [54] proceeded with concomitant cleavage of the benzyl ether moiety, thus furnishing lactone **57** as a single isomer in 98% yield. The large *trans*-diaxial coupling constants of 11.9 Hz and 10.5 Hz in the ^1^H-NMR spectrum of **57** (Scheme 8) confirmed the *cis*-substitution of the δ-lactone ring.

**Scheme 9:**
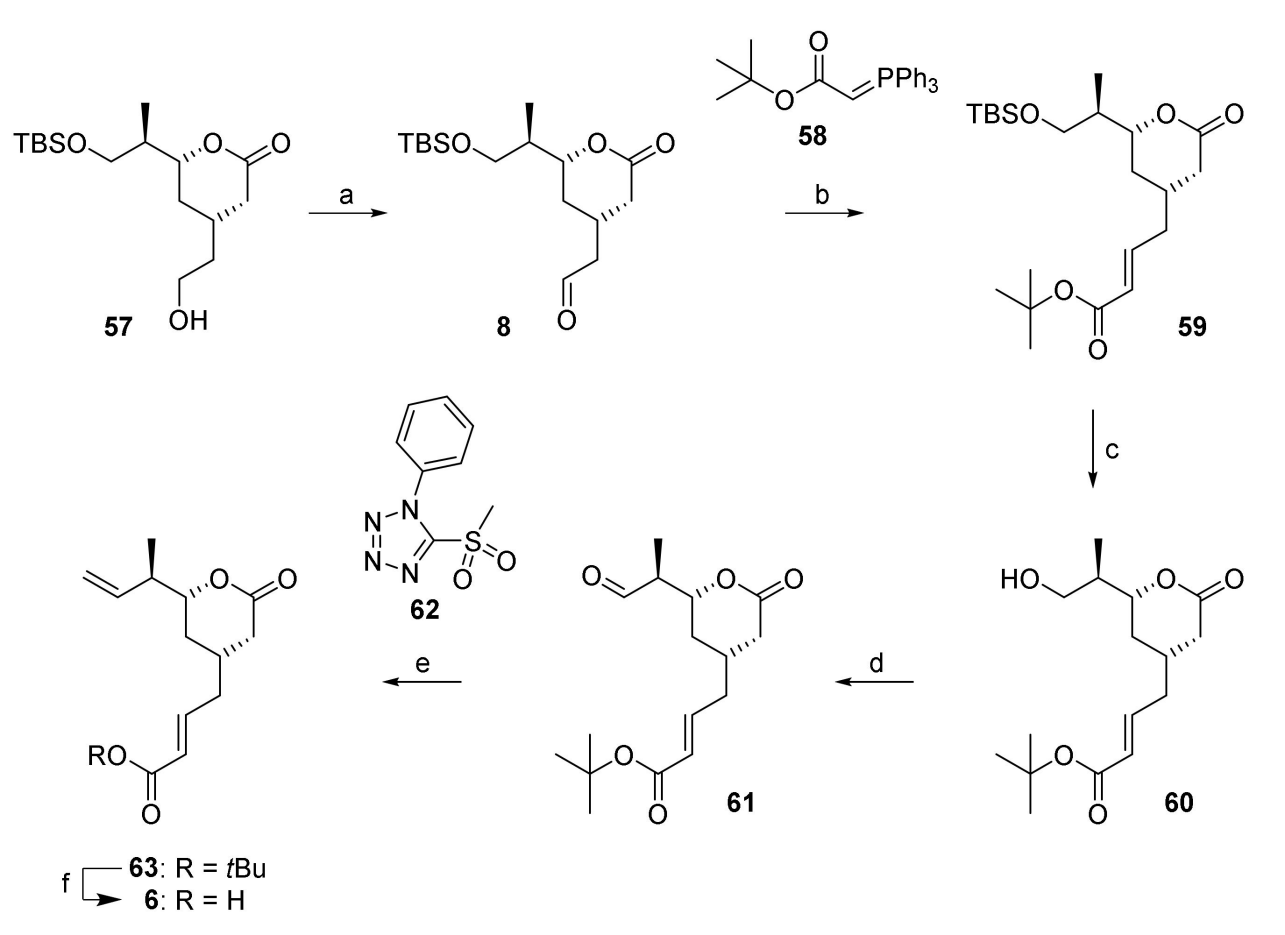
Reagents and conditions: (**a**) (COCl)_2_, DMSO, NEt_3_, CH_2_Cl_2_, −78 °C, 94%; (**b**) **58**, CH_2_Cl_2_, 0 °C to rt, 84%; (**c**) TBAF, AcOH, CH_2_Cl_2_, 0 °C to rt, 96%; (**d**) DMP, CH_2_Cl_2_, 0 °C to rt; (**e**) NaHMDS, PT-sulfone **62**, THF, −78 °C to rt, 38% (2 steps); (**f**) TFA/CH_2_Cl_2_ (*ca*. 14 equiv TFA), rt, quant. DMP, Dess-Martin periodinane (1,1,1-tris(acetyloxy)-1,1-dihydro-1,2-benziodoxol-3-(1H)-one); NaHMDS, sodium bis(trimethylsilyl)amide; PT, phenyltetrazole; TFA, trifluoroacetic acid.

The further elaboration of lactone **57** into acid building block **6** in a first stage required a two-carbon extension at the free hydroxy end that was achieved by Swern oxidation, to yield aldehyde **8**, followed by Wittig olefination with stabilized ylide **58** (Scheme 9). Ester **59** was obtained as a single isomer in 79% overall yield from **57**. In contrast to the Wittig reaction with ylide **58**, the HWE reaction of aldehyde **8** with the *t*butyl diethylphosphonoacetate gave ester **59** in much lower and highly variable yields. After installation of the carboxy functionality, completion of the carbon framework of acid building block **6** required the elaboration of the terminal double bond at the non-carboxy end of the structure. To this end, the TBS-ether moiety in **59** was cleaved with buffered (AcOH) TBAF, to afford alcohol **60** in excellent yield (provided that the acid present was not neutralized with NaHCO_3_ during work-up). After Dess-Martin oxidation [31], aldehyde **61** was isolated as a crude product after simple extractive work-up, as significant racemization on silica gel precluded chromatographic purification. Surprisingly, the conversion of the carbonyl group into the terminal double bond proved to be highly challenging. Thus, Wittig methylenation with MePPh_3_Br/KHMDS was accompanied by epimerization of the α-stereocenter and the desired olefin was only obtained in 8% yield. A whole series of alternative methods for double bond formation were then investigated, including Tebbe [55], Takai [56], Oshima-Lombardo [57], Julia [58], as well as Rh- [59] and Cu-catalyzed [60] methylenations; of these, only the Julia olefination of **61** with the phenyltetrazol sulfone **62** gave the desired olefin **63** in a preparatively useful yield of 38% (based on alcohol **60**); but the compound could only be isolated as a mixture with residual sulfone **62**. The latter was readily removed after *t*butyl ester cleavage with TFA, which gave the desired acid **6** in quantitative yield.

### 2.4. Building Block Assembly and Attempted Ring-Closing Olefin Metathesis

**Scheme 10:**
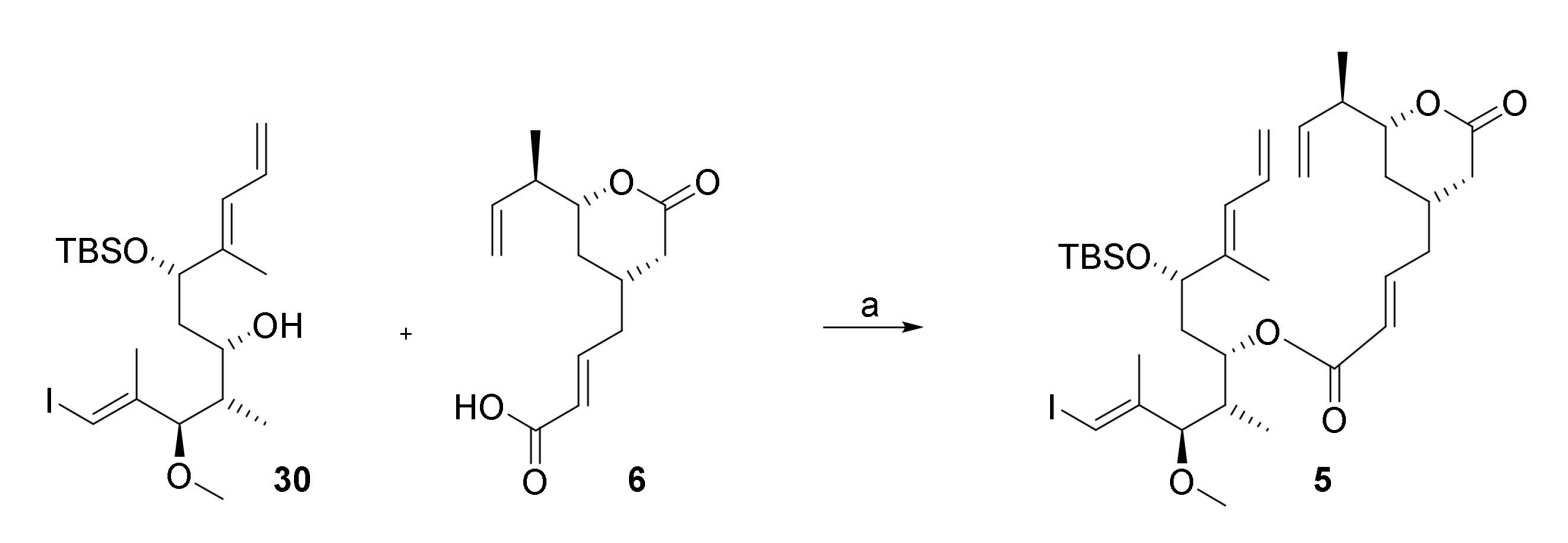
Reagents and conditions: (**i**) **6**, CME-CDI, 0 °C, CH_2_Cl_2_, molecular sieves 4 Å; (**ii**) **30**, 0 °C, then DMAP, rt; (**iii**) 0 °C, 60 h, 14% (72% **30** recovered). CME-CDI, *N*-cyclohexyl-*N*′-(β-[*N*-methyl-morpholino]ethyl) carbodiimide *p*-toluenesulfonate; DMAP, 4-(dimethylamino)pyridine.

The esterification of acid **6** with alcohol **30** was investigated with a number of different coupling agents, including EDCI/DMAP; 2,4,6-trichlorobenzoyl chloride (TCBC)/NEt_3_/DMAP, 2-methyl-6-nitrobenzoic anhydride (MNBA)/NEt_3_/DMAP, and *N*-cyclohexyl-*N*′-(β-[*N*-methyl-morpholino]ethyl) carbodiimide *p*-toluenesulfonate (CME-CDI); however, except for the use of CDI-DME, none of the desired ester **5** could be isolated (Scheme 10). In general, alcohol **30** could be recovered from the reaction mixtures in high yield (>80%), while acid **6** completely decomposed. CME-CDI gave the desired ester in a yield of 14%, which, obviously, was unacceptably low at this stage of the synthesis (Scheme 10).

**Scheme 11:**
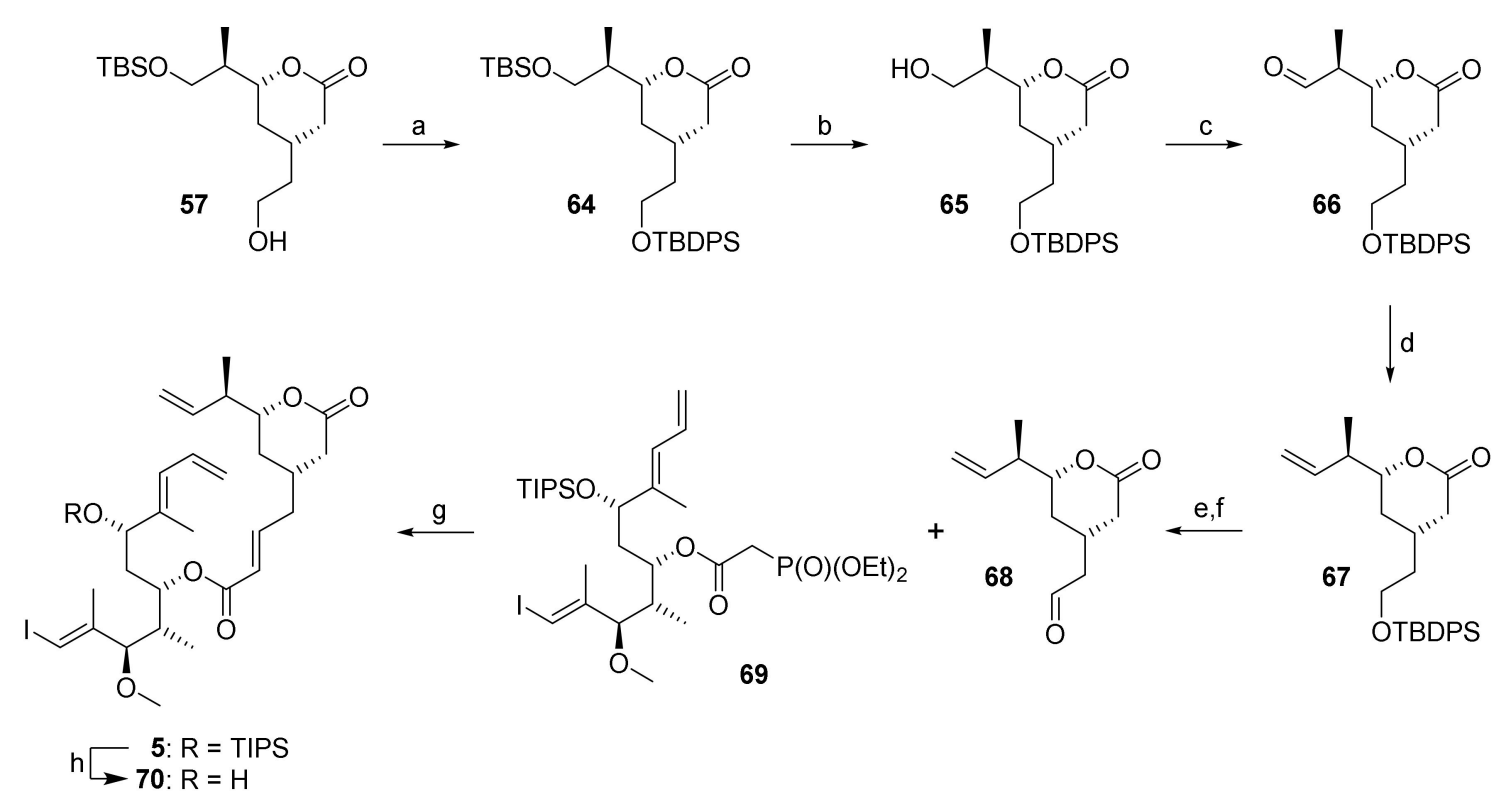
Reagents and conditions: (**a**) TBDPSCl, imidazole, DMF, rt, 95%; (**b**) NaIO_4_, THF/water (4:1), rt, 82%; (**c**) DMP, CH_2_Cl_2_, 0 °C to rt; (**d**) NaHMDS, PT-sulfone **62**, THF, −78 °C to rt, 65% (2 steps); (**e**) TBAF, AcOH, THF, 0 °C to rt, 87%, (**f**) (COCl)_2_, DMSO, NEt_3_, CH_2_Cl_2_, −78 °C, 95%; (**g**) DBU, LiCl, CH_3_CN, 0 °C, 85%; (**h**) HF•py, py, THF, 0 °C to rt, 86%. DBU, 1,8-diazabicyclo[5.4.0]undec-7-ene; DMP, Dess–Martin periodinane (1,1,1-tris(acetyloxy)-1,1-dihydro-1,2-benziodoxol-3-(1H)-one); NaHMDS. sodium bis(trimethylsilyl)amide; PT, phenyltetrazole; TBAF, tetra-*n*-butylammonium fluoride; TBDPS, *tert*-butyldiphenylsilyl.

At the same time, there was ample evidence from previous total syntheses of rhizoxins that the esterification of the C(15) hydroxy group in different rhizoxin precursors with dialkylphosphonoacetic acids was unproblematic. This observation immediately suggested that the projected RCM precursor **5** should be accessible from existing advanced intermediates that we had prepared at this point with very limited additional effort; more specifically, alcohol **30** was to be esterified with diethylphosphonoacetic acid (**55**) and the phosphono ester would then be submitted to HWE reaction with aldehyde **67** (Scheme 11). The latter would be obtained from alcohol intermediate **57** (cf. Scheme 9) by orthogonal protection of the primary hydroxy group, selective cleavage of the TBS-ether, and elaboration of the free hydroxy group into the desired double bond.

To implement this revised strategy, alcohol **57** was converted into TBDPS-ether **64** followed by selective cleavage of the TBS-ether moiety with an excess of NaIO_4_ in aqueous THF [61] to afford alcohol **65** in 78% overall yield (based on **57**) (Scheme 11). Subsequent Dess-Martin oxidation gave aldehyde **66** in low yields if purified by column chromatography, in contrast to what had been reported for a closely related aldehyde (bearing a TIPS- in place of a TBDPS-ether group) by Pattenden and co-workers [22]. The aldehyde was thus used crude in the subsequent Julia olefination with PT-sulfone **62**, which delivered the desired olefin **67** in 65% overall yield from **65**. TBAF-mediated TBDPS deprotection followed by Swern oxidation then furnished aldehyde **68**; the latter underwent smooth HWE olefination with phosphono ester **69** (obtained by CEM-CDI-mediated esterification of **30** with diethylphosphonoacetic acid (**55**)) under Masamune-Roush conditions [62] to provide the desired RCM substrate **5** in 79% overall yield from **67**.

With pentaene **5** in hand, the stage was set for the assessment of the key ring-closing olefin metathesis reaction. To this end, a number of ruthenium catalysts was assessed, including the commonly used 1st and 2nd generation Grubbs and Hoveyda-Grubbs catalysts, but also the less commonly employed Grubbs **3** and Piers-Grubbs**2**catalysts [63], as well as the slowly initiating pyridine-containing complex (sIMes)(Cl)_2_Ru(CH(CH_2_)_2_-*C*,*N*-2-C_5_H_4_N) [64] (see Table S1). Unfortunately, whenever the starting material was consumed, it was converted into an array of products. While the desired macrocycle **4** was sometimes detectable by ESI-MS, it could never be isolated.

In order to alter the properties of the RCM-substrate, the TIPS protecting group was removed from **5** by treatment with HF•py, which gave alcohol **70** in high yield (86%). Notably, if the deprotection was carried out with TBAF, **5** was mainly decomposed and the yield of **70** was poor (27%). To our disappointment, however, all attempts at ring-closing olefin metathesis with **70** were also unsuccessful under a set of standard conditions (see Table S2). As for TIPS-ether **5**, the material was either decomposed to numerous unidentified products or remained unchanged.

As the original, RCM-based synthetic plan could not be implemented, we needed to assess possible alternatives for ring-closure (excepting the commonly employed intramolecular HWE olefination at C(2)−C(3); vide supra) that would allow us to capitalize on the work expended at this point to the greatest extent possible. In this context, the concept of relay ring-closing olefin metathesis (RRCM) as developed by Hoye and co-workers presented itself as a plausible and attractive approach [65]. RRCM broadens the scope of RCM to substrates that are either sterically hindered or electronically deactivated by attaching a five-carbon tether with an easily accessible terminal double bond. The latter is intended to direct the metathesis catalyst to the site of choice via a favorable loss of cyclopentene. Our first choice was to attach the tether to the diene moiety of **5**, as this part of the substrate was presumed to display low reactivity towards metathesis catalysts.

**Scheme 12:**
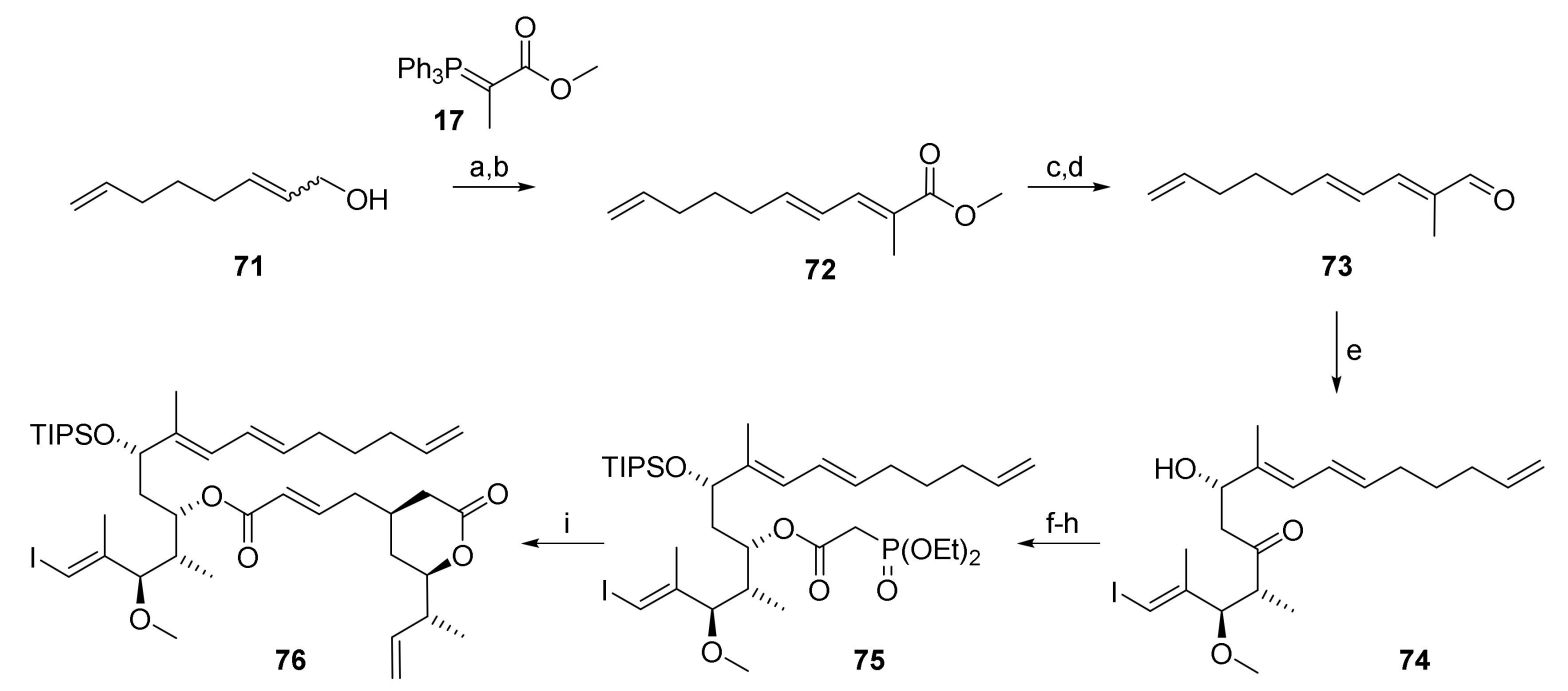
Reagents and conditions: (**a**) PCC, celite, CH_2_Cl_2_, 82%; (**b**) **17**, CH_2_Cl_2_, rf, 60% *trans* only, plus 33% *trans:cis* 4:1; (**c**) LiAlH_4_, Et_2_O, 0 °C, 94%; (**d**) activated MnO_2_, 3 Å molecular sieves, CH_2_Cl_2_, rt, 85%; (**e**) (**i**) **27**, (+)-DIPCl, NEt_3_, CH_2_Cl_2_, −78 °C, then **73**, −78 °C to −20 °C; (**ii**) H_2_O_2_, phosphate buffer (pH7), MeOH, 0 °C to rt, 68%, *dr* 7.2:1; (**f**) NMe_4_BH(OAc)_3_, CH_3_CN/AcOH (2:1), −40 °C to rt, 86%, *dr* 7.8:1 (from aldol); (**g**) TIPSOTf, 2,6-lutidine, 3 Å sieves, CH_2_Cl_2_, −78 °C, 85%; (**h**) diethylphosphonoacetic acid (**55**), CME-CDI, DMAP, 3 Å sieves, CH_2_Cl_2_, 0 °C to rt, 82%; (**i**) **68**, DBU, LiCl, CH_3_CN, 0 °C to rt, 85%. CME-CDI, *N*-cyclohexyl-*N*′-(β-[*N*-methyl-morpholino]ethyl) carbodiimide *p*-toluenesulfonate; DBU, 1,8-diazabicyclo[5.4.0]undec-7-ene; DIPCl, *B*-chlorodiisopinocampheylborane; TIPS, triisopropylsilyl.

The requisite substrate, substrate **76**, was prepared according to the same Paterson aldol-based strategy that had led to ene-diene **5** (vide supra) (Scheme 12). Thus, departing from a mixture of *cis-* and *trans* 2,7-octadienol (**71**), aldehyde **73** was obtained by PCC oxidation, Wittig olefination of the ensuing *trans*/*trans* aldehyde (obtained in 60% yield) with phosphonium ylide **17**, reduction of the resulting unsaturated ester **72** with LiAlH_4_ and oxidation of the ensuing primary alcohol with activated MnO_2_. Aldehyde **73** was obtained in 37% overall yield for the 4-step sequence from **71**. Subsequent Paterson aldol reaction of **73** with the boron enolate derived from ketone **27** gave β-hydroxy ketone **74** in 6% yield, although with lower selectivity (*dr* 7.2:1) than for reaction of **27** aldehyde **26** (*dr* 14.8:1, vide supra). Reduction of **74** followed by regioselective TIPS protection of the resulting diol gave **75**; esterification of the latter with phosphonoacetic acid (**55**) and, finally, HWE reaction of the phosphono ester with aldehyde **68** uneventfully delivered relay substrate **76** in 51% overall yield under the conditions elaborated previously.

Various conditions were then screened to effect macrocyclization. Unfortunately, however, macrocycle **4** was never obtained; instead, the tether was cleaved within seconds in an intermolecular reaction, to produce **5**, which did not undergo RCM, as had been found previously. The formation of **5** by tether cleavage from **76** was observed at concentrations as low as 1.2 μM.

In addition to the conditions for macrocyclization that had been investigated in previous screenings (see Tables S1 and S2), experiments were also conducted where **76** was added slowly to a refluxing solution of the catalyst via a syringe pump, a procedure that had been reported by *Porco* and co-workers to suppress unproductive relay cleavage [66]. However, this method proved to be unsuccessful with **76**. Likewise, microwave-assisted heating according to Robinson et al. [67] or the use of CuI as a phosphine scavenger [68], did not effect RRCM.

**Scheme 13:**
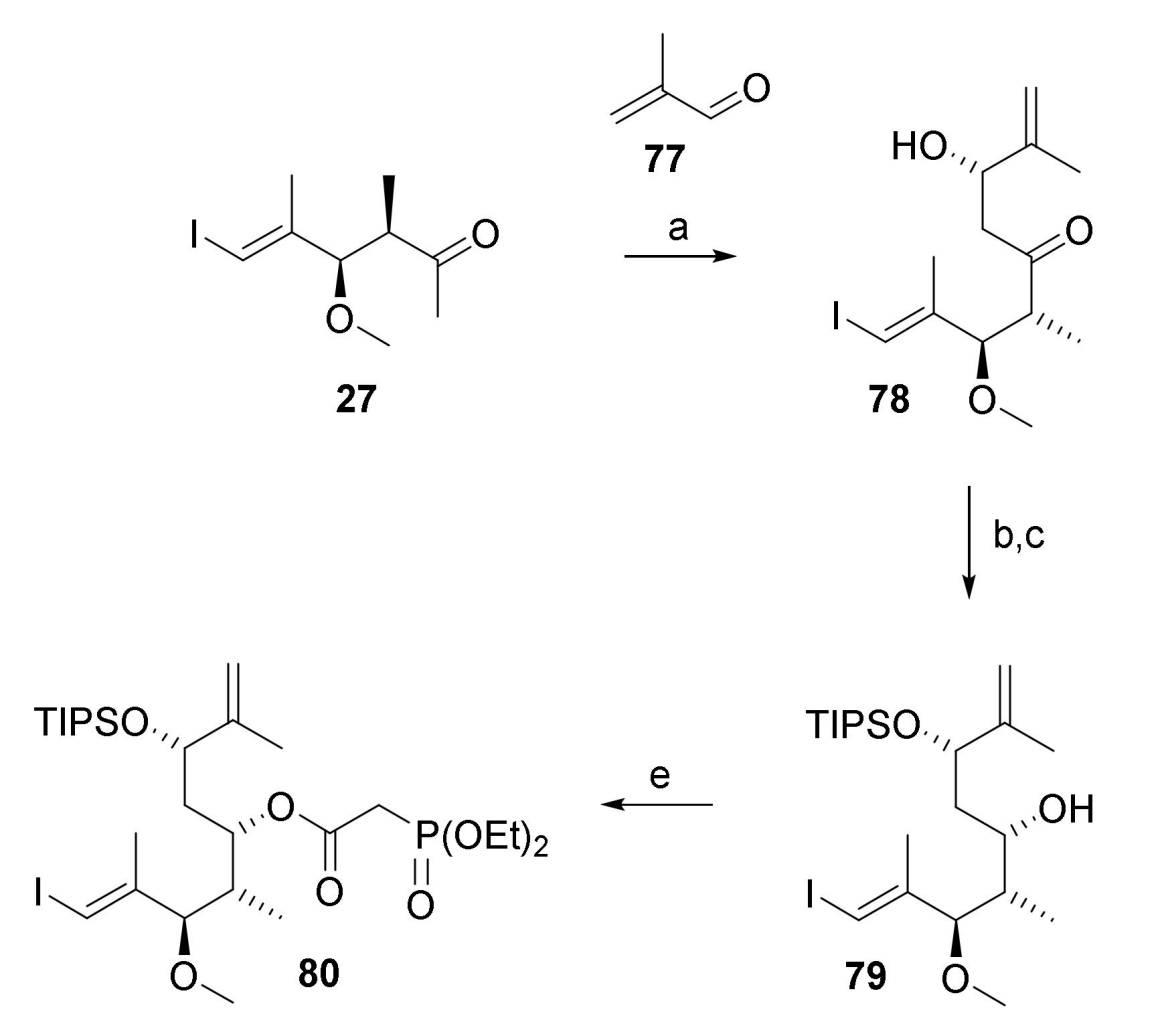
Reagents and conditions: (**i**) (+)-DIPCl, NEt_3_, CH_2_Cl_2_, −78 °C, then 77, −78 °C to −20 °C; (**ii**) H_2_O_2_, phosphate buffer (pH7), MeOH, 0 °C to rt, 67%, *dr* 19.6:1; (**b**) NMe_4_BH(OAc)_3_, CH_3_CN/AcOH (1:1), −40 °C to −18°C to rt, 95%; (**c**) TIPSOTf, 2,6-lutidine, 3 Å molecular sieves, CH_2_Cl_2_, −78 °C, 97%; d) diethylphosphonoacetic acid (**55**), CME-CDI, DMAP, 3 Å molecular sieves, CH_2_Cl_2_, 0 °C to rt, 81%. CME-CDI, *N*-cyclohexyl-*N*′-(β-[*N*-methyl-morpholino]ethyl) carbodiimide *p*-toluenesulfonate; DMAP, 4-(dimethylamino)pyridine, DIPCl, *B*-chlorodiisopinocampheylborane; TIPS, triisopropylsilyl.

The resistance of substrate **76** towards RRCM led us to consider shifting the site of ring-closure from C(9)/C(10) to C(11)/C(12) as a possible alternative approach towards macrocycle **4**. The requisite RRCM substrate was prepared based on the same overall strategy that had been followed for the synthesis of **5** and **76**. Thus, phosphono ester **80** (Scheme 13) was obtained from ketone **27** via Paterson aldol reaction with methacrolein (**77**), stereoselective reduction of the ensuing β-hydroxy ketone **78**, regioselective TIPS protection of the resulting diol product and esterification of the monoprotected diol **79** with diethylphosphono acetic acid (**55**) in 49% overall yield.

Notably, of all aldehydes submitted to Paterson aldol reaction with ketone **27**, methacrolein (**77**) gave the cleanest reaction and furnished the aldol product with the highest selectivity ((*dr* 19.6:1).

**Scheme 14:**
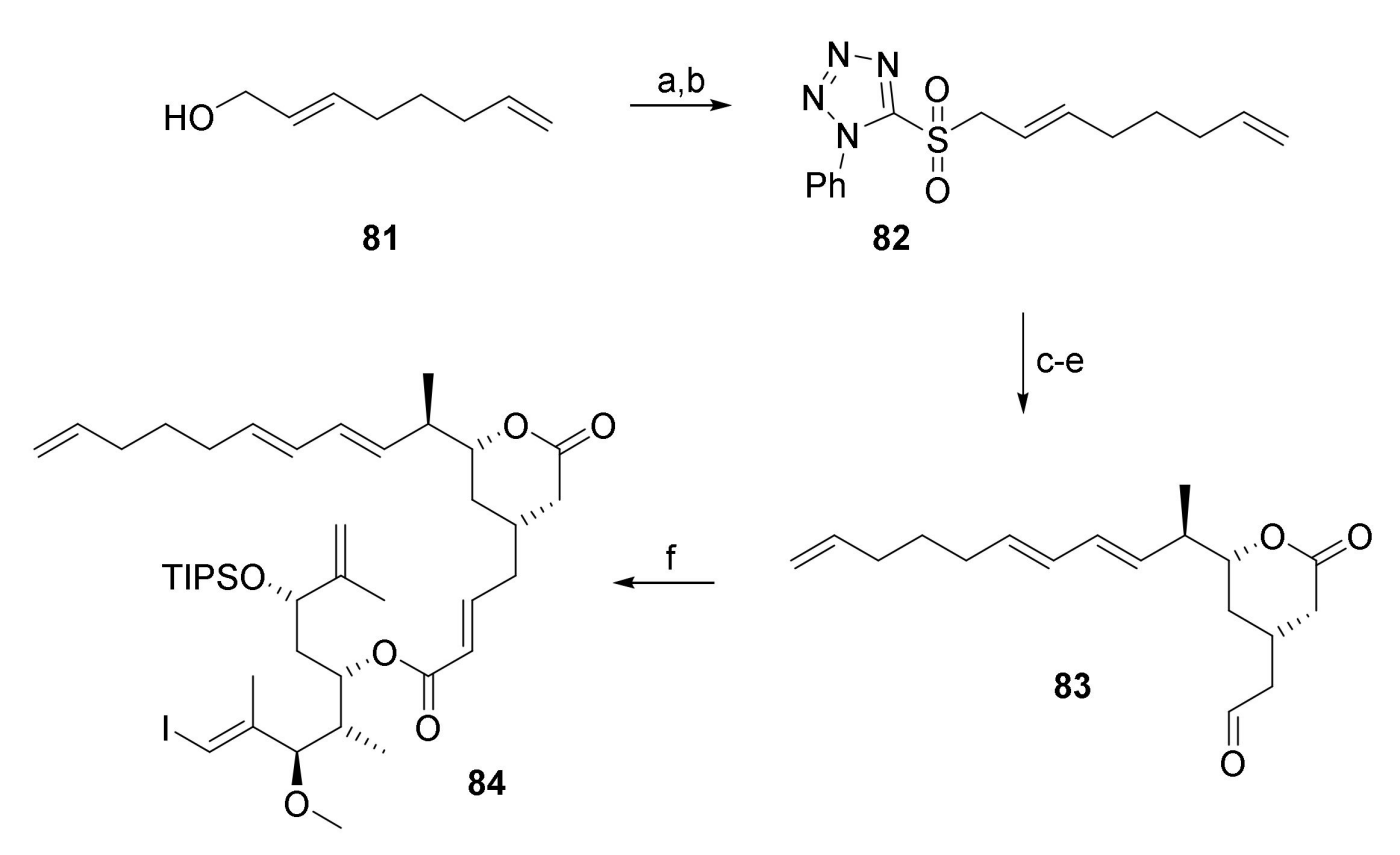
Reagents and conditions: (**a**) 2-Mercaptophenyltetrazole, PPh_3_, DEAD, THF, rt; (**b**) Mo_7_O_24_(NH_4_)_6_, H_2_O_2_, EtOH, 0 °C to rt, 57% (2 steps); (**c**) LiHMDS, aldehyde **66**, 34% (*E*/*Z* > 30:1); (**d**) TBAF, AcOH, THF, 0 °C to rt, 89%; (**e**) (COCl)_2_, DMSO, NEt_3_, CH_2_Cl_2_, −78 °C, 95%; (**f**) **80**, DBU, LiCl, CH_3_CN, 0 °C to rt, 69%. DBU, 1,8-diazabicyclo[5.4.0]undec-7-ene; DEAD, diethyl azodicarboxylate; LiHMDS, lithium bis(trimethylsilyl)amide; TBAF, tetra-*n*-butylammonium fluoride.

As shown in Scheme 14, aldehyde **83** was obtained from aldehyde **66** by Julia-Kocienski olefination with sulfone **82** (obtained from 2,7-octadienol (**81**) via Mitsunobu reaction and subsequent oxidation of the thioether with Mo_7_O_24_(NH_4_)_6_, H_2_O_2_ in 57% yield) followed by TBDPS removal and Swern oxidation of the resulting free alcohol in 30% overall yield. HWE reaction of **80** and **83** under Masamune-Roush conditions then furnished the RRCM substrate **84** in 69% yield.

Unfortunately, treatment of **84** with Grubbs 2nd generation catalyst in toluene, which was the only system that had shown some initial promise in screening experiments, did not result in macrocyclization (employing catalyst loadings of 5 mol% or 10 mol% at temperatures of 45 °C, 60 °C, or 90 °C). As for **76**, only the tether was cleaved and more and more side products appeared over time.

**Scheme 15:**
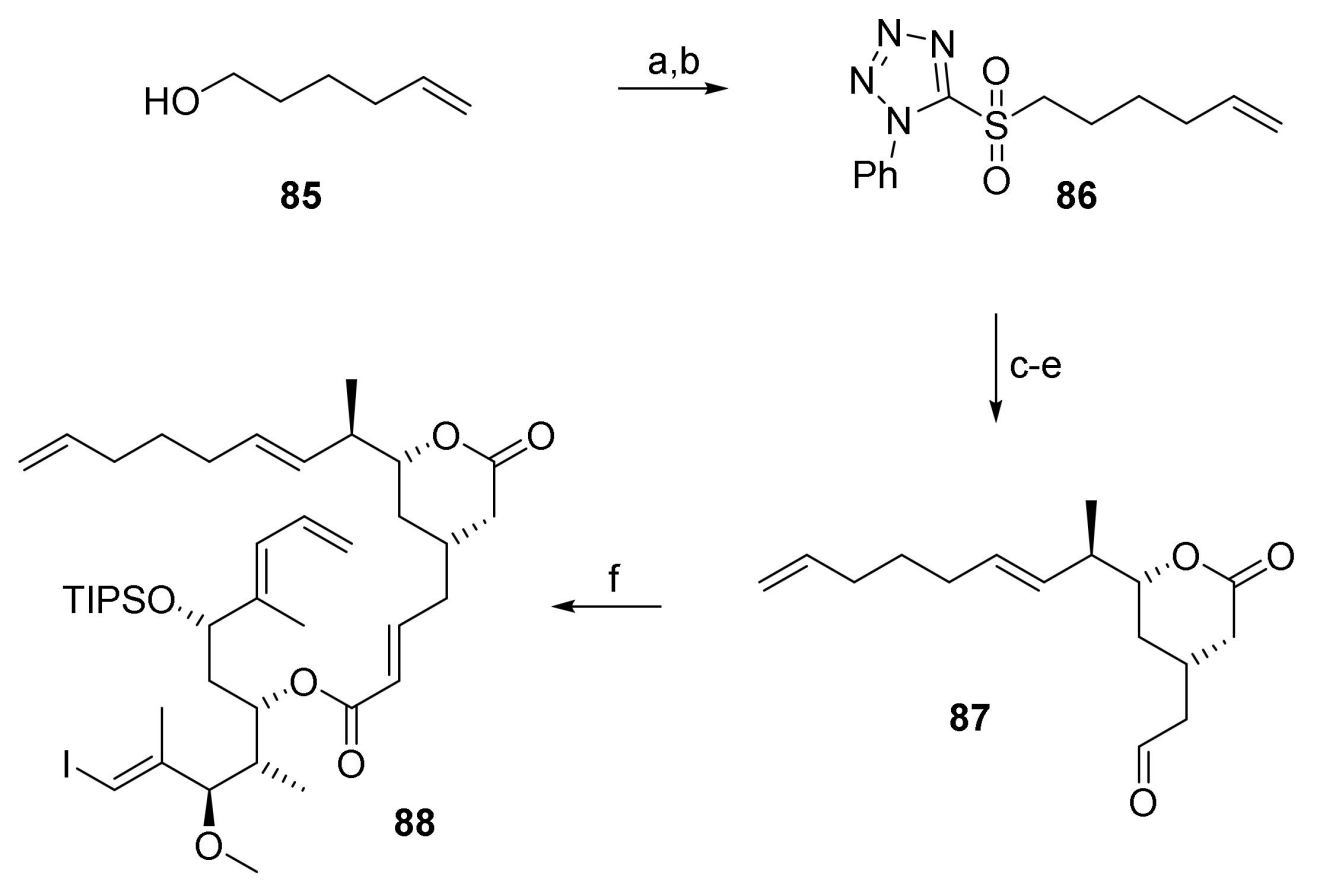
Reagents and conditions: (**a**) 2-Mercaptophenyltetrazole, DEAD, PPh_3_, THF, rt; (**b**) H_2_O_2_, (NH_4_)_6_Mo_7_O_24_, EtOH/THF (5:3), 0 °C to rt, 78% (2 steps); (**c**) NaHMDS, THF, −78 °C to −9 °C, then 66, −78 °C to 0 °C, 62%, *E* only; (**d**) TBAF, AcOH, THF, 0 °C to rt, 94%; (**e**) (COCl)_2_, DMSO, CH_2_Cl_2_, −78 °C, then NEt_3_, −78 °C to rt, 92%, *E:Z* = 9.6:1; (**f**) **87**, DBU, LiCl, CH_3_CN, 0 °C to rt, 85%. DBU, 1,8-diazabicyclo[5.4.0]undec-7-ene; DEAD, diethyl azodicarboxylate; NaHMDS, sodium bis(trimethylsilyl)amide; TBAF, *tetra*-*n*-butylammonium fluoride.

As simply shifting the position of the (projected) site of cyclization from C(9)/C(10) in **76** to C(11)/C(12) in **84** did not enable RRCM, we went back to investigate the possibility of RRCM at the originally conceived site between C(9) and C(10), but now with the tether attached to the terminal double bond of the eastern part of the original RCM substrate **5**. Directing the catalyst to this double bond was felt to offer some promise, as the experiments with RRCM substrate **76** had suggested that it did not react with a ruthenium-carbene at C10. The corresponding RRCM substrate **88** was obtained by HWE of phosphono ester **69** and aldehyde **87** under Masamune-Roush conditions (Scheme 15). The latter was prepared from the known phenyltetrazole sulfone **86** [66] via Julia-Koczienski olefination, followed by TBDPS removal and Swern oxidation.

Disappointingly, **88**, as all other previous substrates, did not undergo macrocyclization to any detectable extent for a variety of solvents and catalysts.

**Figure 2:**
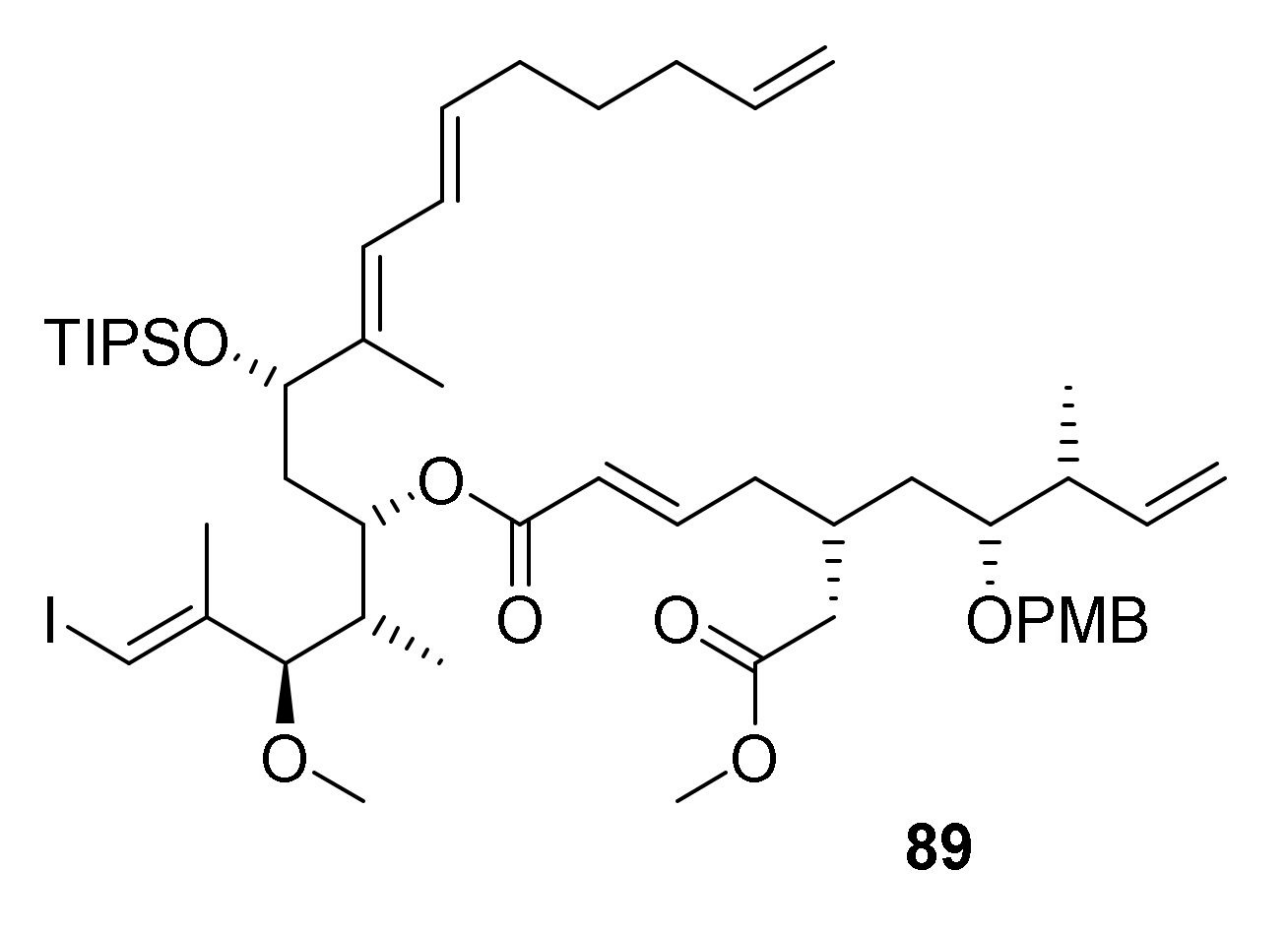
Structure of the potential RRCM substrate 89.

In light of the various unsuccessful attempts at making the rhizoxin core macrocycle either by RCM or RRCM, the question arose if specific structural features of the corresponding cyclization precursors might perhaps prevent the adoption of a favorable conformation for productive ring-closure. While it was not entirely clear what these features would be, we hypothesized that macrocylization could perhaps be promoted by a more flexible cyclization precursor that did not incorporate the δ-lactone ring that had been present in all RCM/RRCM substrates investigated up to this point. To address this question, we targeted the potential RRCM substrate **89** (Figure 2), which incorporates two additional, rotatable bonds when compared to **5**, **76**, **84**, and **88**.

**Scheme 16:**
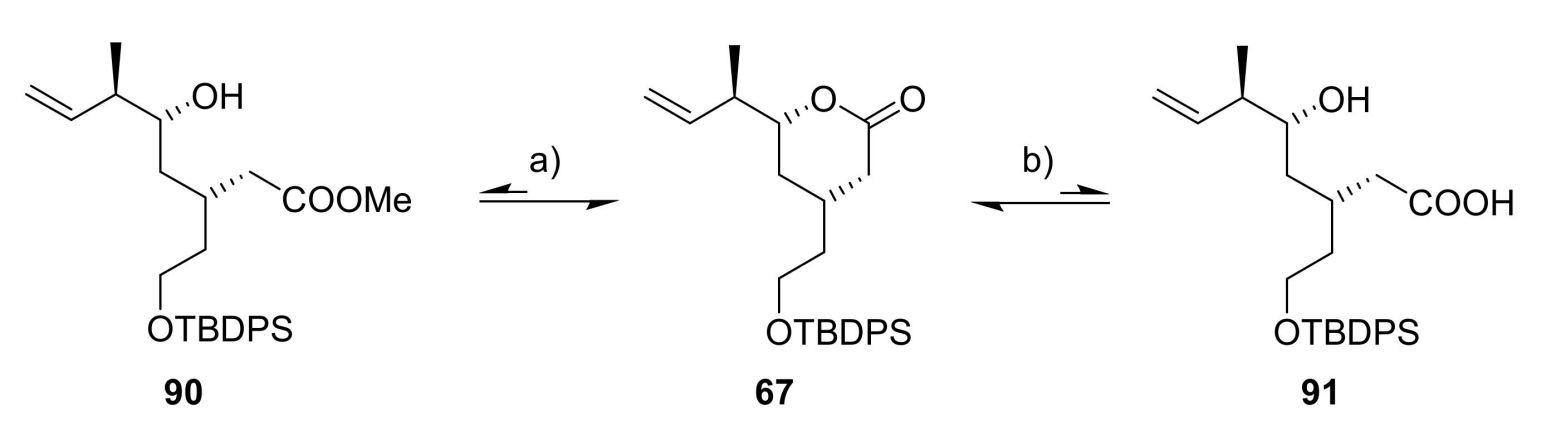
Reagents and conditions: (**a**) NaOMe, or NEt_3_, MeOH, or LiOMe; (**b**) (**i**) LiOH, THF/water (1:1), 0 °C to rt, 98%, (**ii**) TMSCHN_2_, Et_2_O/MeOH, rt, quant (**67**). TMS, trimethylsilyl.

During the initial planning phase for the synthesis of **89**, we assumed that ester **90** could serve as a suitable intermediate that would also be readily accessible from lactone **67** by simple treatment with NaOMe (Scheme 16).

However, while initial attempts using a large excess of NaOMe led to led to significant but still incomplete conversion to hydroxy ester **90** according to TLC, even neutral or slightly acidic conditions during attempted purification on silica gel or in deuterated chloroform were sufficient to shift the equilibrium back to the starting material **67**. This was also the case for the trapped lithium carboxylate of acid **91**, which immediately recyclized to δ-lactone **67** after work-up or TMS diazomethane treatment.

**Scheme 17:**
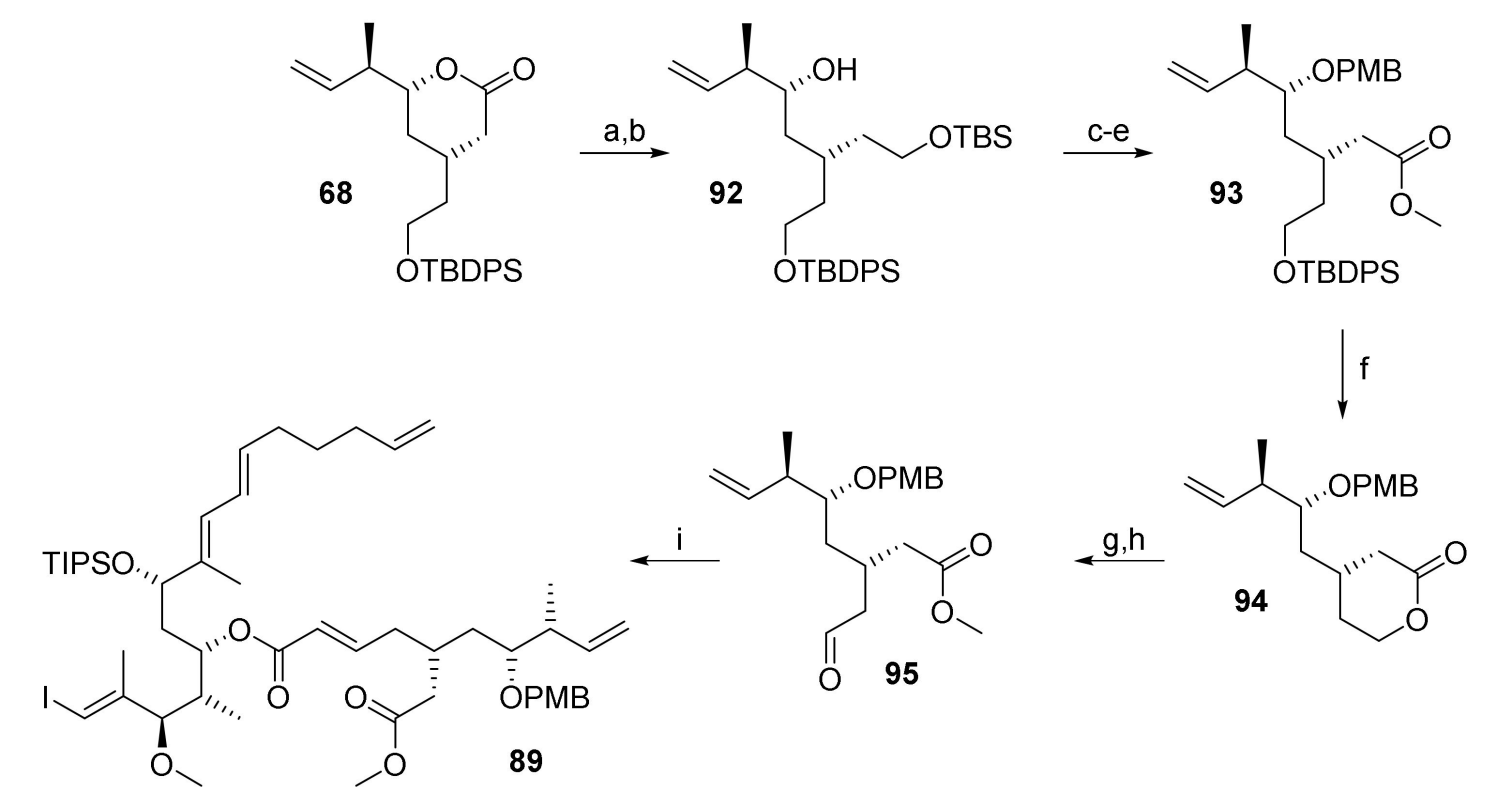
Reagents and conditions: (**a**) LiAlH_4_, Et_2_O, 0 °C to rt, quant; (**b**) TBSCl, imidazole, CH_2_Cl_2_, rt, 98% (2 steps); (**c**) Sc(OTf)_3_ (6 mol%), PMB-2,2,2-trichloroacetimidate, toluene, rt; (**d**) NaIO_4_, THF/H_2_O (4:1), rt, 67% (2 steps); (**e**) (**i**) (COCl)_2_, DMSO, CH_2_Cl_2_, −78 °C, then NEt_3_, −78 °C to rt, (**ii**) NaClO_2_, NaH_2_PO_4_, 2-methyl-2-butene, *t*BuOH, THF, rt, (**iii**) TMSCHN_2_, toluene/MeOH, 0 °C, 79% (3 steps); (**f**) TBAF, AcOH, THF, rt, 79%; g) LiOMe, MeOH, rt; (**h**) DMP, CH_2_Cl_2_, 0 °C to rt, 68% (2 steps) (93% brsm); (**i**) **75**, LiCl, DBU, THF/MeCN (3:1), 0 °C to rt, 78%. DBU, 1,8-diazabicyclo[5.4.0]undec-7-ene; DMP, Dess–Martin periodinane (1,1,1-tris(acetyloxy)-1,1-dihydro-1,2-benziodoxol-3-(1H)-one); PMB, *para*-methoxy benzyl; TBS, *tert*-butyldimethylsilyl; TMS, trimethylsilyl.

For this reason, a longer route toward **91** had to be elaborated which is summarized in Scheme 17. Thus, **67** was reduced with LiAlH_4_, to furnish a diol which was selectively TBS-protected at the primary hydroxy group in 98% overall yield (from **67**). Lewis acid-catalyzed PMB protection followed by selective TBS deprotection furnished a primary alcohol that was elaborated into methyl ester **93** in 53% overall yield in a three-step sequence involving Swern oxidation, Pinnick oxidation of the ensuing aldehyde to the acid, and methyl ester formation with TMS-diazomethane. TBDPS deprotection with buffered TBAF furnished a 9:1 mixture of lactone **94** and the corresponding hydroxy (methyl)ester in 88% combined yield. After sequential treatment of this (separable) mixture with lithium methoxide and Dess-Martin periodinane, aldehyde **95** was isolated in 68% yield (93% brsm). Subsequent HWE reaction of **95** with phosphonate **69** under Masamune-Roush conditions furnished ester **89**.

Unfortunately, as for other RRCM substrates, Grubbs 2nd and Hoveyda-Grubbs 2nd generation catalysts in dichloroethane led to efficient cleavage of the relay tether, but no macrocyclization was observed based on MS and TLC analysis of reaction mixtures. In refluxing toluene, both catalysts led to a complex mixture of products as evidenced by HPLC and NMR analysis.

### 2.5. Macrocyclization by Ring-Closing Alkyne Metathesis (RCAM)

**Scheme 18:**
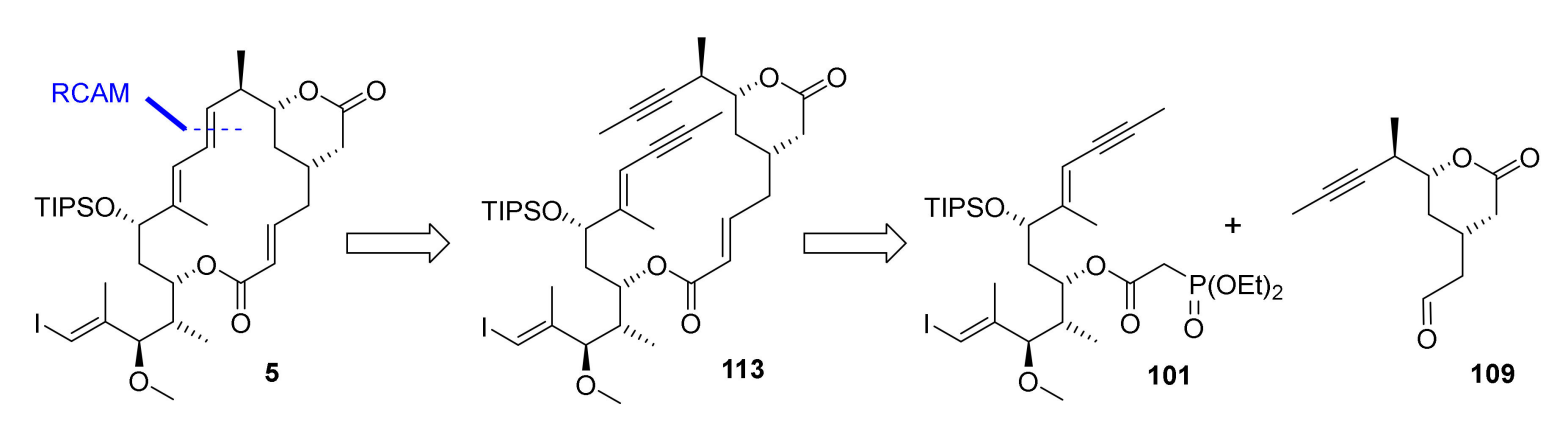
Global retrosynthesis of **5** via **113**.

At the point where our numerous attempts at synthesizing the macrocyclic portion of rhizoxin F (**2**) by means of RCM/RRCM had all met with failure, we felt that a radical change in strategy was necessary. Triggered by Fürstner’s groundbreaking work on RCAM in the field of natural product synthesis (for early examples, see in [69,70,71,72,73,74,75,76,77,78]; for more recent examples, see in [23,79,80,81], we reasoned that this method would be a promising alternative to RCM. (For a review on the use of RCAM in natural product synthesis, see in [82].) Thus, macrocyclization was planned to be achieved by RCAM at C(9)/C(10), i.e., at the same site that was initially foreseen for RCM, leading to diyne **113** as the ultimate cyclisation precursor (Scheme 18). Obviously, the triple bond in the RCAM product would have to be converted into a *trans* double bond, but methods for the *trans*-selective reduction of alkynes to olefins had been described (see, for example, in [83,84,85]).

Diyne **113** was to be obtained from phosphono ester **101** and aldehyde **109** in analogy to the synthesis of the various RCM/RRCM substrates. This approach offered the advantage that it would allow us to capitalize on the chemistry developed in the course of the synthesis of the corresponding RCM/RRCM macrocyclization precursors. The largest part of the chemistry described in the following has already been reported in [20], but is included here to provide a coherent and complete narrative of the work that finally led to the successful synthesis of rhizoxin F.

#### 2.5.1. Synthesis of Building Blocks

**Scheme 19:**
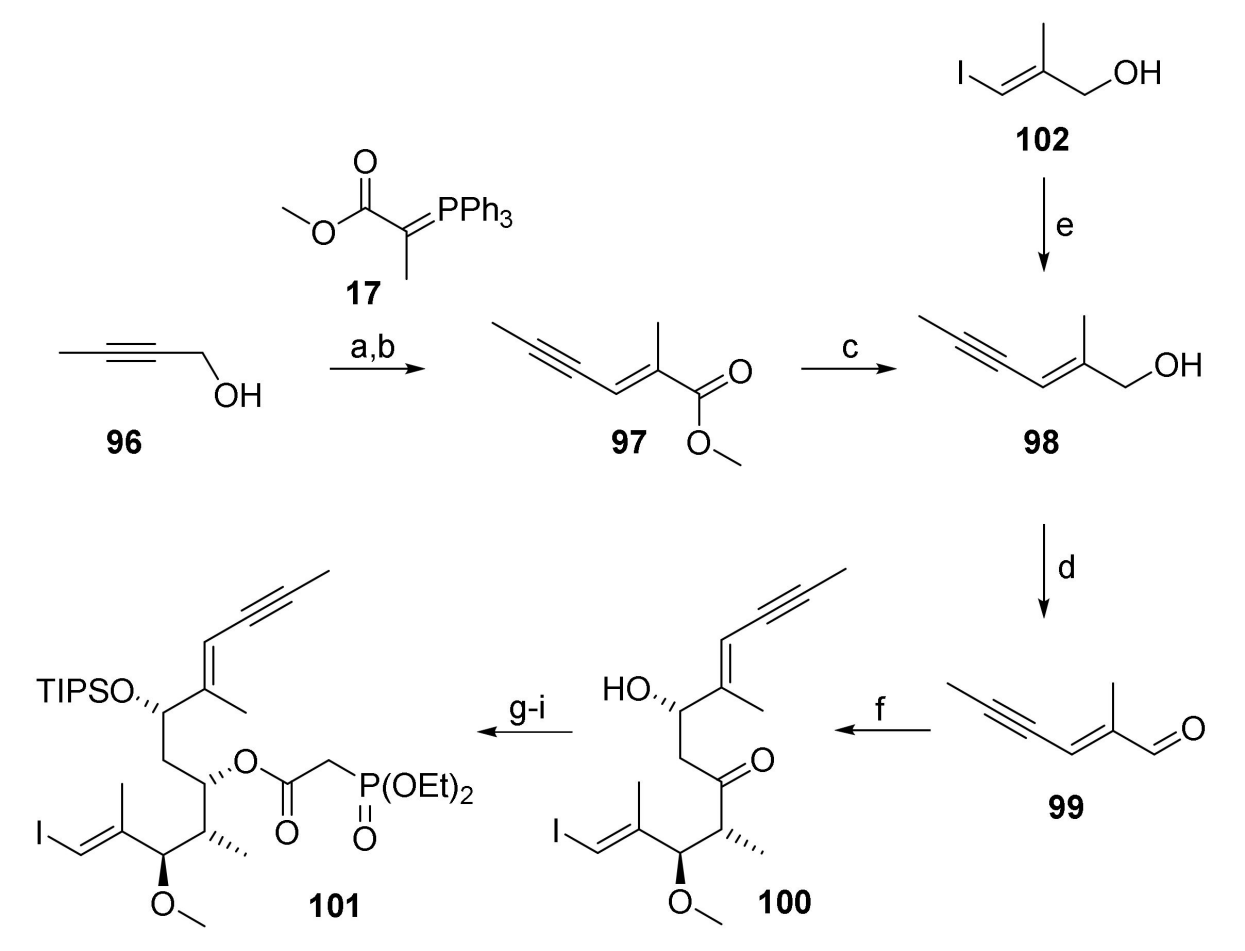
Reagents and conditions: (**a**) Activated MnO_2_, 3 Å molecular sieves, CH_2_Cl_2_, rt, 82%; (**b**) 17, CH_2_Cl_2_, rf, 45%; (**c**) LiAlH_4_, Et_2_O, 0 °C; (**d**) activated MnO_2_, 3 Å molecular sieves, CH_2_Cl_2_, rt, 82%; (**e**) propyne, DIPEA, CuI (20 mol%), PdCl_2_(PPh_3_)_2_ (15 mol%), THF, −78 °C to rt, 63%; (**f**) (**i**) 27, (+)-DIPCl, NEt_3_, CH_2_Cl_2_, −78 °C, then 99, −78 °C to −18 °C; (**ii**) H_2_O_2_, phosphate buffer (pH7), MeOH, 0 °C to rt, ca. 73%, *dr* n.d.; (**g**) NMe_4_BH(OAc)_3_, CH_3_CN/AcOH (2:1.5), −40 °C to rt, 90%; (**h**) TIPSOTf, 2,6-lutidine, 3 Å molecular sieves, CH_2_Cl_2_, −78 °C, 97%; (**i**) diethylphosphonoacetic acid (55), CME-CDI, DMAP, 3 Å molecular sieves, CH_2_Cl_2_, 0 °C to rt, 82%. DIPCl, *B*-Chlorodiisopinocampheylborane; DIPEA, *N,N*-diisopropylethylamine (Hünig’s base); TIPS, triisopropylsilyl.

As illustrated in Scheme 19, the synthesis of phosphono ester **101** relied on the same type of aldol chemistry that had been successfully exploited in the synthesis of the different RCM/RRCM substrates. In this context, a short route was devised towards the requisite aldehyde **99**, making use of vinyl iodide **102**, which was already at hand from our previous synthesis of ketone **27**. Thus, **102** was submitted to Sonogashira cross-coupling with propyne to yield ene-yne **98**, which was readily oxidized to the desired aldehyde **99** (Scheme 19).

However, the Sonogashira coupling proved to be very tedious; not only were vinyl iodide **102** and the coupling product **98** inseparable on silica gel (TLC), thus creating the need for reaction monitoring by RP-HPLC and requiring full conversion of the starting material, but the coupling also relied on the use of expensive propyne and a precious catalyst. Furthermore, it was found that the optimized conditions in small-scale experiments were not amenable to scale up (>1 g), which was a key requirement at this early stage of the synthesis. For these reasons an alternative route towards **99** was sought. In the event, oxidation of 2-butyn-1-ol (**96**) with activated MnO_2_ and subsequent *Wittig* olefination of the resulting propargylic aldehyde with phosphonium ylide **17** furnished ester **97**, which was smoothly reduced to allylic alcohol **98** (Scheme 19). In contrast to gaseous propyne, 2-butyn-1-ol (**96**) is an easy-to-handle and relatively cheap liquid. Furthermore, phosphonium ylide **77** could be conveniently synthesized in large quantities (>120 g). The synthesis of **99** from **96** could thus be scaled up without difficulties.

With aldehyde **99** in hand, the synthesis of phosphono ester **101** could be addressed, relying on previously established protocols. Thus, Paterson aldol reaction of **99** with ketone **27** followed by directed reduction of β-hydroxy ketone **100** according to Evans [35], regioselective TIPS protection of the ensuing diol, and esterification with diethylphosphonacetic acid (**55**) furnished **101** in 52% overall yield as a single isomer.

**Scheme 20:**
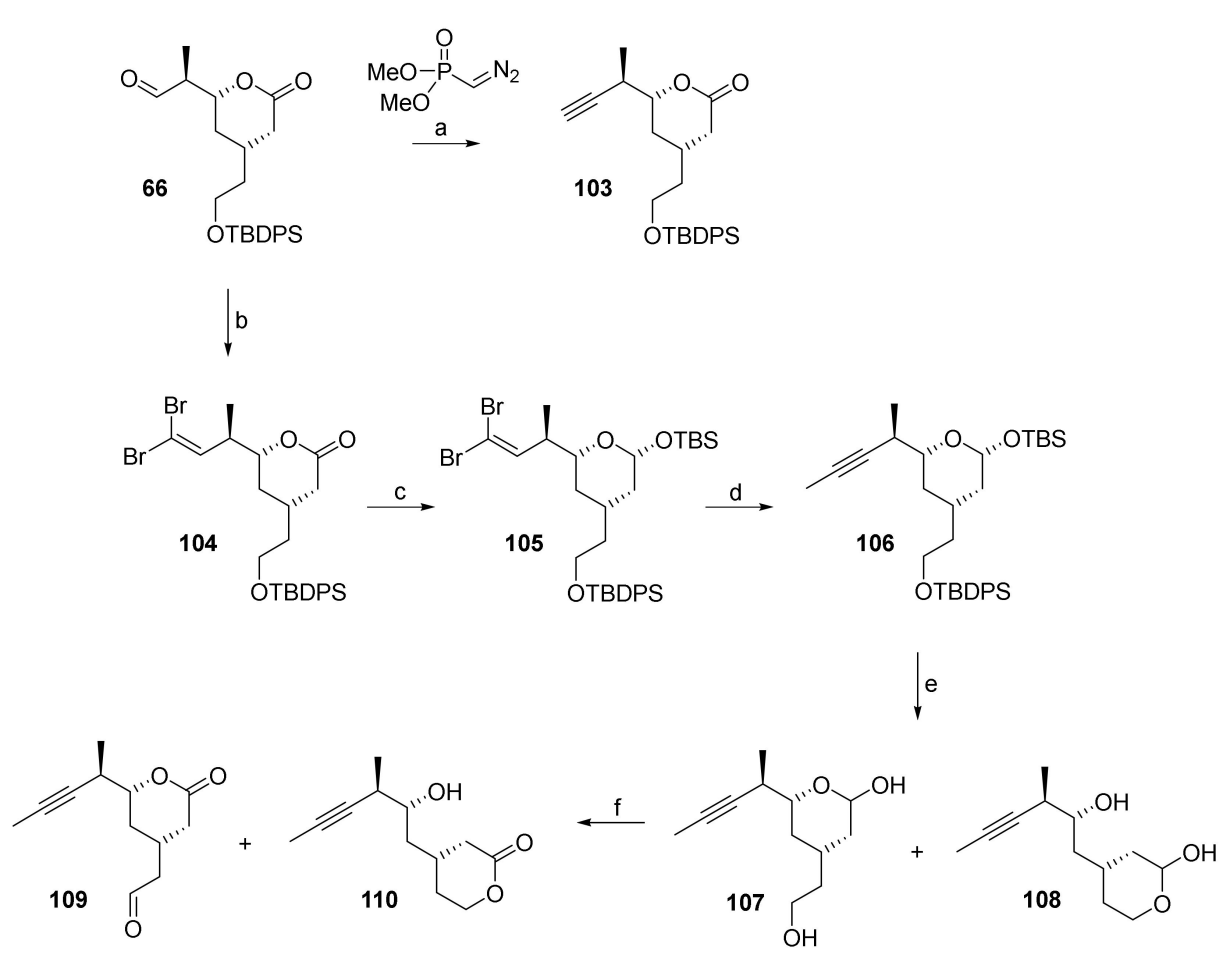
Reagents and conditions: (**a**) KO*t*Bu, *Seyferth-Gilbert* reagent (dimethyl (diazomethyl)phosphonate), THF, −78 °C, 43%; (**b**) CBr_4_, PPh_3_, CH_2_Cl_2_, −78 °C, 80% (2 steps, based on **65**); (**c**) (**i**) DIBAL, CH_2_Cl_2_, −78 °C, *dr* 1:1, (**ii**) TBSCl, imidazole, CH_2_Cl_2_, rt, 87% (2 steps) (single isomer); d) *n*BuLi, MeI, THF, −78 °C to rt, 90%; (**e**) TBAF, AcOH, THF, 0 °C to rt, quant. (107/108, 2:1); (**f**) TEMPO, BAIB, Yb(OTf)_3_ (cat.), CH_2_Cl_2_, 0 °C to rt, **109**: 62%, 110: 13%. BAIB, bis(acetoxy)iodobenzene; DIBAL, diisobutylaluminum hydride; TBAF, tetra-*n*-butylammonium fluoride; TBS, *tert*-butyldimethylsilyl; TEMPO, 2,2,6,6-tetramethylpiperidine-1-oxyl.

The synthesis of aldehyde **109** started from crude aldehyde **66** (obtained by Dess-Martin oxidation from primary alcohol **65**, cf. Scheme 11). In an initial experiment on small scale, **66** could be directly converted into terminal alkyne **103** by Seyferth-Gilbert alkynylation [86] (Scheme 20). However, **103** was obtained only in moderate yield (43%) and no efforts were spent on optimizing this transformation. Instead, we decided to assess the feasibility of the alkynylation of **66** via a Corey-Fuchs reaction as an alternative approach. In the event, dibromo olefin **104** could be prepared from **66** in good yield (80%) but, disappointingly, the conversion of the dibromo alkene moiety into a triple bond failed completely and the treatment of **104** with *n*BuLi led to complete decomposition of starting material. However, the installation of the triple bond via the dibromo alkene moiety became possible after reduction of the lactone group to the corresponding hemiacetal with DIBAL and protection of the latter with a TBS group, to furnish mixed acetal **105** as a single isomer in excellent yield (87%) (Scheme 20). Treatment of **105** with *n*BuLi and trapping of the intermediate acetylide with MeI gave alkyne **106** in 63% yield over the four step sequence from aldehyde **66**.

Simultaneous cleavage of both silyl ethers with TBAF/AcOH produced an inseparable mixture of lactols **107** and **108** (each as a pair of diastereoisomers; 2:1 ratio in favor of the desired regioisomer **107**), which was treated with bisacetoxy iodobenzene (BAIB) (2.2 equiv) in the presence of catalytic amounts of TEMPO (19 mol%) and Yb(OTf)_3_ (4 mol%) (this system is similar to the iodosylbenzene/TEMPO/Yb(OTf)_3_ system reported by *Vatèle* [87]) to produce the desired building block **109** as a single isomer in 62% yield (based on **106**) along with the corresponding hydroxy lactone **110** (13%).

**Scheme 21:**
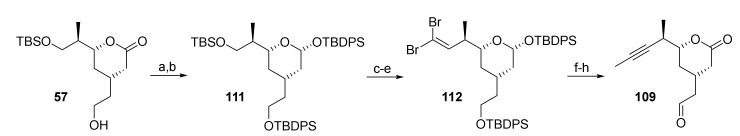
Reagents and conditions: (**a**) DIBAL, CH_2_Cl_2_, −78 °C, *dr* 1:1; (**b**) TBDPSCl, imidazole, CH_2_Cl_2_, rt, 90% (2 steps) (single isomer); (**c**) NaIO_4_, THF/water (4:1), rt, 87%; (**d**) DMP, CH_2_Cl_2_, 0 °C to rt, 88% *or* (COCl)_2_, DMSO, CH_2_Cl_2_, −78 °C, then NEt_3_, −78 °C to rt, 96%; (**e**) CBr_4_, PPh_3_, CH_2_Cl_2_, −78 °C, 99%; (**f**) *n*BuLi, THF, −78 °C to rt, then MeI, rt, 94%; (**g**) TBAF, AcOH, THF, 0 °C to rt; (**h**) TPAP (cat.), NMO, CH_2_Cl_2_, rt, 50% (2 steps). DIBAL, diisobutylaluminum hydride; DMP, Dess–Martin periodinane (1,1,1-tris(acetyloxy)-1,1-dihydro-1,2-benziodoxol-3-(1H)-one); NMO, 4-methylmorpholine *N*-oxide; TBAF, tetra-*n*-butylammonium fluoride; TPAP, tetrapropyl-ammonium perruthenate.

The above synthesis of aldehyde **109** from alcohol **57** involved an avoidable protection/deprotection sequence (cf. Scheme 11); a slightly shorter route to **109** was thus implemented in the scale-up process that relied on lactone reduction at the stage of alcohol **57** (Scheme 21).

The resulting 1:1 mixture of hemiacetals was then converted into tris-silyl derivative **111**, which was obtained as a single isomer in 90% yield from **57**. This finding is in line with Leahy’s observation that the all-equatorial isomer of the lactol is formed preferentially after equilibration under basic conditions [88]. Selective cleavage of the TBS-ether with NaIO_4_ [61] followed by either Dess-Martin or Swern oxidation gave an aldehyde (in 88% or 96% yield, respectively) that could be converted into dibromo olefin **112** in 99% yield. The latter was elaborated into aldehyde **109** by treatment with *n*BuLi/MeI, followed by TBDPS removal and TPAP oxidation. In contrast to the previously used TEMPO-mediated oxidation of **107**/**108**, TPAP/NMO gave aldehyde **109** as a single compound in 50% yield over 2 steps reproducibly. The former method proved to be rather unreliable for scale-up, with yields decreasing down to 20%. Aldehyde **109** could also be obtained from **107**/**108** by Dess-Martin oxidation, but the material was very difficult to purify by column chromatography.

#### 2.5.2. Building Block Assembly and Completion of the Synthesis of Rhizoxin F (**2**)

**Scheme 22:**
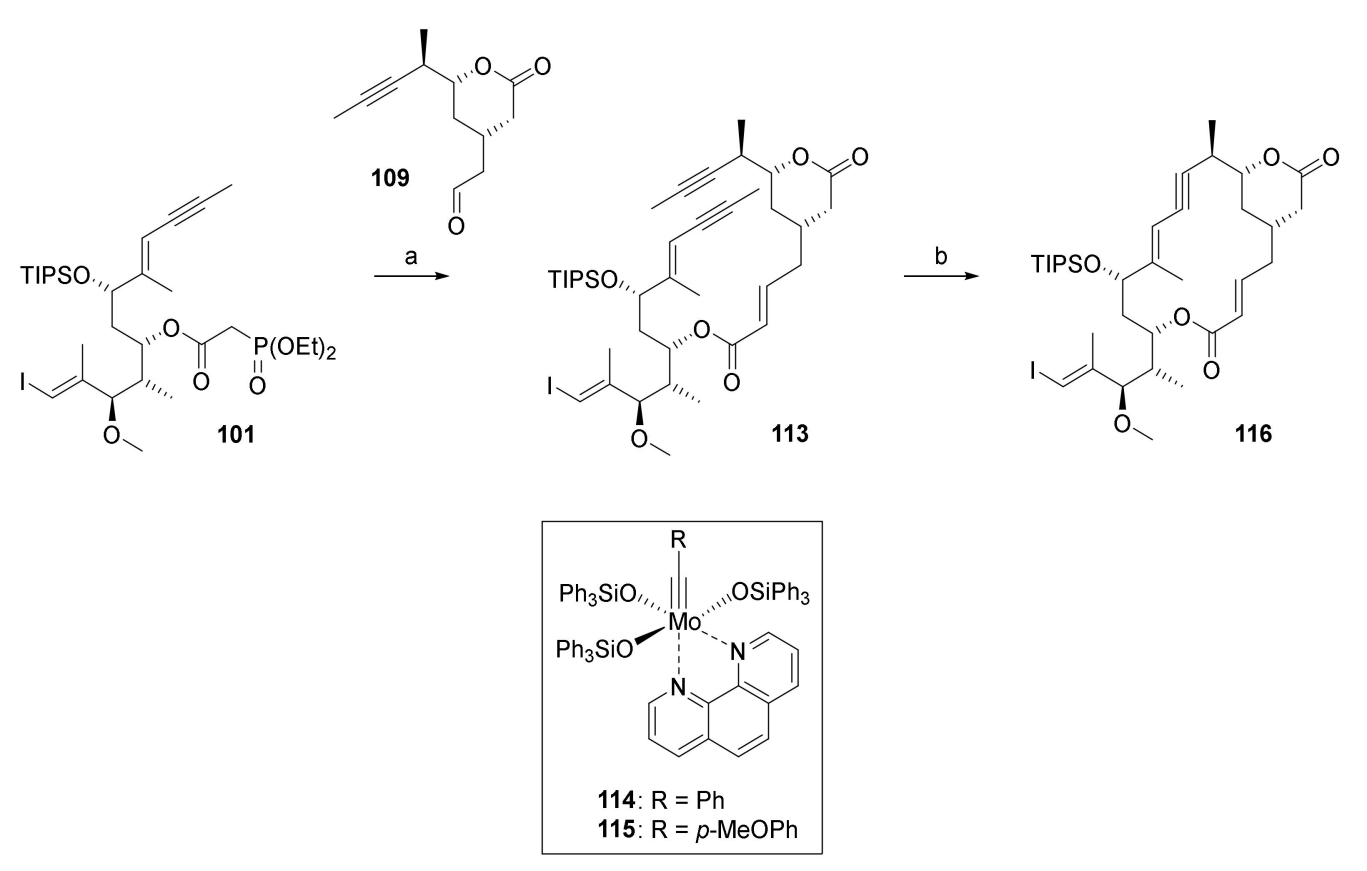
Reagents and conditions: (**a**) DBU, LiCl, CH_3_CN, 0 °C to rt, 81%; (**b**) Mo-complex **114** (35 mol%) or **115** (15 mol%), MnCl_2_, toluene, 5 Å molecular sieves, 125 °C; **114**: 69%, **115**: 56%/31%%. DBU, 1,8-diazabicyclo[5.4.0]undec-7-ene.

The HWE reaction between aldehyde **109** and phosphono ester **101** proceeded smoothly to deliver diyne **113** in 81% yield (Scheme 22).

Gratifyingly, and very much to our relief, heating **113** with the active catalysts obtained by in situ decomplexation of Mo-complexes **114** or **115** (complexes **114** and **115** were generous gifts from Prof. Alois Fürstner, Max Planck Institute for Carbon Research, Mühlheim/Ruhr, Germany) with MnCl_2_ to 125 °C in toluene for 2.45 h (for **114**) or 27 h (for **115**) gave the desired macrocycle **116** in 69% and 56%/31% yield (The two yields were obtained in two independent experiments on a 700 mg scale of **113** that were performed under ostensibly identical conditions. The reasons for the significant discrepancy in yields are unknown at this point.), respectively (Scheme 22). Heating of the reaction to at least 120 °C was necessary to achieve a practical rate of cyclization; at the same time, this finding also highlights the exceptional thermal stability of active catalysts derived from complexes **114** and **115** in the reaction mixture. While the yields obtained in the macrocyclization reaction as such may not be considered spectacular, given our prior extensive unsuccessful efforts at RCM, they were an impressive demonstration of the power of RCAM is an efficient method for macrocyclization.

With the hurdle of the ring-closure finally cleared, we now had to address the *E*-selective alkyne reduction of the newly formed triple bond. While the possibility of generating *Z* or *E* olefins from alkynes selectively, for example, by hydrosilylation/desilylation, is usually regarded as one of the benefits of RCAM, this transformation turned out to be a major challenge for **116**. In spite of extensive efforts, both the yield and selectivity of hydrosilylation and protodesilylation steps were unsatisfactory and no conditions could be identified to affect clean conversion of the ene-yne portion in **116** into the desired *E,E* diene moiety.

**Scheme 23:**
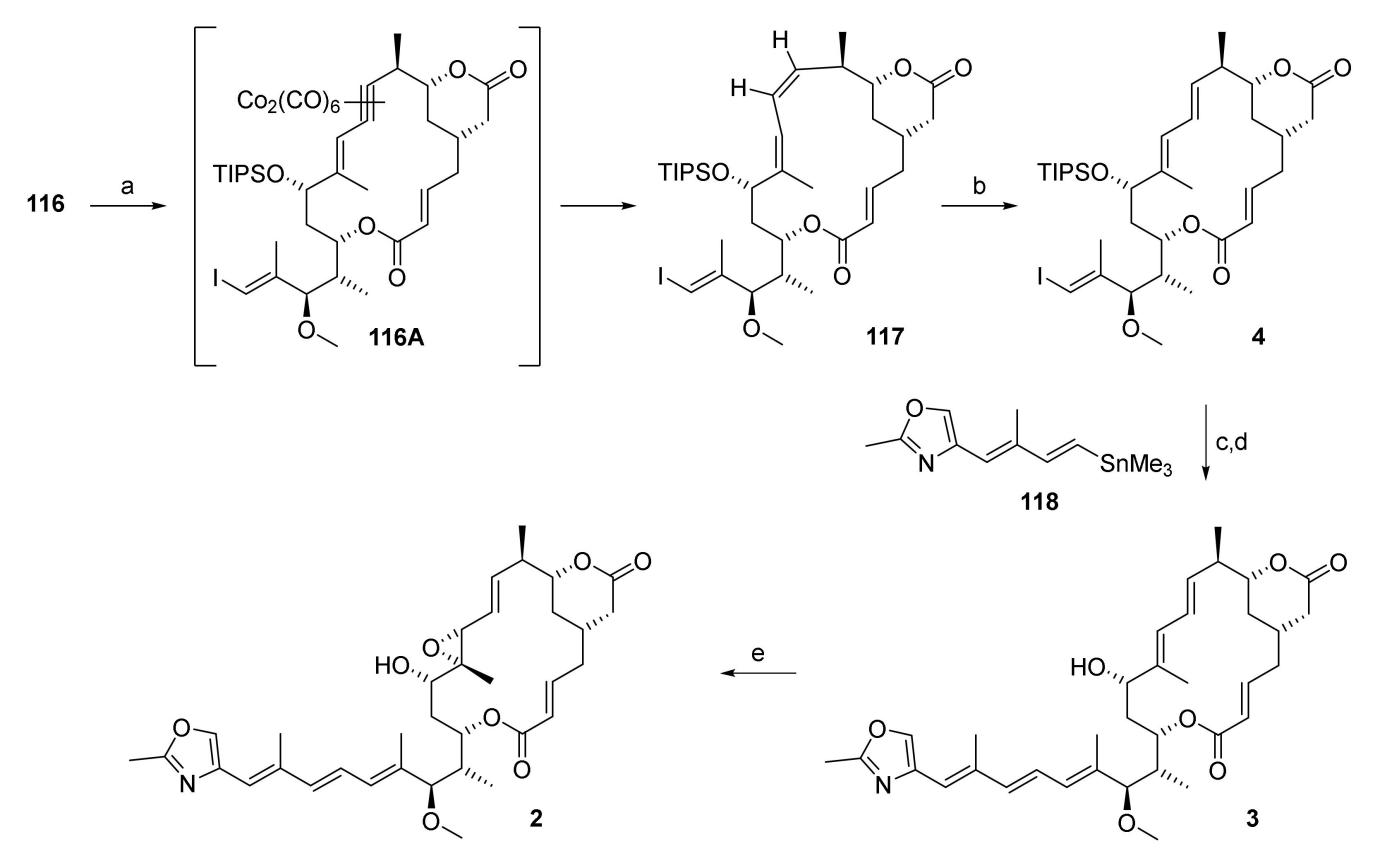
Reagents and conditions: (**a**) (**i**) Co_2_(CO)_8_, CH_2_Cl_2_, rt; (**ii**) *N*-ethylpiperidinium hypophosphite, benzene, reflux, 74% over 3 cycles, *Z* only; (**b**) AIBN, PhSH, benzene, reflux, 88%, *E*/*Z* = 20:1; (**c**) **3**, PdCl_2_(MeCN)_2_, DMF, rt, 68%; (**d**) HF·py, py, THF, 0 °C to rt, 54%; (**e**) *t*BuOOH, VO(acac)_2_, benzene, 0 °C to rt, 65%, 29% after preparative HPLC. acac, acetylacetonato; AIBN, azobisisobutyronitrile; py, pyridine.

However, in the context of the above experiments, we had obtained a small amount of the C(9)/C(10) *Z* isomer of **5**, which we found could be cleanly isomerized to **5** by refluxing with thiophenol and AIBN in benzene [89]. This observation suggested that an alternative approach towards the conversion of **116** into **4** could be the *Z*-selective reduction of the triple bond followed by double bond isomerization. As a consequence, experiments were conducted aiming to access C(9)/C(10)-*Z*-5 (i.e., **117**, Scheme 23) selectively; this included the examination of Red-Al-, Birch-, SmI_2_-, and hydrazine-promoted alkyne reductions, and a plethora of hydrogenations (transfer methods or application of elemental H_2_) and hydrometallations (with boron, tin, silicon), which are not to be discussed here in detail. Suffice it to say, that none of these methods delivered the desired diene **117**.

While alkyne **116** could not be reduced to the corresponding *Z* olefin directly, it could be converted cleanly into its biscobalthexacarbonyl complex **116A** (Scheme 23) by simple treatment with Co_2_(CO)_8_ [90,91]. We considered this a promising finding, as Isobe and co-workers had reported the reductive decomplexation of endocyclic biscobalthexacarbonyl complexes with rhodium on charcoal [92,93] or Wilkinson catalyst [94] under elevated hydrogen pressure to give olefins. They later discovered that the reductive decomplexation could also be brought about not only under radical conditions using *n*Bu_3_SnH [94], but also with the less toxic sodium hypophosphite monohydrate [95].

In our case, reductive decomplexation with *n*Bu_3_SnH was not compatible with the vinyl iodide moiety and the use of hydrogen at elevated pressure with Wilkinson’s catalyst or rhodium on charcoal was considered somewhat impracticable. Thus, the decomplexation of **116A** with sodium hypophosphite was deemed to be the most viable approach towards diene **117**. Unfortunately, Isobe’s original protocol led to transesterification of the δ-lactone moiety with the solvent 2-ethoxy ethanol (cellosolve). At the same time, partial reduction of the complex was observed, thus suggesting that **117** might indeed be accessible from **116A** if the decomplexation was carried out in a different solvent. When the reaction was performed in DMSO or CH_3_CN/H_2_O (6:1), no reduction took place. In order to allow for screening also in more lipophilic solvents, the tetrabutylammonium salt of hypophosphorous acid was then prepared, which proved to be soluble in CH_2_Cl_2_ and THF. Treatment of **116A** with this salt in CH_2_Cl_2_ or THF afforded a mixture of diene **117** and ene-yne **116** (ca. 50% conversion).

The ultimate choice of reducing agent was *N-*ethylpiperidinium hypophosphite, which was commercially available and which had been employed as a substitute for *n*Bu_3_SnH in the radical deoxygenation of alcohols [96]. *N-*Ethylpiperidinium hypophosphite proved to be more soluble than tetrabutylammonium hypophosphite; the most soluble reductant investigated was diphenylphosphine oxide (DPPO) [97], but this only led to decomposition of **116A**. In analogy to Isobe’s protocol with *n*Bu_3_SnH [94], benzene was the solvent of choice for the reduction of **116A** to **117** with *N-*ethylpiperidinium hypophosphite. After some fine-tuning of conditions, the reaction reliably afforded C(9)/C(10) *Z* olefin **117** in a highly selective fashion. In order to achieve full consumption of alkyne **116**, the two-step sequence of complex formation and reductive decomplexation had to be performed three times, as **116** was inseparable from olefin **117** by silica gel chromatography. This procedure initially provided **117** in 47% yield; while this yield was moderate, it was still considered very satisfactory, given the fact that an array of well-established methods had failed in reducing ene-yne **116**. Gratifyingly, it was discovered in subsequent experiments (by serendipity) that the yield for the conversion of **116** into **117** could be increased substantially, up to 74%, if an aqueous workup after the hypophosphite reduction was omitted.

Radical isomerization of **117** with AIBN and thiophenol then proceeded smoothly furnished the desired *E,E*-diene **4** in 88% yield (Scheme 23). Subsequent Stille coupling of **4** with the known vinyl stannane **118** [29], followed by HF-promoted silyl ether cleavage, gave rhizoxin D (**3**) in 37% overall yield. For reasons unknown, this yield is lower than those reported in the literature for the same two-step sequence (50% [29] and 76% [98]); in our hands, all intermediates with the C(15) side chain attached were prone to decomposition, in agreement with reports by Hertweck [99] and Stella [100]. Finally, directed epoxidation of rhizoxin D (**3**) according to Ohno and co-workers [19] proceeded in 65% yield, thus completing the first synthesis of rhizoxin F (**2**).

## 3. Materials and Methods

Detailed protocols for the synthesis of new compounds and the associated analytical data can be found in the Supplementary Material.

## 4. Conclusions

The total synthesis of the bacterial macrolide rhizoxin F (**2**) (also known as WF1360F) was accomplished based on macrocyclization by ring-closing alkyne metathesis (RCAM) at the C(9)–C(10) site, followed by a highly selective radical-based reduction/isomerization sequence to install the macrocyclic (*E,E*)-diene unit. The synthesis comprised 24 steps for the longest linear sequence from 1,4-butanediol (**44**) and delivered the target structure in ~1.5% yield overall yield. Notably, in spite of extensive efforts, all attempts to access macrocycle **4** by ring-closing olefin metathesis (RCM) met with complete failure. Our findings not only further highlight the general potential of RCAM in natural product synthesis, which by now is well established through the work of Fürstner and others; but it also shows that RCAM can serve as a valuable alternative when macrocyclization cannot be effected by RCM. We have also developed a new alkyne semireduction protocol, which reliably furnished diene **4** in good yields and with exquisite selectivity. This protocol provides an additional option for the elaboration of macrocyclic alkynes obtained by RCAM into the corresponding olefins. Finally, although this is not discussed here, macrocyclic vinyl iodide **4** has been smoothly converted into a number of side chain-modified analogs of **2**; in addition, our overall strategy for the synthesis of **2** was also adaptable to the synthesis of a (side chain-modified) lactam analog.

## Supplementary Materials

The following are available online. synthesis protocols and analytical data for all new compounds, Table S1: RCM screening for substrate **5**, Table S2: RCM screening for substrate **70**.
