# Supplementary material for: Ring-Closing Metathesis Approaches towards the Total Synthesis of Rhizoxins
Source: Molecules. 2020 Oct 2;25(19):4527. doi: 10.3390/molecules25194527 (PMC7582377; doi:10.3390/molecules25194527)
Supplement: Supplementary file 1 [file molecules-25-04527-s001.pdf]

Supporting Materials

Ring-closing Metathesis Approaches Towards the Total Synthesis of Rhizoxins

Marc Liniger, Christian M. Neuhaus, Karl-Heinz Altmann

ETH Zürich, Department of Chemistry and Applied Biosciences, Institute of Pharmaceutical Sciences, Vladimir-Prelog-Weg 4, HCI H405, 8093 Zürich, Switzerland

Synthesis protocols and analytical data.

^{1}H- and ^{13}C-NMR spectra of new compounds.

General Methods

All non-aqueous manipulations were conducted under nitrogen or argon atmosphere in flame-dried glassware, using standard *Schlenk*-, syringe/septa- and/or glovebox techniques (MBraun Labmaster 130).
CH_2Cl_2 was distilled from CaH; THF, Et_2O, benzene, toluene were distilled from Na/benzophenone. All other absolute solvents were purchased from Fluka (absolute over molecular sieves). Commercial chemicals were used without further purification, unless otherwise noted.
Solvents for extractions, column chromatography and thin layer chromatography (TLC) were purchased as commercial grade and distilled prior to use. TLC was performed on Merck TLC aluminum sheets (silica gel 60, F254). Spots were visualized with UV light (λ = 254 nm) or through staining with $KMnO_4/K_2CO_3$ or vanillin/H_2SO_4. Chromatographic purification of products was performed by flash chromatography (FC) using Fluka silica gel 60 for preparative column chromatography (particle size 40–63 µm).
NMR spectra were recorded on a Bruker AV-400 400 MHz or a Bruker AV-500 500 MHz NMR spectrometer at 298 K. NMR spectra are referenced to the residual solvent peak (^1H 7.26 ppm, ^{13}C 77.0 ppm for $CDCl_3$; ^1H 7.36 ppm, ^{13}C 128.37 ppm for C_6D_6). All ^{13}C spectra were measured with complete proton decoupling. Spin multiplicities are reported as follows: s = singlet, d = doublet, t = triplet, q = quartet, quint = quintet, sext = sextet, m = multiplet, m_c = centered multiplet, br = broad signal; *J* = coupling constant in Hz.
Infrared spectra (IR) were recorded on a Jasco FT/IR-6200 instrument. Resonance frequencies are given as wavenumbers in cm^{-1}.
Optical rotations were measured on a Jasco P-1020 polarimeter and are reported as follows $[\alpha]_D^{24}$: concentration (g/100 mL) and solvent.
High resolution mass spectra (HRMS) were recorded by the ETH Zürich MS service on a Varian IonSpec Ultima (ESI) or a Waters Micromass Autospec Ultima spectrometer (EI).
For analytical and semipreparative HPLC the following combination of devices by VWR HITACHI was used: column oven L-2350, diode array detector L-2455, autosampler L-2200, pump L-2130. A Waters Symmetry C18, 3.5 µm, 4.6x100 mm column was used for analytical purposes (oven at 40 °C). For semipreparative HPLC a Waters Symmetry C18, 5 µm, 7.8×100 mm column was used (room temperature). For preparative HPLC a device by Gilson equipped with a Waters SymmetryPrep C18, 5 µm, 19x100 mm column was used (room temperature).

Diastereomeric ratios (*dr*) are based on intensity ratios of relevant signals in the ^{1}H-NMR spectrum of the diastereomeric mixture.

When products could not be (fully) separated from specific known impurities or contained significant amounts of solvent, yields were approximated from the w/w ratio of product *vs* impurity/solvent based on the intensity ratio(s) of signals in the ^{1}H-NMR spectrum.

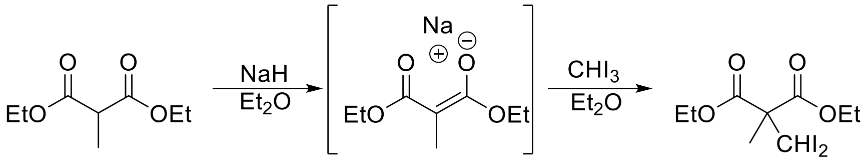

S1 S2

Diethyl 2-(diiodomethyl)-2-methylmalonate (S2): To a suspension of NaH (5.51 g 60% dispersion in mineral oil, 138 mmol, 1.20 eq) in Et_2O (70 mL) was added a solution of diethyl methylmalonate (**S1**) (19.8 mL, 115 mmol, 1.00 eq) in Et_2O (80 mL) over 1 h with mechanical stirring. Gas evolution could be observed and a grayish slurry was formed over time. After another 40 min at rt, the mixture was heated to reflux (bath. temp. 45 °C) for 3 h. Then it was cooled to rt in a water bath and CHI_3 (45.2 g, 115 mmol, 1.00 eq) was added over ca. 10 min, whereupon the suspension turned less viscuous. The yellow reaction mixture was heated to reflux for 24 h. The yellow colour faded slowly over time and became beige in the end. The suspension was cooled to 0 °C, 2 M HCl (100 mL) was added and the orange solution was stirred at rt for ca. 30 min. Then the layers were separated and the aqueous phase was extracted with Et_2O (3×30 mL). The combined organic layers were washed with brine, dried over $MgSO_4$ and concentrated *in vacuo*. Crude **S2** (50.5 g, dark brown liquid) was used without purification.

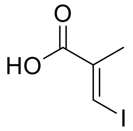

S3

(E)-3-Iodo-2-methylacrylic acid (S3): The crude diester **S2** (50.5 g, ca. 0.115 mol) was dissolved in EtOH/H_2O (320 mL, 3:1) and KOH (16.7 g, 0.298 mol, ca. 2.6 eq) was added. The mixture was heated to reflux (bath temp. = 97 °C) for 24 h. After cooling to rt, ethanol was removed under reduced pressure. The so obtained suspension was diluted with Na_2CO_3 (sat. aq.). The basic solution was extracted with Et_2O (3×100 mL). Then it was acidified (pH≤1) with conc. HCl and extracted with CH_2Cl_2. The combined extracts were washed with brine, dried over $MgSO_4$ and concentrated *in vacuo*. The brown crude was purified by column chromatography (CH_2Cl_2:MeOH 40:1→15:1) to afford the desired vinyl iodide **S3** (17.5 g, 72% over two steps) as an amber liquid.

^{1}H-NMR ($CDCl_3$, 400.1 MHz): δ = 8.02 (m_c, C*H*=C), 2.06 (d, *J* = 1.1 Hz, 3H, C*H*$_3$).

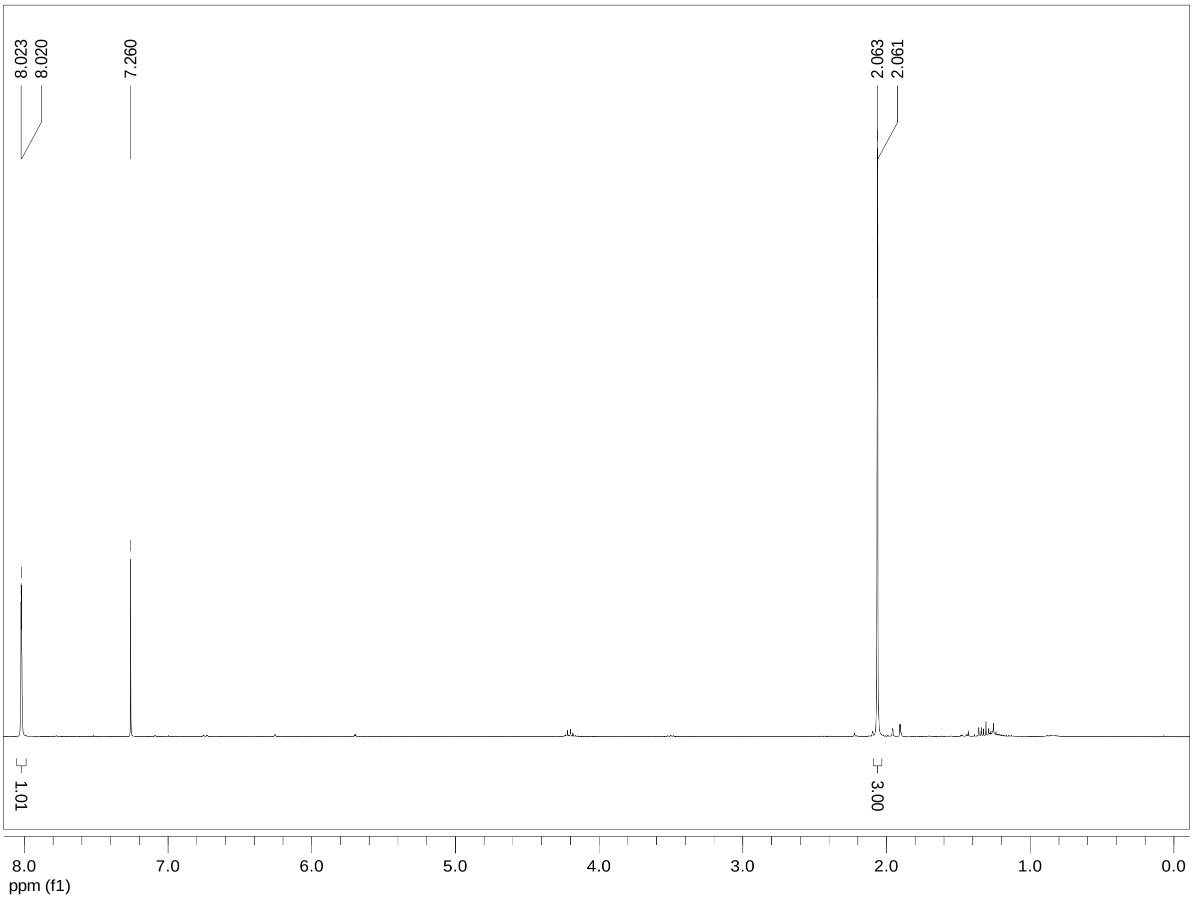
8.023
8.020
7.260
2.063
2.061
1.01
3.00
8.0
7.0
6.0
5.0
4.0
3.0
2.0
1.0
0.0
ppm (f1)

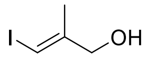

102

(*E*)-3-Iodo-2-methylprop-2-en-1-ol (102): To a solution of the acid **S3** (17.5 g, 82.6 mmol, 1.00 eq) in Et_2O (160 mL) was added $LiAlH_4$ (3.13 g 95% as LAH, 82.5 mmol, 1.00 eq) portionwise at 0 °C. Vigorous gas evolution was observed. The suspension was stirred at 0 °C for 1 h 15 min and was then allowed to warm to rt. After 1 h 35 min, the gray suspension was recooled to 0 °C, the reaction was quenched with water (some Na_2SO_4 (s) added before) and acidified with H_2SO_4 (2 M, 100 mL). The layers were separated and the aqueous phase was extracted with CH_2Cl_2 (4×40 mL). The combined organic extracts were washed with brine, dried over $MgSO_4$ and concentrated under reduced pressure. The crude was purified by column chromatography (pent:Et_2O 2:1→1:1) to afford the desired alcohol **102** (14.3 g, 87%) as a nearly colourless liquid.

TLC (SiO_2, pent:Et_2O 4:1): R_f = 0.27 (smears)
^{1}H-NMR ($CDCl_3$, 400.1 MHz): δ = 6.29 (m_c, 1H), 4.13 (bs, 2H), 1.85 (s, 3H).

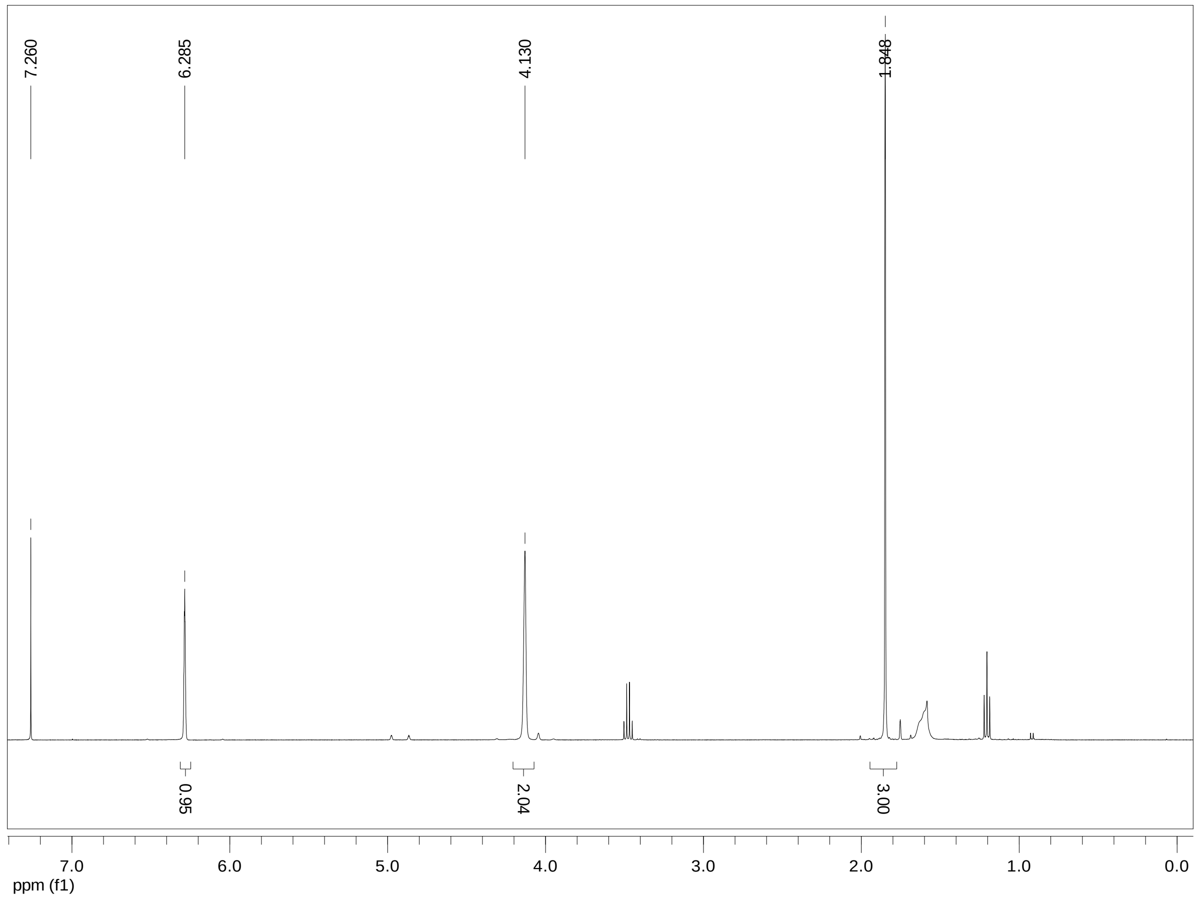

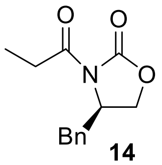

14

(*R*)-4-Benzyl-3-propionyloxazolidin-2-one (14): To a solution of the commercially available oxazolidinone **S4** (5.00 g, 28.2 mmol, 1.00 eq) in THF (120 mL) was added *n*-BuLi (19.4 mL 1.6 M in Hexanes, 31.0 mmol, 1.10 eq) dropwise at −78 °C. The resulting dark orange solution was aged at −78 °C for 35 min. Then propionyl chloride (4.93 mL, 56.4 mmol, 2.00 eq) was added at −78 °C. The dark colour discharged rapidly. The mixture was stirred for further 2 h 20 min at −78 °C. Then Na_2CO_3 (sat. aq., 30 mL) was added to quench the remaining propionyl chloride and the mixture was diluted with NaCl (sat. aq., 15 mL) and EtOAc (20 mL). The layers were separated and the aqueous phase was extracted with EtOAc (3×20 mL). The combined organic layers were washed with brine, dried over $MgSO_4$ and concentrated *in vacuo*. The crude was purified by column chromatography (CH_2Cl_2:acetone 15:1) to afford the title compound as a yellow oil, which crystallised in a freezer (−18 °C). The material was recrystallysed from hex:EtOAc (ca. 10:1, some hexane added). The yellow mother liquour was discarded and the crystals were washed with cold hexane to afford the title compound **14** (5.97 g, 91%) in colourless needles.

TLC (CH_2Cl_2:acetone 15:1): R_f = 0.70

^{1}H-NMR ($CDCl_3$, 400.1 MHz): δ = 7.38-7.27 (m, 3H), 7.24-7.16 (m, 2H), 4.68 (m_c, 1H), 4.25-4.12 (m, 2H), 3.31 (dd, *J* = 3.3 Hz, *J* = 13.4 Hz, 1H), 2.96 (dd, *J* = 7.3 Hz, *J* = 9.3 Hz, 1H), 2.96 (m_c, 1H), 2.77 (dd, *J* = 9.6 Hz, *J* = 13.4 Hz, 1H), 1.21 (t, *J* = 7.3 Hz, 3H).

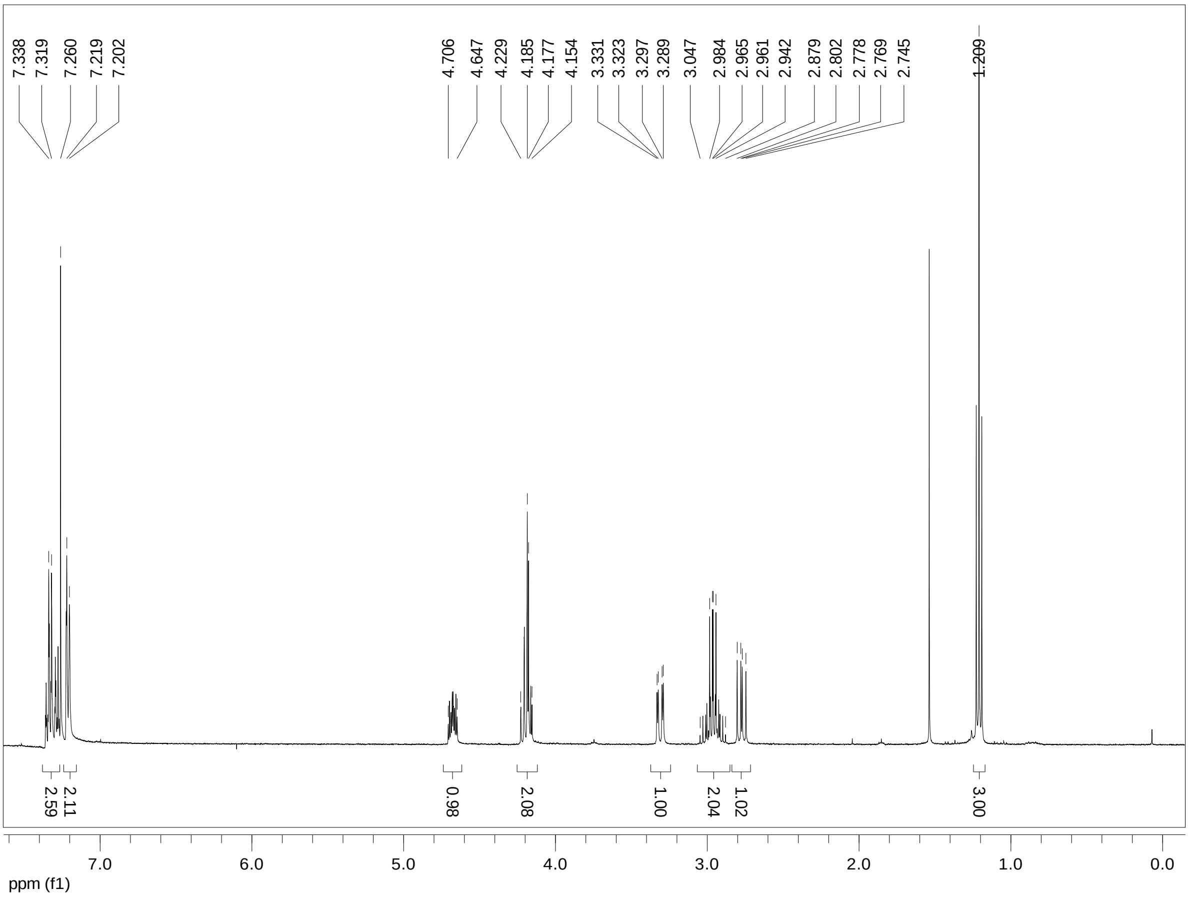
7.338
7.319
7.260
7.219
7.202
4.706
4.647
4.229
4.185
4.177
4.154
3.331
3.323
3.297
3.289
3.047
2.984
2.965
2.961
2.942
2.879
2.802
2.778
2.769
2.745
1.209
2.59
2.11
0.98
2.08
1.00
2.04
1.02
3.00
7.0
6.0
5.0
4.0
3.0
2.0
1.0
0.0
ppm (f1)

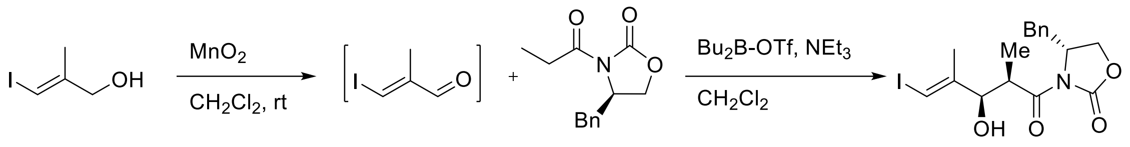

102 13 14 15

(*R*)-4-Benzyl-3-((2*R*,3*R*,*E*)-3-hydroxy-5-iodo-2,4-dimethylpent-4-enoyl)-oxazolidin-2-one (15):

Preparation of the aldehyde: To a solution of the allylic alcohol **102** (7.51 g, 37.9 mmol, 1.00 eq) in CH_2Cl_2 (140 mL) were added 3 Å molecular sieves beads and activated MnO_2 (66.0 g, 759 mmol, 20.0 eq) at rt. The suspension was stirred for ca. 2 h at the same temperature. Then it was diluted with CH_2Cl_2 and filtered through a pad of Celite, topped with a few mm of basic alox. The filter was washed thoroughly with CH_2Cl_2. The combined filtrates were concentrated under reduced pressure to a volume of ca. 55 mL and the crude aldehyde **13** was used directly in the following reaction.

Evans Aldol: To a solution of the *Evans* oxazolidinone **14** (5.90 g, 25.3 mmol, 1.00 eq) in CH_2Cl_2 (50 mL) was added Bu_2BOTf (29.0 mL 1.0 M in hexanes, 29.0 mmol, 1.15 eq) portionwise over ca. 30 min at 0 °C. The resulting brownish solution was aged for 5-10 min, then NEt_3 (4.57 mL, 32.9 mmol, 1.30 eq) was added dropwise over ca. 5 min. The brown colour faded and the mixture turned pale yellow (nearly colourless). The mixture was aged for 5-10 min and then cooled to −78 °C. After a few minutes stirring at −78 °C, a precooled (−78 °C) solution of the aldehyde **13** (ca. 37.9 mmol, 1.50 eq) in CH_2Cl_2 (ca. 55 mL) was added *via* cannula in several portions. The resulting pale yellow solution was stirred at the same temperature for 2 h and for 45 min at 0 °C, the yellow colour darkening, before it was quenched by the addition of pH 7 buffer. Afterwards the mixture was stored in a freezer over the weekend. Subsequently, MeOH (60 mL) and 30% H_2O_2:MeOH 1:3 (60 mL) were added at 0 °C and the mixture was stirred at the same temperature for 1 h. The organic solvents were removed at the rotavap. The dark brown residue was diluted with water (120 mL) and CH_2Cl_2 (50 mL) and the layers were separated. The aqueous phase was extracted with CH_2Cl_2 (3×50 mL). The combined organic extracts were washed with $Na_2S_2O_3$ (sat. aq., discoloured the brown layer on shaking), and brine, dried over $MgSO_4$ and concentrated at the rotavap. The crude was purified by column chromatography (CH_2Cl_2:Et_2O 20:1→10:1) to afford the desired product **15** as amber crystals (9.94 g, 92%).

10

^{1}H-NMR ($CDCl_3$, 400.1 MHz): δ = 7.38-7.27 (m, 3H), 7.23-7.17 (m, 2H), 6.43 (bs, 1H), 4.70 (m_c, 1H), 4.51 (bs, 1H), 4.31-4.16 (m, 2H), 4.00 (m_c, 1H), 3.26 (dd, *J* = 3.3 Hz, *J* = 13.4 Hz, 1H), 3.14 (d, *J* = 2.8 Hz, 1H), 2.80 (dd, *J* = 9.4 Hz, *J* = 13.4 Hz, 1H), 1.81 (s, 3H), 1.18 (d, *J* = 7.0 Hz, 3H).

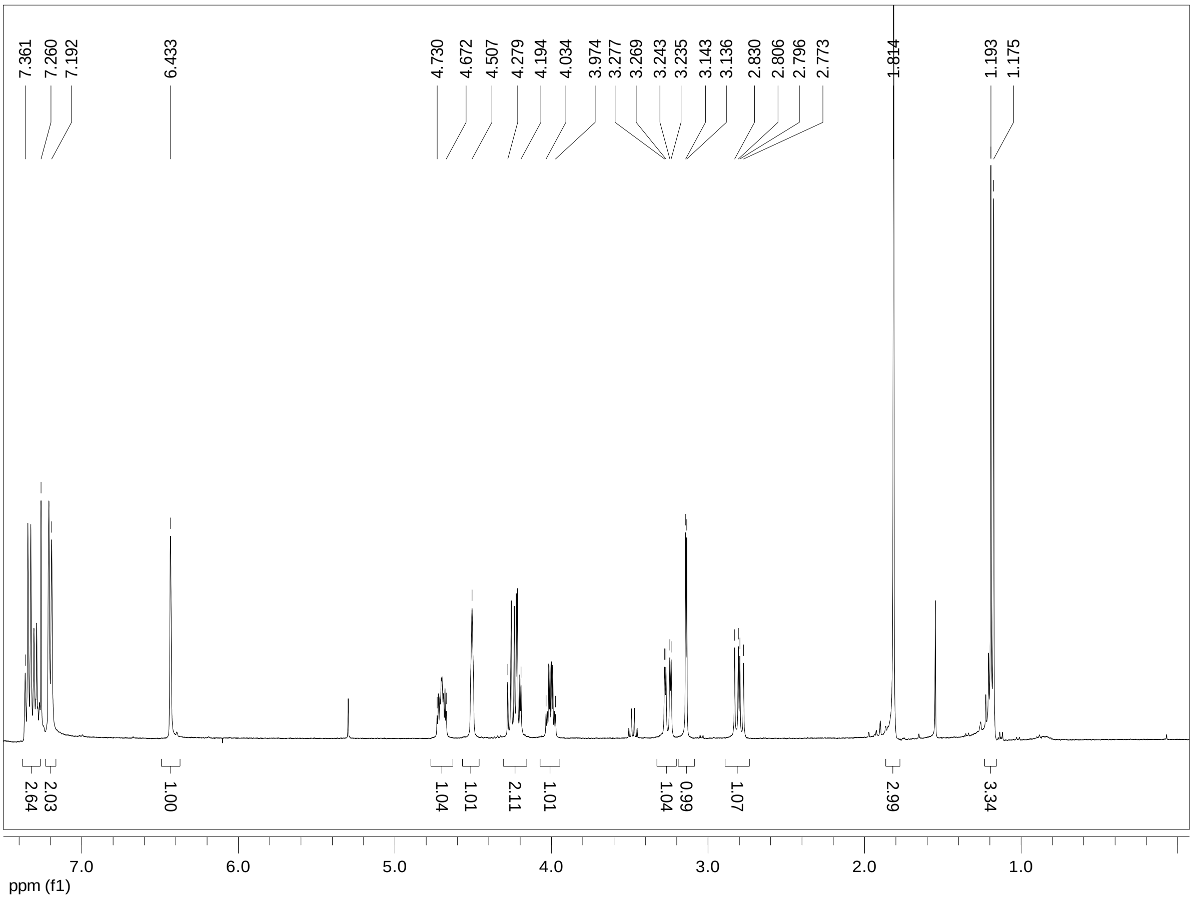

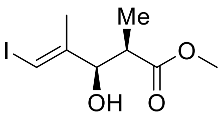

16

Methyl (2*R*,3*R*,*E*)-3-hydroxy-5-iodo-2,4-dimethylpent-4-enoate (16): To a solution of the *Evans* aldol adduct **15** (711 mg, 1.66 mmol, 1.00 eq) in CH_2Cl_2 (15 mL) was added NaOMe (4.56 mL 0.4 M in MeOH, 1.82 mmol, 1.10 eq) over 12 min at 0 °C. The mixture was aged for 13 min, then the reaction was quenched into ice cold NH_4Cl (sat. aq.). The layers were separated and the aqueous phase was extracted with CH_2Cl_2 (3×15 mL). The combined organic extracts were washed with brine, dried over $MgSO_4$ and concentrated *in vacuo*. The crude was purified by column chromatography (CH_2Cl_2:Et_2O 20:1) to afford the desired ester **16** (487 mg, 92% wt/wt along with CH_2Cl_2, 446 mg, 95%) as a pale yellow liquid.

TLC (Hex:EtOAc 10:1): R_f = 0.17
^{1}H-NMR ($CDCl_3$, 400.1 MHz): δ = 6.39 (m_c, 1H), 4.49 (m_c, 1H), 3.71 (s, 3H), 2.70 (qd, J = 7.2 Hz, J = 4.1 Hz, 1H), 2.72 (d, J = 3.3 Hz, 1H), 1.79 (m_c, 3H), 1.12 (d, J = 7.1 Hz, 3H);
^{13}C-NMR ($CDCl_3$, 100.6 MHz): δ = 175.8, 145.9, 79.3, 75.8, 52.1, 42.2, 21.1, 10.5.

12

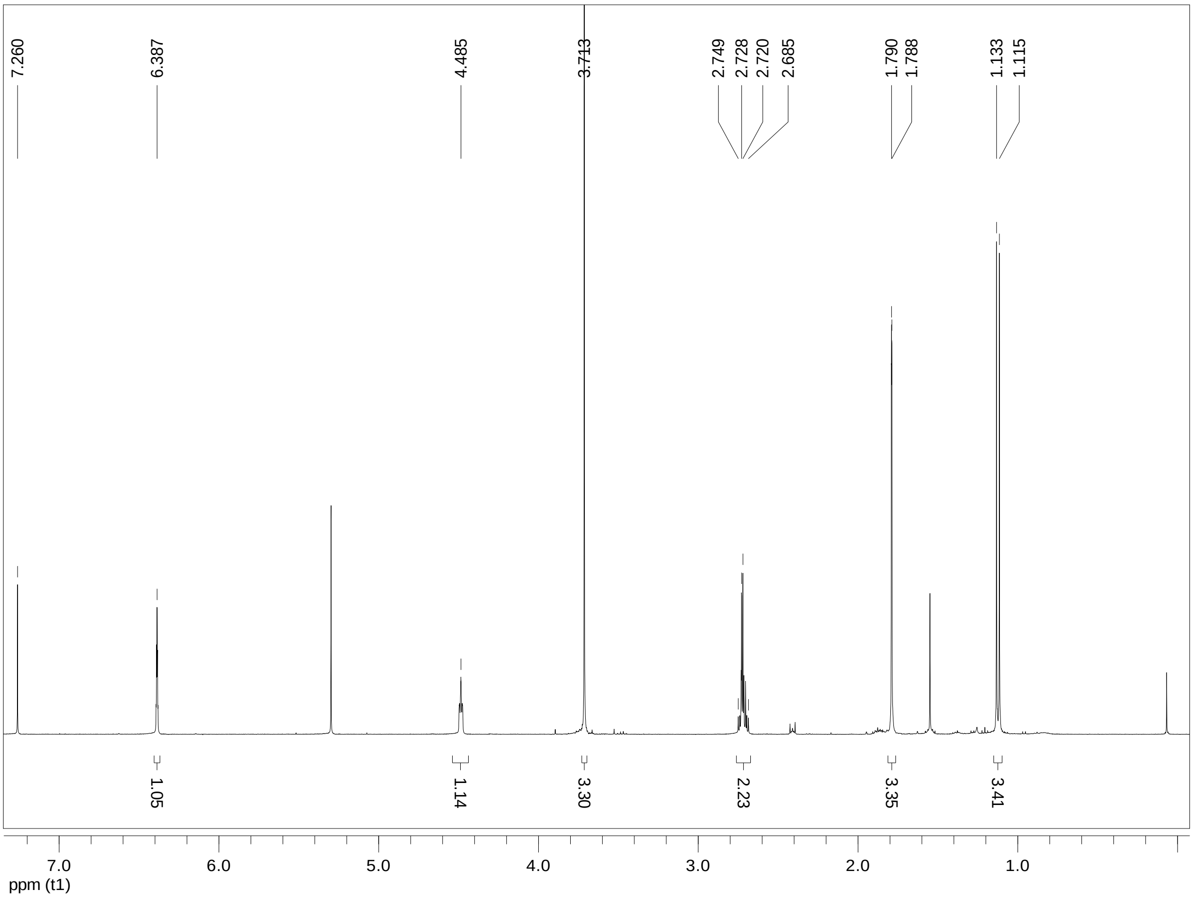
7.260
6.387
4.485
3.713
2.749
2.728
2.720
2.685
1.790
1.788
1.133
1.115
1.05
1.14
3.30
2.23
3.35
3.41
7.0
6.0
5.0
4.0
3.0
2.0
1.0
ppm (t1)

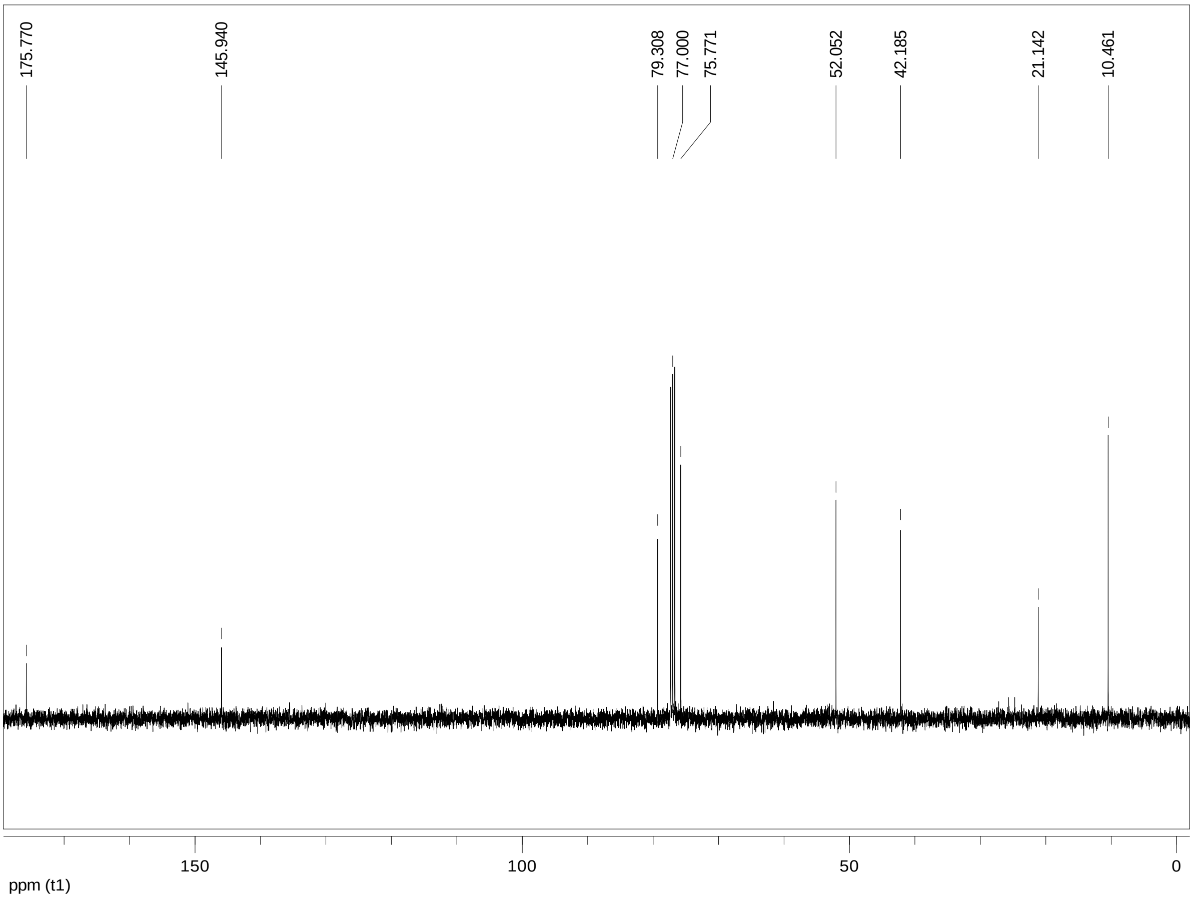
175.770
145.940
79.308
77.000
75.771
52.052
42.185
21.142
10.461
150
100
50
0
ppm (t1)

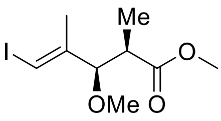

S5

Methyl (2*R*,3*R*,*E*)-5-iodo-3-methoxy-2,4-dimethylpent-4-enoate (S5): To a cooled (0 °C) solution of the alcohol **16** (55.0 mg, 0.194 mmol, 1.00 eq) and MeI (0.121 mL, 1.94 mmol, 10.0 eq) in THF/DMF (3:1, 2 mL) was added NaH (7.00 mg, 0.291 mmol, 1.50 eq). A pale yellow precipitate formed instantaneously. The slurry was stirred for 1 h 10 min at 0 °C. Then the reaction was quenched with pH 7 phosphate buffer (3 *Pasteur* pipettes). CH_2Cl_2 was added and the layers were separated. The aqueous phase was extracted with CH_2Cl_2 (3×10 mL). The combined organic extracts were washed with brine, dried over $MgSO_4$ and concentrated under reduced pressure. The crude was purified by column chromatography (hex:Et_2O 15:1) to afford the desired product **S5** (54.0 mg, 94%) as a colourless liquid.

TLC (hex:EtOAc 10:1): R_f = 0.56
^{1}H-NMR ($CDCl_3$, 400.1 MHz): δ = 6.22 (m_c, 1H), 3.79 (d, *J* = 8.0 Hz, 1H), 3.64 (s, 3H), 3.22 (s, 3H), 2.68 (qd, *J* = 6.9 Hz, *J* = 8.0 Hz, 1H), 1.76 (d, *J* = 1.1 Hz, 3H), 1.20 (d, *J* = 6.9 Hz, 3H).

14

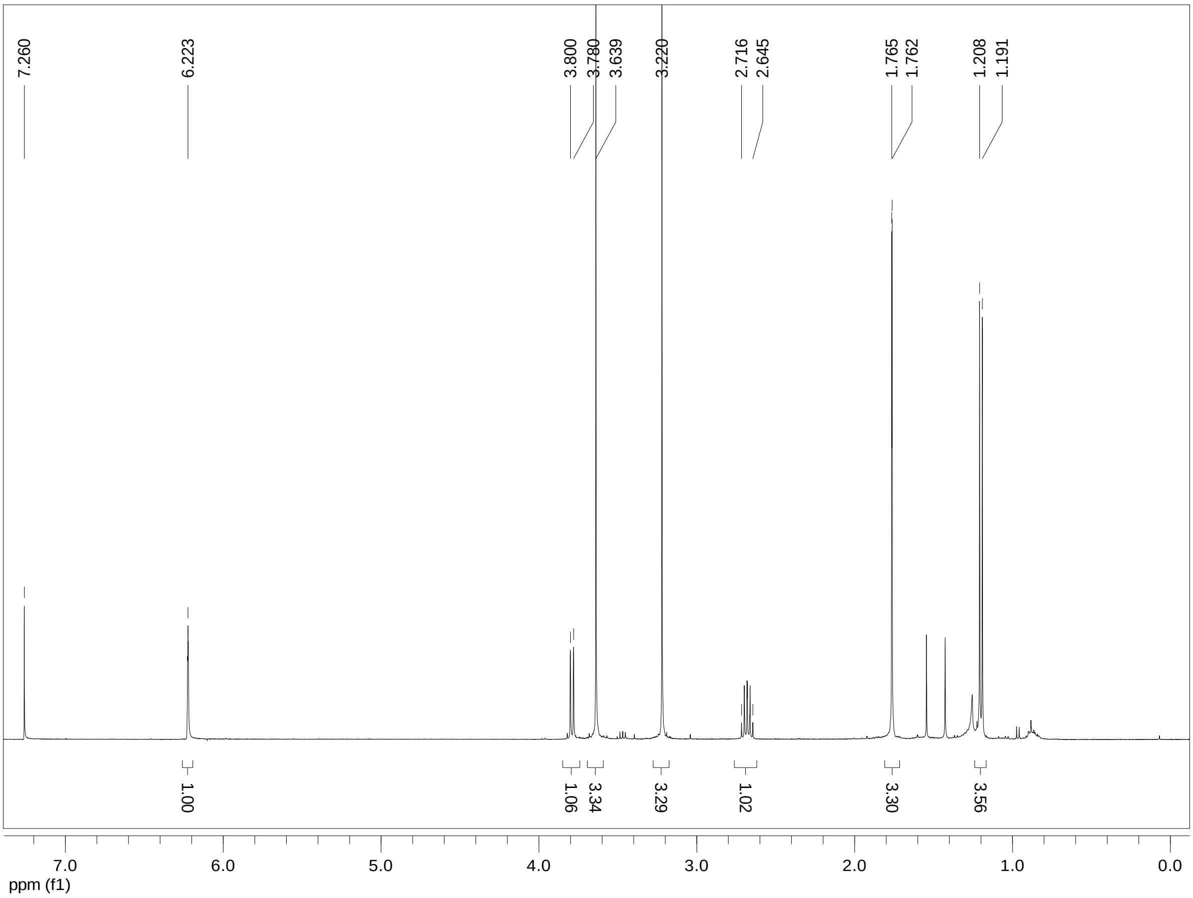

7.260
6.223
3.800
3.780
3.639
3.220
2.716
2.645
1.765
1.762
1.208
1.191
1.00
1.06
3.34
3.29
1.02
3.30
3.56
7.0
6.0
5.0
4.0
3.0
2.0
1.0
0.0
ppm (f1)

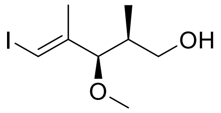

S6

(2*S*,3*R*,*E*)-5-Iodo-3-methoxy-2,4-dimethylpent-4-en-1-ol (S6): To a solution of the ester **S5** (50 mg, 0.168 mmol, 1.00 eq) in CH_2Cl_2 (1.8 mL) was added DIBAL (0.352 mL 1 M in CH_2Cl_2, 0.352 mmol, 2.10 eq) dropwise at −78 °C. The mixture was stirred for 20 min at the same temperature and was then allowed to warm to 0 °C. After 1 h the reaction was quenched by successive addition of MeOH (0.1 mL), H_2O (0.30 mL), 10% aq NaOH (0.30 mL) and H_2O (0.90 mL). The aqueous phase was diluted with water, the layers were separated and the aqueous phase was extracted with CH_2Cl_2 (3×2 mL). The combined organic extracts were washed with brine, dried over $MgSO_4$ and concentrated under reduced pressure. The crude was filtered through a pad of silica (Et_2O as eluent) and concentrated again to afford the desired alcohol **S6** (44.1 mg, 97%) as a colourless oil which was used for the next step without purification.

TLC (Hex:EtOAc 5:1): R_f = 0.20
^{1}H-NMR ($CDCl_3$, 400.1 MHz): δ = 6.20 (m_c, 1H), 3.67 (d, *J* = 6.2 Hz, 1H), 3.53 (m_c, 2H), 3.24 (s, 3H), 1.87 (m_c, 1H), 1.77 (d, *J* = 1.0 Hz, 3H), 1.71 (t, *J* = 5.2 Hz, 1H), 1.20 (d, *J* = 6.9 Hz, 3H).

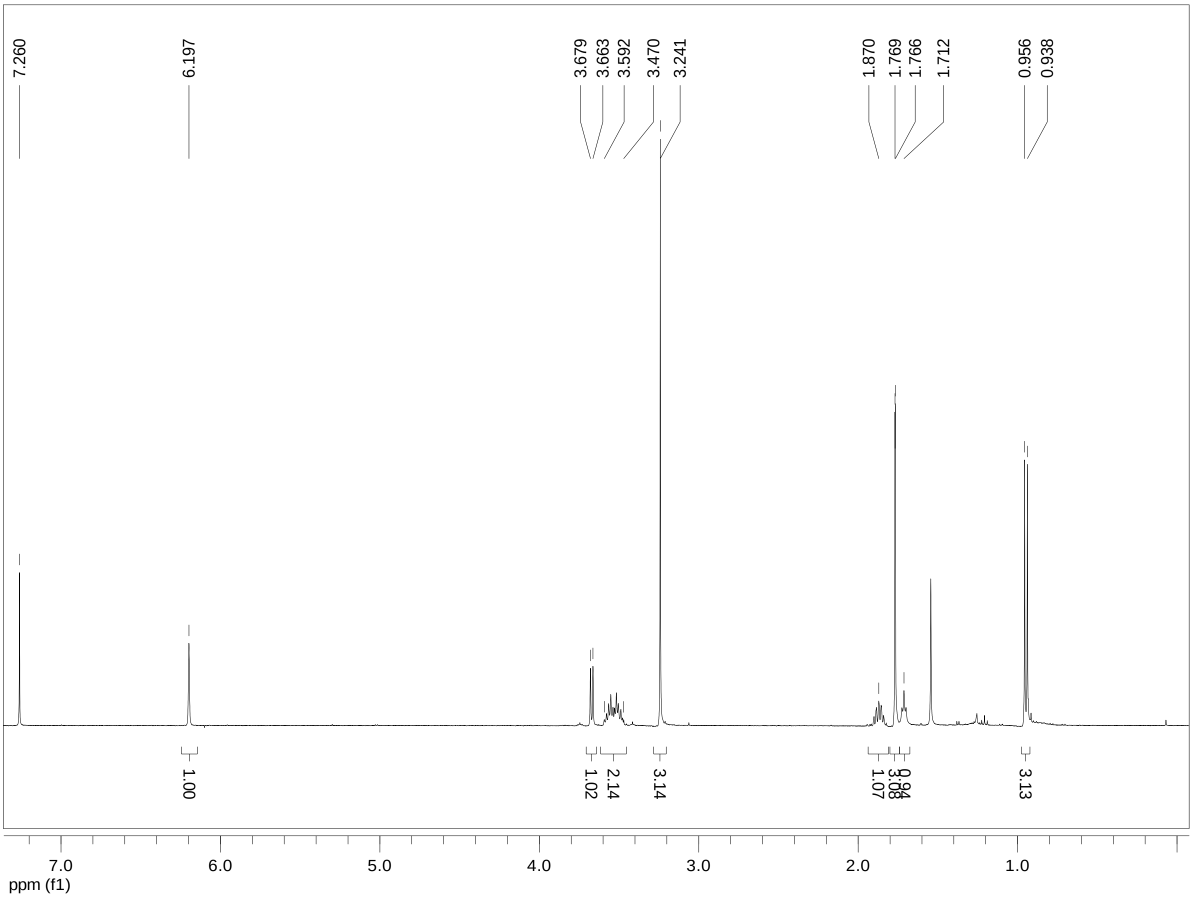
7.260
6.197
3.679
3.663
3.592
3.470
3.241
1.870
1.769
1.766
1.712
0.956
0.938
1.00
1.02
2.14
3.14
1.07
3.08
0.94
3.13
7.0
6.0
5.0
4.0
3.0
2.0
1.0
ppm (f1)

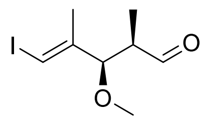

10

(2*R*,3*R*,*E*)-5-Iodo-3-methoxy-2,4-dimethylpent-4-enal (10): To a cooled (0 °C) solution of the alcohol **S6** (41.7 mg, 0.154 mmol, 1.00 eq) in CH_2Cl_2 (1.5 mL) were added $NaHCO_3$ (16.9 mg, 0.201 mmol, 1.30 eq) and *Dess-Martin* periodinane (42.6 mg, 0.100 mmol, 0.65 eq). The suspension was allowed to warm to rt. After 25 min, the mixture was re-cooled to 0 °C again and more DMP (42.6 mg, 0.100 mmol, 0.65 eq) was added. The cooling was removed again and the reaction was stirred at rt for a further 45 min. Since there was still little starting material left, more DMP (0.2 eq) and $NaHCO_3$ (small spatula) were added. After another 20 min stirring at rt, the reaction was quenched with $Na_2S_2O_3$ (sat. aq.) and $NaHCO_3$ (sat. aq.). The layers were separated and the aqueous phase was extracted with CH_2Cl_2 (3×2 mL). The combined organic extracts were washed with $NaHCO_3$ (sat. aq., 3×) and with brine, dried over $MgSO_4$ and concentrated under reduced pressure. The residue was taken up in pentane and the resulting suspension was filtered through celite in a pipette. The filtrate was evaporated under reduced pressure to afford the desired aldehyde **10** (42.7 mg, >100%, dr = 25/1) as a brownish liquid, which was used in the next step without further purification.

^{1}H-NMR ($CDCl_3$, 400.1 MHz): δ = 9.66 (d, *J* = 1.5 Hz, 1H), 6.29 (m_c, 1H), 4.00 (d, *J* = 5.6 Hz, 1H), 3.25 (s, 3H), 2.55 (m_c, 1H), 1.77 (d, *J* = 1.1 Hz, 3H), 1.09 (d, *J* = 7.0 Hz, 3H).

18

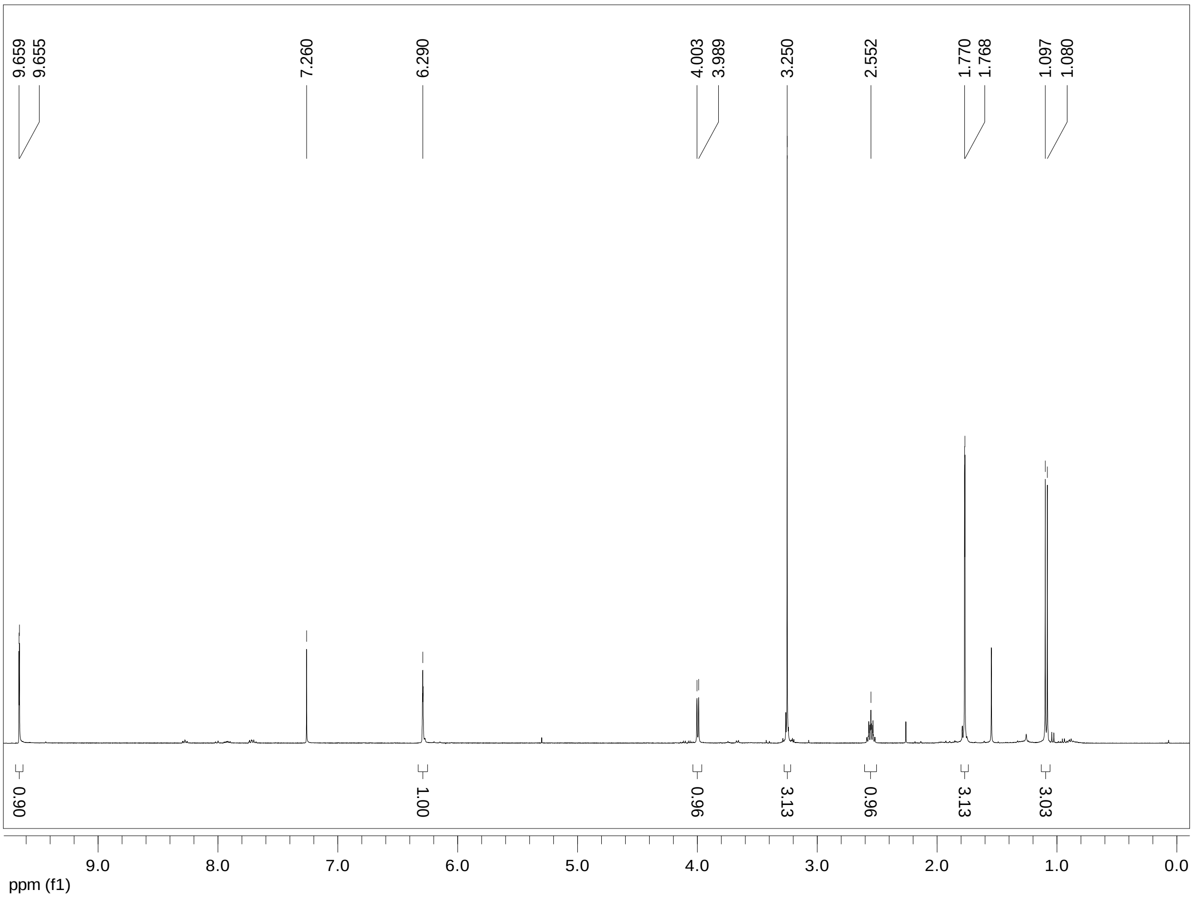

9.659
9.655
7.260
6.290
4.003
3.989
3.250
2.552
1.770
1.768
1.097
1.080
0.90
1.00
0.96
3.13
0.96
3.13
3.03
9.0
8.0
7.0
6.0
5.0
4.0
3.0
2.0
1.0
0.0
ppm (f1)

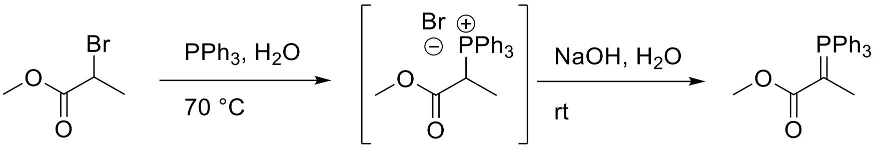

S7 **S8** **17**

Methyl 2-(triphenyl-l5-phosphaneylidene)propanoate (17): A mixture of methyl-2-bromopropionate (**S7**) (2.00 mL, 17.9 mmol, 1.10 eq) and triphenylphosphine (4.27 g, 16.3 mmol, 1.00 eq) in water (18.0 mL) was stirred at 70 °C for 2.5 h. Both the aqueous and the organic layer still contained PPh_3. The temperature was raised to 80 °C and the mixture was stirred for further 2 h. According to TLC, there was still a considerable amount of PPh_3. Therefore, the mixture was stirred at 75 °C overnight. After totally 23 h the mixture was allowed to cool to rt. Afterwards aqueous NaOH (38 mL 1 M, 2.3 eq) was added, which led to the immediate precipitation of a yellow solid. The suspension was stirred for 5 min, then CH_2Cl_2 (50 mL) was added and the layers were separated. The aqueous phase was extracted with CH_2Cl_2 (2×50 mL), washed with brine, dried over $MgSO_4$ and concentrated under reduced pressure. The crude was obtained as a yellow oil, which turned into a solid upon addition of pentane. The solid was washed with hexane (3×20 mL) and dried *in vacuo* to afford phosphonium ylide **17** (5.48 g, 97%) as a slightly yellow powder.

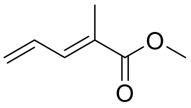

19

Methyl (E)-2-methylpenta-2,4-dienoate (19): To a solution of the phosphonium ylide **17** (62.1 g, 178 mmol, 1.00 eq) in CH_2Cl_2 (230 mL) was added acrolein (**18**) (11.9 mL, 178 mmol, 1.00 eq) dropwise at rt. The addition was ceased from time to time due to excessive boiling of the mixture. After the addition, the yellow solution was heated (47 °C oil bath) to maintain a gentle reflux. After 3 h, the mixture was allowed to cool to rt and stored overnight. Then it was concentrated under reduced pressure (*Vigreux*, short path distillation, "Hausvakuum", 55 °C oil bath), which took several hours. The concentrate was then cooled to 0 °C, pentane was added, the resulting suspension was filtered and the filter cake was washed with pentane. The combined filtrates were concentrated (Vigreux, short

path distillation, 50 °C oil bath) and the crude was purified by distillation (75-77 ° oil bath, 20 mbar, bp = 59-60 °C) to afford **19** as a colourless liquid (70%).

^{1}H-NMR ($CDCl_3$, 400.1 MHz): δ = 7.17 (m$_c$, 1H), 6.66 (ddd, *J* = 10.1 Hz, *J* = 11.3 Hz, *J* = 16.8 Hz, 1H), 5.56 (m$_c$, 1H), 5.45 (m$_c$, 1H), 3.77 (s, 3H), 1.96 (m$_c$, 3H).

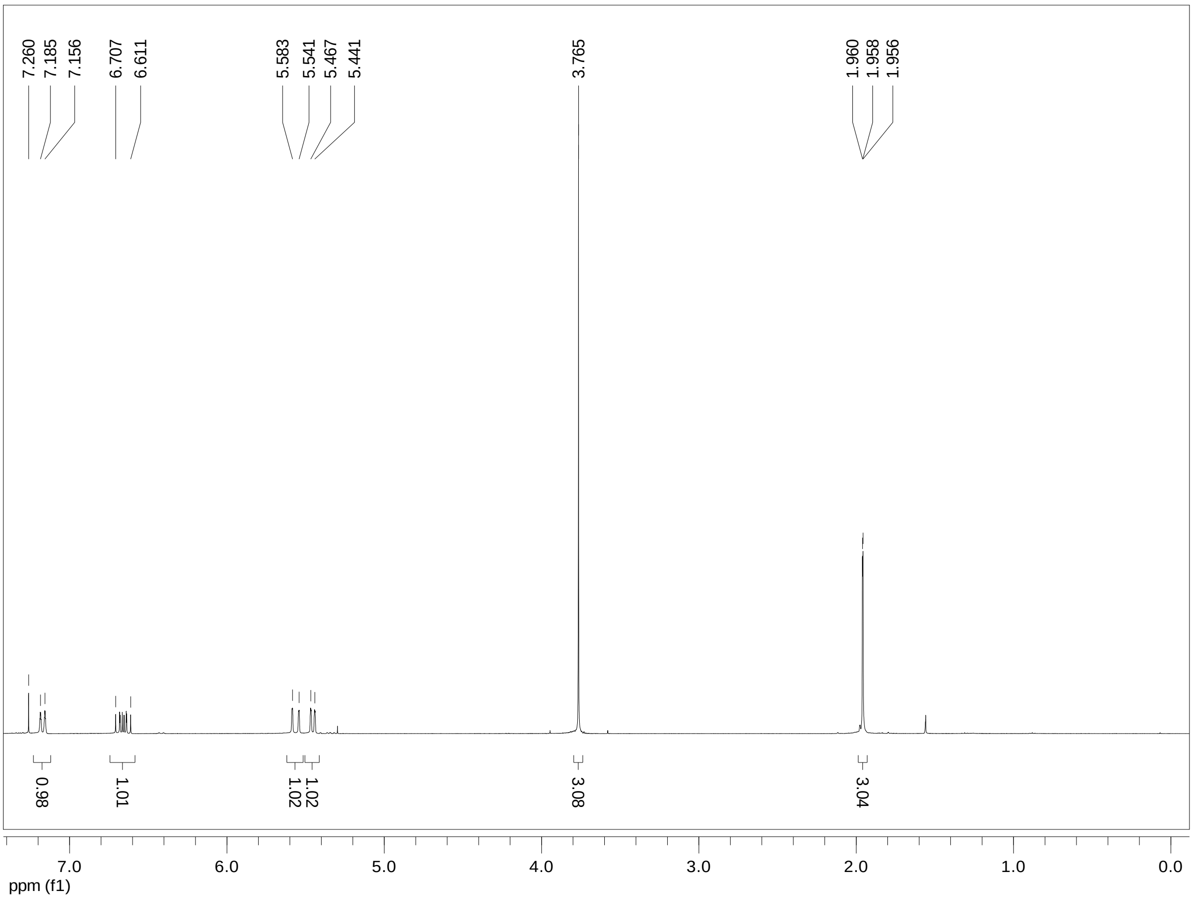

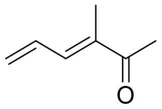

11

(*E*)-3-Methylhexa-3,5-dien-2-one (11): To a suspension (nearly a solution) of the ester **19** (100 mg, 0.793 mmol, 1.00 eq) and *N,O*-dimethylhydroxylamine hydrochloride (116 mg, 1.19 mmol, 1.50 eq) in THF (8 mL) was added MeMgCl (0.66 mL 3 M in THF, 1.98 mmol, 2.50 eq) over 15 min (syringe pump) at −15 °C. The mixture was aged for 10 min at the same temperature, warmed to 0 °C and more MeMgCl (0.66 mL 3 M in THF, 1.98 mmol, 2.50 eq) was added over 15 min. A reaction control by TLC showed clean conversion to two new spots, both more polar than the starting material. MS-Analysis indicated the presence of the *Weinreb* amide, whereas the ketone could not be identified. The mixture was stirred overnight, allowing the cooling bath to thaw. TLC and MS appeared unchanged. Therefore, the mixture was re-cooled to 0 °C and more MeMgCl (0.396 mL 3 M in THF, 1.19 mmol, 1.50 eq) was added over 15 min. After 20 min stirring at 0 °C, the mixture was allowed to warm to rt and stirred for further 2 h. Shortly after the addition, the TLC-spot presumably corresponding to the *Weinreb* amide had weakened, but even after 2 h it had not completely disappeared. Nevertheless, the reaction was quenched into HCl (20 mL 1 M) under rapid stirring. The layers were separated and the aqueous phase was extracted with ether (2×10 mL). The combined organic extracts were washed with water and concentrated under reduced pressure. The crude was purified by column chromatography (pent:Et_2O 10:1) to afford the desired ketone **11** (45.0 mg, 51%) as a colourless liquid.

TLC (Hex:EtOAc 10:1): R_f = 0.35

^{1}H-NMR ($CDCl_3$, 400.1 MHz): δ = 7.01 (bd, *J* = 11.0 Hz, 1H), 6.74 (ddd, *J* = 10.1 Hz, *J* = 10.9 Hz, *J* = 16.8 Hz, 1H), 5.62 (m_c, 1H), 5.51 (m_c, 1H), 2.36 (s, 3H), 1.90 (m_c, 3H).

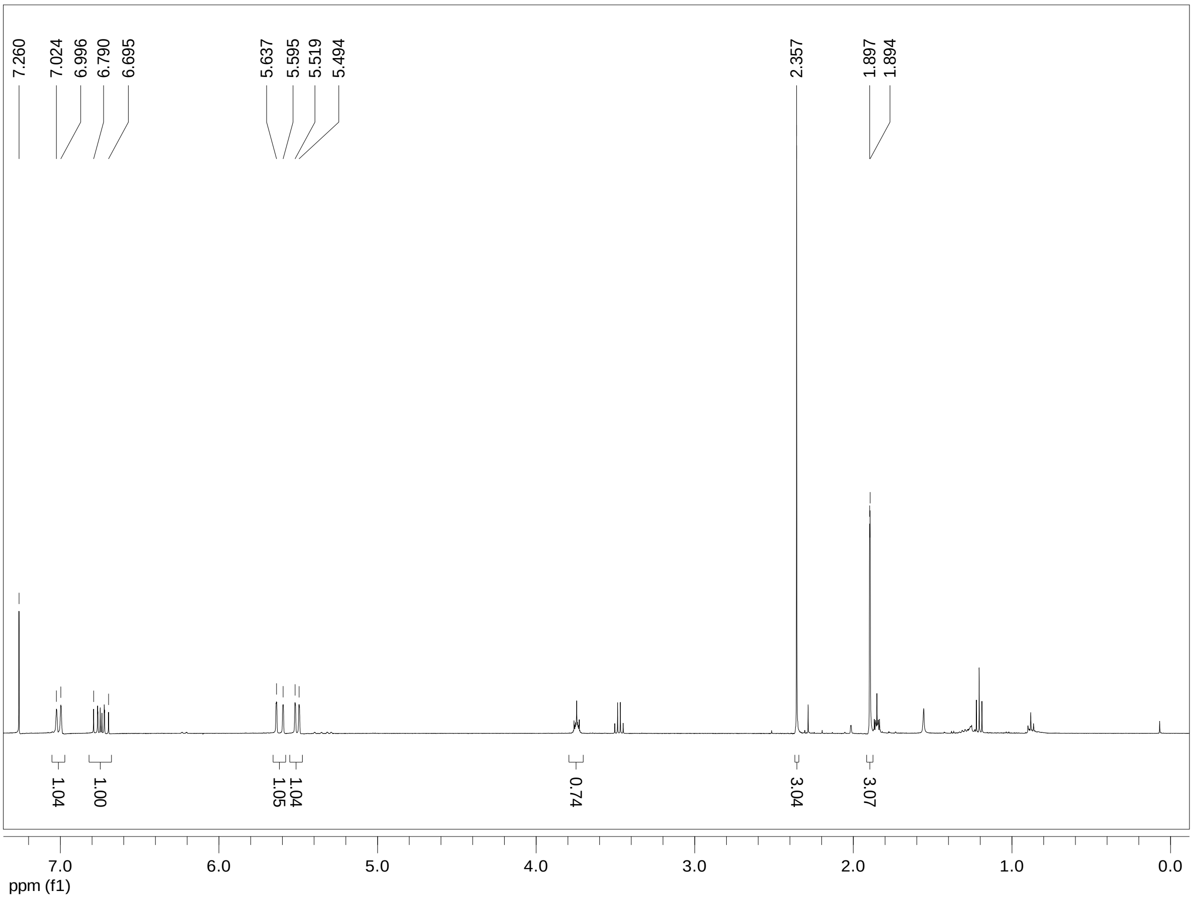
7.260
7.024
6.996
6.790
6.695
5.637
5.595
5.519
5.494
2.357
1.897
1.894
1.04
1.00
1.05
1.04
0.74
3.04
3.07
7.0
6.0
5.0
4.0
3.0
2.0
1.0
0.0
ppm (f1)

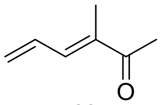

11

(*E*)-3-Methylhexa-3,5-dien-2-one (11): To a solution of the ester **19** (100 mg, 0.793 mmol, 1.00 eq) was added LiHMDS (1.59 mL 1.0 M in THF/Ethylbenzene, 1.59 mmol, 2.00 mmol) at −78 °C. After 15 min, MeMgCl (0.396 mL 3 M in THF, 1.20 mmol, 1.50 eq) was added slowly at the same temperature. The mixture was aged for 2.5 h at −78 °C. TLC showed only very little conversion of ester **19** to ketone **11**. Therefore, the cooling bath was allowed to warm to +10 °C over a period of 1.5 h. TLC then indicated high conversion. The mixture was recooled to 0 °C and more MeMgCl (0.132 mL 3 M in THF, 0.396 mmol, 0.50 eq) was added dropwise. After 1 h at 0 °C (TLC looked about the same as before, but seemingly some side product had formed) the reaction was quenched into ice cold HCl (20 mL 1 M). The layers were separated and the aqueous phase was extracted with ether (3×10 mL). The combined organic extracts were washed with brine, dried over $MgSO_4$ and concentrated under reduced pressure. TLC of the crude revealed, that most probably some overaddition had taken place and that other side products were present. The crude was purified by column chromatography (pent:Et_2O 10:1) to afford the desired ketone **11** (30.6 mg, 35%). The material was identical to the ketone obtained by *Weinreb* ketone synthesis (*vide supra*).

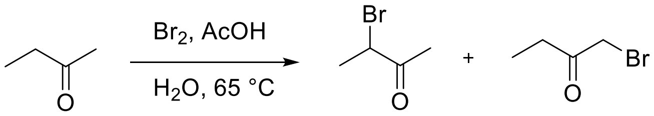

20 21a 21b

1-Bromobutan-2-one (21a): To a solution of 2-butanone (**20**) (18.6 mL, 208 mmol, 1.00 eq) and glacial acetic acid (11.9 mL, 208 mmol, 1.00 eq) in water (50 mL) was added bromine (10.7 mL, 208 mmol, 1.00 eq) dropwise at 65 °C (oil bath at 75 °C) over 1 h 50 min. The first few drops of Br_2 coloured the mixture deep orange and the colour persisted for several minutes before it suddenly faded. The colour of the following drops then discharged quickly. During the addition the solution turned into a biphasic mixture, the pale orange organic phase being at the bottom. After the addition, the mixture was stirred for further 30 min at 65 °C and was then allowed to cool to rt. Cold water was

added and the mixture was neutralized with Na_2CO_3 (ca. 25 g). The layers were separated and the organic phase was dried over $MgSO_4$. The crude was purified by distillation (bath temp.: 100-135 °C, p=100 mbar) to afford the desired product **21a** in a ca. 11:1 mixture with its regioisoner 21b (as an almost colourless liquid (12.8 g, 41%, bp 68-69 °C).

^{1}H-NMR ($CDCl_3$, 400.1 MHz): δ = 4.39 (q, *J* = 6.8 Hz, 1H), 2.37 (s, 3H), 1.74 (d, *J* = 6.8 Hz, 3H).

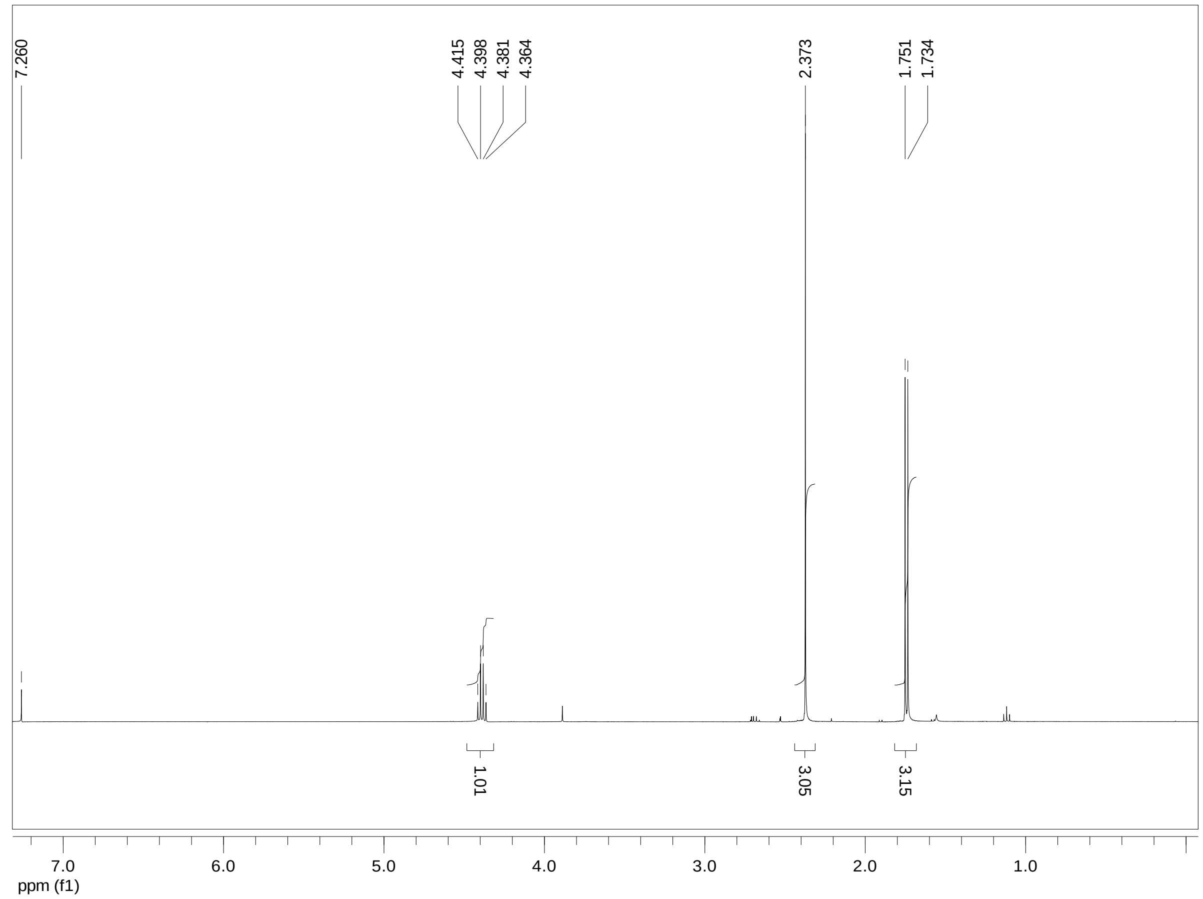

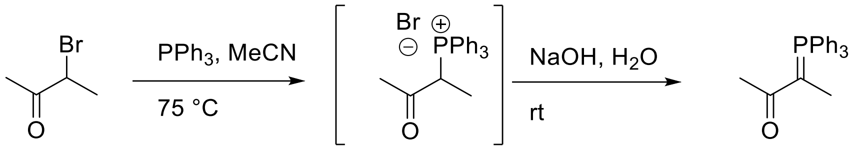

21a S7 22

3-(Triphenyl-l5-phosphaneylidene)butan-2-one (22): To a solution of PPh_3 (6.51 g, 24.8 mmol, 1.25 eq) in MeCN (40 mL) was added the α-bromoketone **21a** (3.00 g, 19.9 mmol, 1.00 eq) at rt and the mixture was heated to 70 °C for 19 h. The nearly colourless solution was allowed to cool to rt. Then phenolphthalein (5 mg) was added and NaOH (4 M) was added until a pink colour persisted. CH_2Cl_2 (50 mL) was added and the layers were separated (organic layer was on top of the aqueous layer). The pink aqueous phase was extracted with CH_2Cl_2 (2×30 mL). The combined organic layers were washed with brine, dried over $MgSO_4$ and concentrated under reduced pressure, yielding a pale yellow solid (7.74 g, ca. 70% wt/wt ylid along with PPh_3, 5.45 g, 82%, 4 peaks in ^{31}P-NMR!). The crude was transferred to a glas filter, triturated with hexane and dried *in vacuo* to afford **22** as a pale yellow solid (6.64 g, ca. 79% wt/wt along with PPh_3 and an unknown impurity, 5.22 g, 79%).

^{31}P-NMR ($CDCl_3$, 400.1 MHz): δ = 29.0 (?), 17.6 (main peak), 14.8 (?), −5.4 (PPh_3). **^{1}H-NMR** ($CDCl_3$, 400.1 MHz): δ = 7.72-7.37 (m, 15H), 2.13 (s, 3H), 1.66 (d, $^{3}J_{HP}$ = 15.5 Hz, 3H).

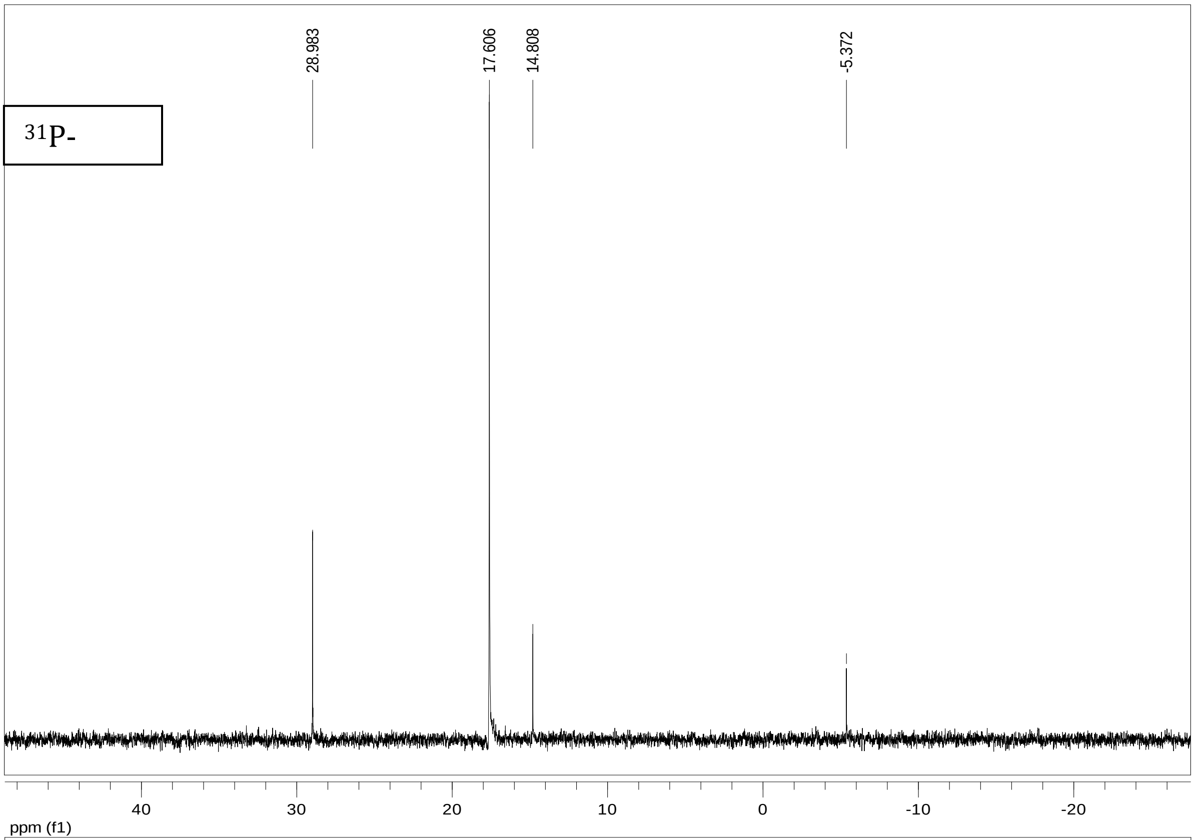

31P-
28.983
17.606
14.808
-5.372
40
30
20
10
0
-10
-20
ppm (f1)

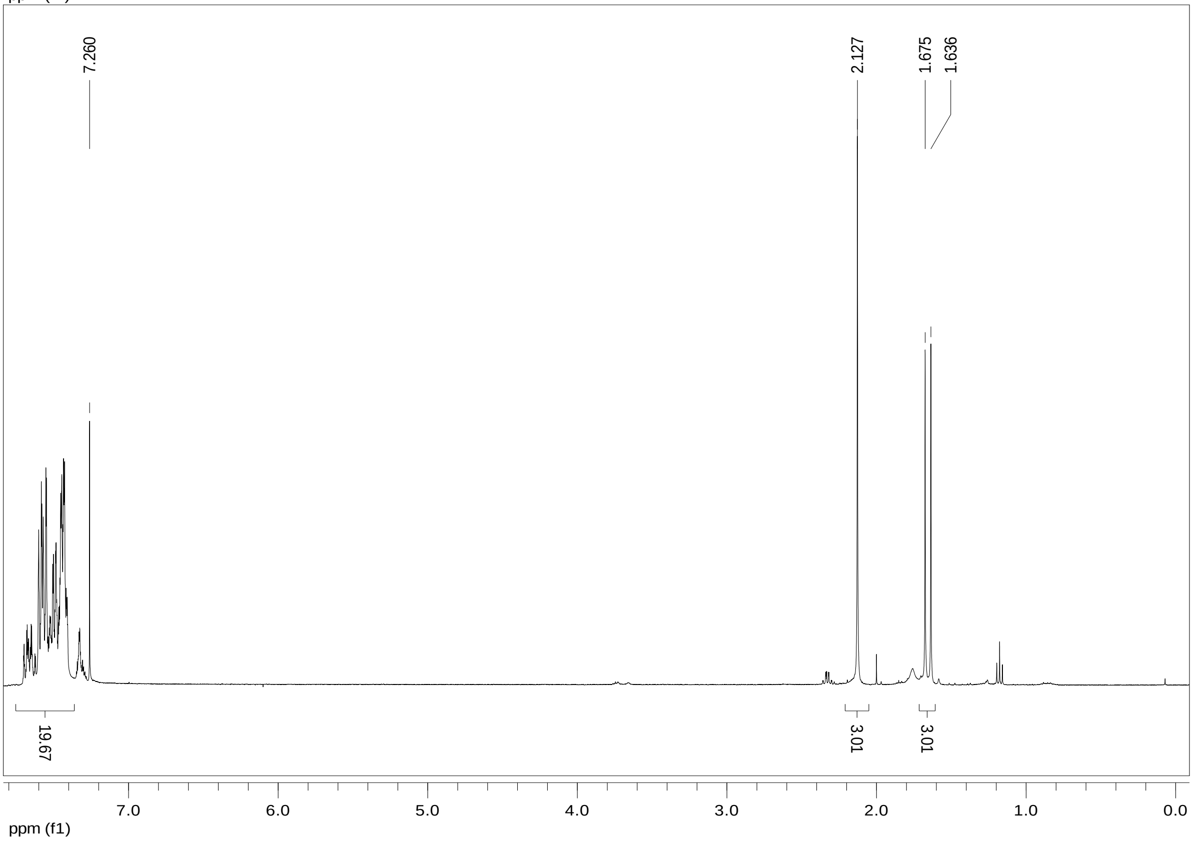

7.260
2.127
1.675
1.636
19.67
3.01
3.01
7.0
6.0
5.0
4.0
3.0
2.0
1.0
0.0
ppm (f1)

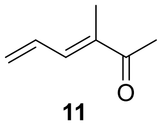

11

(*E*)-3-Methylhexa-3,5-dien-2-one (11): To a solution of the phosphonium ylide **22** (5.22 g, 15.7 mmol, 1.00 eq) in CH_2Cl_2 (20 mL) was added acrolein (**18**) (1.10 mL, 16.5 mmol, 1.05 eq) dropwise at rt. The temperature increased only 2-3 °C. After the addition, the yellow solution was stirred at rt for 5 min before it was heated to reflux (bath temp. 47 °C). After 3 h 45 min, the main spot on TLC was the desired product. However, the ylid was still present in the mixture referring to ESI-MS. Therefore, the mixture was refluxed overnight. ESI-MS and TLC appeared unchanged. Nevertheless, the mixture was allowed to cool to rt, transferred to a round bottom flask and ca. 15 mL of the solvent were removed on a rotary evaporator (800 mbar). The remaining yellow liquid was cooled to 0 °C, pentane (75 mL) was added and the suspension was stored in a fridge overnight. The precipitate was filtered off and washed with pentane. The combined filtrates were concentrated at the rotary evaporator. The crude was then purified by kugelrohr distillation to afford the dienone **11** (993 mg, 57%) as a pale green liquid. For analytical data, see above.

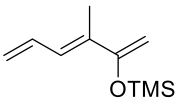

23

(*E*)-Trimethyl((3-methylhexa-1,3,5-trien-2-yl)oxy)silane (23): To a solution of the methyl ketone **11** (100 mg, 0.908 mmol, 1.00 eq), NEt_3 (0.189 mL, 1.36 mmol, 1.50 eq) and TMS-Cl (0.173 mL, 1.36 mmol, 1.50 eq) in MeCN (0.4 mL) was added a solution of NaI (204 mg, 1.36 mmol, 1.50 eq) in MeCN (1.5 mL) at 0 °C. The cooling was removed, the pale yellow suspension was allowed to warm to rt and then stirred at 90 °C overnight. The colour had changed to orange. After 18 h stirring at the same temperature, the mixture was re-cooled to 0 °C. Then cold pentane and ice water were added successively. The layers were separated and the aqueous phase was extracted with pentane (2×2 mL). The combined organic extracts were washed with NH_4Cl (sat. aq., 2×2 mL), dried over $MgSO_4$ and concentrated under reduced pressure. The crude silyl enolether **23** (145 mg, 98% wt/wt along with s.m. and pentane, 142 mg, 86%) was used without purification.\

^{1}H-NMR ($CDCl_3$, 400.1 MHz): δ = 6.67 (ddd, *J* = 10.0 Hz, *J* = 11.3 Hz, *J* = 16.5 Hz, 1H), 6.54 (bd, *J* = 11.3 Hz, 1H), 5.32 (dd, *J* = 1.9 Hz, *J*=16.5 Hz, 1H), 5.19 (dd, *J* = 1.7 Hz, *J* = 9.9 Hz, 1H), 4.56 (d, *J* = 1.1 Hz, 1H), 4.37 (bs, 1H), 1.88 (bs, 3H), 0.23 (bs, 9H).

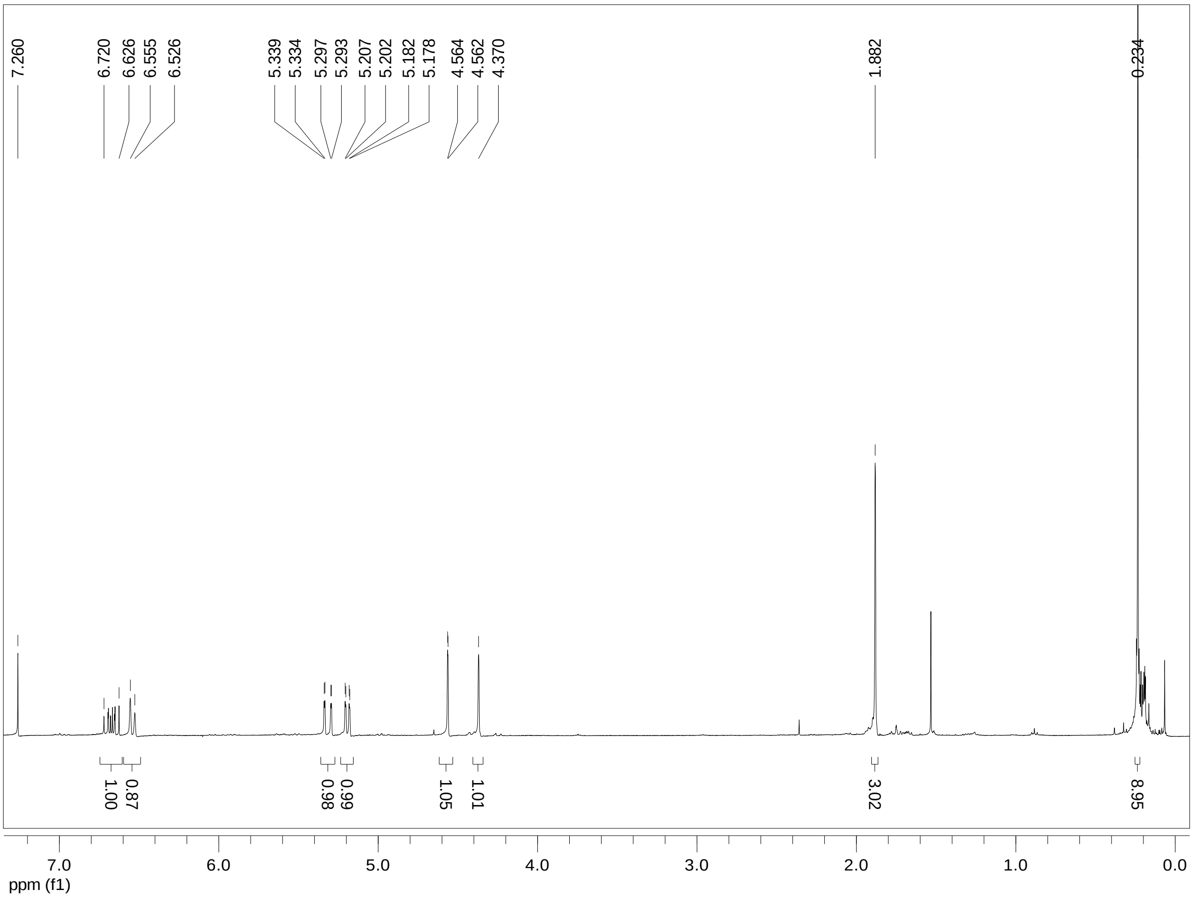

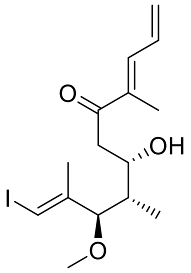

24

(3*E*,7*S*,8*S*,9*R*,10*E*)-7-Hydroxy-11-iodo-9-methoxy-4,8,10-trimethylundeca-1,3,10-trien-5-one (24): A solution of the aldehyde **11** (41.4 mg, 0.154 mmol, 1.00 eq) in CH_2Cl_2 (1.5 mL) was dried over molecular sieves (3-4 pellets, 3 Å) for a few minutes. Then $TiCl_4$ (0.154 mL 1 M in CH_2Cl_2, 0.154 mmol, 1.00 eq) was added dropwise at −78 °C. The deep yellow solution was aged for 3 min before a solution of the TMS enolether **23** (56.3 mg, 0.309 mmol, 2.00 eq) in CH_2Cl_2 (0.5 mL, dried over molecular sieves, flask rinsed with 0.2 mL) was added dropwise at −78 °C. The colour immediately turned dark red to brown. The solution was stirred for 20 min at the same temperature. Then H_2O (0.3 mL), 1 M NaOH (0.3 mL) and H_2O (0.9 mL) were added subsequently, and the layers were separated. The aqueous phase was extracted with CH_2Cl_2 (3×2 mL). The combined organic extracts were washed with brine, dried over $MgSO_4$ and concentrated under reduced pressure. The crude was purified by column chromatography (hex:EtOAc 10:1), which afforded the desired product **24** (7.2 mg, dr=4:1, 12%) as a nearly colourless oil along with some parent aldehyde (9.2 mg, dr=10:1, 22%).

TLC (hex:EtOAc 5:1): R_f = 0.24

^{1}H-NMR ($CDCl_3$, 400.1 MHz): δ = 7.03 (bd, *J* = 10.9 Hz, 1H), 6.74 (m_c, 1H), 6.25 (bs, 1H), 5.65 (d, *J* = 16.8 Hz, 1H), 5.54 (d, *J* = 10.1 Hz, 1H), 4.08 (ddd, *J* = 2.4 Hz, *J* = 5.7 Hz, *J* = 8.5 Hz, 1H), 3.79 (d, *J* = 6.8 Hz, 1H), 3.24 (s, 3H), 3.19 (d, *J* = 2.4 Hz, 1H), 2.91 (dd, *J* = 8.9 Hz, *J* = 17.1 Hz, 1H), 2.77 (dd, *J* = 3.5 Hz, *J* = 17.1 Hz, 1H), 1.90 (d, *J* = 0.7 Hz, 3H), 1.74 (d, *J* = 1.0 Hz, 3H), 1.70 (m_c, 1H), 0.98 (d, *J* = 6.9 Hz, 3H).

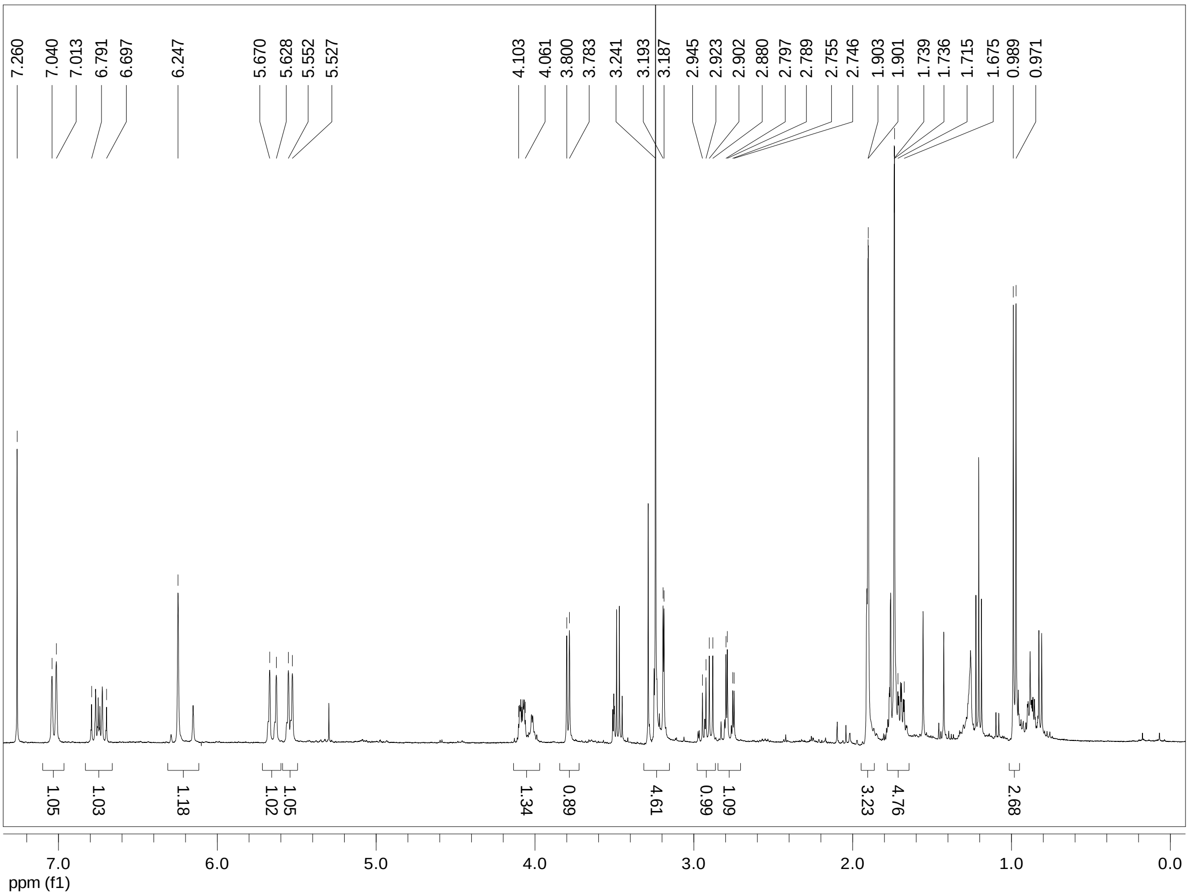
7.260
7.040
7.013
6.791
6.697
6.247
5.670
5.628
5.552
5.527
4.103
4.061
3.800
3.783
3.241
3.193
3.187
2.945
2.923
2.902
2.880
2.797
2.789
2.755
2.746
1.903
1.901
1.739
1.736
1.715
1.675
0.989
0.971
1.05
1.03
1.18
1.02
1.05
1.34
0.89
4.61
0.99
1.09
3.23
4.76
2.68
7.0
6.0
5.0
4.0
3.0
2.0
1.0
0.0
ppm (f1)

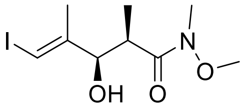

S8

(2*R*,3*R*,*E*)-3-Hydroxy-5-iodo-N-methoxy-N,2,4-trimethylpent-4-enamide (S8): To a suspension of *N,O*-dimethylhydroxylamine hydrochloride (136 mg, 1.40 mmol, 3.00 eq) in THF (2.3 mL) was added $AlMe_3$ (0.687 mL 2 M in heptanes, 1.37 mmol, 2.95 eq) at 0 °C. After 10-15 min the gas evolution had almost ceased and the cooling was removed. The colourless solution was aged for 1 h and then cooled to −40 °C (MeCN/dry ice). Afterwards a solution of the oxazolidinone **15** (200 mg, 0.466 mmol, 1.00 eq) in THF (1 mL, rinsed with 2×0.2 mL) was added and the cooling was removed. After 2 h 25 min the reaction was quenched with *Rochelle's* salt (ca. 3 *Pasteur* pipettes) at −10 °C (ice/NaCl). The suspension was allowed to warm to rt and then stirred for another 2.5 h. CH_2Cl_2 (10 mL) was added and the layers were separated. The aqueous phase was extracted with CH_2Cl_2 (2×10 mL). The combined organic extracts were washed with brine, dried over $MgSO_4$ and concentrated at under reduced pressure. The crude was then purified by column chromatography (hex:EtOAc 1:1) to afford the desired *Weinreb* amide **S8** (140 mg, 96%) as a colourless crystalline solid, along with recovered *Evans* oxazolidinone **S4** (80.5 mg, 98%) as a colourless solid.

TLC (hex:EtOAc 1:1): R_f = 0.36

^{1}H-NMR ($CDCl_3$, 400.1 MHz): δ = 6.43 (m_c, 1H), 4.42 (bs, 1H), 4.24 (bs, 1H), 3.73 (s, 3H), 3.21 (s, 3H), 3.10 (d, *J*=6.5 Hz, 1H), 1.79 (s, 3H), 1.08 (d, *J*=7.1 Hz, 3H).

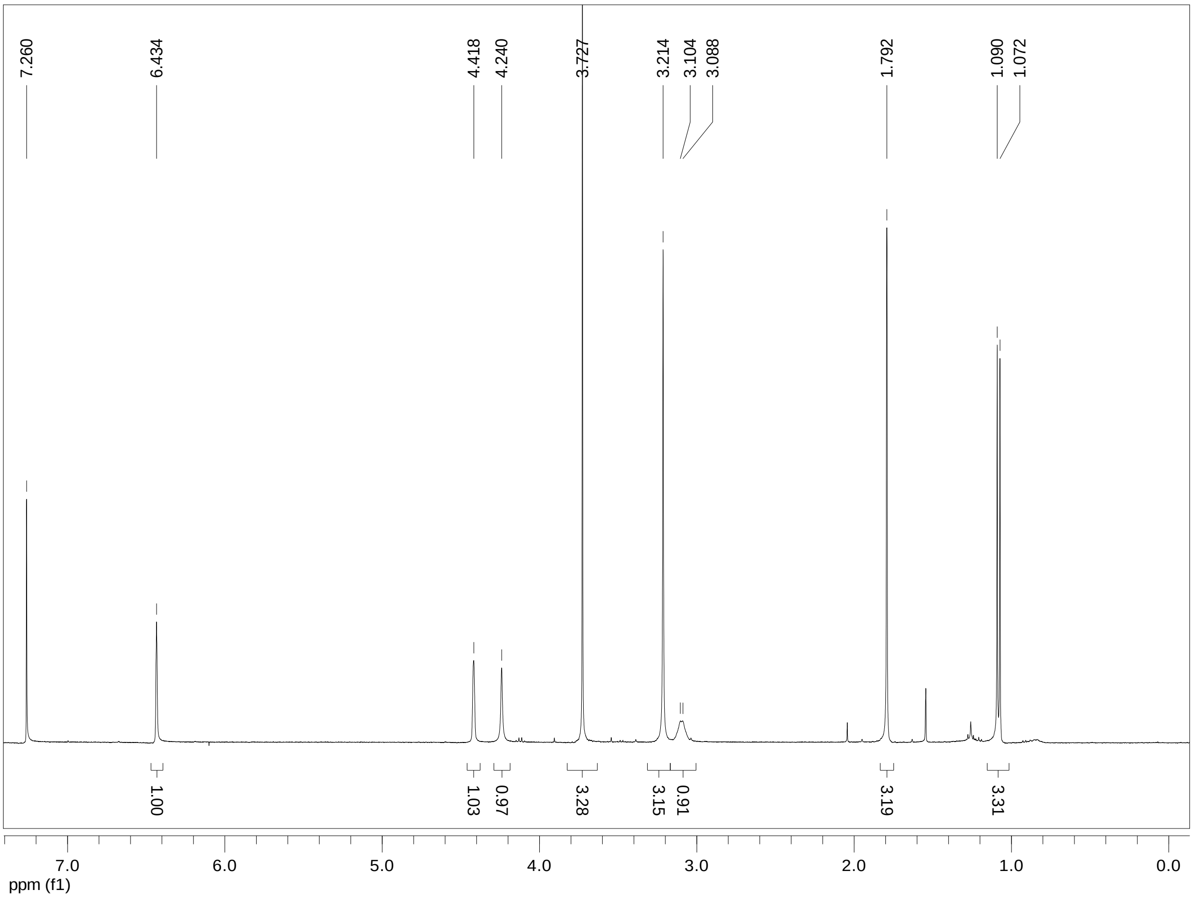
7.260
6.434
4.418
4.240
3.727
3.214
3.104
3.088
1.792
1.090
1.072
1.00
1.03
0.97
3.28
3.15
0.91
3.19
3.31
7.0
6.0
5.0
4.0
3.0
2.0
1.0
0.0
ppm (f1)

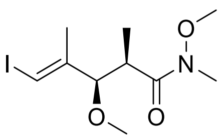

S9

(2*R*,3*R*,*E*)-5-Iodo-N,3-dimethoxy-N,2,4-trimethylpent-4-enamide (*S9*): To a cooled (0 °C) solution of the alcohol **S8** (1.43 g, 4.55 mmol, 1.00 eq) and MeI (2.84 mL, 45.6 mmol, 10.0 eq) in THF:DMF (3:1, 44 mL) was added NaH (273 mg 60% dispersion in mineral oil, 6.84 mmol, 1.50 eq). A white precipitate formed with a short delay. The white suspension was stirred for 2 h at 0 °C. Then the reaction was quenched with pH 7 phosphate buffer. Water (100 mL) and CH_2Cl_2 (30 mL) were added and the layers were separated. The aqueous phase was extracted with CH_2Cl_2 (3×30 mL). The combined organic extracts were washed with water (50 mL) and brine, dried over $MgSO_4$ and concentrated under reduced pressure. The crude was purified by column chromatography (hex:EtOAc 2:1) to afford the desired methyl ether **S9** (1.42 g, 95%) as a nearly colourless liquid.

TLC (hex:EtOAc 1:1): R_f = 0.57

^{1}H-NMR ($CDCl_3$, 400.1 MHz): δ = 6.22 (bs, 1H), 3.80 (d, *J*=9.6 Hz, 1H), 3.65 (s, 3H), 3.27-3.06 (m, 1H), 3.22 (s, 3H), 3.13 (s, 3H), 1.76 (s, 3H), 1.08 (d, *J*=6.8 Hz, 3H).

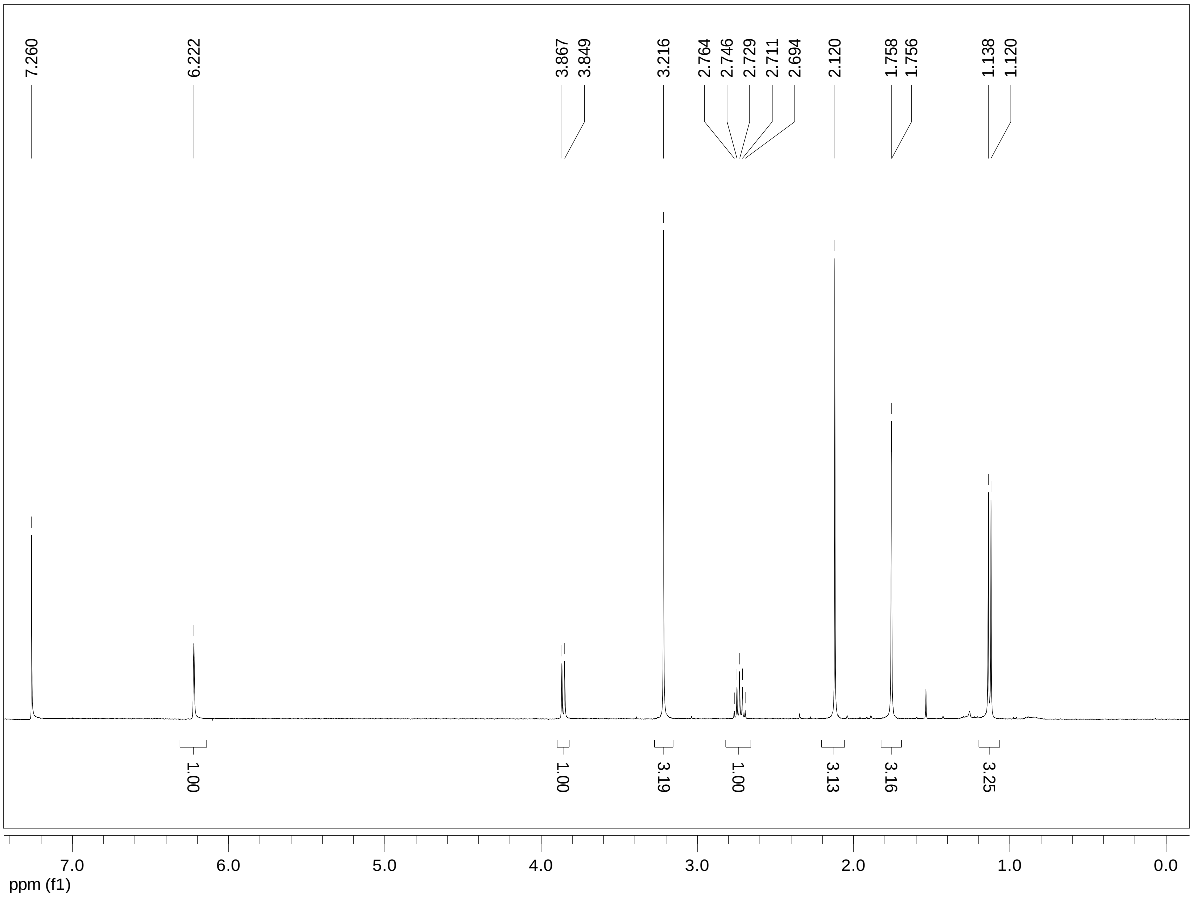
7.260
6.222
3.867
3.849
3.216
2.764
2.746
2.729
2.711
2.694
2.120
1.758
1.756
1.138
1.120
1.00
1.00
3.19
1.00
3.13
3.16
3.25
7.0
6.0
5.0
4.0
3.0
2.0
1.0
0.0
ppm (f1)

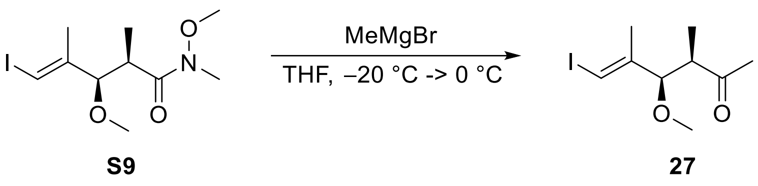

(3*R*,4*R*,*E*)-6-Iodo-4-methoxy-3,5-dimethylhex-5-en-2-one (27): To a cooled solution (ca. −20 °C, ice/NaCl) of the *Weinreb* amide **S9** (1.42 g, 4.34 mmol, 1.00 eq) in THF (55 mL) was added MeMgBr (5.79 mL 2.7 M in Et_2O, 15.6 mmol, 3.60 eq) over <5 min. The reaction mixture was stirred for 2.5 h, allowing for the cooling bath to thaw (it was still at ca. −5 °C). Afterwards the reaction was quenched with NH_4Cl (sat. aq.). Water (100 mL) was added, the mixture was shaken and the layers were separated. The aqueous phase was extracted with ether (3×30 mL). The combined organic extracts were washed with brine, dried over $MgSO_4$ and concentrated at the rotavap. The crude was purified by column chromatography (hex:EtOAc 10:1) to afford the desired ketone **27** (1.135 g, 93%) as a pale yellow liquid.

TLC (Hex:EtOAc 5:1): R_f = 0.42
^{1}H-NMR ($CDCl_3$, 400.1 MHz): δ = 6.22 (m_c, 1H), 3.86 (d, *J*=7.1 Hz, 1H), 3.22 (s, 3H), 2.73 (p, *J*=7.0 Hz, 1H), 2.12 (s, 3H), 1.76 (d, *J*=0.9 Hz, 3H), 1.13 (d, *J*=7.0 Hz, 3H).
^{13}C-NMR ($CDCl_3$, 100.6 MHz): δ = 209.9, 145.3, 86.4, 80.2, 57.0, 50.1, 29.3, 20.0, 12.2.

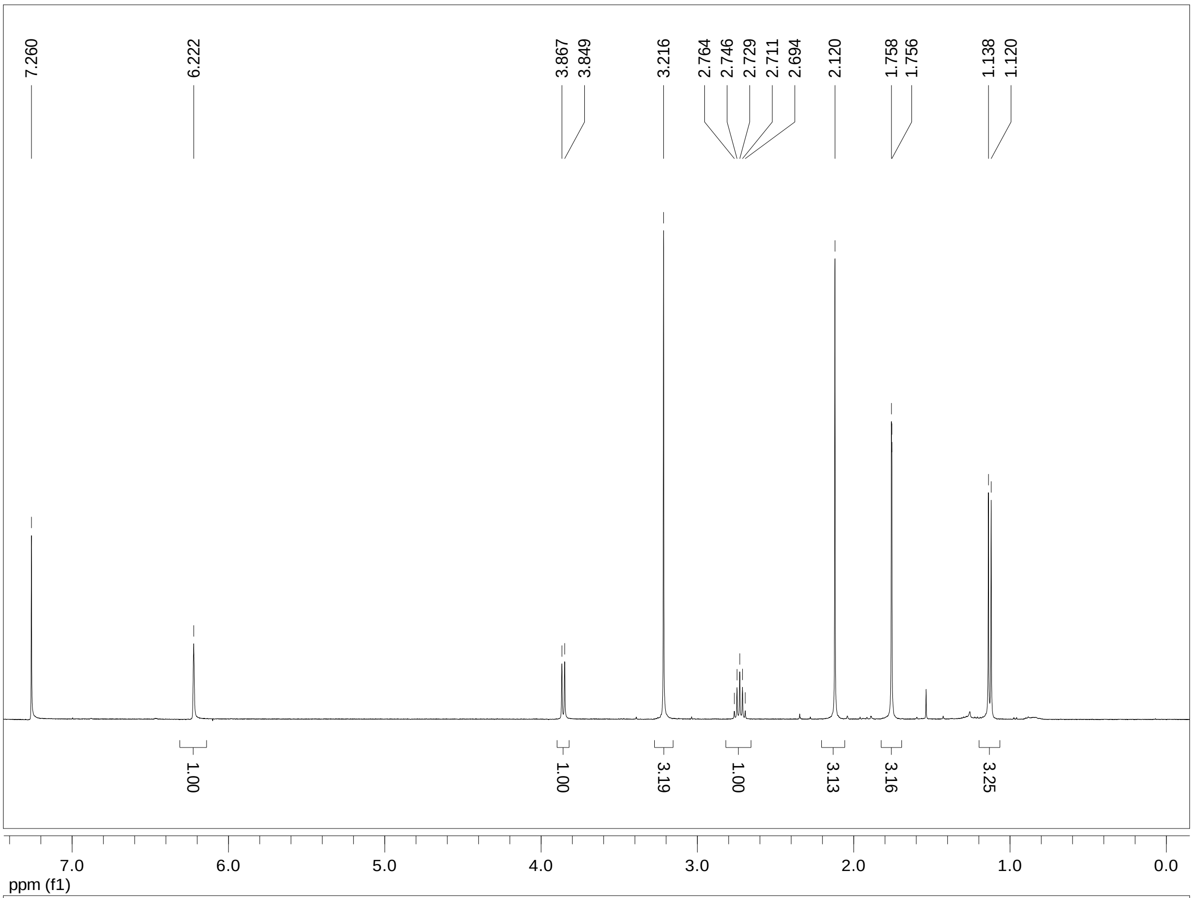
7.260
6.222
3.867
3.849
3.216
2.764
2.746
2.729
2.711
2.694
2.120
1.758
1.756
1.138
1.120
1.00
1.00
3.19
1.00
3.13
3.16
3.25
7.0
6.0
5.0
4.0
3.0
2.0
1.0
0.0
ppm (f1)

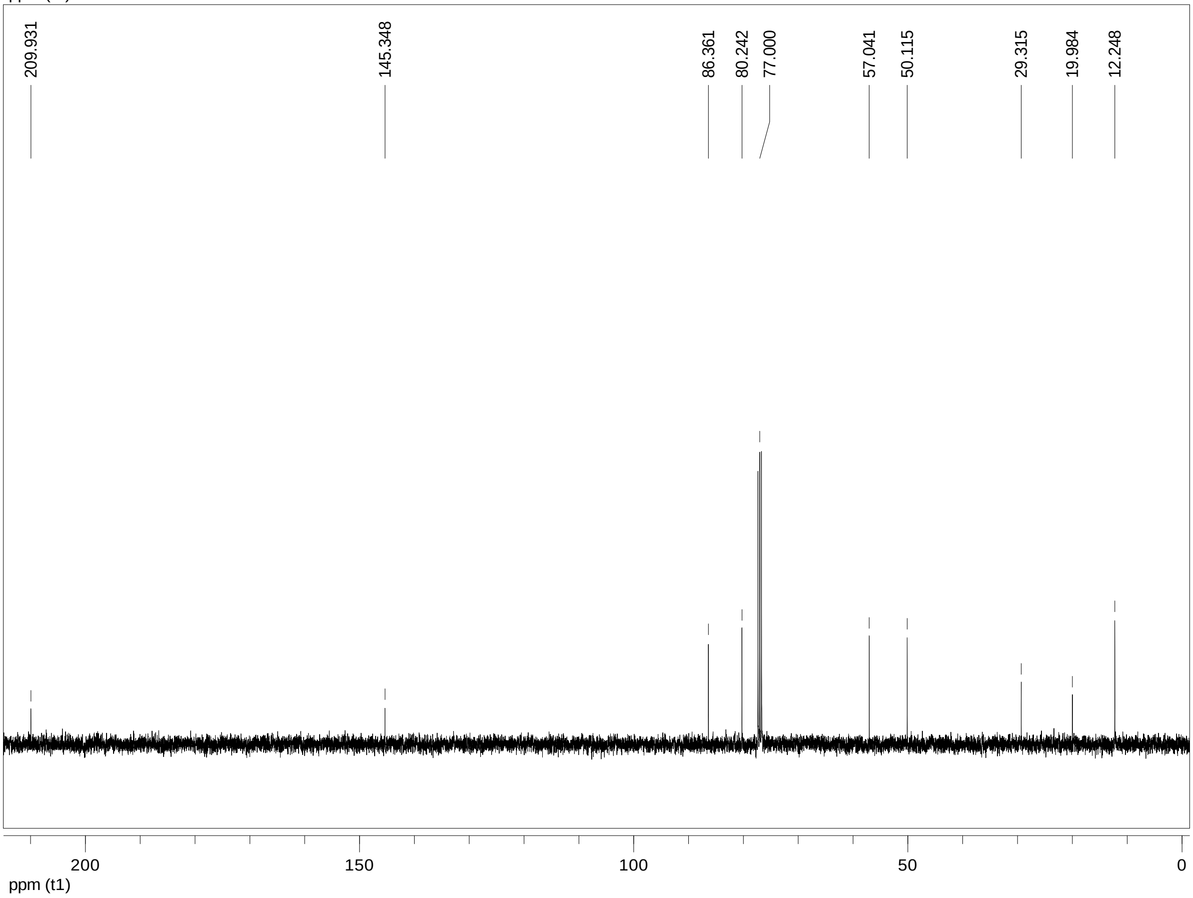
209.931
145.348
86.361
80.242
77.000
57.041
50.115
29.315
19.984
12.248
200
150
100
50
0
ppm (t1)

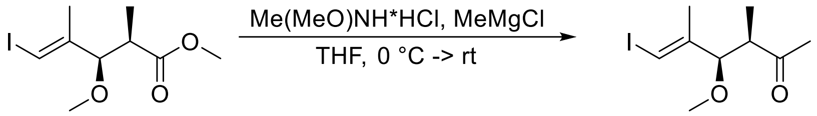

19 **27**

(3*R*,4*R*,*E*)-6-Iodo-4-methoxy-3,5-dimethylhex-5-en-2-one (27): To a suspension (almost a solution) of the ester **19** (100 mg, 0.335 mmol, 1.00 eq) and the hydroxylamine salt (40.8 mg, 0.418 mmol, 1.25 eq) in THF (4 mL) was added MeMgCl (0.738 mL 3 M in THF, 2.21 mmol, 6.60 eq) dropwise at −10 °C (ice/NaCl). The mixture was stirred for 2 h, allowing the cooling bath to thaw. Then the cooling was removed and the reaction was stirred at rt for another 3 h 25 min. The colour turned yellow. Afterwards the solution was poured into NH_4Cl (sat. aq.). The layers were separated and the aqueous phase was extracted with ether (3×10 mL). The combined organic layers were washed with brine, dried over $MgSO_4$ and concentrated under reduced pressure. The crude was purified by column chromatography (hex:EtOAc 10:1) to afford the desired ketone **27** (68.4 mg, 72%) as a colourless oil.

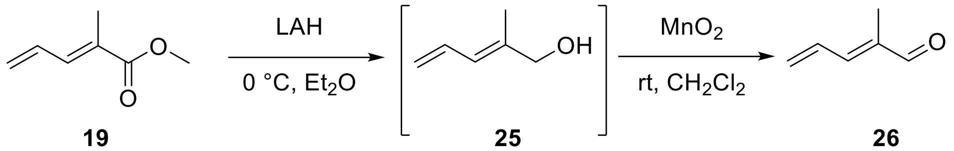

19 **25** **26**

(E)-2-Methylpenta-2,4-dienal (26):

Reduction: To a cooled (0 °C) solution of the ester **19** (1.00 g, 7.93 mmol, 1.00 eq) in Et_2O (40 mL) was added $LiAlH_4$ (602 mg, 15.9 mmol, 2.00 eq) in one portion. The mixture was stirred at 0 °C for 30 min. Afterwards the reaction was quenched with water (8 mL), followed by 1 M NaOH (8 mL) and more water (24 mL). The layers were separated and the aqueous phase was extracted with Et_2O (3×15 mL). The combined organic extracts were washed with brine, dried over $MgSO_4$ and concentrated under reduced pressure. As the substance turned out to be very volatile, not all of the solvent was removed and the crude allylic alcohol **25** was directly used in the next step.

<u>Oxidation:</u> To a solution of the allylic alcohol **25** (estimated 778 mg, 7.93 mmol, 1.00 eq) in CH_2Cl_2 (25 mL) were added molecular sieves (3 Å, beads) and activated MnO_2 (13.8 g, 159 mmol, 20.0 eq) at rt. A slight warming was observed. The suspension was stirred for 3 h at the same temperature. Afterwards it was filtered through a pad of celite, topped with a few mm of silica. The filter was washed thoroughly with CH_2Cl_2 and Et_2O. The combined filtrates were concentrated under reduced pressure and the crude aldehyde **26** (840 mg, 71% wt/wt along with Et_2O and CH_2Cl_2, 597 mg, 78% over two steps) was used directly for the next step.

^{1}H-NMR ($CDCl_3$, 400.1 MHz): δ = 9.48 (s, 1H), 6.90-6.77 (m, 2H), 5.77-5.66 (m, 1H), 5.65-5.56 (m, 1H), 1.87 (s, 3H).

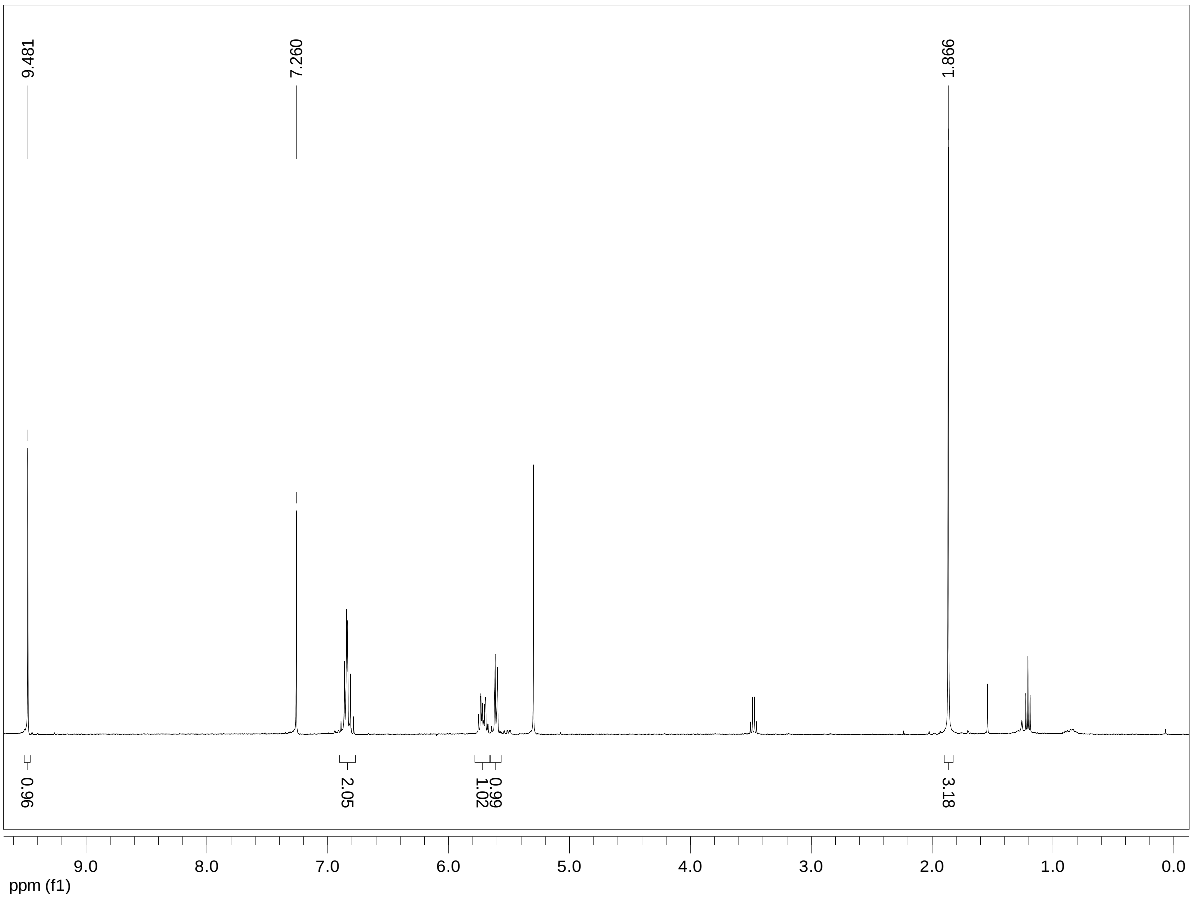

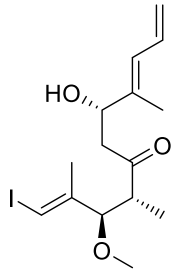

28

(1*E*,3*R*,4*R*,7*S*,8*E*)-7-Hydroxy-1-iodo-3-methoxy-2,4,8-trimethylundeca-1,8,10-trien-5-one (28): To a cooled (−78 °C) solution of (+)-DIP-Cl (1.19 g, 3.71 mmol, 2.00 eq) in CH_2Cl_2 (5 mL) was added NEt_3 (0.618 mL, 4.45 mmol, 2.40 eq) followed by a solution of the ketone **27** (523 mg, 1.85 mmol, 1.00 eq) in CH_2Cl_2 (2 mL, rinsed with 2×2 mL). Ca. 15 min after the addition of **27**, a white precipitate formed. The thick suspension was stirred for 3 h 15 min at −78 °C. Afterwards a solution of the aldehyde **26** (356 mg, 3.71 mmol, 2.00 eq) in CH_2Cl_2 (5.6 mL), which had been dried over molecular sieves for 10 min, was added slowly, whereupon the colour turned increasingly orange. The mixture was stirred for another 2 h at −78 °C (the colour faded towards a pale yellow) and was then allowed to stand in the freezer (−18 °C) for 3 d. During this time the salts were dissolved and an orange, slightly turbid solution resulted. The reaction was quenched with pH 7 phosphate buffer and the layers were separated. The aqueous phase was diluted with water and extracted with CH_2Cl_2 (3×30 mL). The combined extracts were concentrated under reduced pressure and the residue was dissolved in MeOH (10 mL). Then pH 7 phosphate buffer (2 mL) was added. The mixture was cooled to 0 °C and H_2O_2 (30%, 2.5 mL) was added. The cooling was removed after a few minutes and the mixture was stirred for 1 h at rt. Afterwards it was poured into water (200 mL). CH_2Cl_2 (30 mL) was added and the layers were separated. The aqueous phase was then extracted with CH_2Cl_2 (3×30 mL). The combined organic extracts were washed with $NaHCO_3$ (sat. aq.) and brine, dried over $MgSO_4$ and concentrated under reduced pressure. The crude (dr ca. 12:1) was purified by column chromatography (hex:EtOAc 10:1→5:1) to afford the desired β-hydroxy ketone **28** (1.10 g, 37% wt/wt along with isopinocampheol, 407 mg, 58%, dr=14.8:1) as a nearly colourless oil.

TLC (hex:EtOAc 5:1): R_f = 0.16

^{1}H-NMR ($CDCl_3$, 400.1 MHz): δ = 6.57 (ddd, *J* = 10.2 Hz, *J* = 10.8 Hz, *J* = 16.8 Hz, 1H, CH_2=C*H*), 6.25-6.21 (m, 1H, C*H*I), 6.13 (d, *J* = 10.9 Hz, 1H, CH_2=CHC*H*), 5.23 (dd, *J* = 1.9 Hz,

J = 16.8 Hz, 1H, CH*H*=CH, *trans*), 5.12 (dd, *J* = 1.7 Hz, *J* = 10.1 Hz, 1H, C*H*H=CH, *cis*), 4.50 (m, 1H, C*H*OH), 3.84 (dd, *J* = 0.5 Hz, *J* = 7.3 Hz, 1H, C*H*OMe), 3.22 (s, 3H, OCH_3), 2.88 (d, *J* = 3.0 Hz, 1H, O*H*), 2.77 (p, *J* = 7.0 Hz, 1H, C*H*Me), 2.68 (dd, *J* = 9.3 Hz, *J* = 17.5 Hz, 1H, CH_2C=O), 2.55 (dd, *J* = 2.7 Hz, *J* = 17.5 Hz, 1H, CH_2C=O), 1.76 (d, *J* = 1.0 Hz, 3H, CH=CCH_3), 1.75 (d, *J* = 1.1 Hz, 3H, CHI=CCH_3), 1.13 (d, *J* = 8.0 Hz, 3H, CHCH_3).

^{13}C-NMR ($CDCl_3$, 100.6 MHz): δ = 212.7 (*C*=O), 145.3 (C_q), 138.3 (C_q), 132.4 (CH_2=*C*H), 125.8 (CH_2=CH*C*H), 117.6 (H_2C=CH), 86.4 (*C*HOMe), 80.6 (*C*HI), 72.1 (*C*HOH), 57.0 (O*C*H_3), 50.2 (*C*HMe), 47.6 (*C*H_2C=O), 20.7 (CH*C*H_3), 19.9 (CHI=C*C*H_3), 13.0 (CH=C*C*H_3).

HRMS Calcd. for $C_{15}H_{27}INO_3$ $[M+NH_4]^+$ *m/z* 396.1030. Found: 396.1036.

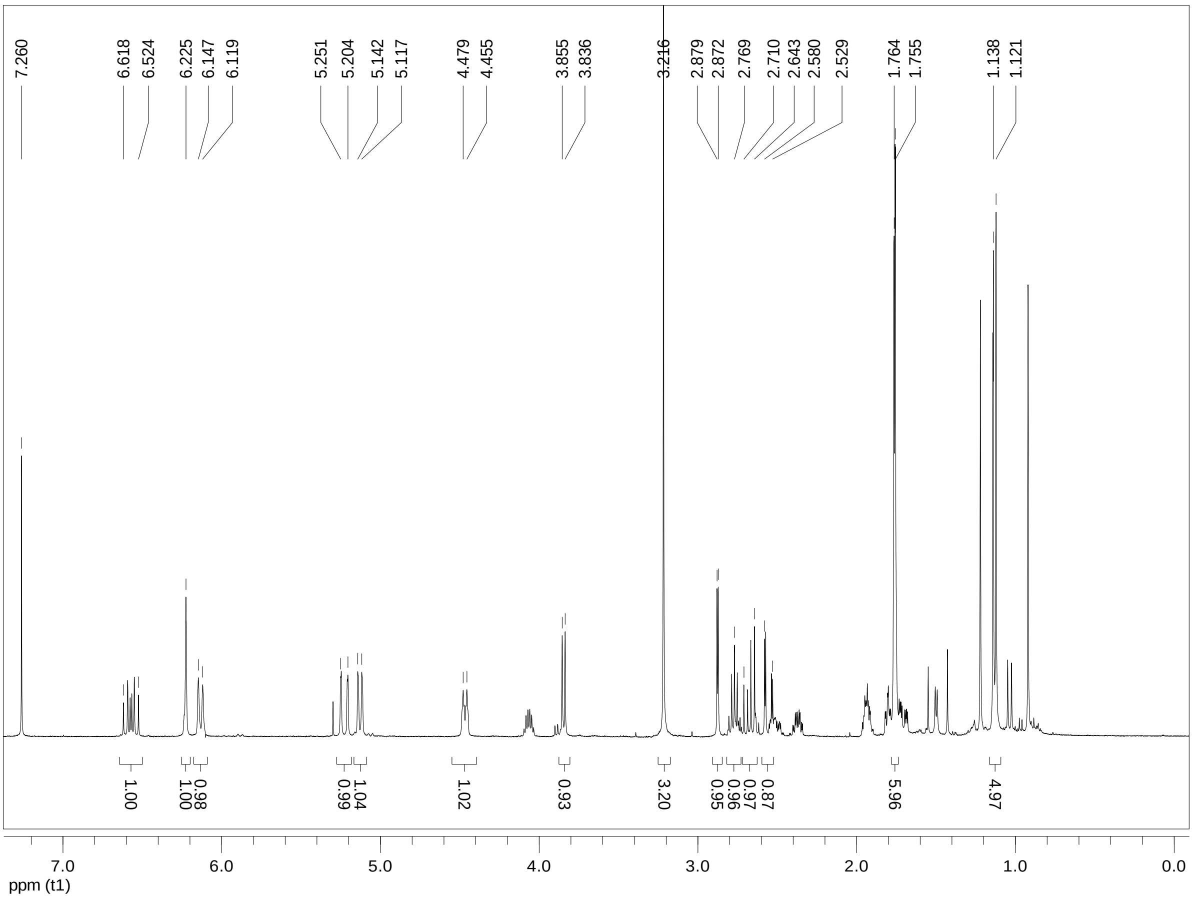
7.260
6.618
6.524
6.225
6.147
6.119
5.251
5.204
5.142
5.117
4.479
4.455
3.855
3.836
3.216
2.879
2.872
2.769
2.710
2.643
2.580
2.529
1.764
1.755
1.138
1.121
1.00
1.00
0.98
0.99
1.04
1.02
0.93
3.20
0.95
0.96
0.97
0.87
5.96
4.97
7.0
6.0
5.0
4.0
3.0
2.0
1.0
0.0
ppm (t1)

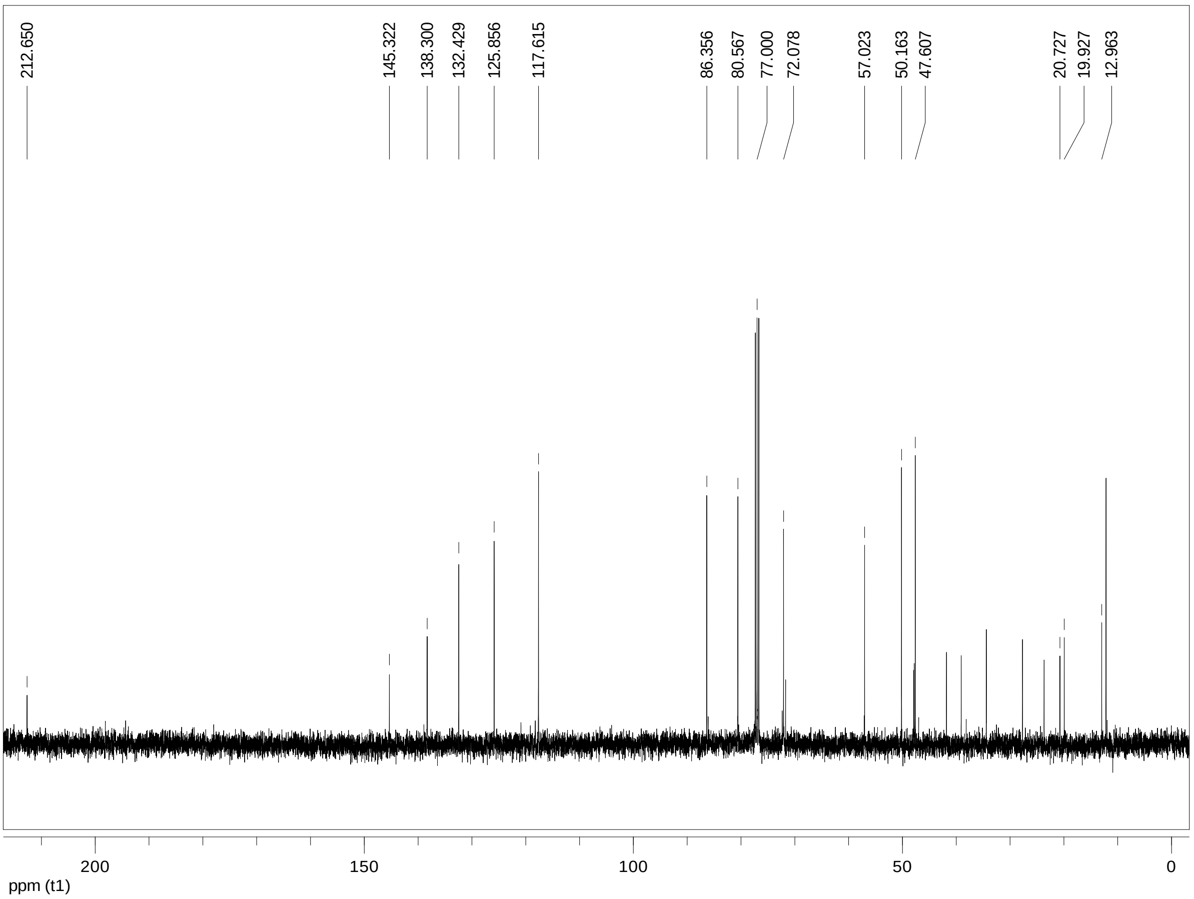
212.650
145.322
138.300
132.429
125.856
117.615
86.356
80.567
77.000
72.078
57.023
50.163
47.607
20.727
19.927
12.963
200
150
100
50
0
ppm (t1)

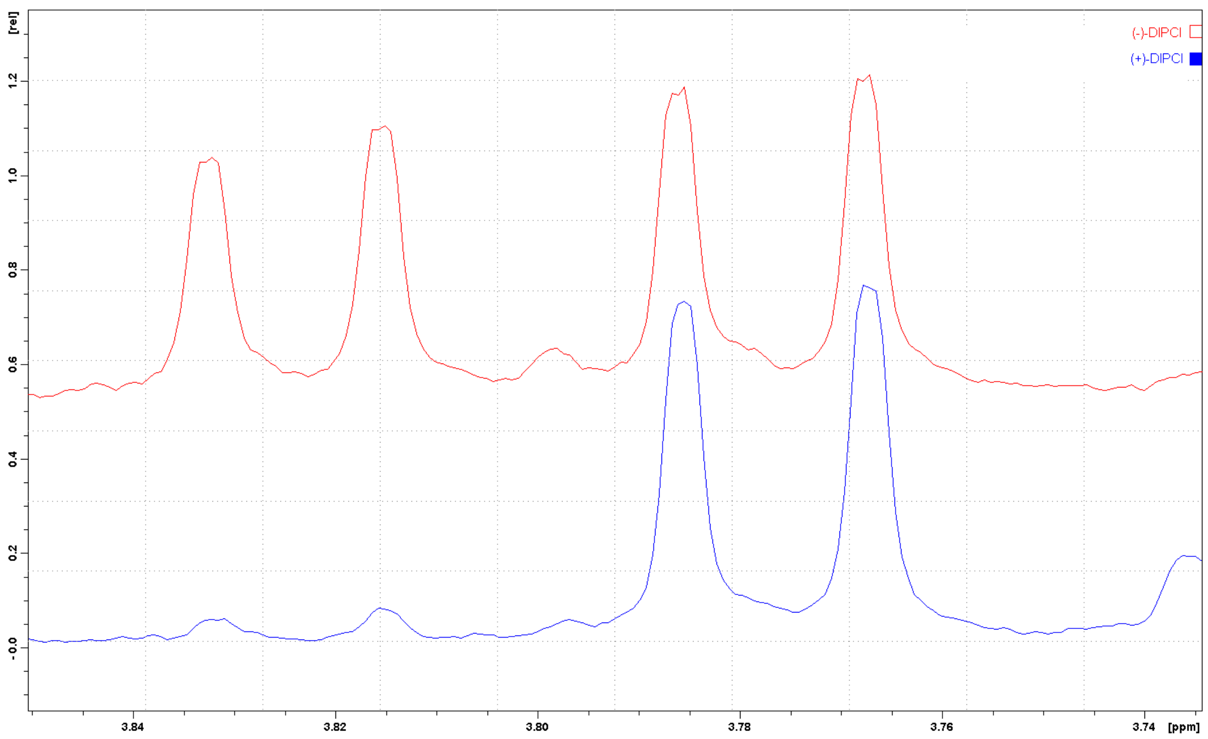
[rel]
1.2
1.0
0.8
0.6
0.4
0.2
-0.0
(-)-DIPCl
(+)-DIPCl
3.84
3.82
3.80
3.78
3.76
3.74
[ppm]

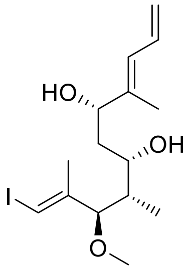

29

(3*E*,5*S*,7*S*,8*S*,9*R*,10*E*)-11-Iodo-9-methoxy-4,8,10-trimethylundeca-1,3,10-triene-5,7-diol (29): A solution of the borohydride (330 mg, 4.65 eq) in MeCN (1.1 mL) and AcOH (1.1 mL) was cooled to −40 °C (MeCN/dry ice). To the frozen mixture was added a solution of the ketone **28** (102 mg, 270 µmol, 1.00 eq) in MeCN (0.3 mL, rinsed with 2×0.3 mL). The resulting thick slurry was stirred for a few minutes at the same temperature and was then aged in the freezer for 19.5 h. Afterwards the suspension was allowed to warm to rt and stirred for another 1 h. Then *Rochelle* salt (aq. sat.) was added to quench the reaction. A white suspension formed immediately. This mixture was stirred at rt for 1.5 h. Then CH_2Cl_2 and $NaHCO_3$ (sat. aq.) were added, the mixture was shaken, and the layers were separated. The aqueous phase was extracted with CH_2Cl_2 (3×10 mL). The combined organic extracts were washed with $NaHCO_3$ (sat. aq.) and with brine, dried over $MgSO_4$ and concentrated under reduced pressure. The crude was purified by column chromatography (hex:Et_2O 2:1→hex:EtOAc 1:1) to afford the desired diol **29** (94.4 mg, 92%, dr = n.d.) as a colourless oil.

TLC (hex:EtOAc 1:1): R_f = 0.58

$[\alpha]_D^{24}$ = +34.9 (*c* = 0.29, $CHCl_3$)

^{1}H-NMR ($CDCl_3$, 400.1 MHz): δ = 6.60 (ddd, *J* = 10.2 Hz, *J* = 10.8 Hz, *J* = 16.8 Hz, 1H, CH_2=C*H*), 6.21-6.16 (bd, *J* = 10.9 Hz, 1H, CH_2=CHC*H*), 6.16-6.14 (m, 1H, C*H*I), 5.23 (dd, *J* = 1.9 Hz, *J* = 16.8 Hz, 1H, CH*H*=CH, *trans*), 5.12 (dd, *J* = 1.6 Hz, *J* = 10.2 Hz, 1H, C*H*H=CH, *cis*), 4.39 (m, 1H, C*H*OHC$_q$Me), 3.84 (bd, *J* = 3.1 Hz, 1H, C*H*OMe), 3.86-3.78 (m, 1H, CHMeC*H*OH), 3.27 (s, 3H, OCH_3), 3.20 (d, *J* = 5.8 Hz, 1H, CHMeCHO*H*), 2.64 (d, *J* = 4.2 Hz, 1H, CHO*H*C$_q$Me), 1.85-1.68 (m, 9H, C*H*Me, CH_2, CH=CCH_3, CHI=CCH_3), 0.85 (d, *J* = 7.1 Hz, 3H, CHCH_3).

^{13}C-NMR ($CDCl_3$, 100.6 MHz): δ = 145.2 (CHI=*C*), 140.3 (CH=*C*), 132.6 (CH_2=*C*H), 125.0 (CH_2=CH*C*H), 117.2 (H_2*C*=CH), 86.6 (*C*HOMe), 78.2 (*C*HI), 74.1 (*C*HOHCMe), 71.4

(*C*HOHCHMe), 57.3 (O*C*H_3), 40.7 (*C*HMe), 38.6 (*C*H_2), 21.8 (CHI=C*C*H_3), 13.3 (CH=C*C*H_3), 11.0 (CH*C*H_3).

IR (ν/[cm^{-1}]) 3408 (br), 2978, 2934, 2830, 1720, 1671, 1620, 1446, 1380, 1364, 1260, 1247, 1216, 1193, 1113, 1088, 1052, 1012, 989, 927, 907, 751 (s), 683, 665, 601, 507, 496, 484, 470, 458.

HRMS Calcd. for $C_{15}H_{25}INaO_3$ $[M+Na]^+$ *m/z* 403.0741. Found: 403.0728.

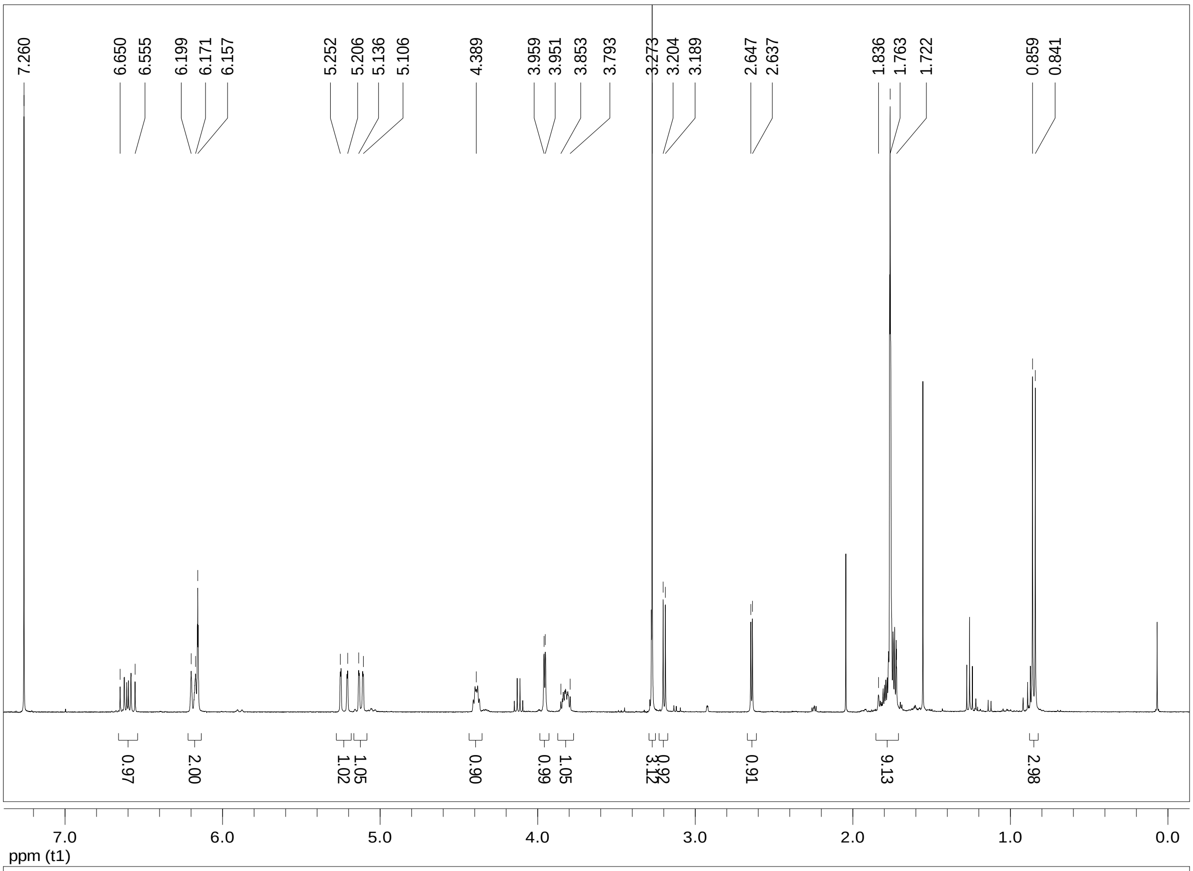
7.260
6.650
6.555
6.199
6.171
6.157
5.252
5.206
5.136
5.106
4.389
3.959
3.951
3.853
3.793
3.273
3.204
3.189
2.647
2.637
1.836
1.763
1.722
0.859
0.841
0.97
2.00
1.02
1.05
0.90
0.99
1.05
3.12
0.92
0.91
9.13
2.98
7.0
6.0
5.0
4.0
3.0
2.0
1.0
0.0
ppm (t1)

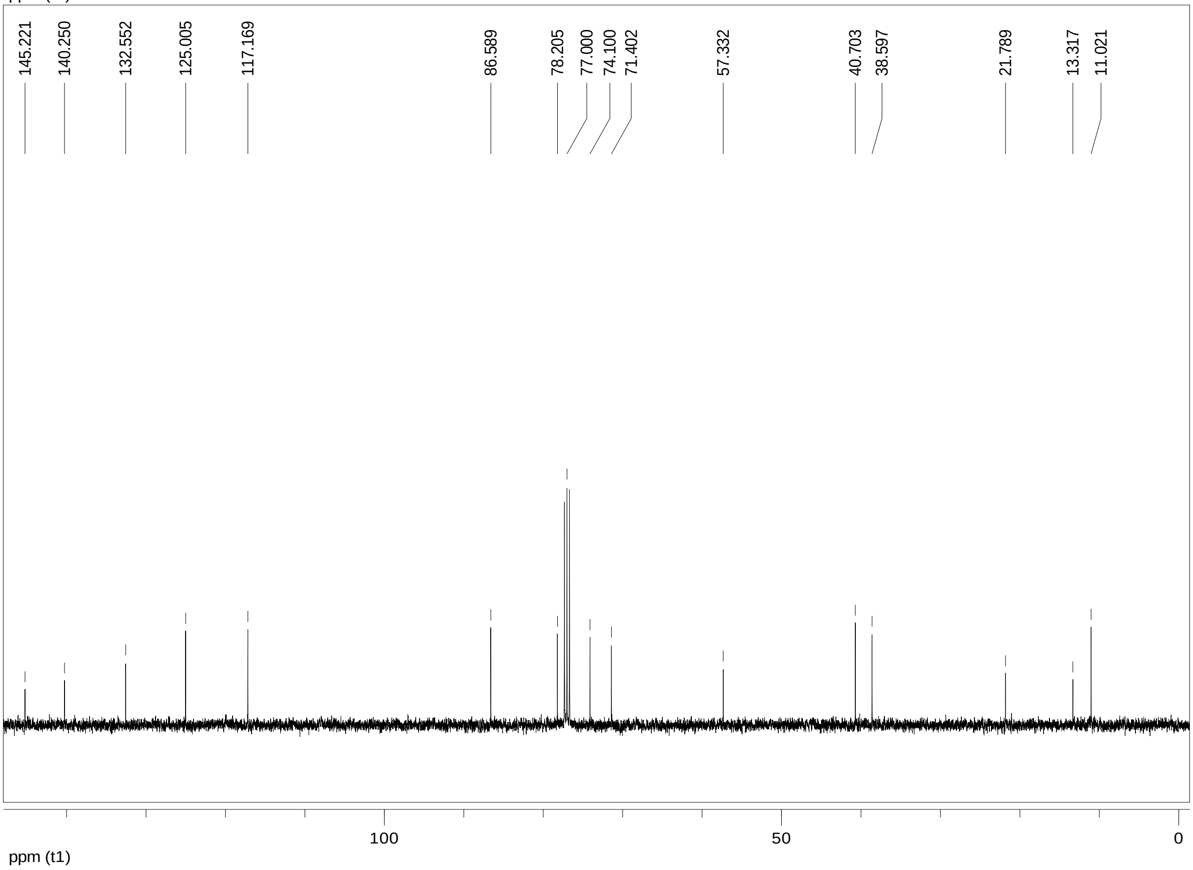
145.221
140.250
132.552
125.005
117.169
86.589
78.205
77.000
74.100
71.402
57.332
40.703
38.597
21.789
13.317
11.021
100
50
0
ppm (t1)

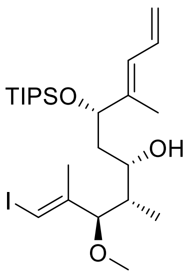

30

(1*E*,3*R*,4*S*,5*S*,7*S*,8*E*)-1-Iodo-3-methoxy-2,4,8-trimethyl-7-((triisopropylsilyl)oxy)-undeca-1,8,10-trien-5-ol (30): To a solution of the diol **29** (85.5 mg, 0.225 mmol, 1.00 eq) in CH_2Cl_2 (6 mL) over molecular sieves (3 Å beads) was added 2,6-lutidine (131 µL, 1.12 mmol, 5.00 eq) at rt. The mixture was stirred at rt for 5 min, then it was cooled to −78 °C and, after another 5 min stirring, TIPSOTf (60.0 µL, 0.225 mmol, 1.00 eq) was added. TLC after 10 min and after 25 min showed incomplete conversion. Therefore, more TIPSOTf (5 µL) was added after 32 min. After 40 min the reaction was complete and was thus quenched with $NaHCO_3$ (sat. aq.). The layers were separated and the aqueous phase was extracted with CH_2Cl_2 (3×10 mL). Then the combined organic extracts were washed with brine, dried over $MgSO_4$ and the solvent was removed under reduced pressure. The crude was purified by column chromatography (hex:Et_2O 7:1) to afford the desired product **30** as a colourless oil (109 mg, 91%), which turned solid in the freezer.

TLC (Hex:EtOAc 5:1): R_f = 0.57

$[\alpha]_D^{24}$ = +25.1 (*c* = 0.32, $CHCl_3$)

^{1}H-NMR ($CDCl_3$, 400.1 MHz): δ = 6.60 (ddd, *J* = 10.2 Hz, *J* = 10.9 Hz, *J* = 16.8 Hz, 1H), 6.24-6.18 (bd, *J* = 11.0 Hz, 1H), 6.08 (m_c, 1H), 5.19 (dd, *J* = 1.8 Hz, *J* = 16.8 Hz, 1H), 5.10 (dd, *J* = 1.7 Hz, *J* = 10.1 Hz, 1H), 4.50 (t, *J* = 4.3 Hz, 1H), 3.78-3.62 (m, 2H), 3.58 (d, *J* = 2.4 Hz, 1H), 3.22 (s, 3H), 1.77-1.66 (m, 3H), 1.73 (d, *J* = 2.4 Hz, 3H), 1.72 (d, *J* = 0.8 Hz, 3H), 1.14-1.01 (m, 21H), 0.84 (d, *J* = 7.0 Hz, 3H).

^{13}C-NMR ($CDCl_3$, 100.6 MHz): δ = 146.2, 138.9, 132.5, 125.8, 116.8, 86.5, 77.9, 76.3, 69.7, 57.2, 41.2, 38.0, 21.1, 18.03, 18.00, 13.9, 12.3, 10.1.

IR (ν/[cm^{-1}]) 3511 (br), 2942 (s), 2893, 2866 (s), 2360, 2340, 2333, 1721, 1696, 1653, 1621, 1601, 1463, 1380, 1256, 1194, 1136, 1112, 1089 (s), 1063 (s), 1011, 988 (s), 948, 883 (s), 823, 757, 680 (s), 601, 577, 512, 495, 464, 445, 428.

HRMS Calcd. for $C_{24}H_{45}INaO_3Si$ $[M+Na]^+$ *m/z* 559.2075. Found: 559.2069.

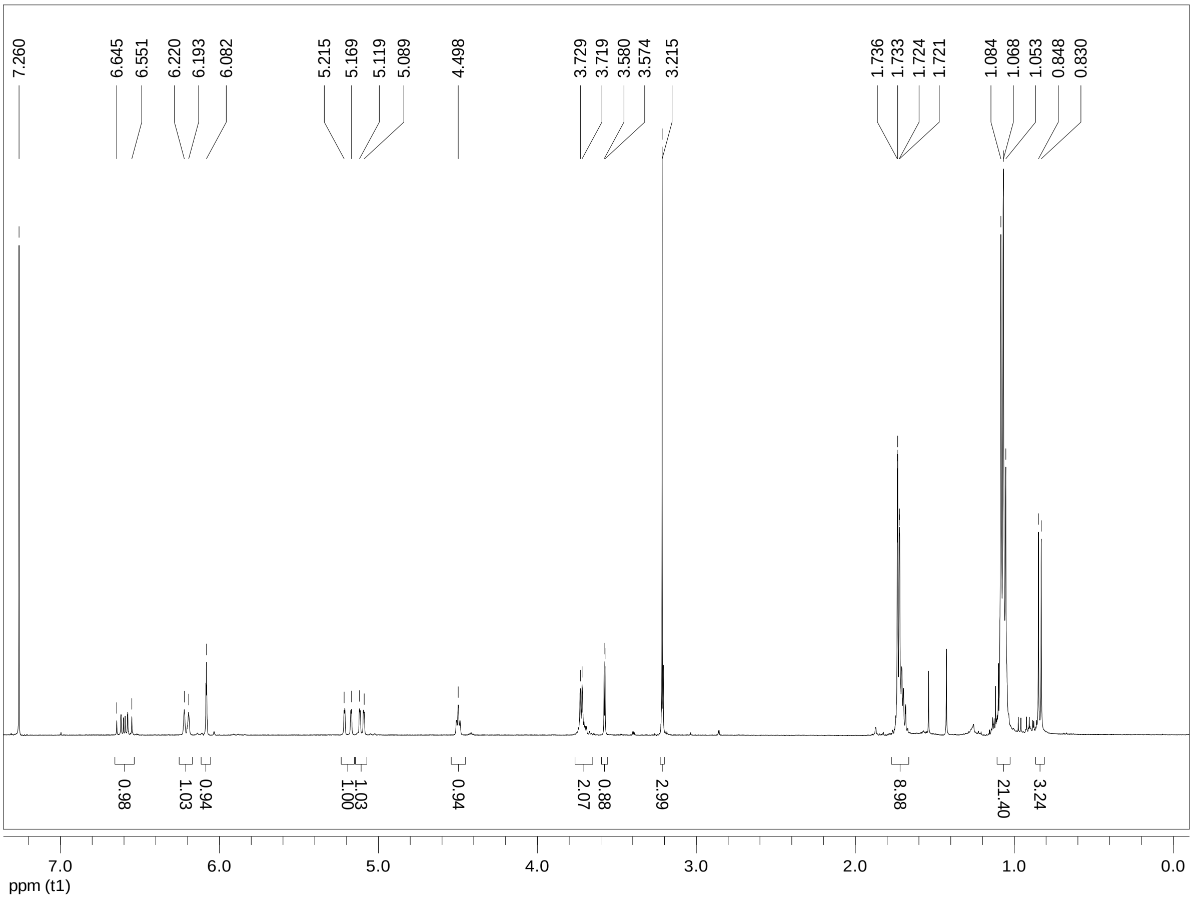
7.260
6.645
6.551
6.220
6.193
6.082
5.215
5.169
5.119
5.089
4.498
3.729
3.719
3.580
3.574
3.215
1.736
1.733
1.724
1.721
1.084
1.068
1.053
0.848
0.830
0.98
1.03
0.94
1.00
1.03
0.94
2.07
0.88
2.99
8.98
21.40
3.24
7.0
6.0
5.0
4.0
3.0
2.0
1.0
0.0
ppm (t1)

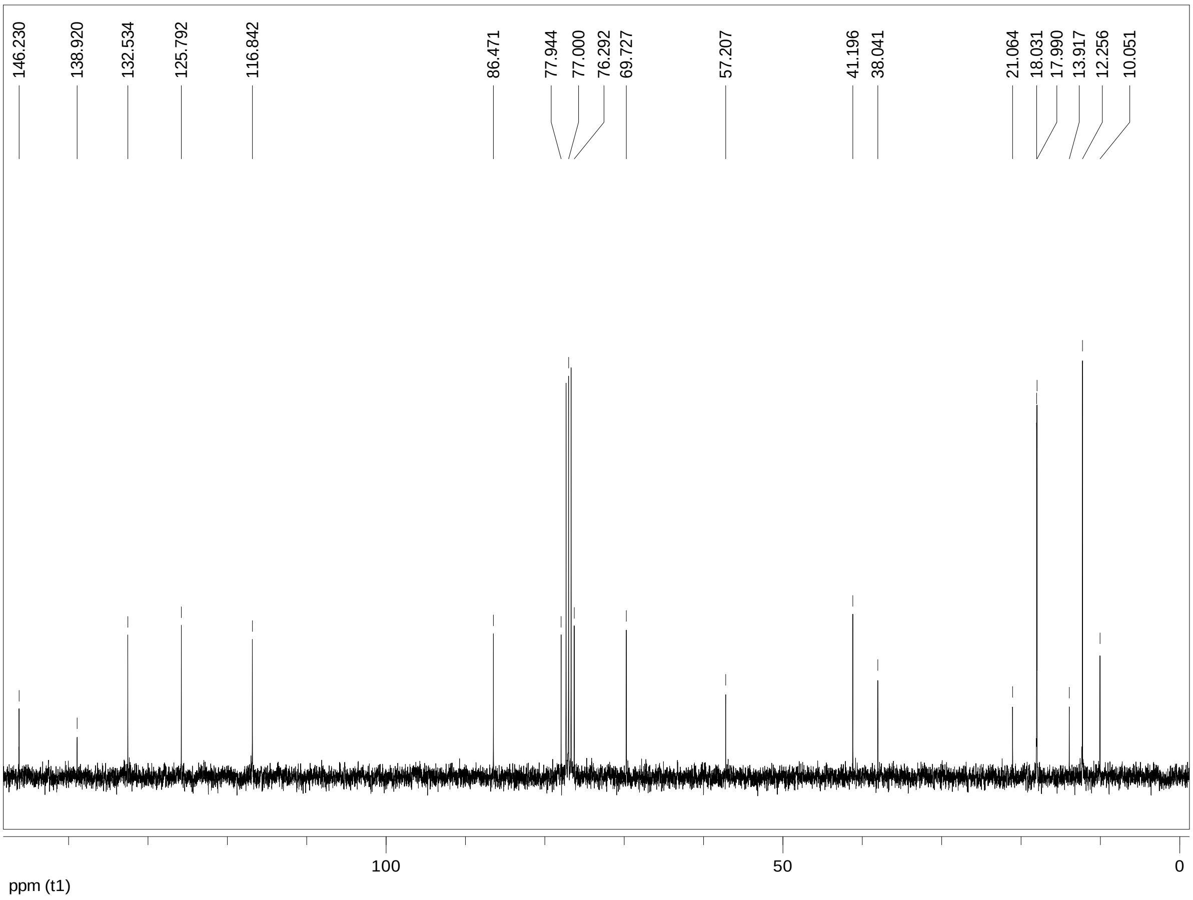
146.230
138.920
132.534
125.792
116.842
86.471
77.944
77.000
76.292
69.727
57.207
41.196
38.041
21.064
18.031
17.990
13.917
12.256
10.051
100
50
0
ppm (t1)

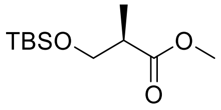

S10a

Methyl (*R*)-3-(*tert*-butyldimethylsilyloxy)-2-methylpropanoate (S10a): To a stirring solution of (*R*)-(−)-3-hydroxyisobutyric acid methyl ester (**36**) (4.00 mL, 36.1 mmol, 1.0 equiv) in CH_2Cl_2 (360 mL) was added at 0 °C imidazole (3.69 g, 54.1 mmol, 1.5 equiv) followed by TBSCl (6.53 g, 43.3 mmol, 1.2 equiv). After stirring for 30 min at 0°C (the reaction was almost complete) and for 2 h at rt, the reaction mixture was quenched at rt with sat. NH_4Cl (70 mL) and water (50 mL). The layers were separated and the aqueous layer was extracted with CH_2Cl_2 (2 x 60 mL). The combined organic extracts were dried over $MgSO_4$ and concentrated under reduced pressure. The residue was purified by column chromatography (hexane → hexane/ Et_2O, 5:1) to give **S10a** (8.33 g, 99%) as a colorless oil. The analytical data were identical to those reported in the literature.[1]

R*f* = 0.68 (hexane/Et_2O, 5:1)

^{1}H-NMR (400.1 MHz, $CDCl_3$): δ = 3.77 (dd, *J* = 6.9, 9.7 Hz, 1H), 3.67 (s, 3H), 3.64 (dd, *J* = 6.1, 9.7 Hz, 1H), 2.64 (m_c, 1H), 1.13 (d, *J* = 7.0 Hz, 3H), 0.87 (s, 9H), 0.03 (s, 6H).

^{13}C-NMR (100.6 MHz, $CDCl_3$): δ = 175.5, 65.2, 51.5, 42.5, 25.8, 18.2, 13.4, −5.5.

IR (neat, ν/cm^{-1}): 2953m, 2930m, 2858m, 1742s, 1462m, 1435w, 1389w, 1254w, 1254m, 1198m, 1175m, 1092s, 835s, 775s, 666w.

HRMS (ESI): *m/z* calcd for $C_{11}H_{24}NaO_3Si$ [M]$^+$: 255.1387, found: 255.1392.

[1] Keck, G. E.; Giles, R. L.; Cee, V. J., Wager, C. A.; Yu, T.; Kraft, M. B. *J. Org. Chem.* **2008**, *73*, 9675–9691.

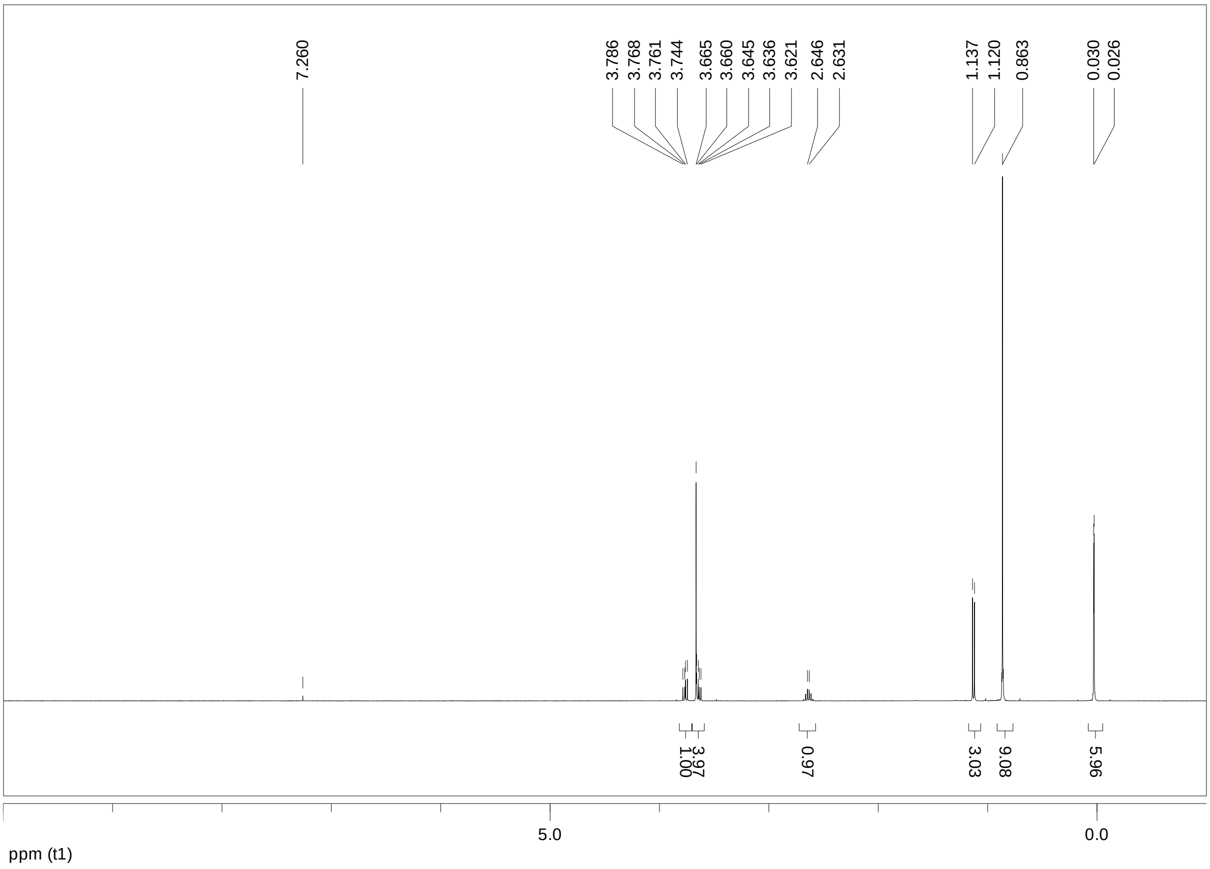
7.260
3.786
3.768
3.761
3.744
3.665
3.660
3.645
3.636
3.621
2.646
2.631
1.137
1.120
0.863
0.030
0.026
1.00
3.97
0.97
3.03
9.08
5.96
5.0
0.0
ppm (t1)

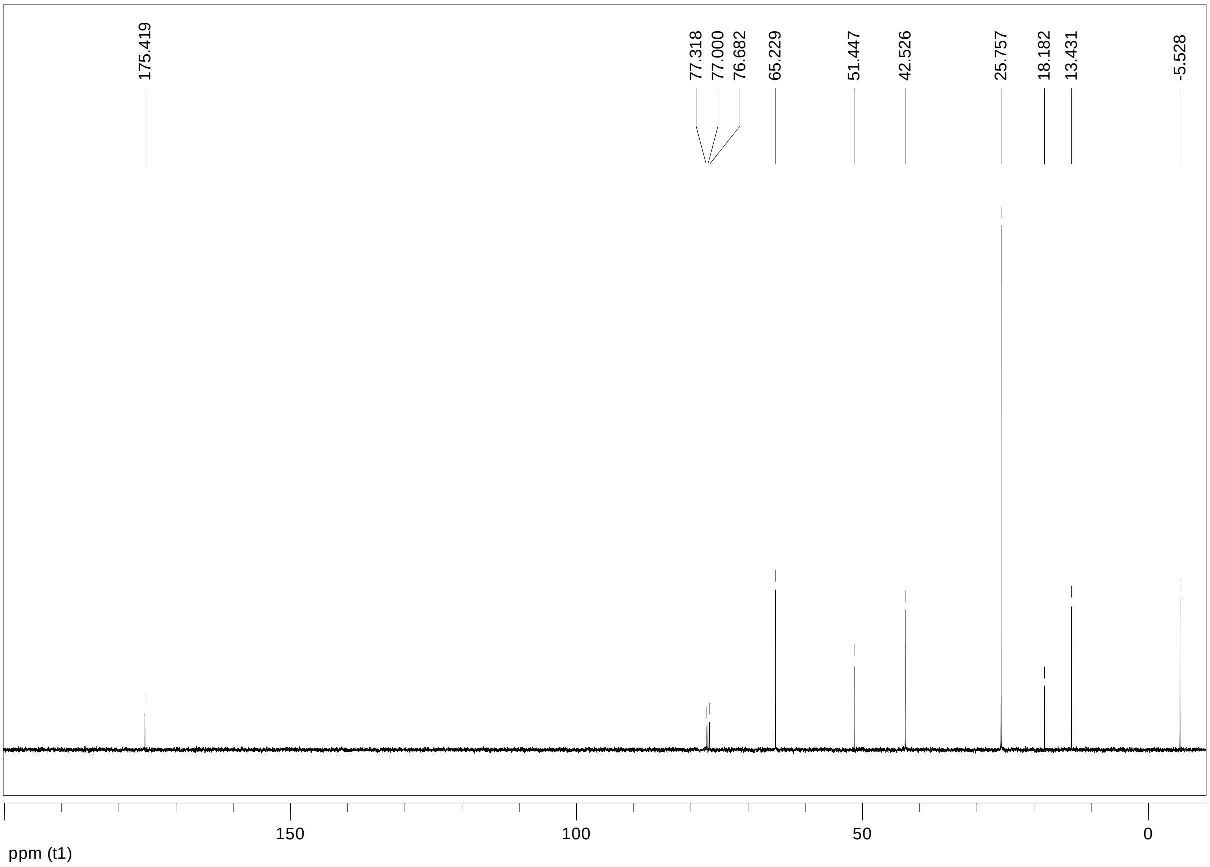
175.419
77.318
77.000
76.682
65.229
51.447
42.526
25.757
18.182
13.431
-5.528
150
100
50
0
ppm (t1)

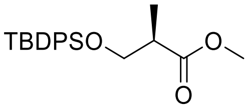

S10b

Methyl (*R*)-3-((*tert*-butyldiphenylsilyl)oxy)-2-methylpropanoate (S10b): To a stirring solution of (*R*)-(−)-3-hydroxyisobutyric acid methyl ester (**36**) (350 mg, 2.96 mmol, 1.0 equiv) in DMF (3 mL) was added at rt imidazole (303 mg, 4.45 mmol, 1.5 equiv) followed by TBDPSCl (0.92 mL, 3.55 mmol, 1.2 equiv) in one portion. After stirring for 18 h at rt, the reaction mixture was diluted with Et_2O (60 mL) and water (15 mL) and the layers were separated. The aqueous layer was extracted once with Et_2O (30 mL). The combined organic extracts were washed with brine (20 mL), dried over $MgSO_4$ and concentrated under reduced pressure. The residue was purified by column chromatography (hexane/EtOAc, 200:1 → 100:1, then hexane/Et_2O, 5:1) to give **S10b** (971 mg, 92%) as a colorless oil.[2]

R*f* = 0.62 (hexane/Et_2O, 5:1)
^{1}H-NMR (400.1 MHz, $CDCl_3$): δ = 7.72-7.62 (m, 4H), 7.47-7.35 (m, 6H), 3.84 (dd, *J* = 6.9, 9.7 Hz, 1H), 3.74 (dd, *J* = 5.8, 9.8 Hz, 1H), 3.69 (s, 3H), 2.73 (m_c, 1H), 1.17 (d, *J* = 7.0 Hz, 3H), 1.05 (s, 9H).

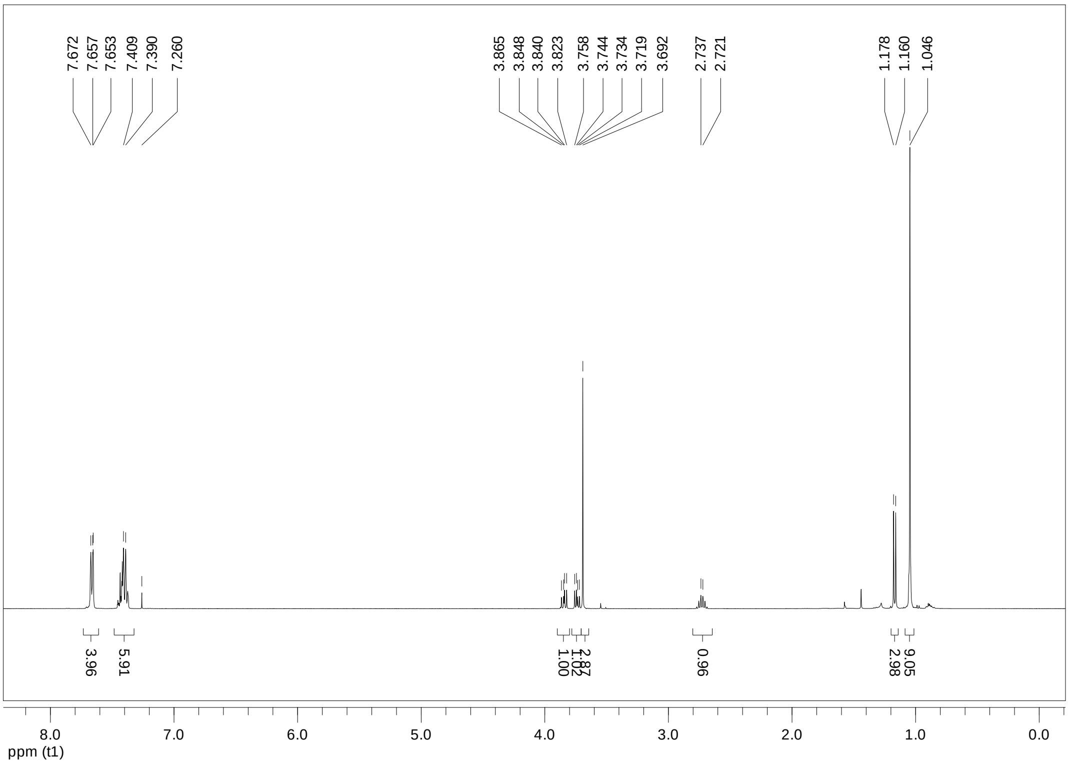

---

[2] The analytical data were identical to those reported in the literature: Fürstner A.; Kattnig, E.; Lepage, O. *J. Am. Chem. Soc.* **2006**, *128*, 9194-9204.

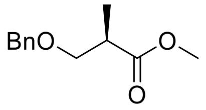

S10c

Methyl (*R*)-3-(benzyloxy)-2-methylpropanoate (S10c): To a solution of (*R*)-(−)-3-hydroxyisobutyric acid methyl ester (**36**) (318 mg, 2.69 mmol, 1.0 equiv) in CH_2Cl_2/cyclohexane (1:1, 9.1 mL) was added benzyl trichloroacetimidate (0.60 mL, 3.23 mmol, 1.2 equiv) and a few drops of TfOH (until the reaction began and the imidate salt started to precipitate). After stirring for 4 h at rt, the reaction mixture was diluted with sat. $NaHCO_3$ (20 mL) and CH_2Cl_2 (20 mL). The aqueous layer was extracted with CH_2Cl_2 (3 x 30 mL). The combined organic extracts were washed with sat. $NaHCO_3$ and brine, dried over $MgSO_4$, filtered and the solvent was removed under reduced pressure. The residue was purified by column chromatography (hexane/EtOAc, 30:1 → 20:1) to give **S10c** as a colorless oil (430 mg, 77%).[3]

R*f* = 0.60 (hexane/EtOAc, 5:1)

^{1}H-NMR (400.1 MHz, $CDCl_3$): δ = 7.39-7.25 (m, 5H), 4.53 (s, 2H), 3.70 (s, 3H), 3.67 (dd, *J* = 7.3, 9.1 Hz, 1H), 3.50 (dd, *J* = 5.9, 9.1 Hz, 1H), 2.80 (m_c, 1H), 1.19 (d, *J* = 7.1 Hz, 3H).

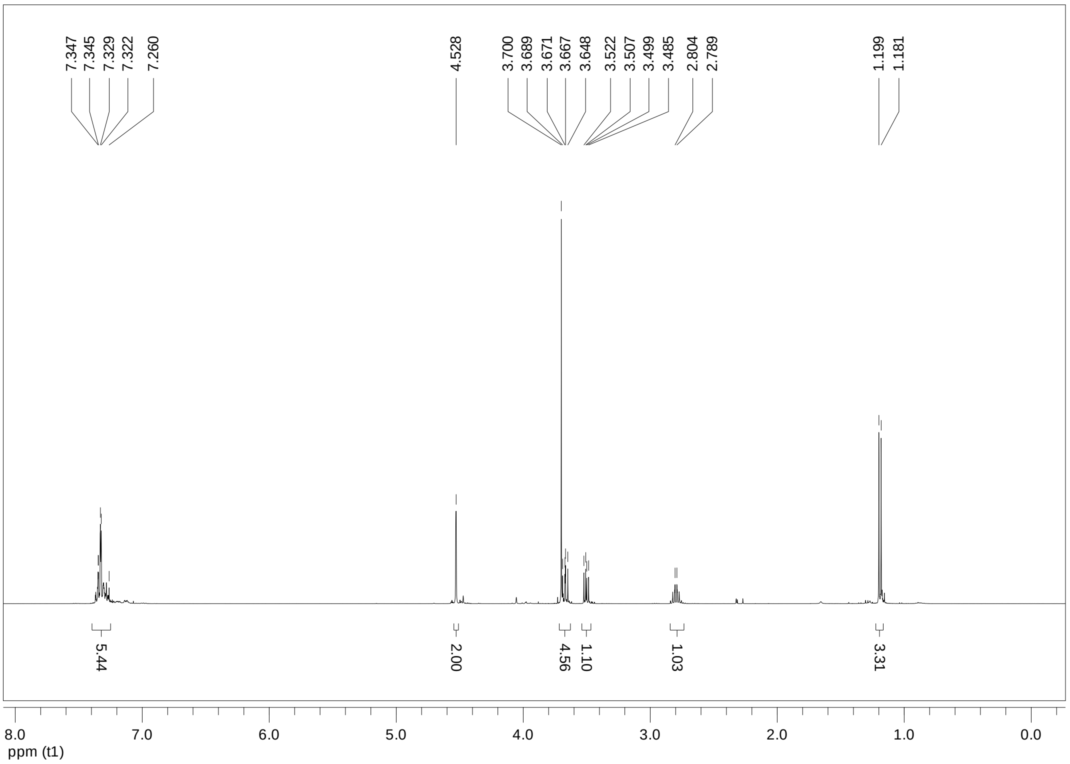

[3] The analytical data were identical to those reported in the literature: Kanada, R. M.; Itoh, D.; Nagai, M.; Nijima, J.; Asai, N.; Yoshiharu, M.; Abe, S.; Kotake, Y. *Angew. Chem. Int. Ed.* **2007**, *46*, 4350-4355.

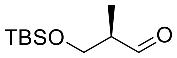

11a

(*R*)-3-(*tert*-butyldimethylsilyloxy)-2-methylpropanal (11a): To a stirring solution of **S10a** (4.30 g, 18.5 mmol, 1.0 equiv) in CH_2Cl_2 (185 mL) was added dropwise at −78 °C DIBAL (19.0 mL, 19.0 mmol, 1.0 equiv, 1.0 M in CH_2Cl_2) with the aid of a mechanical syringe pump over 1.25 h. After the addition was complete, the reaction mixture was stirred for 1.25 h at −78 °C. The reaction mixture was quenched by addition of sat. *Rochelle* salt (100 mL) and stirred for 30 min at rt. The suspension was filtered over celite, the layers were separated and the aqueous layer was extracted with CH_2Cl_2 (2 x 70 mL). The combined organic extracts were dried over $MgSO_4$ and concentrated under reduced pressure. The crude product **11a** was directly used for the next step without further purification due to danger of racemization. The analytical data were identical to those reported in the literature.[4]

R_f = 0.60 (hexane/EtOAc, 10:1)

^{1}H-NMR (400.1 MHz, $CDCl_3$): δ = 9.74 (d, *J* = 1.7 Hz, 1H), 3.86 (dd, *J* = 5.2, 10.2 Hz, 1H), 3.81 (dd, *J* = 6.3, 10.2 Hz, 1H), 2.53 (m_c, 1H), 1.09 (d, *J* = 7.0 Hz, 3H), 0.88 (s, 9H), 0.05 (s, 6H).

^{13}C-NMR (100.6 MHz, $CDCl_3$): δ = 204.6, 63.4, 48.8, 25.8, 18.2, 10.3, −5.5.

[4] Keck, G. E.; Giles, R. L.; Cee, V. J., Wager, C. A.; Yu, T.; Kraft, M. B. *J. Org. Chem.* **2008**, *73*, 9675–9691.

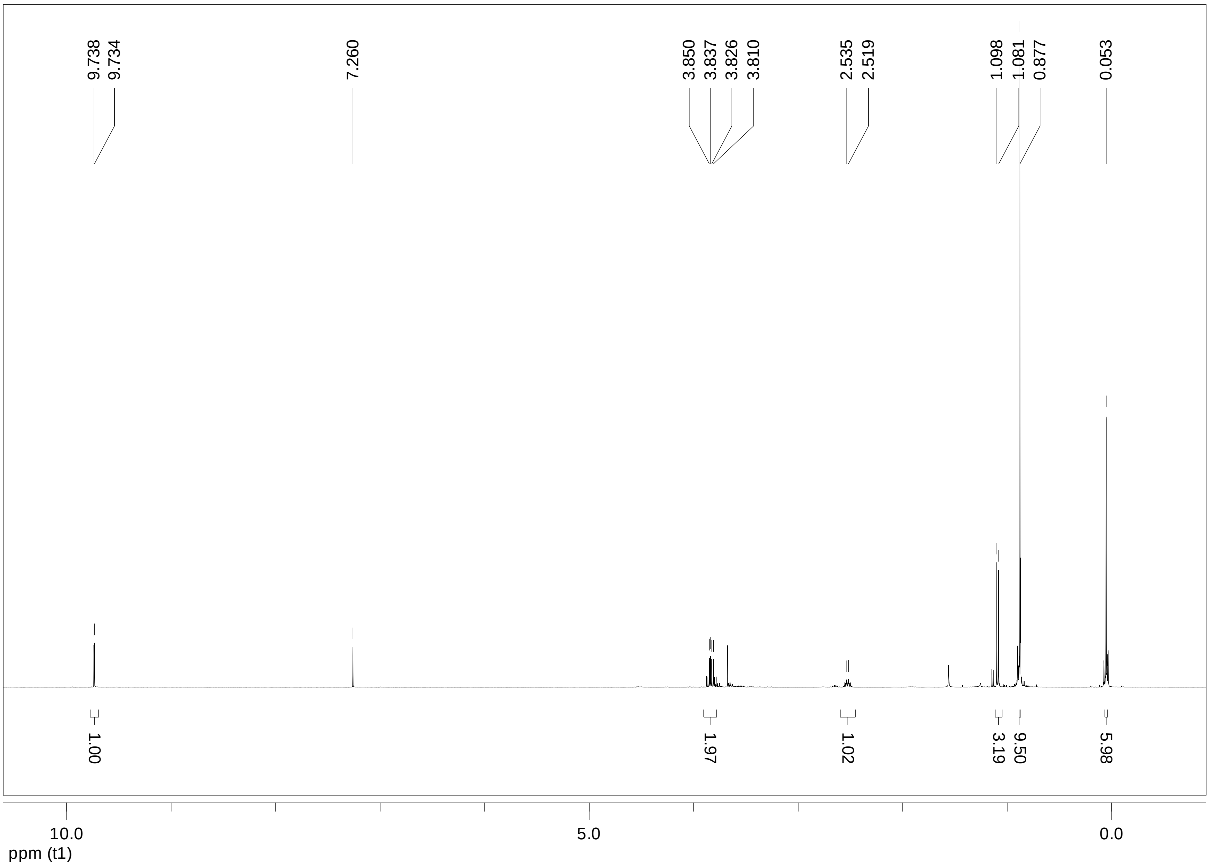
9.738
9.734
7.260
3.850
3.837
3.826
3.810
2.535
2.519
1.098
1.081
0.877
0.053
1.00
1.97
1.02
3.19
9.50
5.98
10.0
5.0
0.0
ppm (t1)

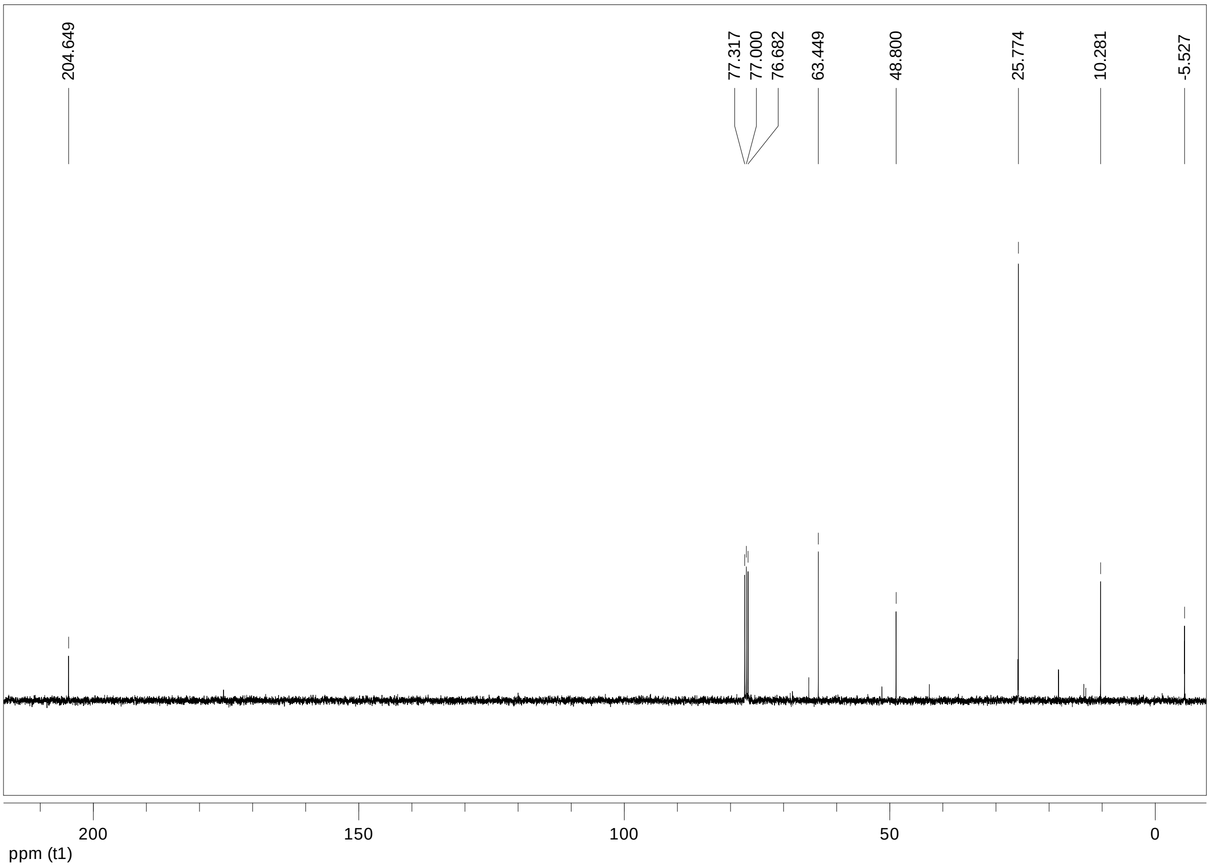
204.649
77.317
77.000
76.682
63.449
48.800
25.774
10.281
-5.527
200
150
100
50
0
ppm (t1)

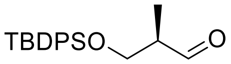

11b

(*R*)-3-((*tert*-Butyldiphenylsilyl)oxy)-2-methylpropanal (11a): To a stirring solution of **S10b** (356 mg, 1.00 mmol, 1.0 equiv) in CH_2Cl_2 (10 mL) was added dropwise at −78 °C DIBAL (1.00 mL, 1.00 mmol, 1.0 equiv, 1.0 M in CH_2Cl_2) over 20 min. After the addition was complete, the reaction mixture was stirred for 1.5 h at −78 °C. The reaction mixture was quenched by addition of sat. *Rochelle* salt (15 mL) and stirred for 1 h at rt. The suspension was then diluted with dichloromethane, the layers were separated and the aqueous layer was extracted with CH_2Cl_2 (3 x 40 mL). The combined organic extracts were washed with brine, dried over $MgSO_4$ and concentrated under reduced pressure. The crude product **11b** was filtered over celite (eluting with hexane), concentrated and was directly used for the next step without further purification due to danger of racemization.[5]

R_f = 0.60 (hexane/Et_2O, 5:1)
^{1}H-NMR (400.1 MHz, $CDCl_3$): δ = 9.77 (d, *J* = 1.6 Hz, 1H), 7.74-7.56 (m, 4H), 7.48-7.33 (m, 6H), 3.91 (dd, *J* = 5.0, 10.3 Hz, 1H), 3.85 (dd, *J* = 6.3, 10.3 Hz, 1H), 2.57 (m_c, 1H), 1.11 (d, *J* = 7.0 Hz, 3H), 1.05 (s, 9H).

[5] The analytical data were identical to those reported in the literature: Fürstner A.; Kattnig, E.; Lepage, O. *J. Am. Chem. Soc.* **2006**, *128*, 9194-9204.

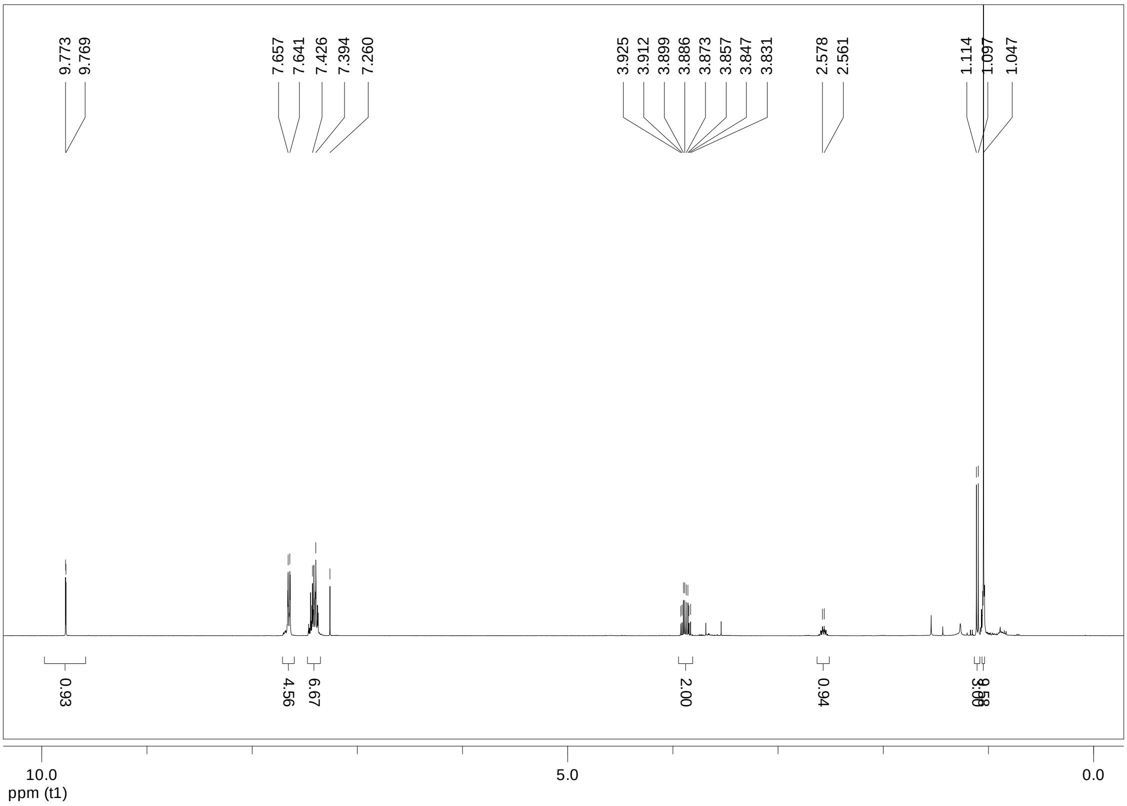
9.773
9.769
7.657
7.641
7.426
7.394
7.260
3.925
3.912
3.899
3.886
3.873
3.857
3.847
3.831
2.578
2.561
1.114
1.097
1.047
0.93
4.56
6.67
2.00
0.94
10.0
5.0
0.0
ppm (t1)

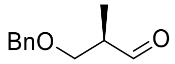

11c

(*R*)-3-(Benzyloxy)-2-methylpropanal (11c): To a stirring solution of **S10c** (61.2 mg, 0.29 mmol, 1.0 equiv) in CH_2Cl_2 (2.8 mL) was added dropwise at −78 °C DIBAL (0.38 mL, 0.38 mmol, 1.3 equiv, 1.0 M in CH_2Cl_2) over 30 min. After the addition was complete, the reaction mixture was stirred for 1 h at −78 °C. The reaction mixture was quenched by addition of sat. *Rochelle* salt (10 mL) and stirred for 1 h at rt. The suspension was then diluted with CH_2Cl_2, the layers were separated and the aqueous layer was extracted with CH_2Cl_2 (3 x 20 mL). The combined organic extracts were washed with brine, dried over $MgSO_4$ and concentrated under reduced pressure. The crude product **1c** was used for the next step without further purification due to danger of racemization.[6]

R*f* = 0.42 (hexane/EtOAc, 5:1)

^{1}H-NMR (400.1 MHz, $CDCl_3$): δ = 9.73 (d, *J* = 1.5 Hz, 1H), 7.39-7.26 (m, 5H), 4.53 (s, 2H), 3.69 (dd, *J* = 6.3, 9.8 Hz, 1H), 3.65 (dd, *J* = 3.9, 9.2 Hz, 1H), 2.67 (m_c, 1H), 1.14 (d, *J* = 7.1 Hz, 3H).

[6] The analytical data were identical to those reported in the literature: Paquette, L. A.; Guevel, R.; Sakamoto, S.; Kim, I. H.; Crawford, J. *J. Org. Chem.* **2003**, *68*, 6096-6107.

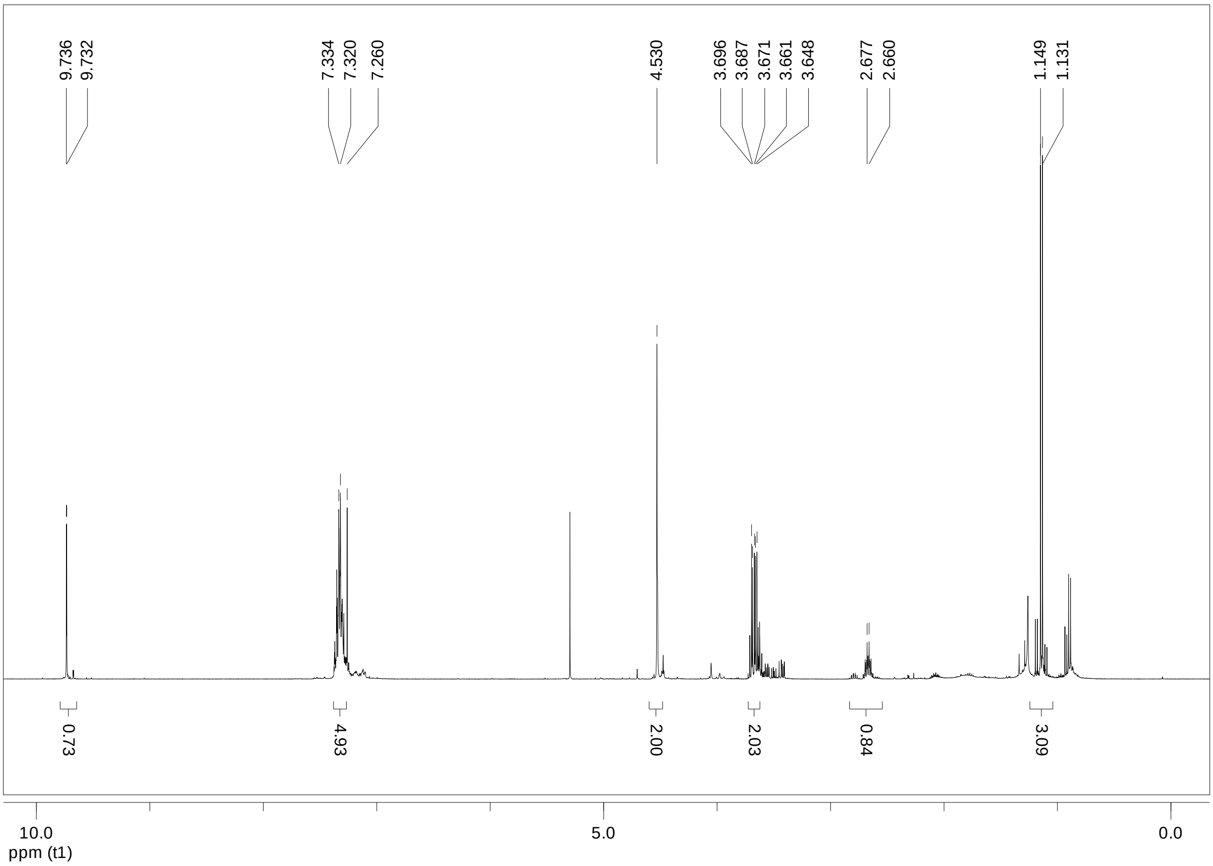

9.736
9.732
7.334
7.320
7.260
4.530
3.696
3.687
3.671
3.661
3.648
2.677
2.660
1.149
1.131
0.73
4.93
2.00
2.03
0.84
3.09
10.0
5.0
0.0
ppm (t1)

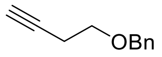

31

((But-3-yn-1-yloxy)methyl)benzene (31): A solution of 3-butyn-1-ol (2.0 mL, 26 mmol, 1.0 equiv) in THF (16 mL) was added dropwise to a suspension of NaH (1.3 g, 54 mmol, 2.1 equiv) in THF (28 mL) at 0 °C. After the adding TBAI (50 mg, 0.1 mmol, 0.5 mol%) and benzylbromide (3.5 mL, 29 mmol, 1.1 equiv) to the reaction mixture, stirring was continued for 1.5 h at 0 °C and overnight at rt. The reaction mixture was quenched at 0 °C with crushed ice until a clear, biphasic solution was formed. The layers were separated and the aqueous phase was extracted with EtOAc (2 x 60 mL). The combined organic extracts were washed with water and brine, dried over $MgSO_4$ and concentrated under reduced pressure. The residue was purified by column chromatography (hexane/EtOAc, 90:1 → 80:1 → 60:1, then hexane/Et_2O, 10:1) to provide **31** (4.22 g, quant) as a yellowish oil.[7]

R_f = 0.57 (hexane/Et_2O, 10:1)
^{1}H-NMR (400.1 MHz, $CDCl_3$): δ = 7.44-7.27 (m, 5H), 4.59 (s, 2H), 3.63 (t, J = 6.9 Hz, 2H), 3.63 (t, J = 6.9 Hz, 2H), 2.53 (dt, J = 2.7, 7.0 Hz, 2H), 2.02 (t, J = 2.7 Hz, 1H).
^{13}C-NMR (100.6 MHz, $CDCl_3$): δ = 138.0, 128.3, 127.6, 81.2, 72.9, 69.3, 68.1, 19.8.

[7] The analytical data were identical to those reported: Yadav, J. S.; Srihari, P. *Tetrahedron Asymmetry* **2004**, *15*, 81-89.

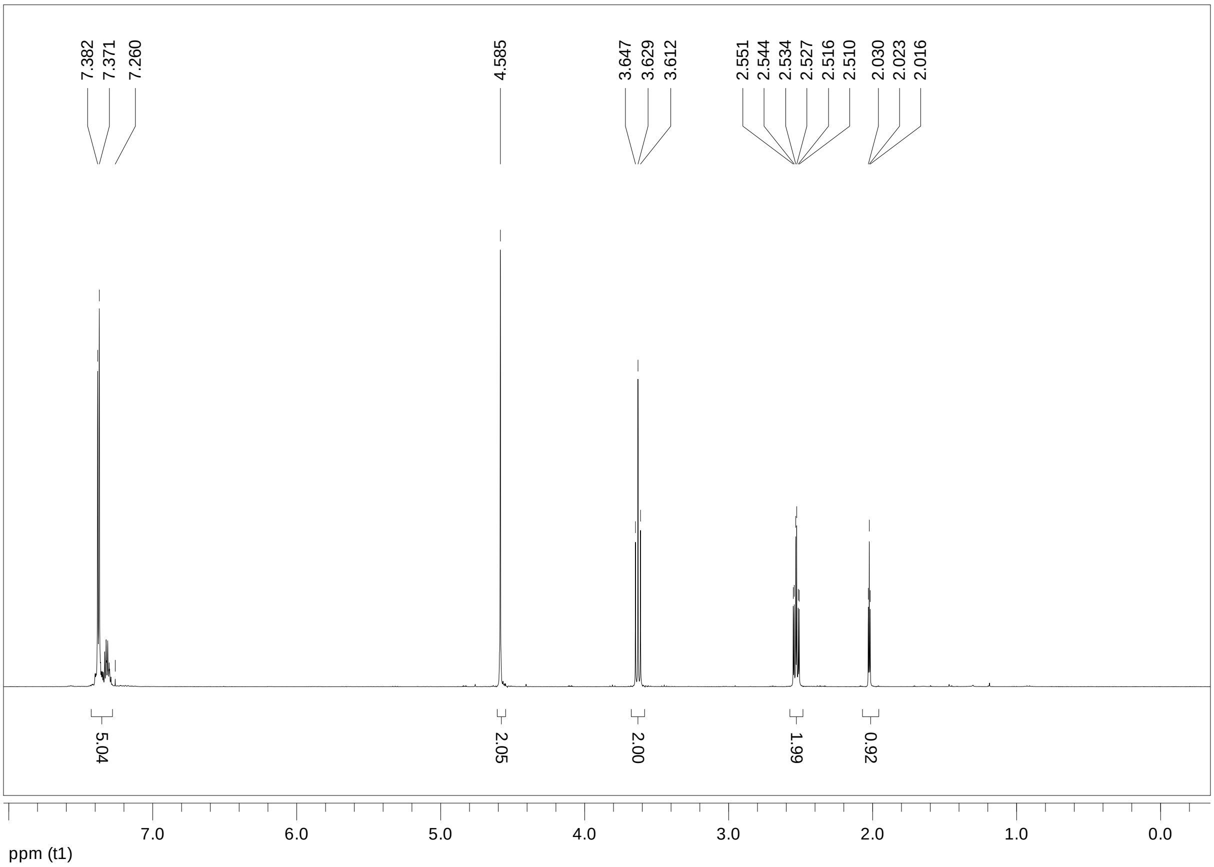
7.382
7.371
7.260
4.585
3.647
3.629
3.612
2.551
2.544
2.534
2.527
2.516
2.510
2.030
2.023
2.016
5.04
2.05
2.00
1.99
0.92
7.0
6.0
5.0
4.0
3.0
2.0
1.0
0.0
ppm (t1)

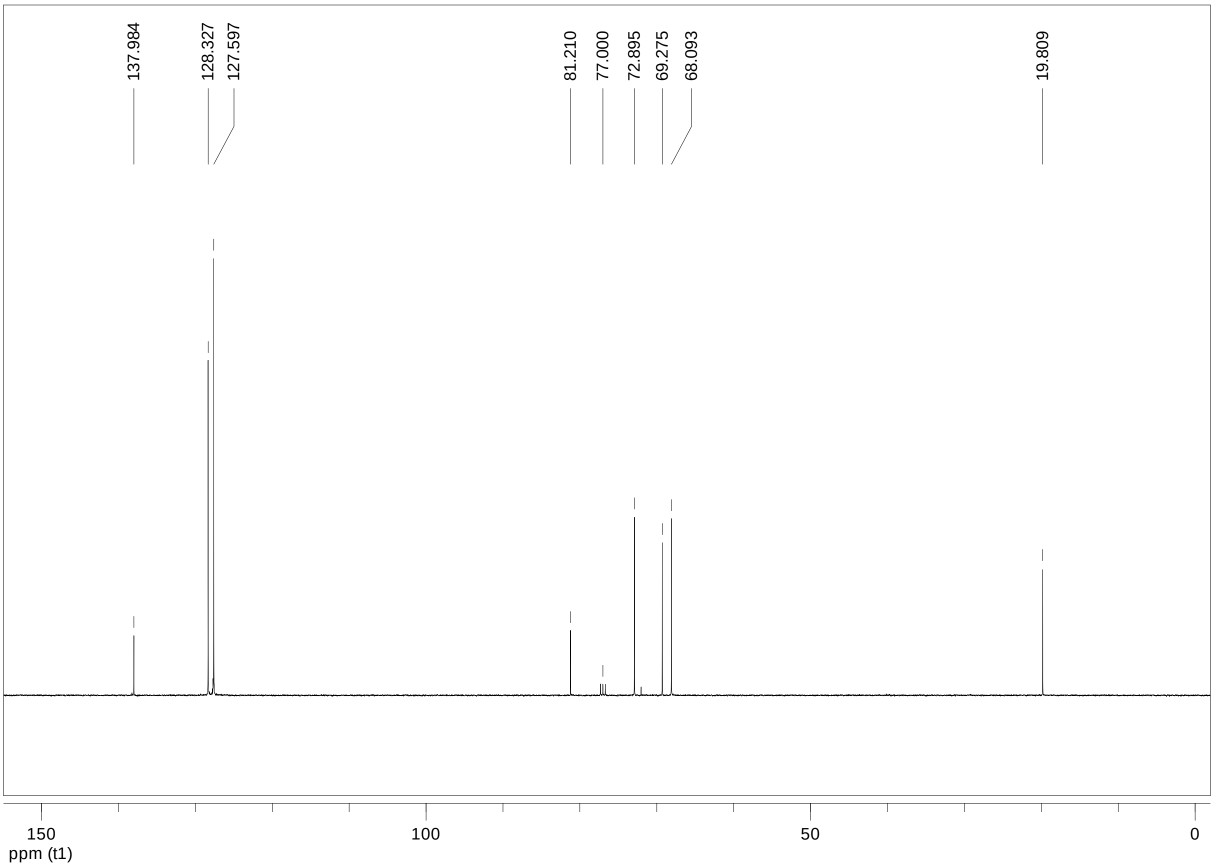
137.984
128.327
127.597
81.210
77.000
72.895
69.275
68.093
19.809
150
100
50
0
ppm (t1)

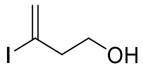

32

3-Iodobut-3-en-1-ol (32): To a suspension of NaI (5.99 g, 40.0 mmol, 2.0 equiv) in acetonitrile (28 mL) was added TMSCl (5.07 mL, 40.0 mmol, 2.0 equiv) followed by water (0.36 mL, 20.0 mmol, 1.0 equiv). After 10 min, a solution of **32** (3.20 g, 20.0 mmol, 1.0 equiv) in acetonitrile (7 mL) was added to the suspension and the reaction mixture was stirred for 1 h at rt. The reaction was quenched with water (70 mL) and extracted with Et_2O (3 x 120 mL). The combined organic extracts were washed with brine, dried over $MgSO_4$ and concentrated under reduced pressure. The residue was purified by column chromatography (hexane/Et_2O, 10:1 → 5:1, then hexane/EtOAc, 5:1 → 3:1) to provide iodoalcohol **32** (1.59 g, 40%) as a brown liquid.[8]

$\mathbf{R}_f$ = 0.33 (hexane/EtOAc, 1:1)
^{1}H-NMR (400.1 MHz, $CDCl_3$): δ = 6.14 (m_c, 1H), 5.81 (m_c, 1H), 3.71 (t, *J* = 6.0 Hz, 2H), 2.60 (dt, *J* = 1.0, 6.0 Hz, 2H), 2.30 (brs, 1H).
^{13}C-NMR (100.6 MHz, $CDCl_3$): δ = 128.4, 107.4, 60.9, 48.0.

[8] Procedure adapted from Sugiyama, H.; Yokokawa, F.; Shioiri, T. *Tetrahedron* **2003**, *59*, 6579-6593.

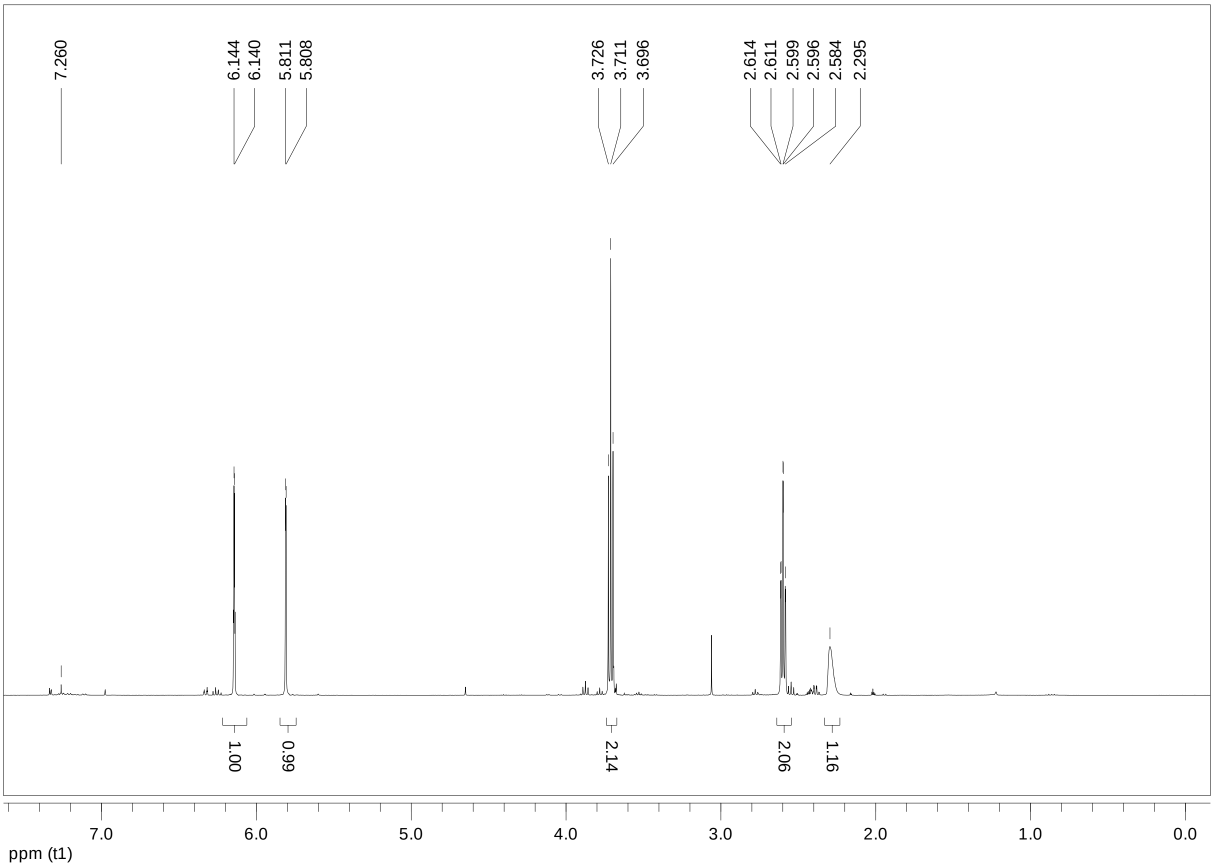
7.260
6.144
6.140
5.811
5.808
3.726
3.711
3.696
2.614
2.611
2.599
2.596
2.584
2.295
1.00
0.99
2.14
2.06
1.16
7.0
6.0
5.0
4.0
3.0
2.0
1.0
0.0
ppm (t1)

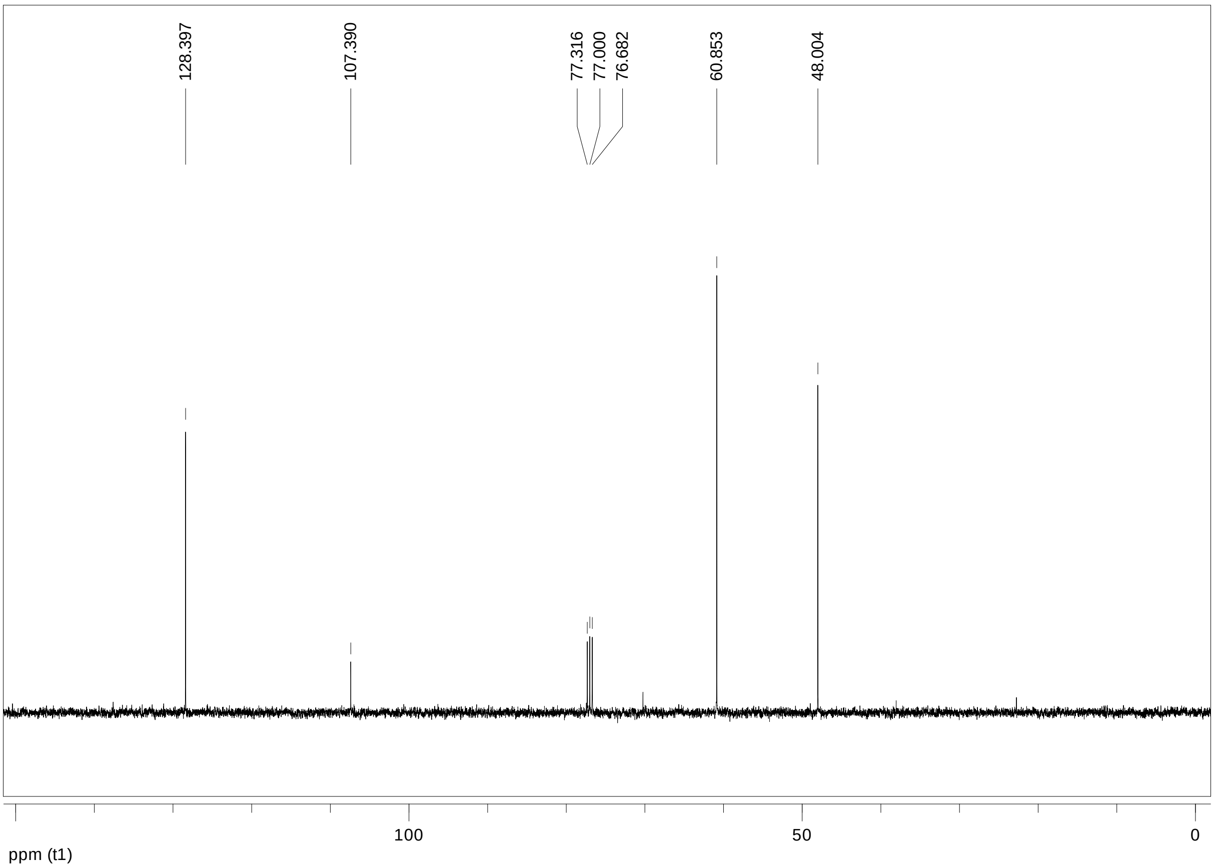
128.397
107.390
77.316
77.000
76.682
60.853
48.004
100
50
0
ppm (t1)

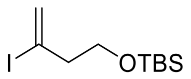

33

***tert*-Butyl((3-iodobut-3-en-1-yl)oxy)dimethylsilane (33):** To a stirring solution of **32** (652 mg, 3.29 mmol, 1.0 equiv) in DMF (5 mL) was added at rt imidazole (336 mg, 4.94 mmol, 1.5 equiv) followed by TBSCl (596 mg, 3.03 mmol, 1.2 equiv). After stirring for 1.5 h at rt, anhydrous methanol (0.5 mL) was added to the reaction mixture to facilitate removal of excess TBSCl. After stirring for 30 min at rt, the reaction mixture was diluted with Et_2O (60 mL) and water (20 mL). The layers were separated and the aqueous layer was extracted once with Et_2O (30 mL). The combined organic extracts were washed with brine (25 mL), dried over $MgSO_4$ and concentrated under reduced pressure. The residue was purified by column chromatography (hexane/ Et_2O, 100:1 → hexane/ Et_2O, 50:1) to give **33** (1.00 g, 98%) as a light purple oil.

$\mathbf{R}_f$ = 0.56 (hexane/Et_2O, 50:1)
^{1}**H-NMR** (400.1 MHz, $CDCl_3$): δ = 6.08 (m_c, 1H), 5.76 (m_c, 1H), 3.73 (t, *J* = 6.3 Hz, 2H), 2.59 (dt, *J* = 0.9, 6.3 Hz, 2H), 0.89 (s, 9H), 0.07 (s, 6H).
^{13}**C-NMR** (100.6 MHz, $CDCl_3$): δ = 127.4, 107.6, 61.7, 48.4, 25.9, 18.3, −5.2.

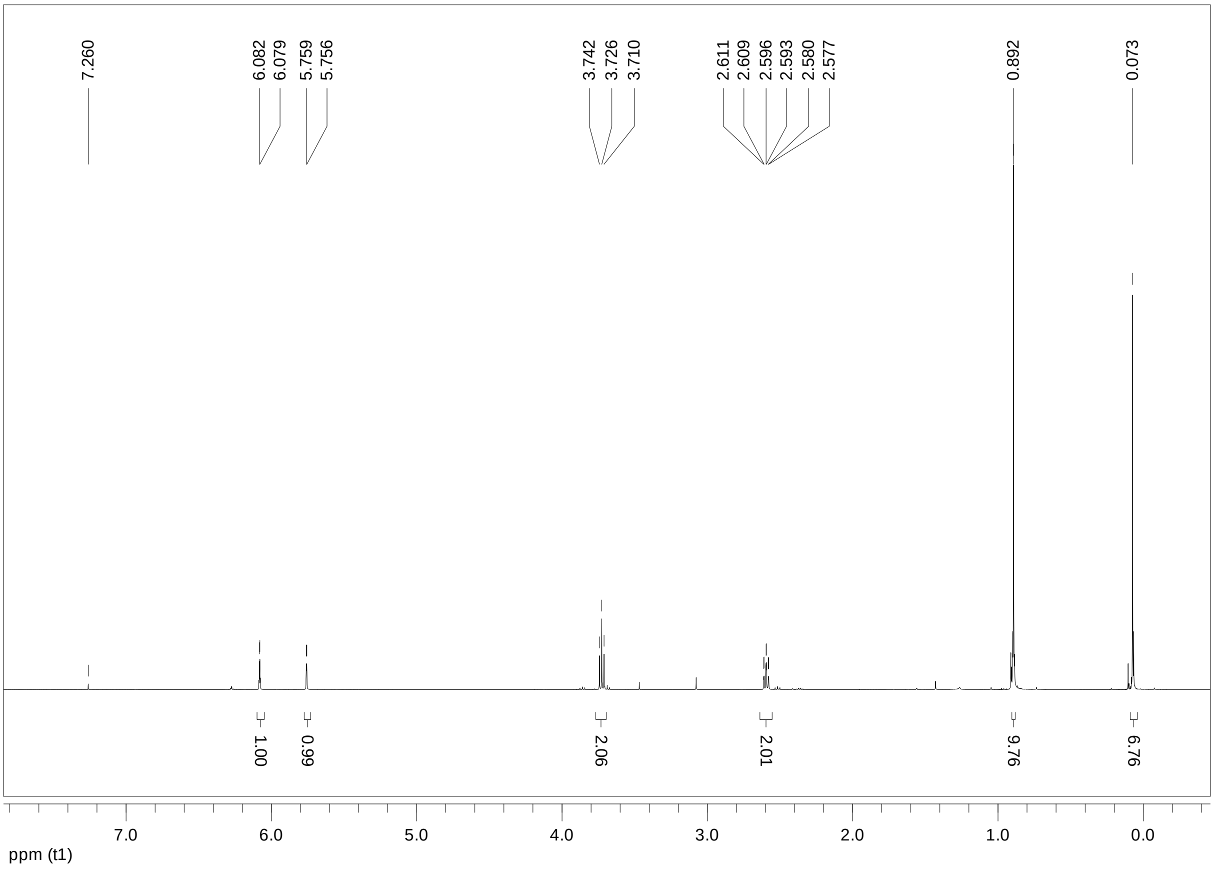
7.260
6.082
6.079
5.759
5.756
3.742
3.726
3.710
2.611
2.609
2.596
2.593
2.580
2.577
0.892
0.073
1.00
0.99
2.06
2.01
9.76
6.76
7.0
6.0
5.0
4.0
3.0
2.0
1.0
0.0
ppm (t1)

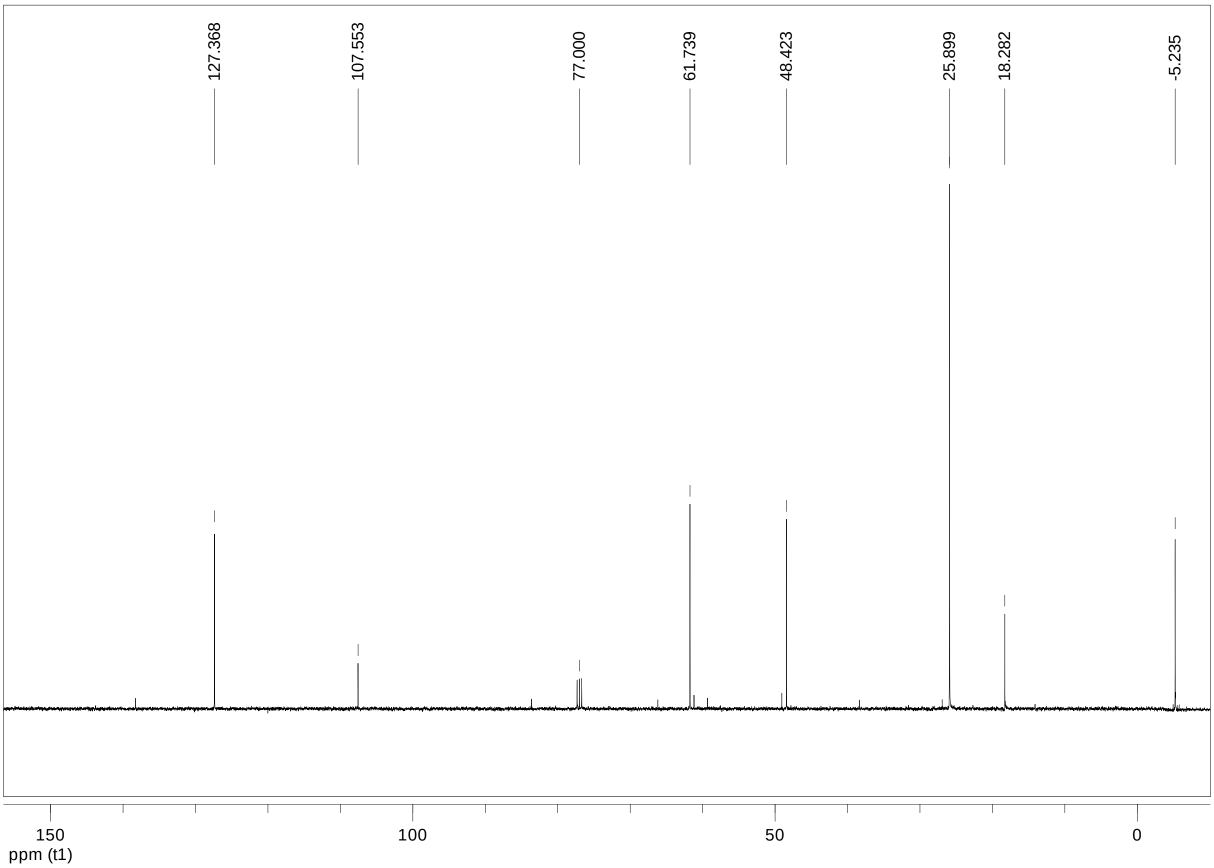
127.368
107.553
77.000
61.739
48.423
25.899
18.282
-5.235
150
100
50
0
ppm (t1)

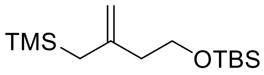

12a

***tert*-Butyldimethyl((3-((trimethylsilyl)methyl)but-3-en-1-yl)oxy)silane (12a):** To a slurry of LiCl (37.4 mg, 0.88 mmol, 4.0 equiv) and $Pd(PPh_3)_4$ (1.27 mg, 0.5 mol%) in Et_2O (3.6 mL) was added a solution of **33** (68.9 mg, 0.22 mmol, 1.0 equiv) in Et_2O (2.1 mL). After 10 min, (trimethylsilyl)methylmagnesium chloride (0.40 mL, 2.0 equiv, 1.1 M solution in Et_2O)[9] was added at once. After stirring at rt for 1 h, the reaction mixture was quenched with sat. $NaHCO_3$ (10 mL) and was extracted with hexane/Et_2O (1:1, 3 x 30 mL). The combined organic extracts were washed with brine, dried over $MgSO_4$ and concentrated under reduced pressure. The crude product was purified by column chromatography (hexane/Et_2O, 0.5% NEt_3, 100% → 400:1→ 200:1) to provide **12a** (55.7 mg, 93%) as a colorless oil.

R_f = 0.78 (hexane/Et_2O, 50:1)
^{1}H-NMR (400.1 MHz, C_6D_6): δ = 4.75 (m_c, 1H), 4.67 (m_c, 1H), 3.74 (t, J = 6.9 Hz, 2H), 2.26 (dt, J = 0.9, 6.9 Hz, 2H), 1.53 (d, J = 0.9 Hz, 2H), 0.98 (s, 9H), 0.07 (s, 6H), 0.01 (s, 9H).
^{13}C-NMR (100.6 MHz, C_6D_6): δ = 144.8, 108.9, 62.5, 42.0, 27.4, 26.1, 18.5, -1.3, -5.2.

[9] For the preparation of the *Grignard* reagent see procedure of **96**.

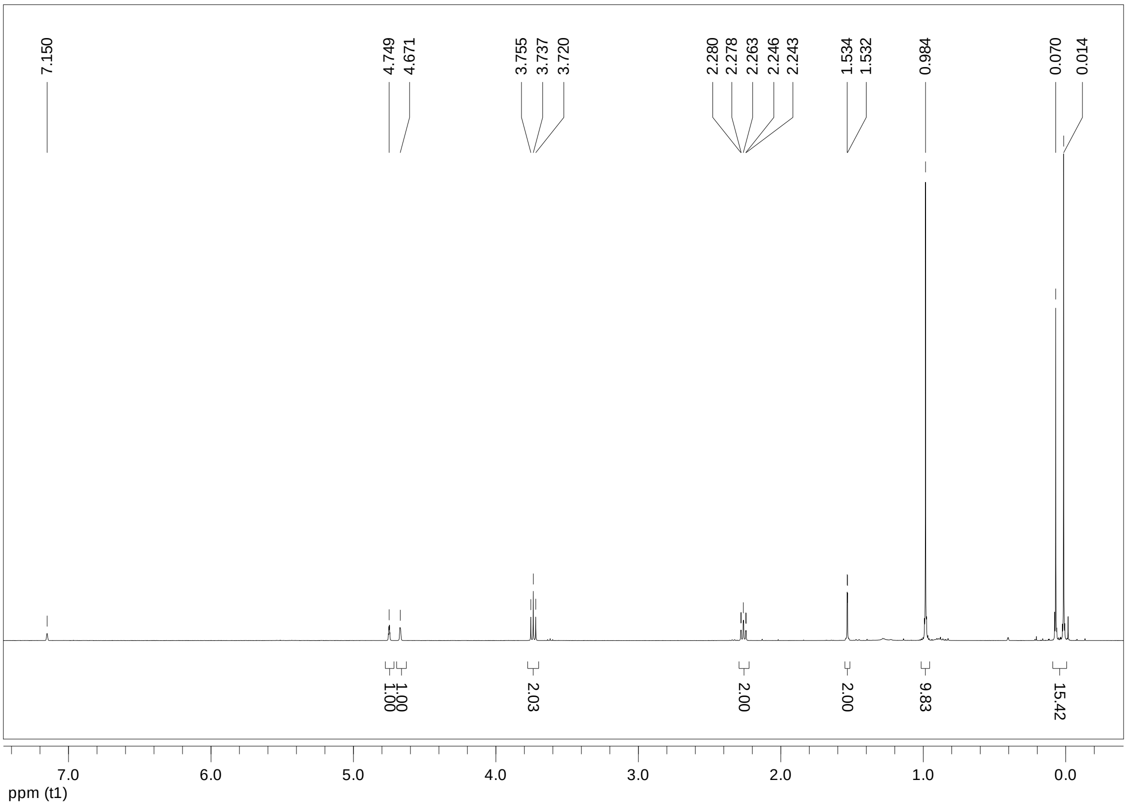
7.150
4.749
4.671
3.755
3.737
3.720
2.280
2.278
2.263
2.246
2.243
1.534
1.532
0.984
0.070
0.014
1.00
1.00
2.03
2.00
2.00
9.83
15.42
7.0
6.0
5.0
4.0
3.0
2.0
1.0
0.0
ppm (t1)

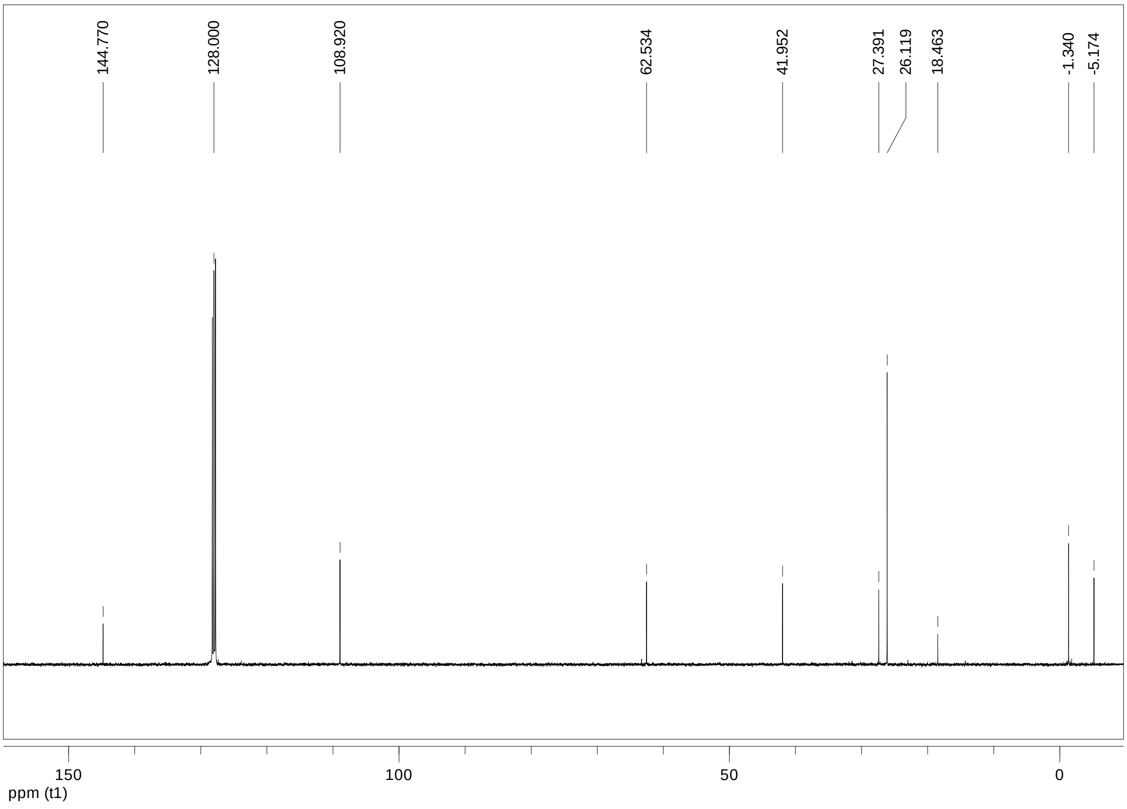
144.770
128.000
108.920
62.534
41.952
27.391
26.119
18.463
-1.340
-5.174
150
100
50
0
ppm (t1)

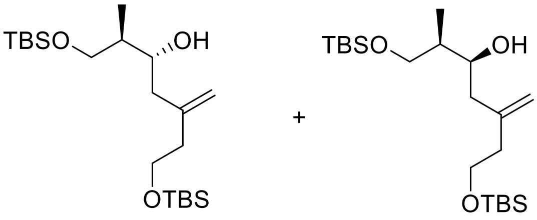

34a (major) **35a** (minor)

(6*R*,7*R*)-2,2,3,3,6,13,13,14,14-Nonamethyl-9-methylene-4,12-dioxa-3,13-disilapentadecan-7-ol (34a): To a stirring suspension of **12a** (178 mg, 0.65 mmol, 2.1 equiv) and powdered 4 Å molecular sieves (20 mg) in CH_2Cl_2 (1.8 mL) was added dropwise $SnCl_4$ (0.62 mL, 0.62 mmol, 2.0 equiv, 1.0 M solution in CH_2Cl_2) at −78 °C. After stirring for 15 min at that temperature, a solution of **11a** (62.7 mg, 0.31 mmol, 1.0 equiv) in CH_2Cl_2 (2 mL) was added dropwise. After stirring for 3 h at −78 °C, the reaction mixture was quenched with sat. $NaHCO_3$ (5 mL). The layers were separated and the aqueous phase was extracted with CH_2Cl_2 (3 x 15 mL). The combined organic extracts were washed with brine, dried over $MgSO_4$ and concentrated under reduced pressure. The crude product was purified by column chromatography (hexane/Et_2O, 10:1 → 4:1 → 2:1) to provide an inseparable mixture of **34a/35a** (88.2 mg, 71% over 2 steps, *dr* 10:6.2) as a colorless oil. The analytical data are reported for the mixture of diastereoisomers.

$\mathbf{R}_f$ = 0.43 and 0.38 (hexane/ Et_2O, 5:1)

^{1}H-NMR (400.1 MHz, $CDCl_3$): δ = 4.94-4.83 (m, 2H), 4.00-3.58 (m, 5H), 3.26 (d, *J* = 3.1 Hz, OH minor), 2.83 (d, *J* = 2.7 Hz, OH major), 2.37-2.07 (m, 4H), 1.80-1.66 (m, 1H), 0.93 (d, *J* = 7.0 Hz, Me major), 0.91 (d, *J* = 5.4 Hz, Me minor), 0.90-0.87 (2s, 18H), 0.08-0.03 (2s, 6H).

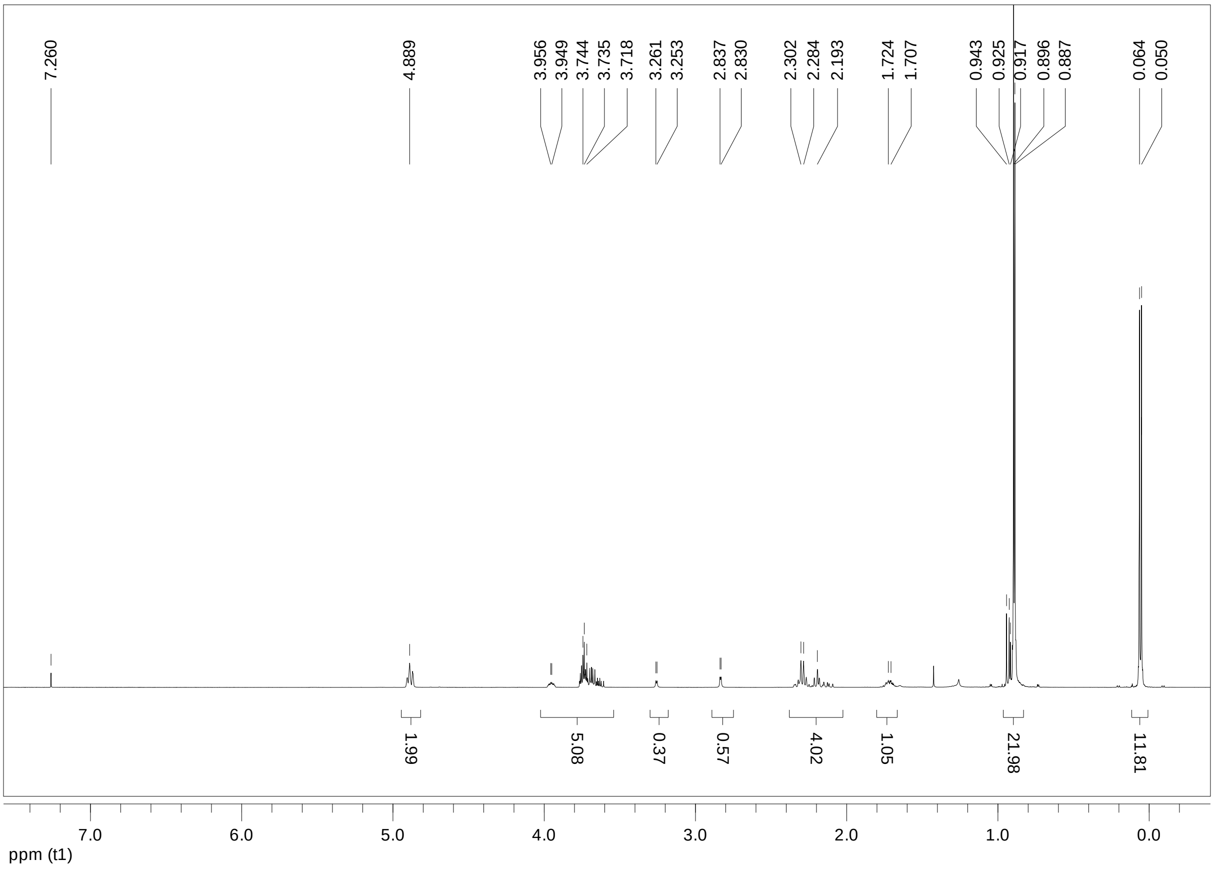
7.260
4.889
3.956
3.949
3.744
3.735
3.718
3.261
3.253
2.837
2.830
2.302
2.284
2.193
1.724
1.707
0.943
0.925
0.917
0.896
0.887
0.064
0.050
1.99
5.08
0.37
0.57
4.02
1.05
21.98
11.81
7.0
6.0
5.0
4.0
3.0
2.0
1.0
0.0
ppm (t1)

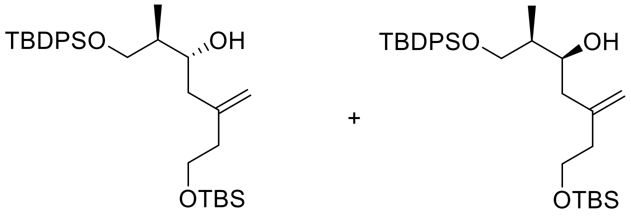

34b (major) **35b** (minor)

(6*R*,7*R*)-2,2,6,13,13,14,14-Heptamethyl-9-methylene-3,3-diphenyl-4,12-dioxa-3,13-disilapentadecan-7-ol (34b): To a stirring solution of **11b** (103 mg, 0.32 mmol, 1.0 equiv) and **12a** (188 mg, 0.69 mmol, 2.2 equiv) in CH_2Cl_2 (4 mL) was added dropwise at −78 °C $TiCl_4$ (0.35 mL, 0.35 mmol, 1.1 equiv, 1.0 M solution in CH_2Cl_2). After stirring for 1.5 h at −78 °C, the reaction mixture was quenched with sat. $NaHCO_3$ (15 mL). The layers were separated and the aqueous phase was extracted with CH_2Cl_2 (3 x 25 mL). The combined organic extracts were washed with brine, dried over $MgSO_4$ and concentrated under reduced pressure. The crude product was purified by column chromatography (hexane/Et_2O, 20:1 → 10:1 → 5:1) to provide an inseparable mixture of **34b/35b** (62.1 mg, 37% over 2 steps, *dr* 3:1.72) as a colorless oil. The analytical data are reported for the mixture of diastereoisomers.

$\mathbf{R}_f$ = 0.51 and 0.45 (hexane/ Et_2O, 5:1)

^{1}H-NMR (400.1 MHz, $CDCl_3$): δ = 7.75-7.62 (m, 4H), 7.49-7.35 (m, 6H), 4.95-4.85 (m, 2H), 4.03 (m_c, 1H), 3.85-3.62 (m, 4H), 3.04 (d, *J* = 3.1 Hz, OH minor), 2.61 (d, *J* = 3.1 Hz, OH major), 2.40-2.07 (m, 4H), 1.86-1.70 (m, 1H), 1.07 (s, 9H), 0.97-0.92 (2d, 3H), 0.90 (s, 9H), 0.06 (s, 6H).

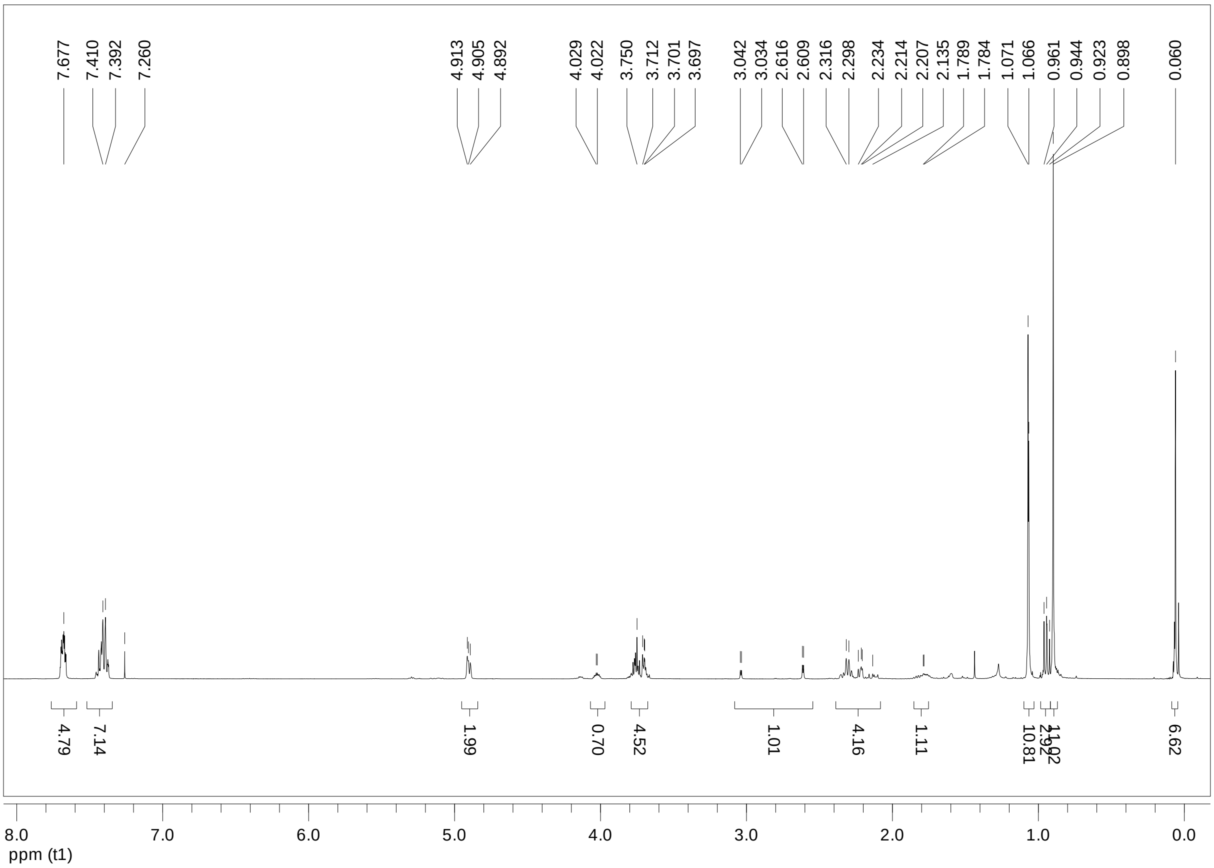
7.677
7.410
7.392
7.260
4.913
4.905
4.892
4.029
4.022
3.750
3.712
3.701
3.697
3.042
3.034
2.616
2.609
2.316
2.298
2.234
2.214
2.207
2.135
1.789
1.784
1.071
1.066
0.961
0.944
0.923
0.898
0.060
4.79
7.14
1.99
0.70
4.52
1.01
4.16
1.11
10.81
2.92
11.02
6.62
8.0
7.0
6.0
5.0
4.0
3.0
2.0
1.0
0.0
ppm (t1)

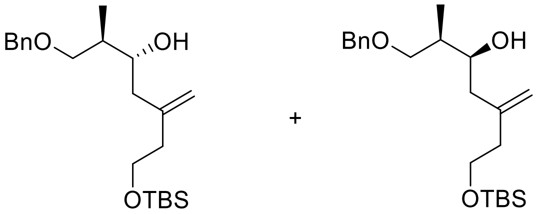

34c (minor) **35c** (major)

(2*R*,3*S*)-1-(Benzyloxy)-7-(*tert*-butyldimethylsilyloxy)-2-methyl-5-methyleneheptan-3-ol (35c): To a stirring solution of **11c** (26.2 mg, 0.15 mmol, 1.0 equiv) and **12a** (105 mg, 0.39 mmol, 2.6 equiv) in CH_2Cl_2 (1.7 mL) was added at −78 °C $TiCl_4$ (0.20 mL, 0.20 mmol, 1.4 equiv, 1.0 M solution in CH_2Cl_2). After stirring for 1.5 h at −78 °C, the reaction mixture was quenched with sat. $NaHCO_3$ (5 mL) and extracted with CH_2Cl_2 (3 x 15 mL). The combined organic extracts were washed with brine, dried over $MgSO_4$ and concentrated under reduced pressure. The crude product was purified by column chromatography (hexane/Et_2O, 10:1 → 4:1 → 2:1) to provide an inseparable mixture of **35c**/**34c** (27.7 mg, 50% over 2 steps, *dr* 10:3) as a colorless oil. The analytical data are reported for the major isomer **35c**:

$\mathbf{R}_f$ = 0.26 (hexane/ Et_2O, 5:1)

^{1}H-NMR (400.1 MHz, $CDCl_3$): δ = 7.39-7.25 (m, 5H), 4.90 (s, 2H), 4.52 (s, 2H), 3.74 (t, *J* = 6.8 Hz, 2H), 3.72-3.65 (m, 1H), 3.55 (dd, *J* = 2.1, 5.7 Hz, 2H), 2.36-2.25 (m, 3H), 2.10 (dd, *J* = 9.8, 13.9 Hz, 1H), 1.89 (m_c, 1H), 0.97 (d, *J* = 6.9 Hz, 3H), 0.89 (s, 9H), 0.05 (s, 6H).

^{13}C-NMR (100.6 MHz, $CDCl_3$): δ = 144.3, 138.2, 128.4, 127.6, 113.8, 73.8, 73.3, 72.4, 62.4, 42.2, 38.9, 38.7, 25.9, 18.3, 14.0, -5.3.

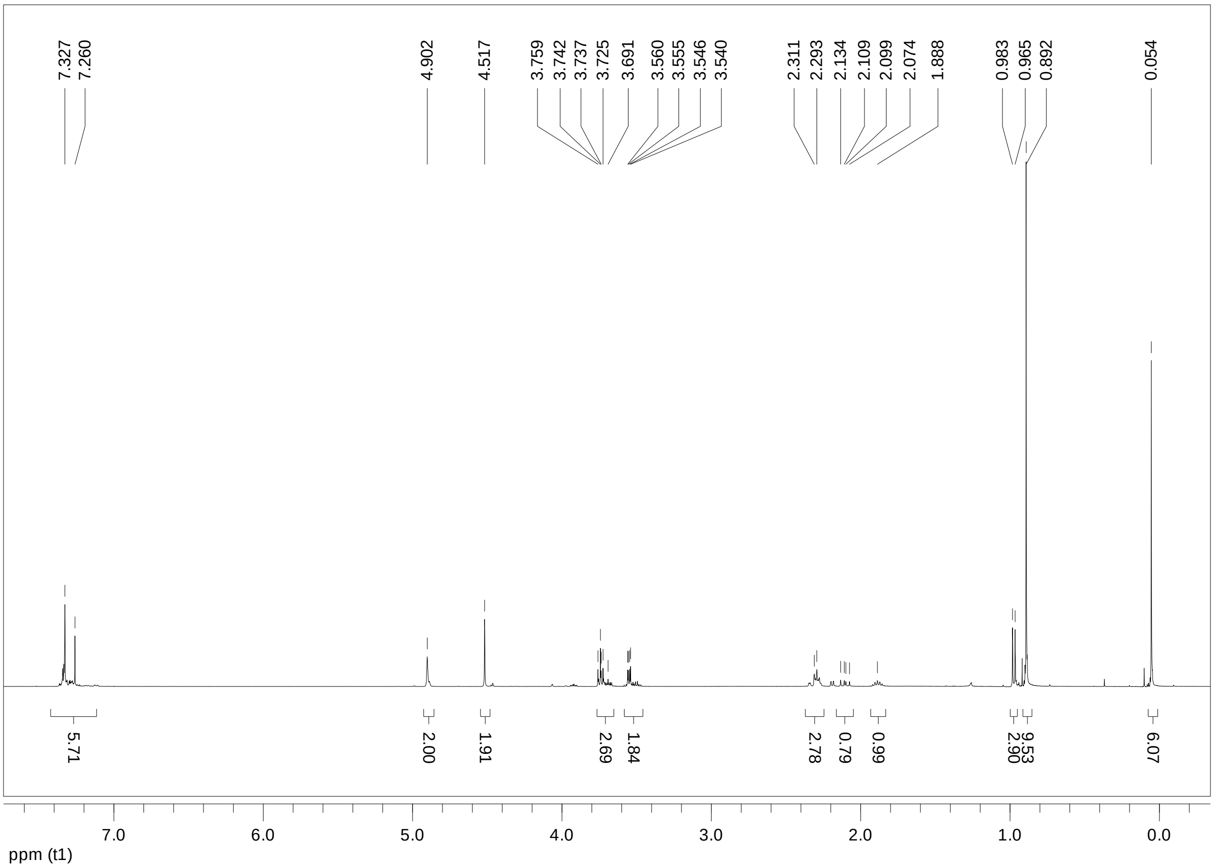
7.327
7.260
4.902
4.517
3.759
3.742
3.737
3.725
3.691
3.560
3.555
3.546
3.540
2.311
2.293
2.134
2.109
2.099
2.074
1.888
0.983
0.965
0.892
0.054
5.71
2.00
1.91
2.69
1.84
2.78
0.79
0.99
2.90
9.53
6.07
7.0
6.0
5.0
4.0
3.0
2.0
1.0
0.0
ppm (t1)

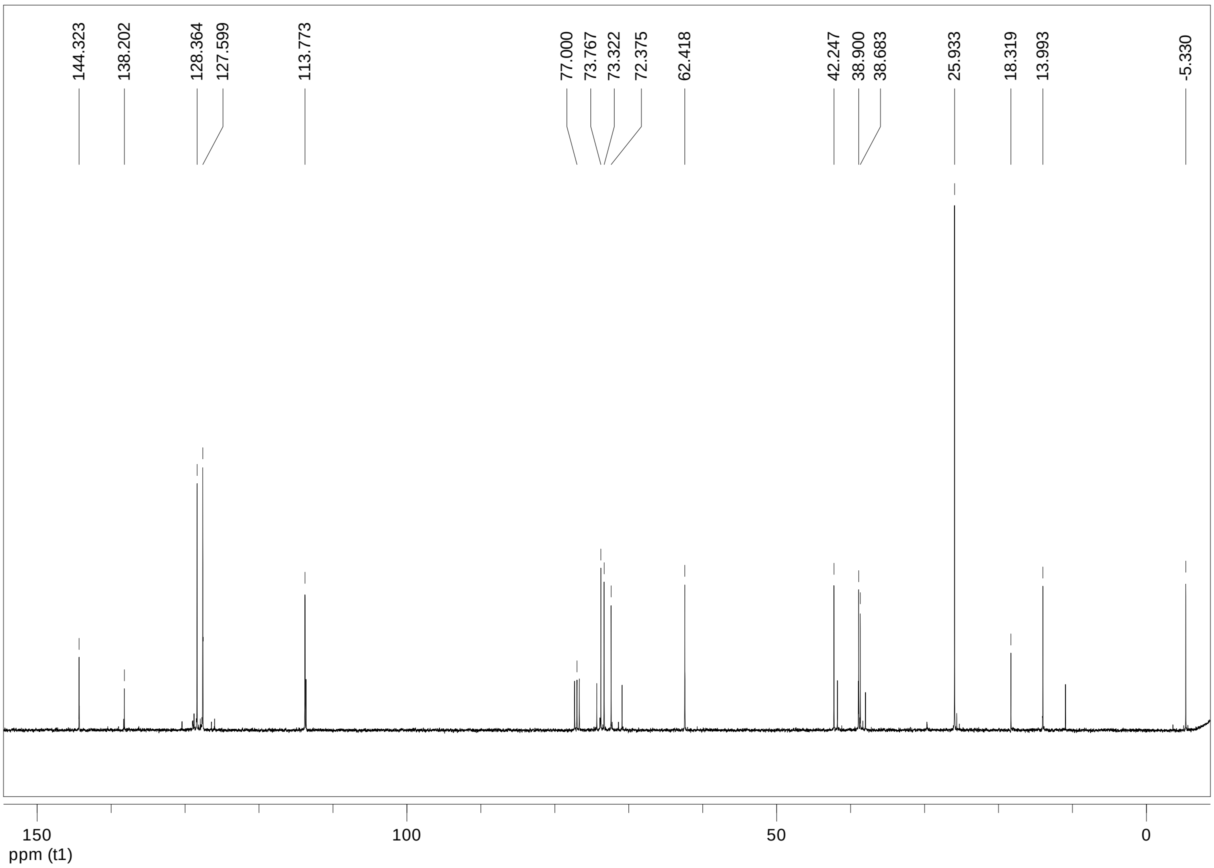
144.323
138.202
128.364
127.599
113.773
77.000
73.767
73.322
72.375
62.418
42.247
38.900
38.683
25.933
18.319
13.993
-5.330
150
100
50
0
ppm (t1)

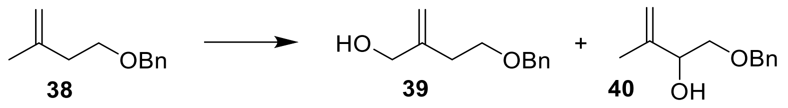

4-(Benzyloxy)-2-methylenebutan-1-ol (39) and 1-(benzyloxy)-3-methylbut-3-en-2-ol (40): To a solution of **38**[10] (252 mg, 1.43 mmol, 1.0 equiv) in CH_2Cl_2 (7 mL) was added *t*-BuOOH (0.29 mL, 1.45 mmol, 1.0 equiv, 5.0 M in decane) and SeO_2 (16.9 mg, 0.15 mmol, 10 mol%). After stirring for 2 h at rt, MeOH (7 mL) and $NaBH_4$ (210 mg, 5.55 mmol, 3.9 equiv) were added to the reaction mixture at 0 °C and stirring was continued for 30 min at that temperature. Sat. NH_4Cl (25 mL) was added and the aqueous layer was extracted with CH_2Cl_2 (3 x 30 mL). The combined organic extracts were washed with brine, dried over $MgSO_4$ and concentrated under reduced pressure. The residue was purified by column chromatography (hexane/Et_2O, 6:1 → 4:1 → 2:1 → 1:1) to provide **39** (64.5 mg, 23%, 38% brsm) as a yellow oil.[11] Furthermore, secondary alcohol **40** (76.6 mg, 28%) and starting material **38** (97.3 mg, 39%) were isolated. The analytical data for **39** matched those reported below.

Analytical data for **40**:

R*f* = 0.30 (hexane/Et_2O, 2:1)

^{1}H-NMR (400.1 MHz, $CDCl_3$): δ = 7.40-7.27 (m, 5H), 5.07 (m_c, 1H), 4.93 (m_c, 1H), 4.58 (s, 2H), 4.29 (dd, *J* = 2.0, 7.8 Hz, 1H), 3.59 (dd, *J* = 3.3, 9.6 Hz, 1H), 3.43 (dd, *J* = 8.1, 9.6 Hz, 1H), 2.54 (brs, 1H), 1.74 (s, 3H).

^{13}C-NMR (100.6 MHz, $CDCl_3$): δ = 143.8, 137.8, 128.4, 127.8, 127.7, 112.0, 73.3 (2C), 18.7.

[10] Benzyl ether **38** was prepared according to a literature procedure: Cleary, P. A.; Woerpel, K. A. *Org. Lett.* **2005**, *7*, 5531-5533.
[11] Procedure adapted from: Smith, A. B. *et al. Tetrahedron* **2009**, *65,* 6489-6509.

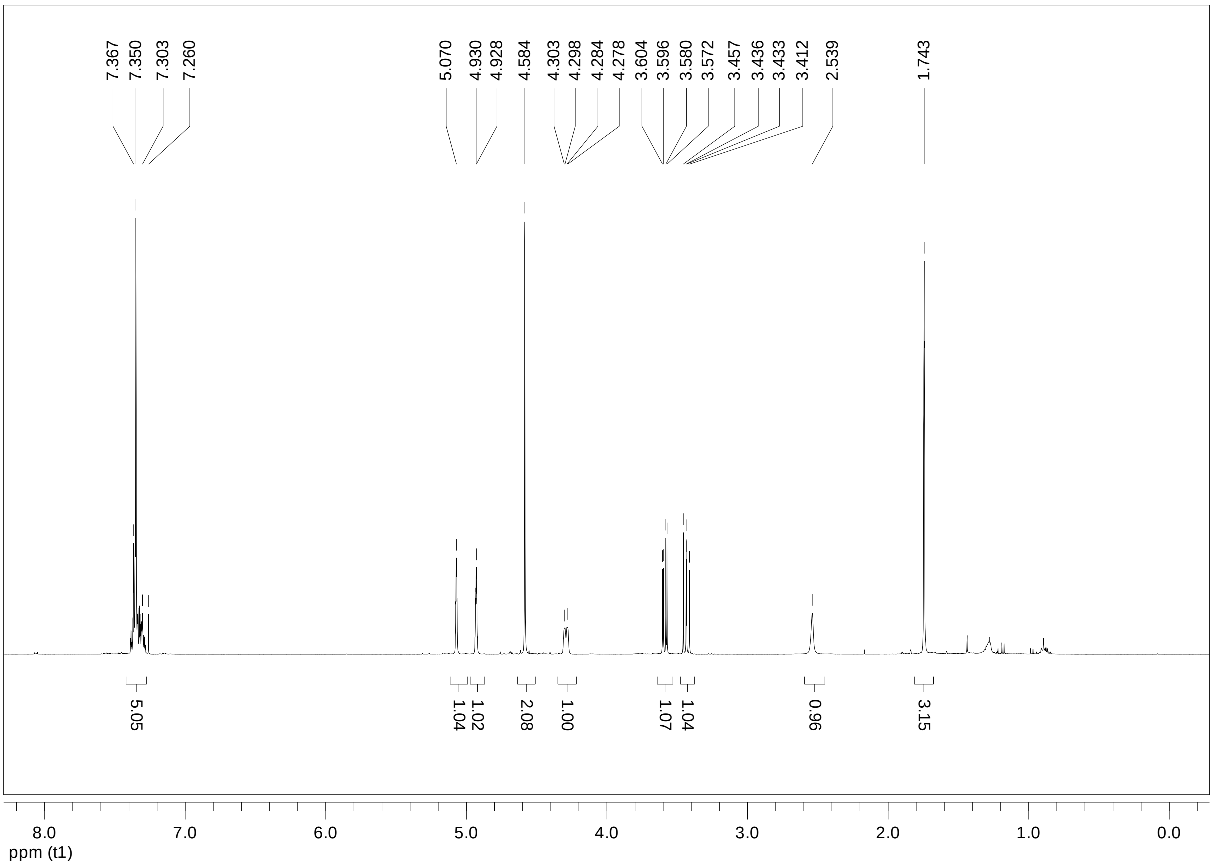
7.367
7.350
7.303
7.260
5.070
4.930
4.928
4.584
4.303
4.298
4.284
4.278
3.604
3.596
3.580
3.572
3.457
3.436
3.433
3.412
2.539
1.743
5.05
1.04
1.02
2.08
1.00
1.07
1.04
0.96
3.15
8.0
7.0
6.0
5.0
4.0
3.0
2.0
1.0
0.0
ppm (t1)

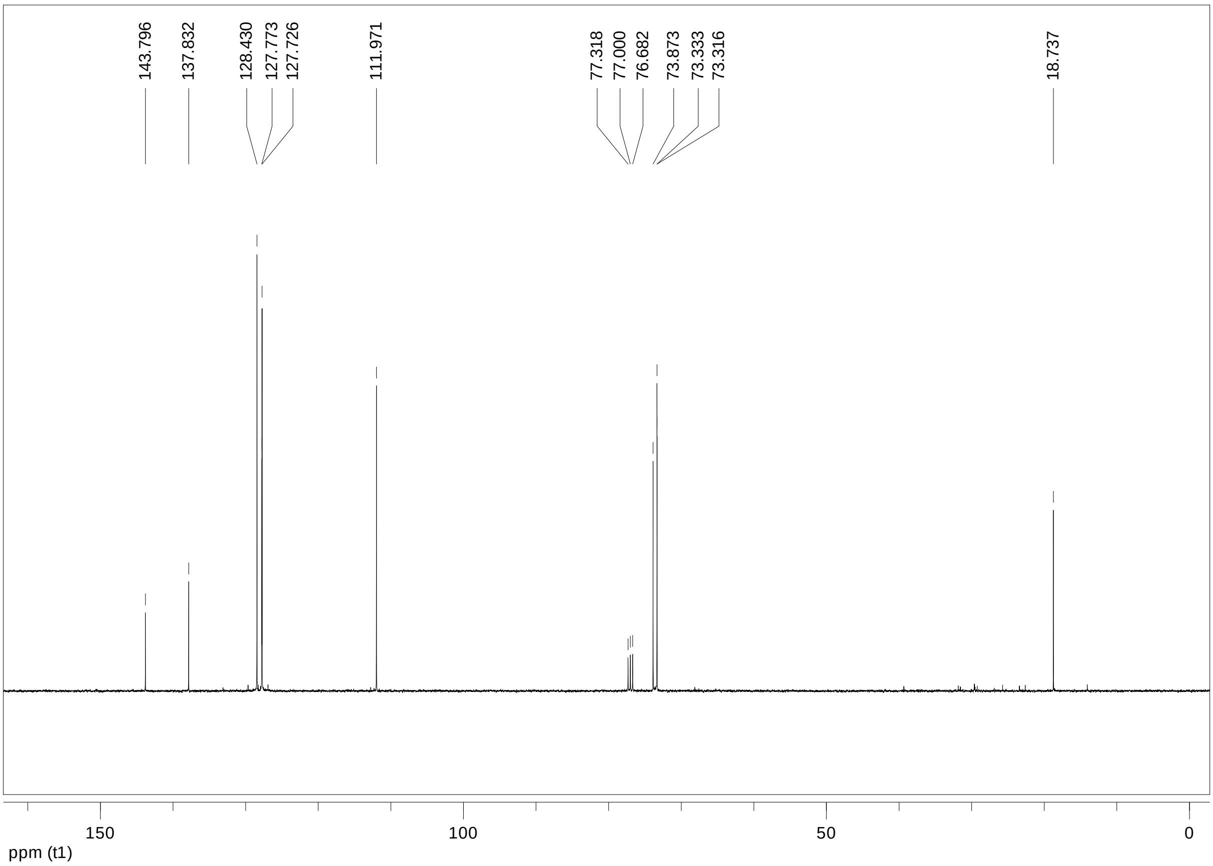
143.796
137.832
128.430
127.773
127.726
111.971
77.318
77.000
76.682
73.873
73.333
73.316
18.737
150
100
50
0
ppm (t1)

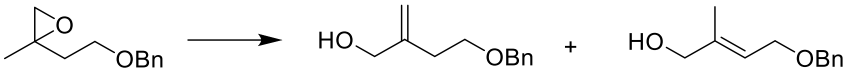

42 39 43

4-(Benzyloxy)-2-methylenebutan-1-ol (39) and (*E*)-4-(benzyloxy)-2-methylbut-2-en-1-ol (43): To a stirred solution of 2,2,6,6-tetramethylpiperidine (0.59 mL, 3.51 mmol, 2.25 equiv) in toluene (9.5 mL) was added at 0 °C *n*-BuLi (2.15 mL, 3.43 mmol, 2.20 equiv, 1.6 M in hexane) and stirring was continued for 30 min at that temperature. Then, Et_2AlCl (3.43 mL, 3.43 mmol, 2.20 equiv, 1.0 M in hexane) was added. After stirring for 30 min at 0 °C, a solution of **42**[12] (300 mg, 1.56 mmol, 1.00 equiv) in toluene (2.4 mL) was added slowly *via* syringe down the side of the flask. After 3 h at 0 °C, 2 M HCl (10 mL), water (50 mL) and Et_2O (100 mL) were added. The reaction mixture was extracted with Et_2O (5 x 60 mL) and the combined organic extracts were washed with brine, dried over $MgSO_4$ and concentrated under reduced pressure. The residue was purified by column chromatography (hexane/Et_2O, 10:1 → 5:1 → 2:1 → 1:1→ 0:1) to provide the desired alcohol **39** (144 mg, 48%) and the regioisomers **43** (46.9 mg, 16%). The analytical data for **39** matched those reported below.

Analytical data for **43**:

R*f* = 0.39 (hexane/ EtOAc, 2:1)
^{1}H-NMR (400.1 MHz, $CDCl_3$): δ = 7.38-7.26 (m, 5H), 5.66 (m_c, 1H), 4.52 (s, 2H), 4.08 (d, *J* = 6.6 Hz, 2H), 4.00 (s, 2H), 2.10 (brs, 1H), 1.66 (s, 3H).
^{13}C-NMR (100.6 MHz, $CDCl_3$): δ = 139.3, 138.2, 128.3, 127.7, 127.6, 121.2, 72.3, 67.8, 66.1, 13.8.

---

[12] Epoxide **42** was prepared according to a literature procedure: Muehlbacher, M.; Poulter, C. D. *J. Org. Chem.* **1988**, *53*, 1026-1030.

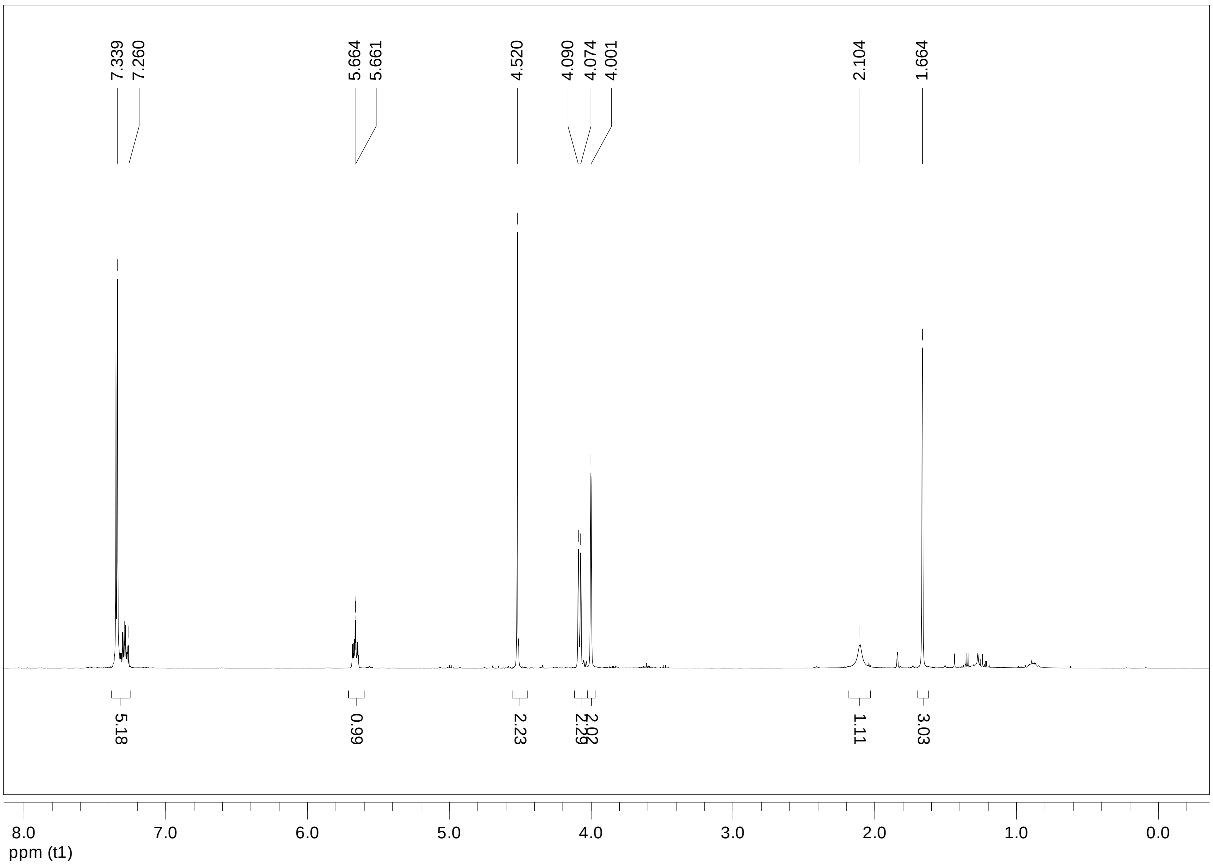
7.339
7.260
5.664
5.661
4.520
4.090
4.074
4.001
2.104
1.664
5.18
0.99
2.23
2.26
2.02
1.11
3.03
8.0
7.0
6.0
5.0
4.0
3.0
2.0
1.0
0.0
ppm (t1)

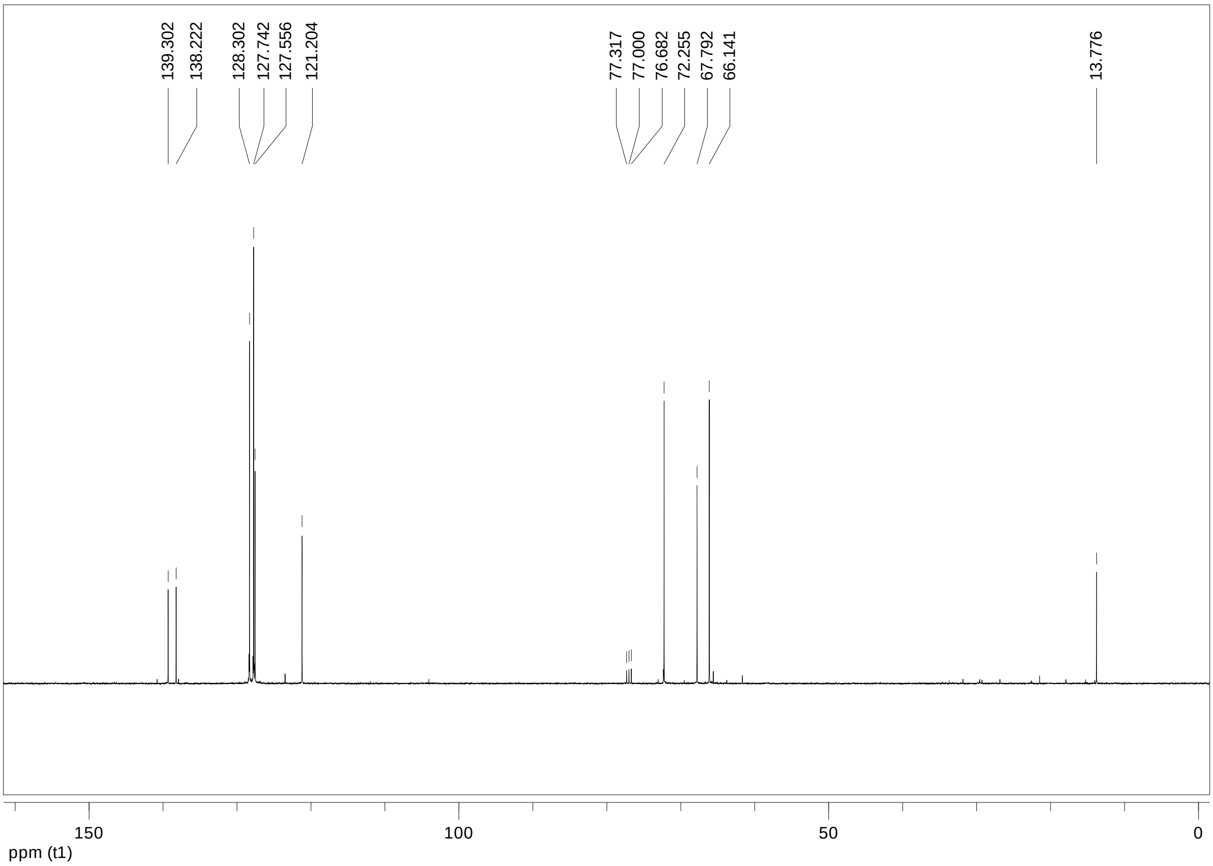
139.302
138.222
128.302
127.742
127.556
121.204
77.317
77.000
76.682
72.255
67.792
66.141
13.776
150
100
50
0
ppm (t1)

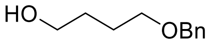

45

4-(Benzyloxy)butan-1-ol (45): To a vigorously stirring suspension of NaH (4.44 g, 111 mmol, 1.2 equiv, 60% dispersion in mineral oil) in THF (151 mL) was added at 0 °C quickly a solution of 1,4-butanediol (**44**) (41 mL, 463 mmol, 5.0 equiv) in THF (61 mL) *via* an addition funnel. After stirring for 50 min at rt, the reaction mixture was again cooled to 0 °C and BnBr (11.0 mL, 92.5 mmol, 1.0 equiv) was added dropwise *via* syringe. After stirring for 14 h at rt, the reaction was quenched with sat. NH_4Cl (80 mL) and water (50 mL). The layers were separated and the aqueous phase was extracted with Et_2O (2 x 80 mL). The combined organic extracts were dried over $MgSO_4$ and concentrated under reduced pressure. The residue was purified by column chromatography (hexane/EtOAc, 2:1 → 1:1) to afford **45** (16.2 g, 97%) as a colorless oil.[13]

$\mathbf{R}_f$ = 0.28 (hexane/EtOAc, 2:1)
^{1}H-NMR (400.1 MHz, $CDCl_3$): δ = 7.40-7.26 (m, 5H), 4.52 (s, 2H), 3.65 (t, *J* = 5.9 Hz, 2H), 3.52 (t, *J* = 5.8 Hz, 2H), 2.04 (bs, 1H), 1.74-1.66 (m_c, 4H).
^{13}C-NMR (100.6 MHz, $CDCl_3$): δ = 138.2, 128.4, 127.7, 127.6, 73.0, 70.3, 62.7, 30.1, 26.7.
IR (neat, ν/cm^{-1}): 3372br, 2938w, 2861m, 1496w, 1453m, 1362m, 1205w, 1096m, 1059s, 1028m, 957w, 734s, 697s, 611m.
HRMS (EI): *m/z* calcd for $C_{11}H_{16}O_2$ $[M]^+$: 180.1145, found: 180.1142.

[13] Crimmins, M. T.; DeBaillie, A. C. *J. Am. Chem. Soc.* **2006**, *128*, 4936-4937.

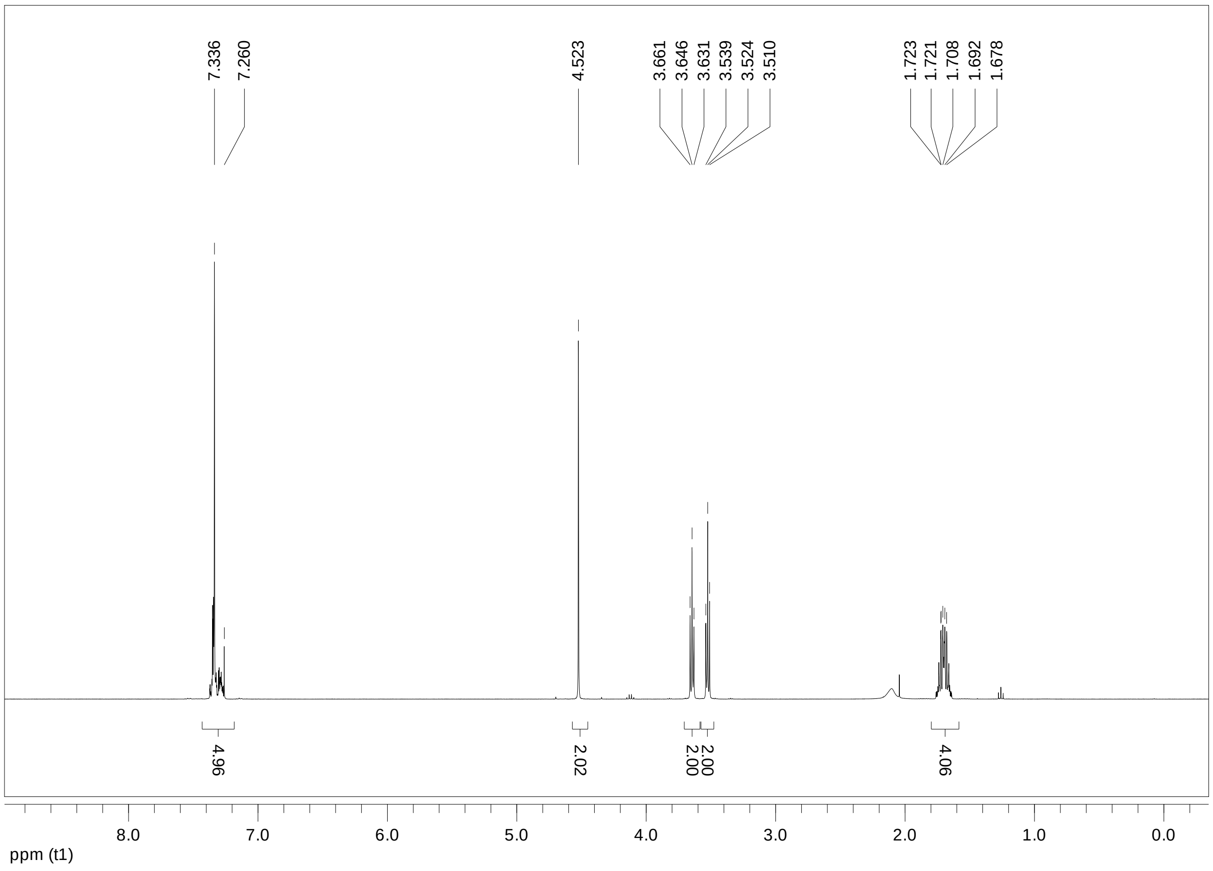
7.336
7.260
4.523
3.661
3.646
3.631
3.539
3.524
3.510
1.723
1.721
1.708
1.692
1.678
4.96
2.02
2.00
2.00
4.06
8.0 7.0 6.0 5.0 4.0 3.0 2.0 1.0 0.0
ppm (t1)

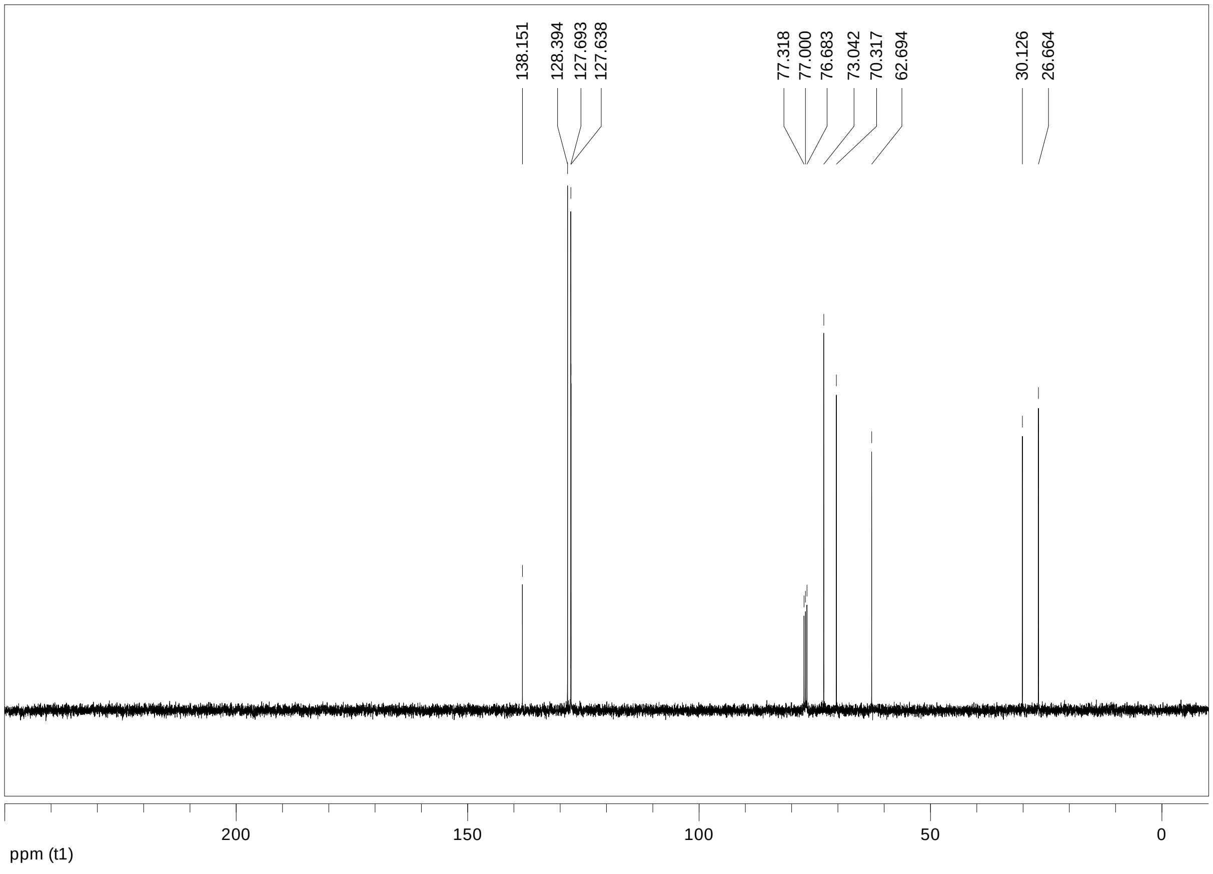
138.151
128.394
127.693
127.638
77.318
77.000
76.683
73.042
70.317
62.694
30.126
26.664
200 150 100 50 0
ppm (t1)

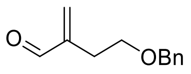

46

4-(Benzyloxy)-2-methylenebutanal (46): To a solution of oxalyl chloride (2.76 mL, 32.6 mmol, 1.5 equiv) in CH_2Cl_2 (157 mL) was added dropwise at −78 °C DMSO (4.64 mL, 65.3 mmol, 3.0 equiv). After stirring for 20 min at that temperature, a solution of **45** (3.92 g, 21.8 mmol, 1.0 equiv) in CH_2Cl_2 (42 mL + 10 mL of washing) was added dropwise over 20 min, and the mixture was stirred at −78 °C for 1 h. Triethylamine (15.2 mL, 109 mmol, 5.0 equiv) was added, and the resulting mixture was stirred at 0 °C for 30 min. CH_2NMe_2Cl (4.07 g, 43.5 mmol, 2.0 equiv) and DBU (3.25 mL, 21.8 mmol, 1.0 equiv) were then added; the mixture was stirred at rt for 22 h, the reaction was quenched with sat. NH_4Cl (150 mL), and the resulting mixture was extracted with CH_2Cl_2 (2 x 300 mL). The combined organic extracts were dried over $MgSO_4$ and concentrated under reduced pressure. The residue was purified by column chromatography (hexane/EtOAc, 30:1 → 10:1) to provide **46** (3.56 g, 86% over 2 steps) as a colorless oil.

R*f* = 0.33 (hexane/EtOAc, 10:1)
^{1}H-NMR (400.1 MHz, $CDCl_3$): δ = 9.54 (s, 1H), 7.39-7.24 (m, 5H), 6.39 (dd, *J* = 0.8, 2.0 Hz, 1H), 6.08 (d, *J* = 0.5 Hz, 1H), 4.51 (s, 2H), 3.60 (t, *J* = 6.4 Hz, 2H), 2.58 (dt, *J* = 0.7, 6.4 Hz, 2H).
^{13}C-NMR (100.6 MHz, $CDCl_3$): δ = 194.4, 147.0, 138.2, 135.6, 128.4, 127.6, 72.8, 67.9, 28.3.
IR (neat, ν/cm^{-1}): 3088w, 3063w, 3031w, 2925w, 2858w, 1686s, 1496w, 1454m, 1361m, 1101s, 1029w, 948m, 737s, 698s..
HRMS (EI): *m/z* calcd for $C_{12}H_{14}O_2$ [M]$^+$: 190.0989, found: 190.0991.

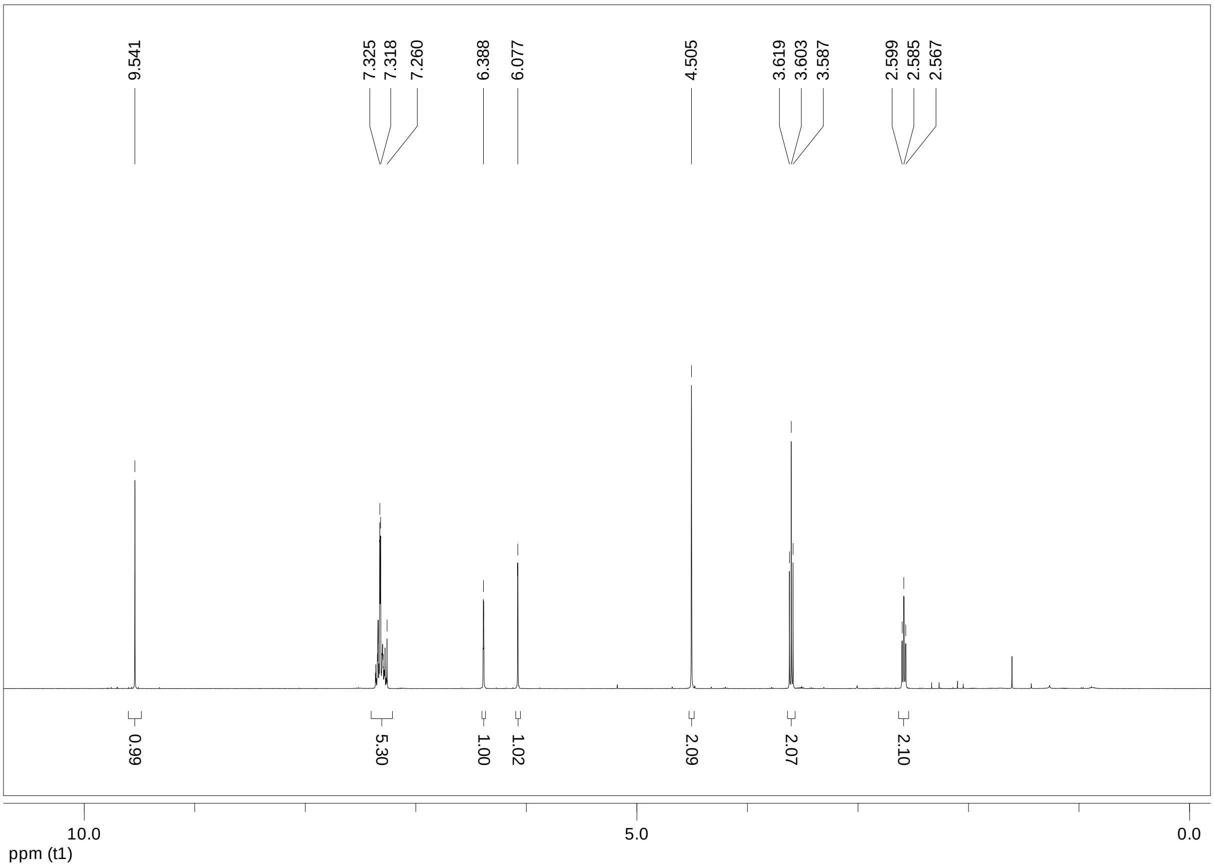
9.541
7.325
7.318
7.260
6.388
6.077
4.505
3.619
3.603
3.587
2.599
2.585
2.567
0.99
5.30
1.00
1.02
2.09
2.07
2.10
10.0
5.0
0.0
ppm (t1)

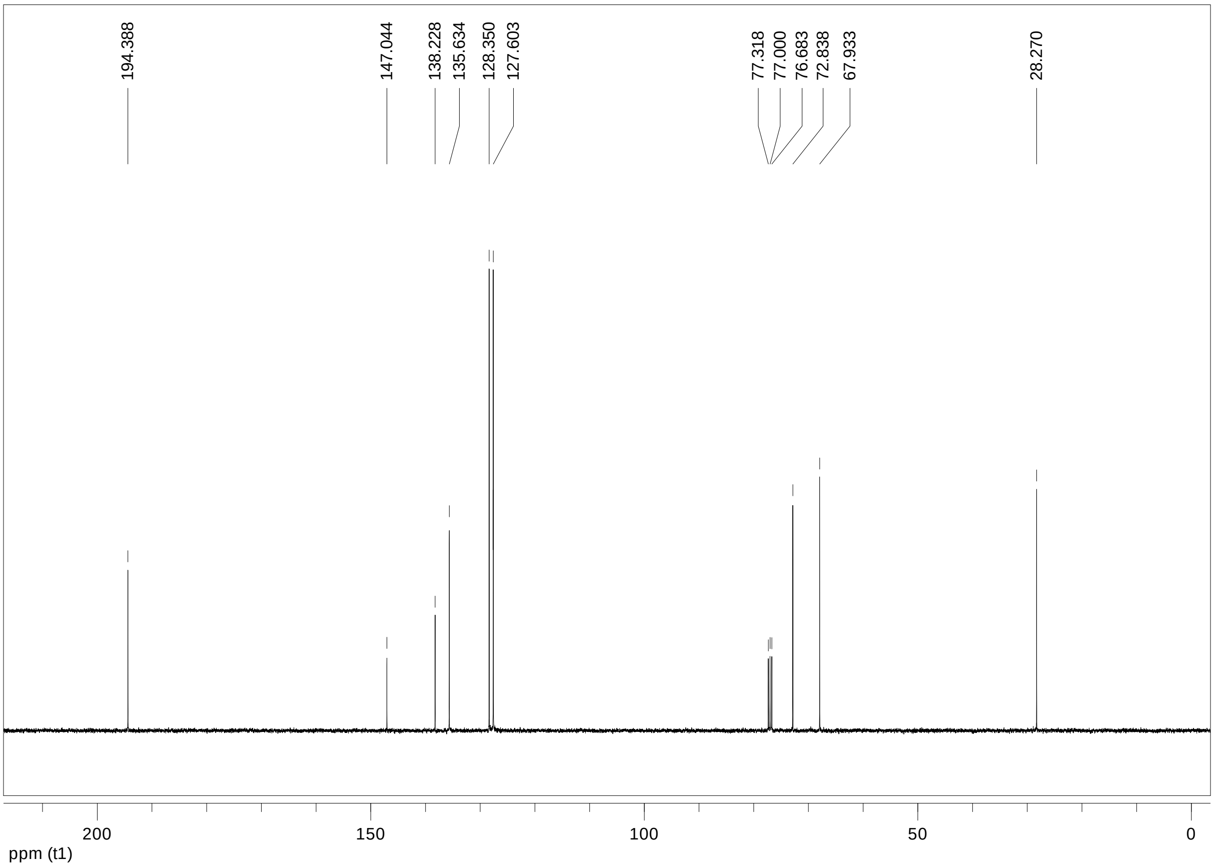
194.388
147.044
138.228
135.634
128.350
127.603
77.318
77.000
76.683
72.838
67.933
28.270
200
150
100
50
0
ppm (t1)

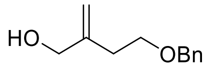

39

$NaBH_4$ reduction: A solution of **46** (3.55 g, 18.7 mmol, 1.0 equiv) in EtOH (40 mL) was added over 20 min to a stirred suspension of $NaBH_4$ (367 mg, 9.70 mmol, 0.52 equiv) in EtOH (100 mL) at 0 °C. After stirring at 0 °C for 40 min, brine (70 mL) and water (40 mL) were added, and the mixture was extracted with EtOAc (2 x 350 mL). The combined organic extracts were washed with brine (80 mL), dried over $MgSO_4$ and concentrated under reduced pressure. The residue was purified by column chromatography (hexane/EtOAc, 4:1 → 2:1) to provide **39** (3.38 g, 94%) as a colorless oil.

$LiAlH_4$ reduction: To a solution of **46** (12.7 g, 66.8 mmol, 1.0 equiv) in THF (600 mL) was added at 0 °C $LiAlH_4$ (1.32 g, 38.0 mmol, 0.52 equiv) in small portions over 5 min. After stirring at 0 °C for 30 min, water (16 mL) was added dropwise at 0 °C to the reaction mixture followed by 10% NaOH (25 mL) and water (150 mL). The colorless precipitate was filtered off and washed with THF. The mother liquor was transferred to a separatory funnel and diluted with Et_2O (150 mL) and (80 mL). The layers were separated and the aqueous layer was extracted with Et_2O (2 x 100 mL). The combined organic extracts were dried over $MgSO_4$ and concentrated under reduced pressure. The residue was purified by column chromatography (hexane/EtOAc, 4:1 → 2:1) to provide **39** (12.3 g, 96%) as a colorless oil.

R_f = 0.47 (hexane/ EtOAc, 2:1)
^{1}H-NMR (400.1 MHz, $CDCl_3$): δ = 7.39-7.26 (m, 5H), 5.07 (d, *J* = 1.4 Hz, 1H), 4.93 (s, 1H), 4.54 (s, 2H), 4.08 (s, 2H), 3.62 (t, *J* = 6.2 Hz, 2H), 2.42 (dt, *J* = 0.6, 6.2 Hz, 2H), 2.32 (brs, 1H).
^{13}C-NMR (100.6 MHz, $CDCl_3$): δ = 146.8, 137.9, 128.4, 127.7 (2C), 112.2, 73.2, 69.7, 66.3, 33.9.
IR (neat, ν/cm^{-1}): 3384br, 3087w, 3065w, 3031w, 2911w, 2860m, 1454m, 1362m, 1206w, 1099s, 1074s, 1028s, 901m, 738s, 698s, 615w.
HRMS (EI): *m/z* calcd for $C_{12}H_{16}O_2$ $[M]^+$: 192.1145, found: 192.1139.

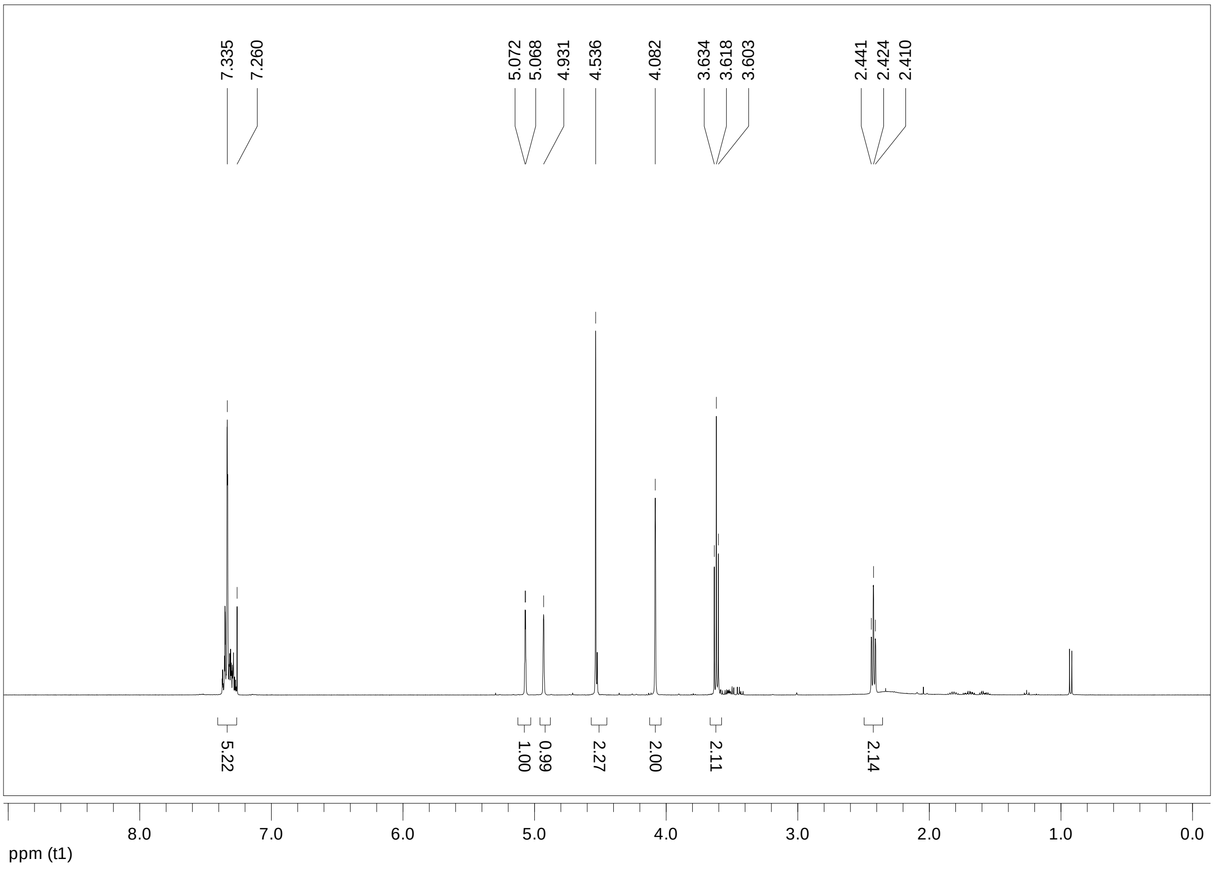
7.335
7.260
5.072
5.068
4.931
4.536
4.082
3.634
3.618
3.603
2.441
2.424
2.410
5.22
1.00
0.99
2.27
2.00
2.11
2.14
8.0
7.0
6.0
5.0
4.0
3.0
2.0
1.0
0.0
ppm (t1)

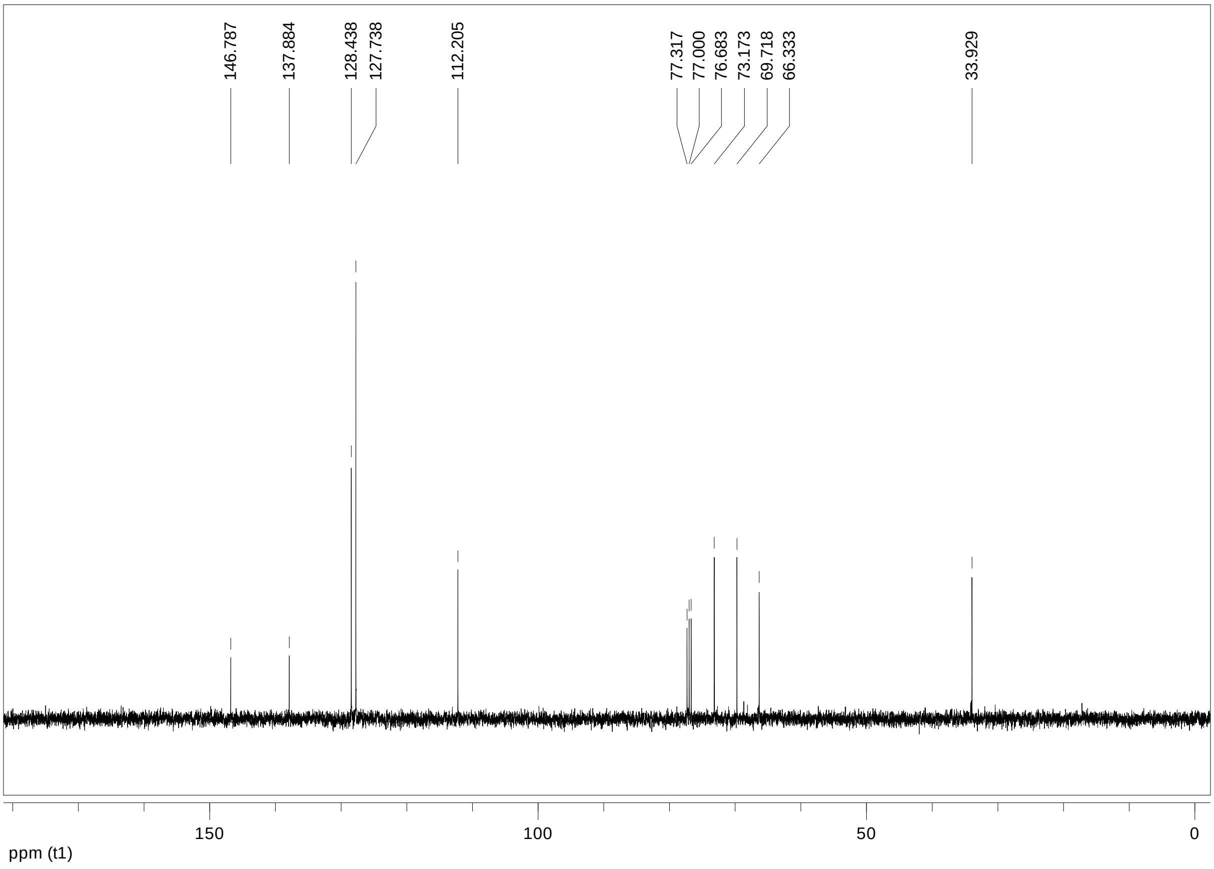
146.787
137.884
128.438
127.738
112.205
77.317
77.000
76.683
73.173
69.718
66.333
33.929
150
100
50
0
ppm (t1)

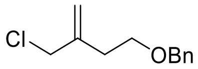

41

(((3-(Chloromethyl)but-3-en-1-yl)oxy)methyl)benzene (41):

***Finkelstein* reaction:** To a suspension of **39** (1.32 g, 6.87 mmol, 1.0 equiv) and LiCl (660 mg, 15.6 mmol, 2.3 equiv) in DMF (33 mL) was added dropwise at 0 °C 2,6-lutidine (1.81 mL, 15.5 mmol, 2.3 equiv) and MsCl (0.94 mL, 12.1 mmol, 1.8 equiv). The reaction mixture was stirred at 0 °C for 3 h and was alowed to warm to rt over 2 h. After quenching with water (70 mL) at 0 °C, the mixture was extracted with Et_2O (3 x 120 mL). The combined organic extracts were washed with water (50 mL) and brine (50 mL), dried over $MgSO_4$ and concentrated under reduced pressure. The residue was purified by column chromatography (hexane/Et_2O, 60:1 → 40:1 → 20:1) to provide **41** (1.27 g, 88%) as a yellow liquid.

***Appel* reaction:** To a solution of **39** (12.2 g, 63.5 mmol, 1.0 equiv) in acetonitrile (8.0 mL) was added at 0 °C triphenylphosphine (21.6 g, 82.4 mmol, 1.3 equiv) and tetrachloromethane (8.0 mL, 82.9 mmol, 1.3 equiv). After stirring for 2 h at rt, the reaction mixture was directly purified by column chromatography (hexane/Et_2O, 20:1) to provide **41** (12.7 g, 95%) as a colorless liquid.

$\mathbf{R}_f$ = 0.33 (hexane/Et_2O, 20:1).
^{1}H-NMR (400.1 MHz, $CDCl_3$): δ = 7.39-7.26 (m, 5H), 5.20 (s, 1H), 5.04 (q, *J* = 1.2 Hz, 1H), 4.53 (s, 2H), 4.08 (d, *J* = 0.9 Hz, 2H), 3.64 (t, *J* = 6.5 Hz, 2H), 2.51 (dt, *J* = 0.7, 6.5 Hz, 2H).
^{13}C-NMR (100.6 MHz, $CDCl_3$): δ = 142.6, 138.3, 128.4, 127.6 (2C), 115.9, 73.0, 68.5, 48.4, 33.3.
IR (neat, ν/cm^{-1}): 3087w, 3064w, 3031w, 2947w, 2859m, 1496w, 1453m, 1361m, 1258m, 1206w, 1100s, 1028m, 909m, 737s, 698s, 611w.
HRMS (EI): *m/z* calcd for $C_{12}H_{15}ClO$ $[M]^+$: 210.0806, found: 210.0812. (For ^{35}Cl).

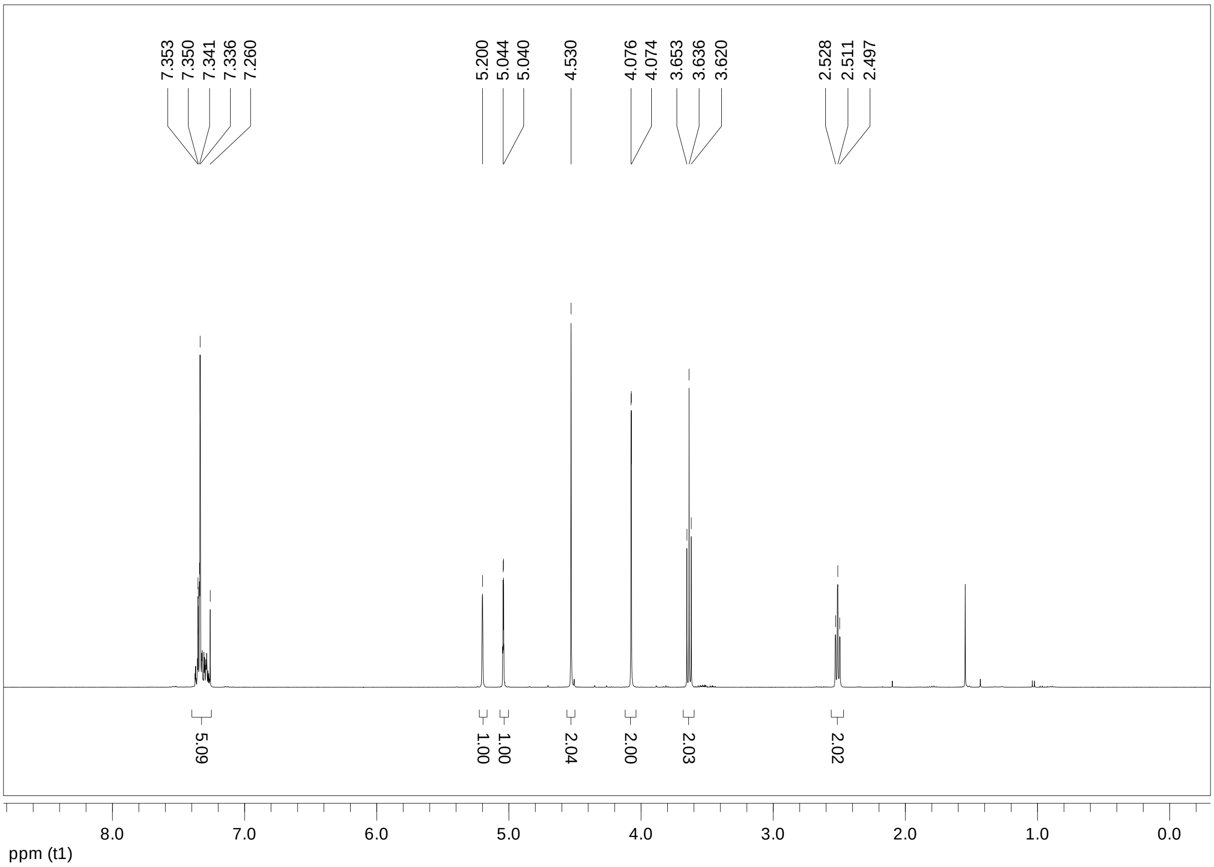
7.353
7.350
7.341
7.336
7.260
5.200
5.044
5.040
4.530
4.076
4.074
3.653
3.636
3.620
2.528
2.511
2.497
5.09
1.00
1.00
2.04
2.00
2.03
2.02
8.0
7.0
6.0
5.0
4.0
3.0
2.0
1.0
0.0
ppm (t1)

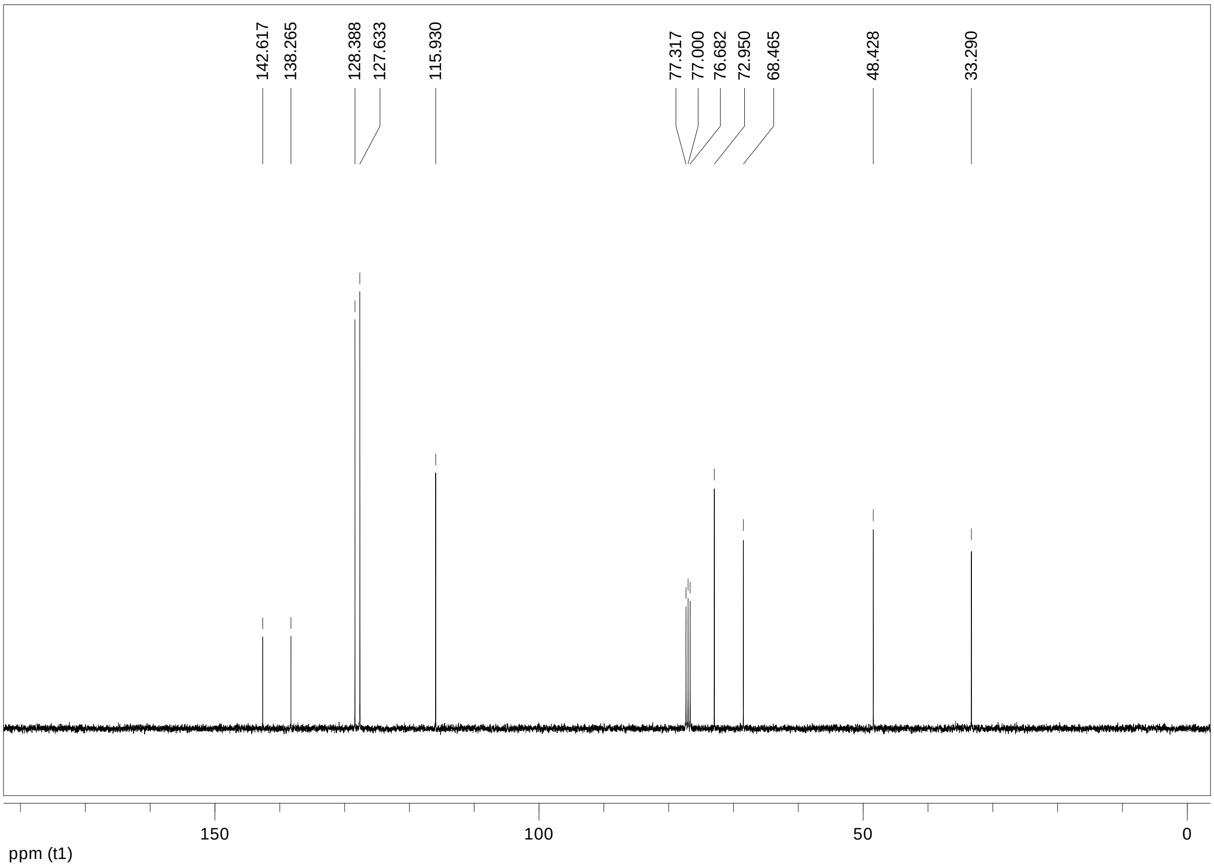
142.617
138.265
128.388
127.633
115.930
77.317
77.000
76.682
72.950
68.465
48.428
33.290
150
100
50
0
ppm (t1)

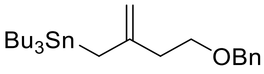

12b

(4-(Benzyloxy)-2-methylenebutyl)tributylstannane (12b): Mg (1.28 g, 52.8 mmol, 1.4 equiv) was dried with a heat gun under vacuum. After the flask had cooled to rt, THF (56 mL) was added followed by tributyltinchloride (10.0 mL, 36.9 mmol, 1.0 equiv) and a crystal of iodine. **41** (10.1 g, 47.9 mmol, 1.3 equiv) was added to this mixture with stirring at 0 °C and with external ultrasound irradiation over a period of 30 min (+3 mL THF for washing). After stirring for 1.5 h (ultrasound) at 0 °C, a suspension was formed. At this point a few drops of dibromoethane were added and the reaction mixture was allowed to warm to rt. The reaction mixture was heated to reflux with a heat gun, the salts precipitated immediately and some more THF was added (10 mL). After stirring over night at rt, the reaction was quenched at 0 °C with water (200mL) and diluted with ether (250 mL). The layers were separated and the aqueous layer was extracted once with ether (300 mL). The combined organic extracts were washed with brine (160 mL), dried over $MgSO_4$ and concentrated under reduced pressure to give the crude stannane **12b** (19.0 g. 80% purity).

^{1}H-NMR (400.1 MHz, $CDCl_3$): δ = 7.39-7.25 (m, 5H), 4.58 (m_c, 1H), 4.53 (m_c, 2H), 4.49 (m_c, 1H), 3.59 (t, *J* = 7.1 Hz, 2H), 2.27 (t, *J* = 7.1 Hz, 2H), 1.80 (s, 2H), 1.56-1.40 (m, 6H), 1.30 (m_c, 6H), 0.95-0.82 (m, 15H).
^{13}C-NMR (100.6 MHz, $CDCl_3$): δ = 146.9, 138.6, 128.3, 127.6, 127.5, 106.0, 72.9, 69.3, 38.2, 29.1, 27.3, 19.4, 13.7, 9.5.
^{119}Sn-NMR (186.5 MHz, $CDCl_3$): δ = –14.8.
HRMS (ESI): *m/z* calcd for $C_{24}H_{42}NaOSn$ [M + Na]$^+$: 489.2154; found: 489.2158.

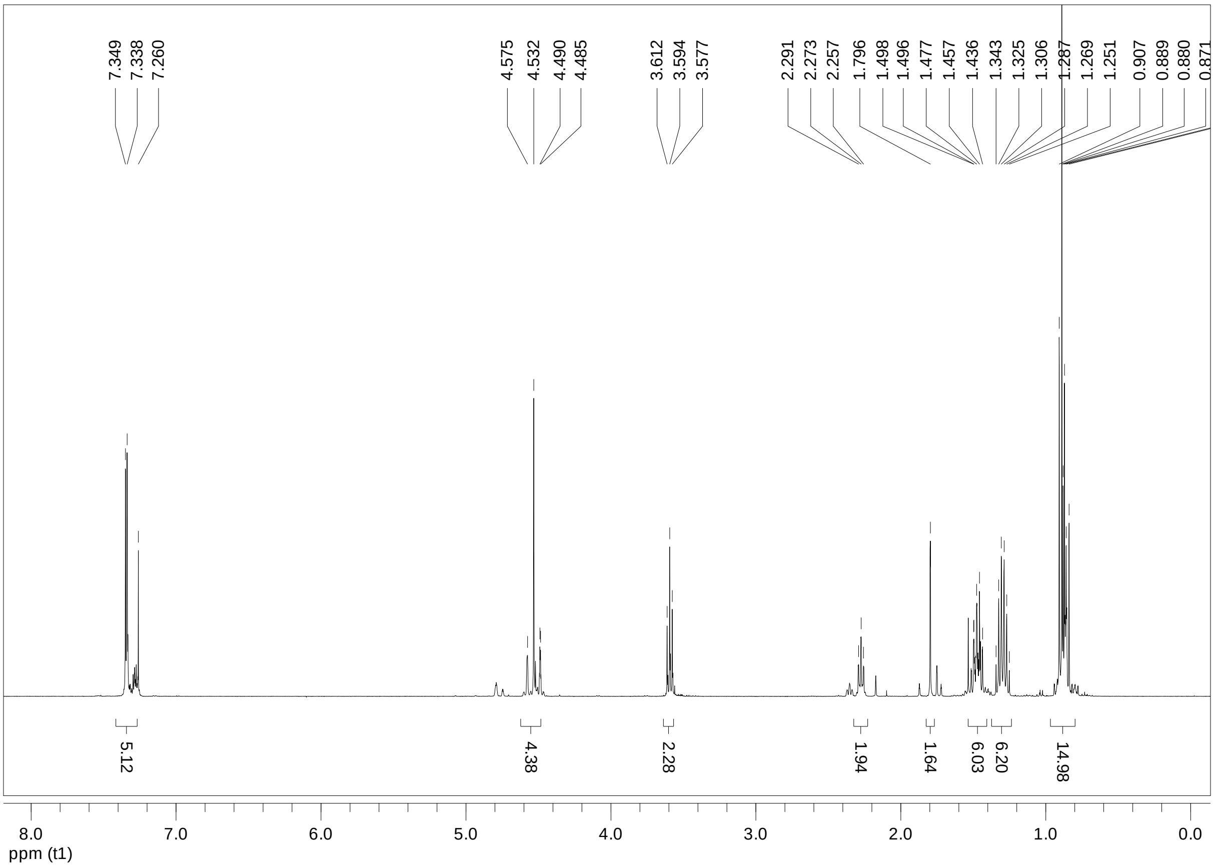
7.349
7.338
7.260
4.575
4.532
4.490
4.485
3.612
3.594
3.577
2.291
2.273
2.257
1.796
1.498
1.496
1.477
1.457
1.436
1.343
1.325
1.306
1.287
1.269
1.251
0.907
0.889
0.880
0.871
5.12
4.38
2.28
1.94
1.64
6.03
6.20
14.98
8.0
7.0
6.0
5.0
4.0
3.0
2.0
1.0
0.0
ppm (t1)

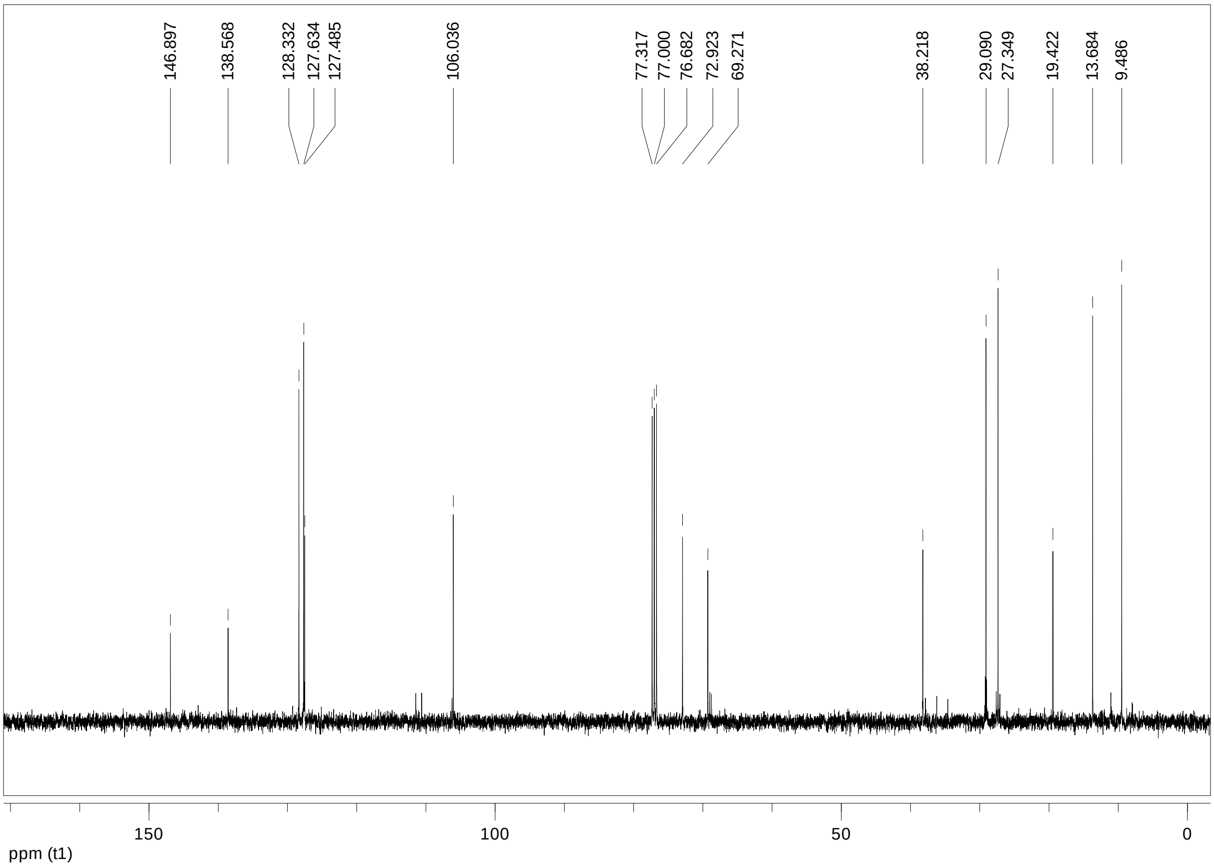
146.897
138.568
128.332
127.634
127.485
106.036
77.317
77.000
76.682
72.923
69.271
38.218
29.090
27.349
19.422
13.684
9.486
150
100
50
0
ppm (t1)

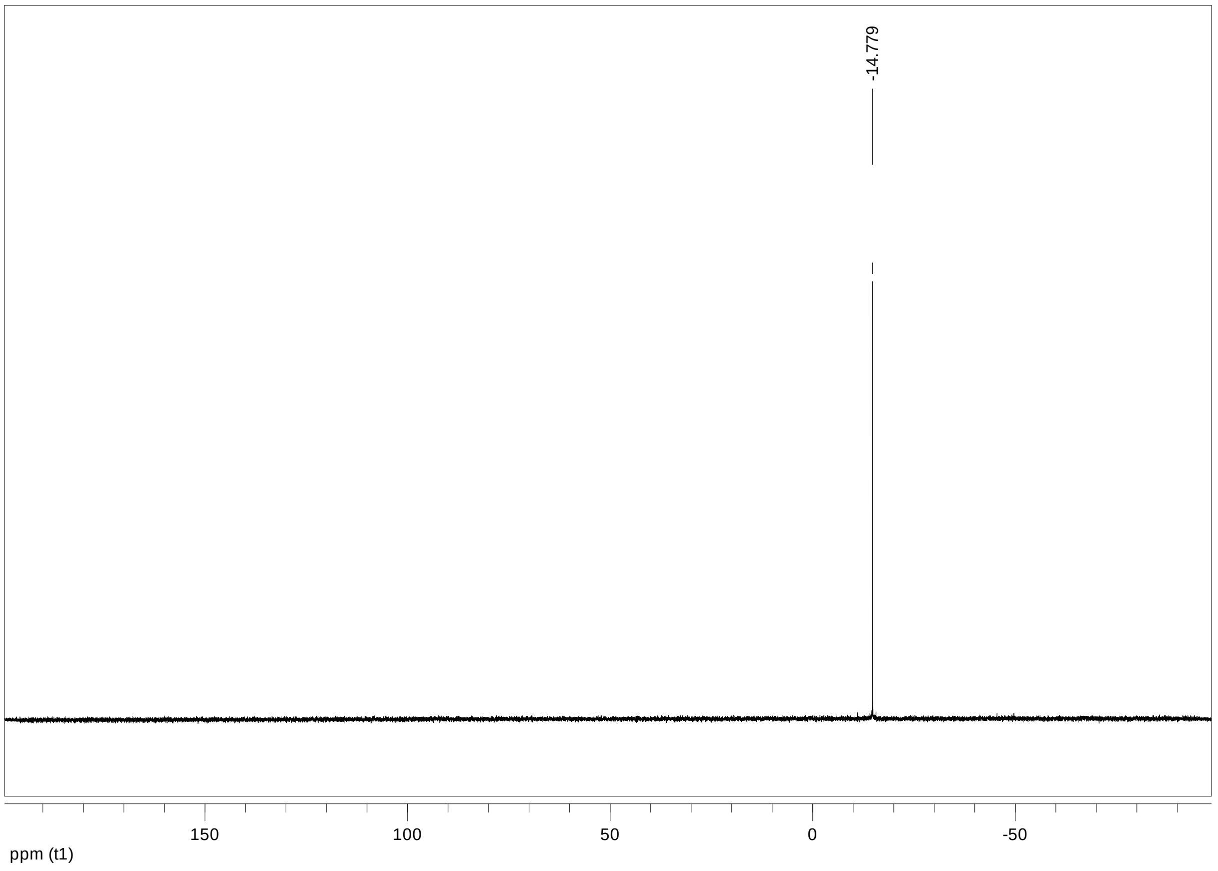
-14.779
150
100
50
0
-50
ppm (t1)

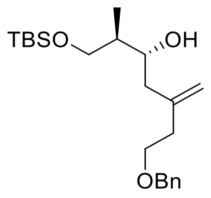

47

(2*R*,3*R*)-7-(benzyloxy)-1-(*tert*-butyldimethylsilyloxy)-2-methyl-5-methyleneheptan-3-ol (47): The reaction was performed in two parallel batches. A 250 mL Schlenk flask was charged with (*R,R*)-*N,N'*-bis-*para*-toluenesulfonyl-1,2-diamino-1,2-diphenylethane (**48**) (4.98 g, 9.57 mmol, 1.03 equiv) and heated to 90 °C under high vacuum (0.03 mbar). After 16 h, the resulting colorless solid was cooled to rt and CH_2Cl_2 (65 mL) was added. This solution was cooled to 0 °C and treated with BBr_3 (9.6 mL, 9.60 mmol, 1.04 equiv, 1.0 M in CH_2Cl_2). The resulting brownish solution was stirred at 0 °C for 10 min, warmed to rt, and stirred for 1 h. The solvent and HBr were removed carefully under reduced pressure (Schlenk). The resulting solid was dried under high vacuum for 1.5 h and was then dissolved in CH_2Cl_2 (87 mL), the solution was cooled to 0 °C, and **12b** (5.00 g, 10.7 mmol, 1.16 equiv) was added dropwise. After stirring for 16 h at rt, a solution of **11a** (1.87 g, 9.25 mmol, 1.00 equiv, crude) in CH_2Cl_2 (20 mL +3 mL for washing) was added dropwise at –78 °C over 1 h by means of a mechanical syringe pump. The reaction mixture was stirred for 2 h at –78 °C; then pH 7 buffer (50 mL) was added and the mixture was allowed to warm to rt. At this point both batches of material were combined. The layers were separated and the aqueous phase was extracted with CH_2Cl_2 (3 x 60 mL). The combined organic extracts were washed with brine, dried over $MgSO_4$ and concentrated under reduced pressure. The resulting solid was dissolved in Et_2O and filtered to recover the ligand (9.36 g, 94% recovery). The filtrate was concentrated and the residue was purified by column chromatography (hexane/Et_2O, 10:1 → 5:1 → 4:1) to provide **47** (5.16 g, 74%, *dr* 91:9) as a slightly yellowish oil.

R_f = 0.19 (hexane/ Et_2O, 5:1)

$[\alpha]_D^{24}$ = –0.35° [*c* = 0.511, $CHCl_3$]

^{1}H-NMR (400.1 MHz, $CDCl_3$): δ = 7.37-7.24 (m, 5H), 4.91 (dd, *J* = 1.4, 3.0 Hz, 2H), 4.52 (s, 2H), 3.97 (ddd, *J* = 2.8, 5.2, 11.3 Hz, 1H), 3.68 (ddd, *J* = 4.9, 9.8, 15.3 Hz, 2H), 3.61 (t, *J* = 6.8 Hz, 2H), 2.88 (d, *J* = 3.0 Hz, 1H), 2.40 (dt, *J* = 2.8, 6.9 Hz, 2H), 2.28 -2.13 (m, 2H), 1.71 (m_c, 1H), 0.92 (d, *J* = 7.0 Hz, 3H), 0.90 (s, 9H), 0.07 (s, 6H).

^{13}C-NMR (100.6 MHz, $CDCl_3$): δ = 144.0, 138.4, 128.3, 127.7, 127.5, 113.3, 72.9, 71.6, 68.9, 67.8, 41.6, 39.0, 35.8, 25.9, 18.2, 10.3, −5.6.

IR (neat, ν/cm^{-1}): 3483br, 2954m, 2930m, 2857m, 1467w, 1362w, 1254m, 1092s, 1025m, 836s, 776s, 738m, 697m, 669w.

HRMS (ESI): *m/z* calcd for $C_{22}H_{39}O_3Si$ $[M + H]^+$: 379.2663; found: 379.2662.

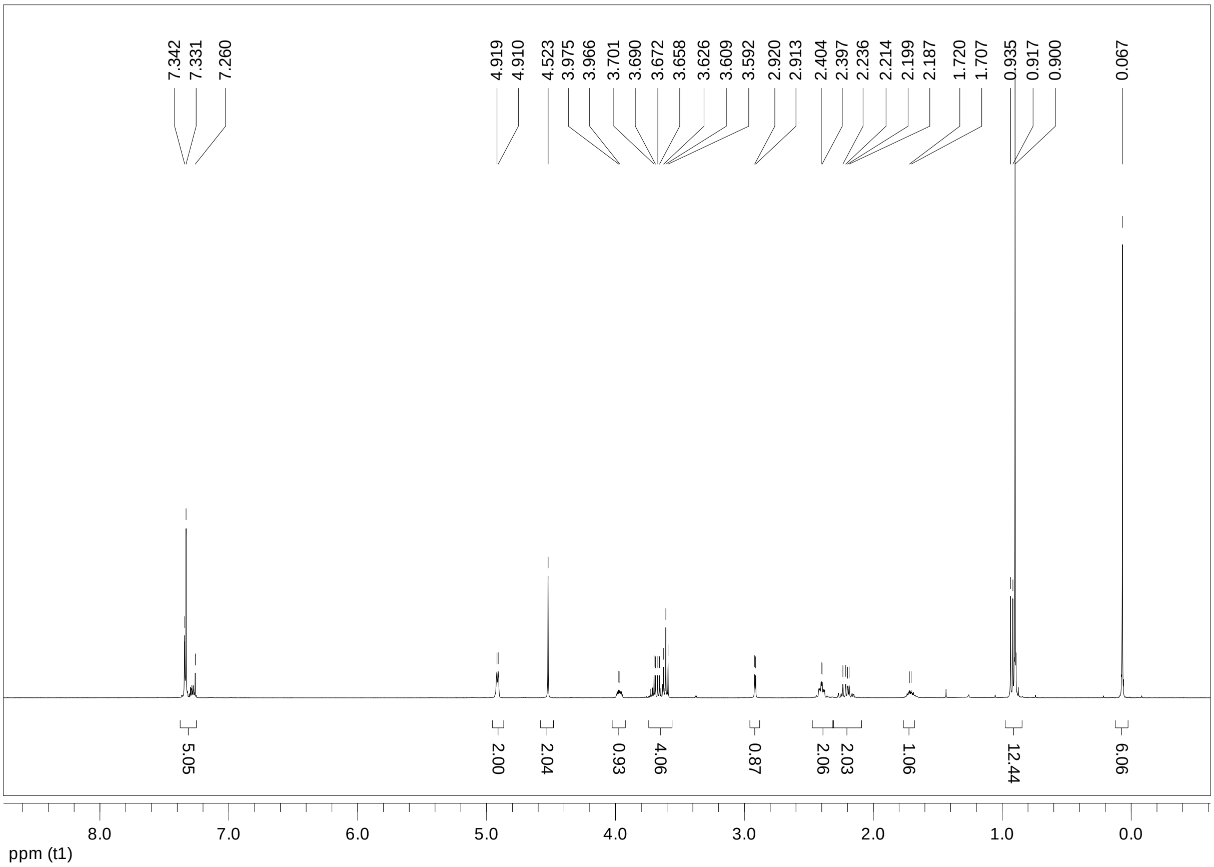
7.342
7.331
7.260
4.919
4.910
4.523
3.975
3.966
3.701
3.690
3.672
3.658
3.626
3.609
3.592
2.920
2.913
2.404
2.397
2.236
2.214
2.199
2.187
1.720
1.707
0.935
0.917
0.900
0.067
5.05
2.00
2.04
0.93
4.06
0.87
2.06
2.03
1.06
12.44
6.06
8.0
7.0
6.0
5.0
4.0
3.0
2.0
1.0
0.0
ppm (t1)

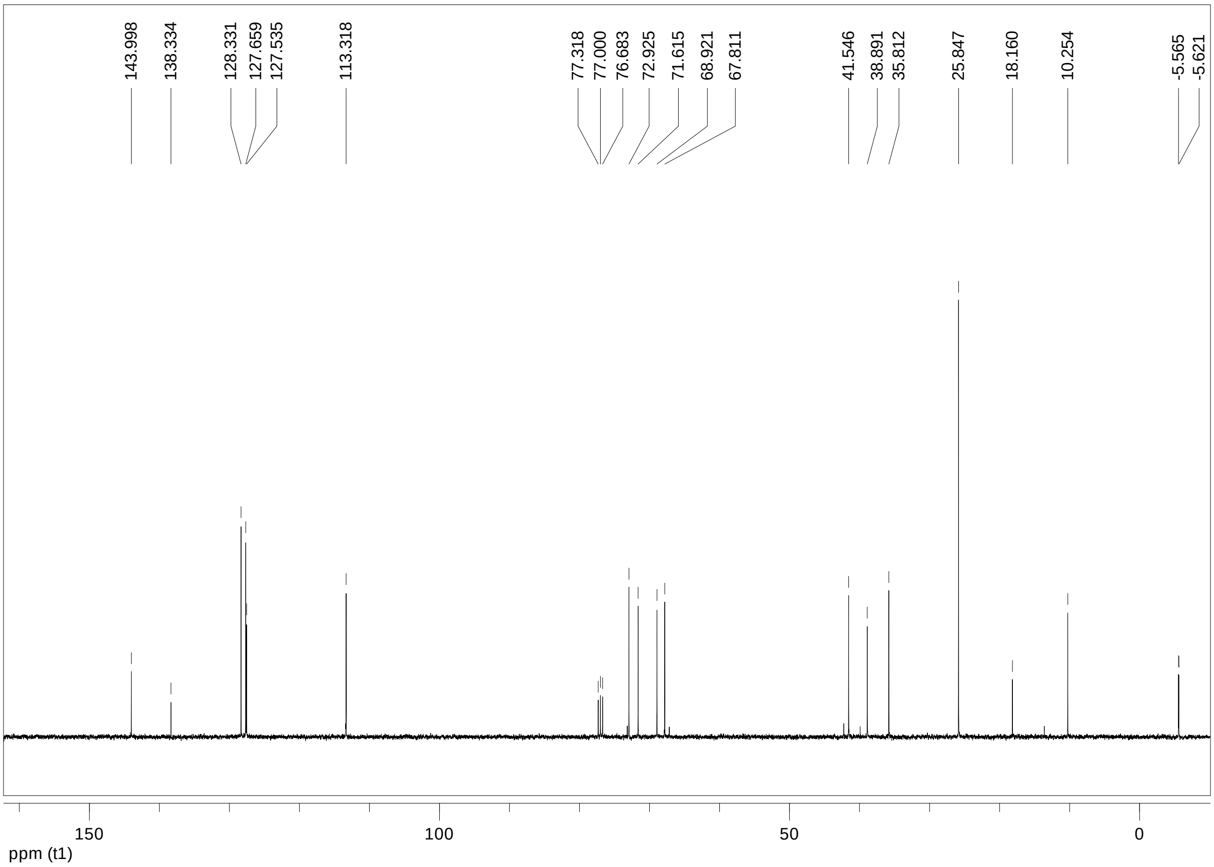
143.998
138.334
128.331
127.659
127.535
113.318
77.318
77.000
76.683
72.925
71.615
68.921
67.811
41.546
38.891
35.812
25.847
18.160
10.254
-5.565
-5.621
150
100
50
0
ppm (t1)

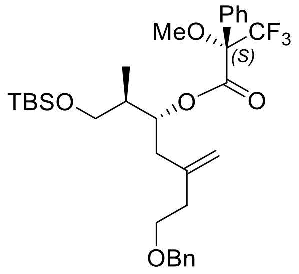

S12

***S*-Mosher ester S12:** To a solution of **47** (16.1 mg, 42.5 µmol, 1.0 equiv) in pyridine (0.1 mL, 1.24 mmol, 29 equiv) was added DMAP (15.5 mg, 127 µmol, 3.0 equiv) and (*R*)-(–)-MTPA-Cl (29 µL, 155 µmol, 3.7 equiv). The reaction mixture was stirred for 16 h at rt and quenched with water (1 mL). The aqueous phase was extracted with CH_2Cl_2 (3 x 6 mL) and the combined organic extracts were dried over $MgSO_4$, filtered and the solvent was removed under reduced pressure. The crude product was purified by column chromatography (hexane/Et_2O, 10:1) to afford **S12** as a colorless oil (12.4 mg, 49%, 77% brsm).

R_f = 0.50 (hexane/ Et_2O, 5:1)

$[\alpha]_D^{24}$ = −26.7° [*c* = 0.570, $CHCl_3$]

^{1}H-NMR (400.1 MHz, $CDCl_3$): δ = 7.59-7.48 (m, 2H), 7.44-7.22 (m, 8H), 5.51 (dt, *J* = 2.6, 7.2 Hz, 1H), 4.90 (d, *J* = 2.8 Hz, 2H), 4.52 (s, 2H), 3.62 (t, *J* = 6.6 Hz, 2H), 3.53 (s, 3H), 3.32 (d, *J* = 2.6 Hz, 1H), 3.31 (d, *J* = 3.6 Hz, 1H), 2.51 (dd, *J* = 7.2, 14.1 Hz, 1H), 2.42 (t, *J* = 6.6 Hz, 2H), 2.38 (m_c, 1H), 1.88 (m_c, 1H), 0.88 (s, 9H), 0.84 (d, *J* = 6.9 Hz, 3H), 0.00 (s, 6H).

^{13}C-NMR (100.6 MHz, $CDCl_3$): δ = 165.9, 142.1, 138.4, 132.5, 129.5, 128.3, 128.2, 127.6, 127.5, 127.5, 123.4 (q, *J* = 288 Hz), 114.4, 74.6, 72.9, 68.5, 64.3, 55.4, 38.3, 38.3, 35.4, 25.8, 18.1, 10.1, -5.5. (quaternary carbon not visible)

IR (neat, ν/cm^{-1}): 3066w, 2953m, 2930m, 2858m, 1747s, 1472w, 1257s, 1169s, 1104s, 1020m, 912w, 837s, 775m, 722m, 697m.

HRMS (ESI): *m/z* calcd for $C_{32}H_{49}F_3NO_5Si$ [M + NH_4]$^+$: 612.3327; found: 612.3313.

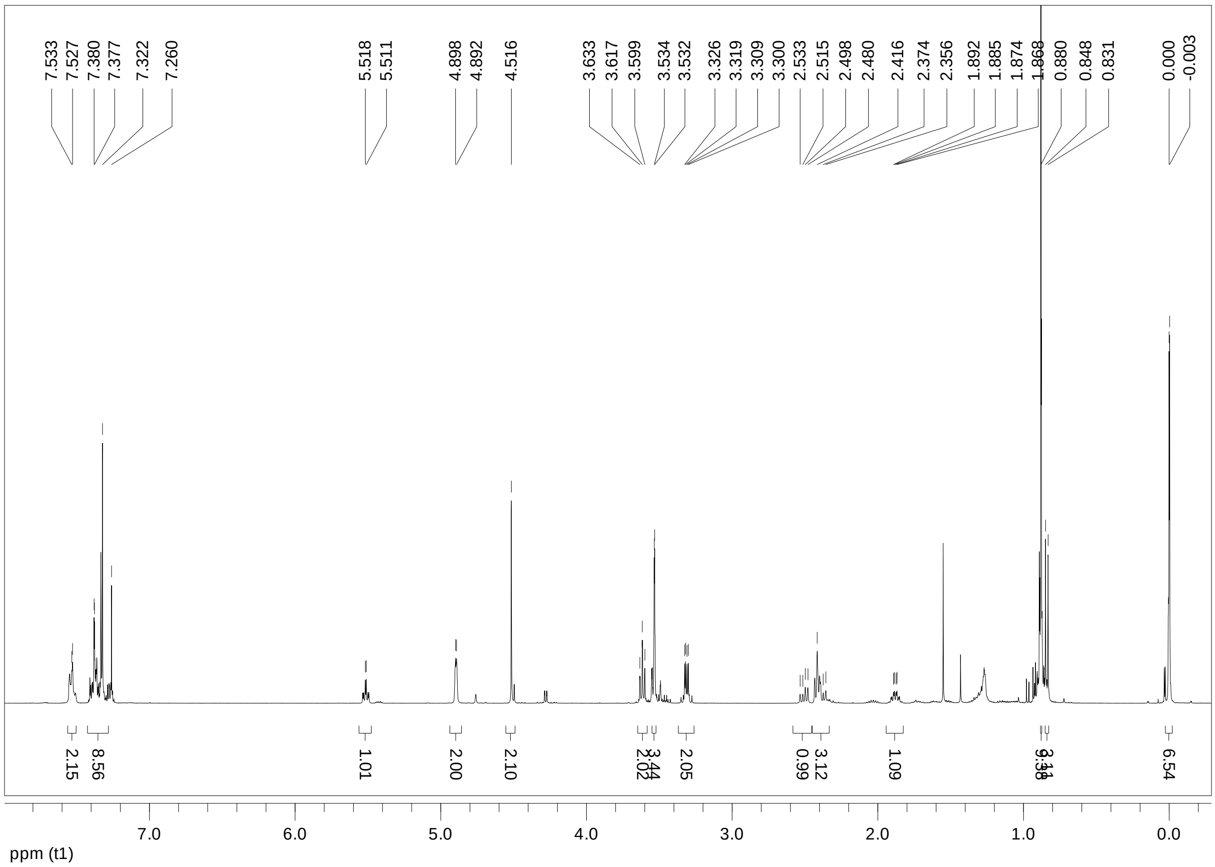
7.533
7.527
7.380
7.377
7.322
7.260
5.518
5.511
4.898
4.892
4.516
3.633
3.617
3.599
3.534
3.532
3.326
3.319
3.309
3.300
2.533
2.515
2.498
2.480
2.416
2.374
2.356
1.892
1.885
1.874
1.868
0.880
0.848
0.831
0.000
-0.003
2.15
8.56
1.01
2.00
2.10
2.02
3.44
2.05
0.99
3.12
1.09
6.54
7.0
6.0
5.0
4.0
3.0
2.0
1.0
0.0
ppm (t1)

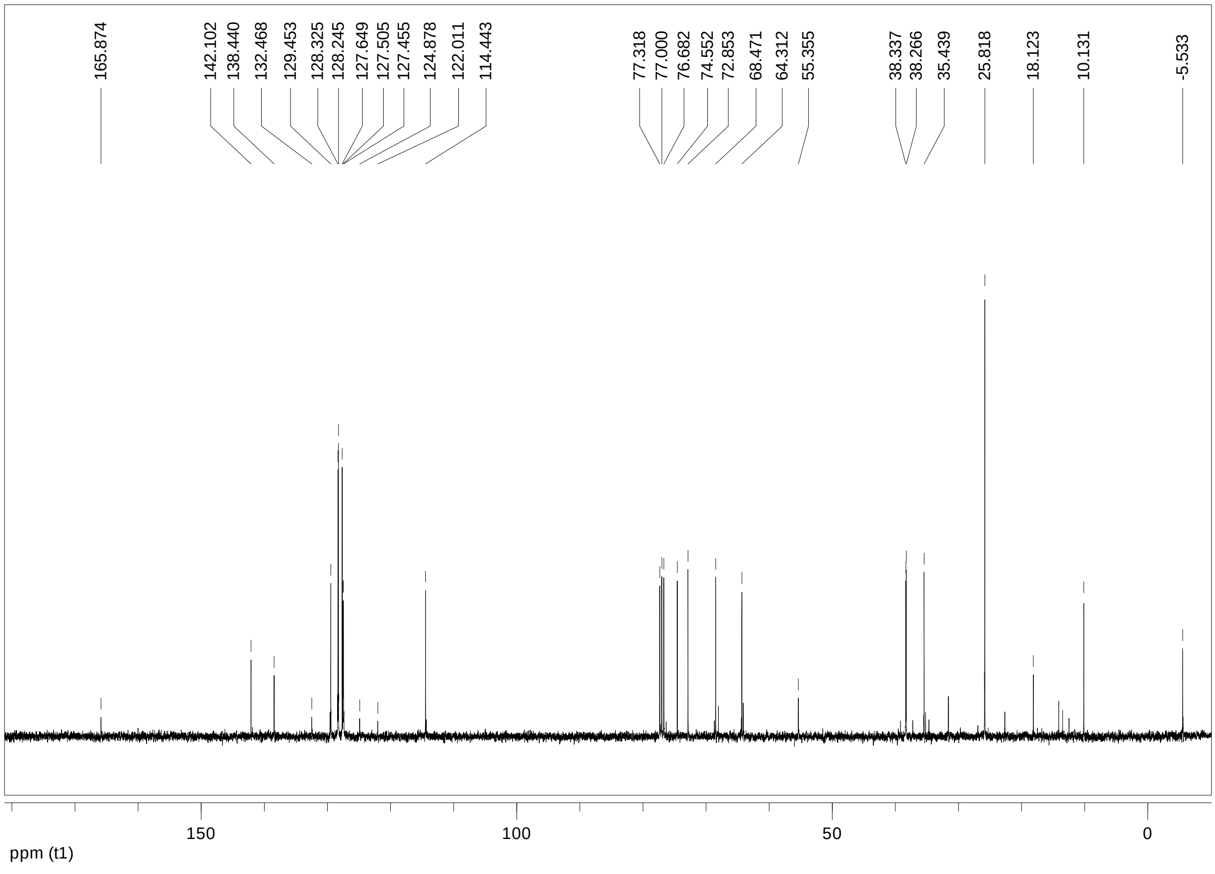
165.874
142.102
138.440
132.468
129.453
128.325
128.245
127.649
127.505
127.455
124.878
122.011
114.443
77.318
77.000
76.682
74.552
72.853
68.471
64.312
55.355
38.337
38.266
35.439
25.818
18.123
10.131
-5.533
150
100
50
0
ppm (t1)

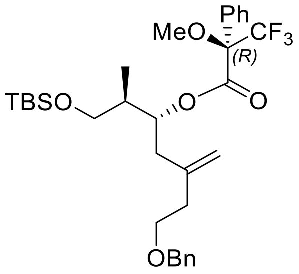

S13

***R*-Mosher ester S13:** To a solution of **47** (17.3 mg, 45.7 µmol, 1.0 equiv) in pyridine (0.1 mL, 1.24 mmol, 27 equiv) was added DMAP (17.5 mg, 143 µmol, 3.1 equiv) and (*S*)-(−)-MTPA-Cl (29 µL, 155 µmol, 3.4 equiv). The reaction mixture was stirred for 16 h at rt and quenched with water (1 mL). The aqueous phase was extracted with CH_2Cl_2 (3 x 6 mL) and the combined organic extracts were dried over $MgSO_4$, filtered and the solvent was removed under reduced pressure. The crude product was purified by column chromatography (hexane/Et_2O, 10:1) to afford **S13** as a colorless oil (22.7 mg, 84%).

R_f = 0.50 (hexane/ Et_2O, 5:1)
$[\alpha]_D^{24}$ = +4.4° [*c* = 1.07, $CHCl_3$]
^{1}H-NMR (400.1 MHz, $CDCl_3$): δ = 7.56-7.48 (m, 2H), 7.44-7.25 (m, 8H), 5.51 (dt, *J* = 2.7, 7.1 Hz, 1H), 4.84 (s, 2H), 4.52 (s, 2H), 3.61 (t, *J* = 6.6 Hz, 2H), 3.49 (s, 3H), 3.43 (s, 1H), 3.41 (d, *J* = 1.8 Hz, 1H), 2.46 (dd, *J* = 7.4, 14.1 Hz, 1H), 2.41 (t, *J* = 6.7 Hz, 2H), 2.32 (dd, *J* = 6.9, 14.1 Hz, 1H), 1.91 (m_c, 1H), 0.89 (s, 9H), 0.88 (d, *J* = 4.6 Hz, 3H), 0.02 (s, 6H).
^{13}C-NMR (100.6 MHz, $CDCl_3$): δ = 166.0, 141.9, 138.5, 132.3, 129.5, 128.3, 128.3, 127.7, 127.6, 127.5, 123.4 (q, *J* = 288 Hz), 114.5, 74.5, 72.9, 68.5, 64.5, 55.2, 38.3, 35.4, 25.8, 18.1, 10.3, -5.5. (quaternary carbon not visible)
IR (neat, ν/cm^{-1}): 3068w, 2953m, 2929m, 2857m, 1744s, 1471w, 1256s, 1169s, 1104s, 1017m, 903w, 837s, 777m, 721m, 698m.
HRMS (ESI): *m/z* calcd for $C_{32}H_{49}F_3NO_5Si$ $[M + NH_4]^+$: 612.3327; found: 612.3320.

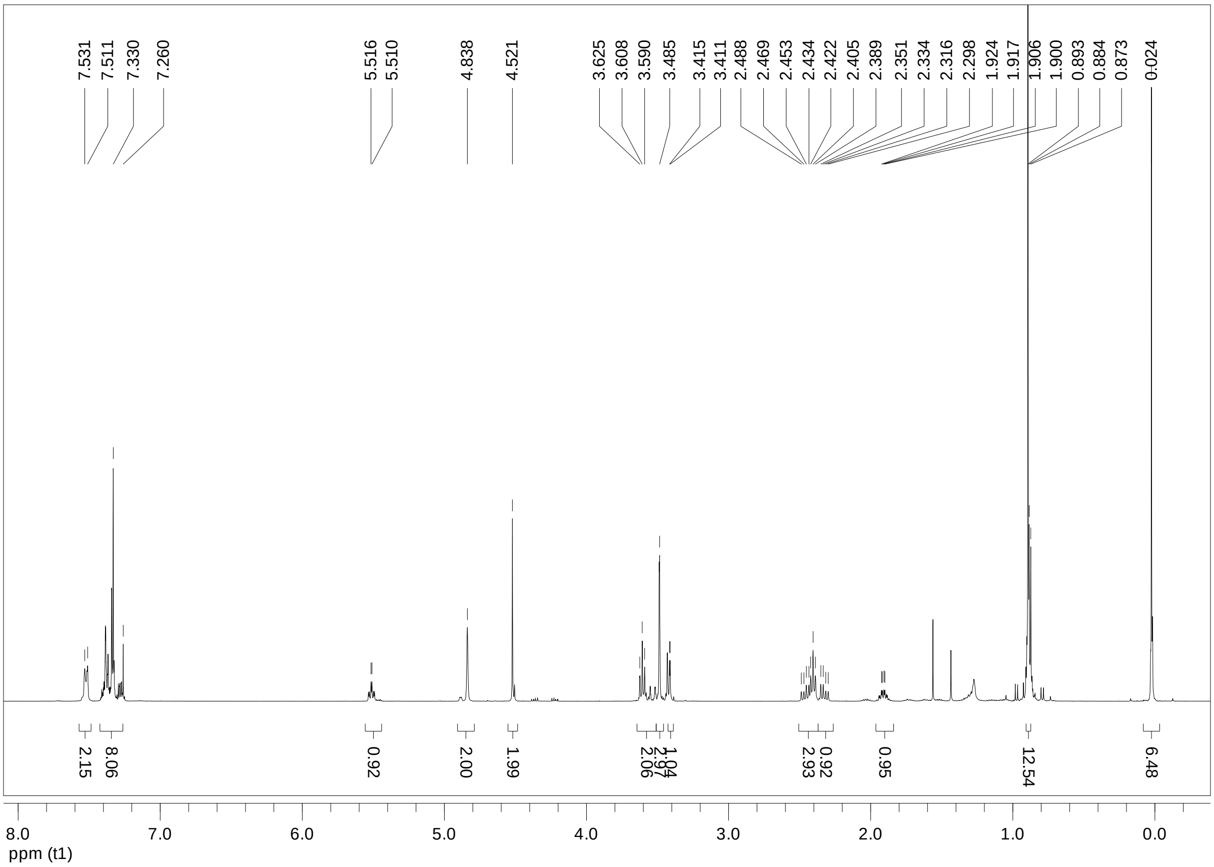
7.531
7.511
7.330
7.260
5.516
5.510
4.838
4.521
3.625
3.608
3.590
3.485
3.415
3.411
2.488
2.469
2.453
2.434
2.422
2.405
2.389
2.351
2.334
2.316
2.298
1.924
1.917
1.906
1.900
0.893
0.884
0.873
0.024
2.15
8.06
0.92
2.00
1.99
2.06
2.97
1.04
2.93
0.92
0.95
12.54
6.48
8.0
7.0
6.0
5.0
4.0
3.0
2.0
1.0
0.0
ppm (t1)

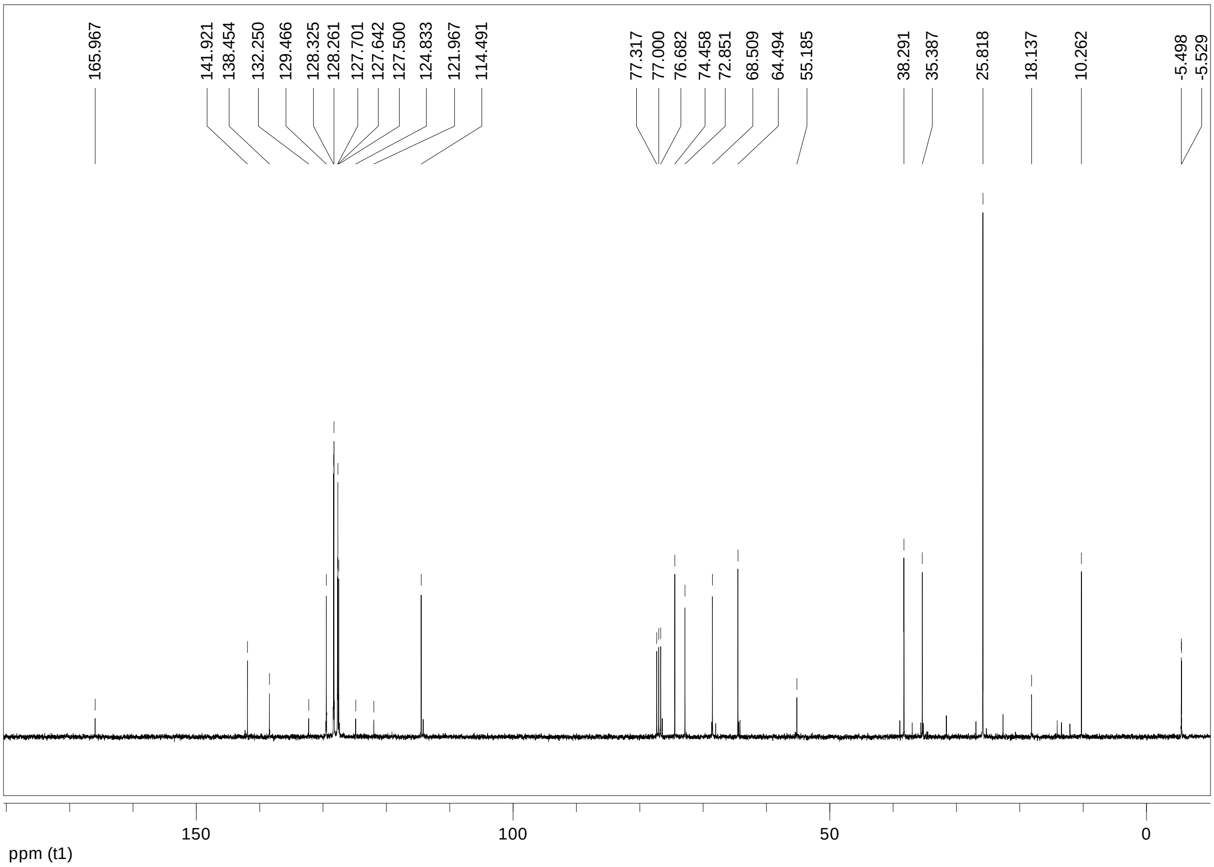
165.967
141.921
138.454
132.250
129.466
128.325
128.261
127.701
127.642
127.500
124.833
121.967
114.491
77.317
77.000
76.682
74.458
72.851
68.509
64.494
55.185
38.291
35.387
25.818
18.137
10.262
-5.498
-5.529
150
100
50
0
ppm (t1)

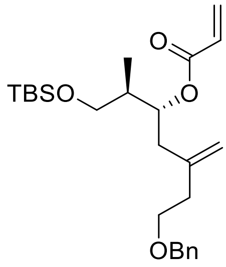

52

(2*R*,3*R*)-7-(Benzyloxy)-1-(*tert*-butyldimethylsilyloxy)-2-methyl-5-methyleneheptan-3-yl acrylate (52): To a solution of **47** (5.16 g, 13.6 mmol, 1.0 equiv) in CH_2Cl_2 (115 mL) was added at −40 °C DIPEA (10.1 mL, 58.6 mmol, 4.3 equiv) followed by dropwise addition of acryloylchloride (4.44 mL, 54.5 mmol, 4.0 equiv). The yellow reaction mixture was stirred at −50 °C for 1.5 h and then transferred into a vigorously stirred solution of sat. $NaHCO_3$ (100 mL). CH_2Cl_2 (70 mL) was then added and the mixture was stirred for 30 min; during this period the pH of the aqueous phase was controlled (pH 6-7) to prevent loss of TBS by acid formation (if necessary, some solid $NaHCO_3$ was added). The layers were separated and the aqueous phase was extracted with CH_2Cl_2 (2 x 70 mL). The combined organic extracts were washed with brine, dried over $MgSO_4$ and concentrated under reduced pressure. The residue was purified by column chromatography (hexane/Et_2O, 30:1 → 7:1) to afford **52** (5.02 g, 85%) as a colorless oil.

R_f = 0.51 (hexane/ Et_2O, 5:1)

$[\alpha]_D^{24}$ = −5.0° [*c* = 0.533, $CHCl_3$]

^{1}H-NMR (400.1 MHz, $CDCl_3$): δ = 7.36-7.24 (m, 5H), 6.35 (dd, *J* = 1.5, 17.3 Hz, 1H), 6.08 (dd, *J* = 10.4, 17.3 Hz, 1H), 5.78 (dd, *J* = 1.6, 10.4 Hz, 1H), 5.27 (ddd, *J* = 3.6, 6.2, 7.8 Hz, 1H), 4.85 (s, 2H), 4.52 (s, 2H), 3.61 (t, *J* = 6.8 Hz, 2H), 3.46 (dq, *J* = 6.6, 9.9 Hz, 2H), 2.45-2.28 (m, 4H), 1.89 (m_c, 1H), 0.93 (d, *J* = 6.9 Hz, 3H), 0.89 (s, 9H), 0.01 (s, 6H).

^{13}C-NMR (100.6 MHz, $CDCl_3$): δ = 165.6, 142.7, 138.5, 130.1, 128.9, 128.3, 127.6, 127.4, 113.9, 72.8, 72.2, 68.7, 64.9, 38.8, 38.7, 35.7, 25.9, 18.2, 11.0, −5.5.

IR (neat, ν/cm^{-1}): 2953m, 2930m, 2857m, 1723s, 1459w, 1405m, 1268m, 1192s, 1095s, 1045m, 984m, 836s, 775s, 736m, 698m.

HRMS (ESI): *m/z* calcd for $C_{25}H_{44}NO_4Si$ $[M + NH_4]^+$: 450.3034; found: 450.3048.

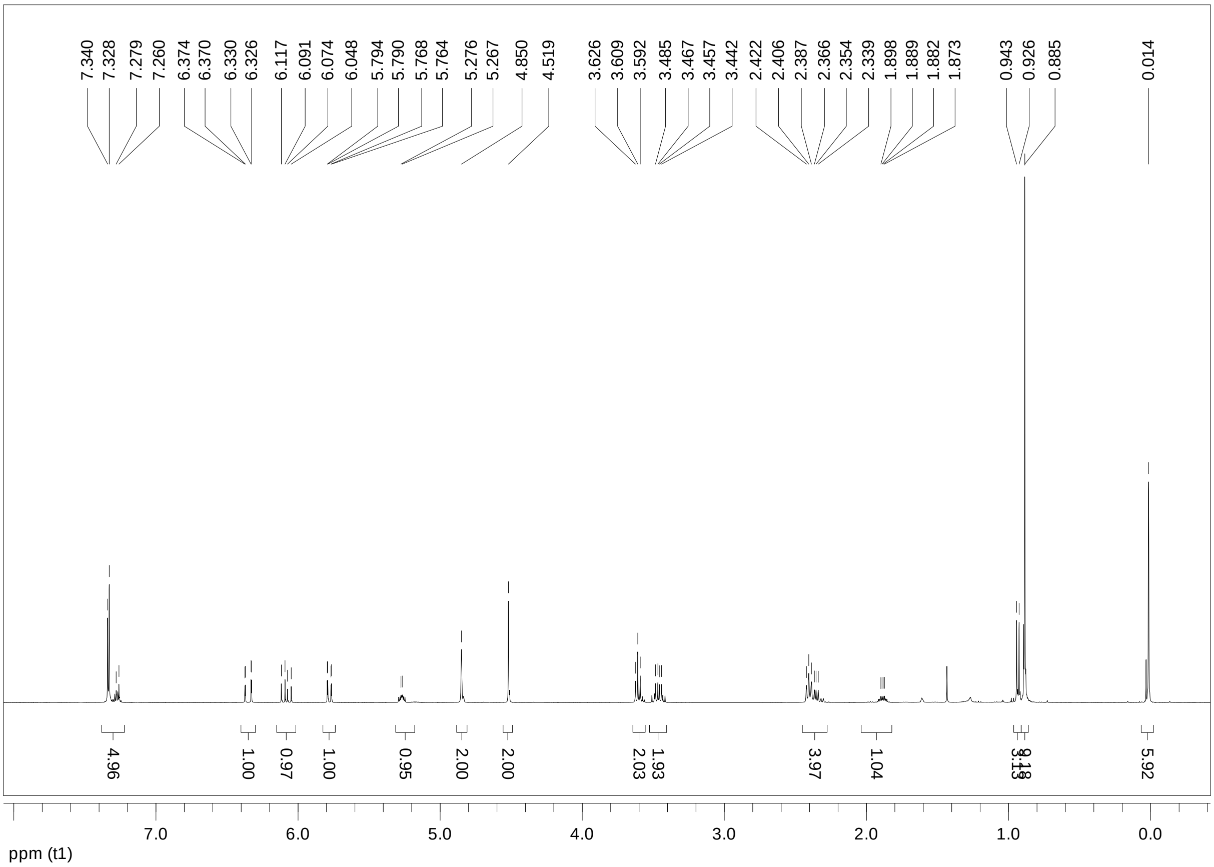
7.340 7.328 7.279 7.260 6.374 6.370 6.330 6.326 6.117 6.091 6.074 6.048 5.794 5.790 5.768 5.764 5.276 5.267 4.850 4.519 3.626 3.609 3.592 3.485 3.467 3.457 3.442 2.422 2.406 2.387 2.366 2.354 2.339 1.898 1.889 1.882 1.873 0.943 0.926 0.885 0.014
4.96 1.00 0.97 1.00 0.95 2.00 2.00 2.03 1.93 3.97 1.04 3.15 9.18 5.92
7.0 6.0 5.0 4.0 3.0 2.0 1.0 0.0
ppm (t1)

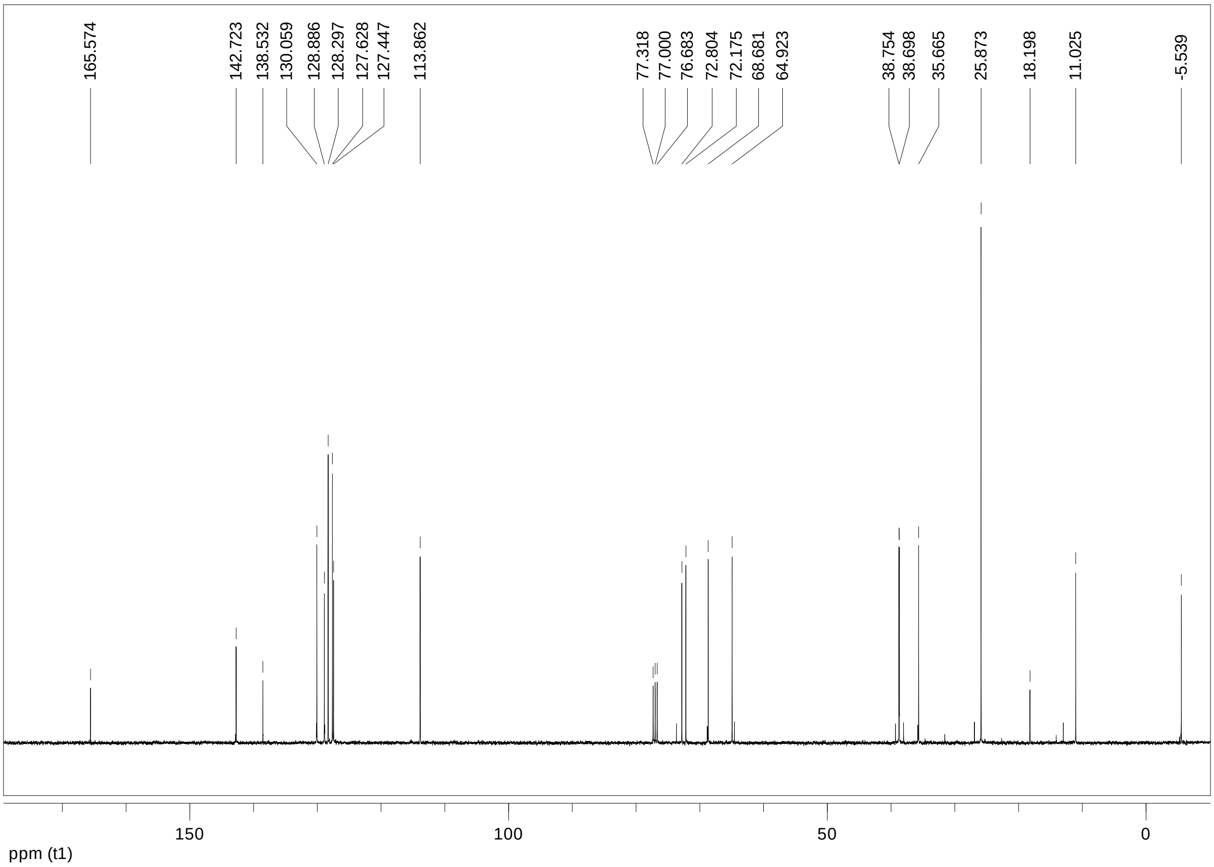
165.574 142.723 138.532 130.059 128.886 128.297 127.628 127.447 113.862 77.318 77.000 76.683 72.804 72.175 68.681 64.923 38.754 38.698 35.665 25.873 18.198 11.025 -5.539
150 100 50 0
ppm (t1)

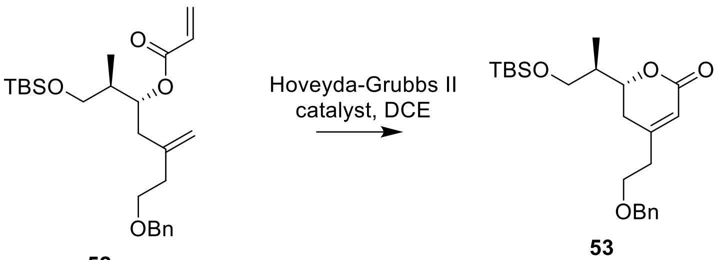

52 **53**

(*R*)-4-(2-(Benzyloxy)ethyl)-6-((*R*)-1-(*tert*-butyldimethylsilyloxy)propan-2-yl)-5,6-dihydro-2H-pyran-2-one (53): To a solution of **52** (44.9 mg, 0.10 mmol, 1.0 equiv) in DCE (1.0 mL) was added *Hoveyda-Grubbs'* 2nd generation catalyst (3.9 mg, 6.0 mol%). After refluxing for 24 h, a solution of *Hoveyda-Grubbs'* 2nd generation catalyst (2.4 mg, 3.7 mol%) in DCE (0.4 mL) was added. After refluxing for 17 h, the reaction mixture was concentrated under reduced pressure and the residue was purified by column chromatography (hexane/EtOAc, 6:1 → 3:1) to afford **53** (37.4 mg, 89%) as a greenish oil. In addition, 2.4 mg of the catalyst were recovered (38%).

R*f* = 0.37 (hexane/EtOAc, 3:1)

$[\alpha]_D^{24}$ = +35.8° [*c* = 0.667, $CHCl_3$].

^{1}H-NMR (400.1 MHz, $CDCl_3$): δ = 7.38-7.26 (m, 5H), 5.85 (m_c, 1H), 4.51 (s, 2H), 4.47 (td, *J* = 3.8, 12.8 Hz, 1H), 3.67-3.55 (m, 2H), 3.64 (t, *J* = 6.2 Hz, 2H), 2.57-2.43 (m, 3H), 2.16 (dd, *J* = 3.5, 17.8 Hz, 1H), 1.86 (m_c, 1H), 0.99 (d, *J* = 6.9 Hz, 3H), 0.88 (s, 9H), 0.04 (s, 6H).

^{13}C-NMR (100.6 MHz, $CDCl_3$): δ = 165.4, 158.7, 137.7, 128.5, 127.8, 127.7, 116.7, 77.6, 73.2, 67.0, 64.2, 39.5, 36.8, 31.5, 25.9, 18.2, 11.5, −5.5.

IR (neat, ν/cm^{-1}): 2953m, 2929m, 2857m, 1718s, 1459w, 1389w, 1249s, 1097s, 1026m, 836s, 777s, 739m, 699m.

HRMS (ESI): *m/z* calcd for $C_{23}H_{37}O_4Si$ $[M + H]^+$: 405.2456; found: 405.2461.

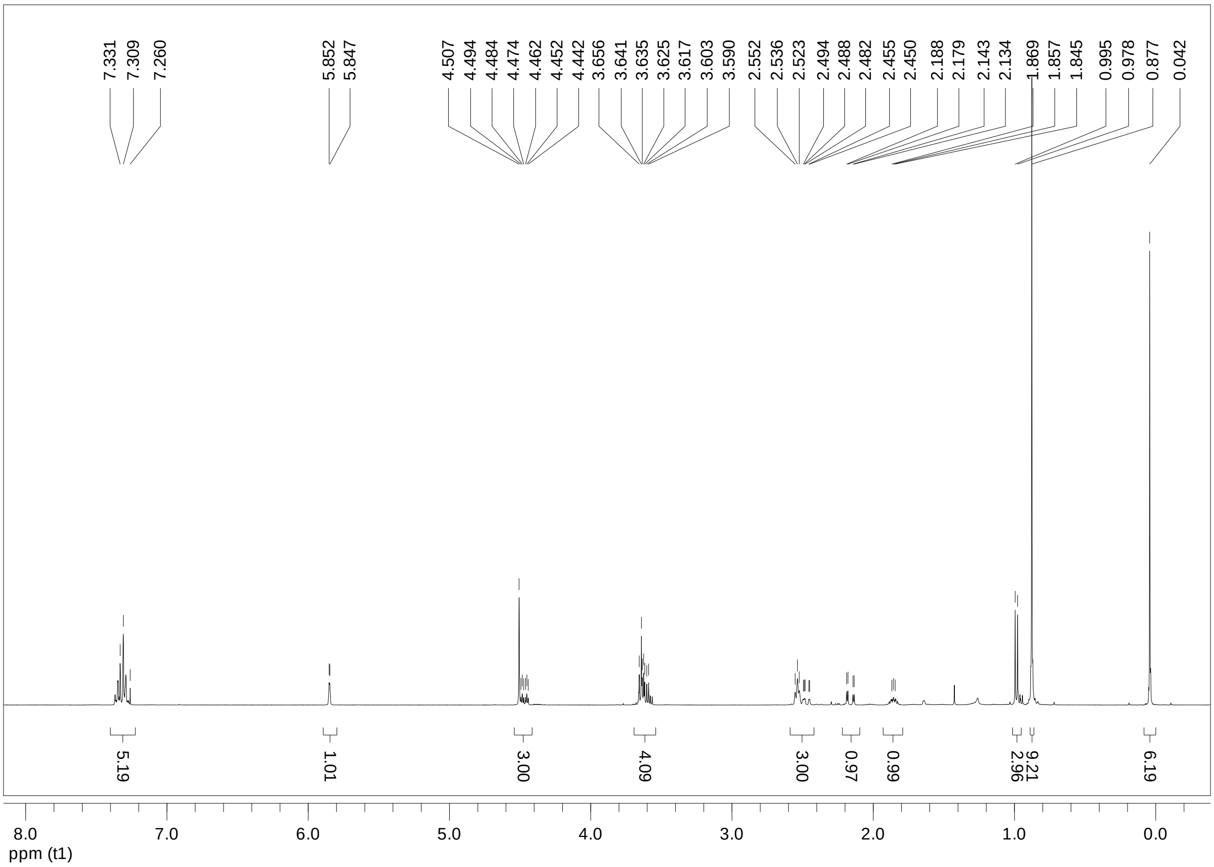
7.331
7.309
7.260
5.852
5.847
4.507
4.494
4.484
4.474
4.462
4.452
4.442
3.656
3.641
3.635
3.625
3.617
3.603
3.590
2.552
2.536
2.523
2.494
2.488
2.482
2.455
2.450
2.188
2.179
2.143
2.134
1.869
1.857
1.845
0.995
0.978
0.877
0.042
5.19
1.01
3.00
4.09
3.00
0.97
0.99
2.96
9.21
6.19
8.0
7.0
6.0
5.0
4.0
3.0
2.0
1.0
0.0
ppm (t1)

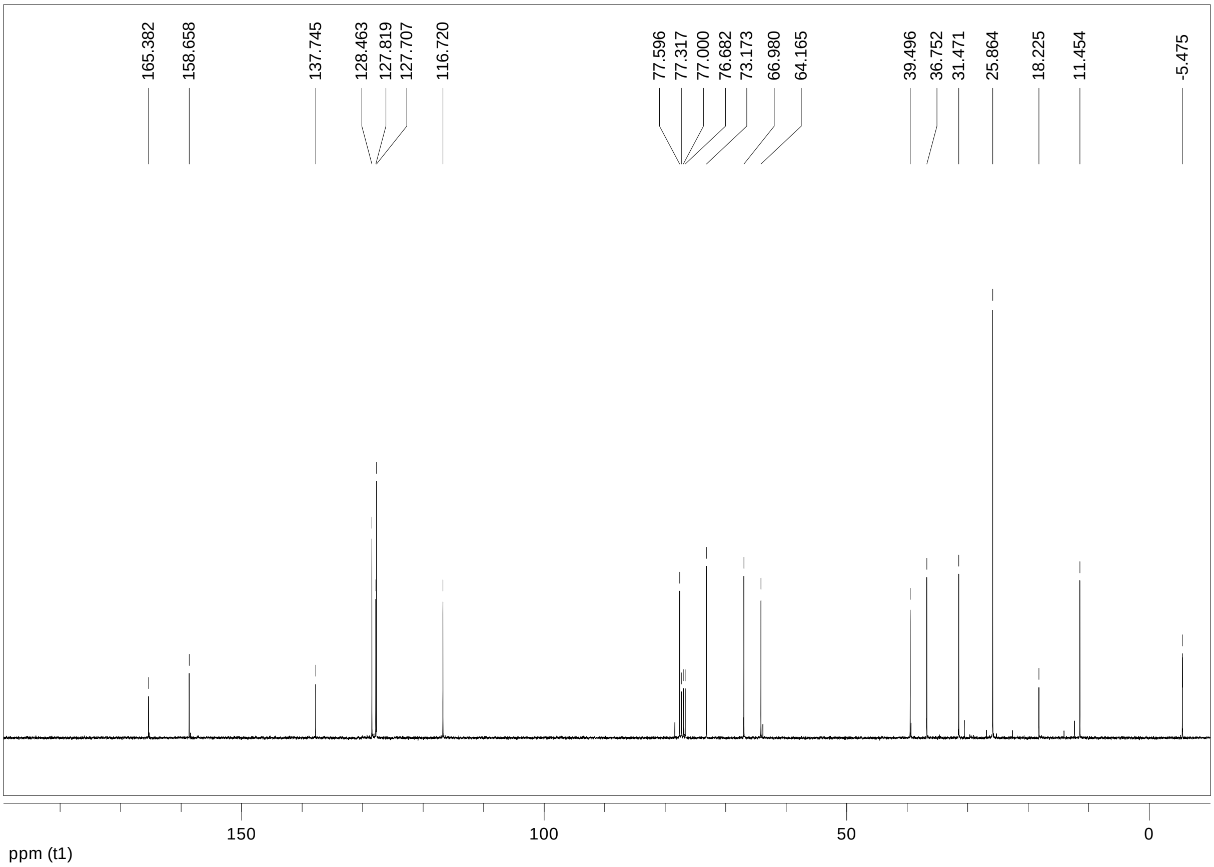
165.382
158.658
137.745
128.463
127.819
127.707
116.720
77.596
77.317
77.000
76.682
73.173
66.980
64.165
39.496
36.752
31.471
25.864
18.225
11.454
-5.475
150
100
50
0
ppm (t1)

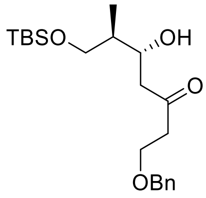

54

(5*R*,6*R*)-1-(Benzyloxy)-7-(*tert*-butyldimethylsilyloxy)-5-hydroxy-6-methyl-heptan-3-one (54): To a solution of **52** (56.6 mg, 0.15 mmol, 1.0 equiv) in dioxane/water (3:1, 1.5 mL) was added at rt OsO_4 (4% aq. solution, 18.3 µL, 2 mol%) followed by 2,6-lutidine (34.8 µL, 0.30 mmol, 2.0 equiv) and $NaIO_4$ (128 mg, 0.60 mmol, 4.0 equiv). The brownish reaction mixture was stirred for 2.5 h at rt, quenched with sat. $Na_2S_2O_3$ (6 mL) and diluted with EtOAc (15 mL)/brine (6 mL). The layers were separated and the aqueous layer was extracted with EtOAc (2 x 20 mL). The combined organic extracts were washed with brine, dried over $MgSO_4$ and the solvent was removed under reduced pressure. The residue was purified by column chromatography (hexane/EtOAc, 4:1 → 3:1) to afford **54** (53.6 mg, 94%) as a colorless oil.

R_f = 0.39 (hexane/ EtOAc, 3:1)
$[\alpha]_D^{24}$ = +13.2° [*c* = 0.700, $CHCl_3$].
^{1}H-NMR (400.1 MHz, $CDCl_3$): δ = 7.37-7.24 (m, 5H), 4.51 (s, 2H), 4.27 (m_c, 1H), 3.75 (m_c, 2H), 3.69 (dd, *J* = 4.4, 9.9 Hz, 1H), 3.62 (dd, *J* = 6.0, 10.0 Hz, 1H), 3.30 (d, *J* = 3.4 Hz, 1H), 2.76 (t, *J* = 6.3 Hz, 2H), 2.70 (dd, *J* = 9.3, 16.5 Hz, 1H), 2.52 (dd, *J* = 3.4, 16.5 Hz, 1H), 1.71 (m_c, 1H), 0.91 (d, *J* = 7.1 Hz, 3H), 0.89 (s, 9H), 0.06 (s, 6H).
^{13}C-NMR (100.6 MHz, $CDCl_3$): δ = 209.5, 138.0, 128.4, 127.7 (2C), 73.2, 69.8, 66.9, 65.2, 47.7, 43.7, 39.3, 25.9, 18.2, 10.8, –5.5, –5.6.
IR (neat, ν/cm^{-1}): 3484brs, 2954m, 2930m, 2858m, 1712m, 1468w, 1364m, 1254m, 1095s, 1025m, 1008m, 836s, 776s, 739m, 699m.
HRMS (ESI): *m/z* calcd for $C_{21}H_{36}NaO_4Si$ [M + Na]$^+$: 403.2275, found: 403.2273.

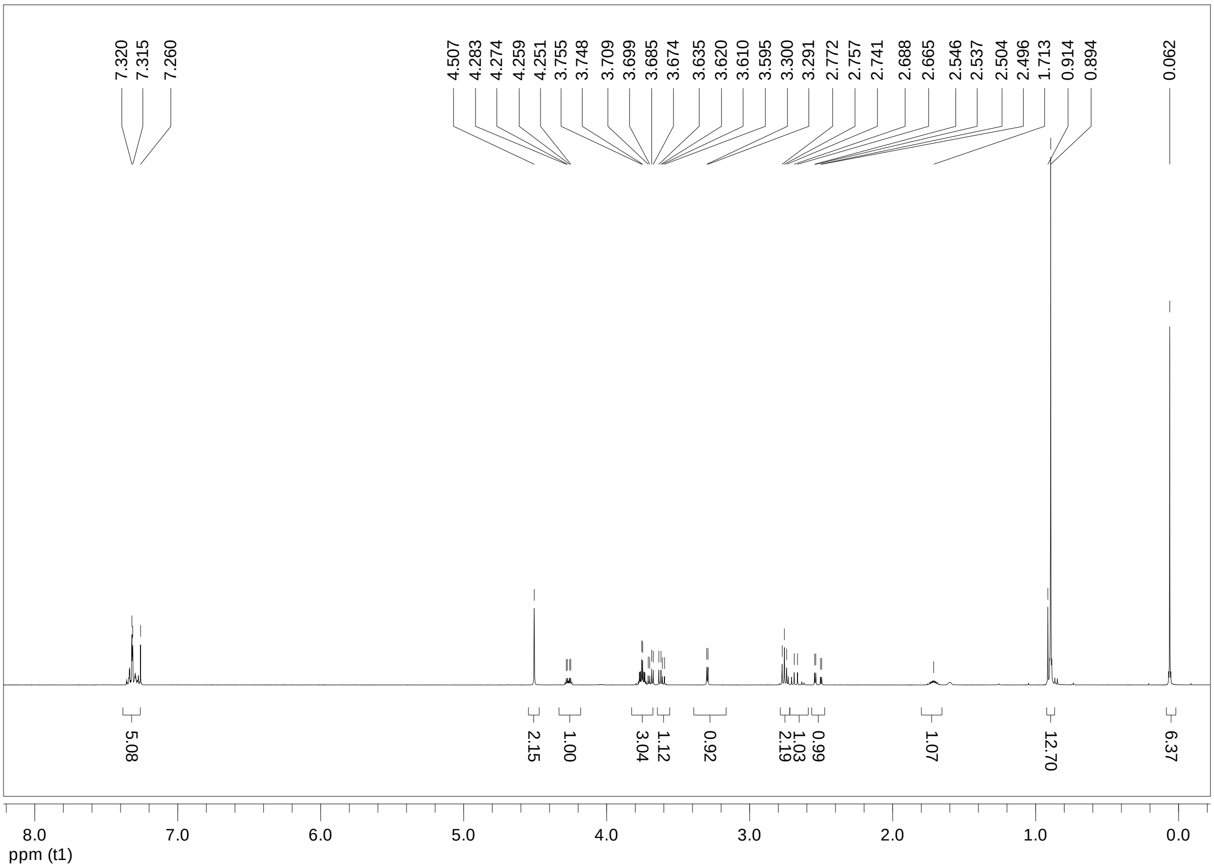
7.320
7.315
7.260
4.507
4.283
4.274
4.259
4.251
3.755
3.748
3.709
3.699
3.685
3.674
3.635
3.620
3.610
3.595
3.300
3.291
2.772
2.757
2.741
2.688
2.665
2.546
2.537
2.504
2.496
1.713
0.914
0.894
0.062
5.08
2.15
1.00
3.04
1.12
0.92
2.19
1.03
0.99
1.07
12.70
6.37
8.0
7.0
6.0
5.0
4.0
3.0
2.0
1.0
0.0
ppm (t1)

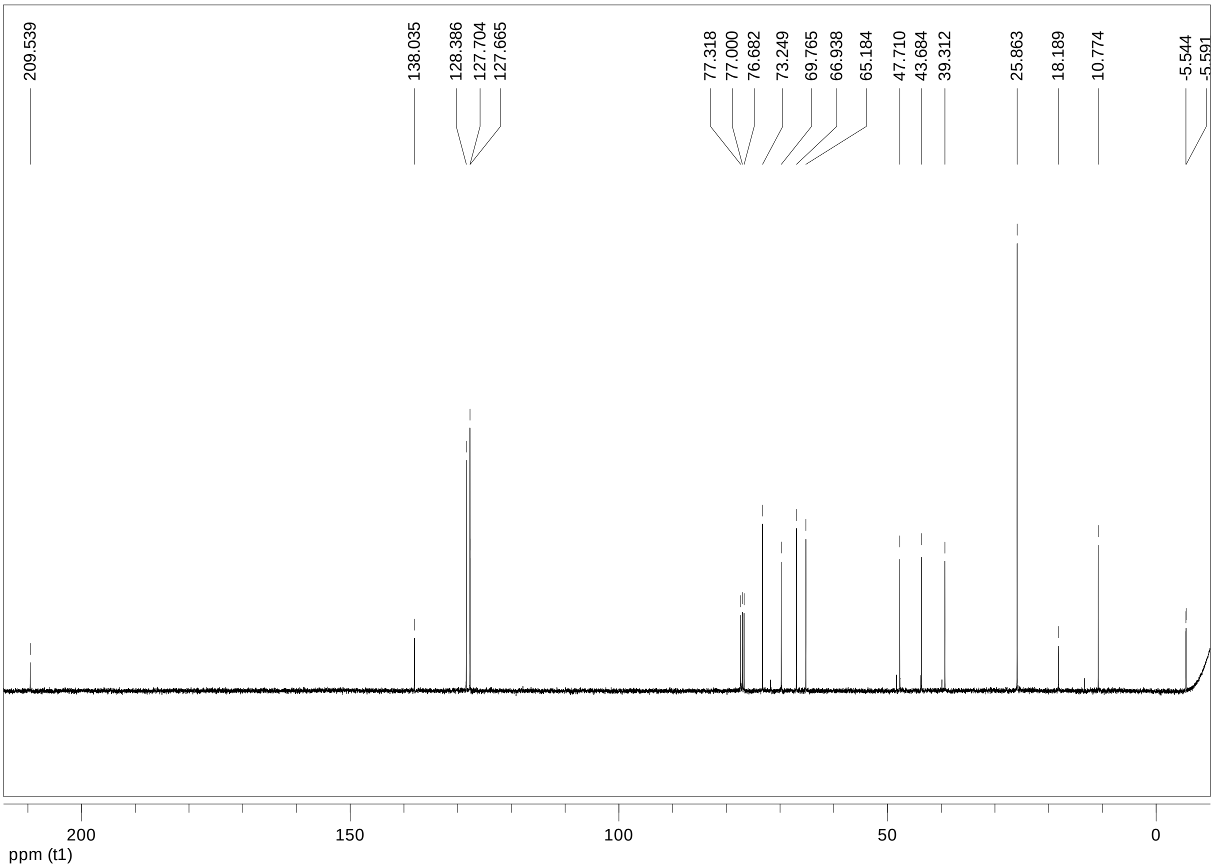
209.539
138.035
128.386
127.704
127.665
77.318
77.000
76.682
73.249
69.765
66.938
65.184
47.710
43.684
39.312
25.863
18.189
10.774
-5.544
-5.591
200
150
100
50
0
ppm (t1)

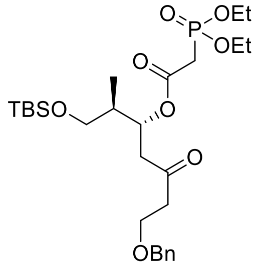

56

(2*R*,3*R*)-7-(Benzyloxy)-1-(*tert*-butyldimethylsilyloxy)-2-methyl-5-oxoheptan-3-yl 2-(diethoxyphosphoryl)acetate (56): To a solution of diethylphosphonoacetic acid (**55**) (0.38 mL, 2.36 mmol, 1.5 equiv) in CH_2Cl_2 (20 mL) over molecular sieves (570 mg powder, 4 Å) was added at 0 °C CME-carbodiimide (1.13 g, 2.68 mmol, 1.7 equiv) in one portion. After stirring the reaction mixture for 10 min at 0 °C, a solution of **54** (599 mg, 1.57 mmol, 1.0 equiv) in CH_2Cl_2 (19 mL) was added at that temperature. The cooling bath was removed and a catalytic amount of DMAP (19.2 mg, 10 mol%) was added. After stirring for 1 h at rt, the reaction mixture was directly purified by column chromatography (CH_2Cl_2,, then EtOAc) to afford **56** (818 mg, 93%) as a colorless oil.

R_f = 0.56 (EtOAc)

$[\alpha]_D^{24}$ = +4.5° [*c* = 0.845, $CHCl_3$].

^{1}H-NMR (400.1 MHz, $CDCl_3$): δ = 7.36-7.23 (m, 5H), 5.42 (m_c, 1H), 4.48 (s, 2H), 4.13 (m_c, 4H), 3.71 (t, *J* = 6.3 Hz, 2H), 3.50 (dd, *J* = 2.4, 6.0 Hz, 2H), 2.95-2.66 (m, 6H), 1.90 (m_c, 1H), 1.32 (t, *J* = 7.1 Hz, 6H), 0.91 (d, *J* = 6.9 Hz, 3H), 0.88 (s, 9H), 0.02 (s, 6H).

^{13}C-NMR (100.6 MHz, $CDCl_3$): δ = 205.9, 164.9 (d, *J* = 6.4 Hz), 138.0, 128.3, 127.7, 127.6, 73.2, 72.1, 65.1, 64.4, 62.5 (t, *J* = 6.7 Hz), 45.3, 43.1, 39.0, 34.3 (d, *J* = 134 Hz), 25.8, 18.1, 16.3 (d, *J* = 6.2 Hz), 11.5, –5.5, –5.6.

^{31}P-NMR (162.0 MHz, $CDCl_3$): δ = 19.8.

IR (neat, ν/cm^{-1}): 2954m, 2931m, 2858m, 1736s, 1469w, 1392w, 1261s, 1210w, 1099s, 1052s, 1025s, 969m, 838s, 777m, 743m, 696w.

HRMS (ESI): *m/z* calcd for $C_{27}H_{47}NaO_8PSi$ [M + Na]$^+$: 581.2670, found: 581.2659.

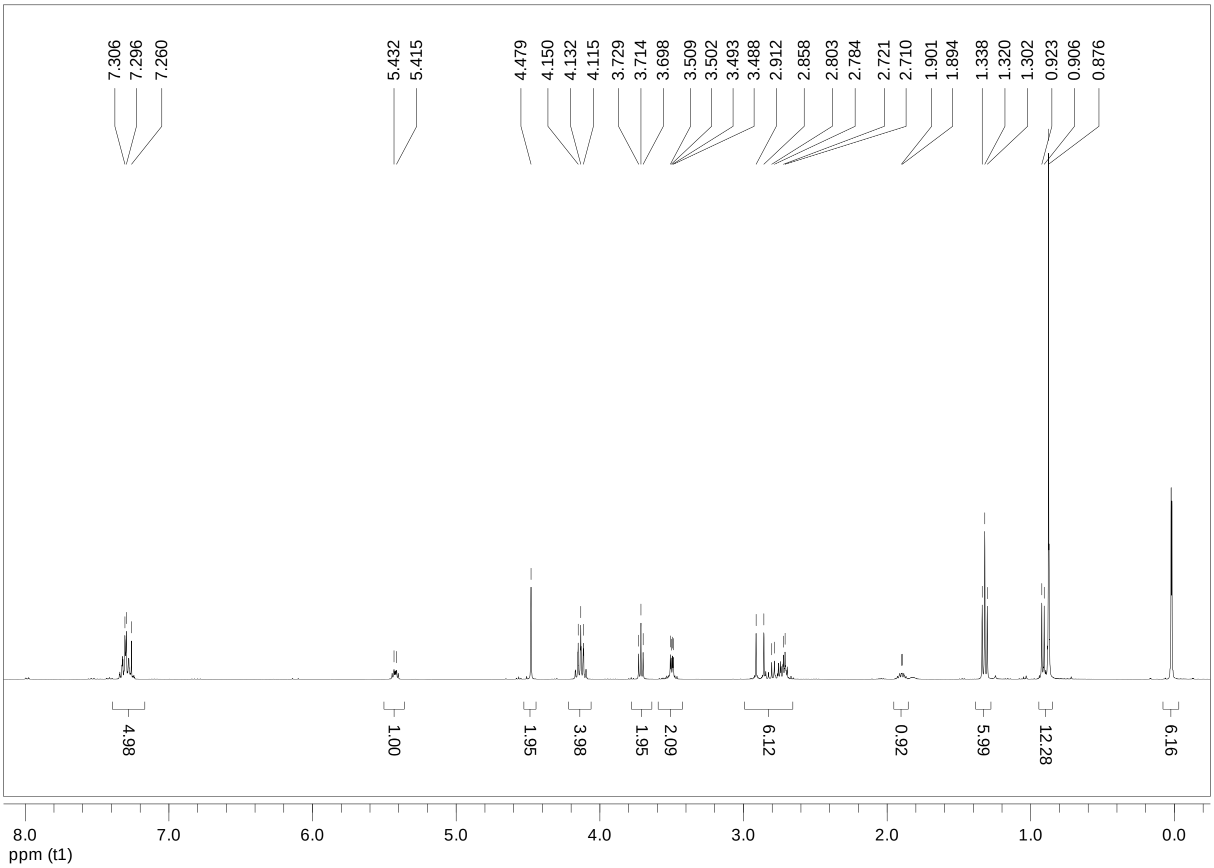
7.306
7.296
7.260
5.432
5.415
4.479
4.150
4.132
4.115
3.729
3.714
3.698
3.509
3.502
3.493
3.488
2.912
2.858
2.803
2.784
2.721
2.710
1.901
1.894
1.338
1.320
1.302
0.923
0.906
0.876
4.98
1.00
1.95
3.98
1.95
2.09
6.12
0.92
5.99
12.28
6.16
8.0
7.0
6.0
5.0
4.0
3.0
2.0
1.0
0.0
ppm (t1)

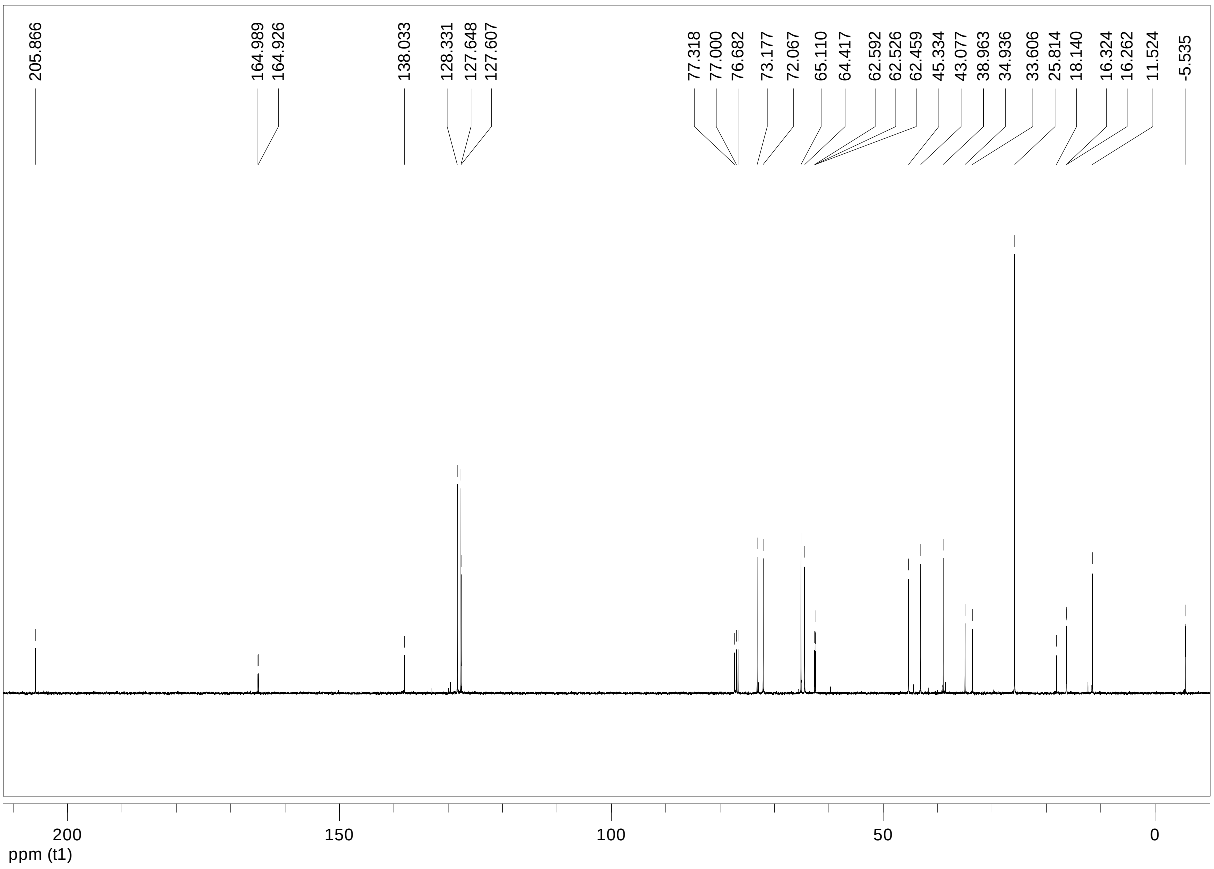
205.866
164.989
164.926
138.033
128.331
127.648
127.607
77.318
77.000
76.682
73.177
72.067
65.110
64.417
62.592
62.526
62.459
45.334
43.077
38.963
34.936
33.606
25.814
18.140
16.324
16.262
11.524
-5.535
200
150
100
50
0
ppm (t1)

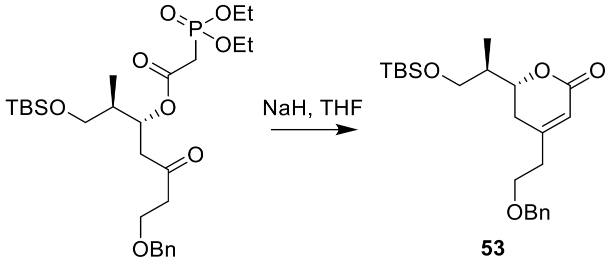

(*R*)-4-(2-(Benzyloxy)ethyl)-6-((*R*)-1-(*tert*-butyldimethylsilyloxy)propan-2-yl)-5,6-dihydro-2H-pyran-2-one (53): To a solution of **56** (38.7 mg, 69.3 µmol, 1.0 equiv) in THF (2.0 mL) was added at 0 °C NaH (1.8 mg, 76.2 µmol, 1.1 equiv) in one portion. After stirring for 45 min at 0 °C, the reaction mixture was quenched with sat NH_4Cl (8 mL) and diluted with Et_2O (10 mL). The layers were separated and the aqueous layer was extracted with Et_2O (2 x 15 mL). The combined organic extracts were dried over $MgSO_4$, filtered and concentrated under reduced pressure. The crude product was purified by column chromatography (hexane/EtOAc, 3:1) to give **53** (23.4 mg, 83%) as a colorless oil. The analytical data for **53** were identical to those reported above (*vide supra*).

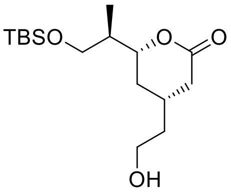

57

(4*R*,6*R*)-6-((*R*)-1-(*tert*-butyldimethylsilyloxy)propan-2-yl)-4-(2-hydroxyethyl)-tetrahydro-2H-pyran-2-one (57): To a solution of **53** (4.09 g, 10.1 mmol, 1.0 equiv) in EtOAc (80 mL) was added palladium hydroxide on carbon (360 mg, 5 mol%, 20% wt/wt Pd). The mixture was stirred under a hydrogen atmosphere (9 bar) for 23 h while being monitored by MS and TLC. Then, the heterogeneous suspension was filtered over celite, the solvent was removed under reduced pressure and the residue was purified by column chromatography (hexane/EtOAc, 1:1 → 0:1) to afford **57** (3.14 g, 98%) as a colorless oil.

R*f* = 0.21 (hexane/EtOAc, 1:1)
$[\alpha]_D^{24}$ = −22.5° [*c* = 0.535, $CHCl_3$].
^{1}H-NMR (400.1 MHz, $CDCl_3$): δ = 4.45 (td, *J* = 3.0, 11.9 Hz, 1H), 3.72 (t, *J* = 6.3 Hz, 2H), 3.62 (dd, *J* = 7.6, 9.8 Hz, 1H), 3.54 (dd, *J* = 5.4, 10.0 Hz, 1H), 2.73 (dd, *J* = 5.3, 16.9 Hz, 1H), 2.17 (m_c, 1H), 2.09 (dd, *J* = 10.5, 16.8 Hz, 1H), 1.90 (d, *J* = 13.8 Hz, 1H), 1.80 (m_c, 1H), 1.71-1.49 (m, 2H), 1.38 (*J* = 11.9, 25.0 Hz, 1H), 0.93 (d, *J* = 6.9 Hz, 3H), 0.88 (s, 9H), 0.04 (s, 6H).
^{13}C-NMR (100.6 MHz, $CDCl_3$): δ = 171.6, 80.2, 64.2, 59.7, 40.3, 38.9, 36.4, 32.7, 28.6, 25.9, 18.2, 10.8, −5.5.
IR (neat, ν/cm^{-1}): 3434br, 2953m, 2928m, 2857m, 1720m, 1471w, 1389w, 1250s, 1088m, 1054m, 1006m, 834s, 775s, 668m.
HRMS (ESI): *m/z* calcd for $C_{16}H_{33}O_4Si$ [M + H]$^+$: 317.2143; found: 317.2143.

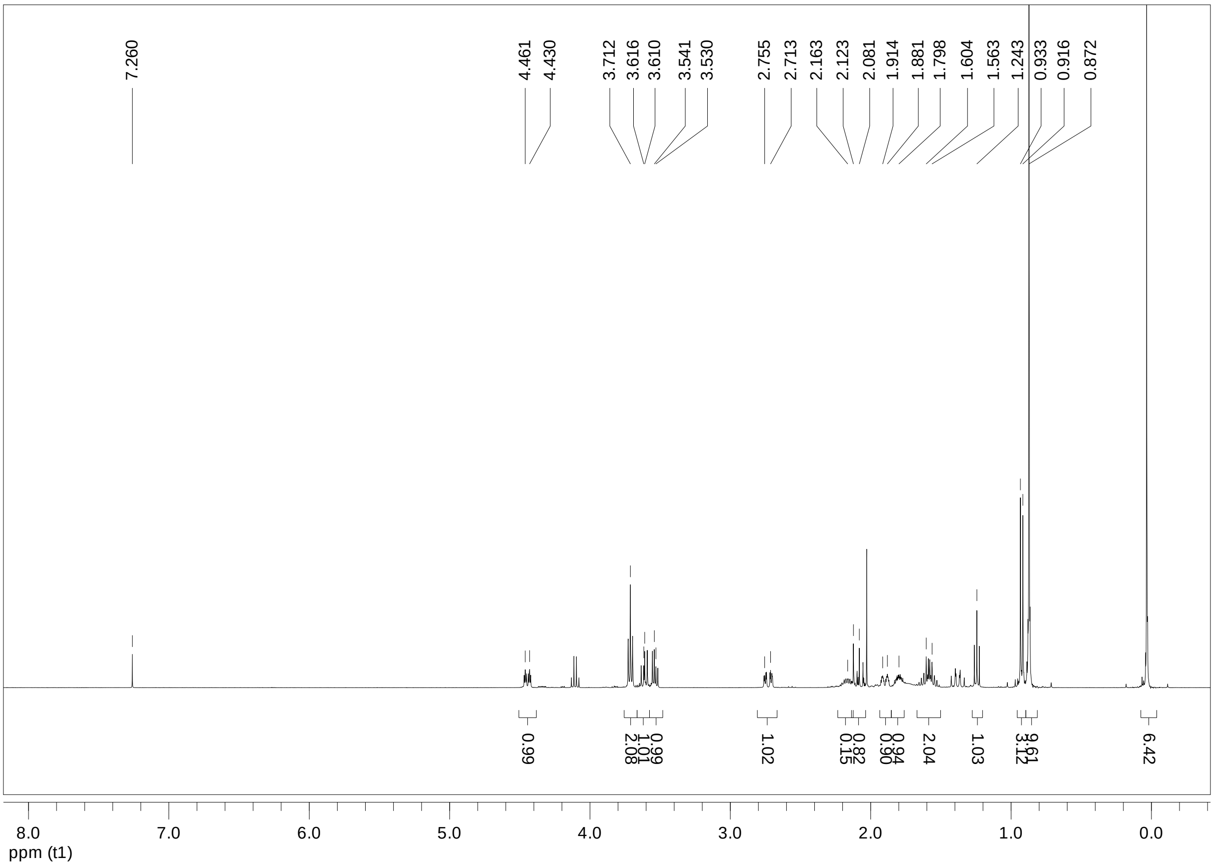
7.260
4.461
4.430
3.712
3.616
3.610
3.541
3.530
2.755
2.713
2.163
2.123
2.081
1.914
1.881
1.798
1.604
1.563
1.243
0.933
0.916
0.872
0.99
2.08
1.01
0.99
1.02
0.15
0.82
0.90
0.94
2.04
1.03
3.12
9.61
6.42
8.0
7.0
6.0
5.0
4.0
3.0
2.0
1.0
0.0
ppm (t1)

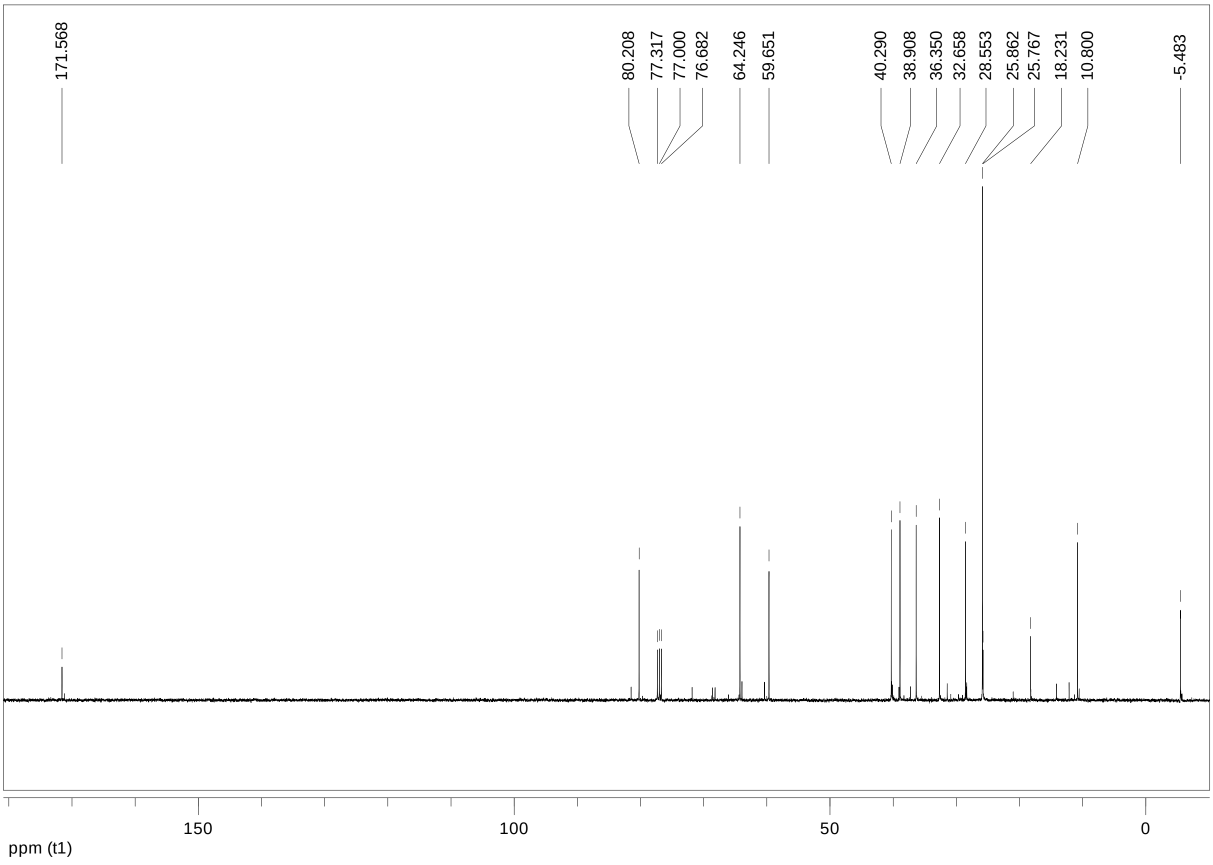
171.568
80.208
77.317
77.000
76.682
64.246
59.651
40.290
38.908
36.350
32.658
28.553
25.862
25.767
18.231
10.800
-5.483
150
100
50
0
ppm (t1)

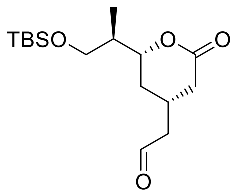

8

2-((2*R*,4*S*)-2-((*R*)-1-(*tert*-butyldimethylsilyloxy)propan-2-yl)-6-oxotetrahydro-2H-pyran-4-yl)acetaldehyde (8): To a solution of oxalyl chloride (0.21 mL, 2.46 mmol, 1.5 equiv) in CH_2Cl_2 (23 mL) at –78 °C was added dropwise a solution of DMSO (0.35 mL, 4.92 mmol, 3.0 equiv) in CH_2Cl_2 (0.5 mL). After stirring at –78 °C for 10 min, a solution of **57** (519 mg, 1.64 mmol, 1.0 equiv) in CH_2Cl_2 (5 mL) was added dropwise. The resultant cloudy mixture was stirred at –78 °C for 1 h, and then TEA (0.91 mL, 6.56 mmol, 4.0 equiv) was added slowly and the reaction mixture was allowed to warm to room temperature (1 h). The reaction was quenched with water (20 mL), and the layers were separated. The aqueous phase was extracted with CH_2Cl_2 (3 x 20 mL) and the combined organic phases were washed with brine, dried over $MgSO_4$, filtered and concentrated *in vacuo*. The crude product was purified by column chromatography (hexane/EtOAc, 2:1) to afford **8** (483 mg, 94%) as a colorless oil.

R*f* = 0.52 (hexane/EtOAc, 1:1)
$[\alpha]_D^{24}$ = –27.5° [*c* = 0.525, $CHCl_3$].
^{1}H-NMR (400.1 MHz, $CDCl_3$): δ = 9.77(t, *J* = 1.0 Hz, 1H), 4.48 (ddd, *J* = 3.0, 3.5, 12.0 Hz, 1H), 3.61 (dd, *J* = 7.4, 10.0 Hz, 1H), 3.54 (dd, *J* = 5.4, 1.0 Hz, 1H), 2.78 (ddd, *J* = 1.7, 6.0, 17.6 Hz, 1H), 2.64-2.48 (m, 3H), 2.10 (dd, *J* = 9.8, 17.5 Hz, 1H), 1.94 (m_c, 1H), 1.80 (m_c, 1H), 1.40 (m_c, 1H), 0.92 (d, *J* = 6.9 Hz, 3H), 0.88 (s, 9H), 0.04 (s, 6H).
^{13}C-NMR (100.6 MHz, $CDCl_3$): δ = 199.6, 170.6, 79.9, 64.1, 49.9, 40.2, 35.9, 32.3, 26.2, 25.9, 18.2, 10.8, –5.5.
IR (neat, ν/cm^{-1}): 2954m, 2928m, 2857m, 1725s, 1471m, 1388m, 1361w, 1248s, 1080m, 1005m, 921w, 835s, 776s, 668m.
HRMS (ESI): *m/z* calcd for $C_{16}H_{30}NaO_4Si$ $[M + Na]^+$: 337.1806, found: 337.1821.

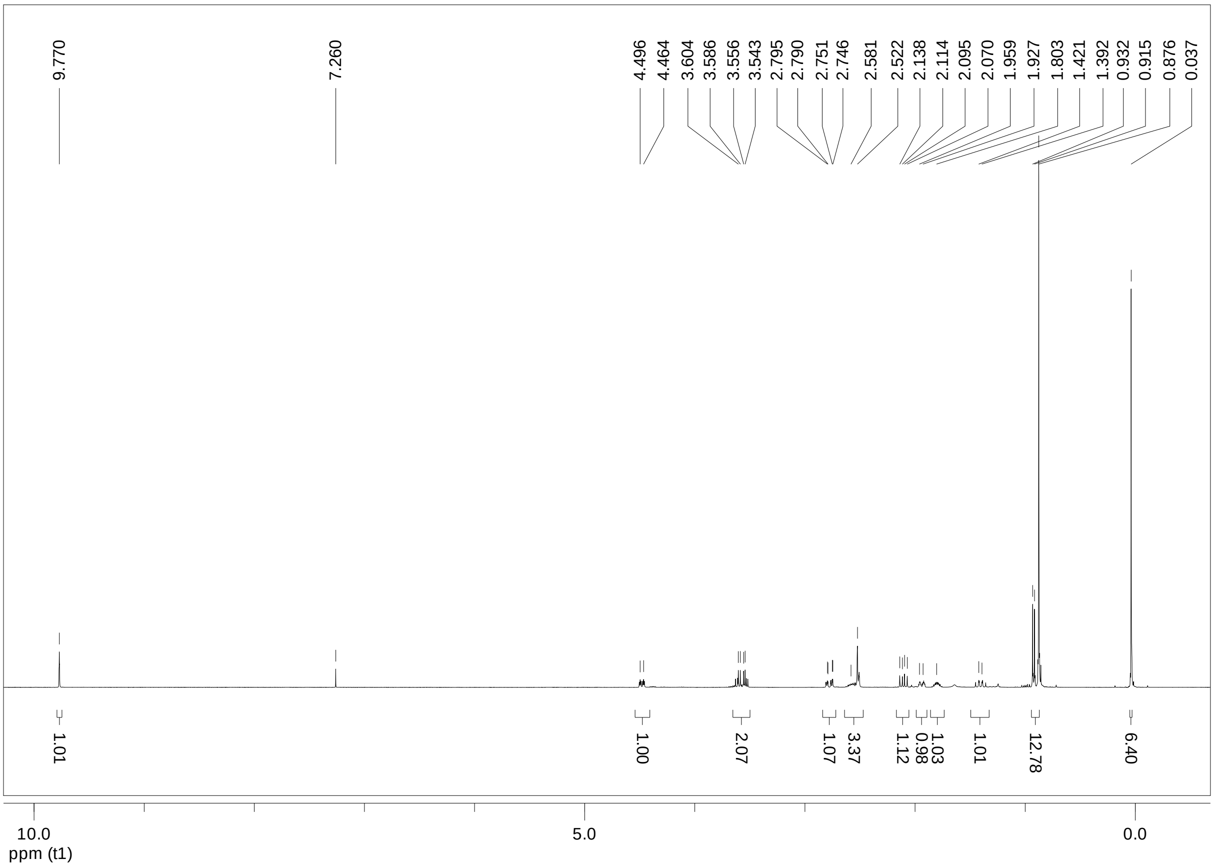
9.770
7.260
4.496
4.464
3.604
3.586
3.556
3.543
2.795
2.790
2.751
2.746
2.581
2.522
2.138
2.114
2.095
2.070
1.959
1.927
1.803
1.421
1.392
0.932
0.915
0.876
0.037
1.01
1.00
2.07
1.07
3.37
1.12
0.98
1.03
1.01
12.78
6.40
10.0
5.0
0.0
ppm (t1)

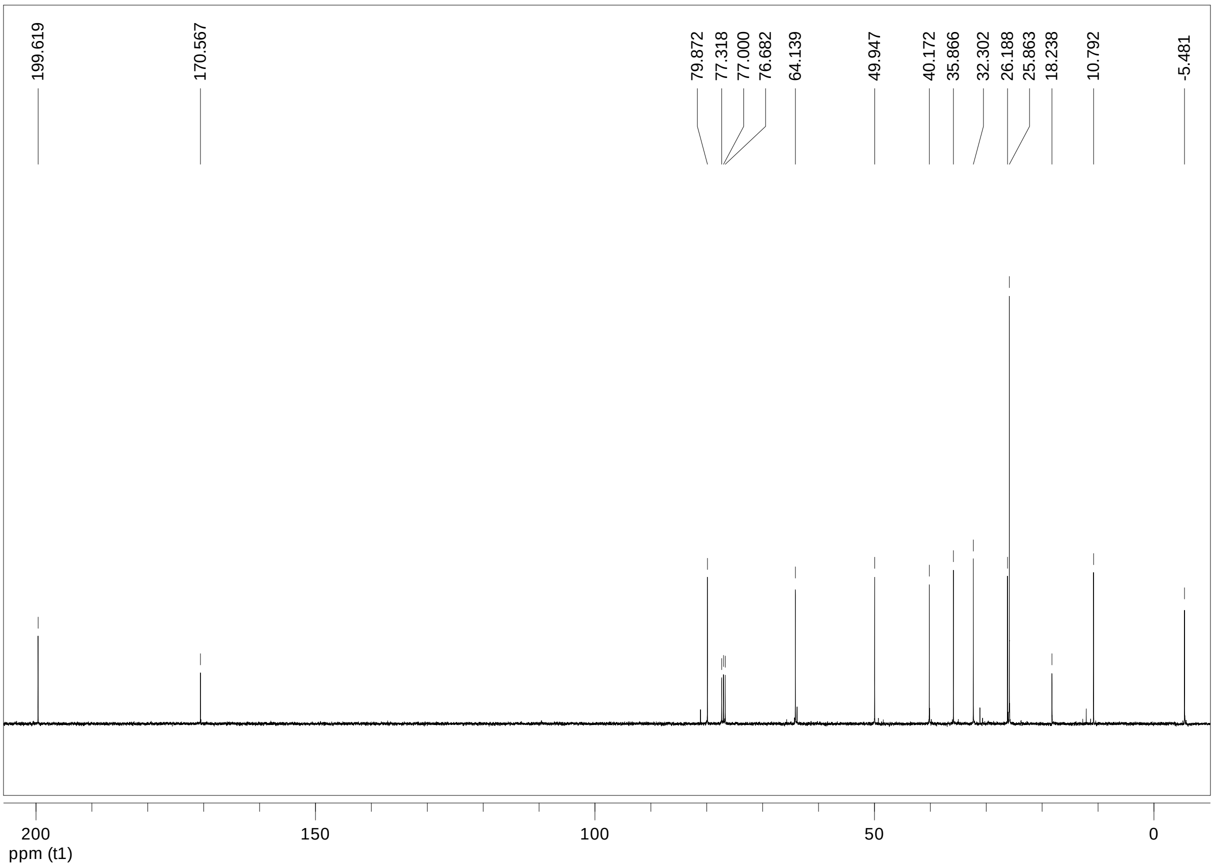
199.619
170.567
79.872
77.318
77.000
76.682
64.139
49.947
40.172
35.866
32.302
26.188
25.863
18.238
10.792
-5.481
200
150
100
50
0
ppm (t1)

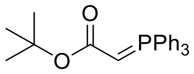

58

***tert*-Butyl 2-(triphenyl-l5-phosphaneylidene)acetate (58):** To a stirred solution of triphenylphosphine (9.90 g, 37.7 mmol, 1.0 equiv) in toluene (22 mL) was added dropwise at 0 °C *tert*-butyl bromoacetate (5.50 mL, 37.7 mmol, 1.0 equiv). The reaction mixture was allowed to warm to rt and stirring was continued over night. The colorless precipitate was filtered off, washed with pentane (2 x 25 mL) and dried under high vacuum to afford the phosphonium salt as a colorless solid (16.1 g, 93%).
This salt was dissolved in water (300 mL) and the solution was washed with Et_2O (60 mL). The aqueous layer was separated and phenolphthalein (6 mg) was added. The homogeneous solution was cooled to 0 °C and aq. NaOH (at the beginning 8 mL of a 4 M solution, then 1 M solution) was added dropwise until the pink color persisted. Unfortunately, the ylide did not precipitate due to small amounts of Et_2O present, and subsequent filtration failed. Therefore, the aqueous solution was extracted with CH_2Cl_2 (3 x 100 mL) and the combined organic extracts were dried over $MgSO_4$, filtered and concentrated under reduced pressure. After drying under high vacuum, a gummy resin was formed, which did not solidify (13.4 g). This residue was dissolved in MeOH (30 mL) and water (70 mL) was added quickly to this vigorously stirred solution, which led to immediate precipitation of the ylide. This heterogeneous solution was stored in the fridge for 3 h, filtered and washed repeatedly with cold water (0 °C, 5 x 20 mL). After drying under high vacuum, **58** (12.5 g, 95%) was obtained as a colorless solid.[14]

^{1}H-NMR (400.1 MHz, $CDCl_3$): δ = 7.74-7.58 (m, 6H), 7.57-7.48 (m, 3H), 7.48-7.35 (m, 6H), 2.67 (br s, 1H), 1.20 (br s, 9H).

[14] The procedure of *Cabral dos Santos et al.* had to be modified due to difficulties with the precipitation of **58** (*vide supra*): Cabral dos Santos, L. *et al. Heterocycles* **2007**, *73*, 751-768.

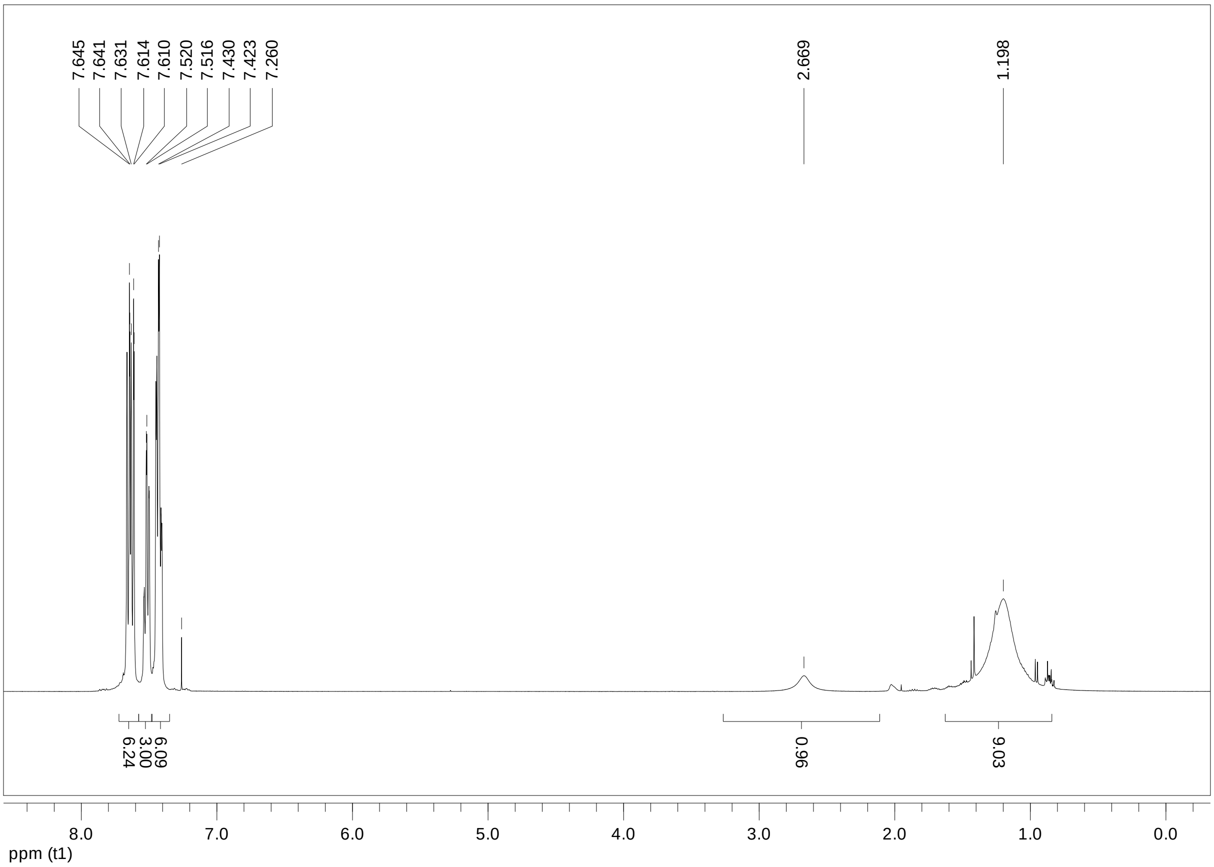

7.645
7.641
7.631
7.614
7.610
7.520
7.516
7.430
7.423
7.260
2.669
1.198
6.24
3.00
6.09
0.96
9.03
8.0
7.0
6.0
5.0
4.0
3.0
2.0
1.0
0.0
ppm (t1)

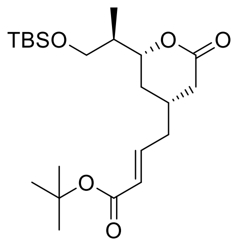

59

***tert*-Butyl (*E*)-4-((2*R*,4*R*)-2-((*R*)-1-(*tert*-butyldimethylsilyloxy)propan-2-yl)-6-oxo-tetrahydro-2H-pyran-4-yl)but-2-enoate (59):** To a solution of **8** (73 mg, 0.23 mmol, 1.0 equiv) in CH_2Cl_2 (2.3 mL) was added 0 °C the ylide **57**[14] (113 mg, 0.30 mmol, 1.3 equiv). After stirring for 30 min at that temperature, the reaction mixture was allowed to warm to rt and was concentrated under reduced pressure. The residue was purified by column chromatography (hexane/EtOAc, 6:1 → 5:1) to give **59** as a colorless oil (84.4 mg, 84%).

R_f = 0.32 (hexane/EtOAc, 3:1)

$[\alpha]_D^{24}$ = –16.0° [*c* = 0.904, $CHCl_3$].

^{1}H-NMR (400.1 MHz, $CDCl_3$): δ = 6.75 (td, *J* = 7.3, 15.5 Hz, 1H), 5.79 (d, *J* = 15.6 Hz, 1H), 4.43 (td, *J* = 3.3, 11.9 Hz, 1H), 3.61 (dd, *J* = 7.4, 10.0 Hz, 1H), 3.54 (dd, *J* = 5.3, 10.0 Hz, 1H), 2.70 (m_c, 1H), 2.28-2.03 (m, 4H), 1.89 (m_c, 1H), 1.80 (m_c, 1H), 1.48 (s, 9H), 1.36 (m_c, 1H), 0.93 (d, *J* = 6.9 Hz, 3H), 0.88 (s, 9H), 0.04 (s, 6H).

^{13}C-NMR (100.6 MHz, $CDCl_3$): δ = 170.9, 165.4, 143.1, 125.8, 80.5, 80.1, 64.2, 40.3, 38.6, 36.1, 32.3, 31.2, 28.1, 25.9, 18.2, 10.9, −5.5.

IR (neat, ν/cm^{-1}): 2955m, 2929m, 2857w, 1733m, 1712m, 1654w, 1472w, 1367m, 1249m, 1150s, 1082m, 1027m, 980m, 835s, 775s, 754m, 669m.

HRMS (ESI): *m/z* calcd for $C_{22}H_{44}NO_5Si$ [M + NH_4]$^+$: 430.2983, found: 430.2982.

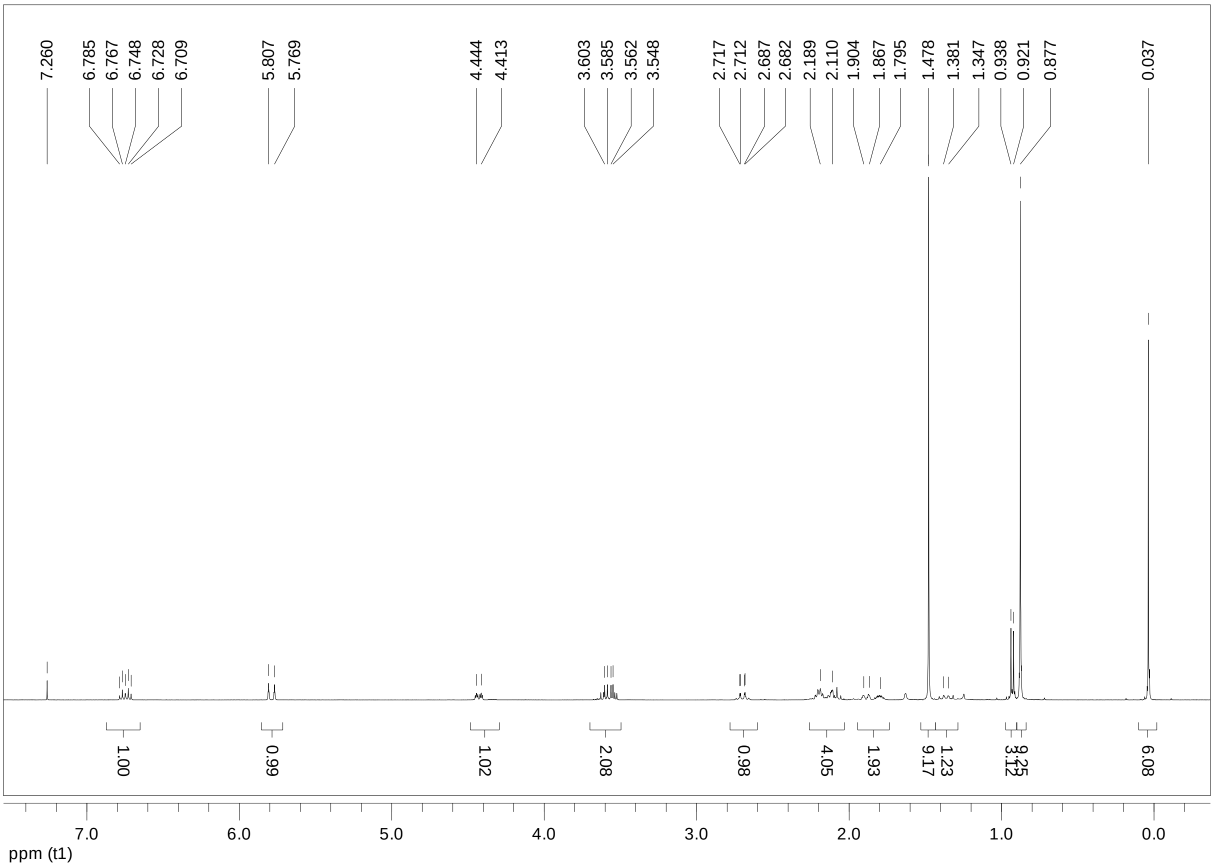
7.260
6.785
6.767
6.748
6.728
6.709
5.807
5.769
4.444
4.413
3.603
3.585
3.562
3.548
2.717
2.712
2.687
2.682
2.189
2.110
1.904
1.867
1.795
1.478
1.381
1.347
0.938
0.921
0.877
0.037
1.00
0.99
1.02
2.08
0.98
4.05
1.93
9.17
1.23
3.12
9.25
6.08
7.0
6.0
5.0
4.0
3.0
2.0
1.0
0.0
ppm (t1)

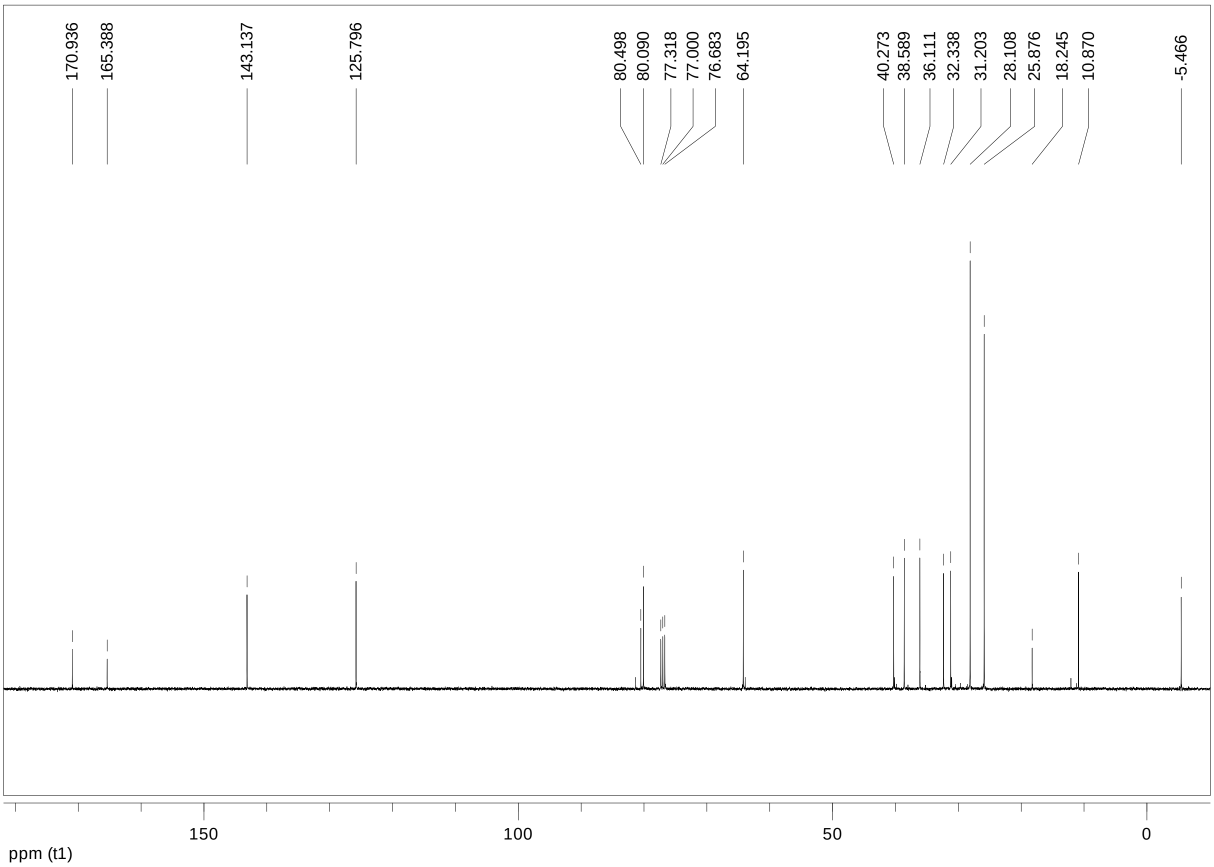
170.936
165.388
143.137
125.796
80.498
80.090
77.318
77.000
76.683
64.195
40.273
38.589
36.111
32.338
31.203
28.108
25.876
18.245
10.870
-5.466
150
100
50
0
ppm (t1)

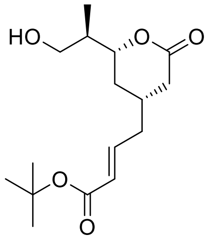

60

***tert*-Butyl (*E*)-4-((2*R*,4*R*)-2-((*R*)-1-hydroxypropan-2-yl)-6-oxotetrahydro-2H-pyran-4-yl)but-2-enoate (60):** To a solution of **59** (79.9 mg, 0.19 mmol, 1.0 equiv) in THF (1.9 mL) was added at 0 °C TBAF (0.77 mL, 0.77 mmol, 4.0 equiv, 1.0 M in THF) and AcOH (44 µL, 0.77 mmol, 4.0 equiv). After stirring for 18 h at rt, the reaction mixture was quenched with sat. NH_4Cl (10 mL), the aqueous layer was extracted with EtOAc (3 x 15 mL). The combined organic extracts were washed with brine, dried over $MgSO_4$ and the solvent was removed under reduced pressure. The residue was purified by column chromatography (hexane/EtOAc, 1:1 → 1:2 → 0:1) to give **60** (57.2 mg, 96%) as a colorless oil.

$\mathbf{R}_f$ = 0.54 (EtOAc)

$[\alpha]_D^{24}$ = –12.9° [*c* = 0.524, $CHCl_3$]

^{1}H-NMR (400.1 MHz, $CDCl_3$): δ = 6.74 (td, *J* = 7.3, 15.5 Hz, 1H), 5.79 (td, *J* = 1.2, 15.4 Hz, 1H), 4.52 (td, *J* = 3.0, 12.0 Hz, 1H), 3.70 (dd, *J* = 7.8, 10.7 Hz, 1H), 3.61 (dd, *J* = 5.3, 10.7 Hz, 1H), 2.71 (m_c, 1H), 2.27-2.08 (m, 4H), 1.93-1.82 (m, 2H), 1.48 (s, 9H), 1.45-1.29 (m, 1H), 0.96 (d, *J* = 7.0 Hz, 3H).

^{13}C-NMR (100.6 MHz, $CDCl_3$): δ = 170.9, 165.4, 143.0, 125.8, 80.6, 80.1, 64.2, 40.3, 38.5, 36.1, 32.1, 31.2, 28.1, 10.5.

IR (neat, ν/cm^{-1}): 3451br, 2975m, 2930m, 2885m, 1709s, 1653m, 1457w, 1391m, 1367m, 1332m, 1313m, 1294m, 1250s, 1151s, 1034m, 988m, 850w.

HRMS (ESI): *m/z* calcd for $C_{16}H_{30}NO_5$ $[M + NH_4]^+$: 316.2118, found: 316.2115.

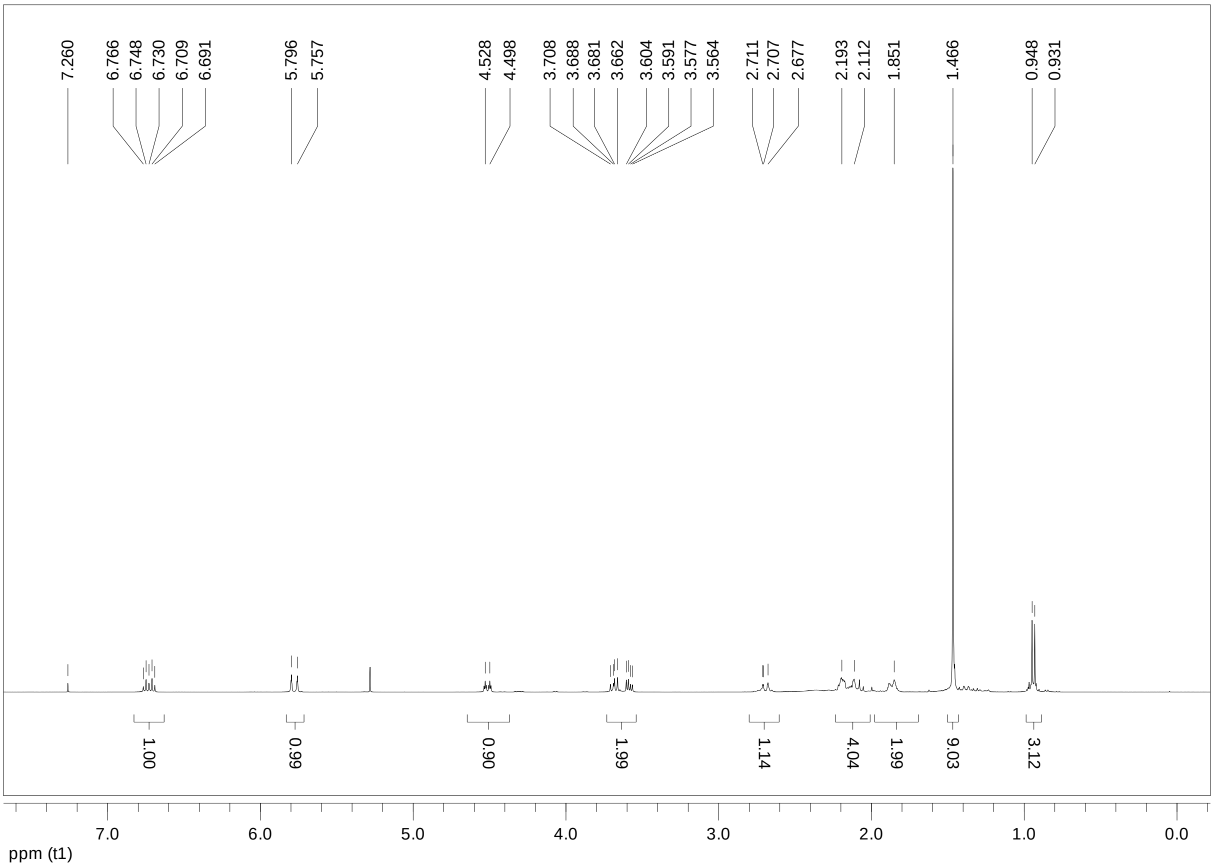
7.260
6.766
6.748
6.730
6.709
6.691
5.796
5.757
4.528
4.498
3.708
3.688
3.681
3.662
3.604
3.591
3.577
3.564
2.711
2.707
2.677
2.193
2.112
1.851
1.466
0.948
0.931
1.00
0.99
0.90
1.99
1.14
4.04
1.99
9.03
3.12
7.0
6.0
5.0
4.0
3.0
2.0
1.0
0.0
ppm (t1)

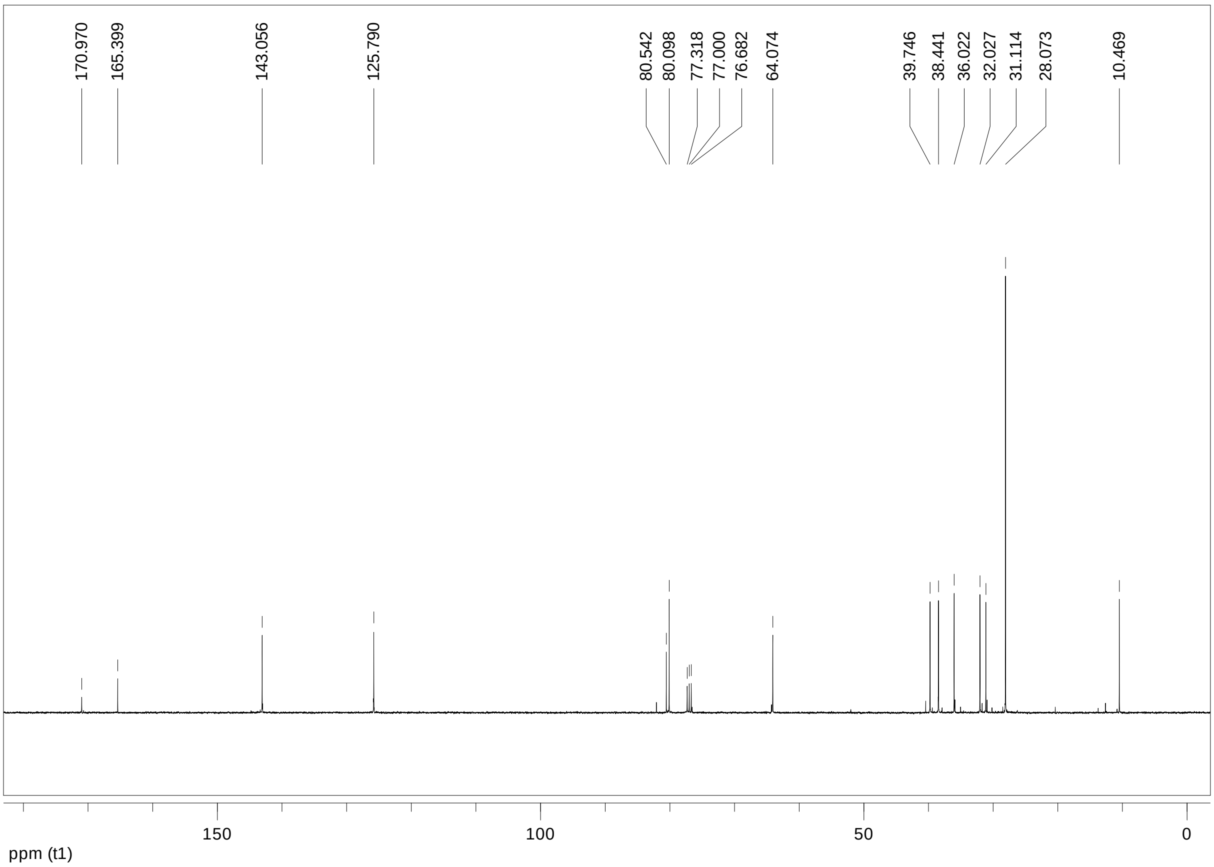
170.970
165.399
143.056
125.790
80.542
80.098
77.318
77.000
76.682
64.074
39.746
38.441
36.022
32.027
31.114
28.073
10.469
150
100
50
0
ppm (t1)

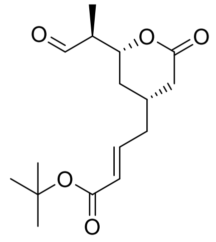

61

***tert*-Butyl (*E*)-4-((4*R*,6*R*)-2-oxo-6-((*S*)-1-oxopropan-2-yl)tetrahydro-2H-pyran-4-yl)but-2-enoate (61):** To a solution of **60** (442 mg, 1.48 mmol, 1.0 equiv) in CH_2Cl_2 (30 mL) was added at 0 °C *Dess-Martin* periodinane (754 mg, 1.78 mmol, 1.2 equiv) in one portion. After stirring for 2 h at rt, sat. $Na_2S_2O_3$ (30 mL), sat. $NaHCO_3$ (30 mL) and Et_2O (150 mL) were added at rt to the reaction mixture. The layers were separated and the aqueous layer was extracted with Et_2O (50 mL). The combined organic extracts were washed with sat. $NaHCO_3$ (30 mL) and brine, dried over $MgSO_4$, filtered and concentrated under reduced pressure. The crude product **140** (514 mg, containing residual solvent) was used for the next step without further purification.

R*f* = 0.75 (EtOAc).

^{1}H-NMR (400.1 MHz, $CDCl_3$): δ = 9.73 (d, *J* = 0.9 Hz, 1H), 6.73 (td, *J* = 7.2, 15.5 Hz, 1H), 5.80 (d, *J* = 15.6 Hz, 1H), 4.70 (ddd, *J* = 2.9, 4.7, 12.0 Hz, 1H), 2.74 (m_c, 1H), 2.62 (m_c, 1H), 2.29-2.06 (m, 4H), 1.99 (m_c, 1H), 1.48 (s, 9H), 1.34 (m_c, 1H), 1.24 (d, *J* = 7.3 Hz, 3H).

^{13}C-NMR (100.6 MHz, $CDCl_3$): δ = 201.7, 169.8, 165.3, 142.6, 126.1, 80.7, 78.8, 50.2, 38.3, 35.9, 32.0, 31.0, 28.1, 8.6.

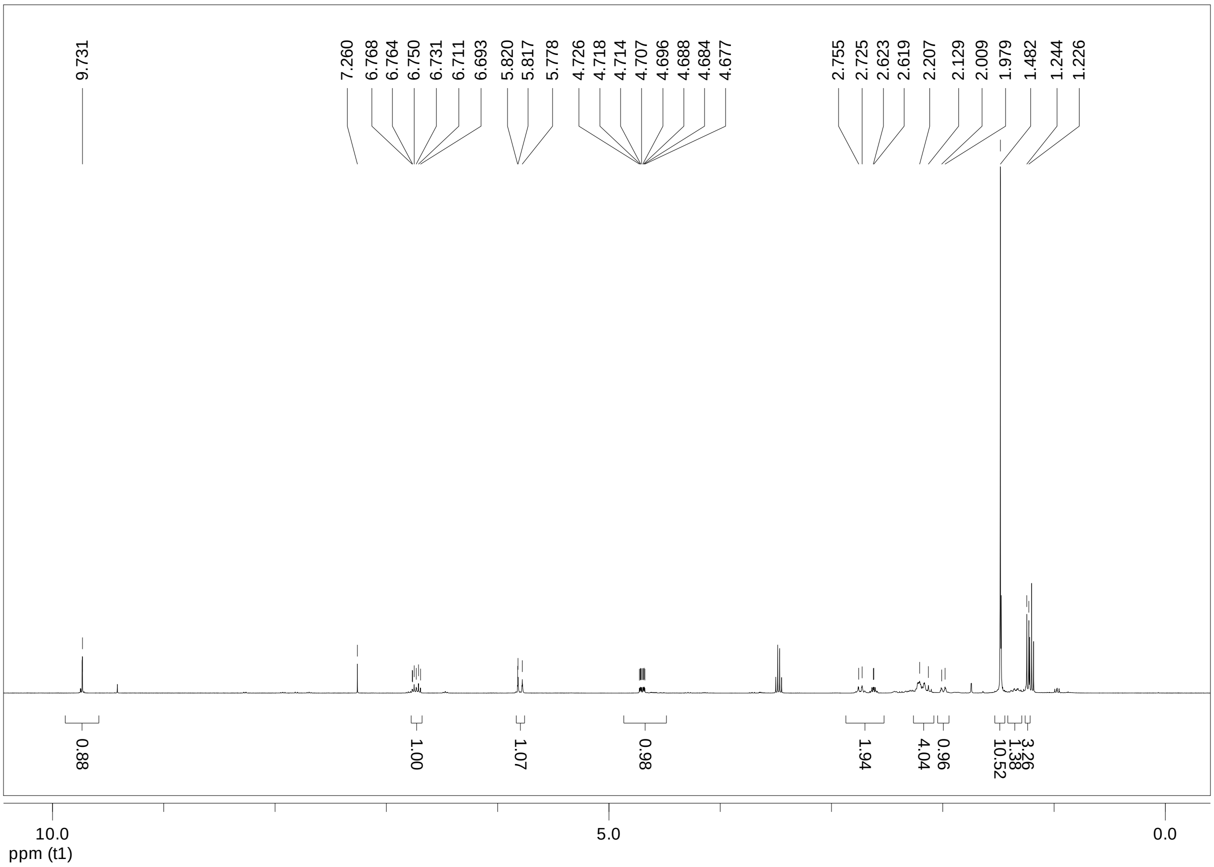
9.731
7.260
6.768
6.764
6.750
6.731
6.711
6.693
5.820
5.817
5.778
4.726
4.718
4.714
4.707
4.696
4.688
4.684
4.677
2.755
2.725
2.623
2.619
2.207
2.129
2.009
1.979
1.482
1.244
1.226
0.88
1.00
1.07
0.98
1.94
4.04
0.96
10.52
1.38
3.26
10.0
5.0
0.0
ppm (t1)

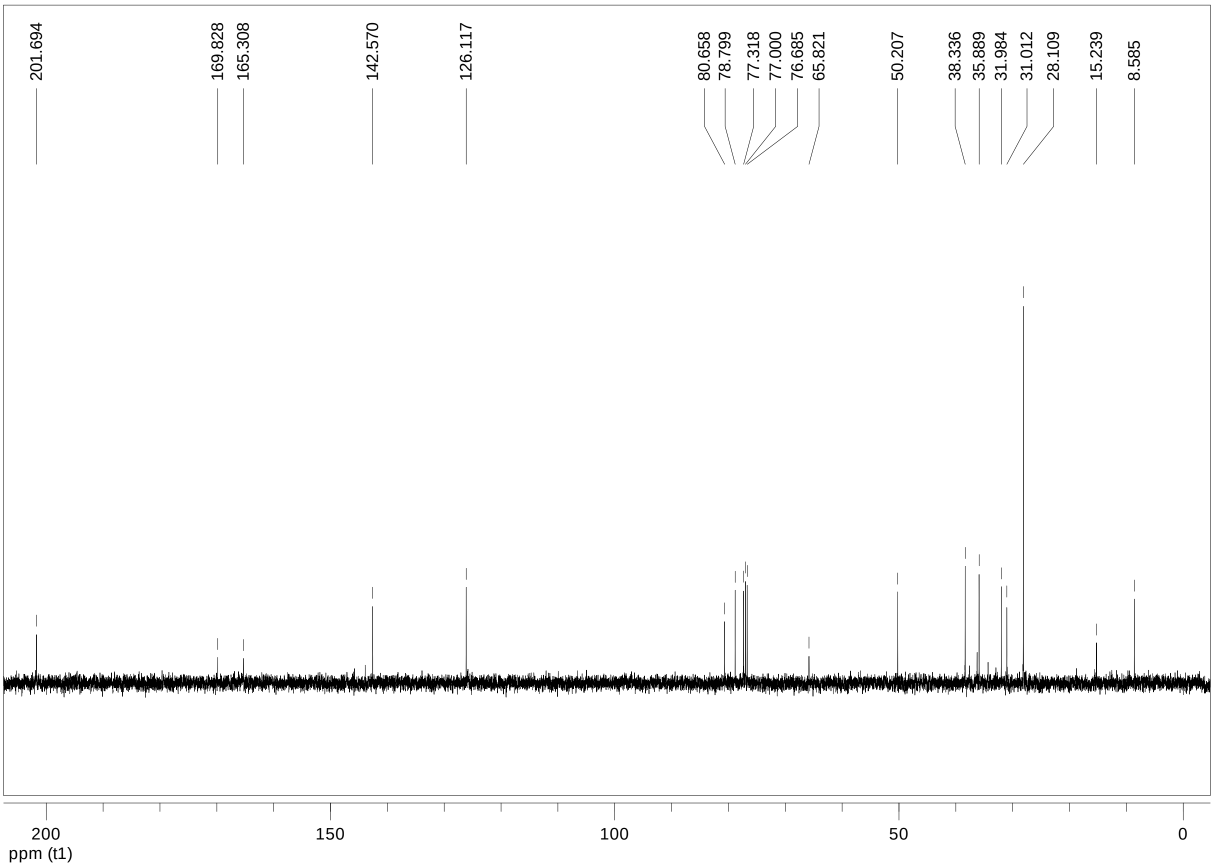
201.694
169.828
165.308
142.570
126.117
80.658
78.799
77.318
77.000
76.685
65.821
50.207
38.336
35.889
31.984
31.012
28.109
15.239
8.585
200
150
100
50
0
ppm (t1)

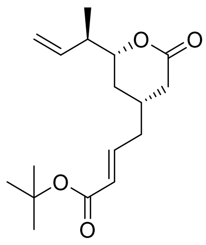

63

***tert*-Butyl (*E*)-4-((2*R*,4*R*)-2-((*R*)-but-3-en-2-yl)-6-oxotetrahydro-2H-pyran-4-yl)but-2-enoate (63):** To a solution of **61** (439 mg, 1.48 mmol, 1.0 equiv) and the *Julia* PT sulfone **62**[15] (432 mg, 1.93 mmol, 1.3 equiv) in THF (15 mL) was added at –78 °C solid NaHMDS (353 mg, 1.93 mmol, 1.3 equiv). After stirring the yellowish solution at –78 °C for 2 h, the reaction mixture was allowed to warm to rt over 2 h. A pH7 buffer solution (20 mL) was added at 0 °C and the mixture was diluted with Et_2O (40 mL). The layers were separated and the aqueous layer was extracted with Et_2O (2 x 30 mL). The combined organic extracts were washed with brine, dried over $MgSO_4$, filtered and concentrated under reduced pressure. The crude product was purified by column chromatography (hexane/EtOAc, 3:1) to afford **63** (294 mg) as a mixture with the Julia PT sulfone **62** (204 mg) (38% yield of **63**).

R*f* = 0.29 (hexane/EtOAc, 3:1)

^{1}H-NMR (400.1 MHz, $CDCl_3$): δ = 6.71 (td, *J* = 7.3, 15.5 Hz, 1H), 5.81-5.67 (m, 2H), 5.13-5.04 (m, 2H), 4.11 (ddd, *J* = 2.8, 6.2, 11.6 Hz, 1H), 2.66 (m_c, 1H), 2.42 (m_c, 1H), 2.26-2.13 (m, 2H), 2.12-1.98 (m, 2H), 1.93 (d, *J* = 13.0 Hz, 1H), 1.45 (s, 9H), 1.27-1.12 (m, 1H), 1.08 (d, *J* = 6.8 Hz, 3H).

^{13}C-NMR (100.6 MHz, $CDCl_3$): δ = 170.6, 165.3, 143.0, 138.4, 125.7, 116.3, 83.1, 80.4, 42.5, 38.4, 35.9, 31.7, 30.8, 28.0, 15.1.

HRMS (ESI): *m/z* calcd for $C_{17}H_{30}NO_4$ $[M + NH_4]^+$: 312.2169, found: 312.2166.

[15] Julia PT sulfone **62** was prepared according to: Lebrun, M.-E.; Le Marquand, P.; Berthelette, C. *J. Org. Chem.* **2006**, *71*, 2009-2013. See below for the experimental procedure.

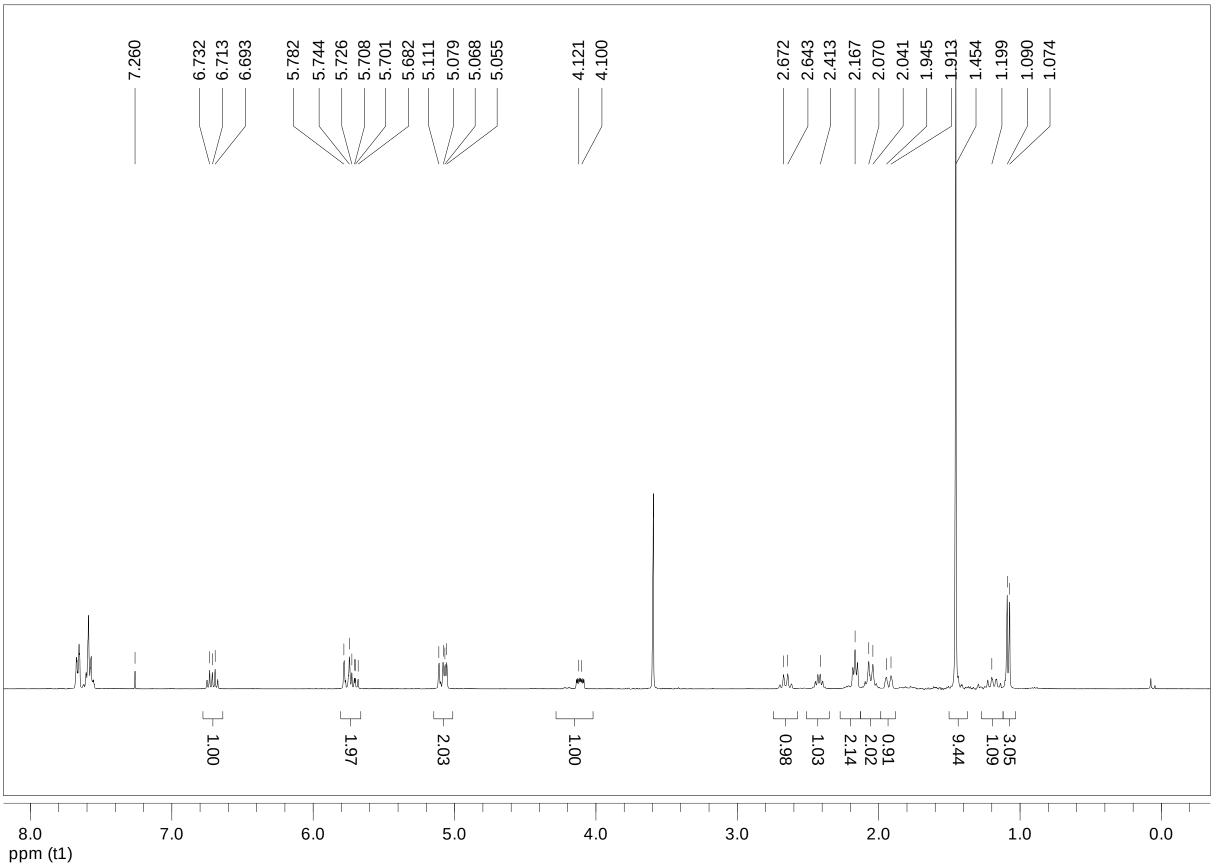
7.260
6.732
6.713
6.693
5.782
5.744
5.726
5.708
5.701
5.682
5.111
5.079
5.068
5.055
4.121
4.100
2.672
2.643
2.413
2.167
2.070
2.041
1.945
1.913
1.454
1.199
1.090
1.074
1.00
1.97
2.03
1.00
0.98
1.03
2.14
2.02
0.91
9.44
1.09
3.05
8.0
7.0
6.0
5.0
4.0
3.0
2.0
1.0
0.0
ppm (t1)

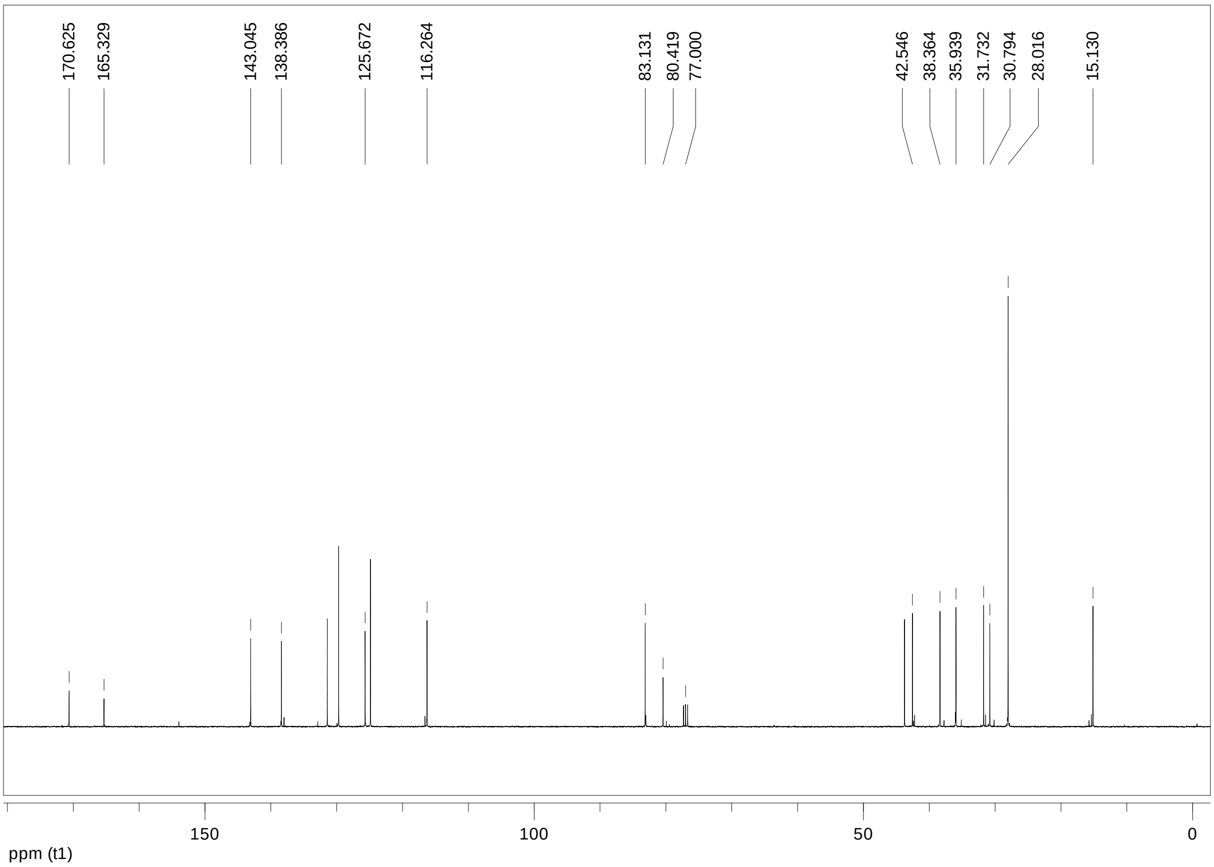
170.625
165.329
143.045
138.386
125.672
116.264
83.131
80.419
77.000
42.546
38.364
35.939
31.732
30.794
28.016
15.130
150
100
50
0
ppm (t1)

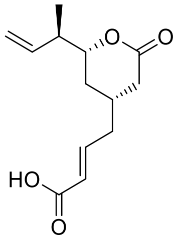

6

(*E*)-4-((2*R*,4*R*)-2-((*R*)-But-3-en-2-yl)-6-oxotetrahydro-2H-pyran-4-yl)but-2-enoic acid (6): To a solution 5 (284 mg, containing Julia PT sulfone) was added at 0 °C a solution of TFA in CH_2Cl_2 (1.0 M, 7.7 mL, ca. 14 equiv of TFA). The reaction mixture was stirred at rt for 17 h. Then, the reaction mixture was concentrated under reduced pressure and the crude purified by column chromatography (hexane/EtOAc, 1:1, then EtOAc/MeOH/AcOH, 30:1:0.5) to afford **6** (164 mg, quant.) as a brownish oil.

R_f = 0.54 (chloroform/MeOH/water/AcOH, 90:10:1.0:0.5)
$[\alpha]_D^{24}$ = +5.5° [*c* = 0.391, $CHCl_3$].
^{1}H-NMR (400.1 MHz, $CDCl_3$): δ = 11.0 (br s, 1H), 6.95 (m_c, 1H), 5.87 (d, *J* = 15.6 Hz, 1H), 5.73 (ddd, *J* = 7.7, 10.4, 17.8 Hz, 1H), 5.16-5.05 (m, 2H), 4.14 (ddd, *J* =2.9, 6.3, 11.7 Hz, 1H), 2.70 (q, *J* = 10.5 Hz, 1H), 2.44 (m_c, 1H), 2.34-2.19 (m, 2H), 2.17-2.02 (m, 2H), 1.95 (d, *J* = 13.2 Hz, 1H), 1.29-1.15 (m, 1H), 1.10 (d, *J* = 6.8 Hz, 3H).
^{13}C-NMR (100.6 MHz, $CDCl_3$): δ = 171.0 (2 x C=O), 147.4, 138.3, 123.3, 116.5, 83.3, 42.6, 38.6, 35.9, 31.7, 30.7, 15.2.
IR (neat, ν/cm^{-1}): 3450br, 3082w, 2924m, 1694s, 1657s, 1420m, 1386m, 1248s, 1204s, 1144m, 1077w, 994m, 921m, 892w, 843w.
HRMS (ESI): *m/z* calcd for $C_{13}H_{19}O_4$ $[M + H]^+$: 239.1278, found: 239.1277.

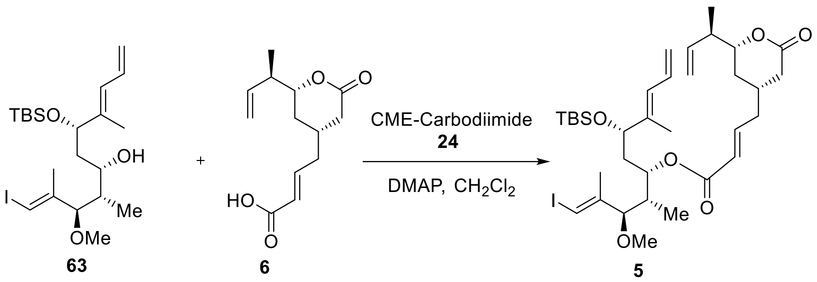

63 6 5

(1*E*,3*R*,4*S*,5*S*,7*S*,8*E*)-7-(*tert*-butyldimethylsilyloxy)-1-iodo-3-methoxy-2,4,8-trimethylundeca-1,8,10-trien-5-yl (*E*)-4-((2R,4R)-2-((*R*)-but-3-en-2-yl)-6-oxo-tetrahydro-2H-pyran-4-yl)but-2-enoate (5): To a cooled (0 °C) solution of acid **6** (10.1 mg, 42.4 μmol, 1.00 eq) in CH_2Cl_2 (0.5 mL) over molecular sieves (10 mg powder, 4 Å) was added CME-carbodiimide (22.3 mg, 52.6 μmol, 1.30 eq). The mixture was aged for 10 min, then a solution of the alcohol **30** (23.9 mg, 48.4 μmol, 1.14 eq) was added. After 20 min the cooling was removed and DMAP (0.5 mg, 10 mol%) was added. TLC after 8 h did not indicate any conversion. Therefore the mixture was stored in the freezer over the weekend. Afterwards it was concentrated under reduced pressure and the residue was purified by column chromatography (Hex:EtOAc 5:1→$CHCl_3$:MeOH:H_2O:CH_3COOH 90:10:1.0:0.5) to afford the desired **5** (4.1 mg, 14%) as a colourless oil along with the parent alcohol **30** (17.3 mg, 72%). No acid **6** could be recovered.

For analytical data see below.

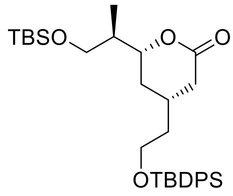

64

(4*R*,6*R*)-6-((*R*)-1-(*tert*-butyldimethylsilyloxy)propan-2-yl)-4-(2-((*tert*-butyl-diphenylsilyl)oxy)ethyl)tetrahydro-2H-pyran-2-one (64): A stirred solution of **57** (1.94 g, 6.12 mmol, 1.0 equiv) in dry DMF (5 mL) was treated at rt with imidazole (500 mg, 7.35 mmol, 1.2 equiv) followed by TBDPSCl (1.90 mL, 7.35 mmol, 1.2 equiv). After stirring at rt for 18 h, the reaction mixture was diluted with EtOAc (80 mL). The resulting mixture was washed with brine (3 x 20 mL), dried over $MgSO_4$ and concentrated *in vacuo*. The residue was purified by column chromatography (hexane/EtOAc, 10:1) to give **64** (3.22 g, 95%) as a colorless oil.

R_f = 0.24 (hexane/EtOAc, 10:1)

$[\alpha]_D^{24}$ = –10.0° [*c* = 0.366, $CHCl_3$]

^{1}H-NMR (400.1 MHz, $CDCl_3$): δ = 7.65 (d, *J* = 6.7 Hz, 4H), 7.48-7.33 (m, 6H), 4.42 (td, *J* = 3.0, 12.0 Hz, 1H), 3.71 (t, *J* =6.1 Hz, 2H), 3.67-3.59 (m, 1H), 3.59-3.50 (m, 1H), 2.65 (dd, *J* =5.1, 17.4 Hz, 1H), 2.18 (m_c, 1H), 2.04 (dd, *J* = 10.7, 17.4 Hz, 1H), 1.86-1.71 (m, 2H), 1.62-1.48 (m, 2H), 1.39-1.20 (m, 1H), 1.05 (s, 9H), 0.92 (d, *J* =6.9 Hz, 3H), 0.89 (s, 9H), 0.05 (s, 6H).

^{13}C-NMR (100.6 MHz, $CDCl_3$): δ = 171.6, 135.5, 133.6, 129.8, 127.7, 80.2, 64.3, 60.8, 40.3, 38.9, 36.5, 32.6, 28.7, 26.9, 25.9, 19.2, 18.3, 10.7, -5.4.

IR (neat, ν/cm^{-1}): 3071w, 2954m, 2929m, 2857m, 1738s, 1472m, 1428m, 1389m, 1250m, 1108s, 836s, 778m, 741m, 702s, 613m, 504m.

HRMS (ESI): *m/z* calcd for $C_{32}H_{54}NO_4Si_2$ $[M + NH_4]^+$: 572.3586, found: 572.3581.

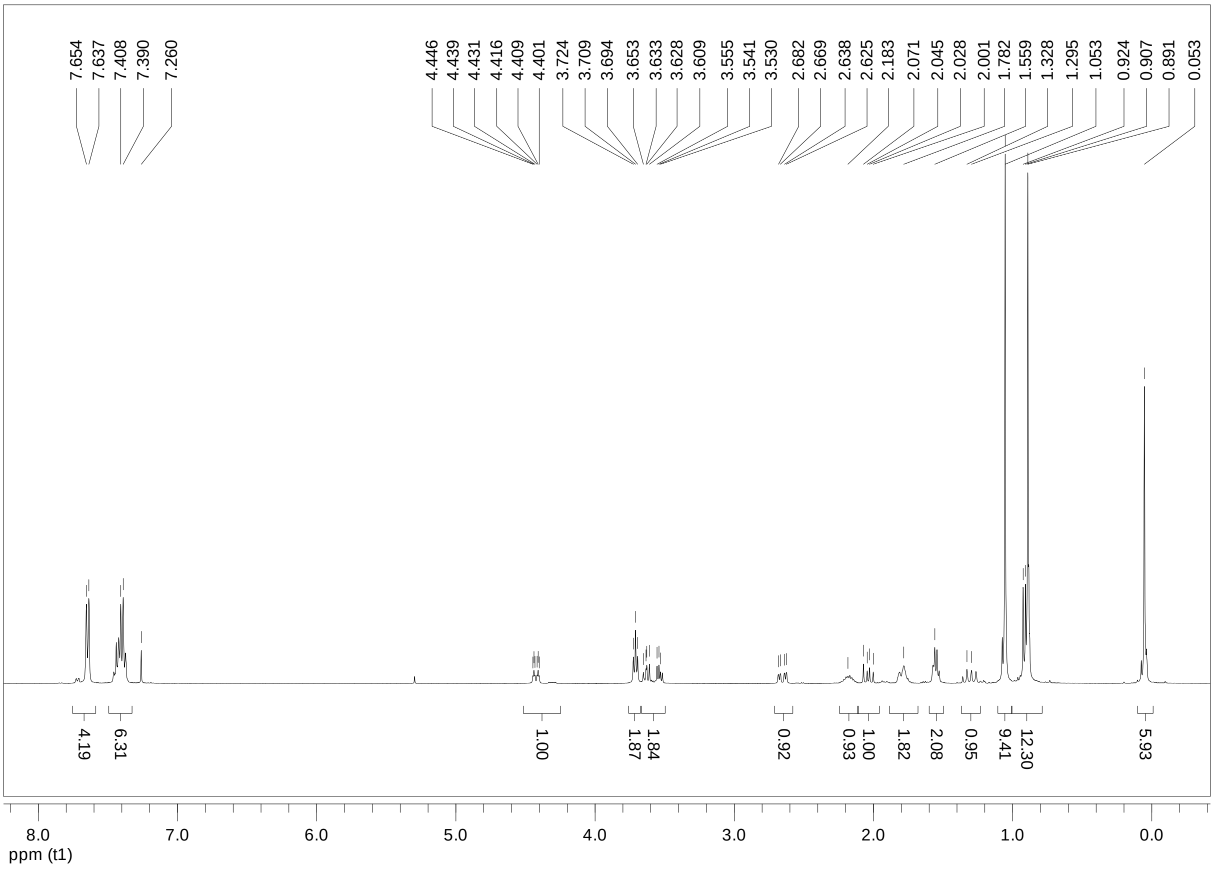
7.654
7.637
7.408
7.390
7.260
4.446
4.439
4.431
4.416
4.409
4.401
3.724
3.709
3.694
3.653
3.633
3.628
3.609
3.555
3.541
3.530
2.682
2.669
2.638
2.625
2.183
2.071
2.045
2.028
2.001
1.782
1.559
1.328
1.295
1.053
0.924
0.907
0.891
0.053
4.19
6.31
1.00
1.87
1.84
0.92
0.93
1.00
1.82
2.08
0.95
9.41
12.30
5.93
8.0
7.0
6.0
5.0
4.0
3.0
2.0
1.0
0.0
ppm (t1)

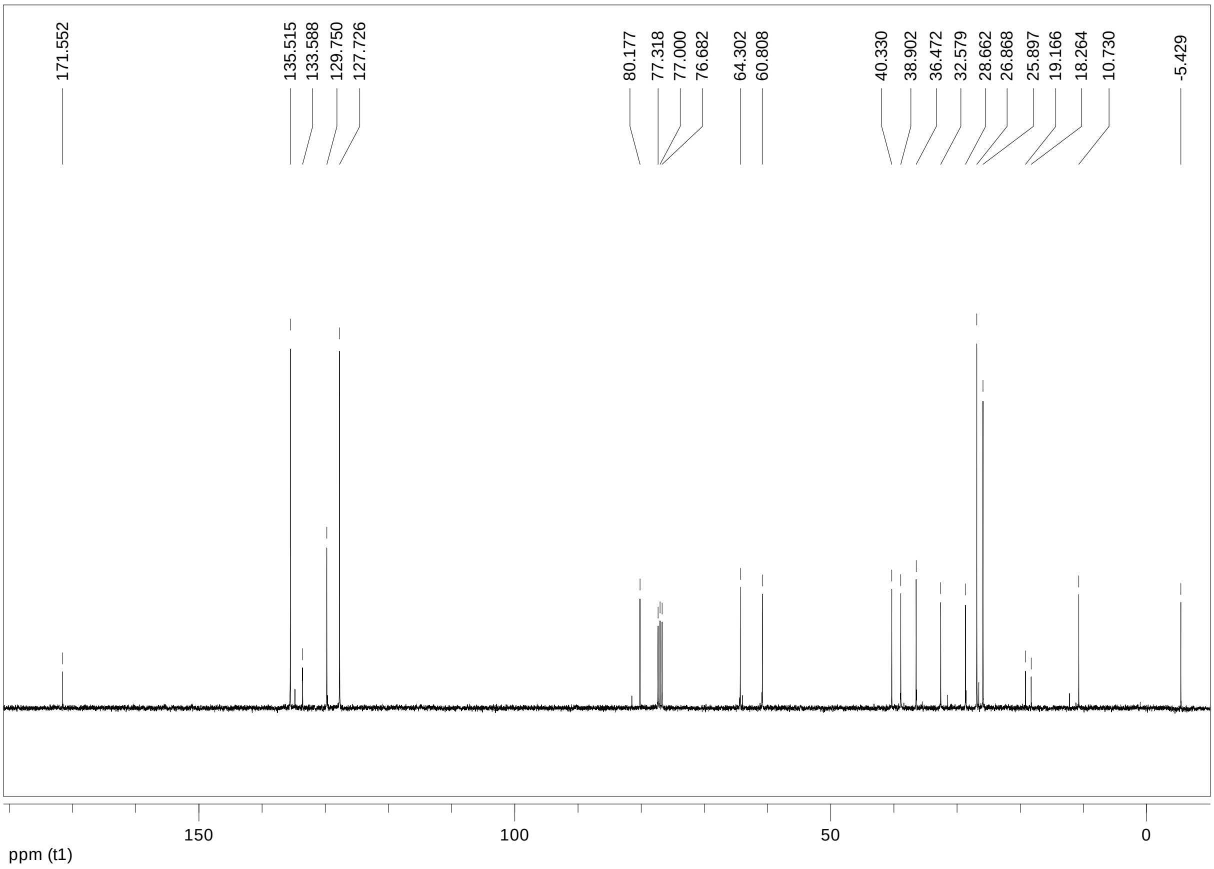
171.552
135.515
133.588
129.750
127.726
80.177
77.318
77.000
76.682
64.302
60.808
40.330
38.902
36.472
32.579
28.662
26.868
25.897
19.166
18.264
10.730
-5.429
150
100
50
0
ppm (t1)

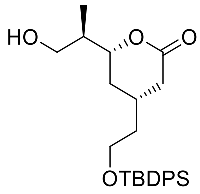

65

(4*R*,6*R*)-4-(2-((*tert*-Butyldiphenylsilyl)oxy)ethyl)-6-((*R*)-1-hydroxypropan-2-yl)tetrahydro-2H-pyran-2-one (65): To a solution of **64** (1.52 g, 2.74 mmol, 1.0 equiv) in THF/water (4:1, 28 mL) was added sodium periodate (3.51 g, 16.4 mmol, 6.0 equiv). After stirring at rt for 20 h, the reaction mixture was quenched with water (40 mL) and diluted with EtOAc (40 mL). The layers were separated and the aqueous layer was extracted with EtOAc (2 x 50 mL). The combined organic extracts were washed with brine, dried over $MgSO_4$ and concentrated under reduced pressure. The residue was purified by column chromatography (hexane/EtOAc, 2:1 → 1:1) to give **65** (995 mg, 82%) as a colorless solid. The analytical data were identical to those reported in literature.[16]

R_f = 0.33 (hexane/EtOAc, 1:1)

^{1}H-NMR (400.1 MHz, $CDCl_3$): δ = 7.68-7.61 (m, 4H), 7.47-7.35 (m, 6H), 4.48 (td, *J* = 3.0, 12.0 Hz, 1H), 3.76-3.67 (m, 1H), 3.71 (t, *J* = 6.1 Hz, 2H), 3.65-3.55 (m, 1H), 2.67 (ddd, *J* = 1.8, 5.8, 17.5 Hz, 1H), 2.18 (m_c, 1H), 2.11-1.98 (m, 1H), 1.91-1.73 (m, 2H), 1.71-1.59 (m, 1H), 1.56 (dd, *J* = 6.3, 12.6 Hz, 2H), 1.40-1.23 (m, 1H), 1.05 (s, 9H), 0.94 (d, *J* = 7.0 Hz, 3H).

^{13}C-NMR (100.6 MHz, $CDCl_3$): δ = 171.4, 135.5, 133.5, 129.8, 127.7, 80.5, 64.4, 60.8, 39.8, 38.8, 36.4, 32.3, 28.6, 26.9, 19.2, 10.5.

HRMS (ESI): *m/z* calcd for $C_{26}H_{40}NO_4Si$ $[M + NH_4]^+$: 458.2721, found: 458.2723.

[16] Mitchell, I. S.; Pattenden, G.; Stonehouse, J. *Org. Biomol. Chem.* **2005**, *3*, 4412-4431.

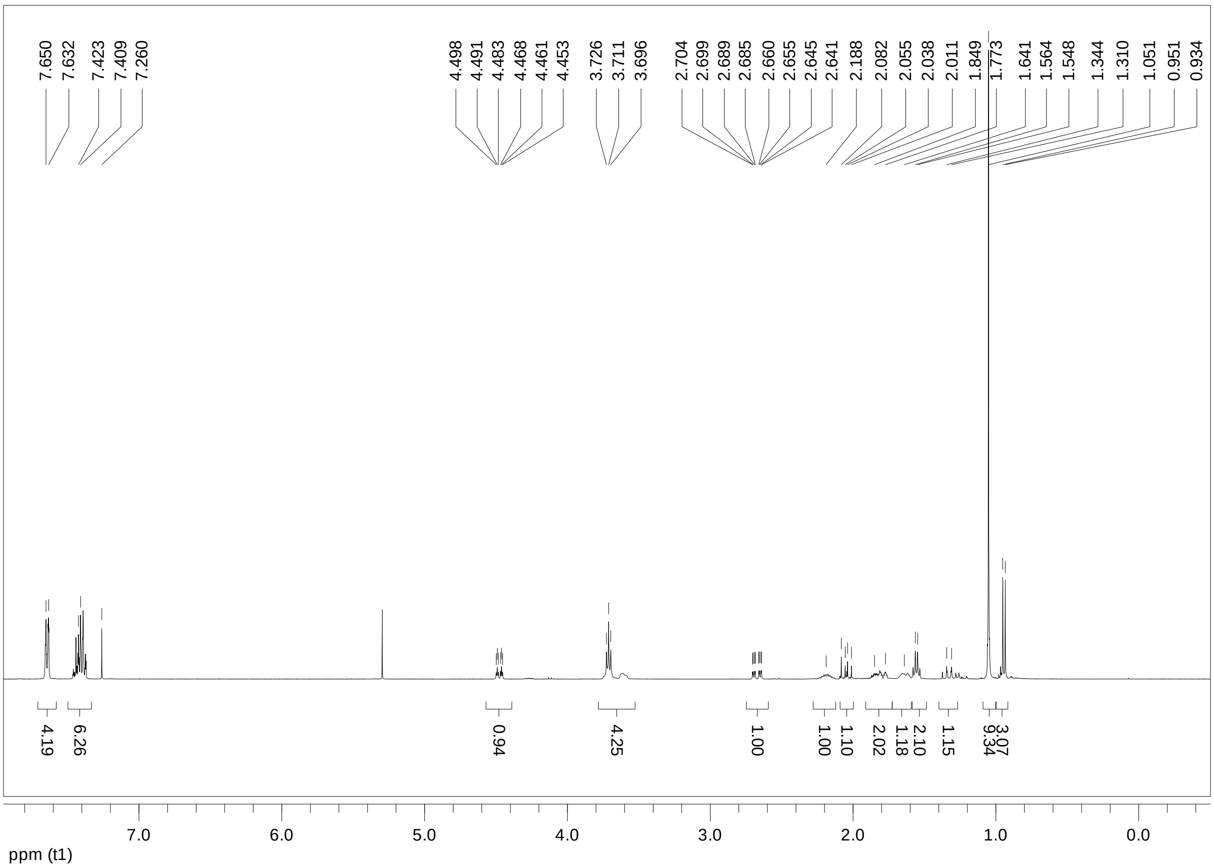
7.650 7.632 7.423 7.409 7.260
4.498 4.491 4.483 4.468 4.461 4.453
3.726 3.711 3.696
2.704 2.699 2.689 2.685 2.660 2.655 2.645 2.641 2.188 2.082 2.055 2.038 2.011 1.849 1.773 1.641 1.564 1.548 1.344 1.310 1.051 0.951 0.934
4.19 6.26 0.94 4.25 1.00 1.00 1.10 2.02 1.18 2.10 1.15 9.34 3.07
7.0 6.0 5.0 4.0 3.0 2.0 1.0 0.0
ppm (t1)

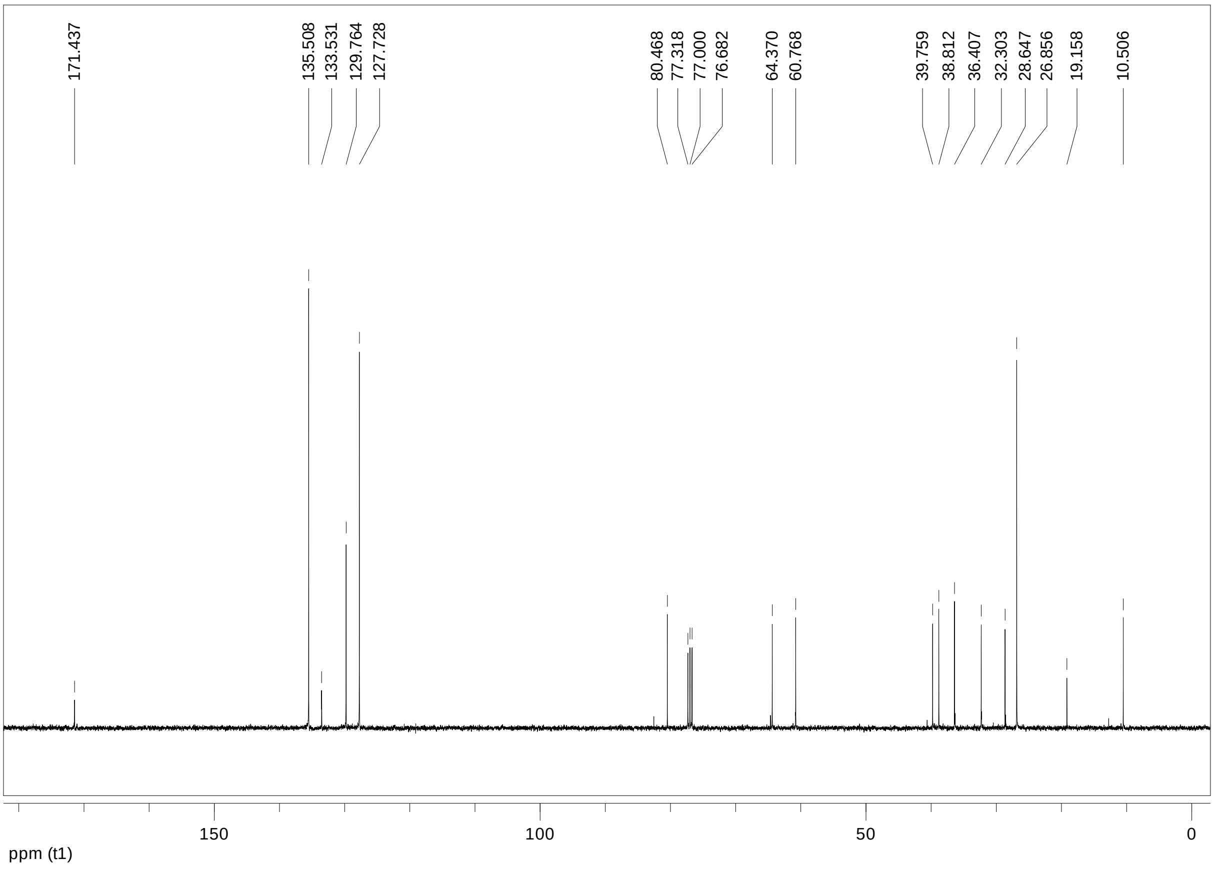
171.437
135.508 133.531 129.764 127.728
80.468 77.318 77.000 76.682
64.370 60.768
39.759 38.812 36.407 32.303 28.647 26.856 19.158 10.506
150 100 50 0
ppm (t1)

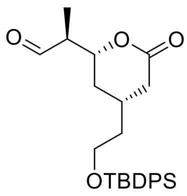

66

(*S*)-2-((2*R*,4*R*)-4-(2-((*tert*-Butyldiphenylsilyl)oxy)ethyl)-6-oxotetrahydro-2H-pyran-2-yl)propanal (66): To a solution of **65** (333 mg, 0.76 mmol, 1.0 equiv) in CH_2Cl_2 (15.1 mL) cooled at 0 °C was added *Dess-Martin* periodinane (385 mg, 0.91 mmol, 1.2 equiv) in one portion. After stirring for 2 h at rt, sat. $Na_2S_2O_3$ (20 mL), sat. $NaHCO_3$ (20 mL) and Et_2O (50 mL) were added at rt to the reaction mixture. The layers were separated and the aqueous layer was extracted with Et_2O (2 x 25 mL). The combined organic extracts were washed with sat. $NaHCO_3$ (20 mL) and brine, dried over $MgSO_4$, filtered and concentrated under reduced pressure. The crude product **66** (388 mg) was used for the step without further purification, due to decomposition on silica gel. The analytical data were in agreement with those reported in the literature.[17]

R*f* = 0.78 (hexane/EtOAc, 1:1)
^{1}H-NMR (400.1 MHz, $CDCl_3$): δ = 9.73 (d, *J* = 0.8 Hz, 1H), 7.67-7.60 (m, 4H), 7.48-7.35 (m, 6H), 4.65 (dd, *J* = 2.9, 4.4, 12.0 Hz, 1H), 3.71 (t, *J* = 5.9 Hz, 2H), 2.69 (ddd, *J* = 1.8, 5.7, 17.5 Hz, 1H), 2.56 (m_c, 1H), 2.22 (m_c, 1H), 2.07 (dd, *J* = 10.9, 17.6 Hz, 1H), 1.88 (m_c, 1H), 1.55 (dd, *J* = 6.3, 12.4 Hz, 2H), 1.27 (m_c, 1H), 1.20 (d, *J* = 7.2 Hz, 3H), 1.05 (s, 9H).
^{13}C-NMR (100.6 MHz, $CDCl_3$): δ = 202.0, 170.4, 135.5, 133.5, 133.4, 129.8, 127.8, 79.1, 60.6, 50.2, 38.6, 36.2, 32.2, 28.5, 26.9, 19.2, 8.5.
HRMS (ESI): *m/z* calcd for $C_{26}H_{34}NaO_4Si$ $[M + Na]^+$: 461.2119, found: 461.2118.

[17] Mitchell, I. S.; Pattenden, G.; Stonehouse, J. *Org. Biomol. Chem.* **2005**, *3*, 4412-4431.

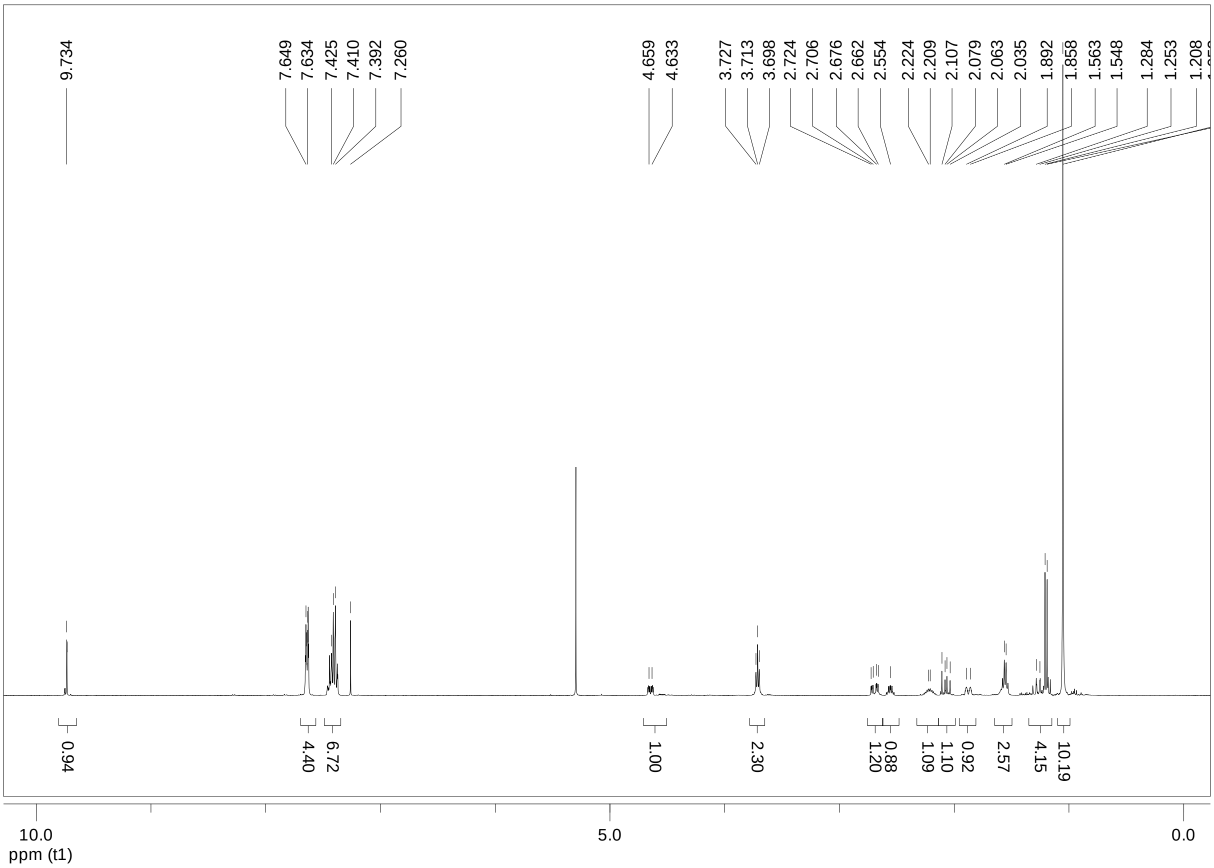
9.734
7.649
7.634
7.425
7.410
7.392
7.260
4.659
4.633
3.727
3.713
3.698
2.724
2.706
2.676
2.662
2.554
2.224
2.209
2.107
2.079
2.063
2.035
1.892
1.858
1.563
1.548
1.284
1.253
1.208
0.94
4.40
6.72
1.00
2.30
1.20
0.88
1.09
1.10
0.92
2.57
4.15
10.19
10.0
5.0
0.0
ppm (t1)

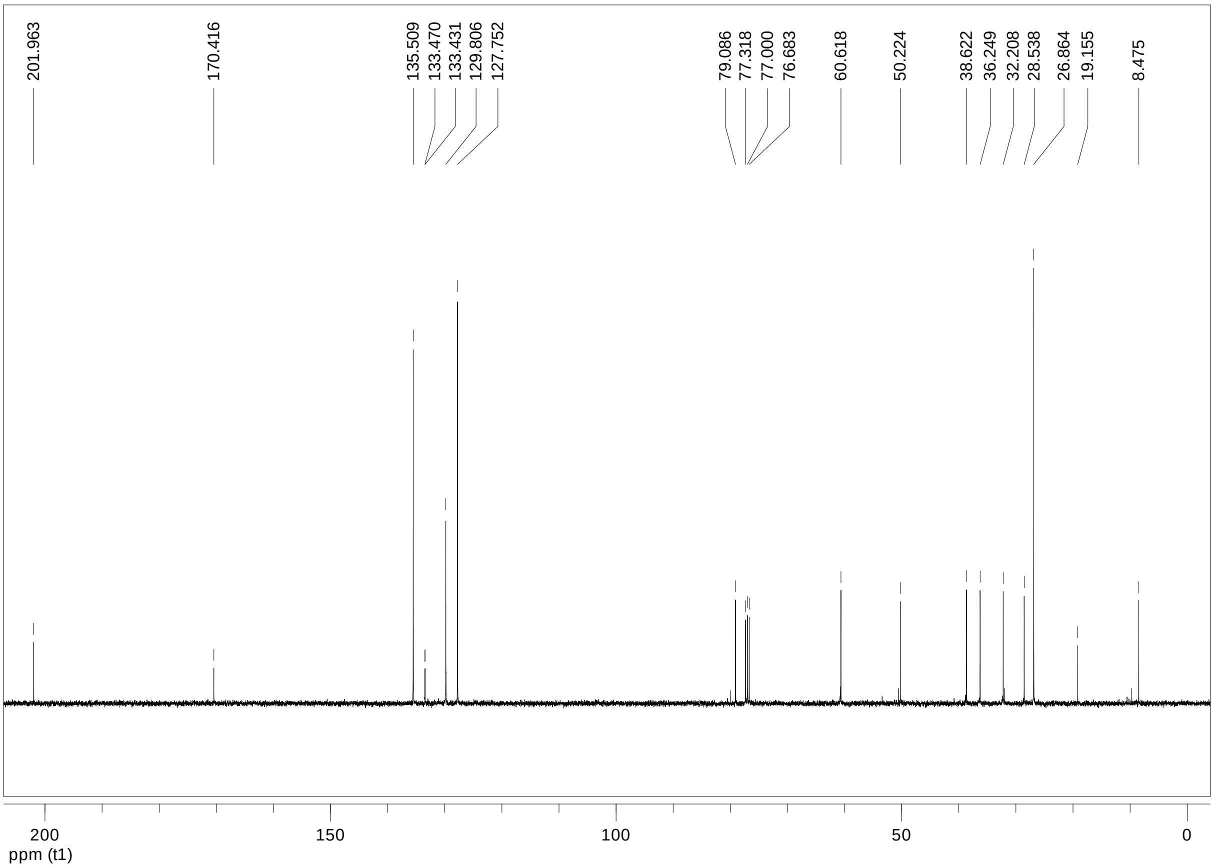
201.963
170.416
135.509
133.470
133.431
129.806
127.752
79.086
77.318
77.000
76.683
60.618
50.224
38.622
36.249
32.208
28.538
26.864
19.155
8.475
200
150
100
50
0
ppm (t1)

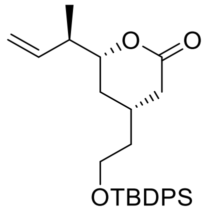

67

(4*R*,6*R*)-6-((*R*)-but-3-en-2-yl)-4-(2-((*tert*-butyldiphenylsilyl)oxy)ethyl)tetra-hydro-2H-pyran-2-one (67): To a solution of **66** (388 mg, crude) and the Julia PT sulfone **62**[18] (220 mg, 0.98 mmol, 1.3 equiv) in THF (7.7 mL) was added at –78 °C solid NaHMDS (180 mg, 0.98 mmol, 1.3 equiv). After stirring the yellowish solution at –78 °C for 2 h, the reaction mixture was allowed to warm to rt over 2.25 h. pH7 buffer solution (20 mL) was added and the mixture was diluted with Et_2O (30 mL). The layers were separated and the aqueous layer was extracted with Et_2O (2 x 30 mL). The combined organic extracts were washed with brine, dried over $MgSO_4$, filtered and concentrated under reduced pressure. The crude product was purified by column chromatography (hexane/EtOAc, 10:1 → 7:1) to afford **67** (216 mg, 65% over 2 steps) as a colorless oil.

R*f* = 0.43 (hexane/EtOAc, 5:1)

$[\alpha]_D^{24}$ = +5.2° [*c* = 0.360, $CHCl_3$]

^{1}H-NMR (400.1 MHz, $CDCl_3$): δ = 7.67-7.60 (m, 4H), 7.47-7.35 (m, 6H), 5.74 (ddd, *J* = 7.7, 10.4, 17.3 Hz, 1H), 5.14-5.04 (m, 2H), 4.10 (ddd, *J* = 2.9, 6.2, 11.8 Hz, 1H), 3.70 (t, *J* = 6.0 Hz, 2H), 2.65 (ddd, *J* = 1.8, 5.4, 17.1 Hz, 1H), 2.42 (m_c, 1H), 2.13 (m_c, 1H), 2.03 (dd, *J* = 10.9, 17.1 Hz, 1H), 1.91 (m_c, 1H), 1.59-1.50 (m, 2H), 1.21-1.07 (m, 1H), 1.10 (d, *J* = 6.8 Hz, 3H), 1.05 (s, 9H).

^{13}C-NMR (100.6 MHz, $CDCl_3$): δ = 171.3, 138.7, 135.5, 133.5, 129.8, 127.7, 116.1, 83.5, 60.8, 42.7, 38.7, 36.4, 32.2, 28.5, 26.9, 19.2, 15.3.

IR (neat, ν/cm^{-1}): 3072w, 2958m, 2929m, 2857m, 1738s, 1472w, 1428m, 1388w, 1234m, 1110s, 1008w, 822m, 745m, 703s, 613w.

HRMS (ESI): *m/z* calcd for $C_{27}H_{40}NO_3Si$ [M + NH_4]$^+$: 454.2772, found: 454.2761.

---

[18] Julia PT sulfone **62** was prepared according to: Lebrun, M.-E.; Le Marquand, P.; Berthelette, C. *J. Org. Chem.* **2006**, *71*, 2009-2013. See below for the experimental procedure.

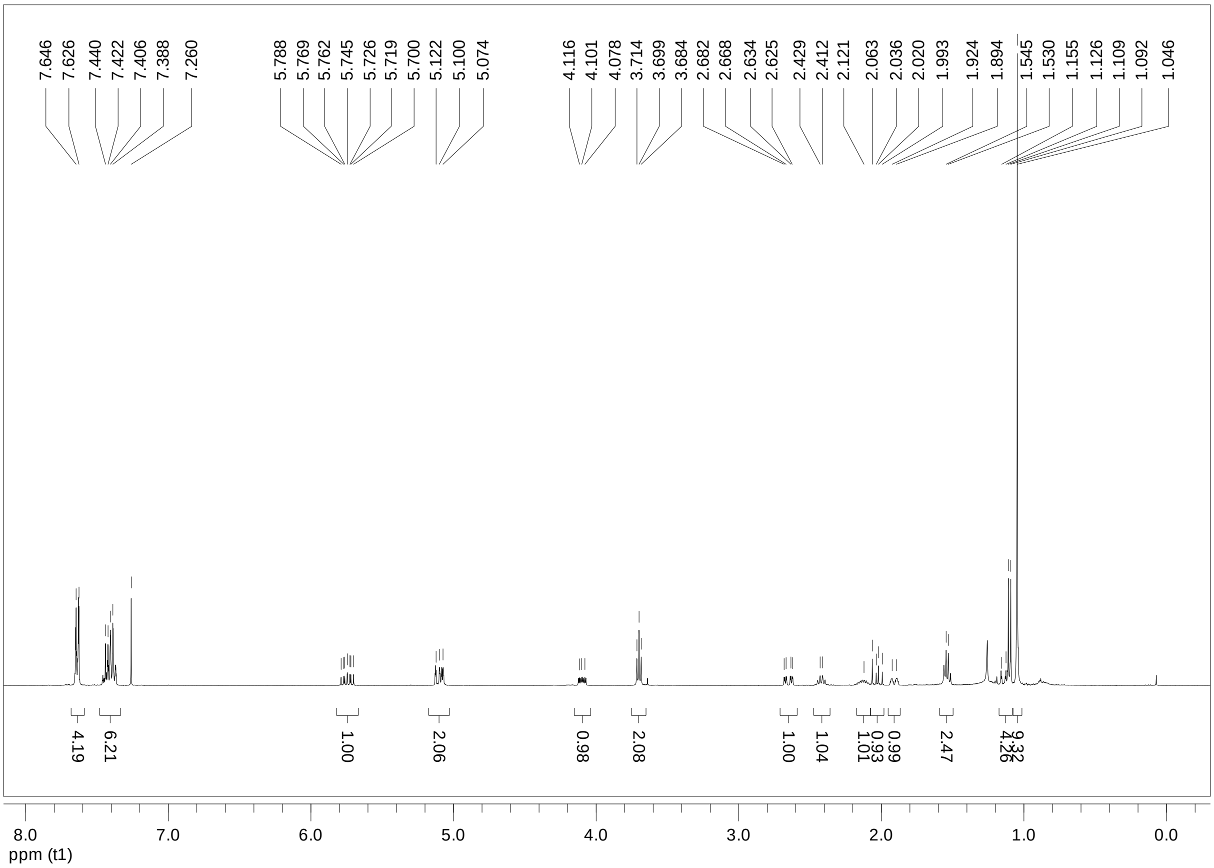
7.646 7.626 7.440 7.422 7.406 7.388 7.260
5.788 5.769 5.762 5.745 5.726 5.719 5.700 5.122 5.100 5.074
4.116 4.101 4.078 3.714 3.699 3.684 2.682 2.668 2.634 2.625 2.429 2.412 2.121 2.063 2.036 2.020 1.993 1.924 1.894 1.545 1.530 1.155 1.126 1.109 1.092 1.046
4.19 6.21 1.00 2.06 0.98 2.08 1.00 1.04 1.01 0.93 0.99 2.47 4.26 9.32
8.0 7.0 6.0 5.0 4.0 3.0 2.0 1.0 0.0
ppm (t1)

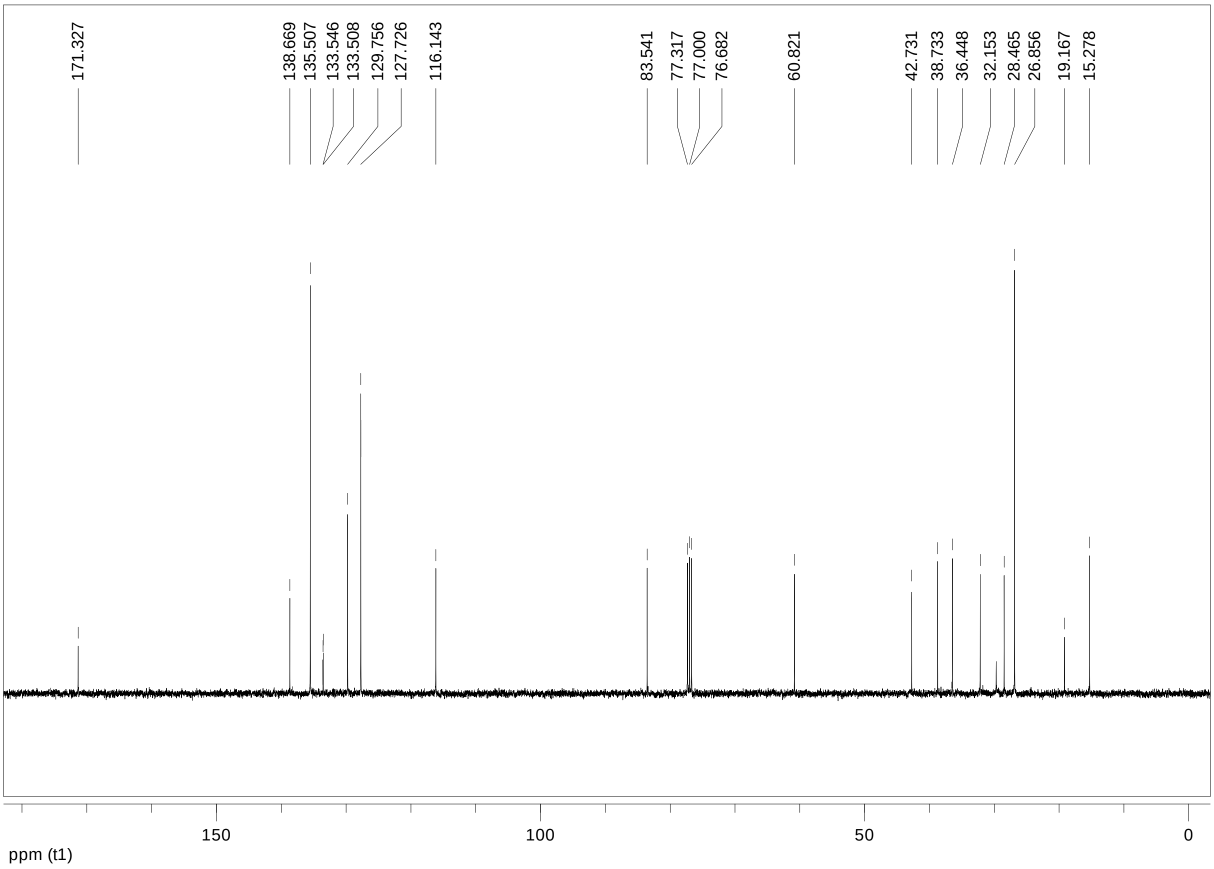
171.327
138.669 135.507 133.546 133.508 129.756 127.726 116.143
83.541 77.317 77.000 76.682
60.821
42.731 38.733 36.448 32.153 28.465 26.856 19.167 15.278
150 100 50 0
ppm (t1)

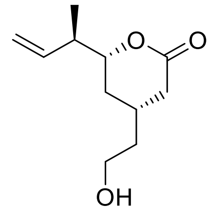

S14

(4*R*,6*R*)-6-((*R*)-But-3-en-2-yl)-4-(2-hydroxyethyl)tetrahydro-2H-pyran-2-one (S14): To a solution of **67** (34.4 mg, 0.08 mmol, 1.0 equiv) in THF (0.8 mL) was added at 0 °C TBAF (0.32 mL, 0.32 mmol, 4.0 equiv, 1.0 M in THF) and AcOH (18.1 µL, 0.32 mmol, 4.0 equiv). After stirring for 20 h at rt, the reaction mixture was quenched with sat. NH_4Cl (10 mL) and diluted with EtOAc (15 mL). The layers were separated and the aqueous layer was extracted with EtOAc (2 x 15 mL). The combined organic extracts were washed with brine, dried over $MgSO_4$ and the solvent was removed under reduced pressure. The residue was purified by column chromatography (hexane/EtOAc, 1:2 → 0:1) to give **S14** as a colorless oil (13.6 mg, 87%).

R*f* = 0.38 (EtOAc)

$[\alpha]_D^{24}$ = +14.0° [*c* = 0.595, $CHCl_3$]

^{1}H-NMR (400.1 MHz, $CDCl_3$): δ = 5.69 (m_c, 1H), 5.10-4.99 (m, 2H), 4.09 (ddd, *J* = 2.9, 6.3, 11.8 Hz, 1H), 3.65 (t, *J* = 6.3 Hz, 2H), 2.67 (m_c, 1H), 2.39 (m_c, 1H), 2.16-1.97 (m, 2H), 1.96-1.87 (m, 1H), 1.67 (br s, 1H), 1.51 (m_c, 2H), 1.26-1.07 (m, 1H), 1.05 (d, *J* = 6.8 Hz, 3H).

^{13}C-NMR (100.6 MHz, $CDCl_3$): δ = 171.4, 138.6, 116.2, 83.5, 59.6, 42.7, 38.8, 36.3, 32.1, 28.2, 15.3.

IR (neat, ν/cm^{-1}): 3420br, 2966m, 2923m, 1714s, 1383m, 1245s, 1175w, 1089m, 1053s, 1011m, 921m, 699w.

HRMS (ESI): *m/z* calcd for $C_{11}H_{19}O_3$ [M + H]$^+$: 199.1329, found: 199.1326.

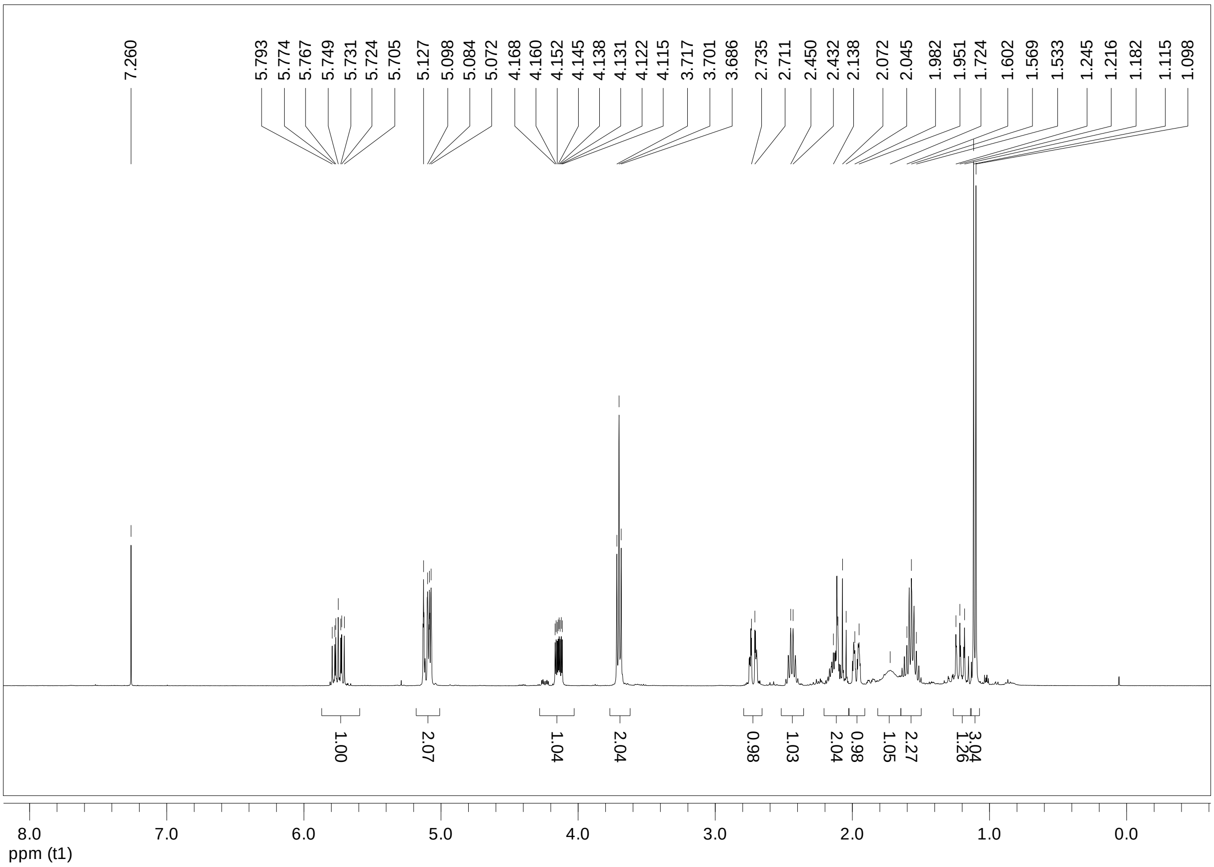
7.260
5.793
5.774
5.767
5.749
5.731
5.724
5.705
5.127
5.098
5.084
5.072
4.168
4.160
4.152
4.145
4.138
4.131
4.122
4.115
3.717
3.701
3.686
2.735
2.711
2.450
2.432
2.138
2.072
2.045
1.982
1.951
1.724
1.602
1.569
1.533
1.245
1.216
1.182
1.115
1.098
1.00
2.07
1.04
2.04
0.98
1.03
2.04
0.98
1.05
2.27
1.26
3.04
8.0
7.0
6.0
5.0
4.0
3.0
2.0
1.0
0.0
ppm (t1)

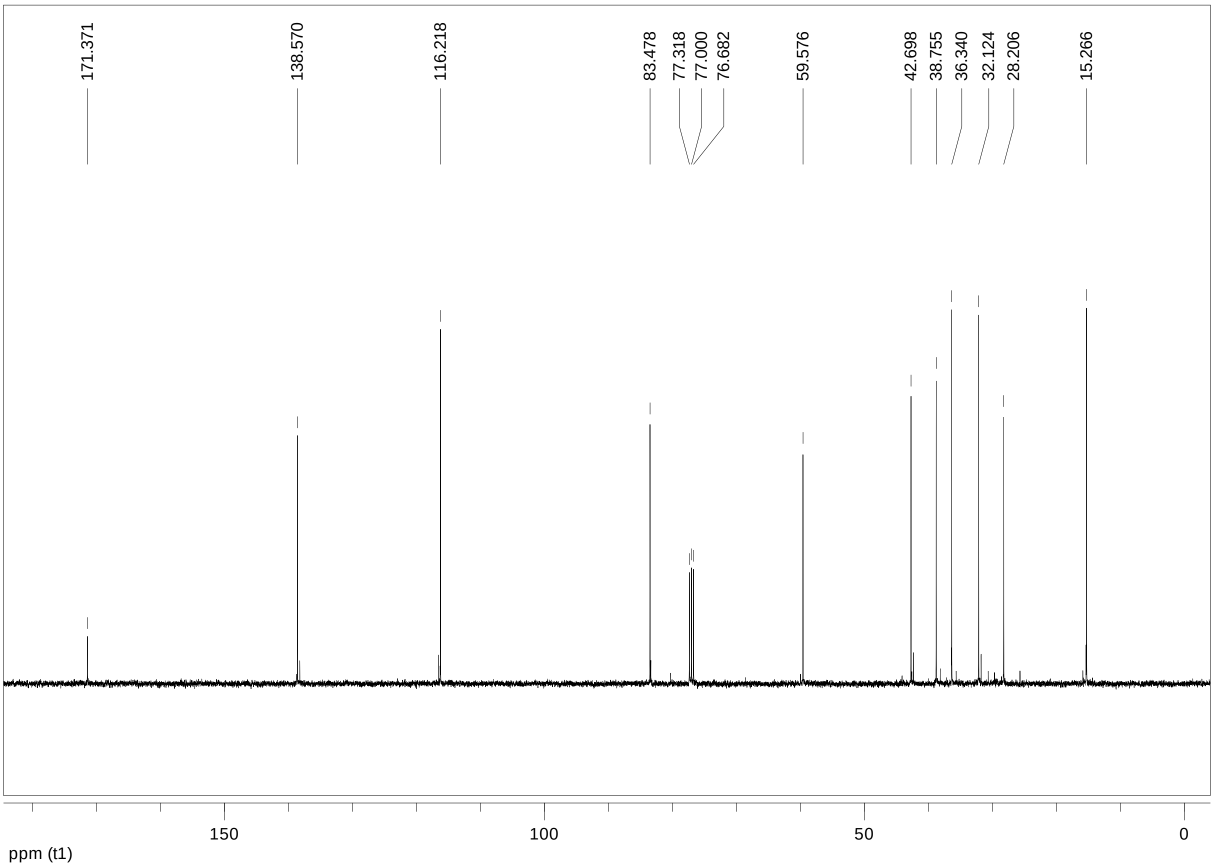
171.371
138.570
116.218
83.478
77.318
77.000
76.682
59.576
42.698
38.755
36.340
32.124
28.206
15.266
150
100
50
0
ppm (t1)

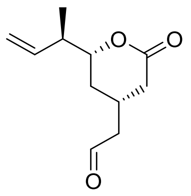

68

2-((2*R*,4*S*)-2-((*R*)-But-3-en-2-yl)-6-oxotetrahydro-2H-pyran-4-yl)acetaldehyde (68): To a solution of oxalyl chloride (0.05 mL, 0.61 mmol, 1.5 equiv) in CH_2Cl_2 (5.0 mL) at –78 °C was added dropwise DMSO (0.09 mL, 1.22 mmol, 3.0 equiv). After stirring at –78 °C for 10 min, a solution of **S14** (80.6 mg, 0.41 mmol, 1.0 equiv) in CH_2Cl_2 (2.1 mL) was added dropwise. The resultant cloudy mixture was stirred at –78 °C for 1.5 h, and then TEA (0.23 mL, 1.63 mmol, 4.0 equiv) was added slowly and the reaction mixture was allowed to warm to room temperature (1 h). The reaction was quenched with water (10 mL), and the layers were separated. The aqueous phase was extracted with CH_2Cl_2 (3 x 20 mL) and the combined organic phases were washed with brine, dried over $MgSO_4$, filtered and concentrated *in vacuo*. The crude product was purified by column chromatography (hexane/EtOAc, 2:1 → 1:1 → 1:2) to afford **68** (76.0 mg, 95%) as a colorless oil.

R_f = 0.59 (EtOAc)

$[\alpha]_D^{24}$ = +4.8° [*c* = 0.535, $CHCl_3$]

^{1}H-NMR (400.1 MHz, $CDCl_3$): δ = 9.76 (s, 1H), 5.73 (ddd, *J* = 7.7, 10.4, 17.3 Hz, 1H), 5.14-5.05 (m, 2H), 4.17 (ddd, *J* = 2.9, 6.3, 11.8 Hz, 1H), 2.81-2.70 (m, 1H), 2.60-2.48 (m, 3H), 2.44 (m_c, 1H), 2.15-2.04 (m, 1H), 2.00 (m_c, 1H), 1.30-1.14 (m, 1H), 1.11 (d, *J* =6.8 Hz, 3H).

^{13}C-NMR (100.6 MHz, $CDCl_3$): δ = 199.6, 170.3, 138.3, 116.4, 83.1, 49.8, 42.6, 35.8, 31.8, 25.9, 15.2.

IR (neat, ν/cm^{-1}): 2974m, 2922m, 1723s, 1385m, 1238s, 1174w, 1080m, 1053m, 1006m, 923m.

HRMS (ESI): *m/z* calcd for $C_{11}H_{17}O_3$ $[M + H]^+$: 197.1172, found: 197.1173.

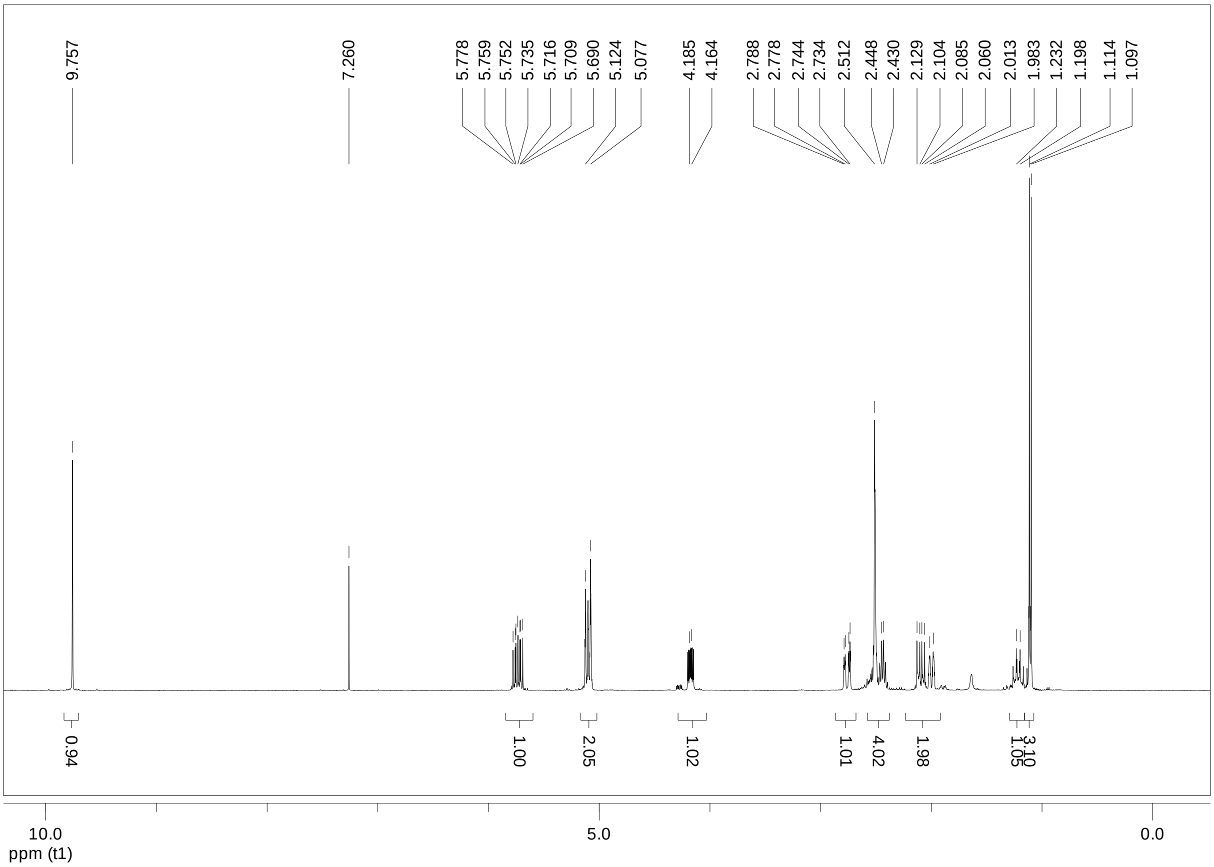
9.757
7.260
5.778
5.759
5.752
5.735
5.716
5.709
5.690
5.124
5.077
4.185
4.164
2.788
2.778
2.744
2.734
2.512
2.448
2.430
2.129
2.104
2.085
2.060
2.013
1.983
1.232
1.198
1.114
1.097
0.94
1.00
2.05
1.02
1.01
4.02
1.98
1.05
3.10
10.0
5.0
0.0
ppm (t1)

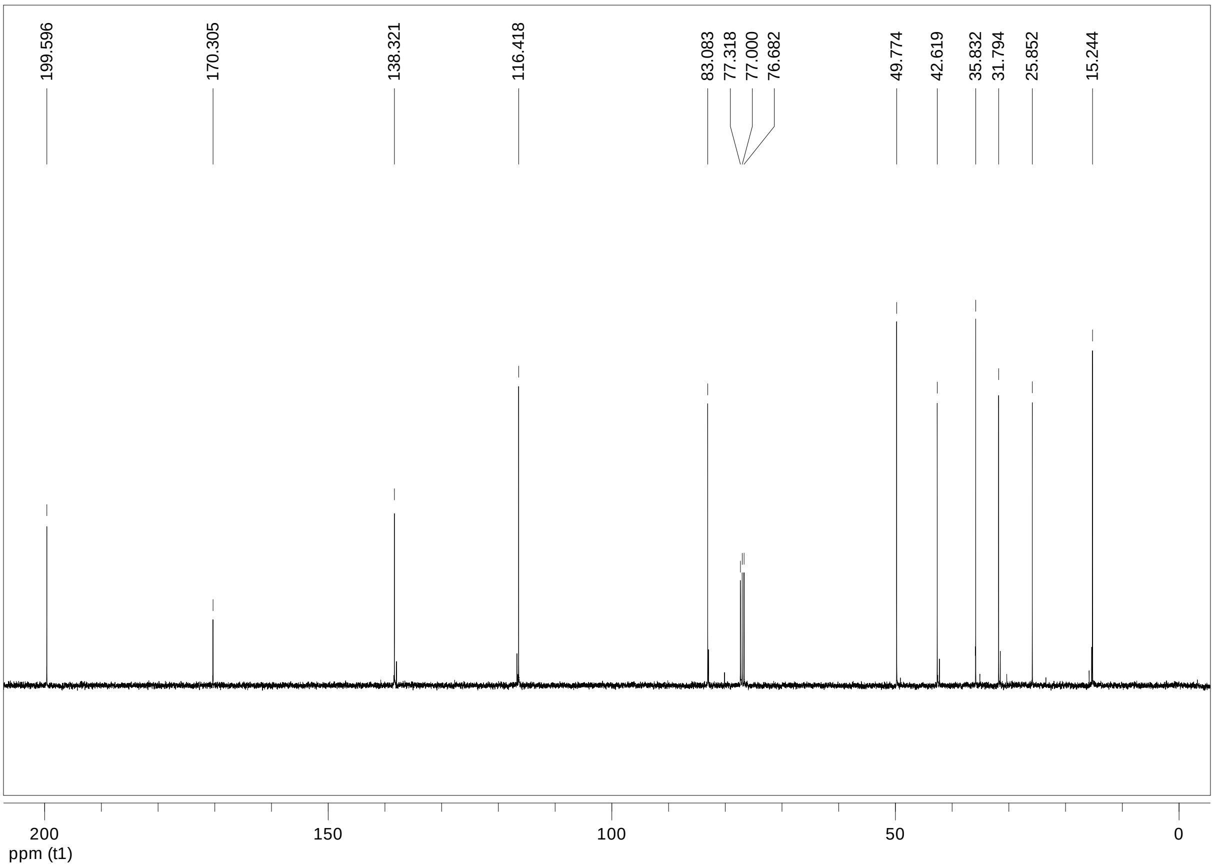
199.596
170.305
138.321
116.418
83.083
77.318
77.000
76.682
49.774
42.619
35.832
31.794
25.852
15.244
200
150
100
50
0
ppm (t1)

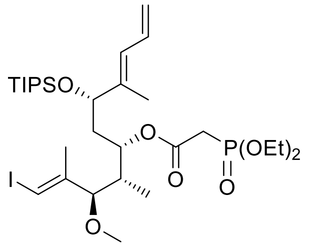

69

(1*E*,3*R*,4*S*,5*S*,7*S*,8*E*)-1-Iodo-3-methoxy-2,4,8-trimethyl-7-((triisopropylsilyl)oxy)-undeca-1,8,10-trien-5-yl 2-(diethoxyphosphoryl)acetate (69): To a cooled (0 °C) solution of diethylphosphonoacetic acid (**55**) (0.178 mL, 1.11 mmol, 1.50 eq) in CH_2Cl_2 (10 mL) over molecular sieves (beads, 3 Å) was added CME-carbodiimide (563 mg, 1.33 mmol, 1.80 eq). The mixture was stirred at 0 °C for 10-15 min, then a solution of the alcohol **30** (396 mg, 0.738 mmol, 1.00 eq) in CH_2Cl_2 (4 mL, rinsed with 2×3 mL) was added. The cooling was removed and DMAP (9 mg, 10 mol%) was added a few minutes later. A precipitate had formed already by the time of the DMAP addition. TLC reaction control after 45 min showed full conversion. After 1 h, the mixture was transferred into a flask and concentrated under reduced pressure. The crude was purified by column chromatography (hex:EtOAc 2:1→1:1) to afford the desired ester **69** (458 mg, 87%) as a colourless oil, which crystallized upon storage in a freezer.

TLC (Hex:EtOAc 1:1): R_f = 0.48

$[\alpha]_D^{24}$ = +29.1 (c = 1.26, $CHCl_3$)

^{1}H-NMR ($CDCl_3$, 400.1 MHz): δ = 6.50 (m_c, 1H), 6.17 (m_c, 1H), 5.87 (dd, *J* = 10.9 Hz, *J* = 0.5 Hz, 1H), 5.18 (dd, *J* = 1.8 Hz, *J* = 16.9 Hz, 1H), 5.08 (dd, *J* = 1.8 Hz, *J* = 10.2 Hz, 1H), 4.77 (ddd, *J* = 1.5 Hz, *J* = 4.5 Hz, *J* = 10.3 Hz, 1H), 4.18-4.07 (m, 5H), 3.43 (d, *J* = 6.7 Hz, 1H), 3.17 (s, 3H), 2.81 (dd, *J* = 14.3 Hz, *J* = 28.7 Hz, 1H), 2.76 (dd, *J* = 14.3 Hz, *J* = 28.8 Hz, 1H), 2.00 (ddd, *J* = 4.6 Hz, *J* = 6.9 Hz, *J* = 6.9 Hz, 1H), 1.90-1.84 (m, 1H), 1.81 (d, *J* = 1.1 Hz, 3H), 1.73 (d, *J* = 1.1 Hz, 3H), 1.69 (ddd, *J* = 1.5 Hz, *J* = 5.9 Hz, *J* = 14.6 Hz, 1H), 1.33 (2×t, *J* = 7.1 Hz, 6H), 1.06-0.98 (m, 21H), 0.93 (d, *J* = 6.9 Hz, 3H)/

^{13}C-NMR ($CDCl_3$, 100.6 MHz): δ = 164.9 (d, *J* = 6.4 Hz), 146.4, 140.4, 132.4, 126.3, 117.4, 87.0, 79.0, 77.0, 73.8, 62.52 (d, *J* = 6.2 Hz), 62.48 (d, *J* = 6.3 Hz), 56.7, 39.1, 35.7, 34.5 (d, *J* = 134 Hz), 19.8, 18.1, 18.0, 16.34 (d, *J* = 6.1 Hz), 16.30 (d, *J* = 6.1 Hz), 12.4, 11.4, 9.7.

IR (ν/[cm^{-1}]) 2964, 2942, 2893, 2866, 1734, 1463, 1382, 1261 (s), 1089, 1050 (s), 1025 (s), 988, 967, 905, 882, 810, 785, 736, 681, 653, 621, 572, 506, 459, 446, 424; **HRMS** Calcd. for $C_{30}H_{60}INO_7PSi$ $[M+NH_4]^+$ *m/z* 732.2916. Found: 732.2921.

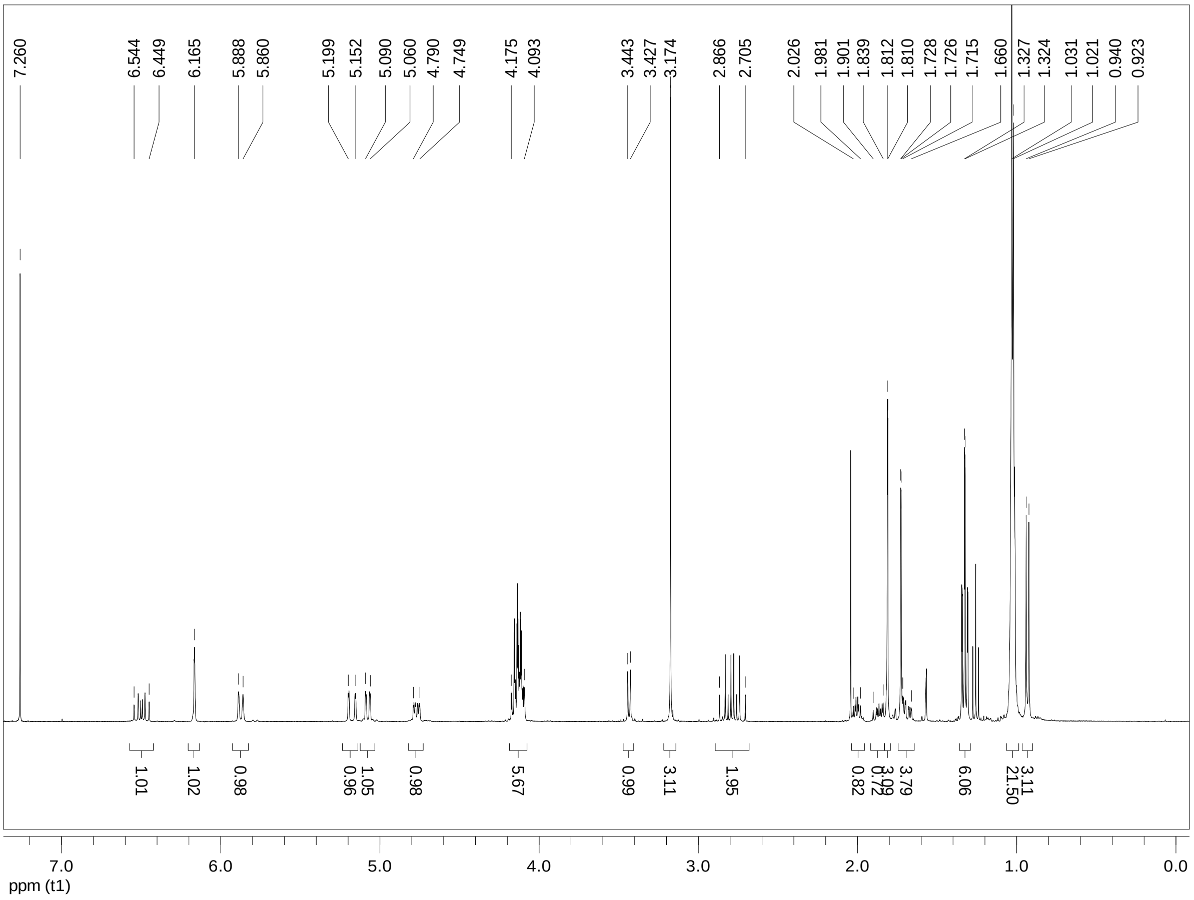

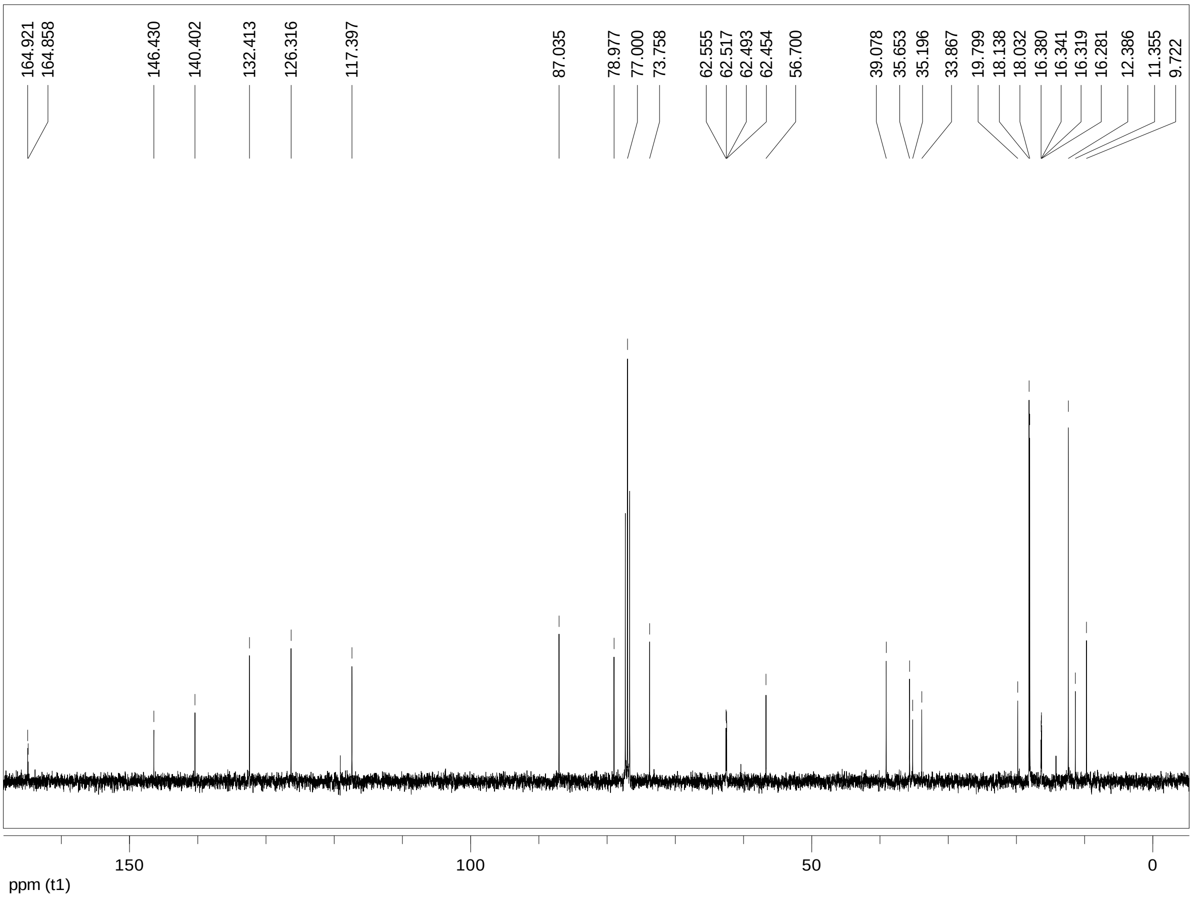

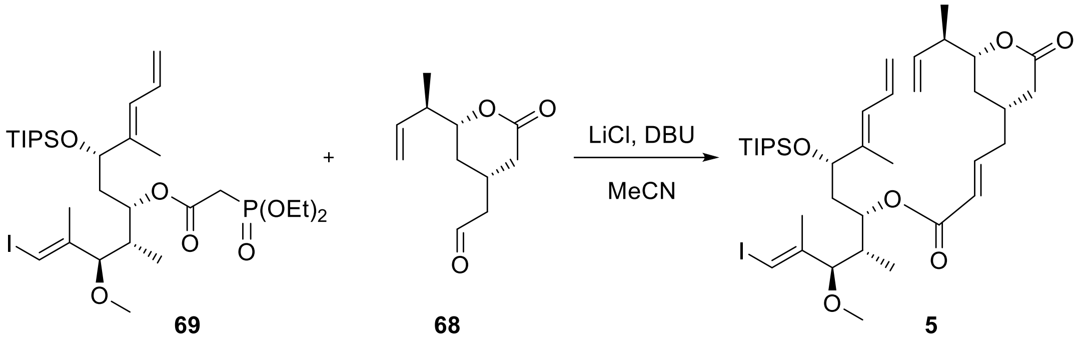

69 68 5

(1*E*,3*R*,4*S*,5*S*,7*S*,8*E*)-1-iodo-3-methoxy-2,4,8-trimethyl-7-((triisopropylsilyl)oxy)-undeca-1,8,10-trien-5-yl (*E*)-4-((2*R*,4*R*)-2-((*R*)-but-3-en-2-yl)-6-oxotetrahydro-2H-pyran-4-yl)but-2-enoate (5): To LiCl (9.19 mg, 217 µmol, 1.20 eq) was added a solution of the phosphonate **69** (155 mg, 217 µmol, 1.20 eq) in MeCN (1.1 mL), followed by DBU (30.0 µL, 199 µmol, 1.10 eq). After 20 min stirring at rt, the mixture was cooled to 0 °C, whereupon the solution turned turbid. Then a solution of the aldehyde **68** (35.5 mg, 181 µmol, 1.00 eq) in MeCN (1.7 mL) was added dropwise. TLC and ESI/MS-control after 45 min indicated, that no aldehyde was left. After 1 h the mixture was concentrated at the rotavap and the residue was purified by flash chromatography (hex:EtOAc 5:1→2:1) to afford the desired α,β-unsaturated ester **5** (127.5 mg, 92% wt/wt along with EtOAc, 117 mg, 85%) as a colourless oil.

TLC (hex:EtOAc 1:1): R_f = 0.79

[α]$_D^{24}$ = +22.5 (*c* = 0.96, $CHCl_3$)

^{1}H-NMR ($CDCl_3$, 400.1 MHz): δ = 6.75 (m_c, 1H), 6.43 (m_c, 1H), 6.16 (m_c, 1H), 5.88 (pseudo d, *J* = 10.8 Hz, 1H), 5.80-5.69 (m, 2H), 5.17-5.06 (m, 3H), 5.02 (dd, *J* = 1.8 Hz, *J* = 10.2 Hz, 1H), 4.78 (ddd, *J* = 1.6 Hz, *J* = 4.1 Hz, *J* = 10.2 Hz, 1H), 4.17-4.10 (m, 2H), 3.38 (d, *J* = 7.1 Hz, 1H), 3.16 (s, 3H), 2.69 (m_c, 1H), 2.45 (m_c, 1H), 2.26-2.14 (m, 2H), 2.14-2.01 (m, 3H), 2.00-1.91 (m, 1H), 1.90-1.78 (m, 1H), 1.83 (d, *J* = 1.0 Hz, 3H), 1.76-1.65 (m, 1H), 1.71 (d, *J* = 1.1 Hz, 3H), 1.27-1.14 (m, 1H), 1.12 (d, *J* = 6.8 Hz, 3H), 1.06-0.97 (m, 21H), 0.91 (d, *J* = 6.9 Hz, 3H)

^{13}C-NMR ($CDCl_3$, 100.6 MHz): δ = 170.6, 165.2, 146.5, 144.3, 140.3, 138.4, 132.6, 126.4, 124.2, 116.9, 116.5, 87.6, 83.2, 79.0, 76.6, 72.5, 56.7, 42.7, 39.0, 38.6, 36.0, 35.7, 31.8, 31.0, 19.7, 18.2, 18.1, 15.3, 12.4, 11.6, 9.8.

IR (ν/[cm^{-1}]) 3016, 2963, 2941, 2892, 2866, 1718, 1656, 1462, 1420, 1381, 1329, 1311, 1247, 1217, 1203, 1157, 1082, 1054, 1028, 1009, 989, 919, 906, 883, 809, 752s, 680, 666, 601, 510, 475, 463, 445, 432, 418, 405.

HRMS Calcd. for $C_{37}H_{65}INO_6Si$ $[M+NH_4]^+$ *m/z* 774.3620. Found: 774.3614.

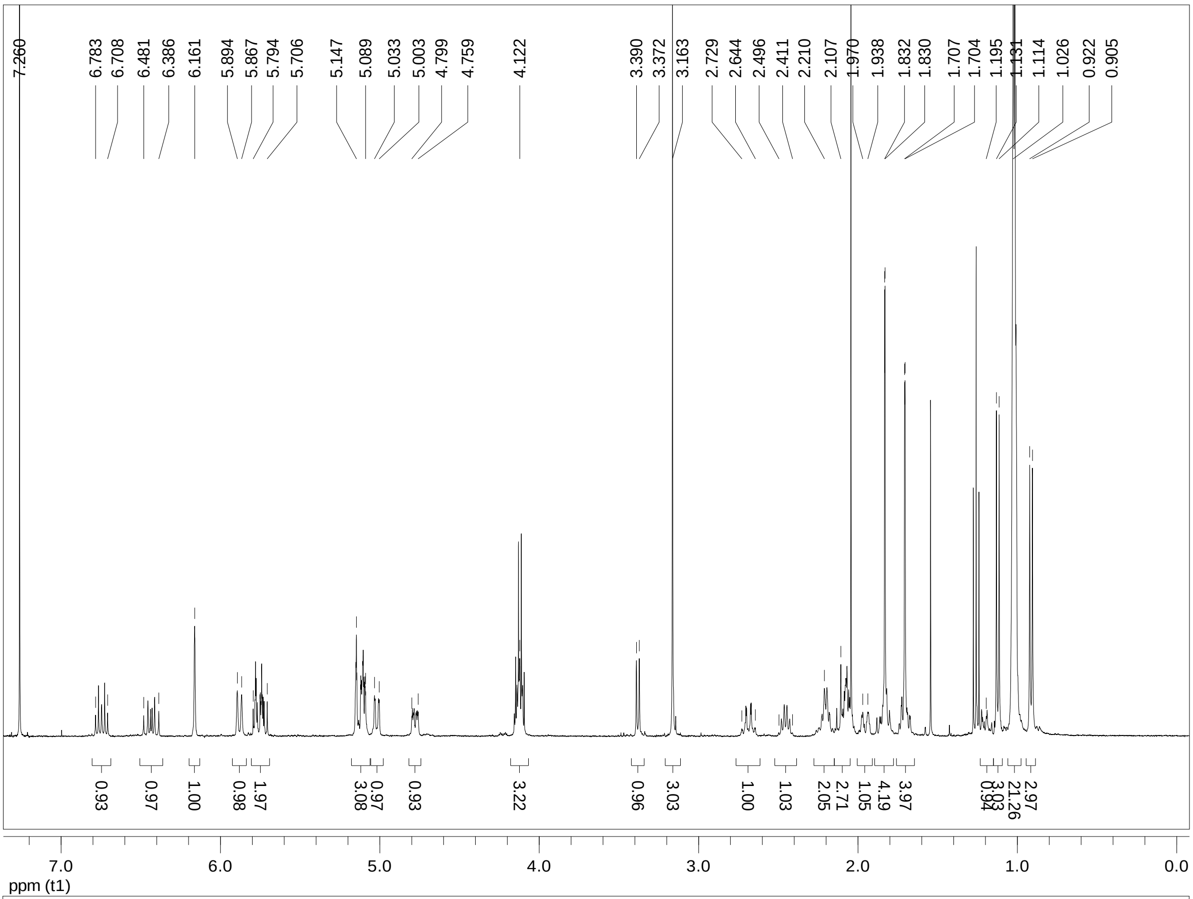
7.260
6.783
6.708
6.481
6.386
6.161
5.894
5.867
5.794
5.706
5.147
5.089
5.033
5.003
4.799
4.759
4.122
3.390
3.372
3.163
2.729
2.644
2.496
2.411
2.210
2.107
1.970
1.938
1.832
1.830
1.707
1.704
1.195
1.131
1.114
1.026
0.922
0.905
0.93
0.97
1.00
0.98
1.97
3.08
0.97
0.93
3.22
0.96
3.03
1.00
1.03
2.05
2.71
1.05
4.19
3.97
0.94
3.03
21.26
2.97
7.0
6.0
5.0
4.0
3.0
2.0
1.0
0.0
ppm (t1)

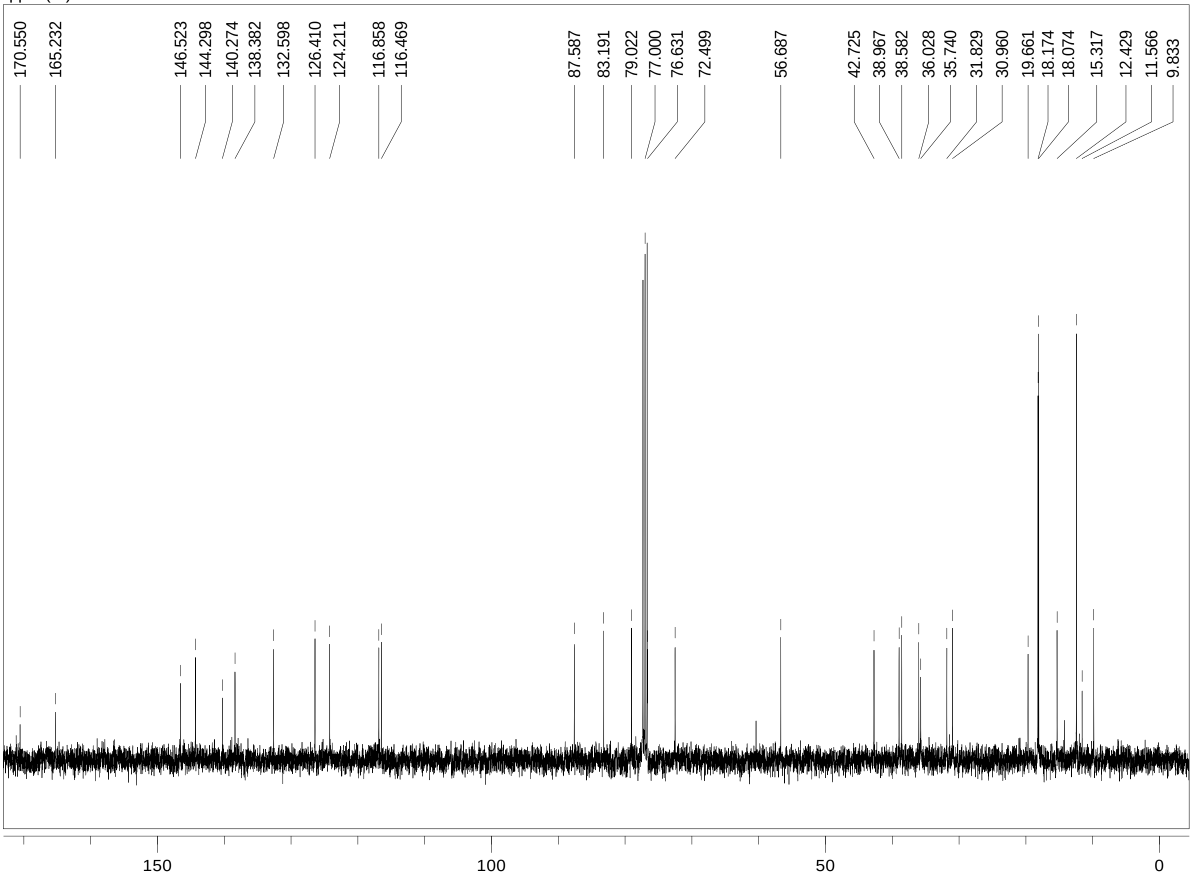
170.550
165.232
146.523
144.298
140.274
138.382
132.598
126.410
124.211
116.858
116.469
87.587
83.191
79.022
77.000
76.631
72.499
56.687
42.725
38.967
38.582
36.028
35.740
31.829
30.960
19.661
18.174
18.074
15.317
12.429
11.566
9.833
150
100
50
0
ppm (t1)

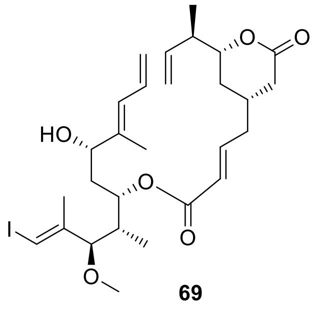

69

(1*E*,3*R*,4*S*,5*S*,7*S*,8*E*)-7-hydroxy-1-iodo-3-methoxy-2,4,8-trimethylundeca-1,8,10-trien-5-yl (*E*)-4-((2*R*,4*R*)-2-((*R*)-but-3-en-2-yl)-6-oxotetrahydro-2H-pyran-4-yl)but-2-enoate (70):

Using TBAF: To a solution of the TIPS-protected alcohol **5** (56.0 mg, 74.0 µmol, 1.00 eq) in THF (1 mL) were added a solution of TBAF (1.0 M in THF, 0.30 mL, 0.300 mmol, 4.00 eq) and acetic acid (17 µL, 0.296 mmol, 4.00 eq) at 0 °C. The cooling was removed after a few minutes and the mixture was stirred at rt overnight. As the conversion was incomplete, more TBAF (4 eq) and acetic acid (4 eq) were added at rt. After another 8 h at rt, the conversion was still incomplete. The reaction was quenched by addition of NH_4Cl (sat. aq.). The aqueous phase was diluted with water, ether (10 mL) was added and the layers were separated. The aqueous phase was extracted with ether (2×10 mL). The combined organic extracts were washed with brine, dried over $MgSO_4$ and concentrated under reduced pressure. The crude was purified by column chromatography (hex:EtOAc 5:1→1:1) to afford the desired alcohol **70** (18.3 mg, 87% wt/wt along with EtOAc, 15.9 mg, 47%) as a colourless oil. Furthermore some starting material **5** was recovered (15.2 mg, 27%).

Using buffered HF*Py: To a solution of the TIPS-protected alcohol **5** (52.4 mg, 69.2 µmol, 1.00 eq) in THF (2.6 mL) and pyridine (0.76 mL) was added a solution of HF*Py (1.53 mL 70% as HF, 1.18 g, 58.9 mmol, 850 eq) carefully at 0 °C. The cooling was removed after 10-15 min and the mixture was stirred at rt overnight. Afterwards the reaction was quenched into a mixture of $NaHCO_3$ (sat. aq., 40 mL) and Et_2O (40 mL). As soon as the gas evolution had ceased, the layers were separated and the organic phase was washed with $CuSO_4$ (sat. aq., 5 mL) and water (2×10 mL). The combined aqueous layers were extracted with Et_2O (10 mL). Afterwards the combined organic extracts were washed with brine, dried over $MgSO_4$ and concentrated under reduced pressure. The crude was purified by

column chromatography (hex:EtOAc 5:1→2:1), to afford the desired alcohol **70** (35.6 mg, 86%) as a colourless oil.

TLC (Hex:EtOAc 1:1): R_f = 0.46
^{1}H-NMR ($CDCl_3$, 400.1 MHz): δ = 6.89 (m_c, 1H), 6.55 (m_c, 1H), 6.19 (m_c, 1H), 6.05 (pseudo d, *J* = 10.9 Hz, 1H), 5.89 (td, *J* = 1.3 Hz, *J* = 15.5 Hz, 1H), 5.74 (ddd, *J* = 7.7 Hz, *J* = 10.4 Hz, *J* = 17.3 Hz, 1H), 5.19 (dd, *J* = 1.8 Hz, *J* = 16.8 Hz, 1H), 5.24-5.02 (m, 5H), 4.18-4.07 (m, 1H), 3.88 (m_c, 1H), 3.49 (d, *J* = 5.7 Hz, 1H), 3.16 (s, 3H), 2.76-2.64 (m, 1H), 2.54 (d, *J* = 3.6 Hz, 1H), 2.45 (m_c, 1H), 2.30-2.21 (m, 2H), 2.19-2.05 (m, 2H), 2.02-1.92 (m, 2H), 1.77 (d, *J* = 1.0 Hz, 3H), 1.75 (d, *J* = 0.9 Hz, 3H), 1.73-1.60 (m, 2H), 1.28-1.16 (m, 1H), 1.12 (d, *J* = 6.8 Hz, 3H), 0.90 (d, *J* = 6.9 Hz, 3H).
HRMS Calcd. for $C_{28}H_{41}INaO_6$ $[M+Na]^+$ *m/z* 623.1840. Found: 623.1839.

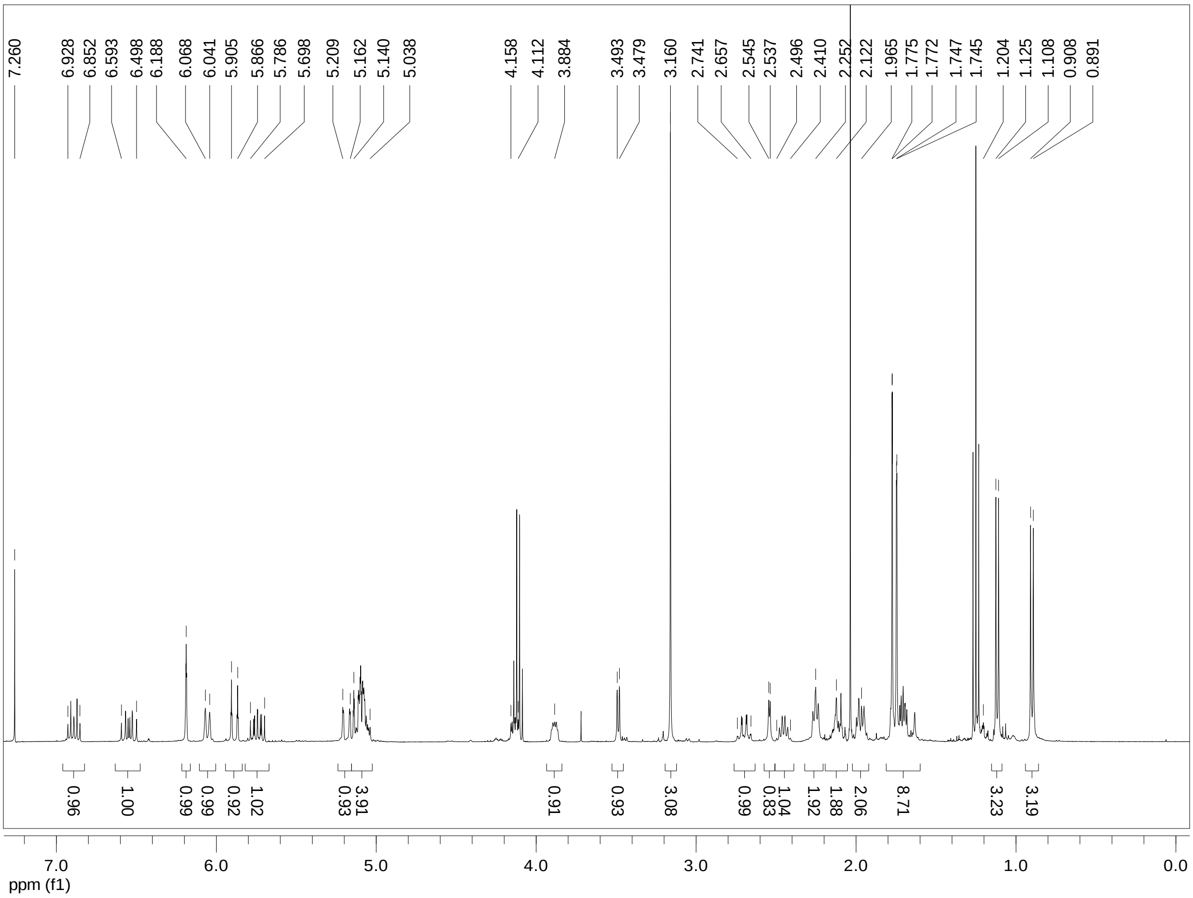
7.260
6.928
6.852
6.593
6.498
6.188
6.068
6.041
5.905
5.866
5.786
5.698
5.209
5.162
5.140
5.038
4.158
4.112
3.884
3.493
3.479
3.160
2.741
2.657
2.545
2.537
2.496
2.410
2.252
2.122
1.965
1.775
1.772
1.747
1.745
1.204
1.125
1.108
0.908
0.891
0.96
1.00
0.99
0.99
0.92
1.02
0.93
3.91
0.91
0.93
3.08
0.99
0.83
1.04
1.92
1.88
2.06
8.71
3.23
3.19
7.0
6.0
5.0
4.0
3.0
2.0
1.0
0.0
ppm (f1)

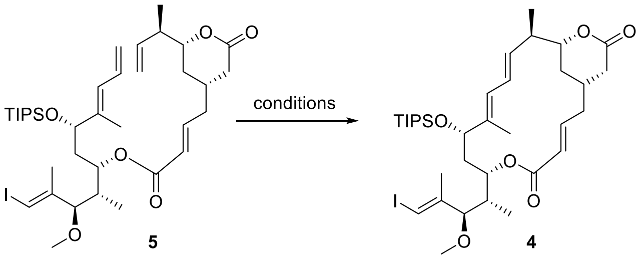

5 4

Representative procedure for initial single-batch test reactions:

To a solution of the catalyst (20 mol%) in CH_2Cl_2 (4.5 mL) was added a solution of the ene-diene **5** (6.7 mg, 8.9 µmol, 1.00 eq) in CH_2Cl_2 (0.5 mL, rinsed with 2×0.5 mL). After a few minutes stirring at rt, the pale brown solution was heated to 50 °C overnight. The conversion was assessed by ESI-MS and TLC.

Remark: The reaction was run in a microwave vial and the catalyst was weighed directly into the tube in a glovebox.

Representative procedure for catalyst-/solvent-/temperature screenings:

A stock solution of the ene-diene **5** in CH_2Cl_2 was prepared such that 0.1 mL of the stock solution contained 2.0 mg of the substrate.

0.1 mL of the substrate stock solution was diluted with 1.7 mL of the solvent to be assessed. Then the respective catalyst stock solution in CH_2Cl_2 (typically around 0.2 mL, corresponding to 20 mol%) was added. The conversion was assessed by ESI-MS from time-to-time. In most of the cases, the reaction mixtures were stirred overnight at the desired temperature, whereafter the conversion was re-assessed by ESI-MS. The samples that had been kept at rt were later heated.

Table S1: RCM screening for substrate **5**

entry	catalyst (mol%)	solvent	Temp./°C	conversion[a]
1	Hoveyda-Grubbs 1 (10)	tol	90	no
2	Hoveyda-Grubbs 1 (20)	CH_2Cl_2	50	no
3	Hoveyda-Grubbs 2 (20)	CH_2Cl_2	50	no
4	Hoveyda-Grubbs 2 (20)	tol	95	yes
5	Grubbs 1 (20)	CH_2Cl_2	50	no
6	Grubbs 1 (20)	tol	95	yes
7	Grubbs 2 (10)	CH_2Cl_2	50	yes, incomplete
8	Grubbs 2 (20)	CH_2Cl_2	50	yes, full
9	Grubbs 2 (10)	tol	rt	no
10	Grubbs 2 (10)	tol	45	no
11	Grubbs 2 (20)	tol	95	yes
12	Grubbs 3 (20)	tol	rt	no
13	Grubbs 3 (20)	CH_2Cl_2	rt	no
14	Piers-Grubbs 2 (20)	tol	rt	no
15	Piers-Grubbs 2 (20)	CH_2Cl_2	rt	no
16	UHGS* (20)	CH_2Cl_2	40	no
17	UHGS* (20)	tol	95	no

[a] consumption of starting material. Yes = complete consumption; no = no consumption. For incomplete conversion, the degree of the consumption of starting material was not quantified.

*

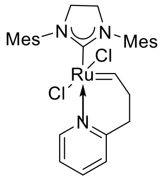

Ung, T.; Hejl, A.; Grubbs, R. H.; Schrodi, Y. Latent Ruthenium Olefin Metathesis Catalysts That Contain an N-Heterocyclic Carbene Ligand. *Organometallics* **2004**, *23*, 5399-5401.

Table S2: RCM screening for substrate **70**

entry	catalyst (20 mol%)	solvent	temp./°C	conversion[a]
1	Grubbs 1	tol	95	no
2	Grubbs 2	tol	95	full
3	Hoveyda-Grubbs 2	tol	95	full
4	Grubbs 1	CH_2Cl_2	50	no
5	Grubbs 2	CH_2Cl_2	50	full
6	Hoveyda-Grubbs 2	CH_2Cl_2	50	incomplete

[a] consumption of starting material. no = no consumption. For incomplete conversion, the degree of the consumption of starting material was not quantified.

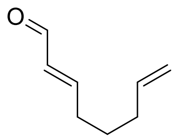

S15

(*E*)-Octa-2,7-dienal (S15): To a slurry of celite (12 g) in CH_2Cl_2 (55 mL) was added PCC (3.84 g, 17.8 mmol, 1.50 eq). To this was then added a solution of 2,7-octadienol (**71**) (1.50 g, 11.9 mmol, 1.00 eq) in CH_2Cl_2 (5 mL) dropwise at rt, whereupon the mixture turned brown and thicker. After 2.5 h stirring at rt, the slurry was diluted with ether (100 mL) and filtered through a paper filter. The residue was washed thoroughly with ether and the combined filtrates were concentrated under reduced pressure (40 °C, 800−690 mbar) to leave a brown liquid. This was passed through a pad of silica washing with ether. The combined filtrates were again concentrated to yield aldehyde **S15** as a slightly green liquid (1.44 g, 84% wt/wt along with Et_2O and CH_2Cl_2, 1.22 g, 82%), which was volatile.

TLC (Hex:EtOAc 5:1): R_f = 0.51
^{1}H-NMR ($CDCl_3$, 400.1 MHz): δ = 9.51 (d, *J* = 7.9 Hz, 1H), 6.85 (td, *J* = 6.8 Hz, *J* = 15.6 Hz, 1H), 6.13 (tdd, *J* = 1.5 Hz, *J* = 7.9 Hz, *J* = 15.6 Hz, 1H), 5.79 (tdd, *J* = 6.7 Hz, *J* = 10.2 Hz, *J* = 16.9 Hz, 1H), 5.07-4.97 (m, 2H), 2.35 (m_c, 2H), 2.11 (m_c, 2H), 1.62 (m_c, 2H).

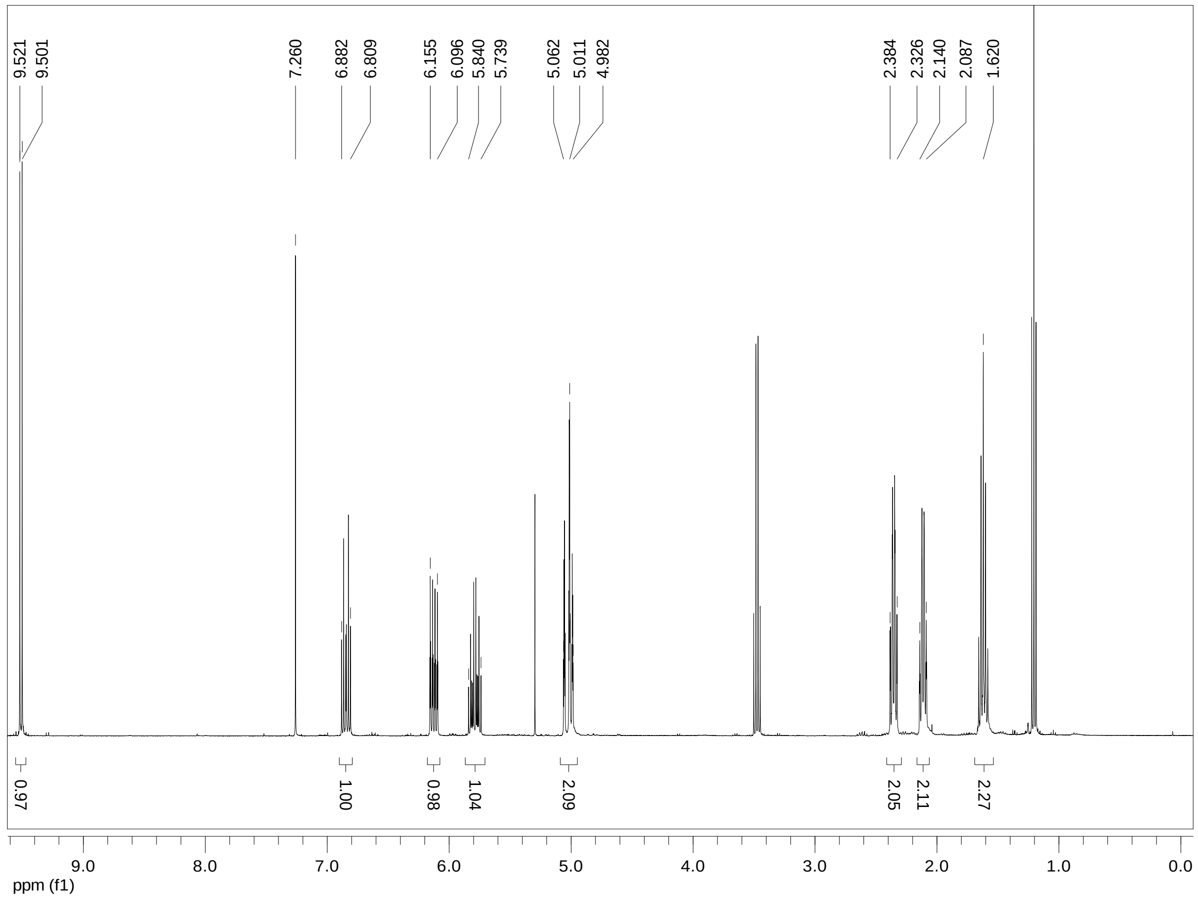
9.521
9.501
7.260
6.882
6.809
6.155
6.096
5.840
5.739
5.062
5.011
4.982
2.384
2.326
2.140
2.087
1.620
0.97
1.00
0.98
1.04
2.09
2.05
2.11
2.27
9.0
8.0
7.0
6.0
5.0
4.0
3.0
2.0
1.0
0.0
ppm (f1)

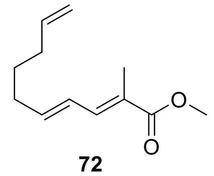

72

Methyl (2*E*,4*E*)-2-methyldeca-2,4,9-trienoate (72): To a solution of the phosphonium ylide **17** (3.41 g, 9.79 mmol, 1.00 eq) in CH_2Cl_2 (15 mL) was added aldehyde **S15** (1.22 g, 9.82 mmol, 1.00 eq) dropwise at rt. Unlike in the corresponding reaction with acrolein (*vide supra*), the addition proceeded only slightly exothermic. The yellow solution was then heated to a gentle reflux (47 °C oil bath). TLC after 3 h 20 min showed, that the conversion was incomplete. Therefore, more ylid (682 mg, 0.2 eq) was added and the mixture was heated to reflux overnight. Despite some starting material still being left, the mixture was allowed to cool to rt and concentrated under reduced pressure. The crude was cooled to 0 °C and pentane was added. The precipitated phosphinoxide was filtered off and washed thoroughly with pentane. The combined filtrates were concentrated under reduced pressure and the remaining yellow liquid was purified by column chromatography (hex:EtOAc 10:1) to afford the desired diene **72** as a colourless liquid (1.14 g, 60%, *trans* only; 636 mg, *trans*/*cis* = 4/1, 33%).

TLC (hex:EtOAc 5:1): $R_{f,cis}$ = 0.73; $R_{f,trans}$ = 0.63
^{1}H-NMR ($CDCl_3$, 400.1 MHz): δ = 7.16 (bd, *J* = 11.3 Hz, 1H, C*H*=CMe), 6.34 (tdd, *J* = 1.4 Hz, *J* = 11.3 Hz, *J* = 14.9 Hz, 1H, C*H*=CHCH$_2$), 6.07 (td, *J* = 7.1 Hz, *J* = 15.0 Hz, 1H, CH=C*H*CH$_2$), 5.80 (tdd, *J* = 6.7 Hz, *J* = 10.2 Hz, *J* = 16.9 Hz, 1H, CH$_2$=C*H*), 5.07-4.93 (m, 2H, C*H*$_2$=CH), 3.75 (s, 3H, OC*H*$_3$), 2.21 (td, *J* = 7.2 Hz, *J* = 7.2 Hz, 2H, C*H*$_2$CH=CH), 2.15-2.03 (m, 2H, C*H*$_2$CH=CH$_2$), 1.93 (d, *J* = 0.9 Hz, 3H, CH=CC*H*$_3$), 1.62-1.47 (m, 2H, CH$_2$C*H*$_2$CH$_2$),
^{13}C-NMR ($CDCl_3$, 100.6 MHz): δ = 169.1, 142.8, 138.8, 138.3, 126.2, 124.9, 114.9, 51.7, 33.2, 32.6, 28.1, 12.6.
IR (ν/[cm^{-1}]) 3078, 3034, 2994, 2980, 2948, 2928, 2857, 1707 (s), 1640, 1610, 1435, 1389, 1292, 1266, 1235 (s), 1191, 1104, 970, 940, 911, 831, 748, 689, 634, 552, 489, 457, 441, 424.
HRMS Calcd. for $C_{12}H_{19}O_2$ $[M+H]^+$ *m/z* 195.1380. Found: 195.1378.

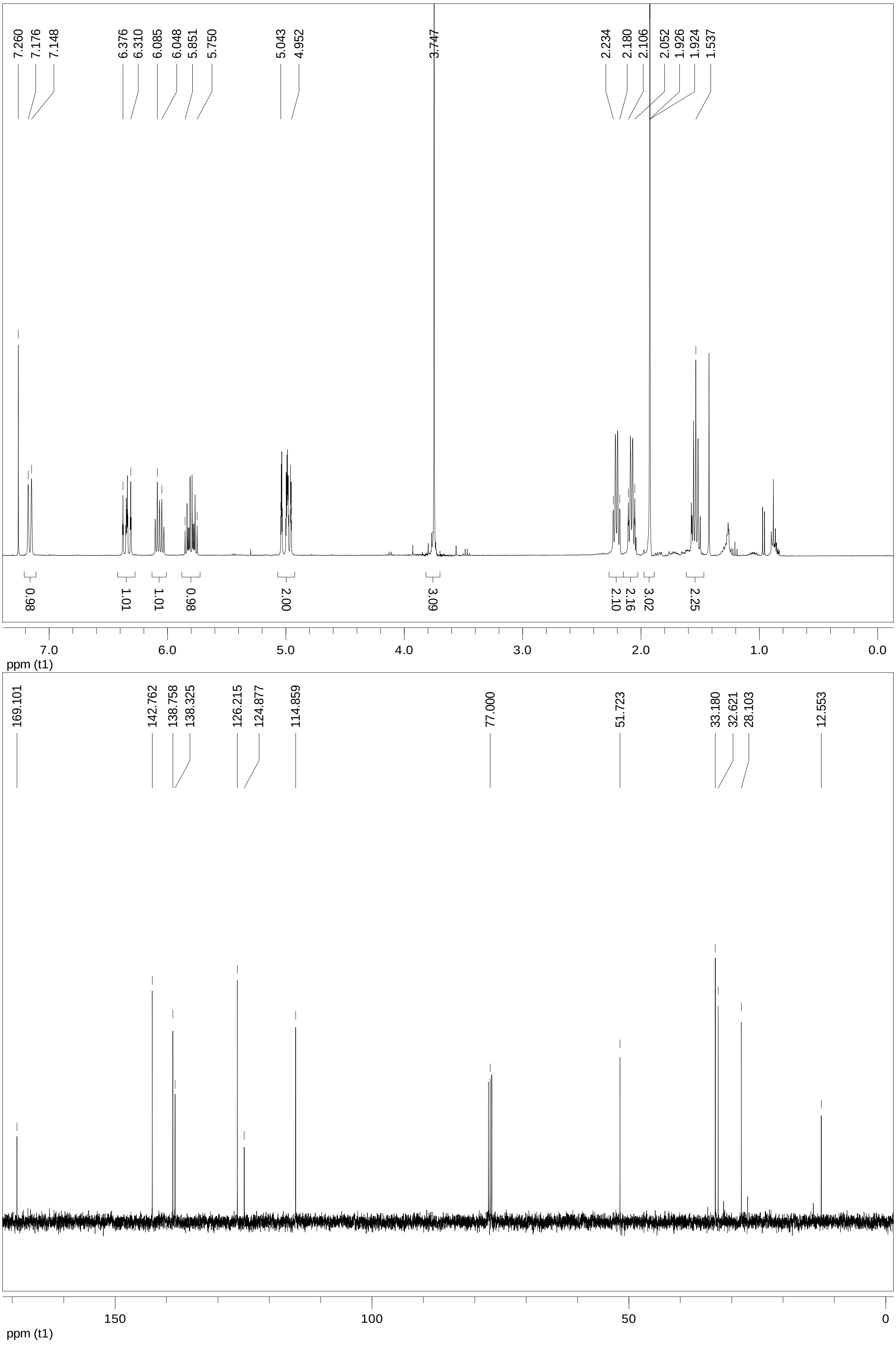
7.260
7.176
7.148
6.376
6.310
6.085
6.048
5.851
5.750
5.043
4.952
3.747
2.234
2.180
2.106
2.052
1.926
1.924
1.537
0.98
1.01
1.01
0.98
2.00
3.09
2.10
2.16
3.02
2.25
7.0 6.0 5.0 4.0 3.0 2.0 1.0 0.0
ppm (t1)
169.101
142.762
138.758
138.325
126.215
124.877
114.859
77.000
51.723
33.180
32.621
28.103
12.553
150 100 50 0
ppm (t1)

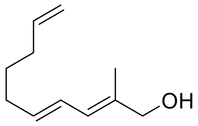

S16

(2*E*,4*E*)-2-Methyldeca-2,4,9-trien-1-ol (S16): To a cooled (0 °C) solution of the ester **72** (1.12 g, 5.75 mmol, 1.00 eq) in Et_2O (30 mL) was added $LiAlH_4$ (218 mg, 5.75 mmol, 1.00 eq) in one portion. The mixture was stirred at 0 °C for 50 min. Afterwards the reaction was quenched with water (10 mL) followed by 1 M NaOH (10 mL) and more water (30 mL). The layers were separated, additional 1 M NaOH was added and the aqueous phase was extracted with Et_2O (3×20 mL). The combined organic extracts were washed with brine, dried over $MgSO_4$ and concentrated under reduced pressure to afford the desired alcohol **S16** (895 mg, 94%) as a colourless liquid.

TLC (hex:EtOAc 5:1): R_f = 0.24.
^{1}H-NMR ($CDCl_3$, 400.1 MHz): δ = 6.26 (tdd, *J* = 1.4 Hz, *J* = 10.8 Hz, *J* = 15.0 Hz, 1H), 6.02 (bd, *J* = 10.8 Hz, 1H), 5.81 (tdd, *J* = 6.7 Hz, *J* = 10.2 Hz, *J* = 16.9 Hz, 1H), 5.69 (m_c, 1H), 5.06-4.91 (m, 2H), 4.06 (d, *J* = 5.8 Hz, 2H), 2.18-2.02 (m, 4H), 1.78 (bs, 3H), 1.50 (m_c, 2H), 1.38-1.30 (m, 1H).
^{13}C-NMR ($CDCl_3$, 100.6 MHz): δ = 138.7, 134.8, 134.7, 126.2, 125.3, 114.6, 68.7, 33.2, 32.3, 28.6, 14.1; **IR** (ν/[cm^{-1}]) 3323 (br), 3077, 3026, 2978, 2924, 2856, 1640, 1453, 1438, 1415, 1388, 1225, 1143, 1066, 996 (s), 966 (s), 909 (s), 883, 668, 635, 610, 456, 448.

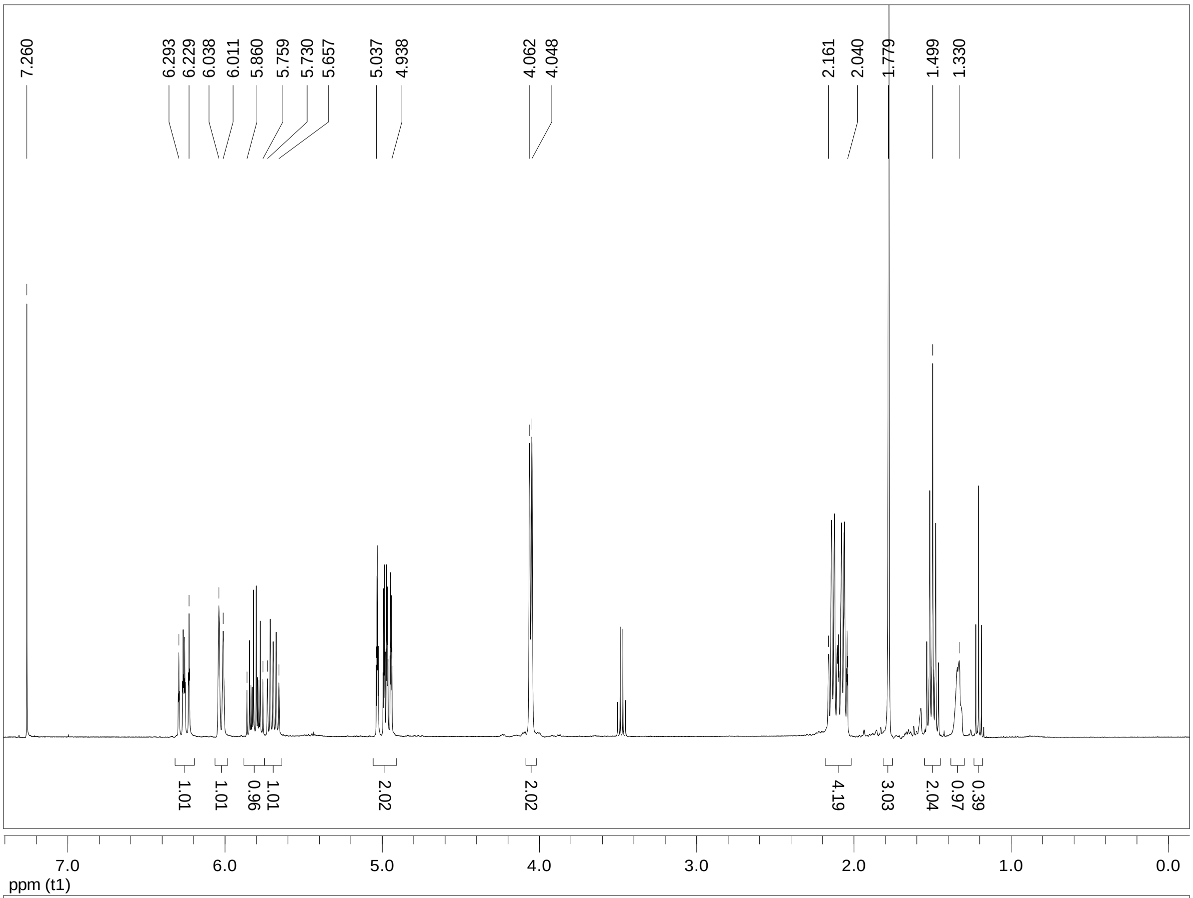
7.260
6.293
6.229
6.038
6.011
5.860
5.759
5.730
5.657
5.037
4.938
4.062
4.048
2.161
2.040
1.779
1.499
1.330
1.01
1.01
0.96
1.01
2.02
2.02
4.19
3.03
2.04
0.97
0.39
7.0
6.0
5.0
4.0
3.0
2.0
1.0
0.0
ppm (t1)

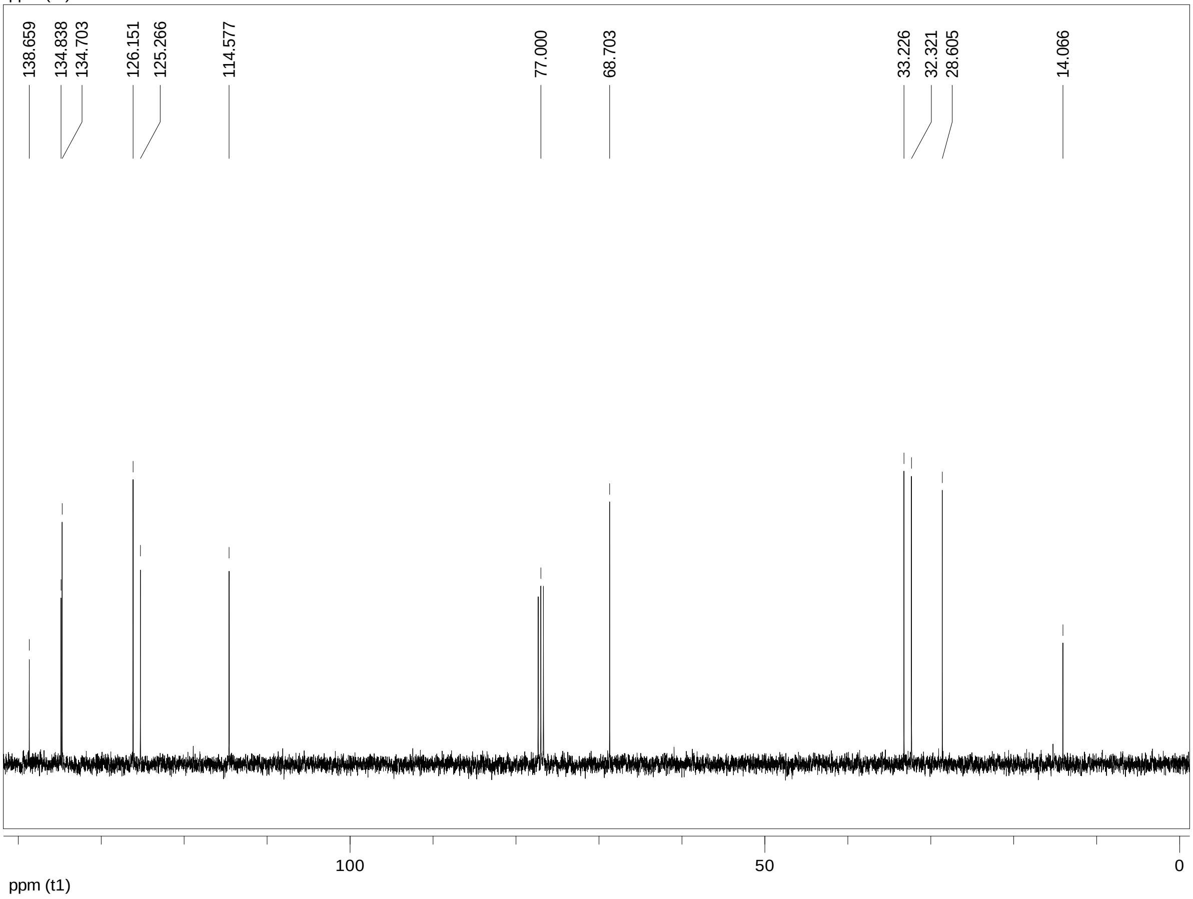
138.659
134.838
134.703
126.151
125.266
114.577
77.000
68.703
33.226
32.321
28.605
14.066
100
50
0
ppm (t1)

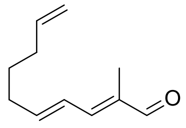

73

(2*E*,4*E*)-2-Methyldeca-2,4,9-trienal (73): To a solution of the allylic alcohol **S16** (150 mg, 0.902 mmol, 1.00 eq) in CH_2Cl_2 (3 mL) were added molecular sieves (3 Å, beads) and activated MnO_2 (1.57 g, 18.0 mmol, 20.0 eq) at rt. The suspension was stirred for 2 h at the same temperature. Afterwards it was filtered through a pad of celite, topped with a few mm of silica. The filter was washed thoroughly with Et_2O. The combined filtrates were concentrated under reduced pressure and the crude aldehyde **73** (161 mg, 86% wt/wt along with Et_2O and CH_2Cl_2, 139 mg, 85%), a pale yellow liquid, was used directly in the next step.

TLC (hex:EtOAc 5:1): R_f = 0.48
^{1}H-NMR ($CDCl_3$, 400.1 MHz): δ = 9.42 (s, 1H), 6.82 (bd, *J* = 11.1 Hz, 1H), 6.53 (tdd, *J* = 1.4 Hz, *J* = 11.1 Hz, *J* = 15.0 Hz, 1H), 6.23 (td, *J* = 7.1 Hz, *J* = 15.0 Hz, 1H), 5.81 (tdd, *J* = 6.7 Hz, *J* = 10.2 Hz, *J* = 16.9 Hz, 1H), 5.07-4.96 (m, 2H), 2.27 (td, *J* = 7.4 Hz, *J* = 7.4 Hz, 2H), 2.10 (m_c, 2H), 1.83 (d, *J* = 0.8 Hz, 3H), 1.58 (m_c, 2H).

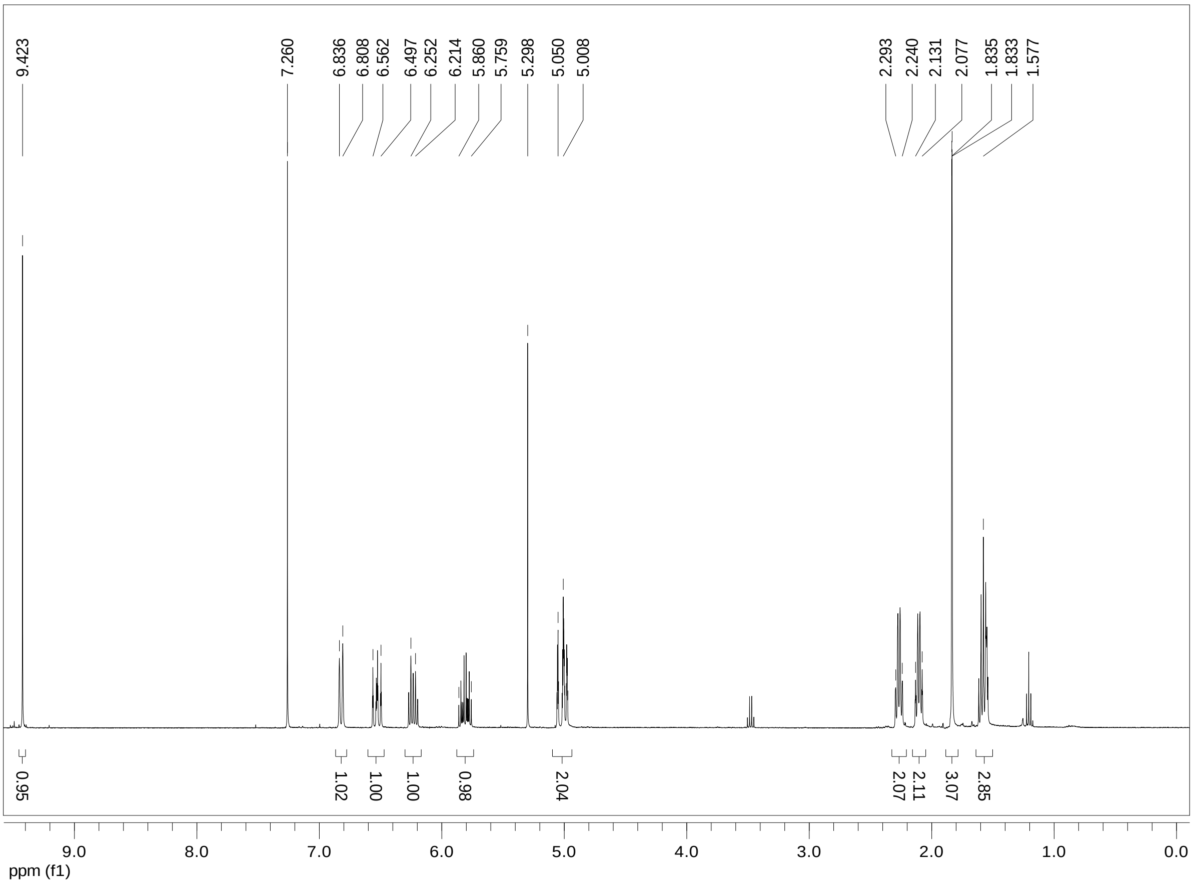
9.423
7.260
6.836
6.808
6.562
6.497
6.252
6.214
5.860
5.759
5.298
5.050
5.008
2.293
2.240
2.131
2.077
1.835
1.833
1.577
0.95
1.02
1.00
1.00
0.98
2.04
2.07
2.11
3.07
2.85
9.0
8.0
7.0
6.0
5.0
4.0
3.0
2.0
1.0
0.0
ppm (f1)

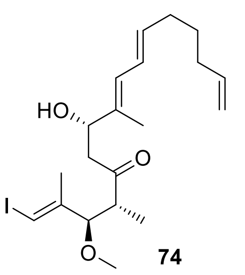

74

(1*E*,3*R*,4*R*,7*S*,8*E*,10*E*)-7-Hydroxy-1-iodo-3-methoxy-2,4,8-trimethylhexadeca-1,8,10,15-tetraen-5-one (74): To a cooled (−78 °C) solution of (+)-DIP-Cl (227 mg, 0.709 mmol, 2.00 eq) in CH_2Cl_2 (1 mL) was added NEt_3 (0.118 mL, 0.851 mmol, 2.40 eq) followed by a solution of the ketone **27** (523 mg, 1.85 mmol, 1.00 eq) in CH_2Cl_2 (0.4 mL, rinsed with 2×0.3 mL). Ca. 5 min following the addition of the ketone, a white precipitate formed. The thick suspension was stirred for 3 h 15 min at −78 °C. Afterwards a solution of the aldehyde **73** (132 mg, 0.804 mmol, 2.27 eq) in CH_2Cl_2 (1 mL) was added slowly, whereupon the colour immediately turned bright yellow. The mixture was stirred overnight, allowing for the cooling bath to warm to − 20 °C. The reaction was then quenched with pH 7 phosphate buffer and the layers were separated. The aqueous phase was diluted with water and extracted with CH_2Cl_2 (3×30 mL). The combined extracts were concentrated under reduced pressure. The residue was dissolved in MeOH (2 mL) and pH 7 phosphate buffer (0.4 mL) was added. The mixture was cooled to 0 °C and H_2O_2 (30%, 0.5 mL) was added. The cooling was removed after a few minutes and the mixture was stirred for 1 h at rt. Afterwards it was poured into water (30 mL). CH_2Cl_2 was added and the layers were separated. The aqueous phase was extracted with CH_2Cl_2 (3×10 mL). The combined organic layers were washed with brine, dried over $MgSO_4$ and concentrated under reduced pressure. The crude was purified by column chromatography (hex:EtOAc 10:1→5:1) to afford the desired product **74** (245 mg, 44% wt/wt along with isopinocampheol, 107 mg, 68%, dr=7.2:1) as a nearly colourless oil.

^{1}H-NMR ($CDCl_3$, 400.1 MHz): δ = 6.28-6.17 (m, 2H), 6.07 (m_c, 1H), 5.80 (tdd, *J* = 6.7 Hz, *J* = 10.2 Hz, *J* = 16.9 Hz, 1H), 5.70 (td, *J* = 7.1 Hz, *J* = 14.9 Hz, 1H), 5.00 (ddd, *J* = 1.6 Hz, *J* = 3.6 Hz, *J* = 17.1 Hz, 1H), 4.95 (tdd, *J* = 1.2 Hz, *J* = 2.2 Hz, *J* = 10.2 Hz, 1H), 4.44 (m_c, 1H), 3.84 (d, *J* = 7.0 Hz, 1H), 3.21 (s, 3H), 2.83 (d, *J* = 2.9 Hz, 1H), 2.76 (p, *J* = 7.0 Hz, 1H), 2.72-2.60 (m, 1H), 2.57-2.50 (m, 1H), 2.16-2.02 (m, 4H), 1.75 (d, *J* = 1.1 Hz, 3H), 1.73 (d, *J* = 0.9 Hz, 3H), 1.49 (m_c, 2H), 1.12 (d, *J* = 6.9 Hz, 3H).

^{13}C-NMR ($CDCl_3$, 100.6 MHz): δ = 212.7, 145.3, 138.6, 135.3, 135.2, 126.0, 125.5, 114.6, 86.3, 80.6, 72.3, 57.0, 50.1, 47.7, 33.2, 32.3, 28.6, 19.9, 12.8, 12.2.

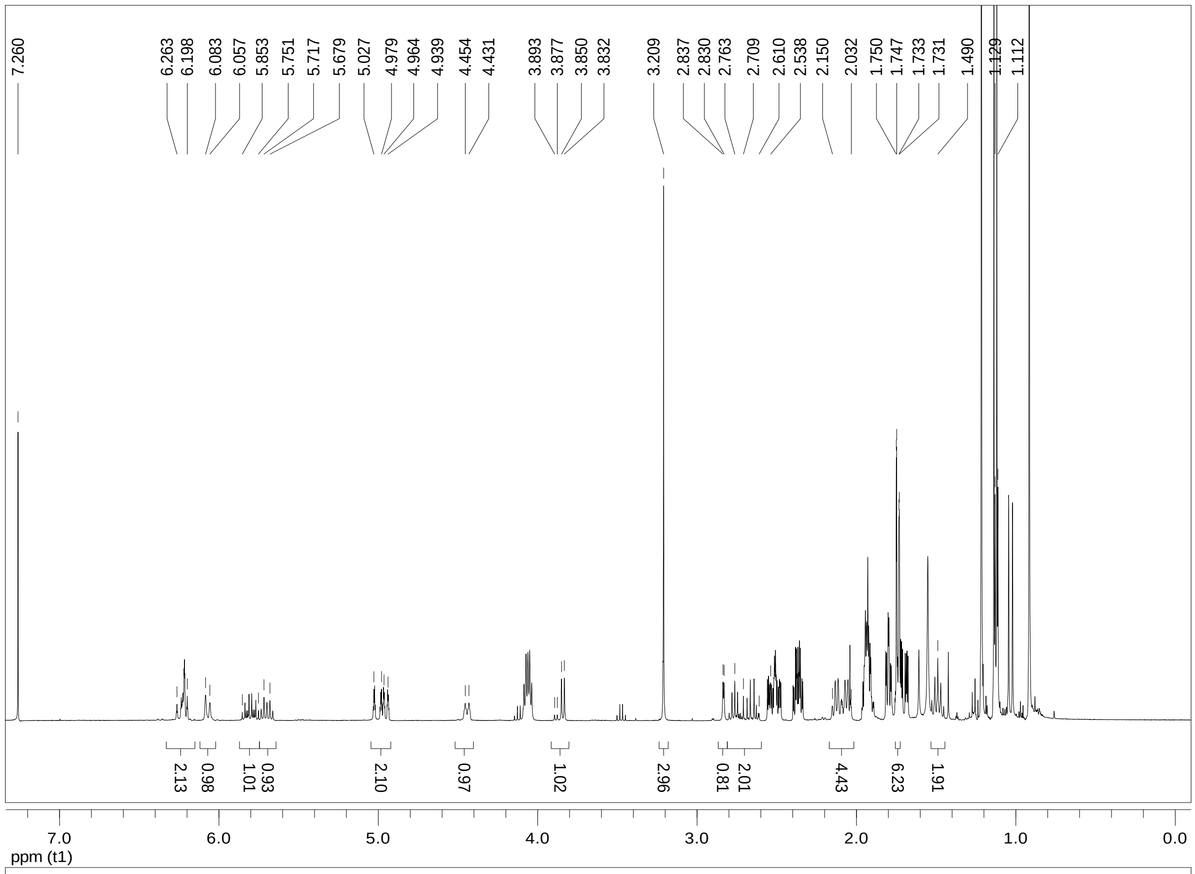
7.260
6.263
6.198
6.083
6.057
5.853
5.751
5.717
5.679
5.027
4.979
4.964
4.939
4.454
4.431
3.893
3.877
3.850
3.832
3.209
2.837
2.830
2.763
2.709
2.610
2.538
2.150
2.032
1.750
1.747
1.733
1.731
1.490
1.129
1.112
2.13
0.98
1.01
0.93
2.10
0.97
1.02
2.96
0.81
2.01
4.43
6.23
1.91
7.0
6.0
5.0
4.0
3.0
2.0
1.0
0.0
ppm (t1)

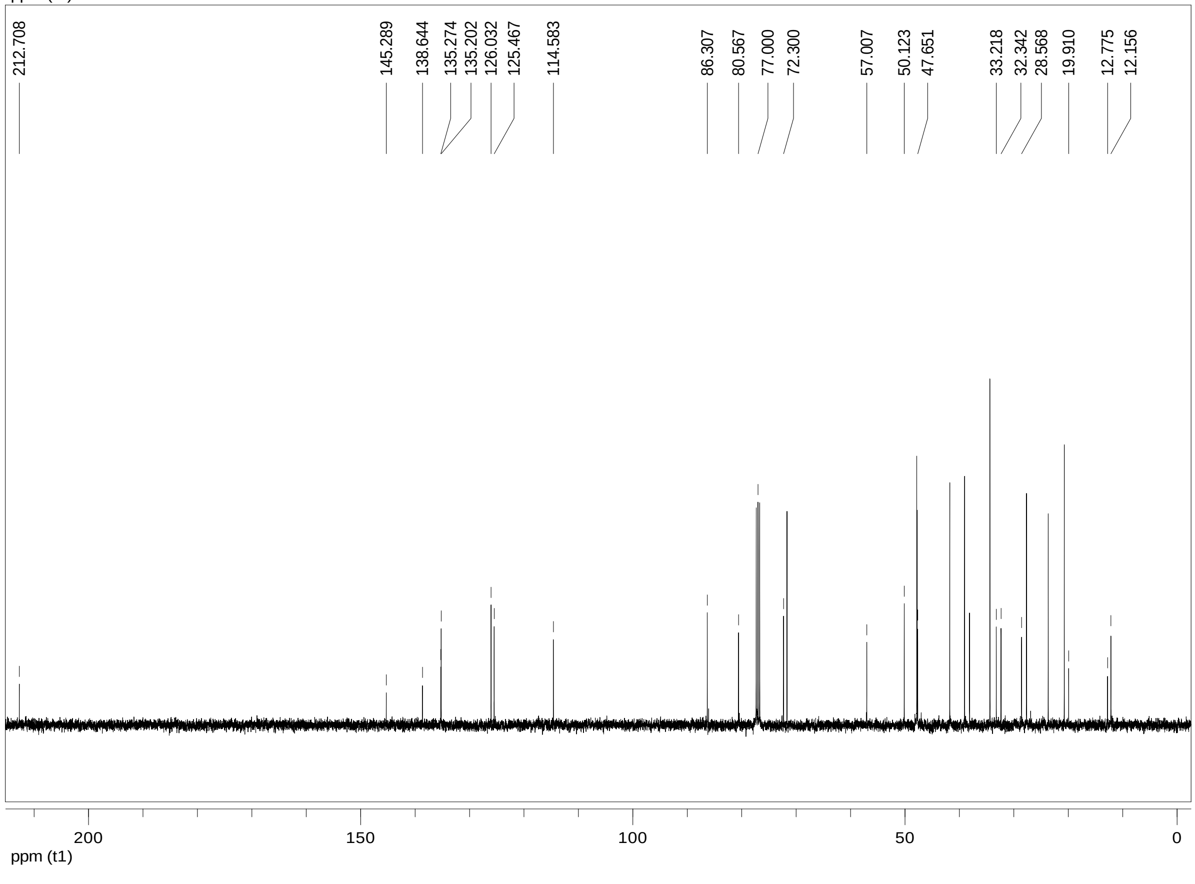
212.708
145.289
138.644
135.274
135.202
126.032
125.467
114.583
86.307
80.567
77.000
72.300
57.007
50.123
47.651
33.218
32.342
28.568
19.910
12.775
12.156
200
150
100
50
0
ppm (t1)

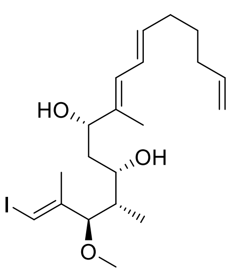

S17

(1*E*,3*R*,4*S*,5*S*,7*S*,8*E*,10*E*)-1-Iodo-3-methoxy-2,4,8-trimethylhexadeca-1,8,10,15-tetraene-5,7-diol (S17): A solution of tetramethylammonium triacetoxyborohydride (283 mg, 1.08 mmol, 4.65 eq) in MeCN (2.5 mL) and AcOH (2.5 mL) was cooled to −40 °C (MeCN/dry ice). To the frozen mixture was added a solution of the ketone **74** (103 mg, 0.231 mmol, 1.00 eq) in MeCN (0.6 mL, rinsed with 2×0.6 mL). The resulting mixture was allowed to stand a few min. at the same temperature and was then aged in a freezer (−18 °C) for 19 h. Afterwards the suspension was allowed to warm to rt and stirred for another 1.5 h. Then the reaction was quenched with *Rochelle* salt (sat. aq.). The so obtained thick slurry was stirred for 2 h 15 min. Afterwards it was transferred with water (ca. 80 mL) into an *Erlenmeyer* flask, then CH_2Cl_2 (20 mL) and $NaHCO_3$ (s, 3.7 g) were added. Once the gas evolution had stopped, the layers were separated and the aqueous phase was extracted with CH_2Cl_2 (3×10 mL). The combined organic extracts were washed with brine, dried over $MgSO_4$ and concentrated under reduced pressure. The crude was purified by column chromatography (hex:Et_2O 2:1→1:1) to afford the desired product **S17** (94.0 mg, 94% wt/wt along with isopinocampheol, 88.6 mg, 86%, dr = 7.8:1 from previous aldol). Despite the fact that still some isopinocampheol was left, the material was taken forward to the next step.

TLC (hex:EtOAc 2:1): R_f = 0.38

^{1}H-NMR ($CDCl_3$, 400.1 MHz): δ = 6.32-6.20 (m, 1H), 6.19-6.07 (m, 2H), 5.81 (tdd, *J* = 6.7 Hz, *J* = 10.2 Hz, *J* = 16.9 Hz, 1H), 5.70 (m_c, 1H), 5.01 (ddd, *J* = 1.6 Hz, *J* = 3.7 Hz, *J* = 17.1 Hz, 1H), 4.96 (tdd, *J* = 1.2 Hz, *J* = 2.2 Hz, *J* = 10.2 Hz, 1H), 4.37 (td, *J* = 3.7 Hz, *J* = 7.4 Hz, 1H), 3.96 (bd, , *J* = 3.2 Hz, 1H), 3.86-3.77 (m, 1H), 3.27 (s, 3H), 3.23 (d, *J* = 5.9 Hz, 1H), 2.54 (d, *J* = 4.0 Hz, 1H), 2.17-2.03 (m, 4H), 1.82-1.67 (m, 3H), 1.76 (d, *J* = 1.0 Hz, 3H), 1.73 (bs, 3H), 1.50 (td, *J* = 7.4 Hz, *J* = 14.9 Hz, 2H), 0.84 (d, *J* = 7.1 Hz, 3H).

^{13}C-NMR ($CDCl_3$, 100.6 MHz): δ = 145.3, 138.7, 137.2, 134.8, 126.1, 124.6, 114.6, 86.5, 78.1, 74.3, 71.3, 57.3, 40.7, 38.6, 33.3, 32.4, 28.6, 21.8, 13.1, 10.9.

HRMS Calcd. for $C_{20}H_{33}INaO_3$ [M+Na]$^+$ *m/z* 471.1367. Found: 471.1368.

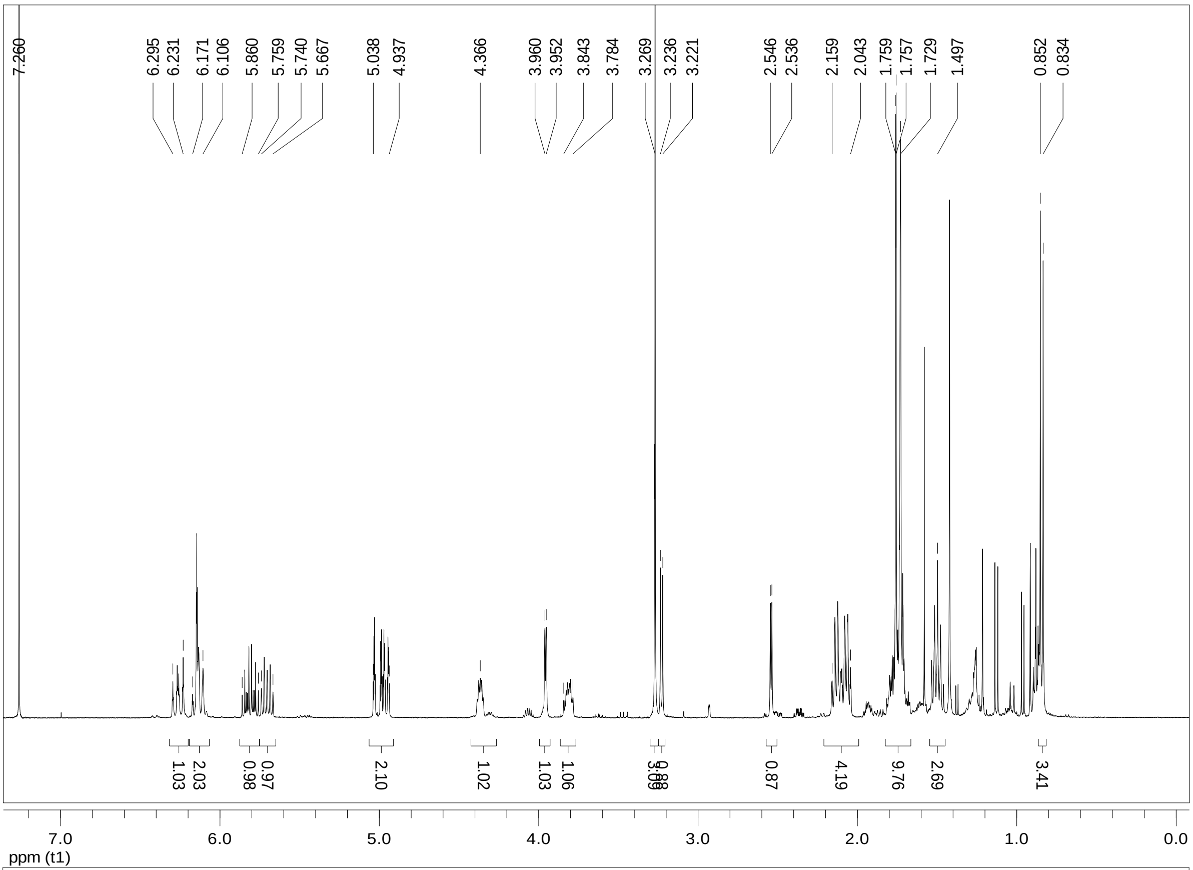

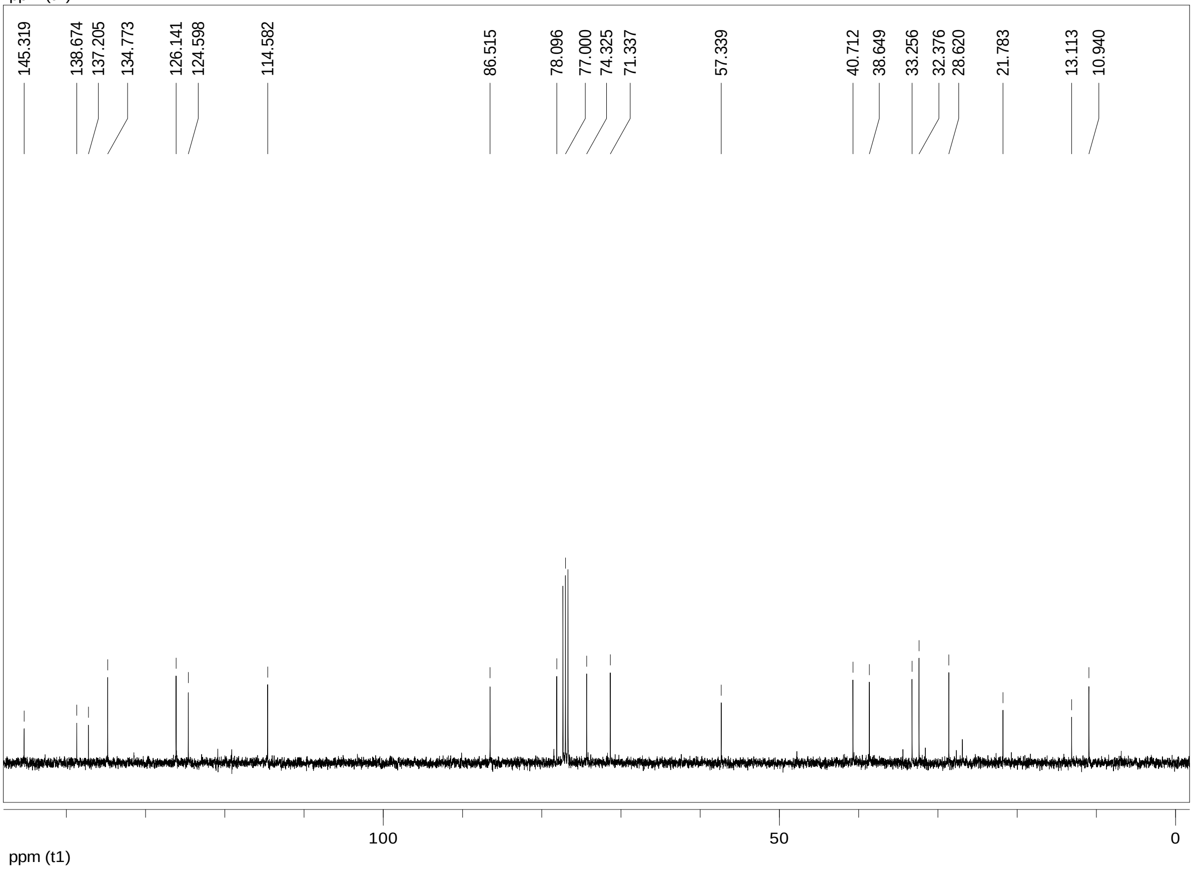

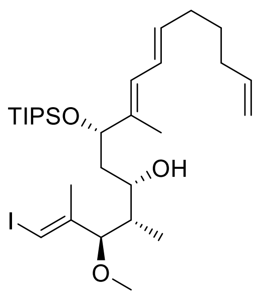

S18

(1*E*,3*R*,4*S*,5*S*,7*S*,8*E*,10*E*)-1-Iodo-3-methoxy-2,4,8-trimethyl-7-((triisopropylsilyl)-oxy)hexadeca-1,8,10,15-tetraen-5-ol (S18): To a solution of the secondary alcohol **S17** (88.6 mg, 0.198 mmol, 1.00 eq) in CH_2Cl_2 (5.5 mL) over molecular sieves (3 Å beads) was added 2,6-lutidine (115 µL, 0.988 mmol, 5.00 eq) at rt. The mixture was stirred at rt for 5 min, then it was cooled to −78 °C and, after another 5 min stirring, TIPSOTf (61.0 µL, 0.227 mmol, 1.15 eq) was added. After 20 min the reaction was quenched with $NaHCO_3$ (sat. aq.). The layers were separated and the aqueous phase was extracted with CH_2Cl_2 (10 mL). Then the combined organic extracts were washed with brine, dried over $MgSO_4$ and the solvent was removed under reduced pressure. The crude was purified by column chromatography (hex:Et_2O 7:1) to afford the desired product **S18** as a colourless oil (102 mg, 85%). ^{1}H-NMR analysis revealed, that three isomers were present, but it was impossible to distinguish the TIPS-regioisomer and the diastereoisomers. In total, the ratio of desired vs. undesired was around 6/1 (see signal at 4.47 ppm).

TLC (hex:EtOAc 5:1): R_f = 0.57

^{1}H-NMR ($CDCl_3$, 400.1 MHz): δ = 6.32-6.18 (m, 1H), 6.18-6.01 (m, 2H), 5.82 (tdd, *J* = 6.7 Hz, *J* = 10.2 Hz, *J* = 16.9 Hz, 1H), 5.65 (m_c, 1H), 5.01 (ddd, *J* = 1.6 Hz, *J* = 3.7 Hz, *J* = 17.1 Hz, 1H), 4.95 (tdd, *J* = 1.2 Hz, *J* = 2.2 Hz, *J* = 10.2 Hz, 1H), 4.7 (m_c, 1H), 3.75-3.65 (m, 2H), 3.60 (d, *J* = 2.3 Hz, 1H), 3.21 (s, 3H), 2.18-2.02 (m, 4H), 1.78-1.63 (m, 3H), 1.73 (d, *J* = 1.0 Hz, 3H), 1.69 (bs, 3H), 1.51 (m_c, 2H), 1.11-1.02 (m, 21H), 0.84 (d, *J* = 7.0 Hz, 3H).

^{13}C-NMR ($CDCl_3$, 100.6 MHz): δ = 146.3, 138.8, 135.7, 134.2, 126.2, 125.3, 114.5, 86.5, 78.0, 76.5, 69.7, 57.2, 41.2, 38.1, 33.3, 32.4, 28.6, 21.0, 18.05, 18.01, 13.8, 12.3, 10.1.

HRMS Calcd. for $C_{29}H_{53}INaO_3Si$ $[M+Na]^+$ *m/z* 627.2701. Found: 627.2696.

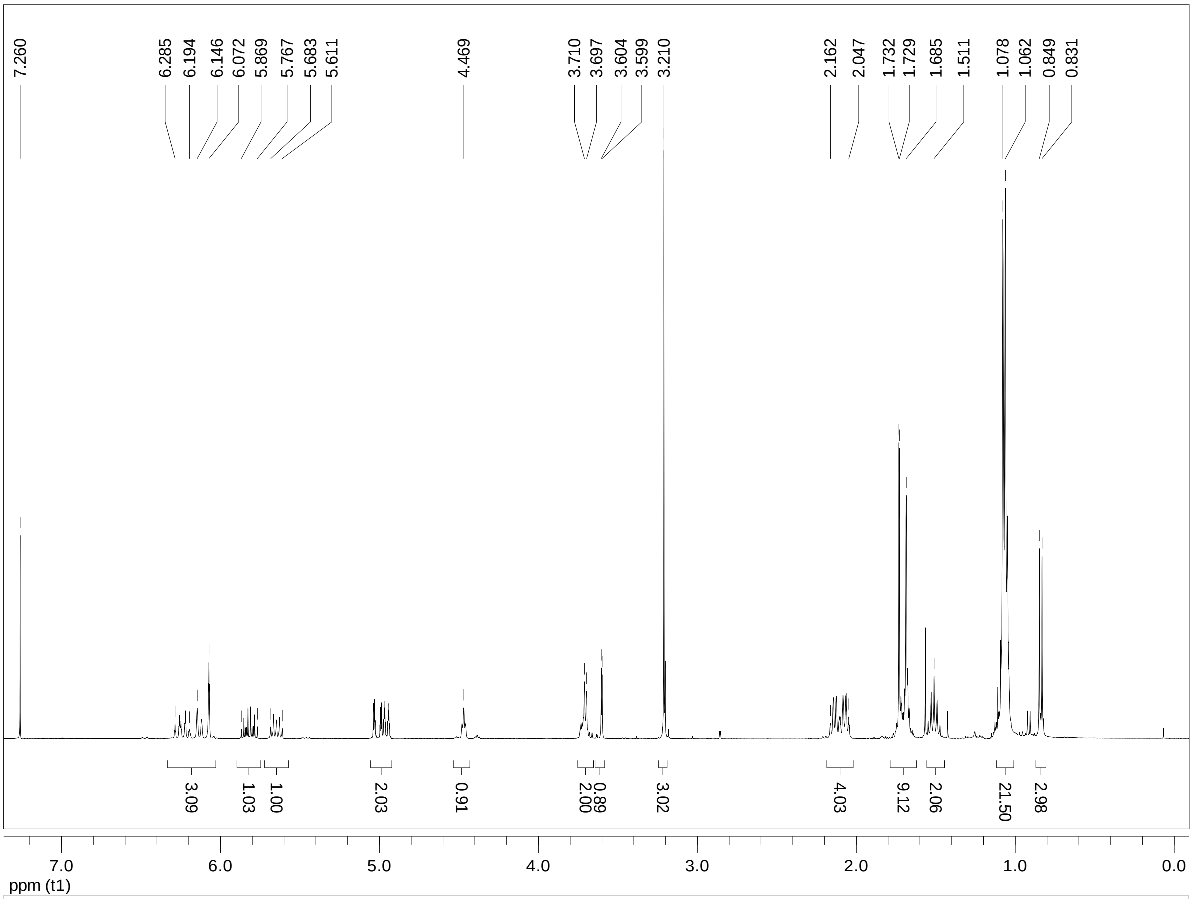
7.260
6.285
6.194
6.146
6.072
5.869
5.767
5.683
5.611
4.469
3.710
3.697
3.604
3.599
3.210
2.162
2.047
1.732
1.729
1.685
1.511
1.078
1.062
0.849
0.831
3.09
1.03
1.00
2.03
0.91
2.00
0.89
3.02
4.03
9.12
2.06
21.50
2.98
7.0
6.0
5.0
4.0
3.0
2.0
1.0
0.0
ppm (t1)

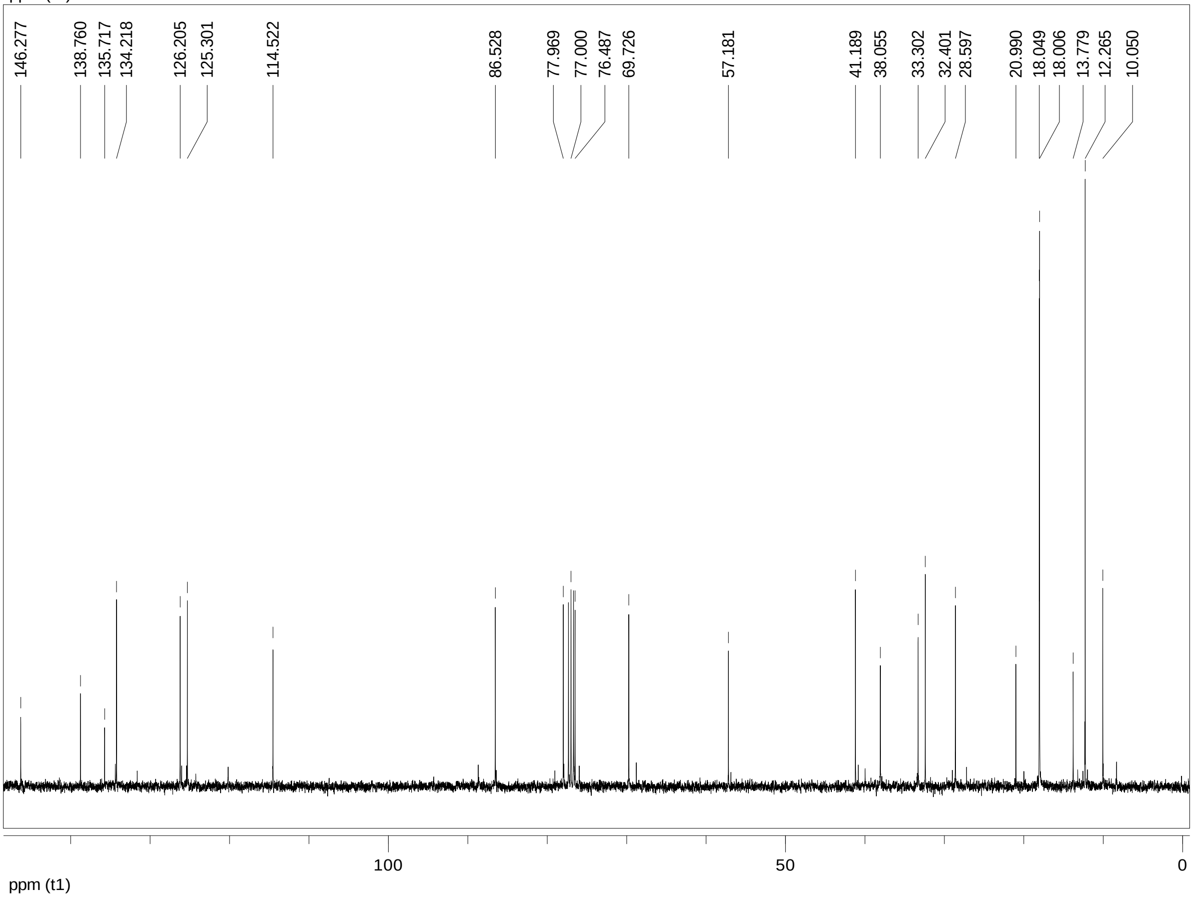
146.277
138.760
135.717
134.218
126.205
125.301
114.522
86.528
77.969
77.000
76.487
69.726
57.181
41.189
38.055
33.302
32.401
28.597
20.990
18.049
18.006
13.779
12.265
10.050
100
50
0
ppm (t1)

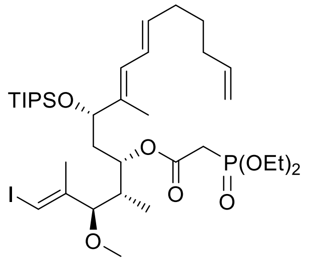

S18

(1*E*,3*R*,4*S*,5*S*,7*S*,8*E*,10*E*)-1-Iodo-3-methoxy-2,4,8-trimethyl-7-((triisopropylsilyl)-oxy)hexadeca-1,8,10,15-tetraen-5-yl 2-(diethoxyphosphoryl)acetate (75): To a cooled (0 °C) solution of diethylphosphonoacetic acid (**55**) (36.0 µL, 1.11 mmol, 1.50 eq) in CH_2Cl_2 (2 mL) over molecular sieves (beads, 3 Å) was added CME-carbodiimide (114 mg, 0.268 mmol, 1.80 eq). The mixture was stirred at 0 °C for 10-15 min, then a solution of the alcohol **S18** (90.0 mg, 0.149 mmol, 1.00 eq) in CH_2Cl_2 (0.8 mL, rinsed with 2×0.6 mL) was added. The cooling was removed and DMAP (1.8 mg, 10 mol%) was added a few minutes later. A precipitate had formed already before the addition of DMAP. TLC after 45 min indicated full conversion. The mixture was concentrated under reduced pressure and the crude was purified by column chromatography (hex:EtOAc 2:1→1:1) to afford the desired ester **75** (95.7 mg, 82%) as a colourless oil.

TLC (hex:EtOAc 1:1): R_f = 0.61

^{1}H-NMR ($CDCl_3$, 400.1 MHz): δ = 6.22-6.08 (m, 2H), 5.89-5.71 (m, 2H), 5.62 (td, *J* = 7.1 Hz, *J* = 15.0 Hz, 1H), 5.00 (ddd, *J* = 1.6 Hz, *J* = 3.6 Hz, *J* = 17.1 Hz, 1H), 4.94 (tdd, *J* = 1.2 Hz, *J* = 2.2 Hz, *J* = 10.2 Hz, 1H), 4.75 (ddd, *J* = 1.4 Hz, *J* = 4.5 Hz, *J* = 10.3 Hz, 1H), 4.23-3.99 (m, 5H), 3.42 (d, *J* = 6.7 Hz, 1H), 3.16 (s, 3H), 2.77 (ddd, *J* = 14.3 Hz, *J* = 21.5 Hz, *J* = 41.5 Hz, 2H), 2.15-1.93 (m, 5H), 1.89-1.76 (m, 1H), 1.80 (d, *J* = 1.0 Hz, 3H), 1.72-1.61 (m, 1H), 1.68 (d, *J* = 0.6 Hz 3H), 1.47 (m_c, 2H), 1.32 (t, *J* = 7.1 Hz, 3H), 1.31 (t, *J* = 7.0 Hz, 3H), 1.06-0.96 (m, 21H), 0.92 (d, *J* = 6.9 Hz, 3H).

^{13}C-NMR ($CDCl_3$, 100.6 MHz): δ = 164.8 (d, *J* = 6.7 Hz), 146.4, 138.6, 137.2, 134.7, 126.0, 125.8, 114.6, 87.0, 79.0, 77.1, 73.8, 62.5 (d, *J* = 6.3 Hz), 56.7, 39.0, 35.6, 34.5 (d, *J* = 134 Hz), 33.2, 32.3, 28.6, 19.8, 18.1, 18.0, 16.32 (d, *J* = 6.2 Hz), 16.28 (d, *J* = 6.2 Hz), 12.4, 11.2, 9.7.

HRMS Calcd. for $C_{35}H_{64}INaO_7PSi$ $[M+Na]^+$ *m/z* 805.3096. Found: 805.3097.

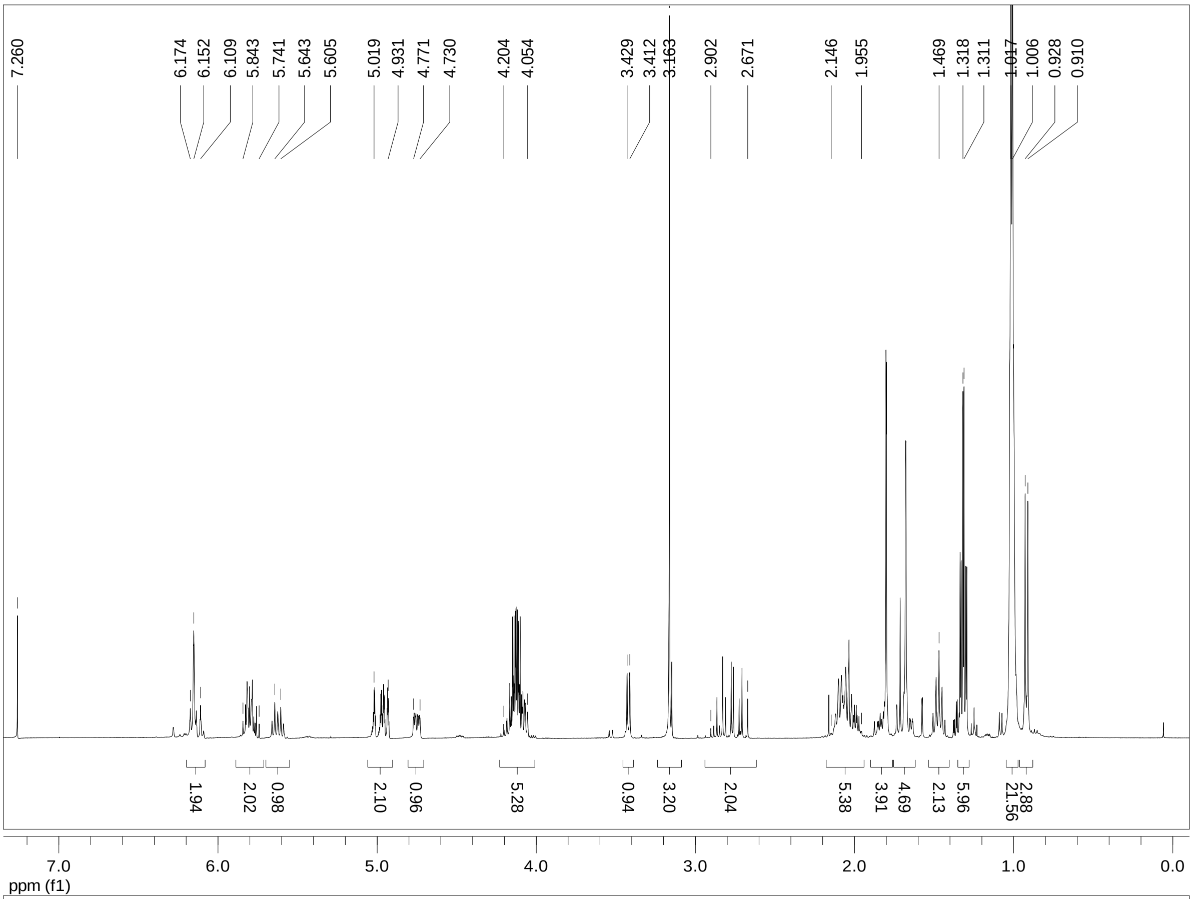
7.260
6.174
6.152
6.109
5.843
5.741
5.643
5.605
5.019
4.931
4.771
4.730
4.204
4.054
3.429
3.412
3.163
2.902
2.671
2.146
1.955
1.469
1.318
1.311
1.017
1.006
0.928
0.910
1.94
2.02
0.98
2.10
0.96
5.28
0.94
3.20
2.04
5.38
3.91
4.69
2.13
5.96
21.56
2.88
7.0
6.0
5.0
4.0
3.0
2.0
1.0
0.0
ppm (f1)

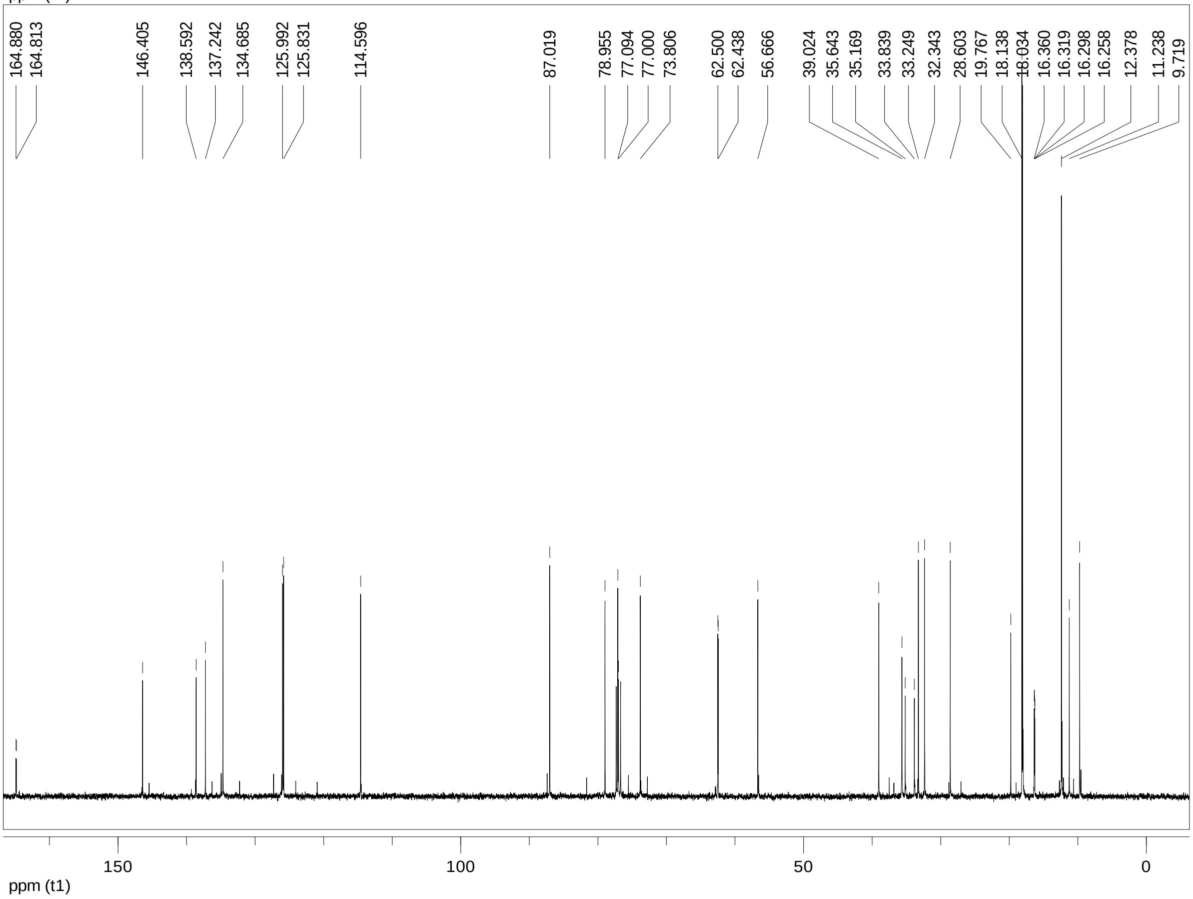
164.880
164.813
146.405
138.592
137.242
134.685
125.992
125.831
114.596
87.019
78.955
77.094
77.000
73.806
62.500
62.438
56.666
39.024
35.643
35.169
33.839
33.249
32.343
28.603
19.767
18.138
18.034
16.360
16.319
16.298
16.258
12.378
11.238
9.719
150
100
50
0
ppm (t1)

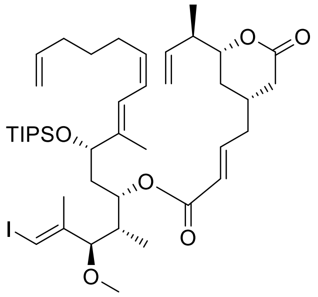

76

(1*E*,3*R*,4*S*,5*S*,7*S*,8*E*,10*Z*)-1-Iodo-3-methoxy-2,4,8-trimethyl-7-((triisopropylsilyl)-oxy)hexadeca-1,8,10,15-tetraen-5-yl (*E*)-4-((2*R*,4*R*)-2-((*R*)-but-3-en-2-yl)-6-oxotetrahydro-2H-pyran-4-yl)but-2-enoate (76): To LiCl (3.88 mg, 91.5 µmol, 1.20 eq) was added a solution of the phosphonate **S18** (71.8 mg, 91.7 µmol, 1.20 eq) in MeCN (0.6 mL), followed by DBU (13.0 µL, 84.1 µmol, 1.10 eq). After 20 min stirring at rt, the mixture was cooled to 0 °C, whereupon the solution turned turbid. Then a solution of the aldehyde **67** (15.0 mg, 76.4 µmol, 1.00 eq) in MeCN (0.3 mL, rinsed with 2×0.2 mL) was added dropwise. TLC reaction control after 45 min indicated, that no aldehyde was left. After 1 h stirring the mixture was concentrated under reduced pressure and the residue was purified by flash chromatography (hex:EtOAc 5:1→2:1) to afford the desired product **76** (48.6 mg, 85%) as a colourless oil.

TLC (hex:EtOAc 5:1): R_f = 0.45

^{1}H-NMR ($CDCl_3$, 400.1 MHz): δ = 6.80-6.69 (m, 1H), 6.16 (bs, 1H), 6.10 (m_c, 1H), 5.88-5.69 (m, 4H), 5.59 (m_c, 1H), 5.17-5.07 (m, 2H), 5.00 (ddd, *J* = 1.6 Hz, *J* = 3.6 Hz, *J* = 17.2 Hz, 1H), 4.95 (tdd, *J* = 1.2 Hz, *J* = 2.2 Hz, *J* = 10.2 Hz, 1H), 4.78 (ddd, *J* = 1.7 Hz, *J* = 4.0 Hz, *J* = 10.0 Hz, 1H), 4.17-4.07 (m, 2H), 3.38 (d, *J* = 7.2 Hz, 1H), 3.16 (s, 3H), 2.69 (m_c, 1H), 2.45 (m_c, 1H), 2.29-1.89 (m, 10H), 1.87-1.75 (m, 1H), 1.83 (d, *J* = 1.0 Hz, 3H), 1.74-1.62 (m, 1H), 1.67 (bs, 3H), 1.47 (m_c, 2H), 1.30-1.14 (m, 1H), 1.12 (d, *J* = 6.8 Hz, 3H), 1.06-0.95 (m, 21H), 0.90 (d, *J* = 6.9 Hz, 3H).

^{13}C-NMR ($CDCl_3$, 100.6 MHz): δ = 170.5, 165.2, 146.5, 144.0, 138.7, 138.4, 137.2, 134.1, 126.2, 125.9, 124.3, 116.5, 114.6, 87.6, 83.2, 79.0, 76.5, 72.6, 56.7, 42.7, 38.9, 38.7, 36.0, 35.8, 33.2, 32.4, 31.9, 31.0, 28.6, 19.7, 18.2, 18.1, 15.3, 12.5, 11.5, 9.8.

HRMS Calcd. for $C_{42}H_{73}INO_6Si$ $[M+NH_4]^+$ *m/z* 842.4246. Found: 842.4250.

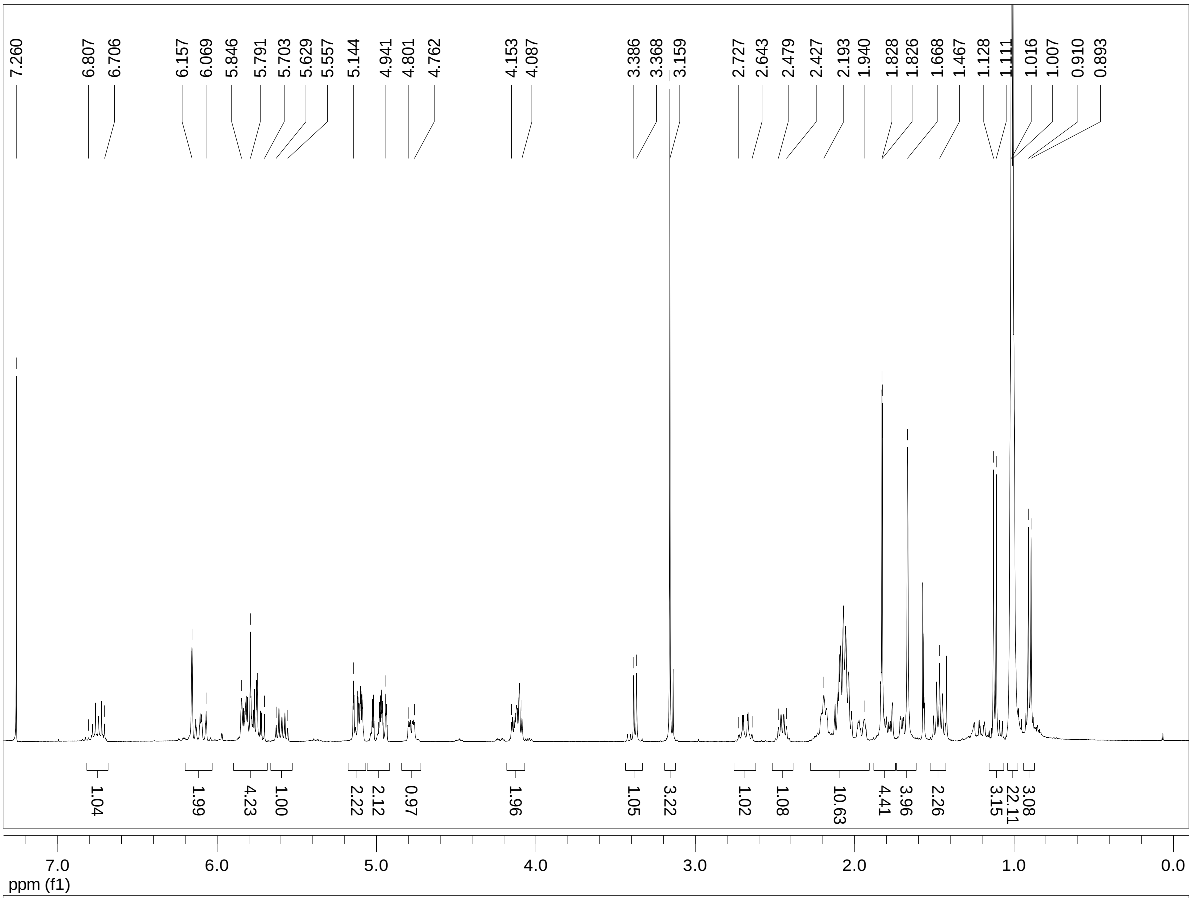
7.260
6.807
6.706
6.157
6.069
5.846
5.791
5.703
5.629
5.557
5.144
4.941
4.801
4.762
4.153
4.087
3.386
3.368
3.159
2.727
2.643
2.479
2.427
2.193
1.940
1.828
1.826
1.668
1.467
1.128
1.111
1.016
1.007
0.910
0.893
1.04
1.99
4.23
1.00
2.22
2.12
0.97
1.96
1.05
3.22
1.02
1.08
10.63
4.41
3.96
2.26
3.15
22.11
3.08
7.0
6.0
5.0
4.0
3.0
2.0
1.0
0.0
ppm (f1)

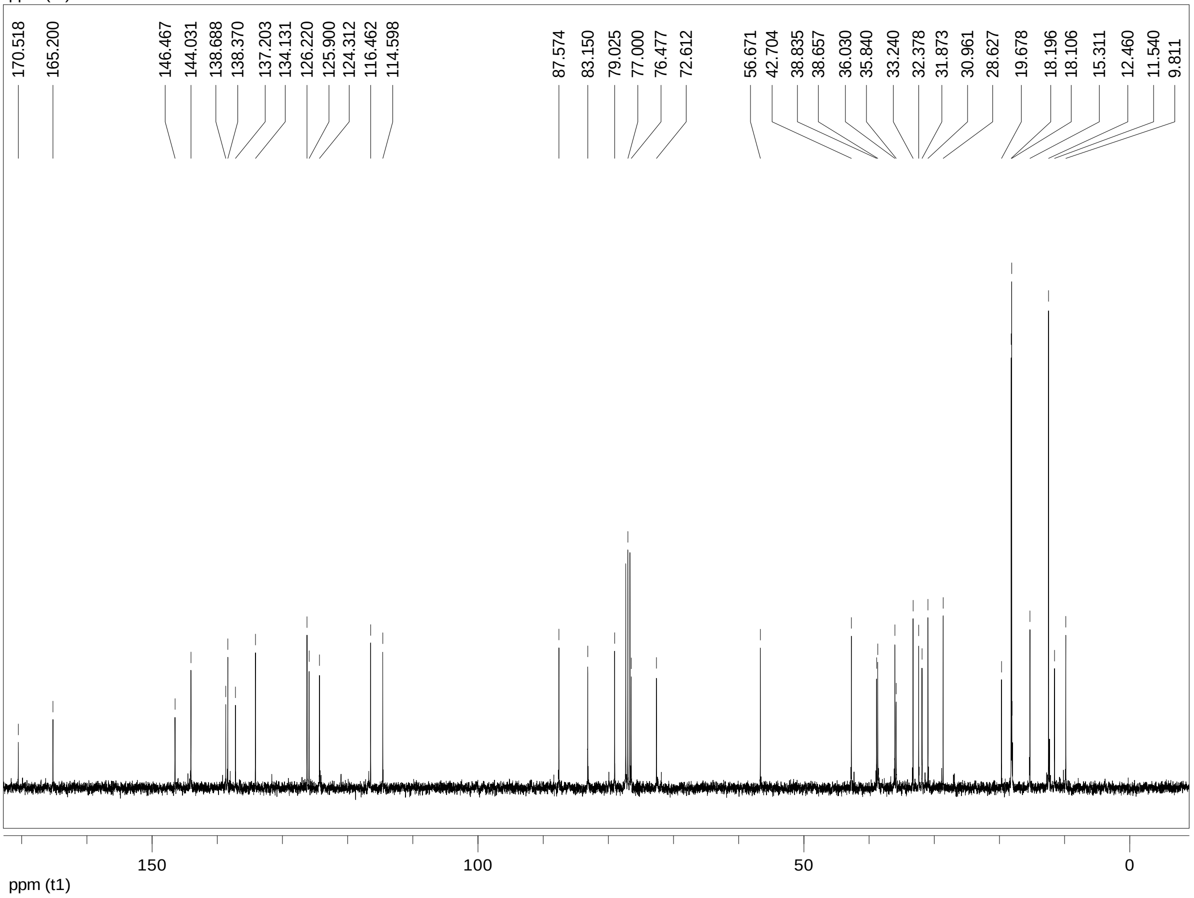
170.518
165.200
146.467
144.031
138.688
138.370
137.203
134.131
126.220
125.900
124.312
116.462
114.598
87.574
83.150
79.025
77.000
76.477
72.612
56.671
42.704
38.835
38.657
36.030
35.840
33.240
32.378
31.873
30.961
28.627
19.678
18.196
18.106
15.311
12.460
11.540
9.811
150
100
50
0
ppm (t1)

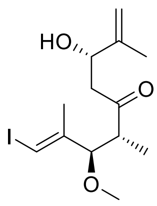

78

(3*R*,4*R*,7*S*,*E*)-7-Hydroxy-1-iodo-3-methoxy-2,4,8-trimethylnona-1,8-dien-5-one (78): A solution of (+)-DIP-Cl (342 mg, 1.07 mmol, 2.00 eq) in CH_2Cl_2 (0.4 mL) was cooled to −78 °C. Upon cooling, the solution froze which rendered the mixture instirrable. Next NEt_3 (0.177 mL, 1.28 mmol, 2.40 eq), followed by a solution of ketone **27** (150 mg, 0.532 mmol, 1.00 eq) in CH_2Cl_2 (0.2 mL, rinsed with 2×0.2 mL) were added. After 1 h 40 min the cooling was removed and, after a few minutes, replaced by an ice bath. This led to formation of a thick suspension, which was slightly stirrable. After 5 min at 0 °C, the mixture was recooled to −78 °C. It was more homogeneous than before warming, but still hardly stirrable. After a total of 2 h 45 min, neat methacrolein (**77**) (88 µL, 1.06 mmol, 2.00 eq) was added. No change was observed, except that stirring was slightly facilitated. The mixture was stirred for another 50 min at −78 °C, and was then aged in a freezer (−18 °C) for ca. 3.5 d. Afterwards the reaction was quenched with pH 7 phosphate buffer and the layers were separated. The aqueous phase was diluted with phosphate buffer and extracted with CH_2Cl_2 (3×10 mL). The combined extracts were concentrated under reduced pressure. The residue was dissolved in MeOH (3 mL) and pH 7 phosphate buffer (0.6 mL) was added. After cooling to 0 °C H_2O_2 (30%, 0.75 mL) was added and the mixture was stirred at 0 °C for 45 min and at rt for 15 min. Afterwards it was poured into water (40 mL). CH_2Cl_2 was added and the layers were separated. The aqueous phase was extracted with CH_2Cl_2 (3×10 mL). The combined organic extracts were washed with brine, dried over Na_2SO_4 and concentrated under reduced pressure. The crude was purified by column chromatography (hex:EtOAc 5:1) to afford the desired product **78** (312 mg, 40% wt/wt along with isopinocampheol/Et_2O/EtOAc, 125 mg, 67%, dr=19.6:1) as a nearly colourless oil.

TLC (hex:EtOAc 5:1): R_f = 0.17

^{1}H-NMR ($CDCl_3$, 400.1 MHz): δ = 6.22 (m_c, 1H), 5.02 (m_c, 1H), 4.87 (m_c, 1H), 4.45 (td, *J* = 2.8 Hz, *J* = 8.6 Hz, 1H), 3.84 (dd, *J* = 7.4 Hz, *J* = 0.4 Hz, 1H), 3.21 (s, 3H), 2.93 (d, *J* = 3.3 Hz, 1H), 2.77 (p, *J* = 7 Hz, 1H), 2.71-2.54 (m, 2H), 1.76 (d, *J* = 1.1 Hz, 3H), 1.74 (m_c, 3H), 1.13 (d, *J* = 7.4 Hz, 3H)

^{13}C-NMR ($CDCl_3$, 100.6 MHz): δ = 212.7, 145.5, 145.2, 111.2, 86.3, 80.6, 70.8, 57.0, 50.0, 47.6, 19.8, 18.4, 12.1.

HRMS Calcd. for $C_{13}H_{21}INaO_3$ $[M+Na]^+$ *m/z* 375.0428. Found: 375.0435.

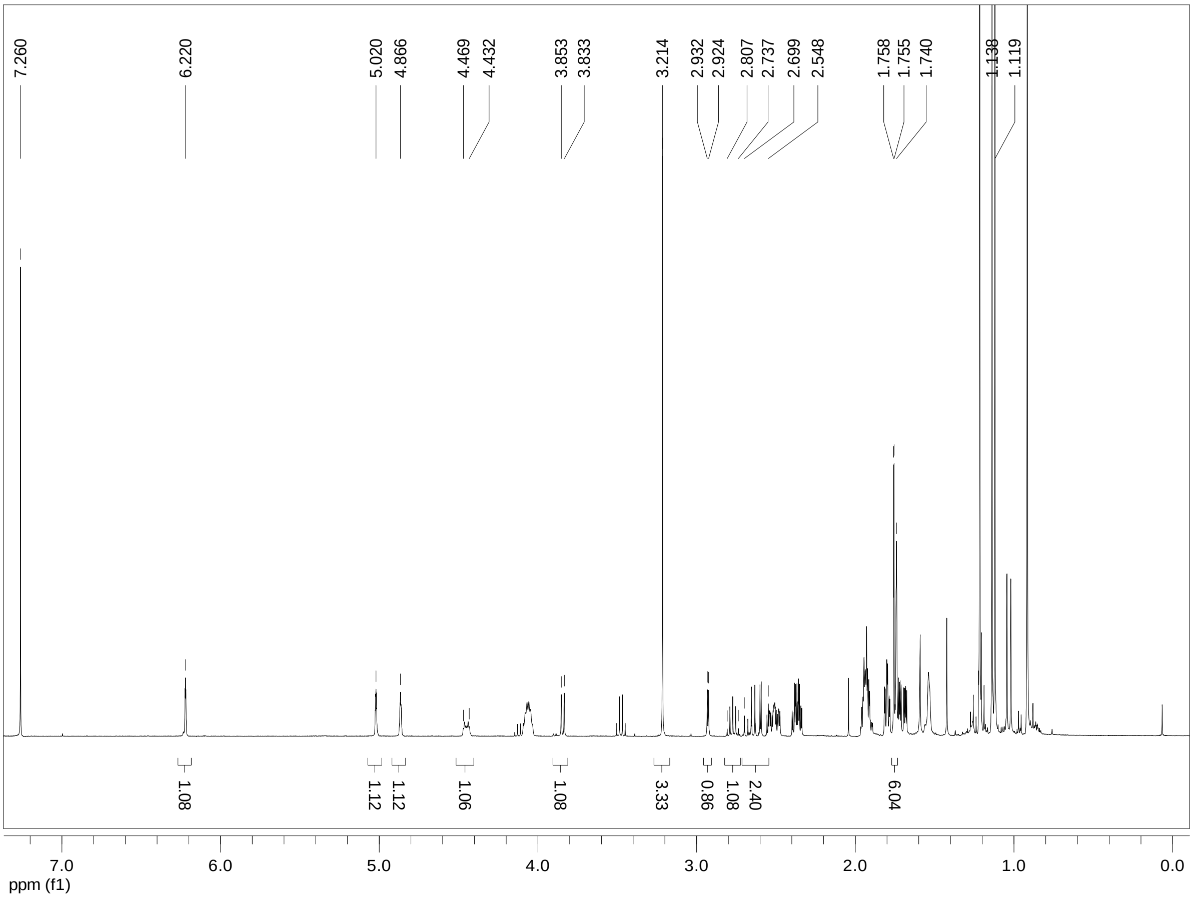
7.260
6.220
5.020
4.866
4.469
4.432
3.853
3.833
3.214
2.932
2.924
2.807
2.737
2.699
2.548
1.758
1.755
1.740
1.138
1.119
1.08
1.12
1.12
1.06
1.08
3.33
0.86
1.08
2.40
6.04
7.0 6.0 5.0 4.0 3.0 2.0 1.0 0.0
ppm (f1)

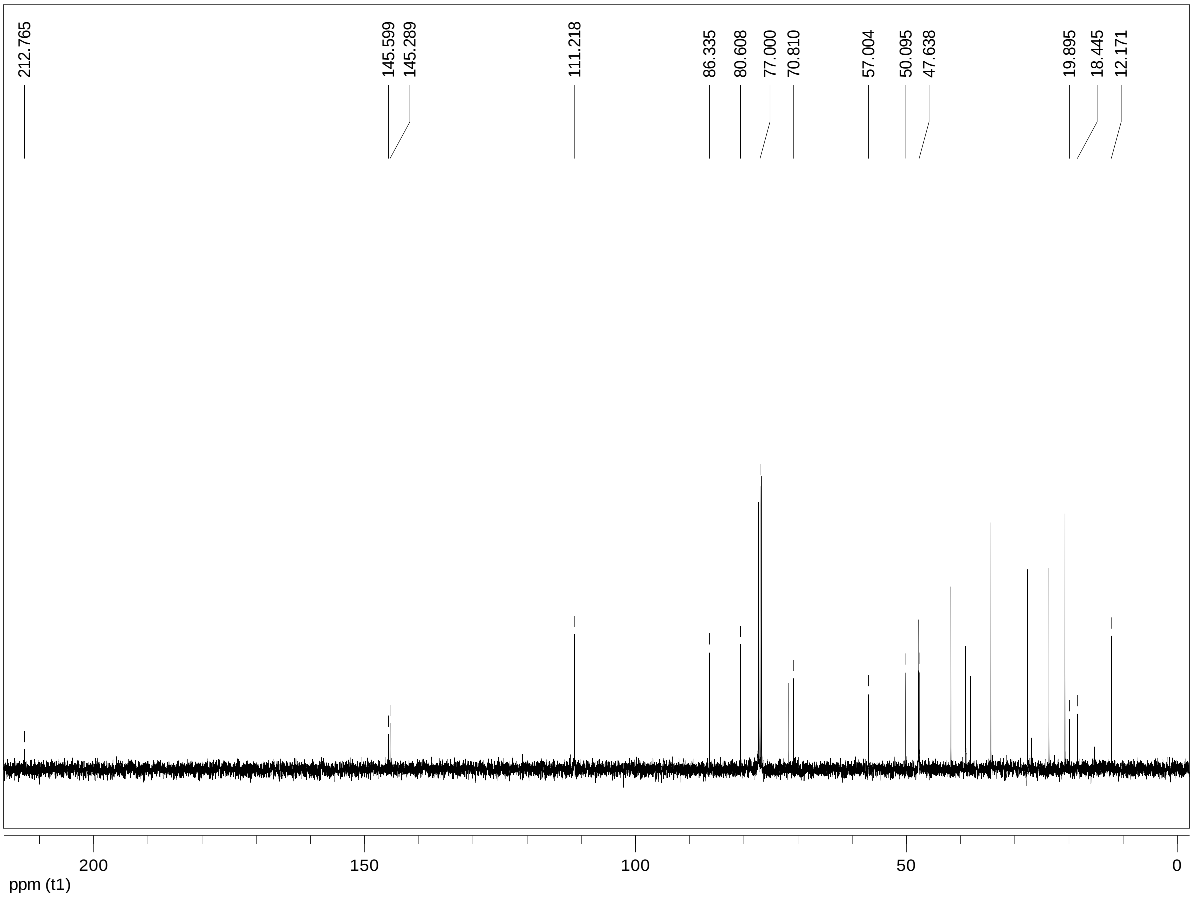
212.765
145.599
145.289
111.218
86.335
80.608
77.000
70.810
57.004
50.095
47.638
19.895
18.445
12.171
200 150 100 50 0
ppm (t1)

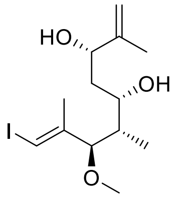

S19

(3*S*,5*S*,6*S*,7*R*,*E*)-9-Iodo-7-methoxy-2,6,8-trimethylnona-1,8-diene-3,5-diol (S19): A solution of tetramethylammonium triacetoxyborohydride (417 mg, 4.65 eq) in MeCN (1.4 mL) and AcOH (1.4 mL) was cooled to −40 °C (MeCN/dry ice). To the frozen mixture was added a solution of the ketone **78** (120 mg, 341 µmol, 1.00 eq) in MeCN (0.4 mL, rinsed with 2×0.4 mL). The resulting thick slurry was stirred for a few minutes at the same temperature and was then aged in a freezer (−18 °C) for 18 h. Afterwards the suspension was allowed to warm to rt and stirred for another 1 h 15 min. Then *Rochelle* salt (aq. sat.) was added. A white suspension formed immediately. This mixture was stirred at rt for 2 h. Then it was diluted with water, some CH_2Cl_2 was added and the acetic acid was quenched with $NaHCO_3$ (s). As soon as the gas evolution stopped, the layers were separated. The aqueous phase was extracted with CH_2Cl_2 (3×10 mL). The combined organic extracts were washed with brine, dried over $MgSO_4$ and concentrated under reduced pressure. The crude was purified by column chromatography (hex:Et_2O 2:1→hex:EtOAc 1:1) to afford the desired product **S20** (121 mg, 95% wt/wt along with EtOAc, 115 mg, 95%, dr=49:1) as a colourless oil.

TLC (Hex:EtOAc 1:1): R_f = 0.64
[α]$_D^{24}$ = +44.2 (*c* = 1.00, $CHCl_3$)
^{1}H-NMR ($CDCl_3$, 400.1 MHz): δ = 6.15 (m_c, 1H), 5.07 (bs, 1H), 4.91 (m_c, 1H), 4.37 (m_c, 1H), 3.95 (d, *J* = 3.6 Hz, 1H), 3.88-3.78 (m, 1H), 3.27 (s, 3H), 3.25 (d, *J* = 5.5 Hz, 1H), 2.74-2.68 (m, 1H), 1.83-1.68 (m, 3H), 1.76 (d, *J* = 1.0 Hz, 3H), 1.73 (bs, 3H), 0.85 (d, *J* = 7.1 Hz, 3H).
^{13}C-NMR ($CDCl_3$, 100.6 MHz): δ = 147.3, 145.3, 110.4, 86.5, 78.2, 73.1, 71.2, 57.3, 40.7, 38.2, 21.7, 18.8, 10.9.
IR (ν/[cm^{-1}]) 3385br, 3073, 2974, 2935, 2918, 2830, 1738, 1725, 1651, 1620, 1443, 1376, 1243, 1193, 1113, 1087s, 1049s, 1010, 948, 931, 921, 899, 852, 785, 683, 669, 647, 637, 597, 579, 569, 550, 542, 535, 515, 505, 499, 492, 483, 469, 459, 440, 424.
HRMS Calcd. for $C_{13}H_{23}INaO_3$ [M+Na]$^+$ *m/z* 377.0584. Found: 377.0571.

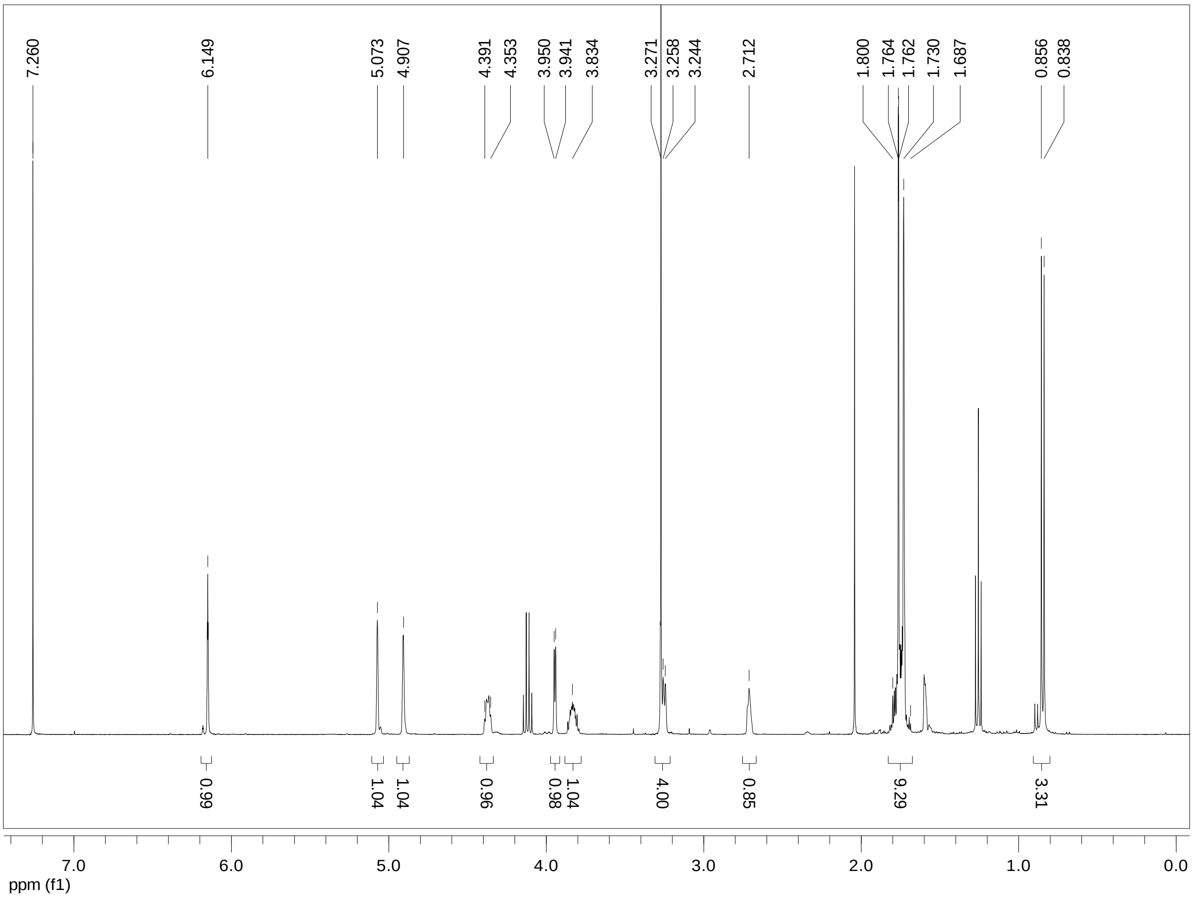
7.260
6.149
5.073
4.907
4.391
4.353
3.950
3.941
3.834
3.271
3.258
3.244
2.712
1.800
1.764
1.762
1.730
1.687
0.856
0.838
0.99
1.04
1.04
0.96
0.98
1.04
4.00
0.85
9.29
3.31
7.0
6.0
5.0
4.0
3.0
2.0
1.0
0.0
ppm (f1)

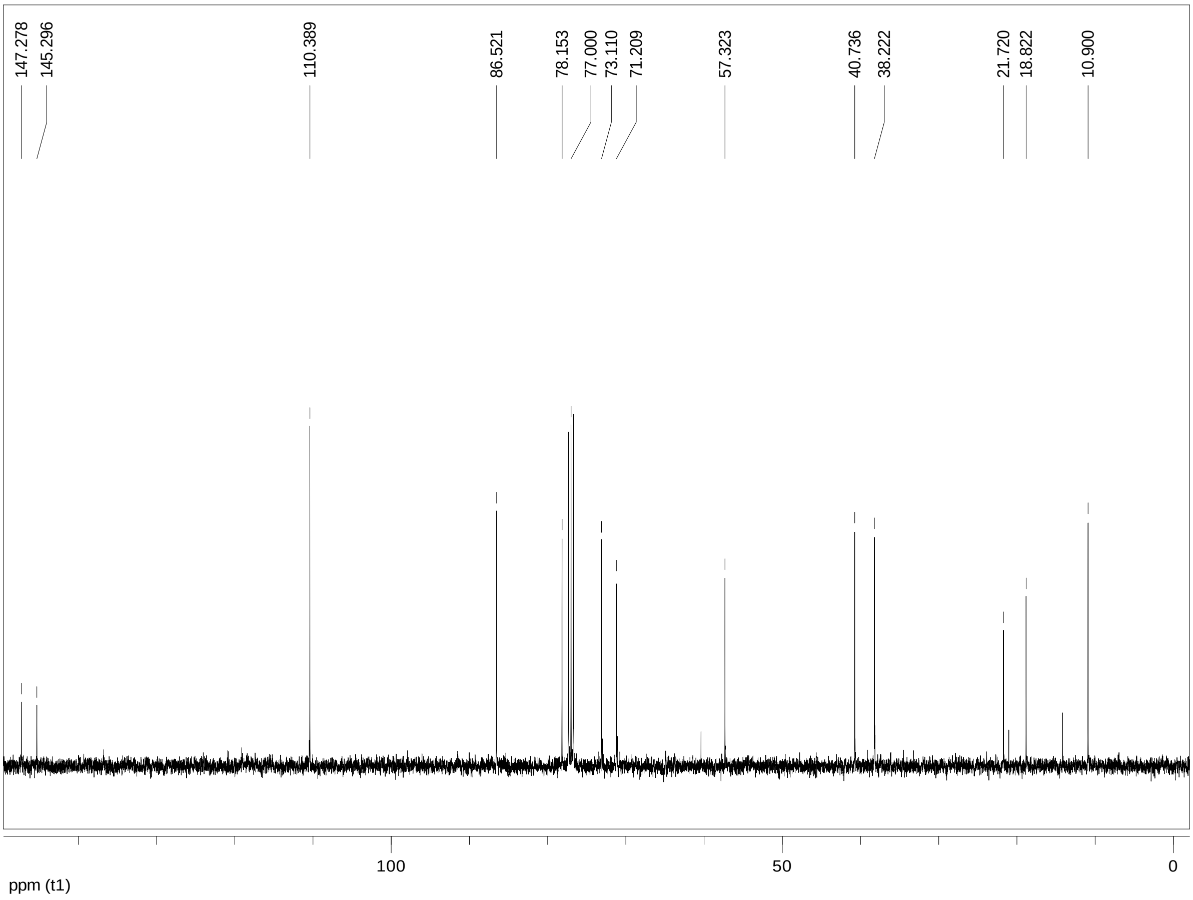
147.278
145.296
110.389
86.521
78.153
77.000
73.110
71.209
57.323
40.736
38.222
21.720
18.822
10.900
100
50
0
ppm (t1)

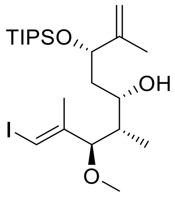

79

(3*R*,4*S*,5*S*,7*S*,*E*)-1-Iodo-3-methoxy-2,4,8-trimethyl-7-((triisopropylsilyl)oxy)nona-1,8-dien-5-ol (79): To a solution of the diol **S19** (104 mg, 0.294 mmol, 1.00 eq) in CH_2Cl_2 (8 mL) over molecular sieves (3 Å beads) was added 2,6-lutidine (171 µL, 1.47 mmol, 5.00 eq) at rt. The mixture was stirred at rt for 5 min, then it was cooled to −78 °C and, after another 5 min stirring, TIPSOTf (87.0 µL, 0.323 mmol, 1.10 eq) was added. TLC reaction control after 25 min indicated nearly full conversion. After 35 min the reaction was quenched with $NaHCO_3$ (sat. aq.). The layers were separated and the aqueous phase was extracted with CH_2Cl_2 (3×10 mL). Then the combined organic extracts were washed with brine, dried over $MgSO_4$ and the solvent was removed under reduced pressure. The crude was purified by column chromatography (hex:Et2O 7:1) to afford the desired product **79** as a colourless oil (146 mg, 97%).

TLC (hex:EtOAc 5:1): R_f = 0.52; **$[\alpha]_D^{24}$** = +16.43 (*c* = 0.85, $CHCl_3$); **^{1}H-NMR** ($CDCl_3$, 400.1 MHz): δ = 6.08 (m_c, 1H), 5.14 (m_c, 1H), 4.98 (m_c, 1H), 4.49 (t, *J* = 4.1 Hz, 1H), 3.80-3.68 (m, 3H), 3.22 (s, 3H), 1.79-1.64 (m, 3H), 1.74 (d, *J* = 1.0 Hz, 3H), 1.69 (bs, 3H), 1.13-1.02 (m, 21H), 0.84 (d, *J* = 7.0 Hz, 3H); **^{13}C{^{1}H}-NMR** ($CDCl_3$, 100.6 MHz): δ = 146.4, 145.5, 111.6, 86.4, 77.9, 75.6, 69.5, 57.2, 41.2, 37.1, 20.9, 19.3, 18.04, 18.02, 12.2, 9.9; **IR** (ν/[cm^{-1}]) 3518br, 2942, 2893, 2867, 1654, 1620, 1462, 1379, 1256, 1193, 1112, 1082s, 1064s, 998, 952, 919, 883s, 818, 784, 764, 725, 719, 679s, 658, 596, 569, 561, 510, 499, 485, 463, 446, 433, 424, 410, 404; **HRMS** Calcd. for $C_{22}H_{44}IO_3Si$ $[M+H]^+$ *m/z* 511.2099. Found: 511.2103.

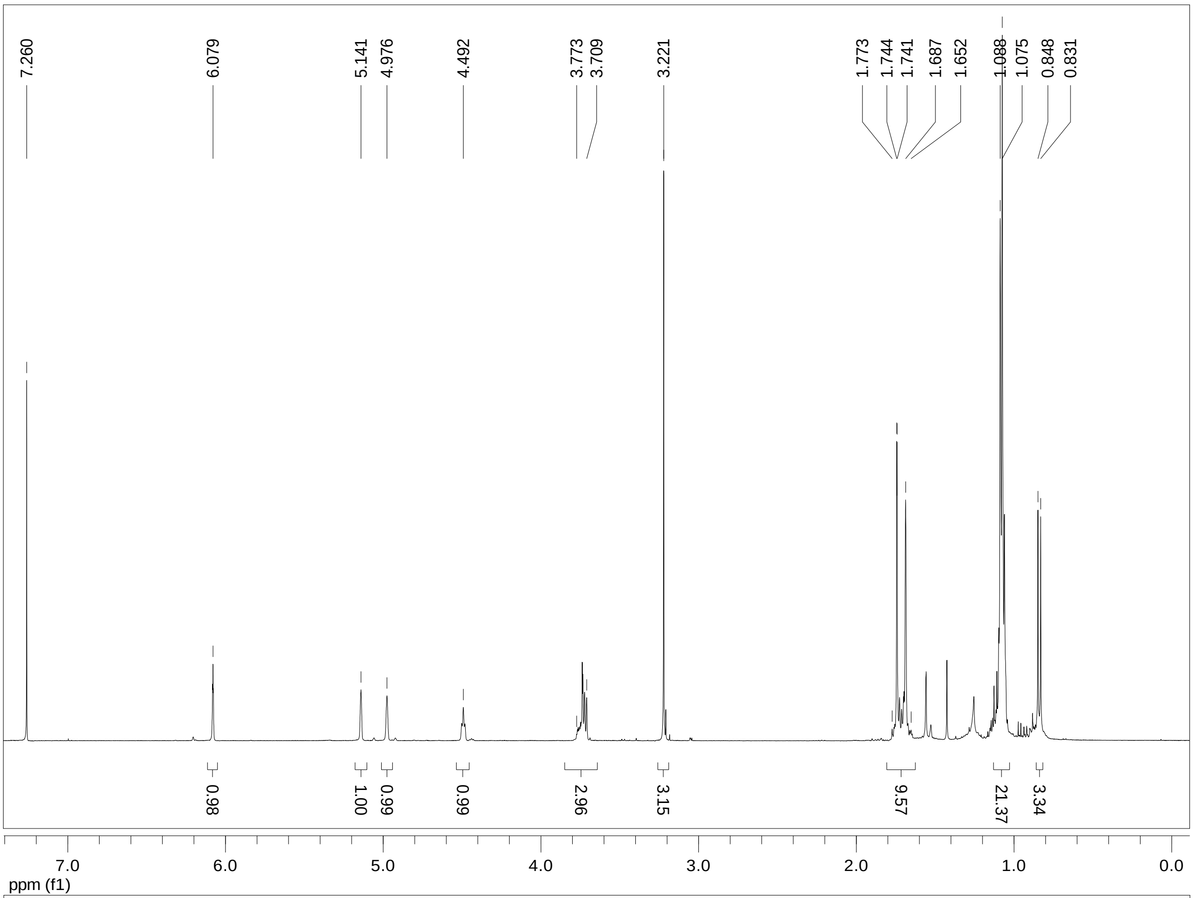
7.260
6.079
5.141
4.976
4.492
3.773
3.709
3.221
1.773
1.744
1.741
1.687
1.652
1.088
1.075
0.848
0.831
0.98
1.00
0.99
0.99
2.96
3.15
9.57
21.37
3.34
7.0
6.0
5.0
4.0
3.0
2.0
1.0
0.0
ppm (f1)

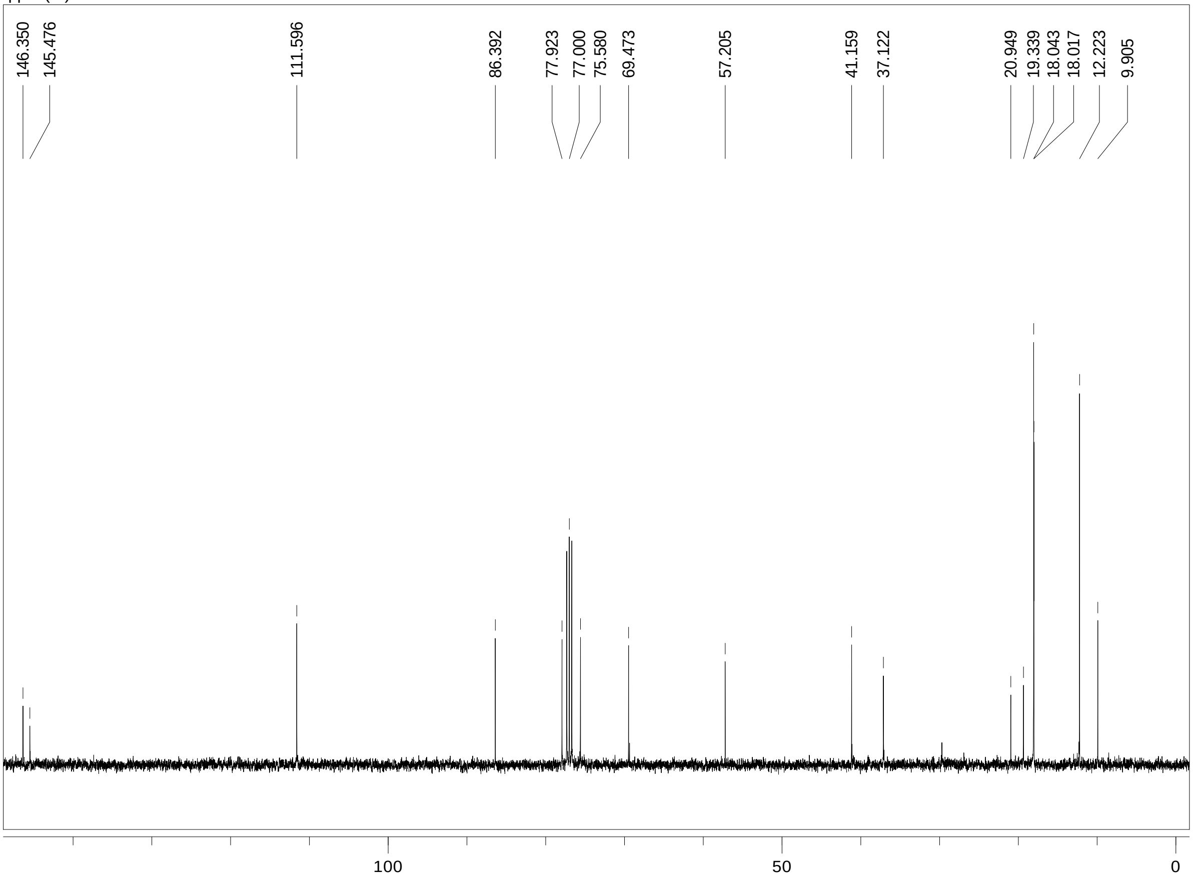
146.350
145.476
111.596
86.392
77.923
77.000
75.580
69.473
57.205
41.159
37.122
20.949
19.339
18.043
18.017
12.223
9.905
100
50
0
ppm (t1)

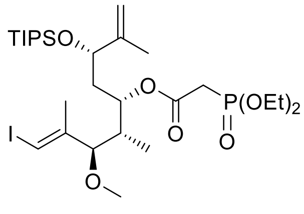

80

(3*R*,4*S*,5*S*,7*S*,*E*)-1-Iodo-3-methoxy-2,4,8-trimethyl-7-((triisopropylsilyl)oxy)nona-1,8-dien-5-yl 2-(diethoxyphosphoryl)acetate (80): To a cooled (0 °C) solution of diethylphosphonoacetic acid (**55**) (66 µL, 0.413 mmol, 1.50 eq) in CH_2Cl_2 (3.5 mL) over molecular sieves (beads, 3 Å) was added CME-carbodiimide (210 mg, 0.496 mmol, 1.80 eq). The mixture was stirred at 0 °C for 10-15 min, then a solution of the alcohol **79** (141 mg, 0.275 mmol, 1.00 eq) in CH_2Cl_2 (1.1 mL, rinsed with 2×1.1 mL) was added. The cooling was removed and DMAP (3.4 mg, 10 mol%) was added. After 2.5 h the mixture was concentrated under reduced pressure. The crude was purified by column chromatography (hex:EtOAc 2:1→1:1) to afford the desired ester **80** (154 mg, 81%) as a colourless oil.

TLC (Hex:EtOAc 1:1): R_f = 0.43

$[\alpha]_D^{24}$ = +18.5 (c = 0.905, $CHCl_3$)

^{1}H-NMR ($CDCl_3$, 400.1 MHz): δ = 6.16 (m_c, 1H), 4.80 (m_c, 1H), 4.75 (ddd, J = 1.7 Hz, J = 4.4 Hz, J = 10.0 Hz, 1H), 4.71 (m_c, 1H), 4.20-4.10 (m, 5H), 3.43 (d, J = 6.6 Hz, 1H), 3.17 (s, 3H), 2.85 (ddd, J = 14.4 Hz, J = 21.5 Hz, J = 34.1 Hz, 2H), 2.02 (m_c, 1H), 1.89-1.78 (m, 1H), 1.81 (d, J = 1.1 Hz, 3H), 1.74-1.64 (m, 1H), 1.70 (d, J = 0.4 Hz, 3H), 1.33 (m_c, 6H), 1.07-1.00 (m, 21H), 0.94 (d, J = 6.9 Hz, 3H).

^{13}C-NMR ($CDCl_3$, 100.6 MHz): δ = 164.9 (d, J = 6.2 Hz), 147.2, 146.5, 111.7, 87.1, 79.0, 75.6, 73.8, 62.53 (d, J = 6.3 Hz), 62.51 (d, J = 6.3 Hz), 56.7, 39.0, 35.9, 34.3 (d, J = 135 Hz), 19.8, 18.16, 18.90, 16.4, 16.34 (d, J = 6.4 Hz), 16.32 (d, J = 6.3 Hz), 12.4, 9.7.

IR (ν/[cm^{-1}]) 3072, 2942, 2866, 1734, 1651, 1614, 1462, 1385, 1264s, 1199, 1162, 1089, 1051s, 1052s, 964, 883, 825, 784, 713, 678, 656, 621, 569, 505, 500, 487, 482, 462, 422, 417.

HRMS Calcd. for $C_{28}H_{54}INaO_7PSi$ $[M+Na]^+$ m/z 711.2313. Found: 711.2

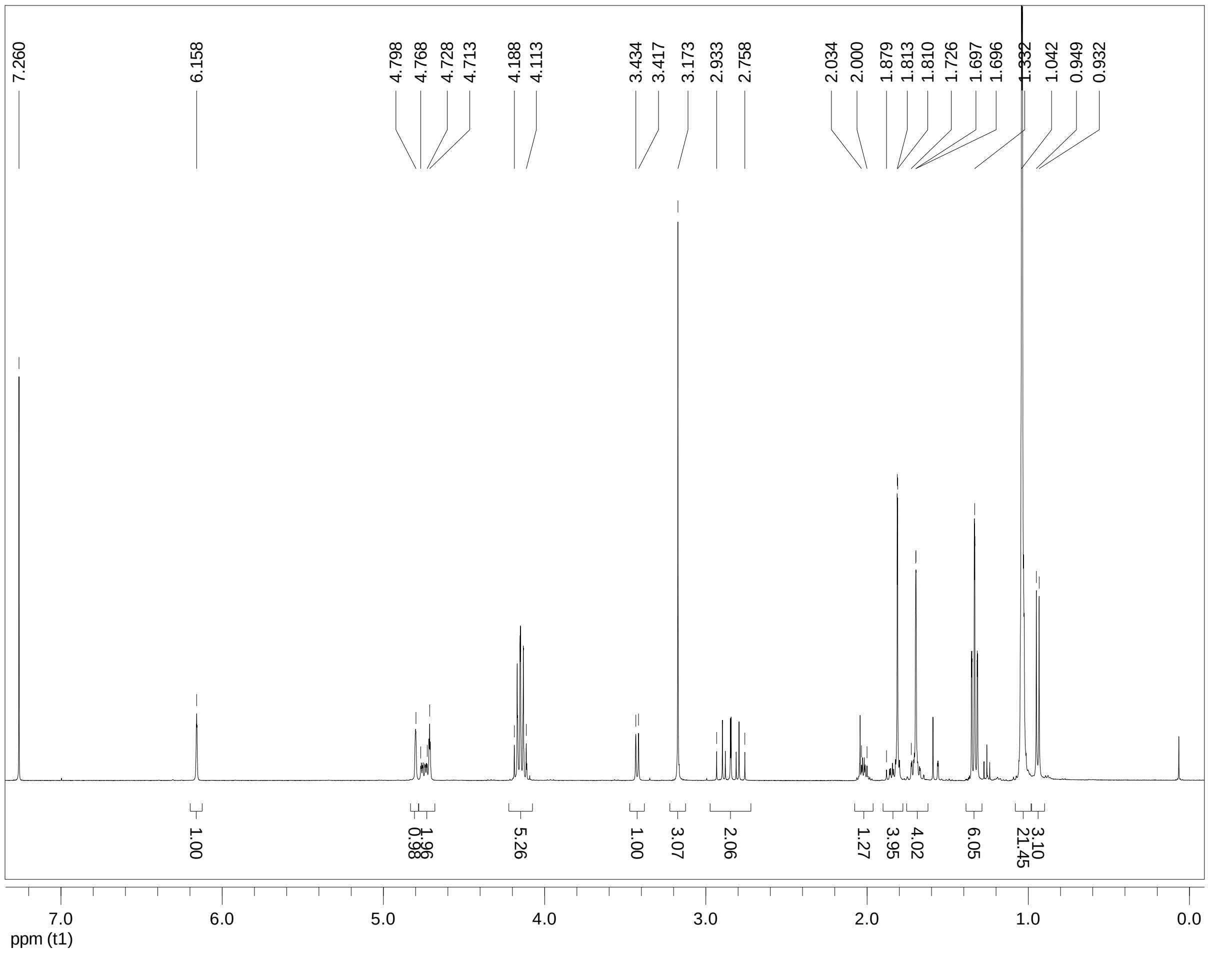
7.260
6.158
4.798
4.768
4.728
4.713
4.188
4.113
3.434
3.417
3.173
2.933
2.758
2.034
2.000
1.879
1.813
1.810
1.726
1.697
1.696
1.332
1.042
0.949
0.932
1.00
0.98
1.96
5.26
1.00
3.07
2.06
1.27
3.95
4.02
6.05
21.45
3.10
7.0
6.0
5.0
4.0
3.0
2.0
1.0
0.0
ppm (t1)

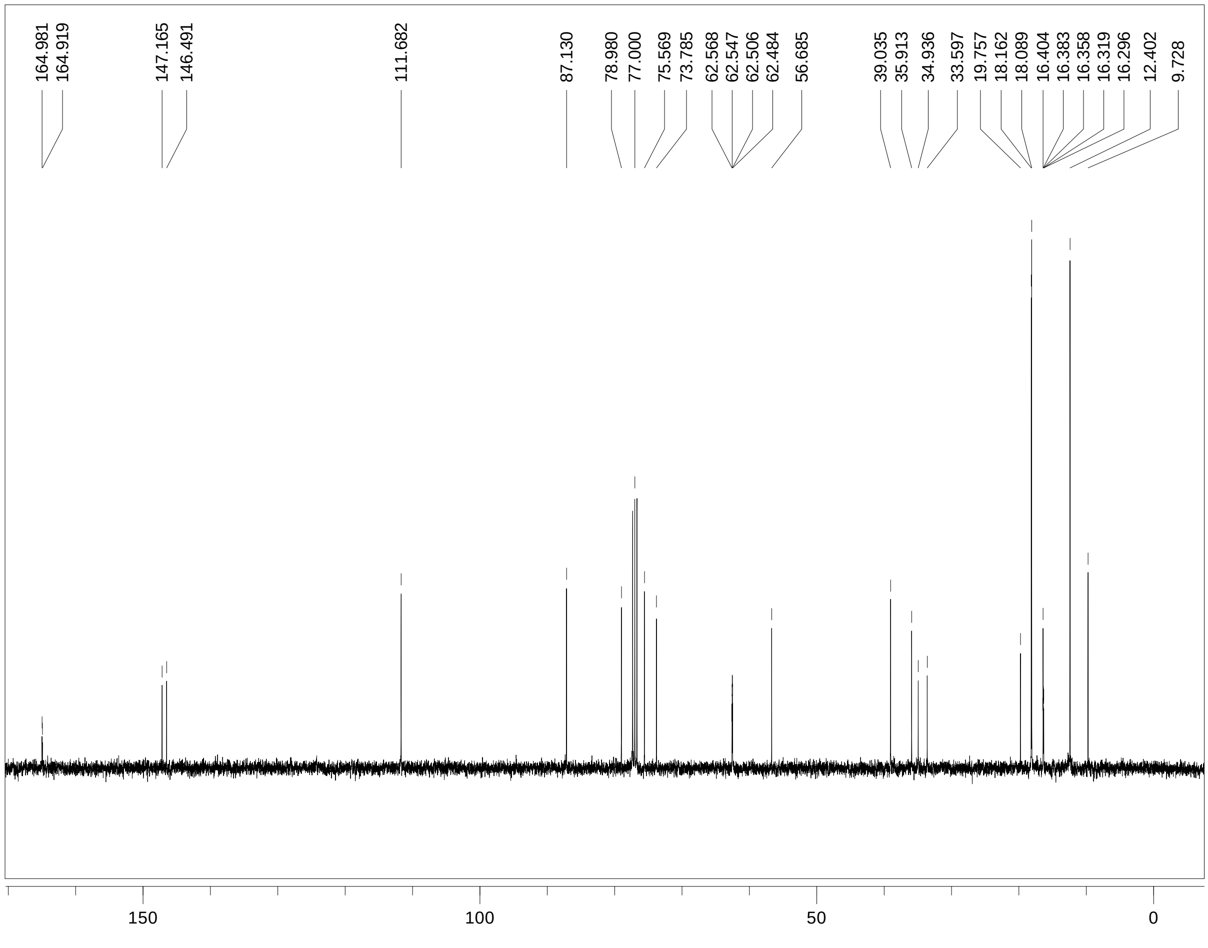
164.981
164.919
147.165
146.491
111.682
87.130
78.980
77.000
75.569
73.785
62.568
62.547
62.506
62.484
56.685
39.035
35.913
34.936
33.597
19.757
18.162
18.089
16.404
16.383
16.358
16.319
16.296
12.402
9.728
150
100
50
0

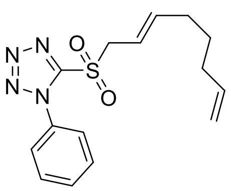

81

(*E*)-5-(Octa-2,7-dien-1-ylsulfonyl)-1-phenyl-1H-tetrazole (81): To a solution of (*E*)-2,7-octadienol (**80**) [19] (300 mg, 2.38 mmol, 1.0 equiv), 2-mercaptophenyltetrazole (509 mg, 2.86 mmol, 1.2 equiv) and triphenylphosphine (749 mg, 2.86 mmol, 1.2 equiv) in THF (12.8 mL) was added dropwise at rt DEAD (0.45 mL, 2.86 mmol, 1.2 equiv). After strirring for 3 h at rt, the reaction mixture was diluted with ethanol (21 mL) and cooled to 0 °C. Then, a premixed solution of 50% H_2O_2 (2.23 mL, 36.4 mmol, 15 equiv) and ammonium molybdate (486 mg, 0.39 mmol, 17 mol%) in water (1.5 mL) was added dropwise to the reaction mixture. After stirring over night at rt, the yellow reaction mixture was diluted with water (50 mL). The layers were separated and the aqueous layer was extracted with CH_2Cl_2 (3 x 30 mL). The combined organic extracts were washed with water and brine, dried over $MgSO_4$, filtered and concentrated under reduced pressure. The crude purified by column chromatography (hexane/EtOAc, 15:1 → 10:1) to afford **81** (436 mg, 57% over 2 steps) as a colorless oil, which solidified upon storage in the freezer to give a colorless solid.

R*f* = 0.41 (hexane/EtOAc, 5:1)

^{1}H-NMR (400.1 MHz, $CDCl_3$): δ = 7.70-7.55 (m, 5H), 5.96 (td, *J* = 6.9, 15.1 Hz, 1H), 5.74 (tdd, *J* = 6.7, 10.2, 17.0 Hz, 1H), 5.50 (dtt, *J* = 15.4, 7.4, 1.5 Hz, 1H), 5.02-4.92 (m, 2H), 4.37 (dd, *J* = 0.8, 7.4 Hz, 2H), 2.10 (m_c, 2H), 1.99 (dd, *J* = 7.0, 14.6 Hz, 2H), 1.45 (m_c, 2H).

^{13}C-NMR (100.6 MHz, $CDCl_3$): δ = 144.8, 138.0, 133.0, 131.4, 129.6, 125.1, 115.0, 113.1, 59.8, 32.9, 32.0, 27.6 (one quaternary carbon missing)

HRMS (ESI): *m/z* calcd for $C_{15}H_{19}N_4O_2S$ [M + H]$^+$: 319.1223, found: 319.1228.

[19] Isomerically pure (*E*)-2,7-octadienol was prepared from commerically available (*E/Z*)-2,7-octadienol *via* PCC oxidation and DIBAL reduction according to a literature procedure: Singh, O. V.; Han, H. *Org. Lett.* **2004**, *6*, 3067-3070.

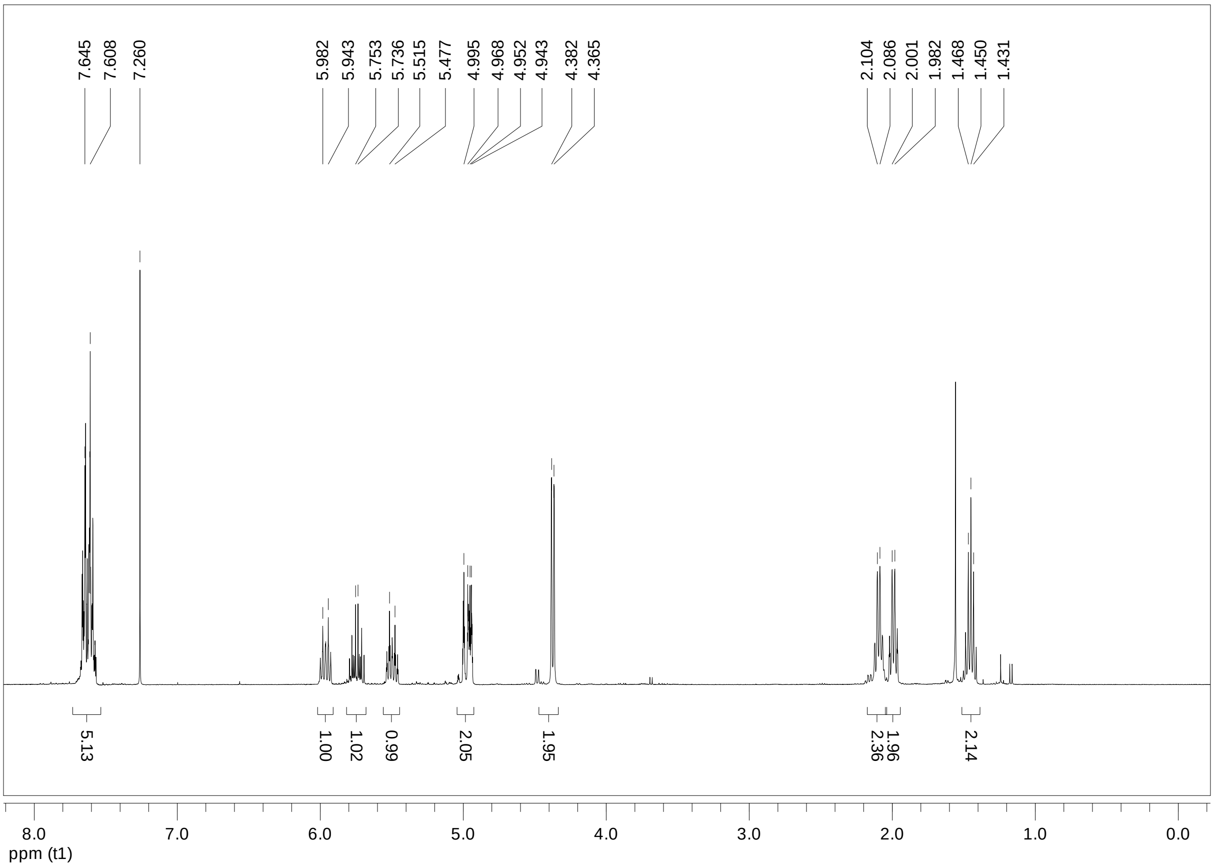
7.645
7.608
7.260
5.982
5.943
5.753
5.736
5.515
5.477
4.995
4.968
4.952
4.943
4.382
4.365
2.104
2.086
2.001
1.982
1.468
1.450
1.431
5.13
1.00
1.02
0.99
2.05
1.95
2.36
1.96
2.14
8.0
7.0
6.0
5.0
4.0
3.0
2.0
1.0
0.0
ppm (t1)

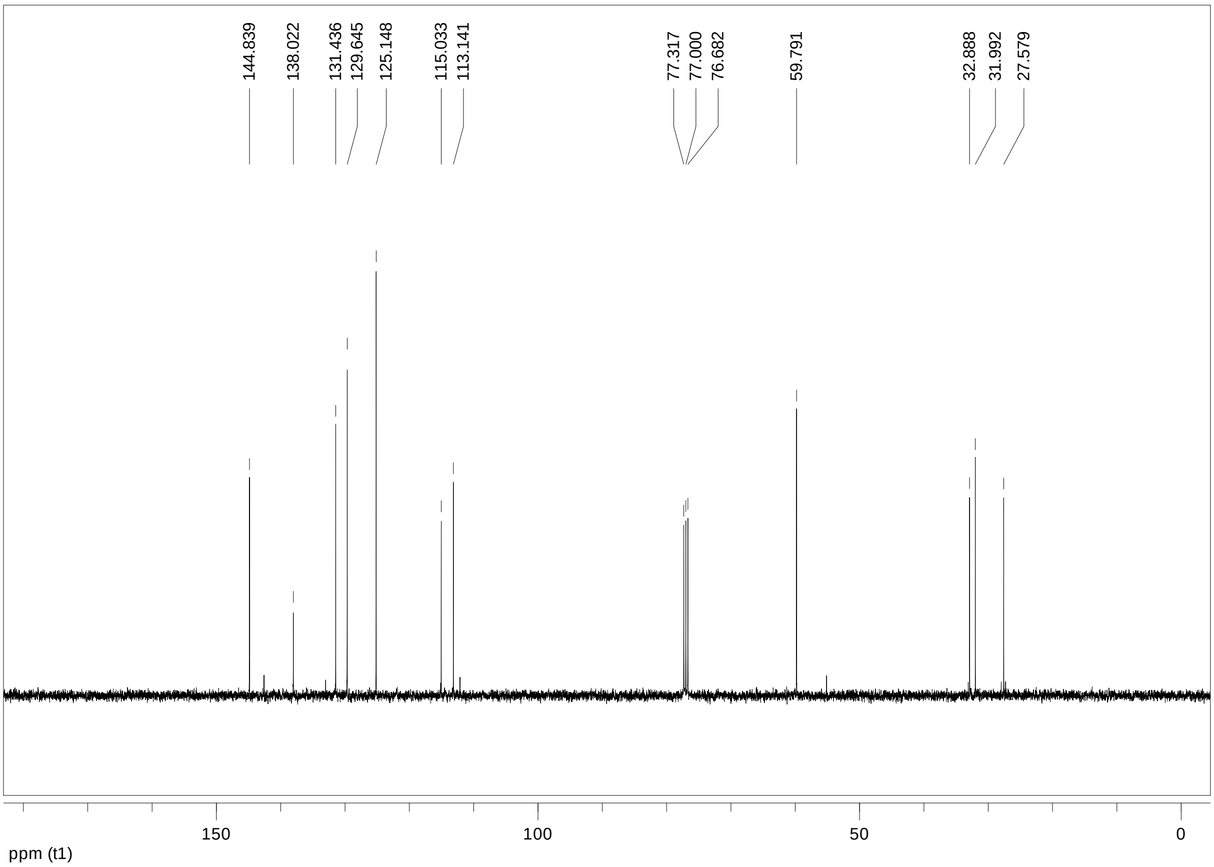
144.839
138.022
131.436
129.645
125.148
115.033
113.141
77.317
77.000
76.682
59.791
32.888
31.992
27.579
150
100
50
0
ppm (t1)

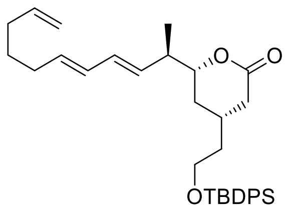

S20

(4*R*,6*R*)-4-(2-((*tert*-Butyldiphenylsilyl)oxy)ethyl)-6-((*R*,3*E*,5*E*)-undeca-3,5,10-trien-2-yl)tetrahydro-2H-pyran-2-one (*S*20): To a solution of crude aldehyde **68** (380 mg) and sulfone **81** (332 mg, 1.04 mmol, 1.3 equiv) in THF (7.6 mL) was added at –78 °C LiHMDS (2.10 mL, 1.05 mmol, 1.3 equiv, 0.5 M solution in THF). After stirring the yellowish solution at –78 °C for 1.5 h, the reaction mixture was allowed to warm to rt over 2 h. pH7 buffer solution (20 mL) was added and the mixture was diluted with Et_2O (20 mL). The layers were separated and the aqueous layer was extracted with Et_2O (2 x 25 mL). The combined organic extracts were washed with brine, dried over $MgSO_4$, filtered and concentrated under reduced pressure. The crude product was purified by column chromatography (pentane/Et_2O, 15:1 → 10:1 → 7:1 → 6:1 → 5:1) to afford **S20** (144 mg, 34% over 2 steps, E/Z >30:1) as a colorless oil.

$\mathbf{R}_f$ = 0.18 (pentane/Et_2O, 5:1)

$[\alpha]_D^{24}$ = +7.5° [*c* = 0.610, $CHCl_3$]

^{1}**H-NMR** (400.1 MHz, $CDCl_3$): δ = 7.68-7.58 (m, 4H), 7.48-7.33 (m, 6H), 6.05 (dd, *J* = 10.3, 17.2 Hz, 1H), 6.01 (dd, *J* = 10.4, 17.0 Hz, 1H), 5.80 (ddd, *J* = 6.7, 10.2, 16.9 Hz, 1H), 5.63 (td, *J* = 6.8, 13.9 Hz, 1H), 5.46 (dd, *J* = 8.0, 14.5 Hz, 1H), 5.01 (ddd, *J* = 1.6, 3.5, 17.2 Hz, 1H), 4.96 (ddd, *J* = 2.8, 6.2, 11.8 Hz, 1H), 4.08 (ddd, *J* = 2.8, 6.2, 11.8 Hz, 1H), 3.69 (t, *J* = 6.0 Hz, 2H), 2.64 (ddd, *J* = 1.7, 5.4, 17.2 Hz, 1H), 2.43 (m_c, 1H), 2.24-1.95 (m, 6H), 1.89 (d, *J* = 13.7 Hz, 1H), 1.61-1.41 (m, 4H), 1.20-1.08 (m, 1H), 1.10 (d, *J* = 6.9 Hz, 3H), 1.04 (s, 9H).

^{13}**C-NMR** (100.6 MHz, $CDCl_3$): δ = 171.4, 138.6, 135.5, 133.8, 133.6, 133.5, 131.7, 131.5, 130.2, 129.8, 127.7, 114.6, 83.9, 60.8, 41.8, 38.7, 36.4, 33.2, 32.3, 32.0, 28.5, 26.9, 19.2, 15.8.

IR (neat, ν/cm^{-1}): 3071w, 2928m, 2857m, 1736s, 1472w, 1428m, 1389w, 1235m, 1110s, 990m, 910w, 823m, 739m, 703s, 615m, 505s, 488m.

HRMS (ESI): *m/z* calcd for $C_{34}H_{46}NaO_3Si$ $[M + Na]^+$: 553.318, found: 553.3109.

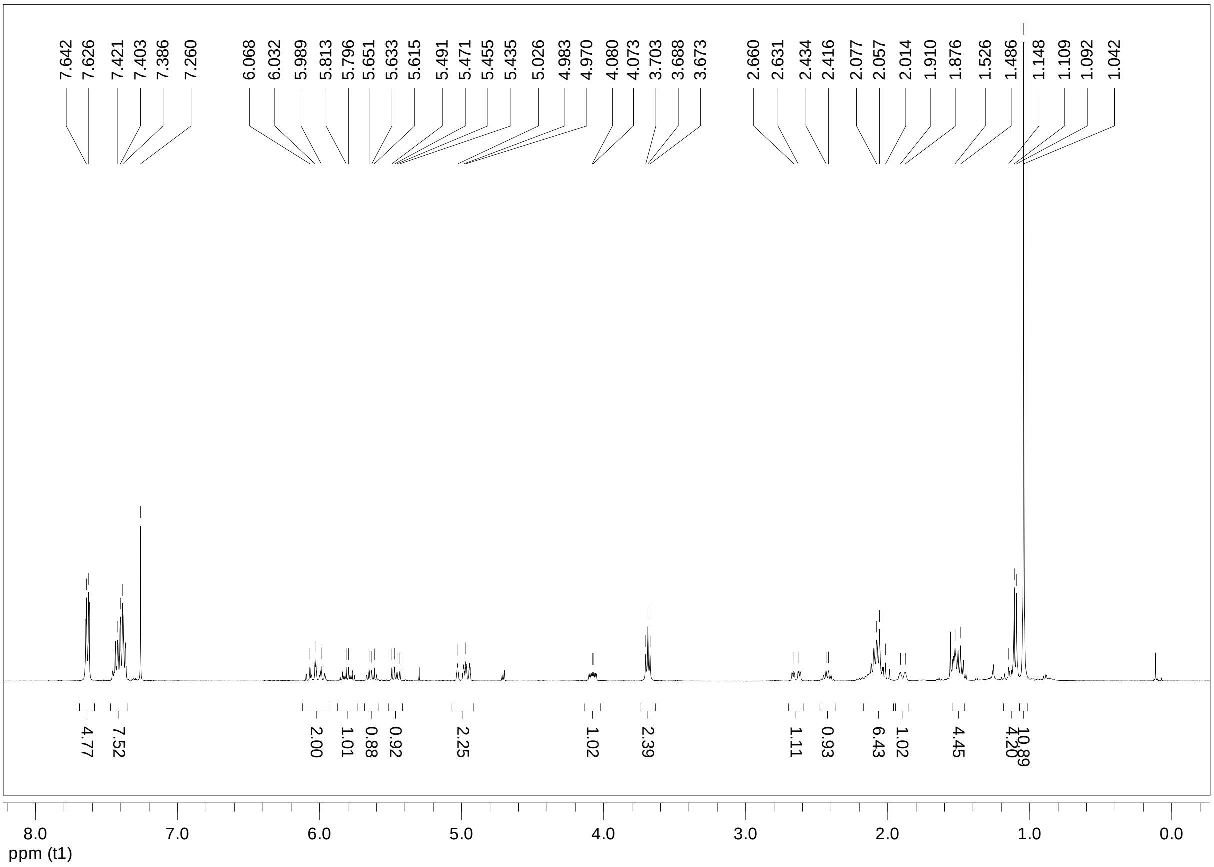
7.642
7.626
7.421
7.403
7.386
7.260
6.068
6.032
5.989
5.813
5.796
5.651
5.633
5.615
5.491
5.471
5.455
5.435
5.026
4.983
4.970
4.080
4.073
3.703
3.688
3.673
2.660
2.631
2.434
2.416
2.077
2.057
2.014
1.910
1.876
1.526
1.486
1.148
1.109
1.092
1.042
4.77
7.52
2.00
1.01
0.88
0.92
2.25
1.02
2.39
1.11
0.93
6.43
1.02
4.45
4.20
10.89
8.0
7.0
6.0
5.0
4.0
3.0
2.0
1.0
0.0
ppm (t1)

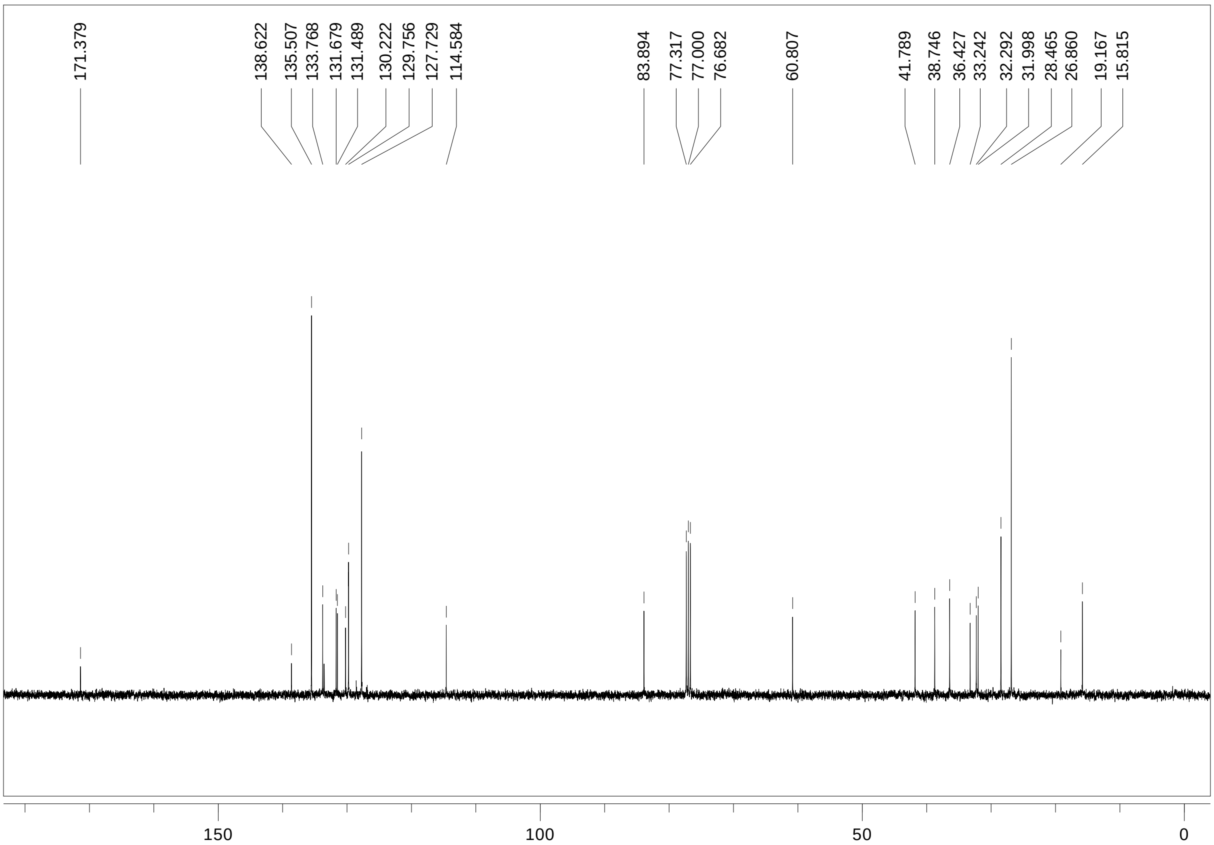
171.379
138.622
135.507
133.768
131.679
131.489
130.222
129.756
127.729
114.584
83.894
77.317
77.000
76.682
60.807
41.789
38.746
36.427
33.242
32.292
31.998
28.465
26.860
19.167
15.815
150
100
50
0
ppm (t1)

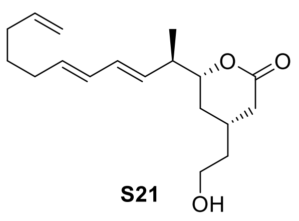

S21

(4*R*,6*R*)-4-(2-Hydroxyethyl)-6-((*R*,3*E*,5*E*)-undeca-3,5,10-trien-2-yl)tetrahydro-2H-pyran-2-one (S21): To a solution of **S20** (144 mg, 0.27 mmol, 1.0 equiv) in THF (2.70 mL) was added at 0 °C TBAF (1.10 mL, 1.10 mmol, 4.0 equiv, 1.0 M in THF) and AcOH (63 µL, 1.1 mmol, 4.0 equiv). After stirring for 16 h at rt, the reaction mixture was quenched with sat. NH_4Cl (15 mL). The layers were separated and the aqueous layer was extracted with EtOAc (3 x 20 mL). The combined organic extracts were washed with brine, dried over $MgSO_4$ and the solvent was removed under reduced pressure. The residue was purified by column chromatography (hexane/EtOAc, 1:2 → 0:1) to give **S21** (72.4 mg, 89%, E/Z >30:1, 3% wt/wt CH_2Cl_2) as a colorless oil.

R_f = 0.52 (EtOAc).

$[\alpha]_D^{24}$ = +10.7° [*c* = 0.635, $CHCl_3$].

^{1}H-NMR (400.1 MHz, $CDCl_3$): δ = 6.13-5.91 (m, 2H), 5.80 (tdd, *J* = 6.7, 10.2, 16.9 Hz, 1H), 5.63 (td, *J* = 6.8, 13.9 Hz, 1H), 5.47 (dd, *J* = 8.0, 14.6 Hz, 1H), 5.00 (ddd, *J* = 1.6, 3.6, 17.2 Hz, 1H), 4.95 (tdd, *J* = 1.2, 2.2, 10.2 Hz, 1H), 4.12 (ddd, *J* = 2.9, 6.3, 11.8 Hz, 1H), 3.70 (t, *J* = 6.3 Hz, 2H), 2.78-2.65 (m, 1H), 2.45 (m_c, 1H), 2.20-2.01 (m, 6H), 1.96 (m_c, 1H), 1.66-1.42 (m, 5H), 1.28-1.10 (m, 1H), 1.11 (d, *J* = 6.8 Hz, 3H).

^{13}C-NMR (100.6 MHz, $CDCl_3$): δ = 171.3, 138.6, 133.9, 131.8, 131.4, 130.2, 114.6, 83.8, 59.6, 41.8, 38.8, 36.3, 33.2, 32.3, 32.0, 28.4, 28.2, 15.8.

IR (neat, ν/cm^{-1}): 3427brs, 2925m, 1723s, 1456w, 1440w, 1381m, 1247s, 1087m, 1053m, 990s, 911m.

HRMS (ESI): *m/z* calcd for $C_{18}H_{28}NaO_3$ [M + Na]$^+$: 315.1931, found: 315.1923.

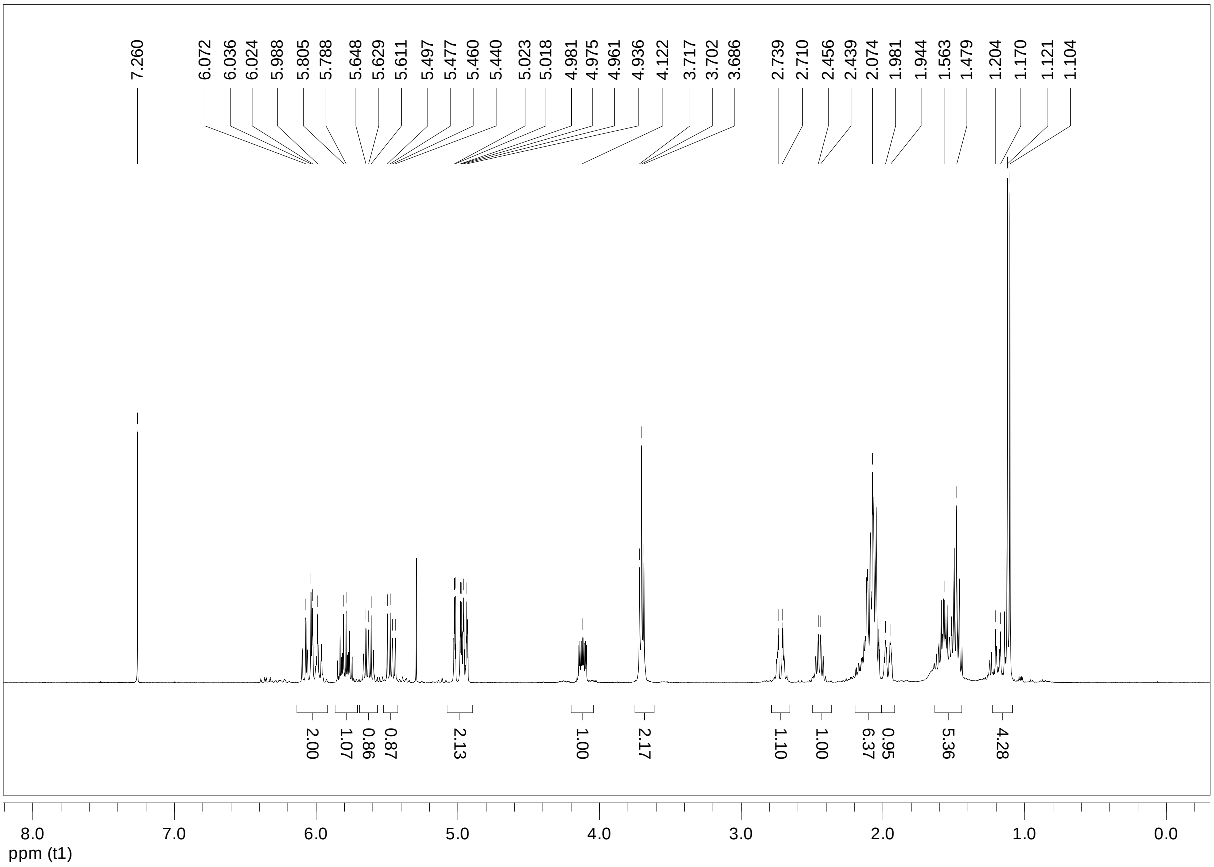
7.260
6.072
6.036
6.024
5.988
5.805
5.788
5.648
5.629
5.611
5.497
5.477
5.460
5.440
5.023
5.018
4.981
4.975
4.961
4.936
4.122
3.717
3.702
3.686
2.739
2.710
2.456
2.439
2.074
1.981
1.944
1.563
1.479
1.204
1.170
1.121
1.104
2.00
1.07
0.86
0.87
2.13
1.00
2.17
1.10
1.00
6.37
0.95
5.36
4.28
8.0
7.0
6.0
5.0
4.0
3.0
2.0
1.0
0.0
ppm (t1)

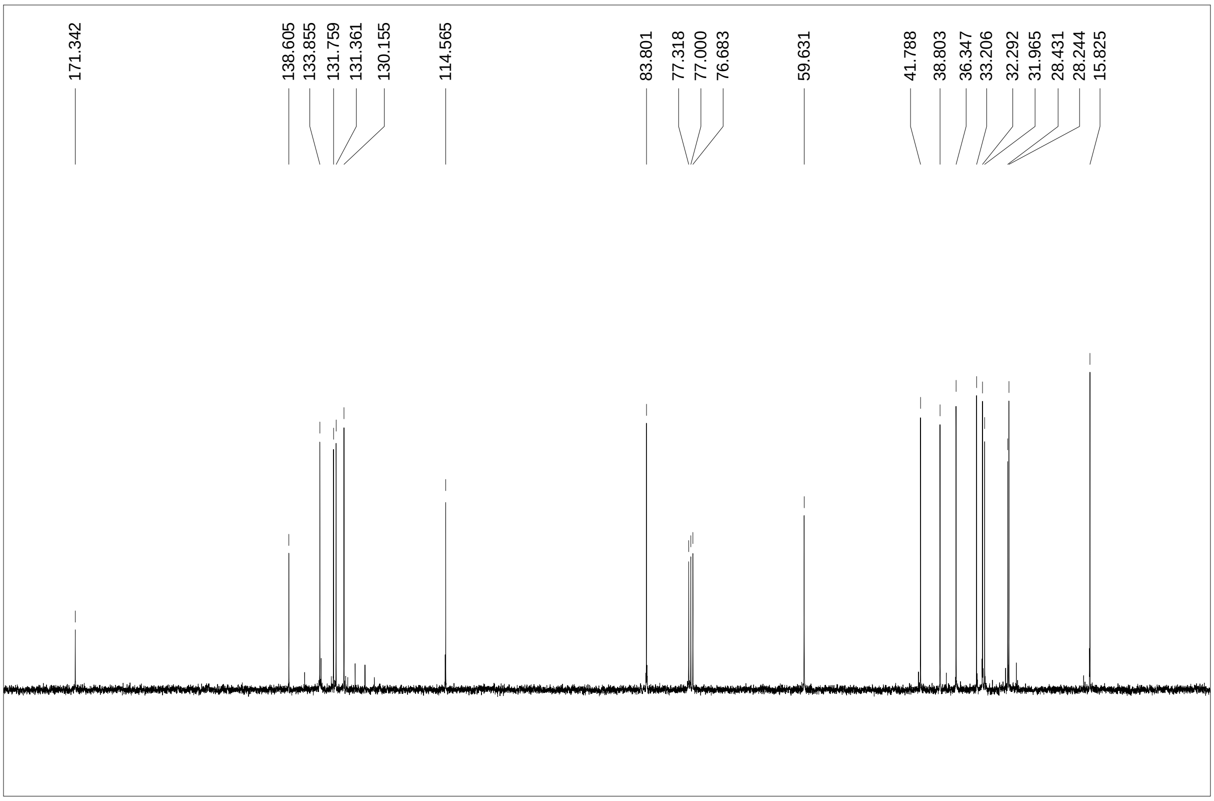
171.342
138.605
133.855
131.759
131.361
130.155
114.565
83.801
77.318
77.000
76.683
59.631
41.788
38.803
36.347
33.206
32.292
31.965
28.431
28.244
15.825
150
100
50
0
ppm (t1)

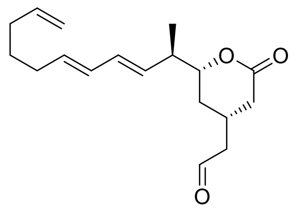

83

2-((4*S*,6*R*)-2-oxo-6-((*R*,3*E*,5*E*)-undeca-3,5,10-trien-2-yl)tetrahydro-2H-pyran-4-yl)acetaldehyde (83): To a solution of oxalyl chloride (30.6 µL, 0.36 mmol, 1.5 equiv) in CH_2Cl_2 (3.0 mL) at –78 °C was added dropwise DMSO (51.3 µL, 0.72 mmol, 3.0 equiv). After stirring at –78 °C for 10 min, a solution of **S21** (70.4 mg, 0.24 mmol, 1.0 equiv) in CH_2Cl_2 (2.1 mL) was added dropwise. The resultant cloudy mixture was stirred at –78 °C for 1.5 h, and then TEA (0.13 mL, 0.96 mmol, 4.0 equiv) was added slowly and the reaction mixture was allowed to warm to room temperature (1 h). The reaction was quenched with water (10 mL), and the layers were separated. The aqueous phase was extracted with CH_2Cl_2 (3 x 15 mL) and the combined organic phases were washed with brine, dried over $MgSO_4$, filtered and concentrated *in vacuo*. The crude product was purified by column chromatography (hexane/EtOAc, 3:1 → 1:1) to afford **83** (66.6 mg, 95%) as a yellow oil.

$\mathbf{R}_f$ = 0.40 (hexane/EtOAc, 1:1).
$[\alpha]_D^{24}$ = +7.4° [*c* = 0.600, $CHCl_3$].
^{1}**H-NMR** (400.1 MHz, $CDCl_3$): δ = 9.75 (s, 1H), 6.12-5.90 (m, 2H), 5.79 (dt, *J* = 6.6, 16.9 Hz, 1H), 5.62 (td, *J* = 6.9, 14.2 Hz, 1H), 5.44 (dd, *J* = 8.0, 14.5 Hz, 1H), 5.00 (d, *J* = 17.2 Hz, 1H), 4.94 (d, *J* = 9.8 Hz, 1H), 4.15 (ddd, *J* = 2.6, 6.3, 11.3 Hz, 1H), 2.78 (m_c, 1H), 2.58-2.38 (m, 4H), 2.20-1.93 (m, 6H), 1.47 (m_c, 2H), 1.29-1.14 (m, 1H), 1.11 (d, *J* = 6.9 Hz, 3H).
^{13}**C-NMR** (100.6 MHz, $CDCl_3$): δ = 199.6, 170.3, 138.6, 134.0, 131.9, 131.0, 130.1, 114.6, 83.4, 49.8, 41.7, 35.8, 33.2, 31.9 (2C), 28.4, 25.9, 15.8.
IR (neat, ν/cm^{-1}): 2974m, 2926m, 2855w, 2728w, 1732s, 1640w, 1456m, 1383m, 1238s, 1173w, 1078m, 991s, 912m.
HRMS (ESI): *m/z* calcd for $C_{18}H_{26}NaO_3$ [M + Na]$^+$: 313.1774, found: 313.1783.

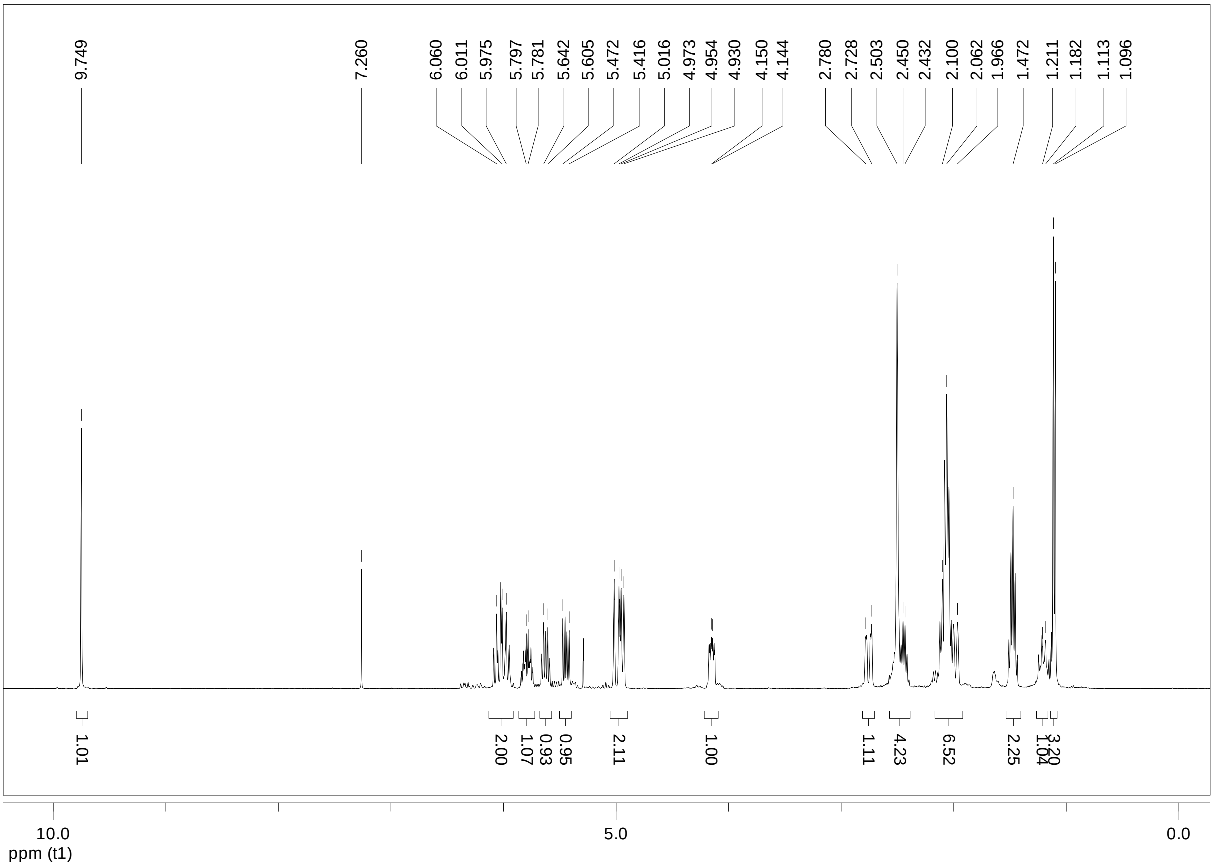
9.749
7.260
6.060
6.011
5.975
5.797
5.781
5.642
5.605
5.472
5.416
5.016
4.973
4.954
4.930
4.150
4.144
2.780
2.728
2.503
2.450
2.432
2.100
2.062
1.966
1.472
1.211
1.182
1.113
1.096
1.01
2.00
1.07
0.93
0.95
2.11
1.00
1.11
4.23
6.52
2.25
1.04
3.20
10.0
5.0
0.0
ppm (t1)

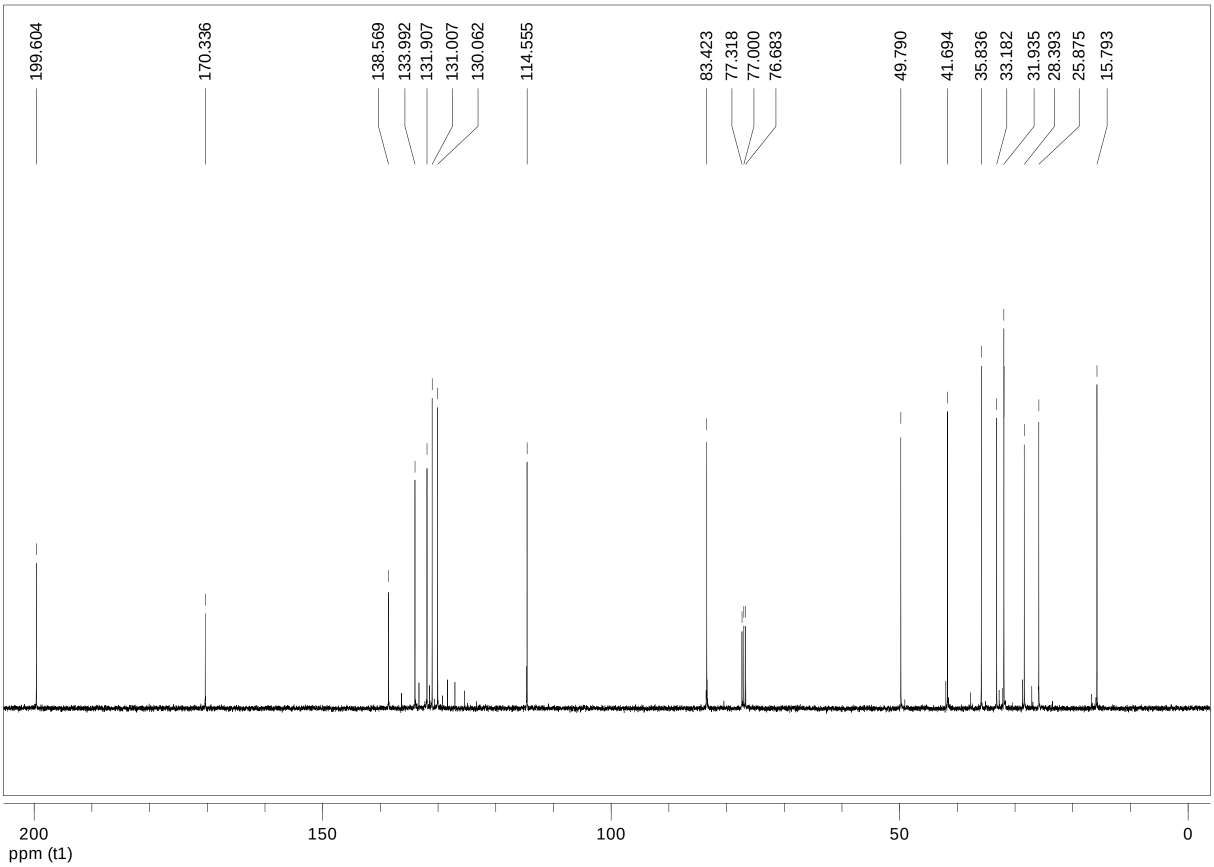
199.604
170.336
138.569
133.992
131.907
131.007
130.062
114.555
83.423
77.318
77.000
76.683
49.790
41.694
35.836
33.182
31.935
28.393
25.875
15.793
200
150
100
50
0
ppm (t1)

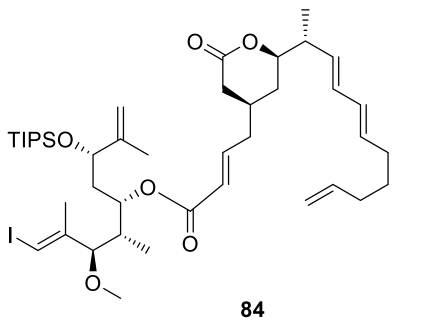

84

(3*R*,4*S*,5*S*,7*S*,*E*)-1-Iodo-3-methoxy-2,4,8-trimethyl-7-((triisopropylsilyl)oxy)nona-1,8-dien-5-yl (*E*)-4-((4*R*,6*R*)-2-oxo-6-((*R*,3*E*,5*E*)-undeca-3,5,10-trien-2-yl)tetra-hydro-2H-pyran-4-yl)but-2-enoate (83): To LiCl (8.9 mg, 211 µmol, 1.15 eq) was added a solution of the phosphonate **80** (145 mg, 211 µmol, 1.15 eq) in MeCN (0.3 mL, rinsed with 2×0.3 mL+0.5 mL), followed by DBU (30.0 µL, 202 µmol, 1.10 eq). After 15 min stirring at rt, the colourless solution was cooled to 0 °C, whereupon the solution turned slightly turbid. After 25 min, a solution of the aldehyde **83** (53.3 mg, 184 µmol, 1.00 eq) in MeCN (1.6 mL) was added. The pale yellow mixture was stirred for 30 min at 0 °C and was then allowed to warm to rt. After 1 h, the mixture was concentrated at the rotavap and the residue was purified by flash chromatography (hex:EtOAc 3:1) to afford the desired product **84** (108 mg 97% wt/wt along with ether and EtOAc, 105 mg, 69%) as a colourless oil.

TLC (hex:EtOAc 3:1): R_f = 0.43

^{1}H-NMR ($CDCl_3$, 400.1 MHz): δ = 6.78 (m_c, 1H), 6.15 (bs, 1H), 6.11-5.94 (m, 2H), 5.87-5.74 (m, 2H),5.63 (td, *J* = 7.0 Hz, *J* = 14.1 Hz, 1H), 5.46 (dd, *J* = 8.0 Hz, *J* = 14.8 Hz, 1H), 5.00 (ddd, *J* = 1.6 Hz, *J* = 3.6 Hz, *J* = 17.1 Hz, 1H), 4.95 (tdd, *J* = 1.2 Hz, *J* = 2.2 Hz, *J* = 10.2 Hz, 1H), 4.78 (m_c, 1H), 4.73 (ddd, *J* = 1.5 Hz, *J* = 4.0 Hz, *J* = 9.9 Hz, 1H), 4.65 (m_c, 1H), 4.18-4.06 (m, 2H), 3.37 (d, *J* = 2.3 Hz, 1H), 3.16 (s, 3H), 2.68 (m, 1H), 2.46 (dd, *J* = 6.9 Hz, *J* = 13.8 Hz, 1H), 2.26-2.16 (m, 2H), 2.14-2.01 (m, 7H), 1.98-1.90 (m, 1H), 1.89-1.78 (m, 1H), 1.83 (d, *J* = 1.0 Hz, 3H), 1.76-1.63 (m, 1H), 1.67 (bs, 3H), 1.48 (m_c, 2H), 1.24-1.16 (m, 1H), 1.11 (d, *J* = 6.8 Hz, 3H), 1.07-0.09 (m, 21H), 0.93 (d, *J* = 6.9 Hz, 3H).

^{13}C-NMR ($CDCl_3$, 100.6 MHz): δ = 170.6, 165.3, 147.0, 146.6, 144.2, 138.6, 134.1, 132.0, 131.0, 130.1, 124.1, 114.6, 111.7, 87.7, 83.5, 79.0, 75.5, 72.5, 56.7, 41.7, 39.0, 38.7, 35.9, 35.8, 33.2, 32.0, 31.9, 31.0, 28.4, 19.6, 18.2, 18.1, 16.5, 15.8, 12.4, 9.9.

HRMS Calcd. for $C_{42}H_{69}INaO_6Si$ $[M+Na]^+$ *m/z* 847.3800. Found: 847.3808.

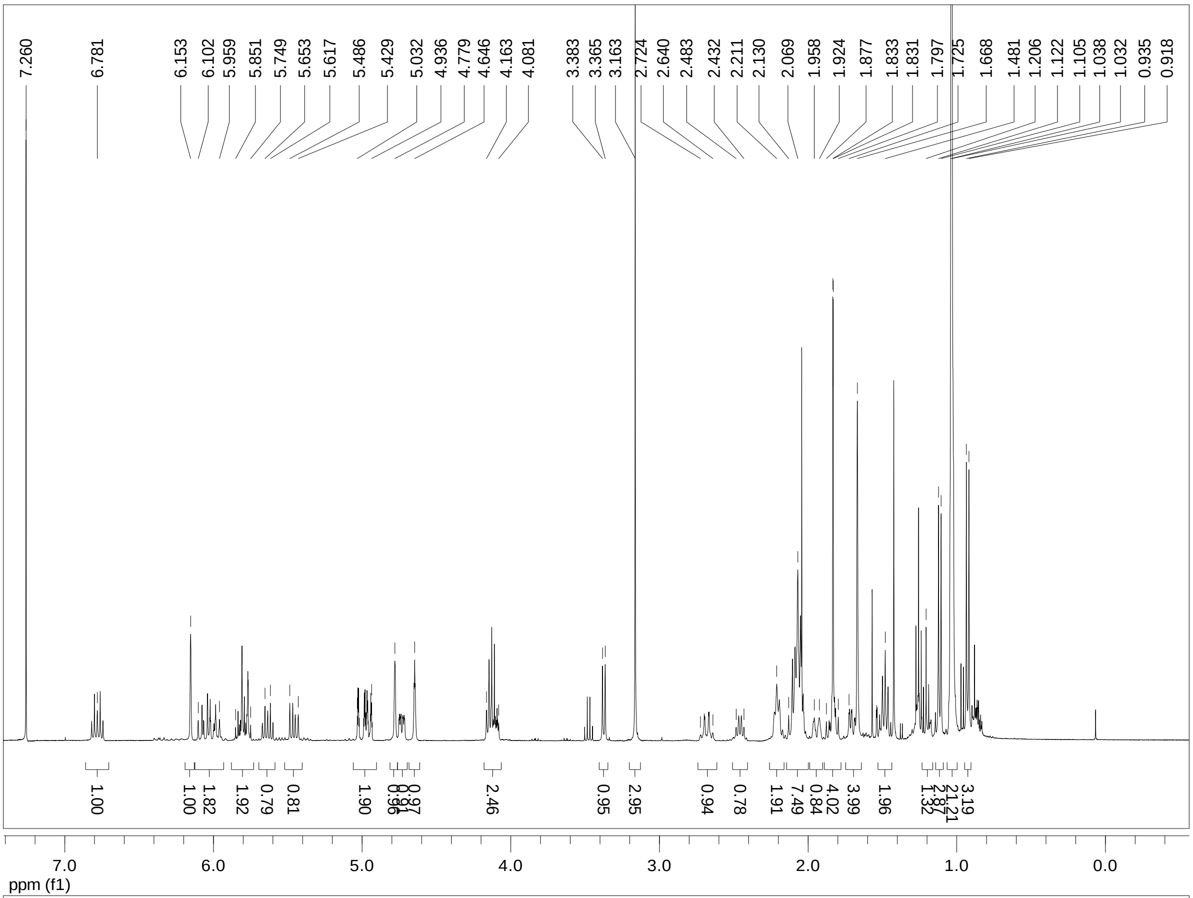

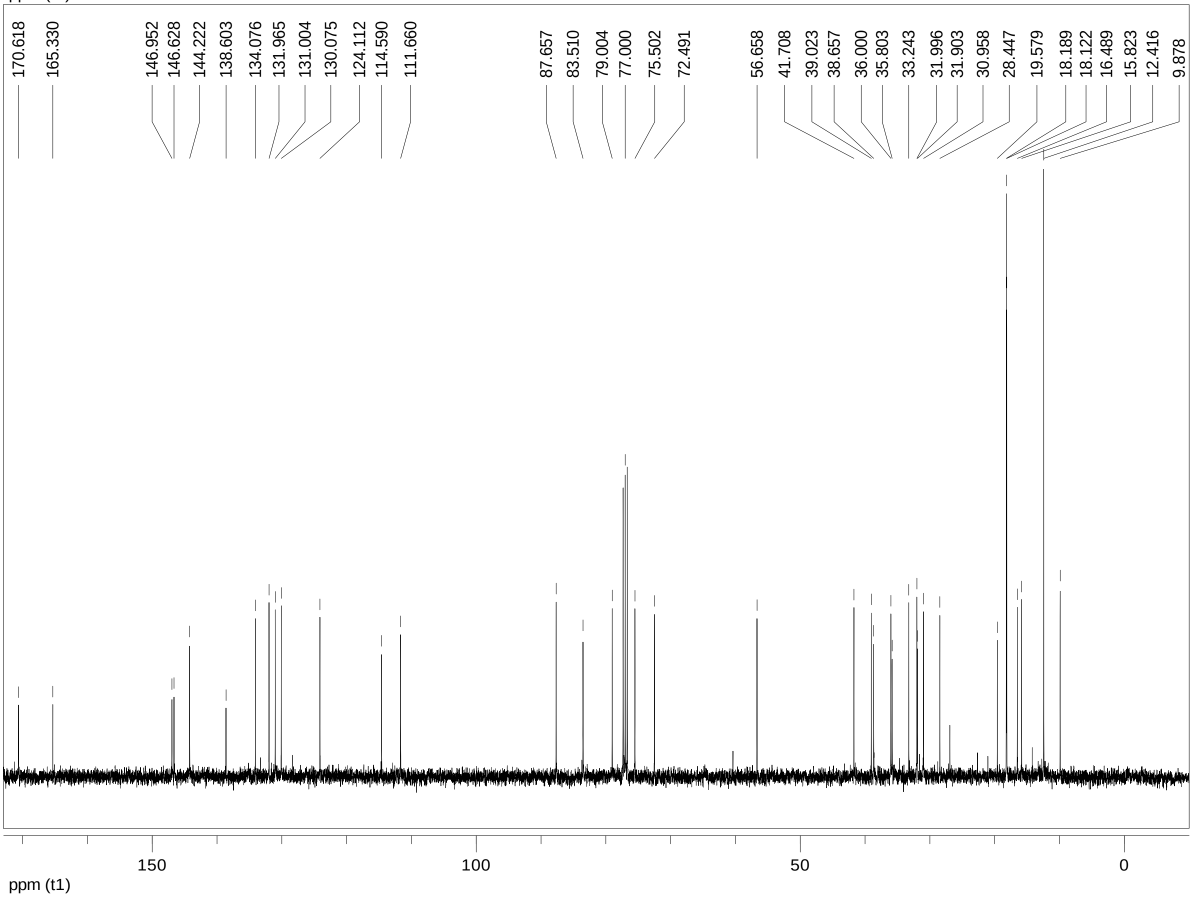

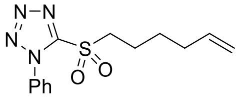

86

5-(Hex-5-en-1-ylsulfonyl)-1-phenyl-1H-tetrazole: To a solution of 5-hexen-1-ol (**85**) (135 µL, 1.18 mmol, 1.00 eq), phenyltetrazole thiol (240 mg, 1.35 mmol, 1.15 eq) and PPh_3 (353 mg, 1.35 mmol, 1.15 eq) in THF (6 mL) was added DEAD (212 µL, 1.35 mmol, 1.15 eq) dropwise at rt. The orange colour of the drops discharged upon contact with the mixture until the very last drops and a slight warming was observed. The resulting pale yellow solution was stirred for 3 h. Then EtOH (10 mL) was added and the reaction was cooled to 0 °C. Afterwards a previously prepared solution of ammonium molybdate tetrahydrate (229 mg, 0.185 mmol, 16 mol%) in aqueous H_2O_2 (30%, 1.86 mL) was added *via* pipette. The cooling was removed after a few minutes and the mixture was stirred at rt overnight. Then it was diluted with water (40 mL) and dichloromethane. The layers were separated and the aqueous phase was extracted with CH_2Cl_2 (3×20 mL). The combined organic extracts were washed with brine and concentrated under reduced pressure. The dark orange crude was purified by column chromatography (hex:EtOAc 5:1) to afford the title compound **86** (269 mg, 78%) as a pale yellow oil.

TLC (hex:EtOAc 5:1): R_f = 0.30

^{1}H-NMR ($CDCl_3$, 400.1 MHz): δ = 7.72-7.67 (m, 2H), 7.65-7.57 (m, 3H), 5.77 (tdd, *J* = 6.7 Hz, *J* = 10.2 Hz, *J* = 16.9 Hz, 1H), 5.09-4.96 (m, 2H), 3.75 (m_c, 2H), 2.13 (m_c, 2H), 1.98 (m_c, 2H), 1.61 (m_c, 2H).

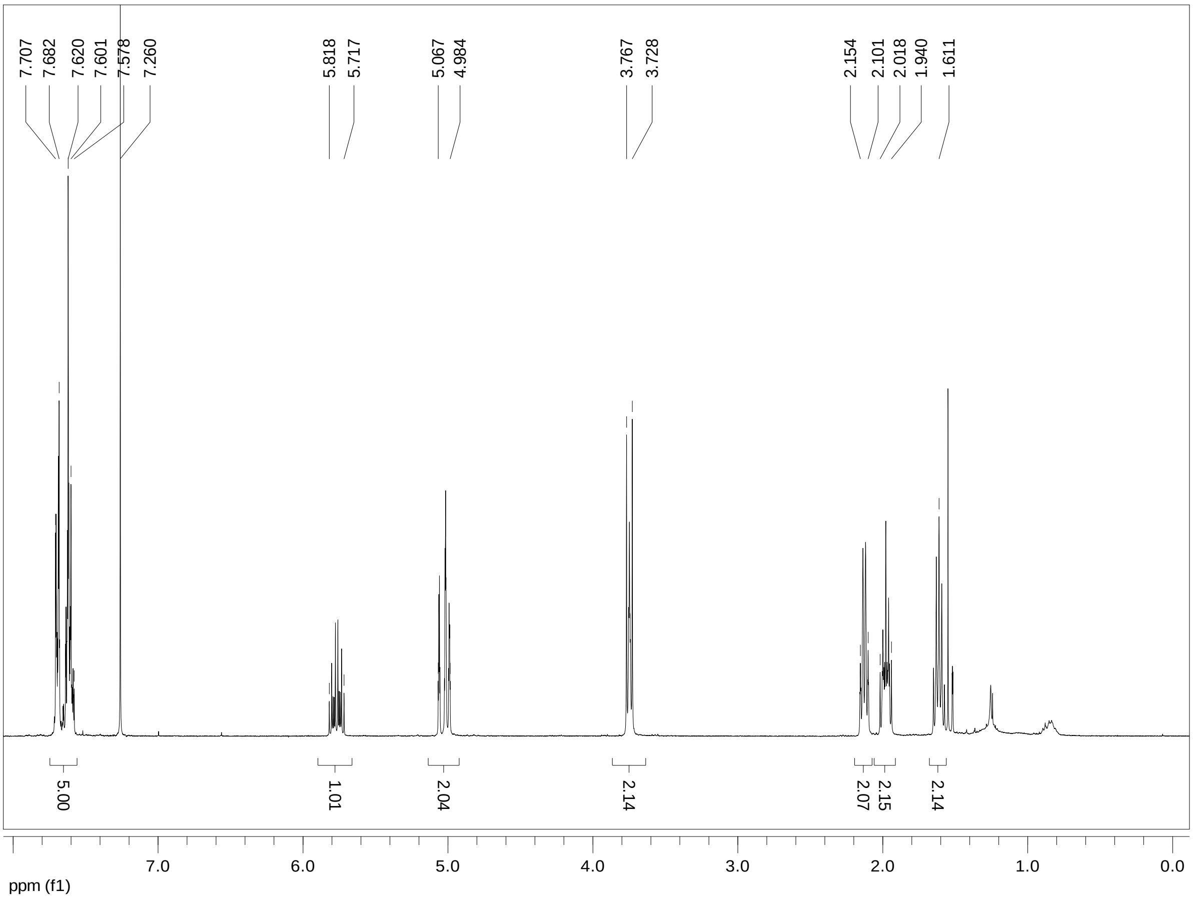
7.707
7.682
7.620
7.601
7.578
7.260
5.818
5.717
5.067
4.984
3.767
3.728
2.154
2.101
2.018
1.940
1.611
5.00
1.01
2.04
2.14
2.07
2.15
2.14
7.0
6.0
5.0
4.0
3.0
2.0
1.0
0.0
ppm (f1)

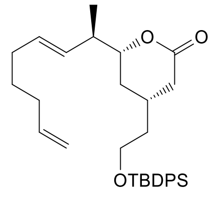

S22

(4*R*,6*R*)-4-(2-((*tert*-Butyldiphenylsilyl)oxy)ethyl)-6-((*R*,*E*)-nona-3,8-dien-2-yl)tetrahydro-2H-pyran-2-one (*S22*): To a solution of the sulfone **86** (173 mg, 0.590 mmol, 1.30 eq) in THF (4.30 mL) was added solid NaHMDS (108 mg, 0.590 mmol, 1.30 eq) at −78 °C, whereupon the colour turned bright yellow. After 10 min stirring at −78 °C, the cooling was removed, allowing for the mixture to warm to −9 °C. After another 5 min, the mixture was re-cooled to −78 °C and after a total of 20 min a solution of the aldehyde **66** (217 mg crude) in THF (0.3 mL, rinsed with 2×0.2 mL) was added. The reaction was aged 1 h at −78 °C and was then warmed to −50 °C (MeCN/dry ice). After 45 min stirring at −50 °C, the mixture was allowed to warm to −19 °C (ice/NaCl), whereupon the yellow colour faded slightly. After 30 min stirring at −19 °C the mixture was allowed to warm to 0 °C. Only at 0 °C a white precipitate formed. After 1.5 h at 0 °C, pH7 buffer solution was added and the layers were separated. The aqueous layer was extracted with Et_2O (3x10 mL). The combined organic extracts were washed with brine, dried over $MgSO_4$, filtered and concentrated under reduced pressure. The crude was purified by column chromatography (hexane/EtOAc, 5:1) to afford the desired compound **S22** (185 mg, 78% wt/wt along with sulfone, 143 mg, 62% over 2 steps) as a colourless oil.

^{1}H-NMR ($CDCl_3$, 400.1 MHz): δ = 7.67-7.57 (m, 4H), 7.47-7.35 (m, 6H), 5.87-5.70 (m, 1H), 5.54-5.39 (m, 1H), 5.32 (tdd, *J* = 1.2 Hz, *J* = 8.0 Hz, *J* = 15.4 Hz, 1H), 4.99 (ddd, *J* = 1.6 Hz, *J* = 3.7 Hz, *J* = 17.3 Hz, 1H), 4.95 (tdd, *J* = 1.2 Hz, *J* = 2.2 Hz, *J* = 10.2 Hz, 1H), 4.05 (ddd, *J* = 2.9 Hz, *J* = 6.3 Hz, *J* = 11.8 Hz, 1H), 3.69 (m_c, 2H), 2.64 (ddd, *J* = 1.8 Hz, *J* = 5.5 Hz, *J* = 17.1 Hz, 1H), 2.35 (m_c, 1H), 2.20-1.93 (m, 6H), 1.89 (m_c, 1H), 1.61 (td, *J* = 7.5 Hz, *J* = 14.9 Hz, 1H), 1.57-1.50 (m, 2H), 1.49-1.40 (m, 2H), 1.19-1.09 (m, 1H), 1.07 (d, *J* = 6.8 Hz, 3H), 1.05 (s, 9H).

^{13}C-NMR ($CDCl_3$, 100.6 MHz): δ = 171.5, 138.7, 135.5, 133.58, 133.54, 131.9, 130.6, 129.8, 127.7, 114.6, 84.0, 60.8, 41.9, 38.8, 36.4, 33.2, 32.3, 32.0, 28.6, 28.4, 26.9 19.2, 16.1.

HRMS Calcd. for $C_{32}H_{44}NaO_3Si$ $[M+Na]^+$ *m/z* 527.2952. Found: 527.2952.

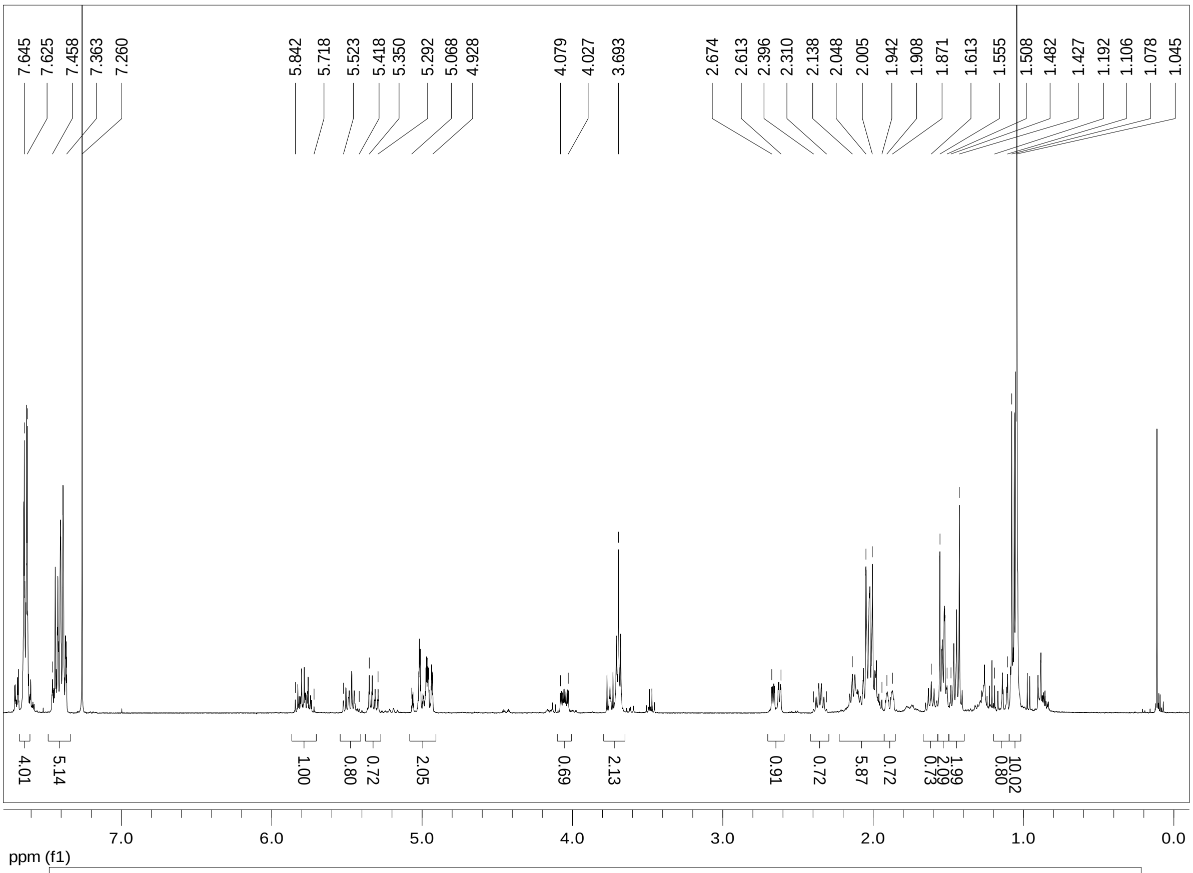

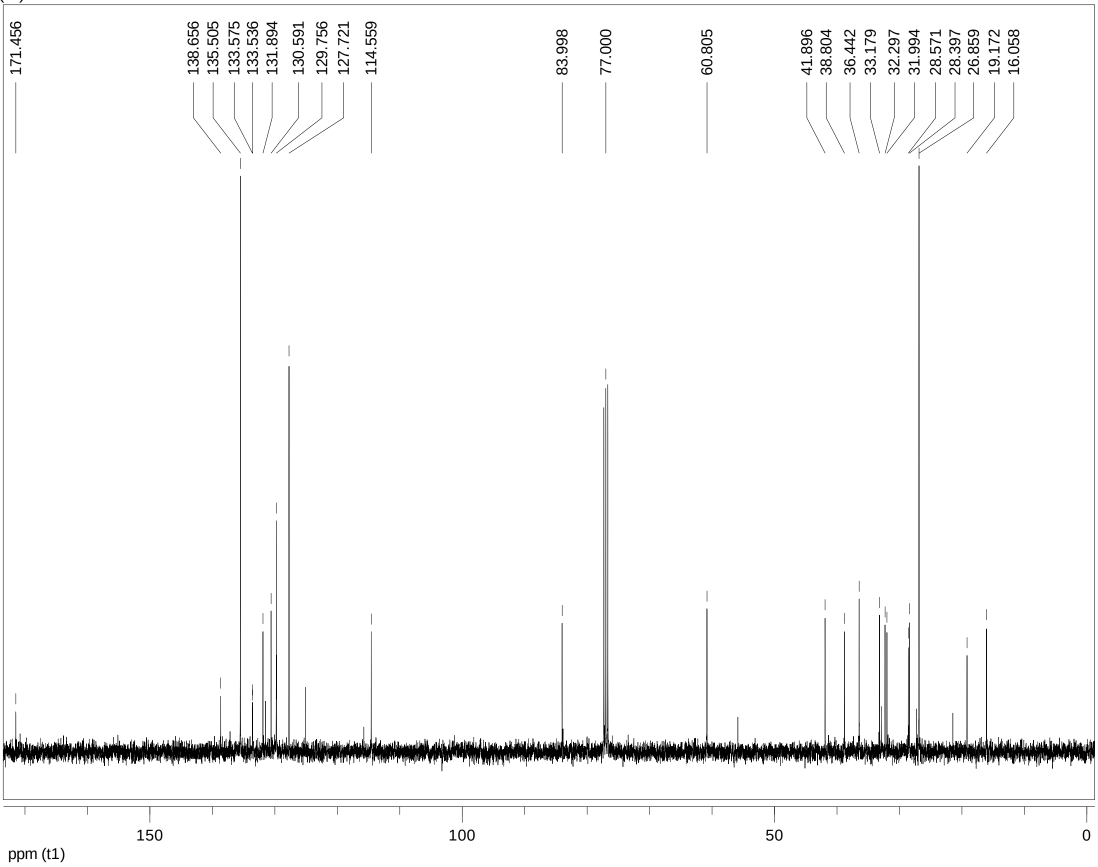

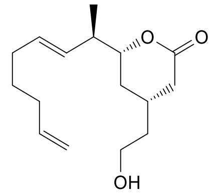

S23

(4*R*,6*R*)-4-(2-Hydroxyethyl)-6-((*R*,*E*)-nona-3,8-dien-2-yl)tetrahydro-2H-pyran-2-one (S23): To a cooled (0 °C) solution of the silyl ether **S22** (143 mg, 283 µmol, 1.00 eq) in THF (0.6 mL) were added AcOH (12.2 µL, 0.217 mmol, 4.00 eq) and TBAF (0.217 mL 1.0 M in THF, 0.217 mmol, 4.0 equiv). The cooling was removed after 5-10 min and the mixture was stirred at rt for 19 h. Then, the reaction mixture was quenched with sat. NH_4Cl and the layers were separated. The aqueous phase was extracted with EtOAc (3 x 10 mL). The combined organic extracts were washed with brine, dried over $MgSO_4$ and the solvent was removed under reduced pressure. The residue was purified by column chromatography (hex/EtOAc, 1:1 → 0:1) to give the primary alcohol **S23** as a colorless oil (74.2 mg, 95% wt/wt along with EtOAc, 70.5 mg, 94%).

TLC (EtOAc): R_f = 0.43
^{1}H-NMR ($CDCl_3$, 400.1 MHz): δ = 5.80 (tdd, *J* = 6.7 Hz, *J* = 10.2 Hz, *J* = 16.9 Hz, 1H), 5.50 (dtd, *J* = 0.6 Hz, *J* = 6.6 Hz, *J* = 15.2 Hz, 1H), 5.33 (tdd, *J* = 1.3 Hz, *J* = 8.0 Hz, *J* = 15.4 Hz, 1H), 5.00 (ddd, *J* = 1.7 Hz, *J* = 3.7 Hz, *J* = 17.3 Hz, 1H), 4.95 (tdd, *J* = 1.2 Hz, *J* = 2.2 Hz, *J* = 10.2 Hz, 1H), 4.09 (ddd, *J* = 2.9 Hz, *J* = 6.5 Hz, *J* = 11.8 Hz, 1H), 3.71 (t, *J* = 6.3 Hz, 2H), 2.73 (m_c, 1H), 2.38 (m_c, 1H), 2.19-1.93 (m, 7H), 1.58 (m_c, 2H), 1.45 (m_c, 2H), 1.24-1.11 (m, 1H), 1.09 (d, *J* = 6.8 Hz, 3H).
^{13}C-NMR ($CDCl_3$, 100.6 MHz): δ = 171.4, 138.7, 132.0, 130.6, 114.6, 83.9, 59.7, 42.0, 38.8, 36.4, 33.2, 32.4, 32.0, 28.6, 28.3, 16.1.
HRMS Calcd. for $C_{16}H_{27}O_3$ $[M+H]^+$ *m/z* 267.1955. Found: 267.1954.

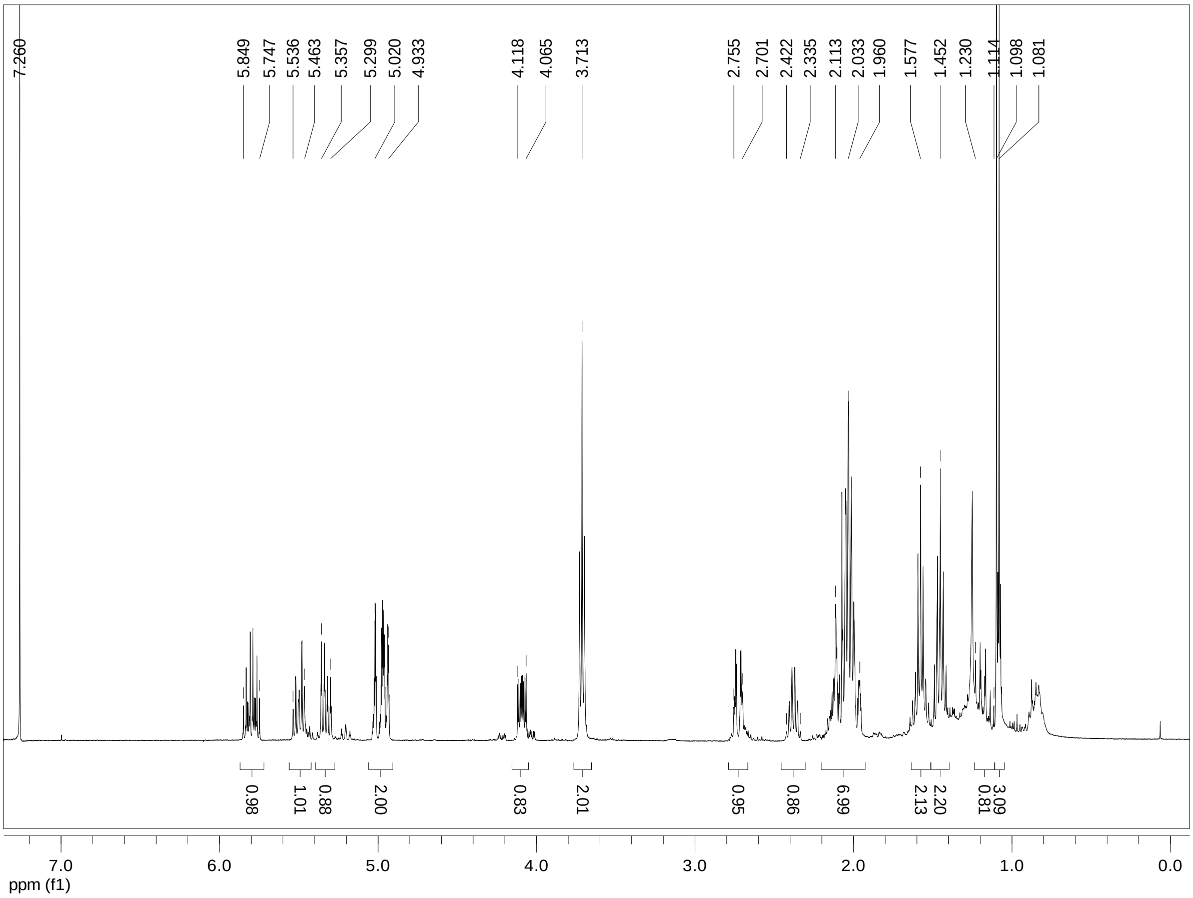

7.260
5.849
5.747
5.536
5.463
5.357
5.299
5.020
4.933
4.118
4.065
3.713
2.755
2.701
2.422
2.335
2.113
2.033
1.960
1.577
1.452
1.230
1.114
1.098
1.081
0.98
1.01
0.88
2.00
0.83
2.01
0.95
0.86
6.99
2.13
2.20
0.81
3.09
7.0
6.0
5.0
4.0
3.0
2.0
1.0
0.0
ppm (f1)

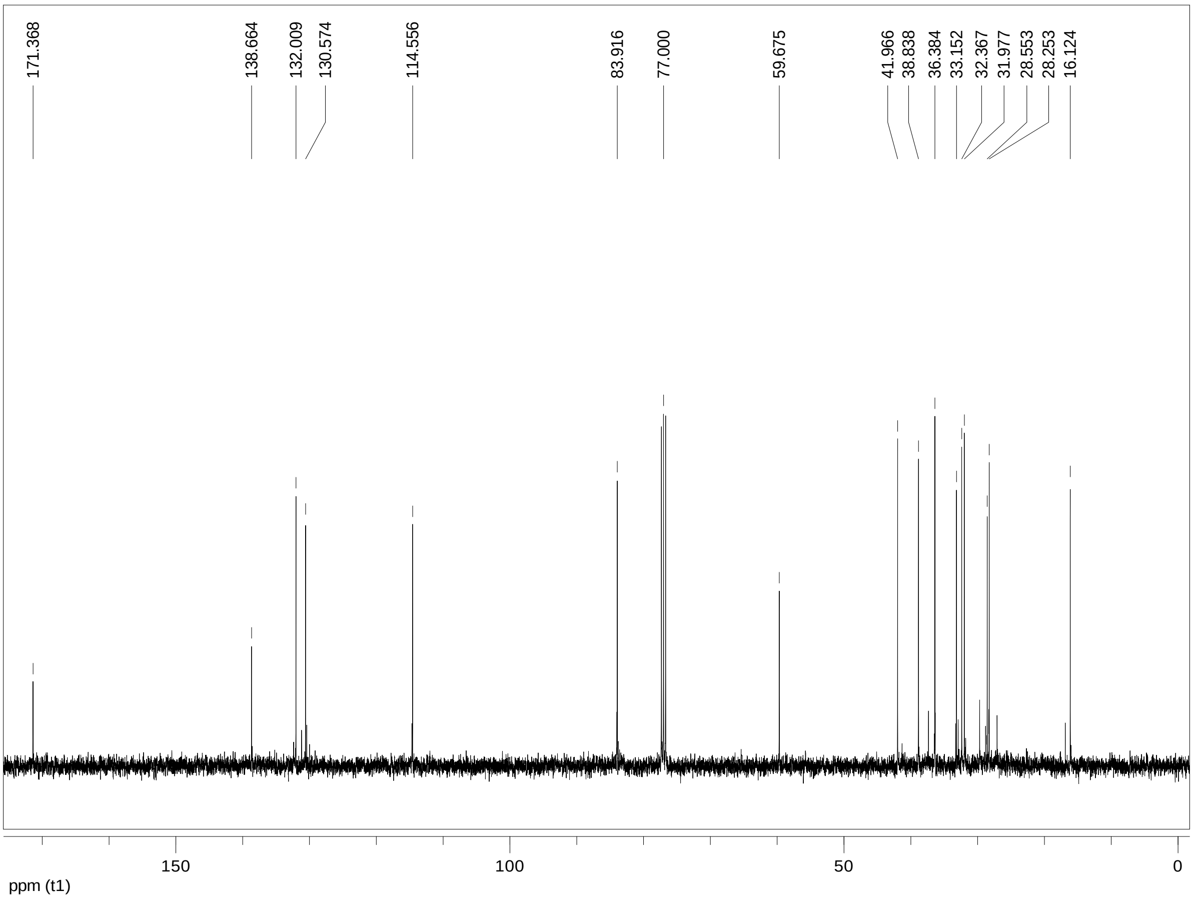

171.368
138.664
132.009
130.574
114.556
83.916
77.000
59.675
41.966
38.838
36.384
33.152
32.367
31.977
28.553
28.253
16.124
150
100
50
0
ppm (t1)

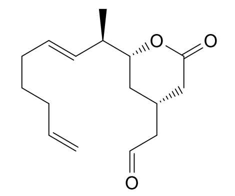

87

2-((2*R*,4*S*)-2-((*R*,*E*)-Nona-3,8-dien-2-yl)-6-oxotetrahydro-2H-pyran-4-yl)acetaldehyde (87): To a solution of oxalyl chloride (34.8 µL, 0.414 mmol, 1.55 eq) in CH_2Cl_2 (3.0 mL) was added a solution of DMSO (58.8 µL, 0.827 mmol, 3.10 eq) dropwise at –78 °C. After stirring at –78 °C for 10 min, a solution of the primary alcohol **S23** (71.1 mg, 0.267 mmol, 1.00 equiv) in CH_2Cl_2 (1.9 mL + 1.9 mL + 1.1 mL) was added dropwise. The resulting cloudy mixture was stirred at –78 °C for 1 h, and then NEt_3 (149 µL, 1.07 mmol, 4.00 eq) was added slowly and the reaction mixture was allowed to warm to rt. After another 40 min stirring, the reaction was quenched with water (5 mL). The layers were separated, the aqueous phase was extracted with CH_2Cl_2 (3 x 10 mL) and the combined organic extracts were washed with brine, dried over $MgSO_4$ and concentrated *in vacuo*. The crude was purified by column chromatography (hex/EtOAc, 2:1→1:1) to afford the desired aldehyde **87** (61.6 mg, 92%, *E:Z* = 9.6:1) as a colorless oil.

TLC (hex:EtOAc 1:1): R_f = 0.40

^{1}H-NMR ($CDCl_3$, 400.1 MHz): δ = 9.76 (bs, 1H), 5.79 (tdd, *J* = 6.7 Hz, *J* = 10.2 Hz, *J* = 16.9 Hz, 1H), 5.50 (dtd, *J* = 0.7 Hz, *J* = 6.7 Hz, *J* = 15.4 Hz, 1H), 5.31 (tdd, *J* = 1.3 Hz, *J* = 8.0 Hz, *J* = 15.4 Hz, 1H), 4.99 (ddd, *J* = 1.6 Hz, *J* = 3.6 Hz, *J* = 17.1 Hz, 1H), 4.95 (tdd, *J* = 1.2 Hz, *J* = 2.2 Hz, *J* = 10.2 Hz, 1H), 4.13 (ddd, *J* = 2.9 Hz, *J* = 6.5 Hz, *J* = 11.7 Hz, 1H), 2.76 (m_c, 1H), 2.59-2.43 (m, 3H), 2.37 (m_c, 1H), 2.15-1.96 (m, 6H), 1.45 (m_c, 2H), 1.28-1.12 (m, 1H), 1.08 (d, *J* = 6.8 Hz, 3H).

^{13}C{^{1}H}-NMR ($CDCl_3$, 100.6 MHz): δ = 199.6, 170.5, 138.6, 132.2, 130.3, 114.6, 83.6, 49.9, 41.9, 35.9, 33.1, 32.0, 32.01, 31.96, 28.5, 25.9, 16.1.

HRMS Calcd. for $C_{16}H_{28}NO_3$ $[M+NH_4]^+$ *m/z* 268.2064. Found: 268.2077.

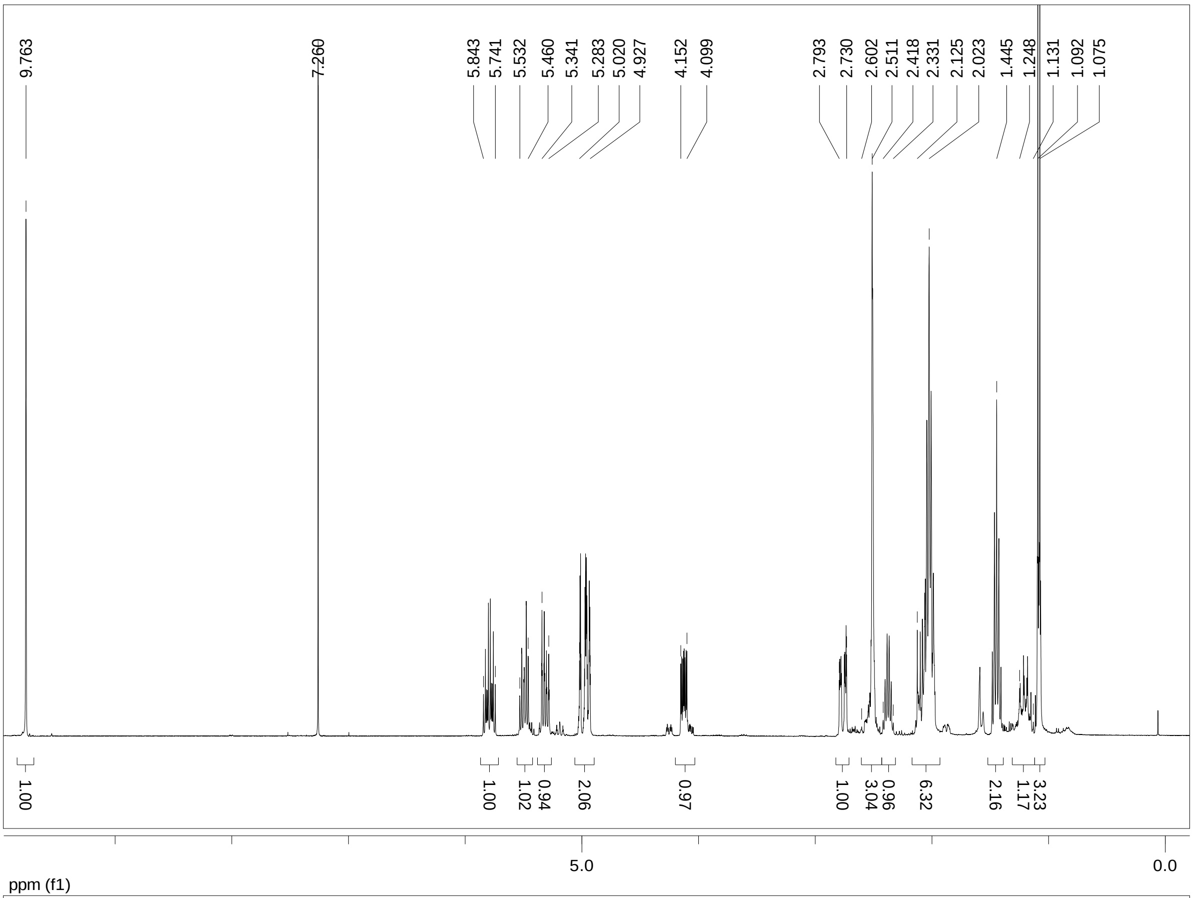
9.763
7.260
5.843
5.741
5.532
5.460
5.341
5.283
5.020
4.927
4.152
4.099
2.793
2.730
2.602
2.511
2.418
2.331
2.125
2.023
1.445
1.248
1.131
1.092
1.075
1.00
1.00
1.02
0.94
2.06
0.97
1.00
3.04
0.96
6.32
2.16
1.17
3.23
5.0
0.0
ppm (f1)

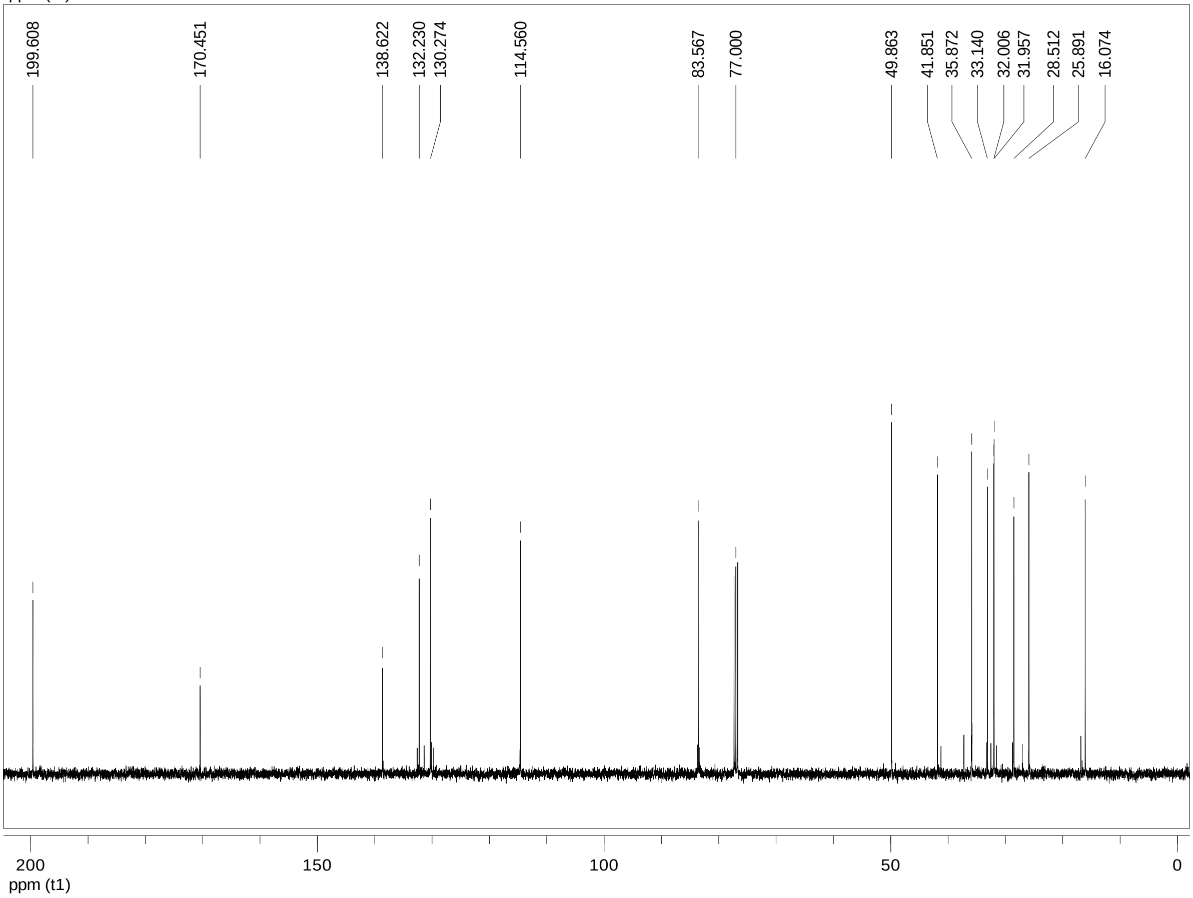
199.608
170.451
138.622
132.230
130.274
114.560
83.567
77.000
49.863
41.851
35.872
33.140
32.006
31.957
28.512
25.891
16.074
200
150
100
50
0
ppm (t1)

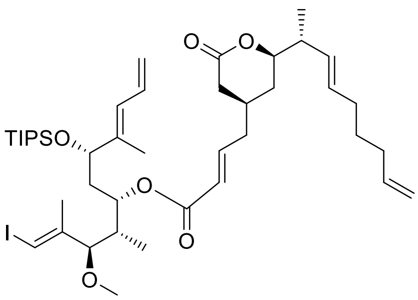

88

(1*E*,3*R*,4*S*,5*S*,7*S*,8*E*)-1-Iodo-3-methoxy-2,4,8-trimethyl-7-((triisopropylsilyl)oxy)-undeca-1,8,10-trien-5-yl (*E*)-4-((2*R*,4*R*)-2-((R,E)-nona-3,8-dien-2-yl)-6-oxotetra-hydro-2H-pyran-4-yl)but-2-enoate (88): To LiCl (5.8 mg, 138 µmol, 1.20 eq) was added a solution of the phosphonate **69** (98.3 mg, 138 µmol, 1.20 eq) in MeCN (0.3 mL, rinsed with 2×0.3 mL), followed by DBU (19.0 µL, 126 µmol, 1.10 eq). After 15 min stirring at rt, the pale yellow mixture was cooled to 0 °C, whereupon the solution turned turbid. As the precipitate settled, stirring became difficult. After 20 min, a solution of the aldehyde **87** (30.3 mg, 115 µmol, 1.00 eq) in MeCN (1.1 mL) was added. The mixture was stirred for 20 min at 0 °C and was then allowed to warm to rt. TLC reaction control after 50 min indicated, that no aldehyde was left. Thus, the mixture was concentrated at a rotavap and the residue was purified by flash chromatography (hex:EtOAc 5:1) to afford the desired product **88** (80.4 mg after HV, 85%) as a colourless oil.

^{1}H-NMR ($CDCl_3$, 400.1 MHz): δ = 6.74 (td, *J* = 7.4 Hz, *J* = 15.0 Hz, 1H), 6.43 (td, *J* = 10.4 Hz, *J* = 16.9 Hz, 1H), 6.16 (bs, 1H), 5.88 (d, *J* = 10.7 Hz, 1H), 5.85-5.72 (m, 2H), 5.51 (td, *J* = 6.8 Hz, *J* = 14.9 Hz, 1H), 5.33 (tdd, *J* = 1.2 Hz, *J* = 7.9 Hz, *J* = 15.4 Hz, 1H), 5.13 (dd, *J* = 1.8 Hz, *J* = 16.8 Hz, 1H), 5.06-4.92 (m, 3H), 4.77 (ddd, *J* = 1.6 Hz, *J* = 4.1 Hz, *J* = 10.2 Hz, 1H), 4.16-4.05 (m, 2H), 3.38 (d, *J* = 7.2 Hz, 1H), 3.16 (s, 3H), 2.67 (m_c, 1H), 2.39 (m_c, 1H), 2.20 (t, *J* = 6.3 Hz, 2H), 2.14-1.98 (m, 7H), 1.98-1.90 (m, 1H), 1.90-1.77 (m, 1H), 1.83 (d, *J* = 0.9 Hz, 3H), 1.76-1.65 (m, 1H), 1.71 (d, *J* = 0.8 Hz, 3H), 1.45 (td, *J* = 7.4 Hz, *J* = 14.8 Hz, 2H), 1.23-1.14 (m, 1H), 1.09 (d, *J* = 6.8 Hz, 3H), 1.06-0.98 (m, 21H), 0.91 (d, *J* = 6.9 Hz, 3H); **^{13}C{^{1}H}-NMR** ($CDCl_3$, 100.6 MHz): δ = 170.7, 165.2, 146.5, 144.3, 140.3, 138.6, 132.6, 132.2, 130.3, 126.4, 124.2, 116.9, 114.6, 87.6, 83.6, 79.0, 76.7, 72.5, 56.7, 41.8, 39.0, 38.6, 36.0, 35.7, 33.2, 32.00, 31.98, 31.0, 28.6, 19.7, 18.2, 18.1, 16.0, 12.4, 11.6, 9.8; **HRMS** Calcd. for $C_{42}H_{69}INaO_6Si$ [M+Na]$^+$ *m/z* 847.2800. Found: 847.3813.

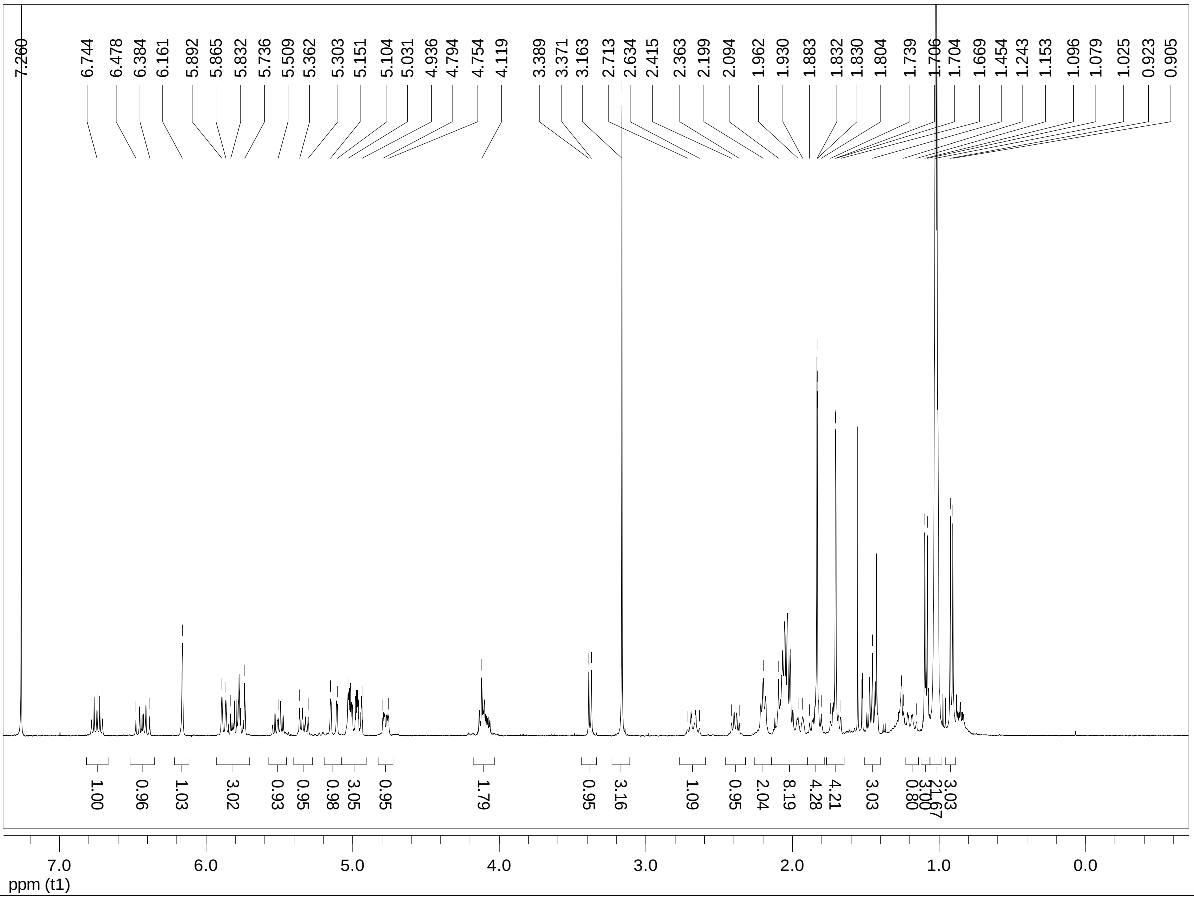
7.260
6.744
6.478
6.384
6.161
5.892
5.865
5.832
5.736
5.509
5.362
5.303
5.151
5.104
5.031
4.936
4.794
4.754
4.119
3.389
3.371
3.163
2.713
2.634
2.415
2.363
2.199
2.094
1.962
1.930
1.883
1.832
1.830
1.804
1.739
1.706
1.704
1.669
1.454
1.243
1.153
1.096
1.079
1.025
0.923
0.905
1.00
0.96
1.03
3.02
0.93
0.95
0.98
3.05
0.95
1.79
0.95
3.16
1.09
0.95
2.04
8.19
4.28
4.21
3.03
0.80
3.03
7.0
6.0
5.0
4.0
3.0
2.0
1.0
0.0
ppm (t1)

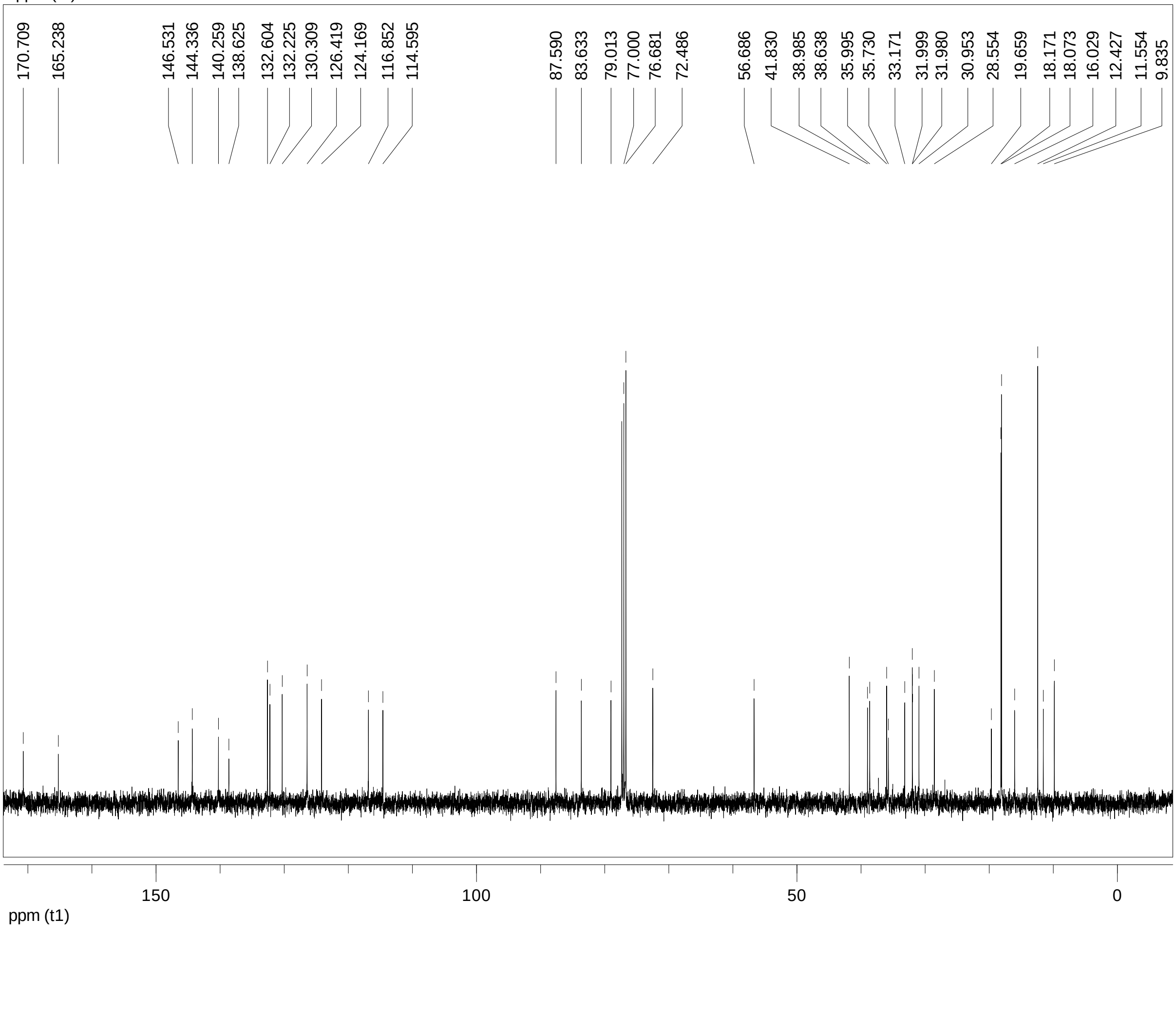
170.709
165.238
146.531
144.336
140.259
138.625
132.604
132.225
130.309
126.419
124.169
116.852
114.595
87.590
83.633
79.013
77.000
76.681
72.486
56.686
41.830
38.985
38.638
35.995
35.730
33.171
31.999
31.980
30.953
28.554
19.659
18.171
18.073
16.029
12.427
11.554
9.835
150
100
50
0
ppm (t1)

Attemped preparation of hydroxy ester 90 by opening of lactone 67:

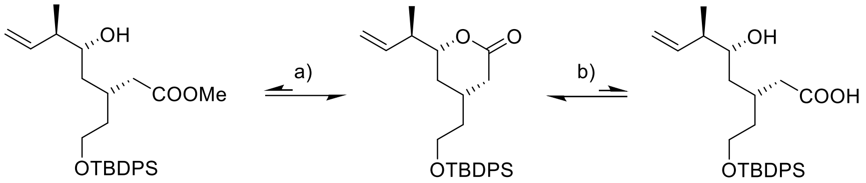

90 **67** **91**

Lactone opening with LiOMe: To a solution of lactone **67** (38.0 mg, 87.0 µmol, 1.0 equiv) in MeOH (3.0 mL) was added at rt a freshly prepared solution of LiOMe in MeOH (prepared by the addition of n-BuLi (0.44 mL, 0.87 mmol, 10 equiv) to 3 mL of MeOH at 0 °C). After stirring for 20 min, conversion was incomplete (TLC) and more LiOMe (10 equiv) was added. After a total reaction time of 60 min the reaction mixture was poured into a mixture of Et_2O (15 mL) and sat. NH_4Cl (20 mL). All volatiles were removed under reduced pressure. The aqueous phase was extracted with Et_2O (3 x 20 mL), the combined organic extracts were dried over $MgSO_4$ and the solvent was removed under reduced pressure. The residue was purified by column chromatography (hexane/EtOAc, 10:1 → 7:1) to afford only the starting lactone **67** (37.2 mg, 98%) as a colorless oil.

Lactone opening with LiOH: To a solution of **67** (37.2 mg, 85.2 µmol, 1.0 equiv) in THF/water (2.5 mL, 1:1 v/v) was added at 0 °C lithium hydroxide monohydrate (35.7 mg, 0.85 mmol, 10 equiv) in one portion. After stirring for 45 min at 0 °C, solid NaH_2PO_4 monohydrate (200 mL) was added and the reaction mixture was diluted with Et_2O (15 mL) and aq. 0.2M NaH_2PO_4 solution (15 mL). The layers were separated and the aqeous layer was extracted with Et_2O (3 x 15 mL). The combined organic extracts were dried over $MgSO_4$ and the solvent was removed under reduced pressure. The crude hydroxy acid (38.0 mg, 98%) was isolated as a colorless oil and used without further purification. To a solution of the crude acid (38.0 mg, 83.6 µmol, 1.0 equiv) in toluene (0.50 mL) and MeOH (0.34 mL) was added at rt $TMSCHN_2$ (46.0 µL, 92 µmol, 1.1 equiv, 2.0M solution in hexane). After stirring for 1 h at rt, the acid was consumed to give lactone **67** exclusively (based on TLC analysis).

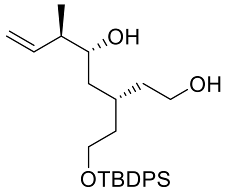

92

(3*R*,5*R*,6*R*)-3-(2-((*tert*-Butyldiphenylsilyl)oxy)ethyl)-6-methyloct-7-ene-1,5-diol (92): To a solution of **67** (28.5 mg, 65.3 µmol, 1.0 equiv) in Et_2O (1.0 mL) was added at 0 °C $LiAlH_4$ (3.7 mg, 97.5 µmol, 1.5 equiv). After stirring for 10 min at 0 °C, the reaction mixture was allowed to warm to rt over 50 min and was quenched at 0 °C by slow addition of sat. *Rochelle* salt (5 mL). The reaction mixture was diluted with Et_2O (10 mL) and brine (10 mL). The layers were separated and the aqueous layer was extracted with Et_2O (2 x 15 mL). The combined organic extracts were dried over $MgSO_4$ and the solvent was removed under reduced pressure. **92** (28.7 mg, quant.) was isolated as a yellowish oil, which was used for the next step without further purification.

R_f = 0.16 (hexane/EtOAc, 2:1)

^{1}H-NMR (400.1 MHz, $CDCl_3$): δ = 7.72-7.63 (m, 4H), 7.47-7.33 (m, 6H), 5.73 (ddd, *J* = 7.3, 9.8, 17.7 Hz, 1H), 5.12-5.01 (m, 2H), 3.76-3.59 (m, 4H), 3.50 (ddd, *J* = 3.1, 5.1, 9.5 Hz, 1H), 2.20 (m_c, 1H), 2.09-1.81 (brs, 2H), 1.86 (m_c, 1H), 1.68-1.49 (m, 3H), 1.49-1.28 (m, 3H), 1.05 (s, 9H), 0.98 (d, *J* = 6.8 Hz, 3H).

^{13}C-NMR (100.6 MHz, $CDCl_3$): δ = 140.8, 135.6, 133.8, 129.6, 127.6, 115.4, 73.3, 62.2, 60.6, 44.0, 38.2, 37.6, 37.5, 28.7, 26.9, 19.1, 14.2.

HRMS (ESI): *m/z* calcd for $C_{27}H_{40}NaO_3Si$ [M + Na]$^+$: 463.2639, found: 463.2631.

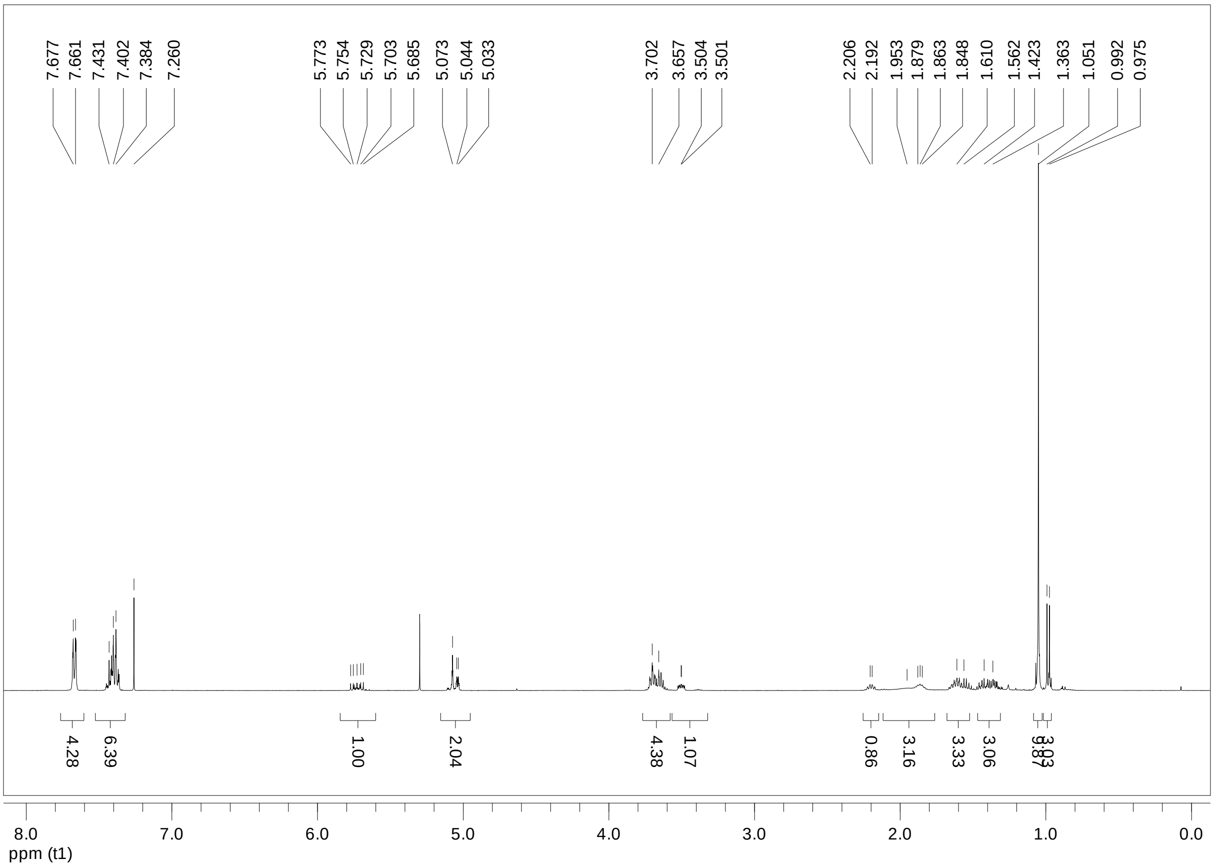
7.677
7.661
7.431
7.402
7.384
7.260
5.773
5.754
5.729
5.703
5.685
5.073
5.044
5.033
3.702
3.657
3.504
3.501
2.206
2.192
1.953
1.879
1.863
1.848
1.610
1.562
1.423
1.363
1.051
0.992
0.975
4.28
6.39
1.00
2.04
4.38
1.07
0.86
3.16
3.33
3.06
8.0
7.0
6.0
5.0
4.0
3.0
2.0
1.0
0.0
ppm (t1)

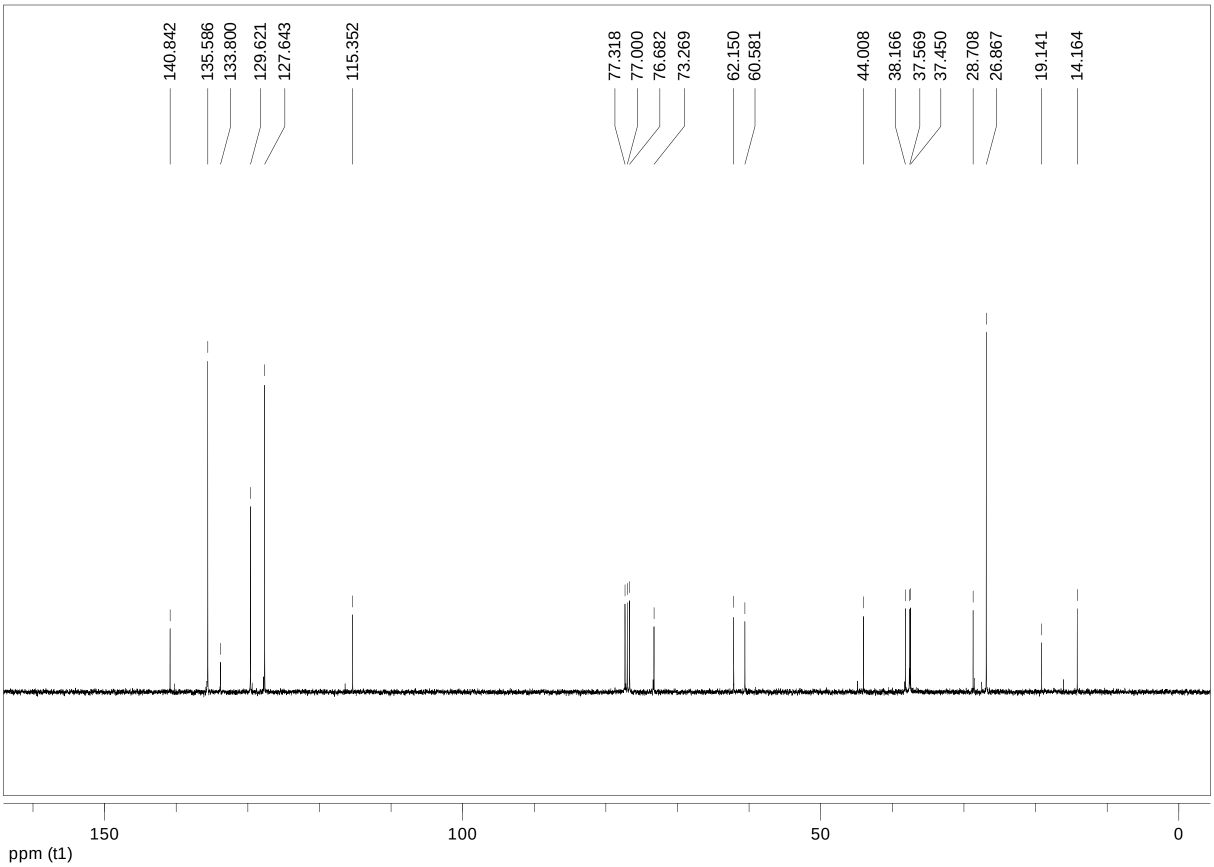
140.842
135.586
133.800
129.621
127.643
115.352
77.318
77.000
76.682
73.269
62.150
60.581
44.008
38.166
37.569
37.450
28.708
26.867
19.141
14.164
150
100
50
0
ppm (t1)

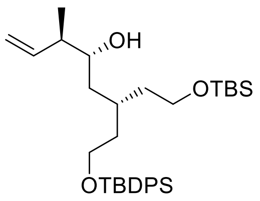

S24

(3*R*,4*R*,6*R*)-8-((*tert*-Butyldimethylsilyl)oxy)-6-(2-((*tert*-butyldiphenylsilyl)-oxy)ethyl)-3-methyloct-1-en-4-ol (S24): To a solution of **92** (33.2 mg, 0.08 mmol, 1.0 equiv) in CH_2Cl_2 (1.1 mL) was added at rt imidazole (10.6 mg, 0.16 mmol, 2.1 equiv) followed by TBSCl (16.3 mg, 0.11 mmol, 1.4 equiv). After stirring for 20 min at rt, the reaction mixture was quenched with brine (10 mL) and diluted with CH_2Cl_2 (9 mL). The layers were separated and the aqueous layer was extracted with CH_2Cl_2 (2 x 15 mL). The combined organic extracts were dried over $MgSO_4$ and the solvent was removed under reduced pressure. The residue was purified by column chromatography (hexane/EtOAc, 10:1) to afford **S24** (40.9 mg, 98% over 2 steps) as a colorless oil.

R_f = 0.26 (hexane/EtOAc, 15:1)

$[\alpha]_D^{24}$ = +14.0° [*c* = 0.515, $CHCl_3$].

^{1}H-NMR (400.1 MHz, $CDCl_3$): δ = 7.71-7.63 (m, 4H), 7.46-7.34 (m, 6H), 5.75 (ddd, *J* = 7.5, 10.7, 16.9 Hz, 1H), 5.09-5.00 (m, 2H), 3.69 (m_c, 2H), 3.63 (m_c, 2H), 3.50 (m_c, 1H), 2.20 (m_c, 1H), 2.05 (d, *J* = 4.6 Hz, 1H), 1.82 (m_c, 1H), 1.68-1.41 (m, 4H), 1.40-1.32 (m, 2H), 1.05 (s, 9H), 0.99 (d, *J* = 6.8 Hz, 3H), 0.88 (s, 9H), 0.04 (s, 6H).

^{13}C-NMR (100.6 MHz, $CDCl_3$): δ = 141.2, 135.6, 133.9, 129.6, 127.6, 114.9, 73.0, 62.2, 61.3, 44.0, 38.7, 37.5, 37.0, 29.0, 26.9, 26.0, 19.1, 18.3, 14.5, –5.3.

IR (neat, ν/cm^{-1}): 3450brs, 2955m, 2929m, 2858m, 1472w, 1428w, 1389w, 1254m, 1106s, 1092s, 1005w, 835m, 776m, 738m, 702s, 614w, 506m.

HRMS (ESI): *m/z* calcd for $C_{33}H_{55}O_3Si_2$ $[M + H]^+$: 555.3684, found: 555.3682.

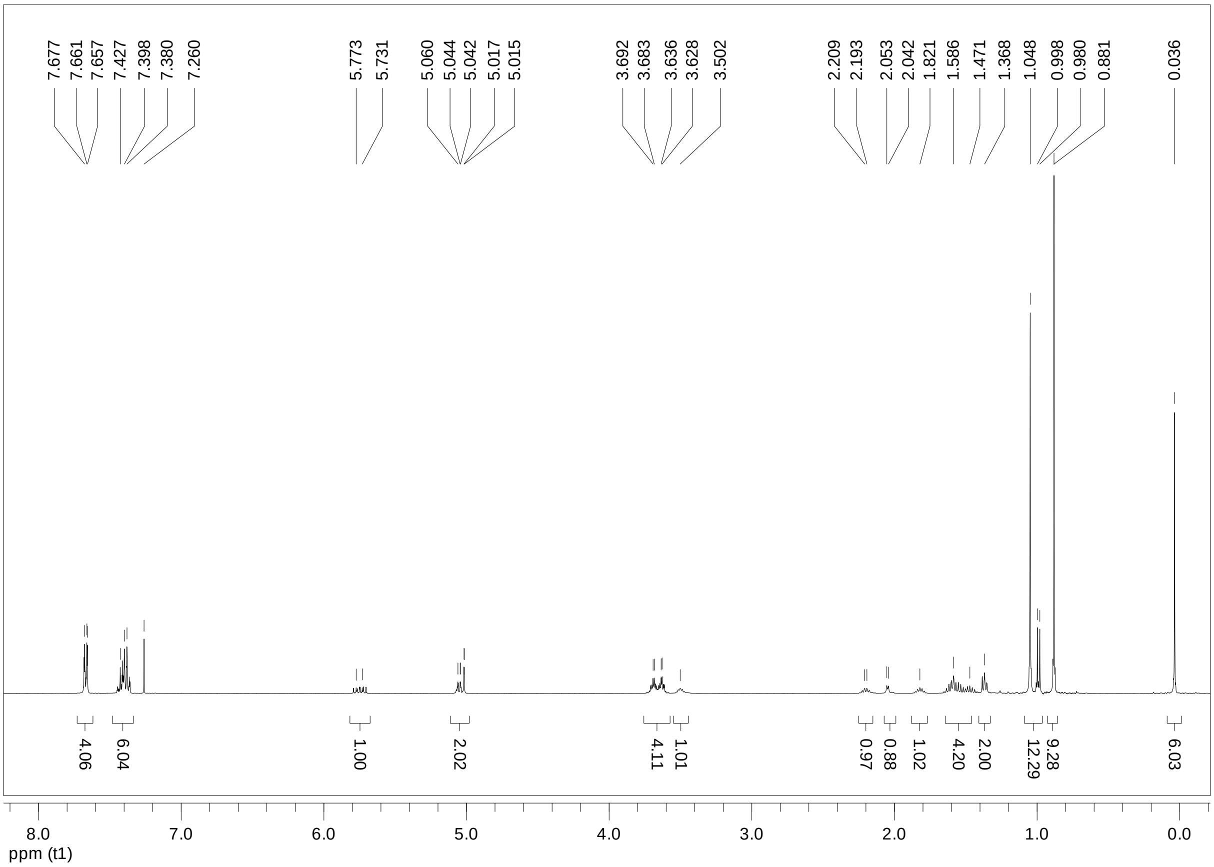
7.677
7.661
7.657
7.427
7.398
7.380
7.260
5.773
5.731
5.060
5.044
5.042
5.017
5.015
3.692
3.683
3.636
3.628
3.502
2.209
2.193
2.053
2.042
1.821
1.586
1.471
1.368
1.048
0.998
0.980
0.881
0.036
4.06
6.04
1.00
2.02
4.11
1.01
0.97
0.88
1.02
4.20
2.00
12.29
9.28
6.03
8.0
7.0
6.0
5.0
4.0
3.0
2.0
1.0
0.0
ppm (t1)

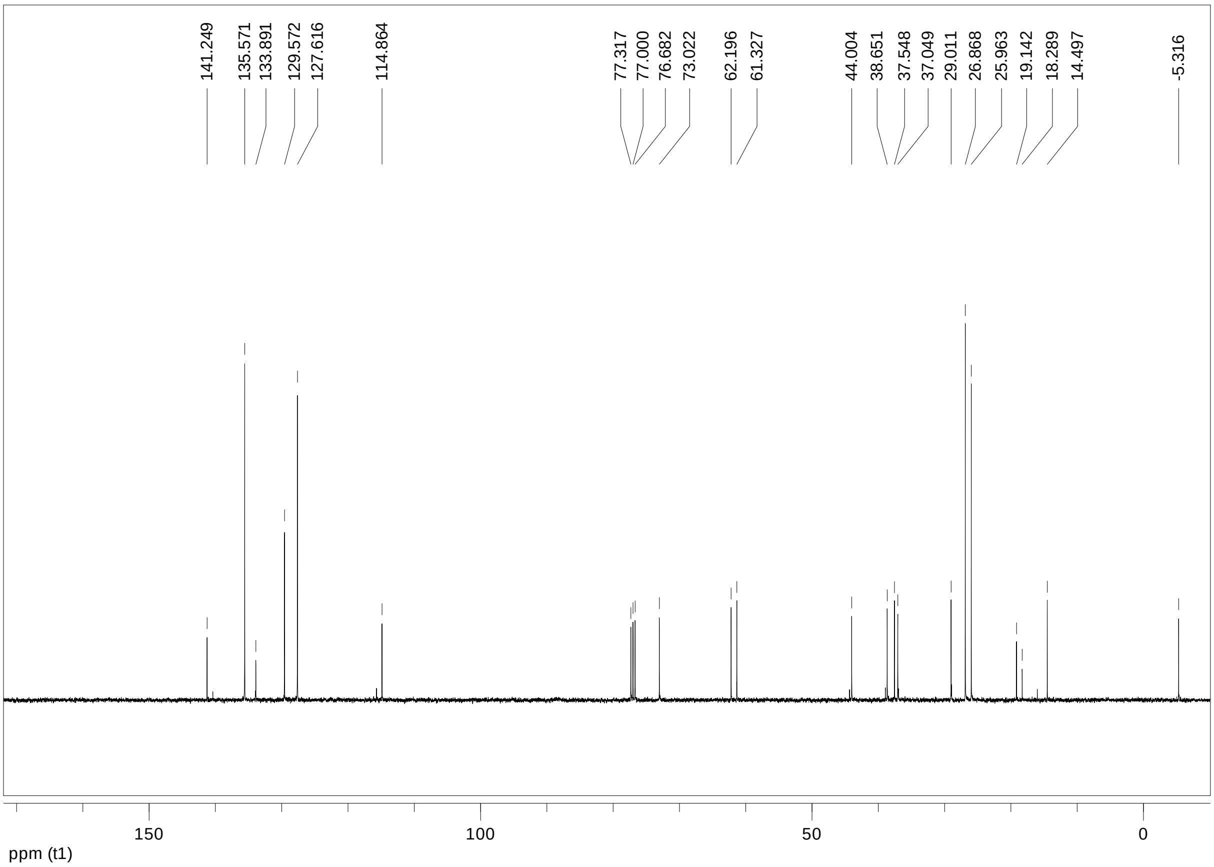
141.249
135.571
133.891
129.572
127.616
114.864
77.317
77.000
76.682
73.022
62.196
61.327
44.004
38.651
37.548
37.049
29.011
26.868
25.963
19.142
18.289
14.497
-5.316
150
100
50
0
ppm (t1)

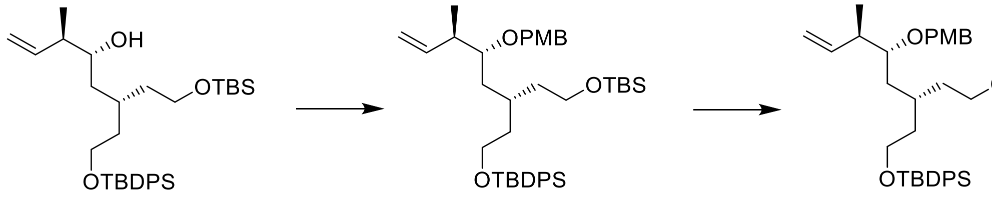

S24 **S25** **S26**

(3*R*,5*R*,6*R*)-3-(2-((*tert*-Butyldiphenylsilyl)oxy)ethyl)-5-((4-methoxybenzyl)oxy)-6-methyloct-7-en-1-ol (S26): To a solution of **S24** (40.9 mg, 0.07 mmol, 1.0 equiv) and PMB-2,2,2-trichloroacetimidate (55.0 mg, 0.19 mmol, 2.6 equiv) in toluene (1.5 mL) was added at rt $Sc(OTf)_3$ (2.3 mg, 6.3 mol%). After stirring for 1.5 h at rt, the reaction mixture was quenched with sat. $NaHCO_3$ (5 mL) and diluted with brine (10 mL)/Et_2O (10 mL). The layers were separated and the aqueous layer was extracted with Et_2O (2 x 20 mL). The combined organic extracts were dried over $MgSO_4$ and the solvent was removed under reduced pressure. The residue was purified by column chromatography (hexane/EtOAc, 50:1 → 30:1 → 10:1) to afford **S25** (44.1 mg, <89%, impure) as a mixture with impurities originating from PMB-2,2,2-trichloroacetimidate. **R*f*** = 0.38 (hexane/EtOAc, 15:1). To a solution of **S25** (44.1 mg, 65.3 µmol, 1.0 equiv) in THF/water (4:1, 0.71 mL) was added sodium periodate (84.2 mg, 0.39 mmol, 6.0 equiv). After stirring at rt for 15 h, the reaction mixture was quenched with water (10 mL) and diluted with CH_2Cl_2 (15 mL). The layers were separated and the aqueous layer was extracted with CH_2Cl_2 (2 x 20 mL). The combined organic extracts were dried over $MgSO_4$ and concentrated under reduced pressure. The residue was purified by column chromatography (hexane/EtOAc, 5:1 → 4:1) to give **S26** (27.8 mg, 76%, 67% over 2 steps) as a colorless oil.

R*f* = 0.14 (hexane/EtOAc, 5:1)

$[\alpha]_D^{24}$ = +34.0° [*c* = 0.365, $CHCl_3$]

^{1}H-NMR (400.1 MHz, $CDCl_3$): δ = 7.73-7.61 (m, 4H), 7.49-7.33 (m, 6H), 7.21 (d, *J* = 8.7 Hz, 2H), 6.83 (d, *J* = 8.7 Hz, 2H), 5.86 (ddd, *J* = 6.8, 11.4, 16.3 Hz, 1H), 5.06-4.97 (m, 2H), 4.53 (d, *J* = 10.8 Hz, 1H), 4.32 (d, *J* = 10.8 Hz, 1H), 3.77 (s, 3H), 3.67 (m_c, 2H), 3.58 (m_c, 2H), 3.29 (ddd, *J* = 2.9, 4.3, 9.7 Hz, 1H), 2.55 (m_c, 1H), 1.89 (brs, 1H), 1.76 (m_c, 1H), 1.68-1.41 (m, 4H), 1.41-1.30 (m, 1H), 1.28-1.18 (m, 1H), 1.06 (s, 9H), 0.98 (d, *J* = 6.9 Hz, 3H).

^{13}C-NMR (100.6 MHz, $CDCl_3$): δ = 159.1, 140.3, 135.6, 133.9, 130.5, 129.6, 129.4, 127.6, 114.5, 113.8, 81.7, 71.5, 62.0, 60.7, 55.2, 39.9, 37.7, 37.4, 35.2, 28.4, 26.9, 19.2, 15.1.

IR (neat, ν/cm[-1]): 3423brs, 2931m, 2857m, 1613w, 1513m, 1428m, 1302w, 1248m, 1173w, 1110s, 1084s, 1037m, 822m, 739m, 702s, 506s, 488m.

HRMS (ESI): *m/z* calcd for $C_{35}H_{48}NaO_4Si$ [M + Na]$^+$: 583.3214, found: 583.3216.

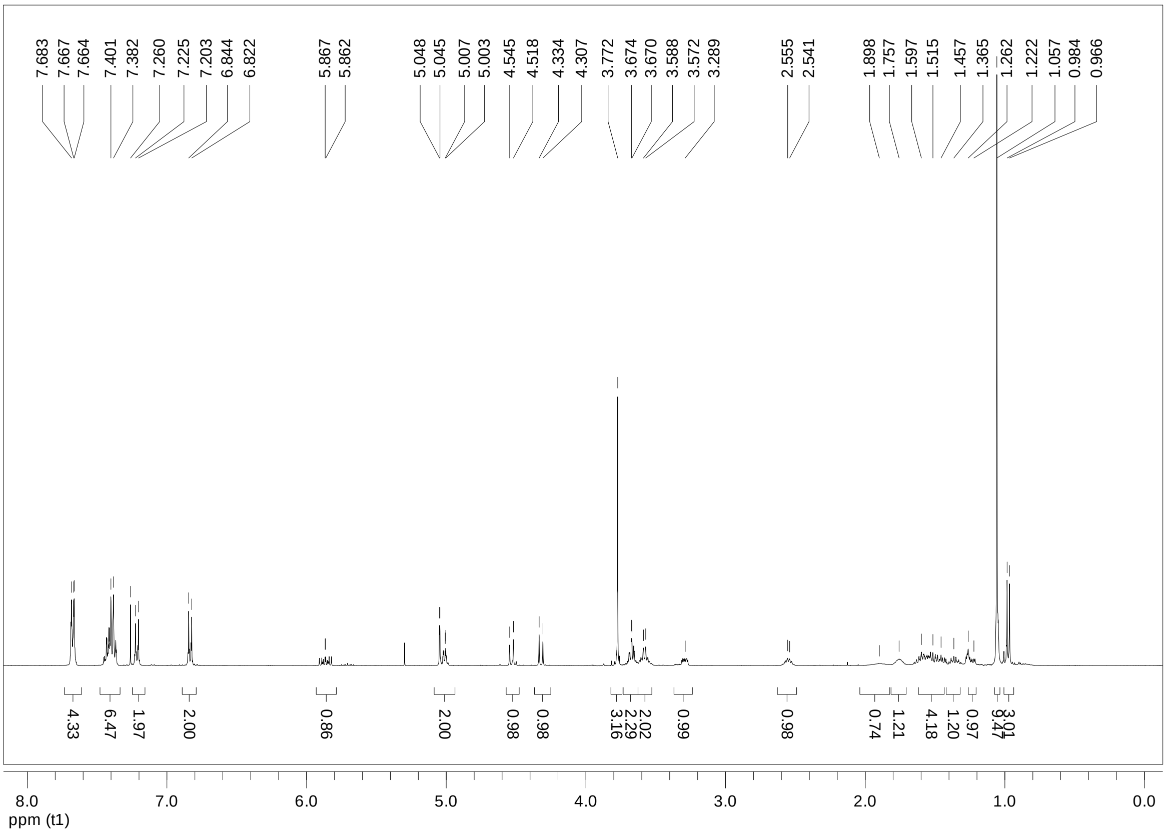

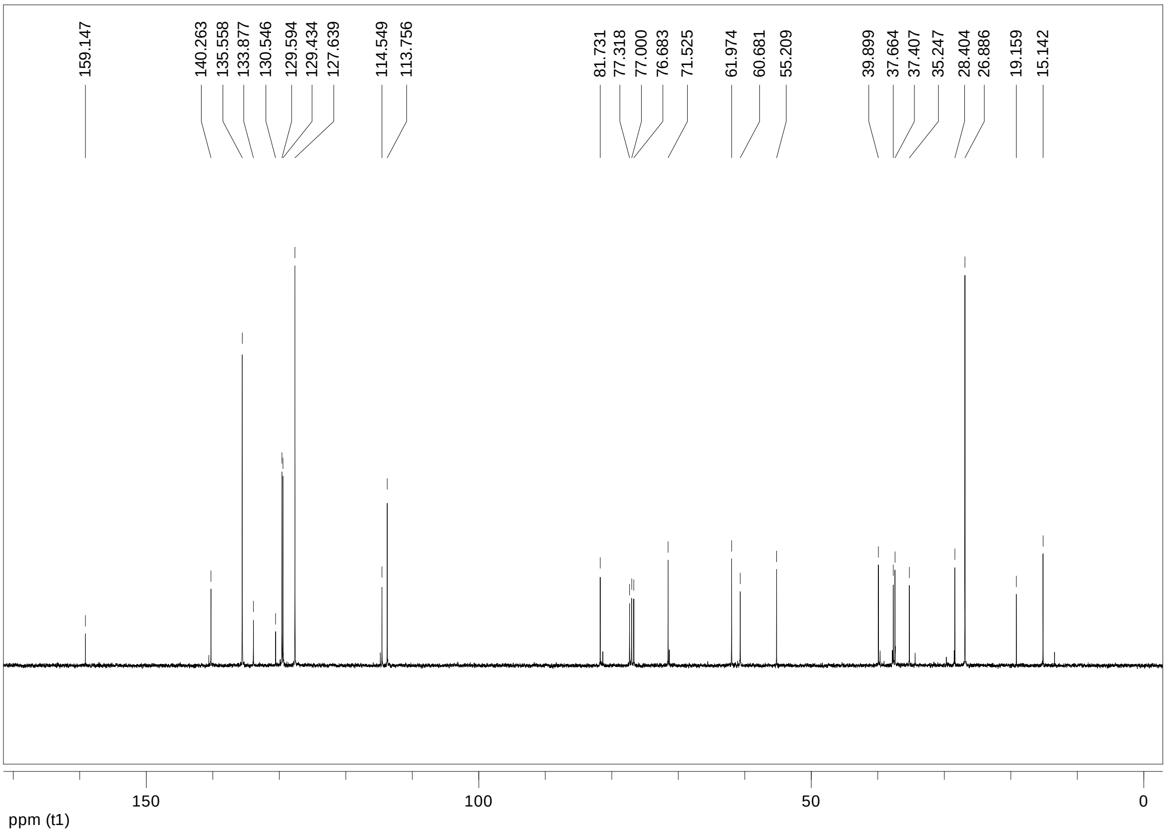

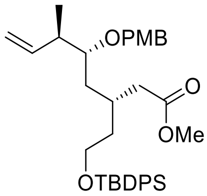

93

Methyl (3*R*,5*R*,6*R*)-3-(2-((*tert*-butyldiphenylsilyl)oxy)ethyl)-5-((4-methoxy-benzyl)oxy)-6-methyloct-7-enoate (93): To a solution of oxalyl chloride (6.3 µL, 74.4 µmol, 1.5 equiv) in CH_2Cl_2 (0.60 mL) at –78 °C was added dropwise DMSO (10.6 µL, 149 µmol, 3.0 equiv). After stirring at –78 °C for 10 min, a solution of **S26** (27.8 mg, 49.6 µmol, 1.0 equiv) in CH_2Cl_2 (0.9 mL) was added dropwise. The resultant cloudy mixture was stirred at –78 °C for 1.5 h, and then TEA (27.6 µL, 198 µmol, 4.0 equiv) was added slowly and the reaction mixture was allowed to warm to room temperature (1 h). The reaction was quenched with water (10 mL), and the layers were separated. The aqueous phase was extracted with CH_2Cl_2 (3 x 20 mL) and the combined organic extracts were washed with brine, dried over $MgSO_4$, filtered and concentrated *in vacuo*. The crude aldehyde (34.3 mg) was isolated as colorless crystals and was used for the next step without further purification.

To a solution of the aldehyde (34.3 mg, crude) in THF (0.30 mL), *tert*-butanol (0.58 mL) and 2-methyl-2-butene (0.21 mL) was added at rt an aqeuous solution of $NaClO_2$ (56.6 mg, 0.63 mmol, 13 equiv) and NaH_2PO_4 monohydrate (86.4 mg, 0.63 mmol, 13 equiv) in water (0.39 mL). After stirring the yellow reaction mixture for 2 h at rt, sat. aq. NaH_2PO_4 (4 mL) was added and the biphasic mixture was stirred for 15 min. EtOAc (10 mL) and brine (5 mL) was added and the layers were separated. The aqueous layer was extracted with EtOAc (3 x 15 mL). The combined organic extracts were dried over $MgSO_4$, filtered and the solvent was removed under reduced pressure to afford the acid (32.5 mg, crude) as a yellow oil, which was used for the next step without further purification.

To a solution of the crude acid (32.5 mg, crude) in toluene (0.30 mL) and MeOH (0.20 mL) was added at 0 °C $TMSCHN_2$ (37.0 µL, 74 µmol, 1.5 equiv, 2.0 M solution in hexane). After stirring for 30 min at 0 °C, the reaction mixture was quenched slowly by the dropwise addition of an AcOH/MeOH solution (10:1), until the yellow color disappeared. The solvent was removed under reduced pressure. The residue was purified by column chromatography (hexane/EtOAc, 15:1 → 10:1) to afford **93** (23.0 mg, 79% over 3 steps) as a colorless oil.

R*f* = 0.59 (hexane/EtOAc, 5:1)

$[\alpha]_D^{24}$ = +19.2° [*c* = 0.520, $CHCl_3$]

^{1}H-NMR (400.1 MHz, $CDCl_3$): δ = 7.70-7.63 (m, 4H), 7.46-7.32 (m, 6H), 7.24 (d, *J* = 8.7 Hz, 2H), 6.83 (d, *J* = 8.7 Hz, 2H), 5.84 (m_c, 1H), 5.07-4.96 (m, 2H), 4.49 (d, *J* = 10.9 Hz, 1H), 4.36 (d, *J* = 10.9 Hz, 1H), 3.78 (s, 3H), 3.67 (m_c, 2H), 3.61 (s, 3H), 3.31 (td, *J* = 4.8, 7.4 Hz, 1H), 2.50 (m_c, 1H), 2.36-2.27 (m, 2H), 2.22 (m_c, 1H), 1.74-1.60 (m, 1H), 1.59-1.47 (m, 1H), 1.47-1.38 (m, 2H), 1.04 (s, 9H), 0.99 (d, *J* = 6.9 Hz, 3H).

^{13}C-NMR (100.6 MHz, $CDCl_3$): δ = 173.4, 159.0, 140.6, 135.6, 133.9, 130.9, 129.6, 129.3, 127.6, 114.5, 113.7, 80.7, 71.0, 61.7, 55.2, 51.3, 40.3, 39.4, 36.3, 35.8, 29.4, 26.8, 19.1, 15.1.

IR (neat, ν/cm^{-1}): 3072w, 2932m, 2857m, 1737s, 1613w, 1513s, 1463w, 1429m, 1248s, 1171m, 1110s, 1091s, 822m, 739m, 703s, 614w, 506m.

HRMS (ESI): *m/z* calcd for $C_{36}H_{48}NaO_5Si$ [M + Na]$^+$: 611.3163, found: 611.3163.

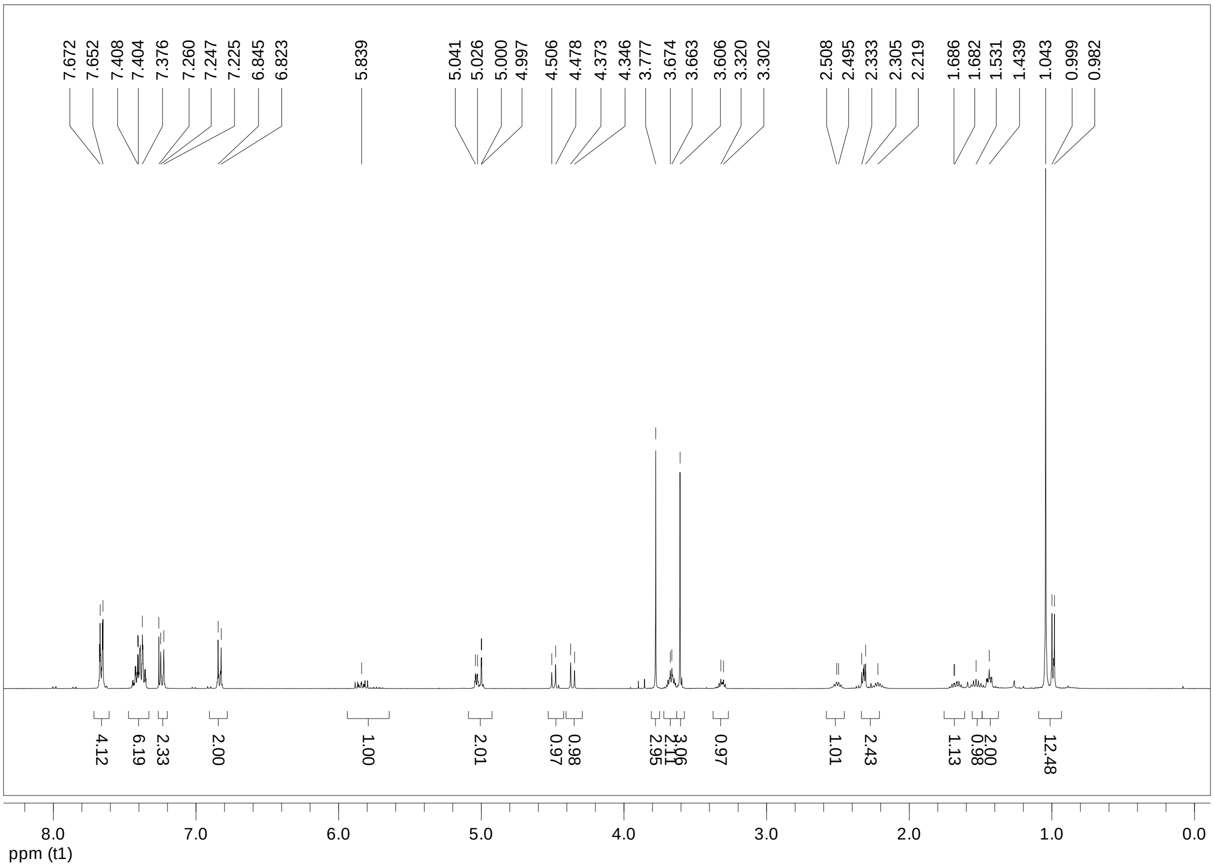
7.672 7.652 7.408 7.404 7.376 7.260 7.247 7.225 6.845 6.823
5.839
5.041 5.026 5.000 4.997 4.506 4.478 4.373 4.346 3.777 3.674 3.663 3.606 3.320 3.302
2.508 2.495 2.333 2.305 2.219 1.686 1.682 1.531 1.439 1.043 0.999 0.982
4.12 6.19 2.33 2.00 1.00 2.01 0.97 0.98 2.95 2.11 3.06 0.97 1.01 2.43 1.13 0.98 2.00 12.48
8.0 7.0 6.0 5.0 4.0 3.0 2.0 1.0 0.0
ppm (t1)

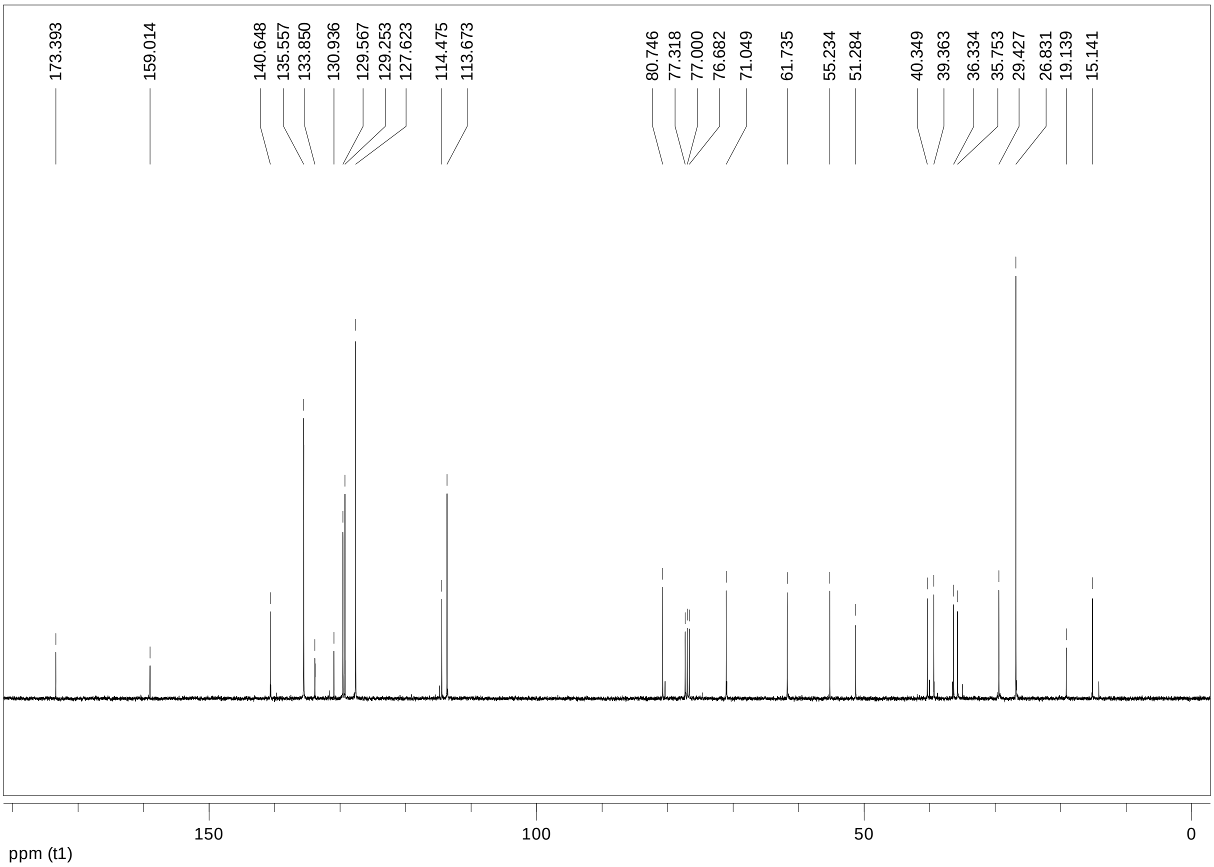
173.393 159.014 140.648 135.557 133.850 130.936 129.567 129.253 127.623 114.475 113.673
80.746 77.318 77.000 76.682 71.049 61.735 55.234 51.284 40.349 39.363 36.334 35.753 29.427 26.831 19.139 15.141
150 100 50 0
ppm (t1)

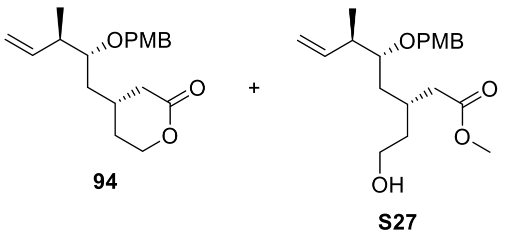

(*R*)-4-((2*R*,3*R*)-2-((4-Methoxybenzyl)oxy)-3-methylpent-4-en-1-yl)tetrahydro-2H-pyran-2-one (94): To a solution of **93** (23.0 mg, 39.1 µmol, 1.0 equiv) in THF (0.39 mL) was added at rt AcOH (11.5 µL, 0.20 mol, 5.1 equiv) and TBAF (0.20 mL, 0.20 mmol, 5.1 equiv, 1.0 M in THF). After stirring the reaction mixture for 20 h at rt, sat. NH_4Cl (15 mL) and EtOAc (15 mL) were added. The layers were separated and the aqueous layer was extracted with EtOAc (3 x 10 mL). The combined organic extracts were washed with brine, dried over $MgSO_4$ and the solvent was removed under reduced pressure. The residue was purified by column chromatography (hexane/EtOAc, 2:1) to give a separable mixture[20] of **94/S27** (11.0 mg, 88%, 9:1) as a colorless oil.

Lactone **94**:[21]

R*f* = 0.33 (hexane/EtOAc, 2:1)

$[\alpha]_D^{24}$ = +53.9° [*c* = 0.330, $CHCl_3$]

^{1}H-NMR (400.1 MHz, $CDCl_3$): δ = 7.24 (d, *J* = 8.6 Hz, 2H), 6.88 (d, *J* = 8.7 Hz, 2H), 5.86 (ddd, *J* = 7.0, 10.7, 17.3 Hz, 1H), 5.13-5.02 (m, 2H), 4.59 (d, *J* = 11.3 Hz, 1H), 4.34 (d, *J* = 11.3 Hz, 1H), 4.33-4.26 (m, 1H), 4.07 (dt, *J* = 3.7, 11.0 Hz, 1H), 3.80 (s, 3H), 3.33 (ddd, *J* = 2.8, 4.8, 9.8 Hz, 1H), 2.70-2.54 (m, 2H), 2.17-2.02 (m, 2H), 1.74-1.62 (m, 1H), 1.53-1.42 (m, 1H), 1.40-1.20 (m, 2H), 1.03 (d, *J* = 6.9 Hz, 3H).

^{13}C-NMR (100.6 MHz, $CDCl_3$): δ = 171.2, 159.3, 139.7, 130.4, 129.7, 115.1, 113.8, 78.8, 70.9, 68.4, 55.3, 39.7, 37.2, 37.1, 28.3, 28.1, 15.4.

IR (neat, ν/cm^{-1}): 3079w, 2961m, 2932m, 1736s, 1612m, 1513s, 1463w, 1401w, 1248s, 1219m, 1173m, 1086m, 1068m, 1034m, 916w, 821m, 771m.

HRMS (ESI): *m/z* calcd for $C_{19}H_{26}NaO_4$ $[M + Na]^+$: 341.1723, found: 341.1725.

Hydroxy methylester **S27**:

R*f* = 0.21 (hexane/EtOAc, 2:1).

---

[20] All fractions containing **94** and **S27** were combined.

[21] Pure Lactone **94** was obtained by treatment of **S27** with PPTS (15 mol%) in MeOH at rt for 16 h (73% yield).

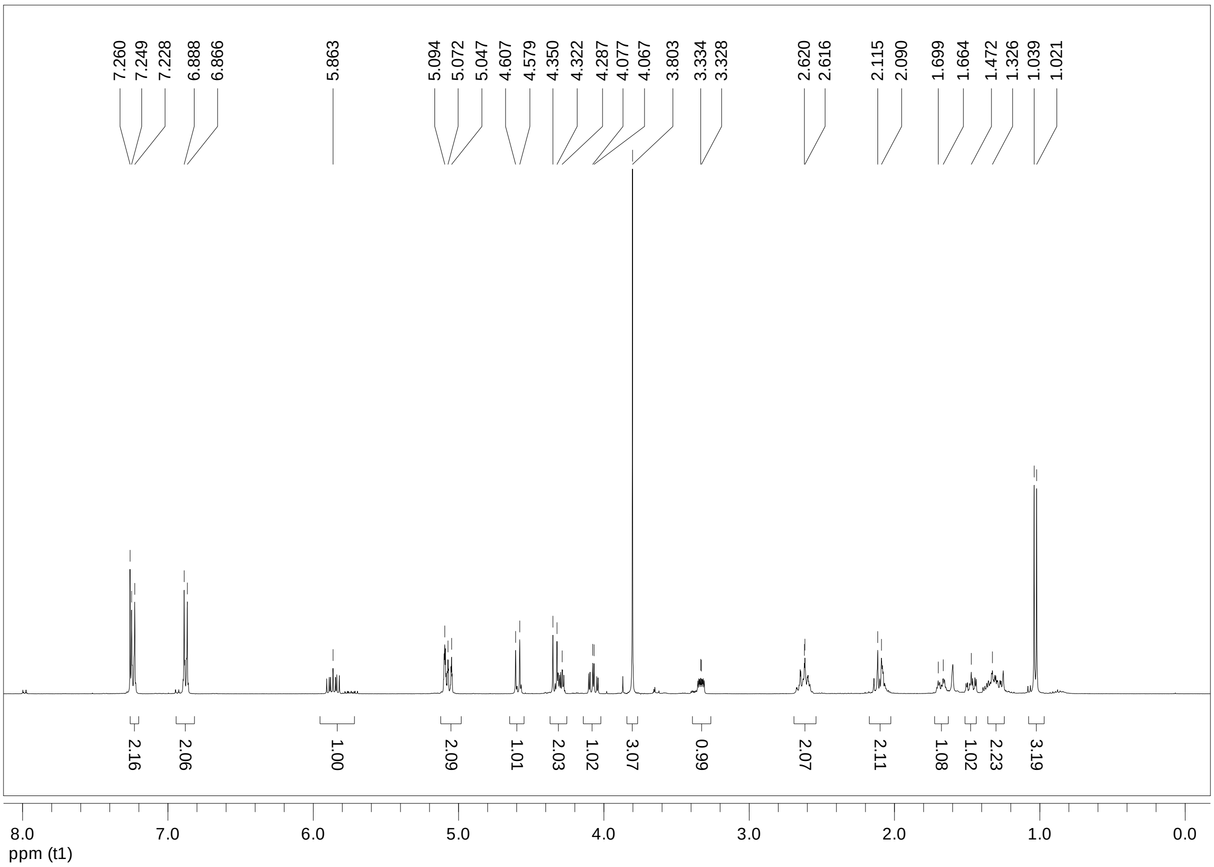
7.260
7.249
7.228
6.888
6.866
5.863
5.094
5.072
5.047
4.607
4.579
4.350
4.322
4.287
4.077
4.067
3.803
3.334
3.328
2.620
2.616
2.115
2.090
1.699
1.664
1.472
1.326
1.039
1.021
2.16
2.06
1.00
2.09
1.01
2.03
1.02
3.07
0.99
2.07
2.11
1.08
1.02
2.23
3.19
8.0
7.0
6.0
5.0
4.0
3.0
2.0
1.0
0.0
ppm (t1)

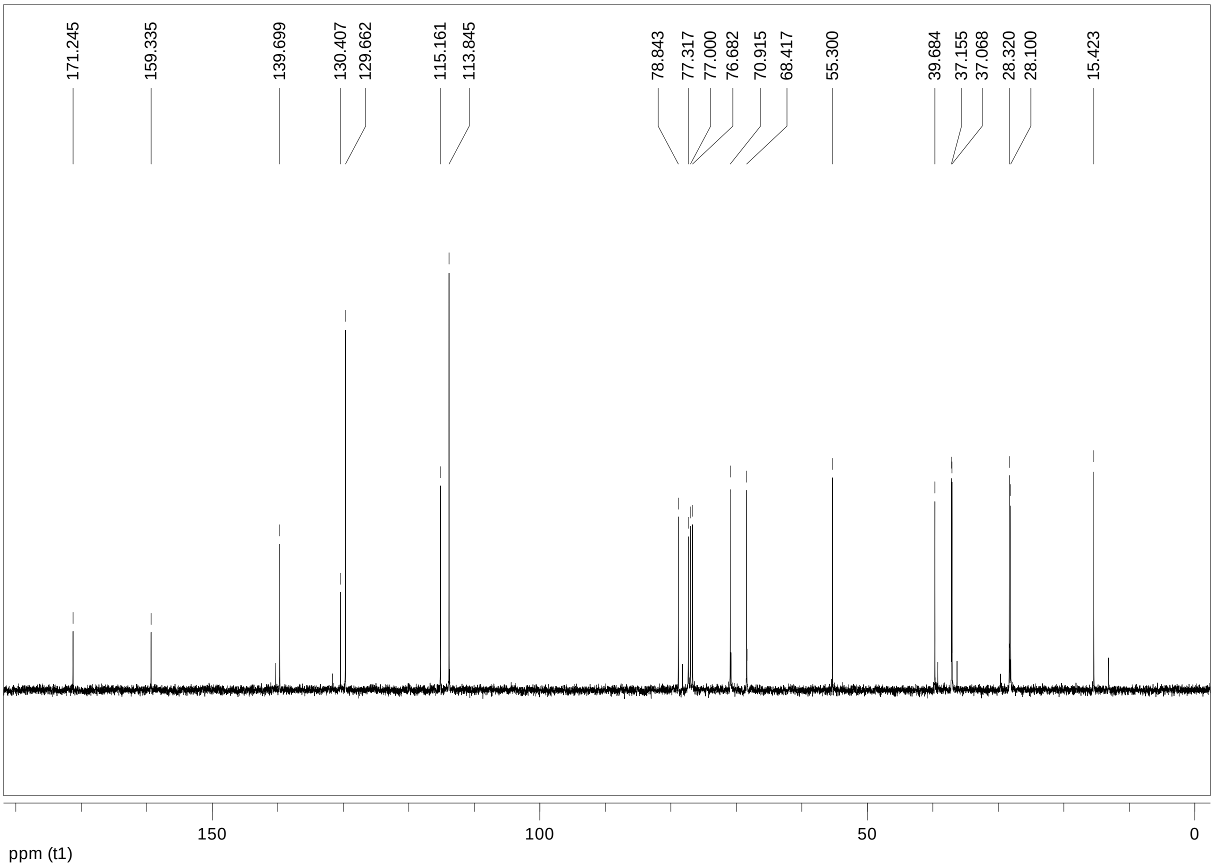
171.245
159.335
139.699
130.407
129.662
115.161
113.845
78.843
77.317
77.000
76.682
70.915
68.417
55.300
39.684
37.155
37.068
28.320
28.100
15.423
150
100
50
0
ppm (t1)

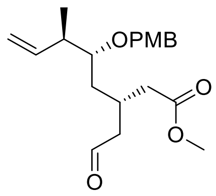

92

Methyl (3*S*,5*R*,6*R*)-5-((4-methoxybenzyl)oxy)-6-methyl-3-(2-oxoethyl)oct-7-enoate (95): To a solution of **94** (6.6 mg, 20.7 µmol, 1.0 equiv) in MeOH (0.9 mL) was added at rt a freshly prepared solution of LiOMe in MeOH (1.36 mL, 30.2 µmol, 1.5 equiv) (prepared by addition of *n*-BuLi (50 µL, 0.10 mmol) to MeOH (4.5 mL) at 0 °C → 22.2 µm). After stirring for 1 h at rt, the reaction mixture was quenched by simultaneous addition of Et_2O (1.2 mL) and sat. NH_4Cl (1.8 mL). All volatiles were removed under reduced pressure. The aqueous phase was extracted with Et_2O (3 x 20 mL). The combined organic extracts were dried over $MgSO_4$ and the solvent was removed under reduced pressure. The crude hydroxyester **S27** (along with some remaining lactone **94**) was used for the next step without further purification due to danger of lactonization.

To a solution of the crude hydroxyester **S27** (8.0 mg) was added solid sodium bicarbonate (7.8 mg, 4.5 equiv) followed by CH_2Cl_2 (6.8 mL). After cooling to 0 °C, *Dess-Martin* periodinane (11.4 mg, 26.9 µmol, 1.3 equiv) was added in one portion. After stirring for 1 h at rt, sat. $Na_2S_2O_3$ (3 mL) and sat. $NaHCO_3$ (3 mL) were added simultaneously. This biphasic mixture was diluted with Et_2O (20 mL) and brine. The layers were separated and the aqueous layer was extracted with Et_2O (2 x 10 mL). The combined organic extracts were dried over $MgSO_4$, filtered and concentrated under reduced pressure. The residue was purified by column chromatography (hexane/EtOAc, 2:1) to afford **95** (4.9 mg, 68%, 93% brsm) as a colorless oil. In addition, the starting material **94** (1.8 mg, 27%) was recovered.

$\mathbf{R}_f$ = 0.62 (hexane/EtOAc, 5:1)

$[\alpha]_D^{24}$ = +40.1° [*c* = 0.245, $CHCl_3$]

^{1}H-NMR (400.1 MHz, $CDCl_3$): δ = 9.62 (t, *J* = 1.8 Hz, 1H), 7.26 (d, *J* = 8.6 Hz, 2H), 6.87 (d, *J* = 8.7 Hz, 2H), 5.86 (m_c, 1H), 5.09-5.06 (m, 1H), 5.06-5.02 (m, 1H), 4.56 (d, *J* = 11.0 Hz, 1H), 4.34 (d, *J* = 11.0 Hz, 1H), 3.80 (s, 3H), 3.63 (s, 3H), 3.32 (ddd, *J* = 3.6, 4.9, 8.7 Hz, 1H), 2.59 (m_c, 2H), 2.47-2.35 (m, 3H), 2.31 (dd, *J* = 7.6, 15.6 Hz, 1H), 1.57-1.40 (m, 2H), 1.02 (d, *J* = 6.9 Hz, 3H).

^{13}C-NMR (100.6 MHz, $CDCl_3$): δ = 201.8, 172.8, 159.2, 139.9, 130.5, 129.5, 114.9, 113.8, 79.9, 70.5, 55.3, 51.5, 47.6, 39.6, 39.1, 35.0, 27.1, 15.3.

IR (neat, ν/cm^{-1}): 2952m, 2935m, 1732s, 1612m, 1512s, 1462w, 1438m, 1373w, 1302m, 1249s, 1172m, 1065w, 1034m, 917w, 821m.

HRMS (ESI): *m/z* calcd for $C_{19}H_{26}NaO_4$ $[M + Na]^+$: 341.1723, found: 341.1725.

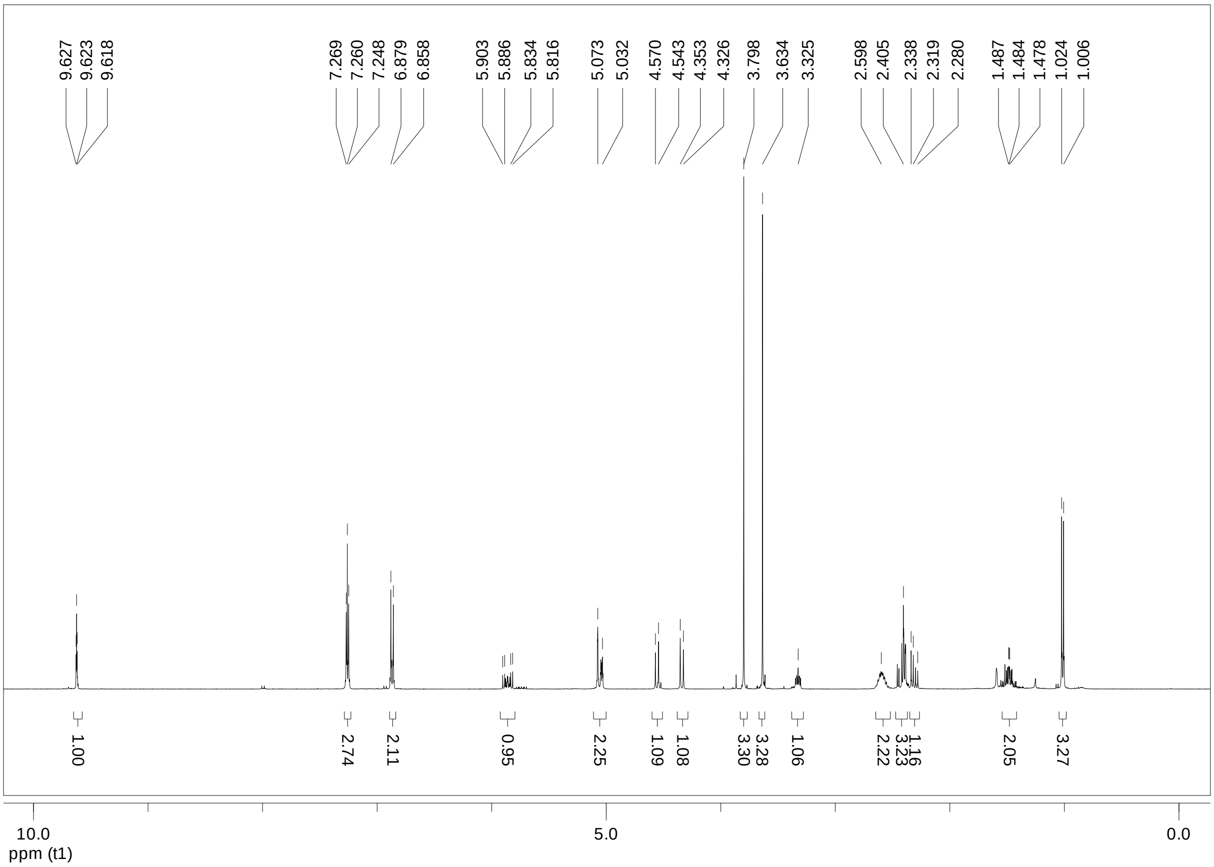
9.627
9.623
9.618
7.269
7.260
7.248
6.879
6.858
5.903
5.886
5.834
5.816
5.073
5.032
4.570
4.543
4.353
4.326
3.798
3.634
3.325
2.598
2.405
2.338
2.319
2.280
1.487
1.484
1.478
1.024
1.006
1.00
2.74
2.11
0.95
2.25
1.09
1.08
3.30
3.28
1.06
2.22
3.23
1.16
2.05
3.27
10.0
5.0
0.0
ppm (t1)

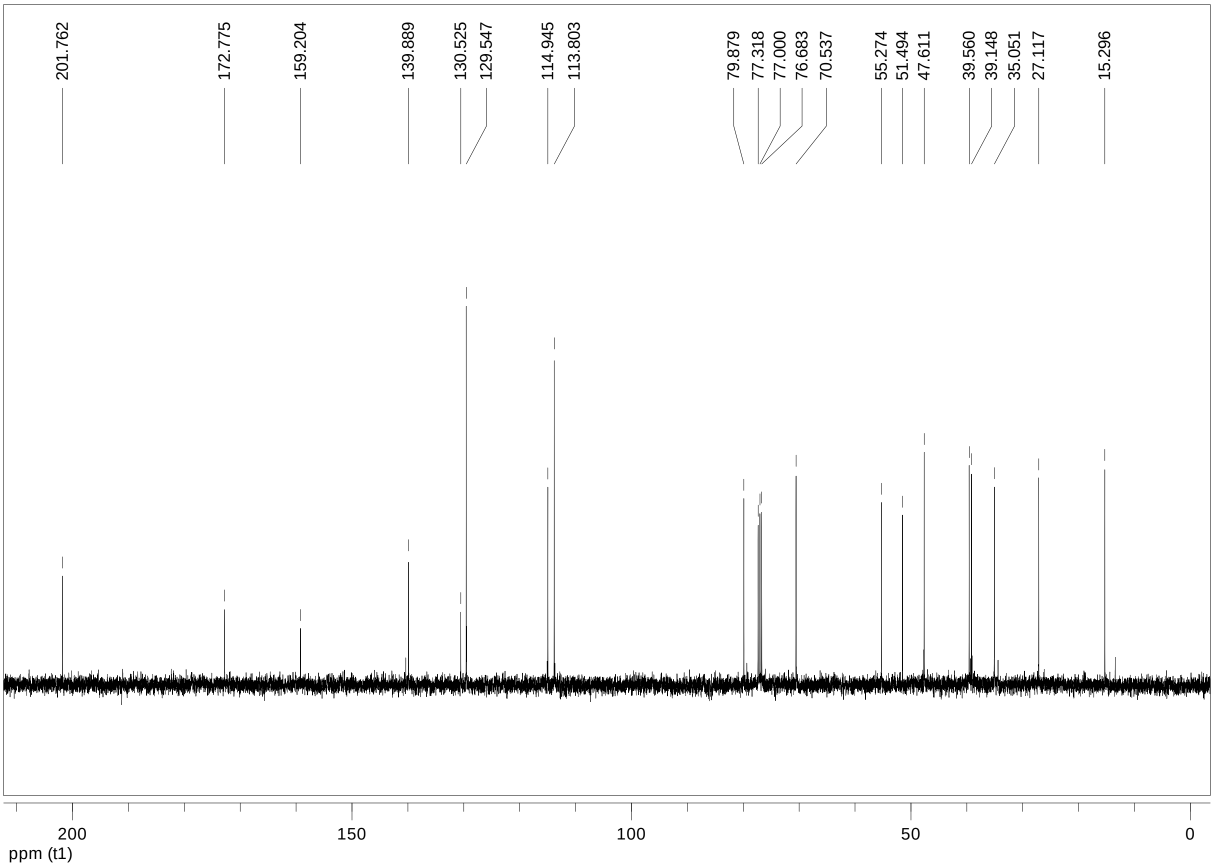
201.762
172.775
159.204
139.889
130.525
129.547
114.945
113.803
79.879
77.318
77.000
76.683
70.537
55.274
51.494
47.611
39.560
39.148
35.051
27.117
15.296
200
150
100
50
0
ppm (t1)

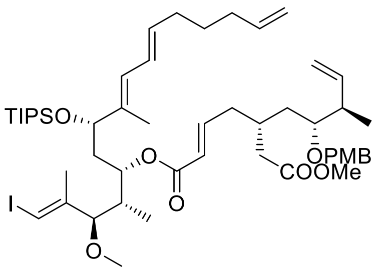

89

1-((1*E*,3*R*,4*S*,5*S*,7*S*,8*E*,10*E*)-1-Iodo-3-methoxy-2,4,8-trimethyl-7-((triisopropyl-silyl)oxy)hexadeca-1,8,10,15-tetraen-5-yl) 7-methyl (*R*,*E*)-5-((2*R*,3*R*)-2-((4-methoxybenzyl)oxy)-3-methylpent-4-en-1-yl)hept-2-enedioate (89): To a mixture of LiCl (0.9 mg, 21.2 µmol, 1.35 equiv) and **S18** (12.3 mg, 15.7 µmol, 1.0 equiv) was added at rt a stock solution of DBU (2.63 µL, 17.4 µmol, 1.10 equiv) in MeCN (0.3 mL, 57.5 mm). After stirring the reaction mixture for 15 min at rt, a solution of **95** (6.7 mg, 19.2 µmol, 1.22 equiv) in THF (0.9 mL) was added dropwise at 0 °C. After stirring for 2 h at rt, more LiCl (1.0 mg, 23.6 µmol, 1.5 equiv) and DBU solution (0.21 mL, 57.5 mm in MeCN, 12.1 µmol, 0.77 equiv) were added. Stirring was continued for 1 h until TLC indicated complete conversion of the phosphonate. The yellowish reaction mixture was concentrated under reduced pressure and the residue was purified by column chromatography (hexane/EtOAc, 7:1) to give **89** (12.3 mg, 78%) as a colorless oil.

$\mathbf{R}_f$ = 0.37 (hexane/EtOAc, 7:1).

$[\alpha]_D^{24}$ = +32.3° [*c* = 0.615, $CHCl_3$].

^{1}**H-NMR** (400.1 MHz, $CDCl_3$): δ = 7.26 (d, *J* = 8.7 Hz, 2H), 6.87 (d, *J* = 8.7 Hz, 2H), 6.74 (m_c, 1H), 6.16 (s, 1H), 6.11 (dd, *J* = 10.8, 15.0 Hz, 1H), 5.92-5.74 (m, 3H), 5.70 (d, *J* = 15.5 Hz, 1H), 5.59 (m_c, 1H), 5.09-4.91 (m, 4H), 4.77 (ddd, *J* = 1.6, 4.0, 9.8 Hz, 1H), 4.56 (d, *J* = 11.0 Hz, 1H), 4.37 (d, *J* = 11.0 Hz, 1H), 4.11 (t, *J* = 6.6 Hz, 1H), 3.80 (s, 3H), 3.63 (s, 3H), 3.45-3.31 (m, 2H), 3.16 (s, 3H), 2.57 (m_c, 1H), 2.34-2.17 (m, 4H), 2.17-1.98 (m, 6H), 1.85-1.74 (m, 1H), 1.83 (d, *J* = 0.9 Hz, 3H), 1.73-1.63 (m, 1H), 1.67 (s, 3H), 1.53-1.34 (m, 4H), 1.07-0.96 (m, 24H), 0.90 (d, *J* = 6.9 Hz, 3H).

^{13}**C-NMR** (100.6 MHz, $CDCl_3$): δ = 172.9, 165.3, 159.1, 146.5, 145.8, 140.1, 138.7, 137.2, 134.1, 130.6, 129.4, 126.2, 125.9, 123.9, 114.8, 114.6, 113.8, 87.6, 80.0, 78.9, 76.5, 72.3, 70.7, 56.7, 55.3, 51.4, 39.9, 39.8, 38.7, 35.9, 35.8, 34.9, 33.2, 32.4, 31.4, 28.6, 19.7, 18.2, 18.1, 15.3, 12.5, 11.5, 9.7.

IR (neat, ν/cm^{-1}): 2940s, 2866m, 1738s, 1718s, 1653w, 1613w, 1513m, 1462m, 1379w, 1249s, 1172m, 1088s, 1058m, 1011w, 965w, 913m, 883m, 683m.

HRMS (ESI): *m/z* calcd for $C_{51}H_{85}INO_8Si$ $[M + NH_4]^+$: 994.5084, found: 994.5097.

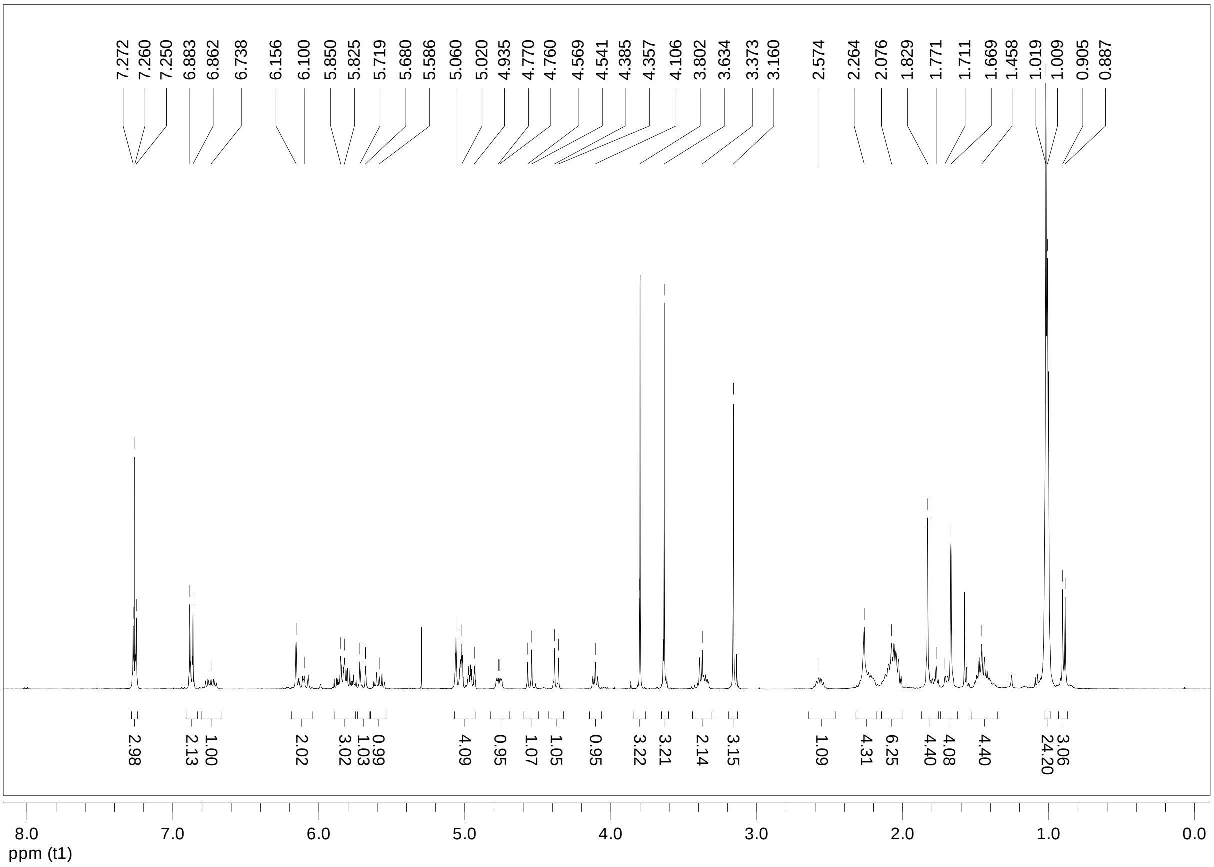

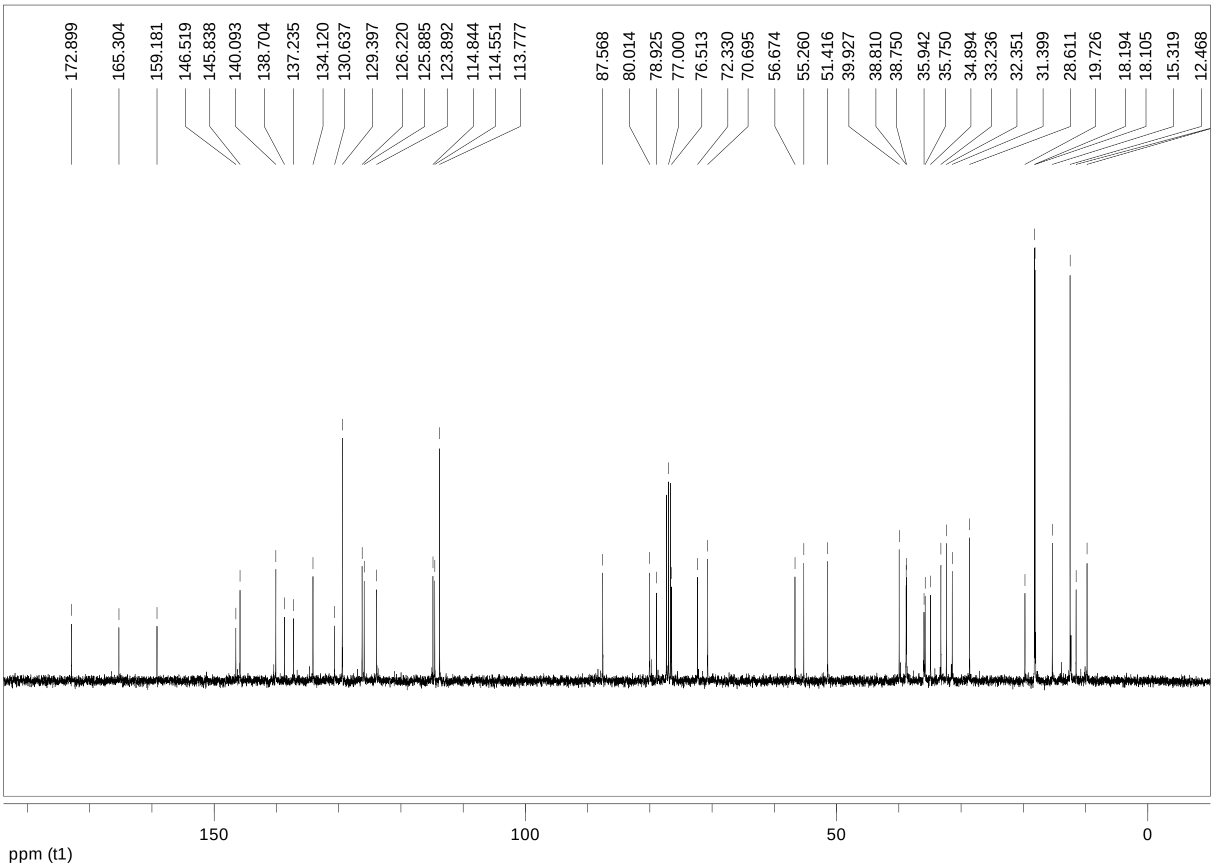

Methyl (*E*)-2-methylhex-2-en-4-ynoate (97):

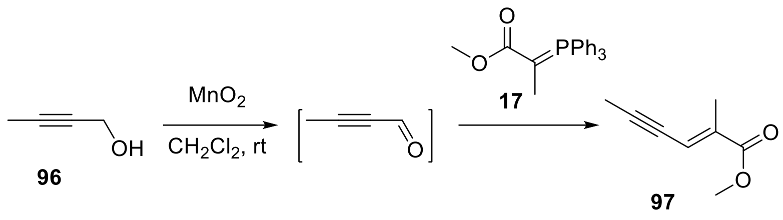

Oxidation: To a solution of 2-butyn-1-ol (**96**) (3.89 mL, 51.6 mmol, 1.20 eq) in CH_2Cl_2 (160 mL) were added molecular sieves (3 Å, beads) and activated MnO_2 (89.8 g, 1.03 mol, 24.0 eq) at 0 °C. The cooling bath was removed and the suspension was stirred for 1.5 h at rt.

Wittig reaction: A solution of ylide **17** (15.0 g, 43.1 mmol, 1.00 eq) was added at rt to the black suspension obtained in the oxidation. Then the mixture was heated to reflux (50 °C oil bath) for 1 h 45 min and then filtered through a pad of celite (topped with a few mm of silica). The pad was rinsed thoroughly with ether, the combined filtrates were concentrated under reduced pressure and the residue was purified by column chromatography (pent/Et_2O 15:1→10:1→5:1) to afford ester **97** as a colourless liquid (2.71 g, 45%).

TLC (hexane/EtOAc, 5:1): R_f = 0.52
^{1}H-NMR ($CDCl_3$, 400.1 MHz): δ = 6.60 (m_c, 1H), 3.75 (s, 3H), 2.07 (m_c, 3H), 2.03 (dd, *J* = 0.6 Hz, *J* = 1.2 Hz, 3H)
^{13}C-NMR ($CDCl_3$, 100.6 MHz): δ = 167.8, 137.6, 120.5, 99.2, 76.6, 52.0, 15.1, 4.9.
IR (neat, ν/cm^{-1}) 2994, 2952, 2920, 2849, 2367, 2357, 2221, 1712 (s), 1618, 1435, 1386, 1348, 1337, 1256 (s), 1191, 1175, 1118 (s), 1017, 974, 944, 891, 827, 802, 745 (s), 690, 512, 481, 439, 425, 409.
HRMS (EI): *m/z* calcd for $C_8H_{10}O_2$ [M]$^+$: 138.0676; found: 138.0676.

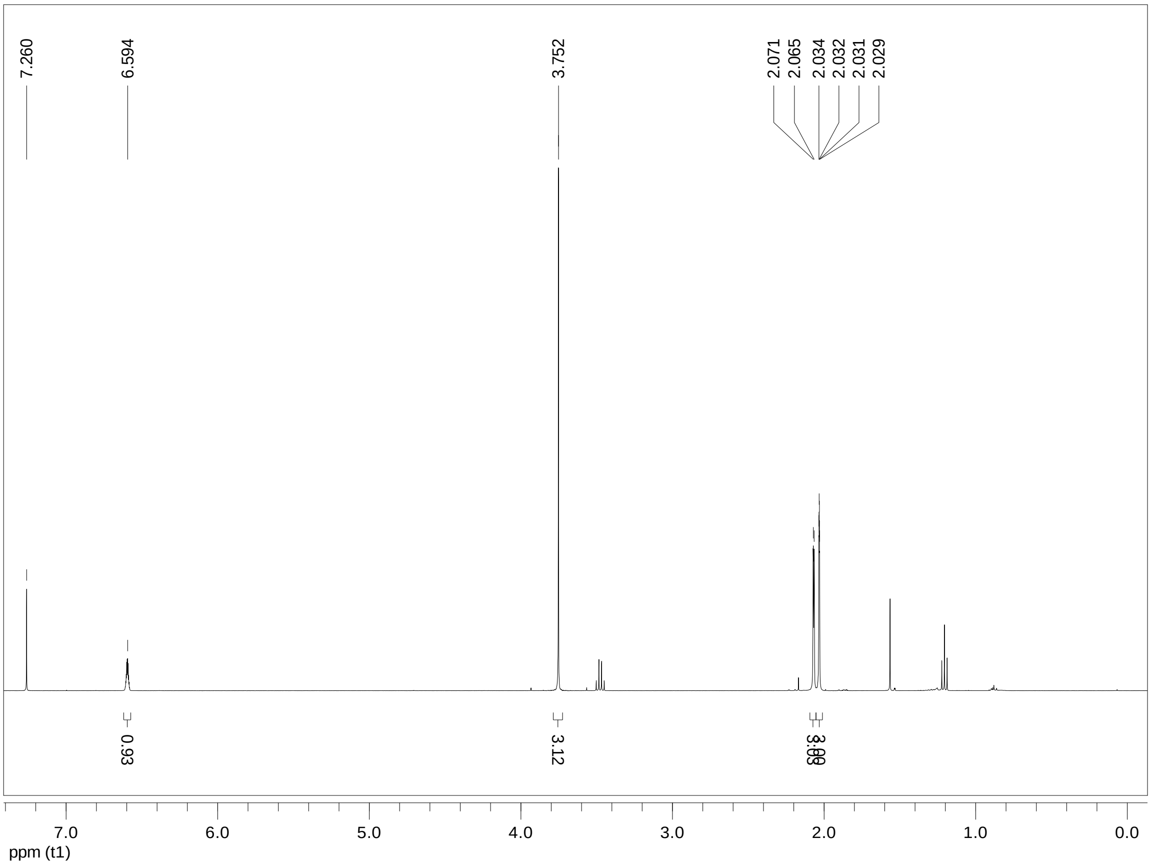
7.260
6.594
3.752
2.071
2.065
2.034
2.032
2.031
2.029
0.93
3.12
7.0
6.0
5.0
4.0
3.0
2.0
1.0
0.0
ppm (t1)

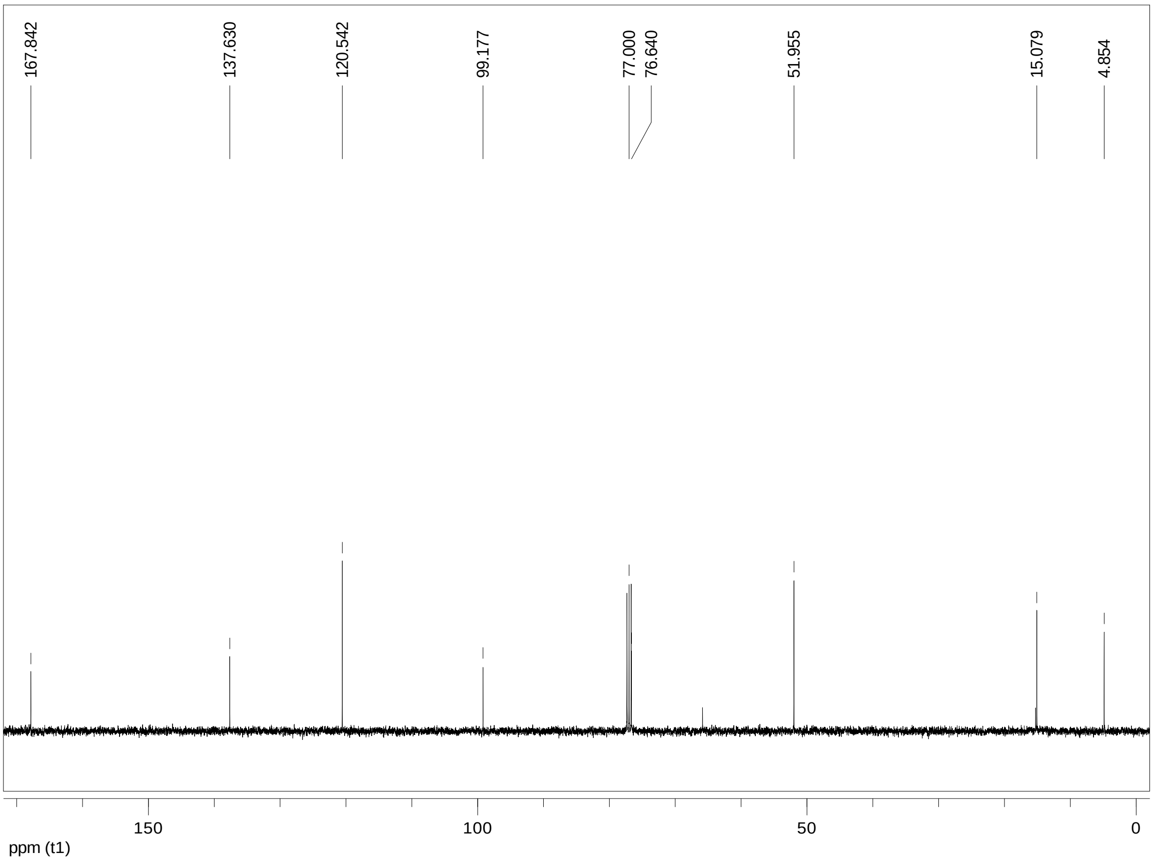
167.842
137.630
120.542
99.177
77.000
76.640
51.955
15.079
4.854
150
100
50
0
ppm (t1)

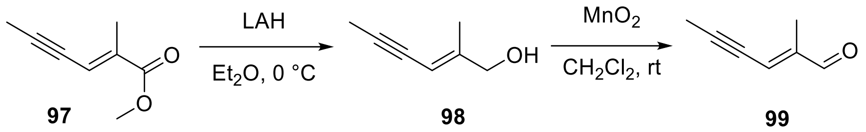

(*E*)-2-methylhex-2-en-4-ynal (99):

Reduction: To a solution of ester **97** (2.71 g, 19.6 mmol, 1.00 eq) in ether (230 mL) was added $LiAlH_4$ (774 mg, 20.4 mmol, 1.04 eq) in small portions at 0 °C. The suspension was stirred at the same temperature for 30 min and the reaction was then quenched by successive addition of H_2O (30 mL), 10% aq NaOH (30 mL), H_2O (90 mL). The layers were separated and the aqueous phase was extracted with ether (3×50 mL). The combined organic layers were washed with brine, dried over $MgSO_4$ and concentrated under reduced pressure. The crude allylic alcohol **98** thus obtained was used for the next step without purification.

Oxidation: To a solution of the crude allylic alcohol **98** (ca. 2.16 g, 19.6 mmol, 1.00 eq) in CH_2Cl_2 (68 mL) were added molecular sieves (3 Å, beads) and activated MnO_2 (34.1 g, 392 mmol, 20.0 eq) at rt. The suspension was stirred for 10 min at the same temperature and then filtered through a pad of Celite, topped with a few mm of silica. The filter was washed thoroughly with Et_2O, the combined filtrates were concentrated under reduced pressure and the residue was purified by column chromatography (Pent/Et_2O 5:1) to afford aldehyde **99** (1.84 g 95% wt/wt along with Et_2O, 1.75 g, 82%) as a pale yellow liquid.

TLC (hexane/EtOAc, 5:1): R_f = 0.53
^{1}H-NMR (400.1 MHz, $CDCl_3$): δ = 9.44 (s, 1H), 6.32 (m_c, 1H), 2.14 (dd, *J* = 0.5 Hz, *J* = 2.4 Hz, 3H), 1.91 (dd, *J* = 0.5 Hz, *J* = 1.2 Hz, 3H).
^{13}C{1H}-NMR (CDCl3, 100.6 MHz): δ = 194.4, 147.7, 130.2, 105.1, 76.3, 11.5, 5.2.
HRMS: *m/z* calcd for C_7H_9O $[M+H]^+$: 109.0648; found: 109.0653.

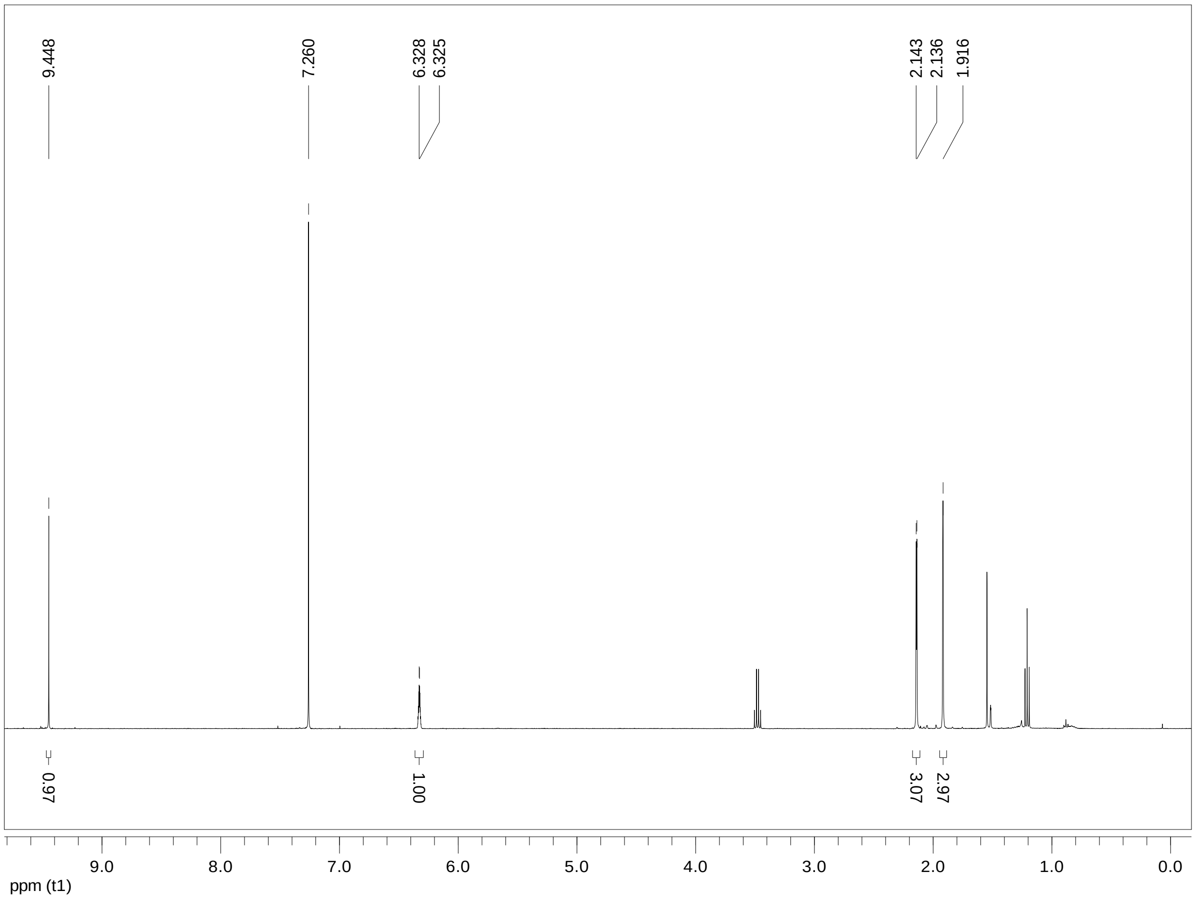
9.448
7.260
6.328
6.325
2.143
2.136
1.916
0.97
1.00
3.07
2.97
9.0
8.0
7.0
6.0
5.0
4.0
3.0
2.0
1.0
0.0
ppm (t1)

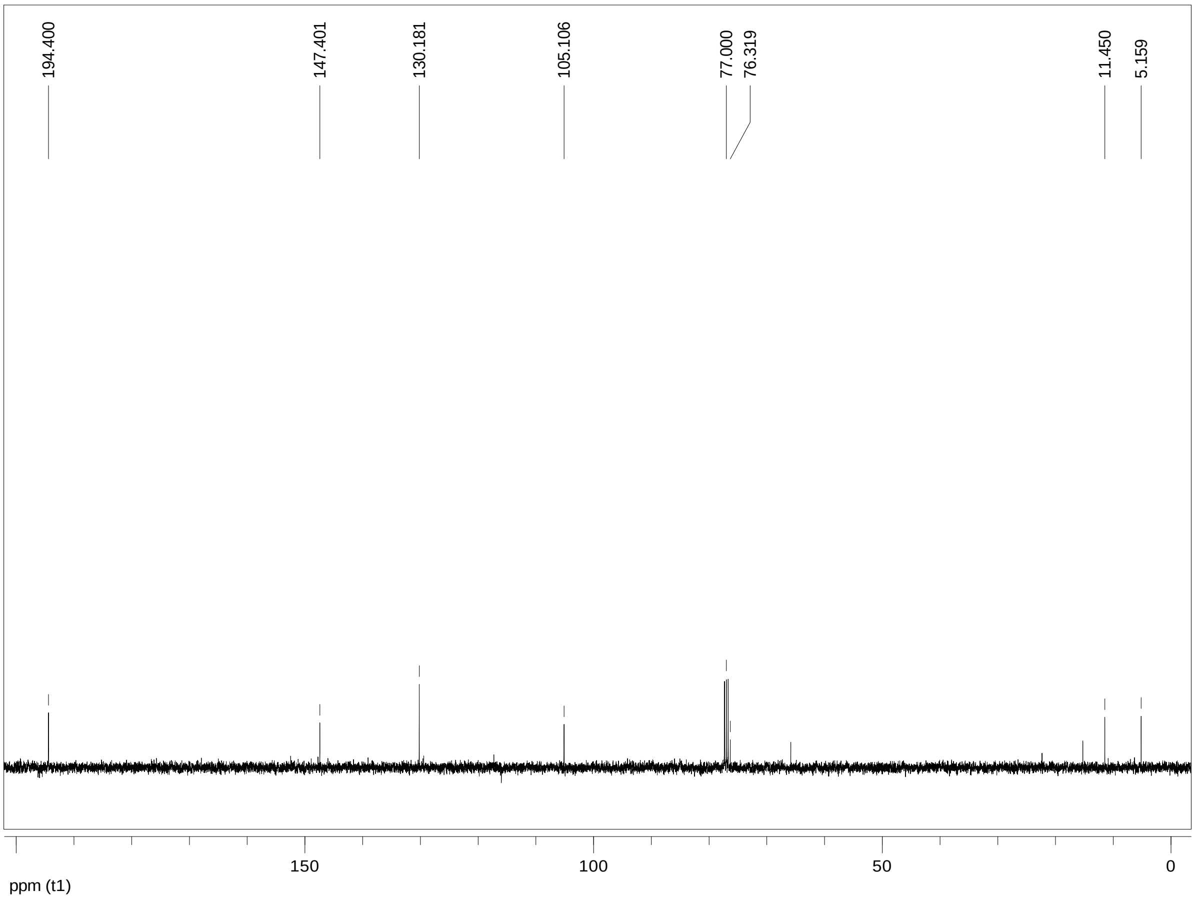
194.400
147.401
130.181
105.106
77.000
76.319
11.450
5.159
150
100
50
0
ppm (t1)

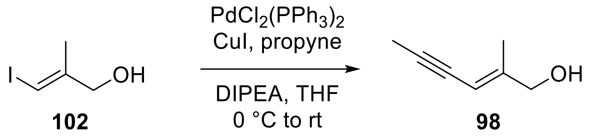

(*E*)-2-Methylhex-2-en-4-yn-1-ol (98): Propyne was condensed into a *Schlenk* tube at −78 °C, then DIPEA (17.5 mL, 20 eq) and CuI (235 mg, 1.01 mmol, 20 mol%) were added. To this suspension was added a solution of the vinyl iodide **102** (1.00 g, 5.05 mmol, 1.00 eq) in THF (1 mL, rinsed with 2×1 mL), followed by THF (7 mL) and the $PdCl_2(PPh_3)_2$ (532 mg, 0.758 mol, 15 mol%) at −78 °C. After 15 min stirring at −78 °C, the reaction was allowed to warm to rt and stirred overnight. Reaction control by HPLC indicated full conversion. The reaction was diluted with ether (20 mL) and washed with acetic acid (3 M, 35 mL). Then the layers were separated and the aqueous phase was extracted with ether (2×20 mL). The combined organic extracts were washed with $NaHCO_3$, dried over $MgSO_4$ and concentrated under reduced pressure. The crude was then purified by column chromatography (pent:Et_2O 1:1) to afford the desired product **98** (662 mg) as an orange oil. This was further purified by Kugelrohr distillation (90-100 °C at 15-8 mbar) to afford **98** as a yellow oil (350 mg, 63%).

^{1}H-NMR ($CDCl_3$, 400.1 MHz): δ = 5.51 (m_c, 1H), 4.09 (bs, 2H), 1.99 (d, *J* = 2.0 Hz, 3H), 1.87 (bs, 3H).

^{13}C-NMR ($CDCl_3$, 100.6 MHz): δ = 148.2, 105.4, 89.7, 76.7, 67.0, 16.2, 4.4.

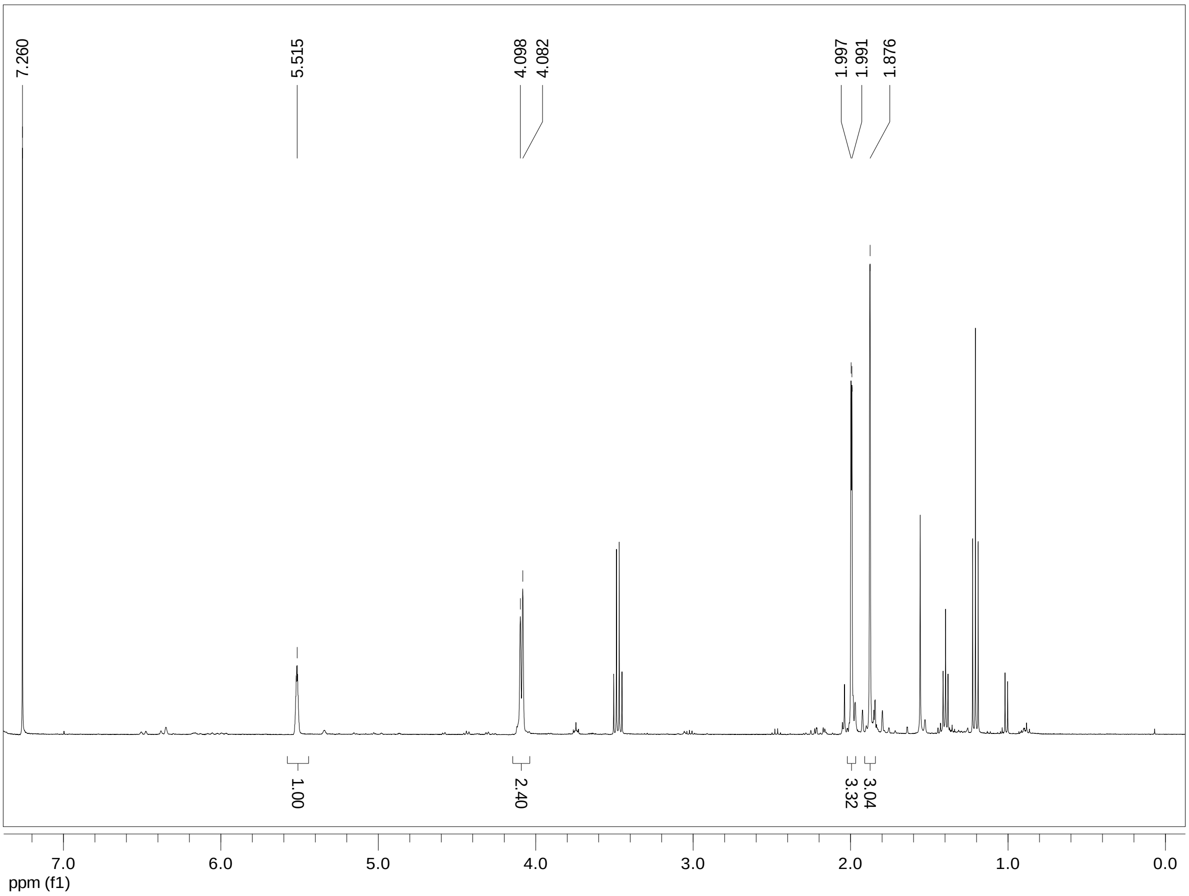
7.260
5.515
4.098
4.082
1.997
1.991
1.876
1.00
2.40
3.32
3.04
7.0
6.0
5.0
4.0
3.0
2.0
1.0
0.0
ppm (f1)

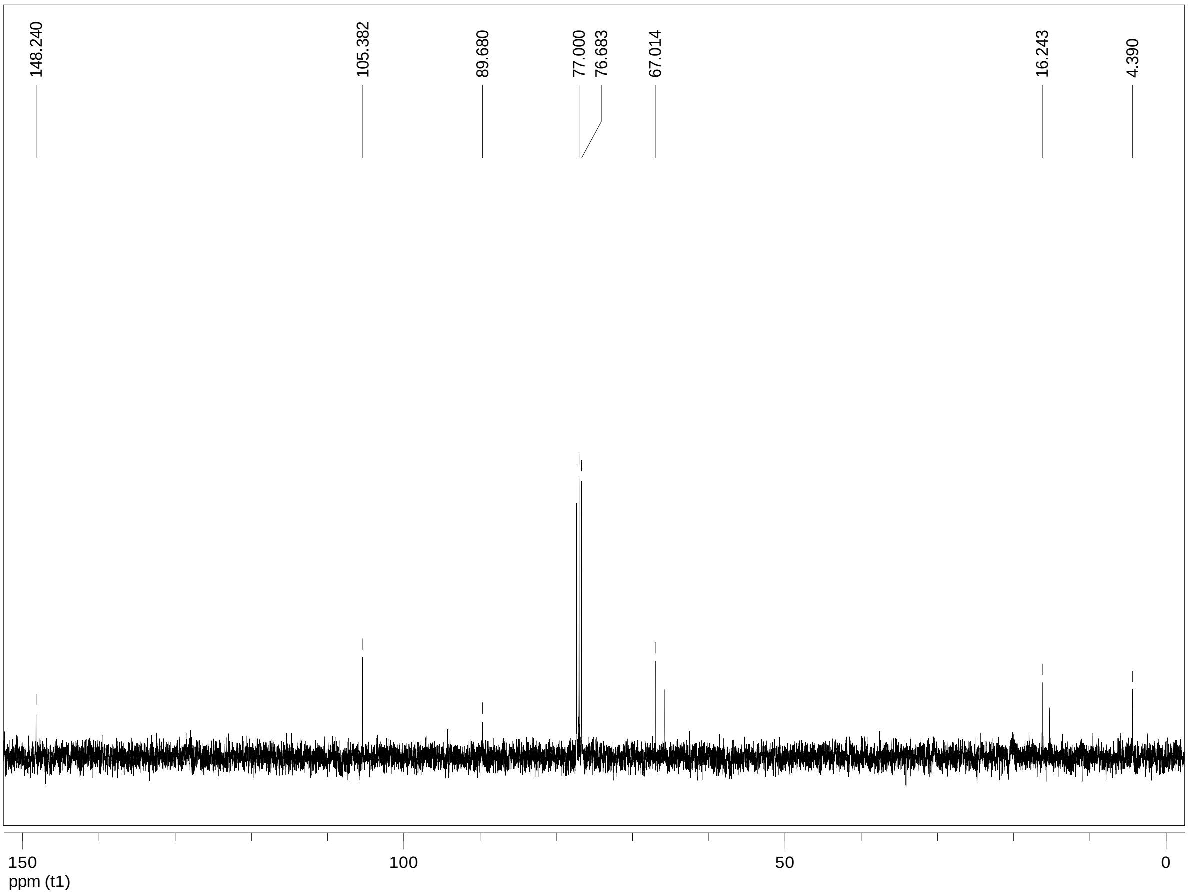
148.240
105.382
89.680
77.000
76.683
67.014
16.243
4.390
150
100
50
0
ppm (t1)

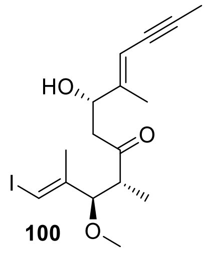

100

(1*E*,3*R*,4*R*,7*S*,8*E*)-7-Hydroxy-1-iodo-3-methoxy-2,4,8-trimethyldodeca-1,8-dien-10-yn-5-one (100): A solution of (+)-DIP-Cl (342 mg, 1.07 mmol, 2.00 eq) in CH_2Cl_2 (0.4 mL) was cooled to −78 °C. Then NEt_3 was added (0.177 mL, 1.28 mmol, 2.40 eq), followed by a solution of ketone **27** (150 mg, 0.532 mmol, 1.00 eq) in CH_2Cl_2 (0.2 mL, rinsed with 2×0.2 mL). After a few min a white precipitate formed, which rendered the mixture unstirrable. After 1.5 h the cooling bath was removed and, a few minutes later, replaced by an ice bath. This led to a thick suspension, which was easily stirrable. After 5 min at 0 °C, the mixture was recooled to −78 °C. After a total of 3 h 30 min, a solution of aldehyde **99** (115 mg, 1.06 mmol, 2.00 eq) in CH_2Cl_2 (0.2 mL, rinsed with 2×0.2 mL) was added. The orange mixture was stirred for another 50 min at −78 °C, and was then stored in a freezer (−18 °C) for 17 h. The reaction was then quenched with pH 7 phosphate buffer and the layers were separated. The aqueous phase was diluted with phosphate buffer and extracted with CH_2Cl_2 (3×10 mL). The combined extracts were concentrated under reduced pressure. The residue was dissolved in MeOH (3 mL) and pH 7 phosphate buffer (0.6 mL) was added. The mixture was cooled to 0 °C and H_2O_2 (30%, 0.75 mL) was added. The mixture was stirred at 0 °C for 5 min and at rt for 50 min. It was then poured into water (40 mL), CH_2Cl_2 was added, the layers were separated, and the aqueous phase was extracted with CH_2Cl_2 (3×10 mL). The combined organic layers were washed with brine, dried over Na_2SO_4 and concentrated under reduced pressure. The residue was purified by column chromatography (hexane/EtOAc 5:1) to afford **100** (352 mg, 43% wt/wt along with isopinocampheol/Et_2O/EtOAc, 151 mg, 73%, dr, nd) as a yellow oil which crystallized in a freezer (−18 °C).

TLC (hexane/EtOAc, 5:1): R_f = 0.17

^{1}H-NMR (400.1 MHz, $CDCl_3$): δ = 6.22 (m_c, 1H), 5.57 (m_c, 1H), 4.47 (m_c, 1H), 3.83 (d, *J* = 7.3 Hz, 1H), 3.21 (s, 3H), 2.94 (d, *J* = 2.6 Hz, 1H), 2.75 (qd, *J* = 7.0 Hz, *J* = 7.0 Hz, 1H),

2.68-2.51 (m, 2H), 1.99 (d, J = 2.0 Hz, 3H), 1.85 (bs, 3H), 1.75 (d, J = 1.1 Hz, 3H), 1.11 (d, J = 6.9 Hz, 3H).

^{13}C-NMR (100.6 MHz, $CDCl_3$): δ = 212.5, 148.8, 145.3, 106.3, 90.4, 86.3, 80.6, 76.6, 71.0, 57.0, 50.1, 47.6, 19.9, 15.4, 12.1, 4.4.

HRMS: *m/z* calcd for $C_{16}H_{23}INaO_3$ $[M+Na]^+$: 413.0584; found: 413.0587.

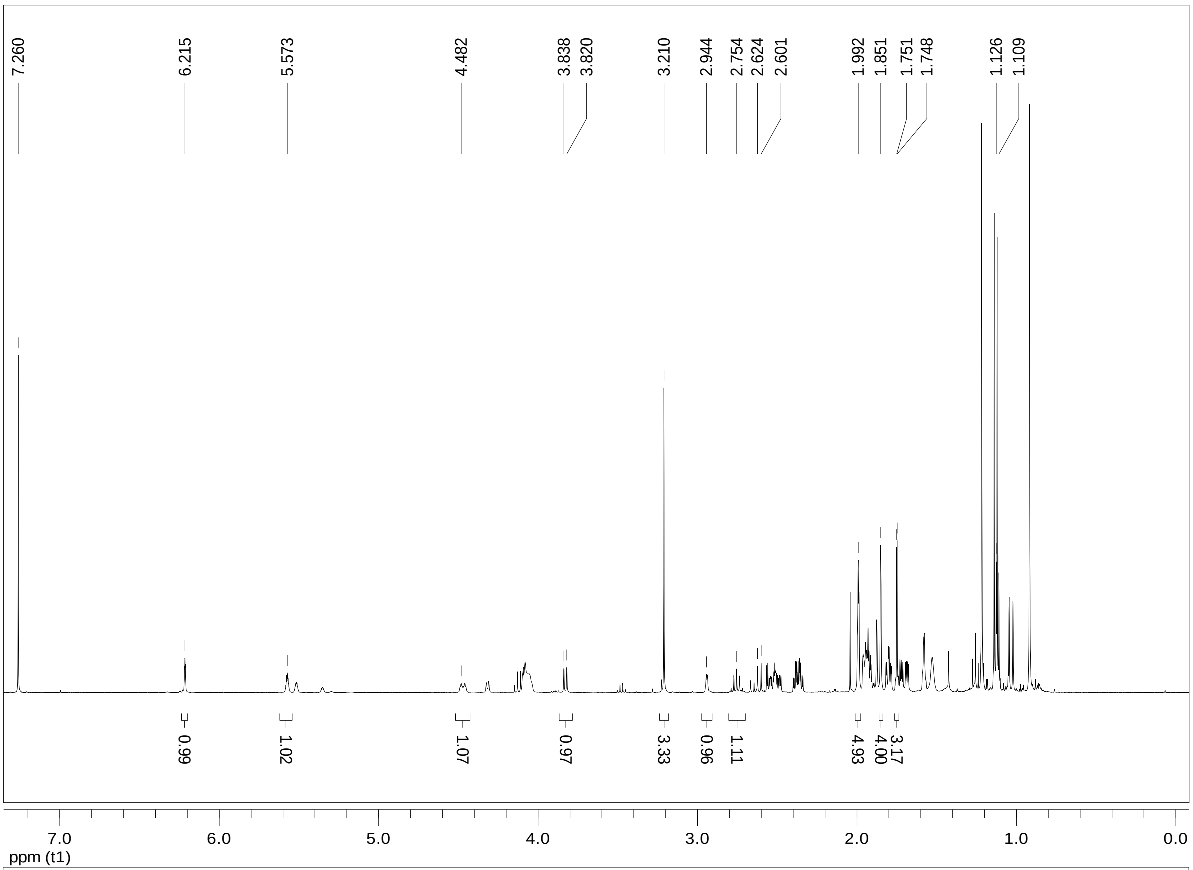
7.260
6.215
5.573
4.482
3.838
3.820
3.210
2.944
2.754
2.624
2.601
1.992
1.851
1.751
1.748
1.126
1.109
0.99
1.02
1.07
0.97
3.33
0.96
1.11
4.93
4.00
3.17
7.0
6.0
5.0
4.0
3.0
2.0
1.0
0.0
ppm (t1)

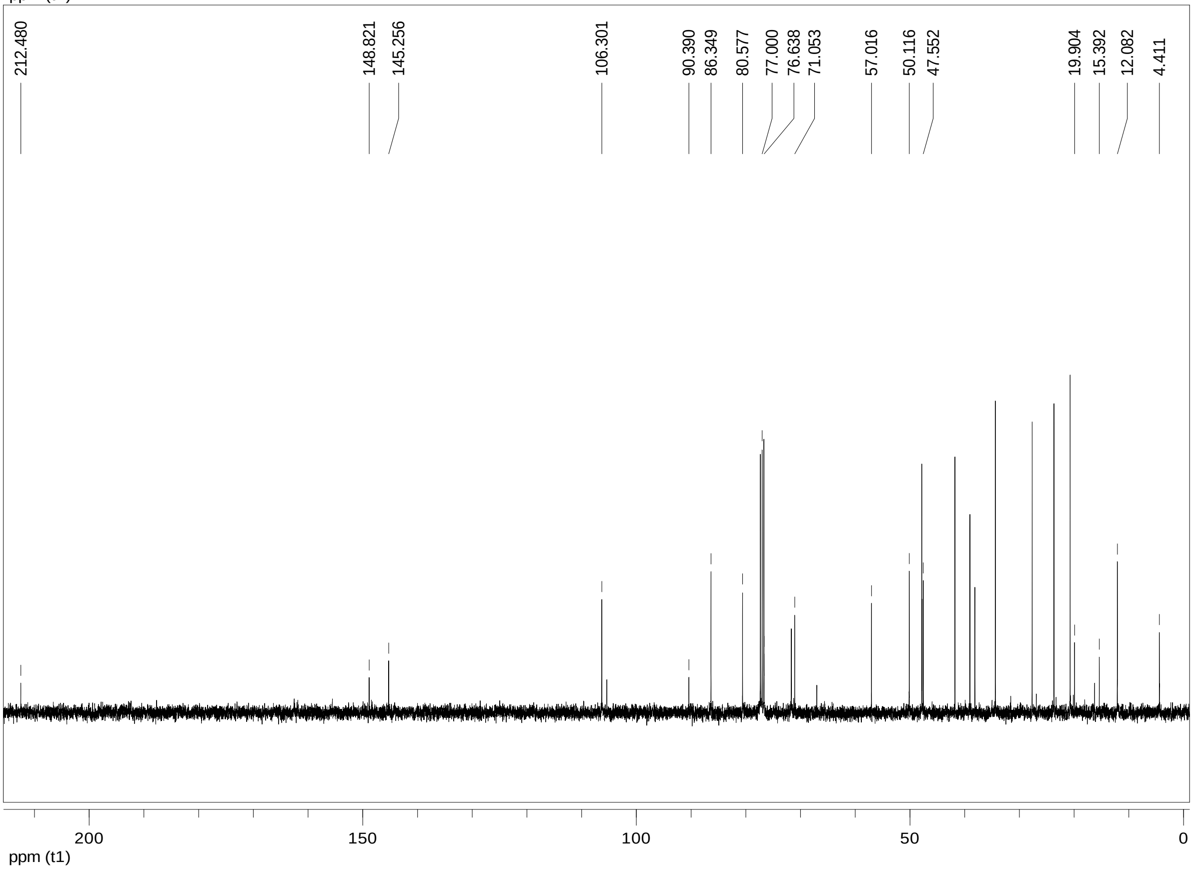
212.480
148.821
145.256
106.301
90.390
86.349
80.577
77.000
76.638
71.053
57.016
50.116
47.552
19.904
15.392
12.082
4.411
200
150
100
50
0
ppm (t1)

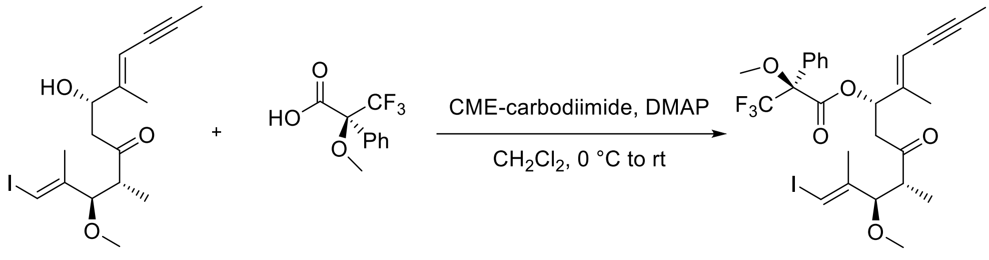

100 **S28**

***R*-Mosher ester *S28*:**

Purification of the starting material: Isopinocampheol was removed from **100** at a rotavap (40 °C, 10^{-2} mbar). Subsequent column chromatography (hexane/EtOAc 5:1) delivered **100** as a colourless oil.

To a cooled (0 °C) solution of (S)-3,3,3-trifluoro-2-methoxy-2-phenylpropanoic acid (43.2 mg, 0.185 mmol, 1.50 eq) in CH_2Cl_2 (1.6 mL) over molecular sieves (beads, 3 Å) was added CME-carbodiimide (93.8 mg, 0.221 mmol, 1.80 eq). The mixture was stirred at 0 °C for 15 min, then a solution of the alcohol **100** (48.0 mg, 0.123 mmol, 1.00 eq) in CH_2Cl_2 (1.4 mL) was added. The cooling bath was removed and DMAP (1.5 mg, 10 mol%) was added. As the conversion was incomplete after 1.5 h, the mixture was stirred at rt overnight. This did apparently not lead to any further conversion. Thus the suspension was directly applied on a silica gel column and purified (hexane/EtOAc 10:1) to afford ester **S28** as a colourless oil containing inseparable and unidentified impurities (21.6 mg, 29%). Furthermore some starting material was recovered (26.9 mg, 56%).

TLC (hexane/EtOAc, 5:1): R_f = 0.39
$[\alpha]_D^{24}$ = −2.84 (*c* = 1.08, $CHCl_3$)
^{1}H-NMR (400.1 MHz, $CDCl_3$): δ = 7.49-7.43 (m, 2H), 7.43-7.34 (m, 3H), 6.18 (m_c, 1H), 5.89 (dd, *J* = 3.2 Hz, *J* = 9.4 Hz, 1H), 5.65 (m_c, 1H), 3.72 (d, *J* = 6.9 Hz, 1H), 3.50 (m_c, 3H), 3.14 (s, 3H), 2.97 (dd, *J* = 9.4 Hz, *J* = 18.0 Hz, 1H), 2.61 (qd, *J* = 7.0 Hz, *J* = 7.0 Hz, 1H), 2.47 (dd, *J* = 3.4 Hz, *J* = 18.0 Hz, 1H), 2.00 (d, *J* = 2.3 Hz, 3H), 1.87 (bs, 3H), 1.70 (d, *J* = 1.1 Hz, 3H), 0.95 (d, *J* = 6.9 Hz, 3H).
^{13}C-NMR (100.6 MHz, $CDCl_3$): δ = 206.8, 165.2, 145.0, 143.9, 132.3, 129.5, 128.3 (2C), 127.5 (2C), 110.7, 92.3, 85.9, 80.6, 76.0, 74.6, 56.9, 55.4, 49.6, 45.1, 20.0, 15.2, 11.7, 4.5 (quartetts of *C*CF_3 and C*C*F_3 not assigned).

IR (neat, ν/cm^{-1}): 2983, 2936, 2875, 2849, 1749s, 1719, 1452, 1377, 1270s, 1253s, 1168s, 1120, 1083, 1018s, 992, 965, 917, 866, 828, 765, 718s, 698, 643, 510, 493, 466, 418, 404.
HRMS Calcd. for $C_{26}H_{30}F_3INaO_5$ $[M+Na]^+$ *m/z* 629.0982. Found: 629.0980.

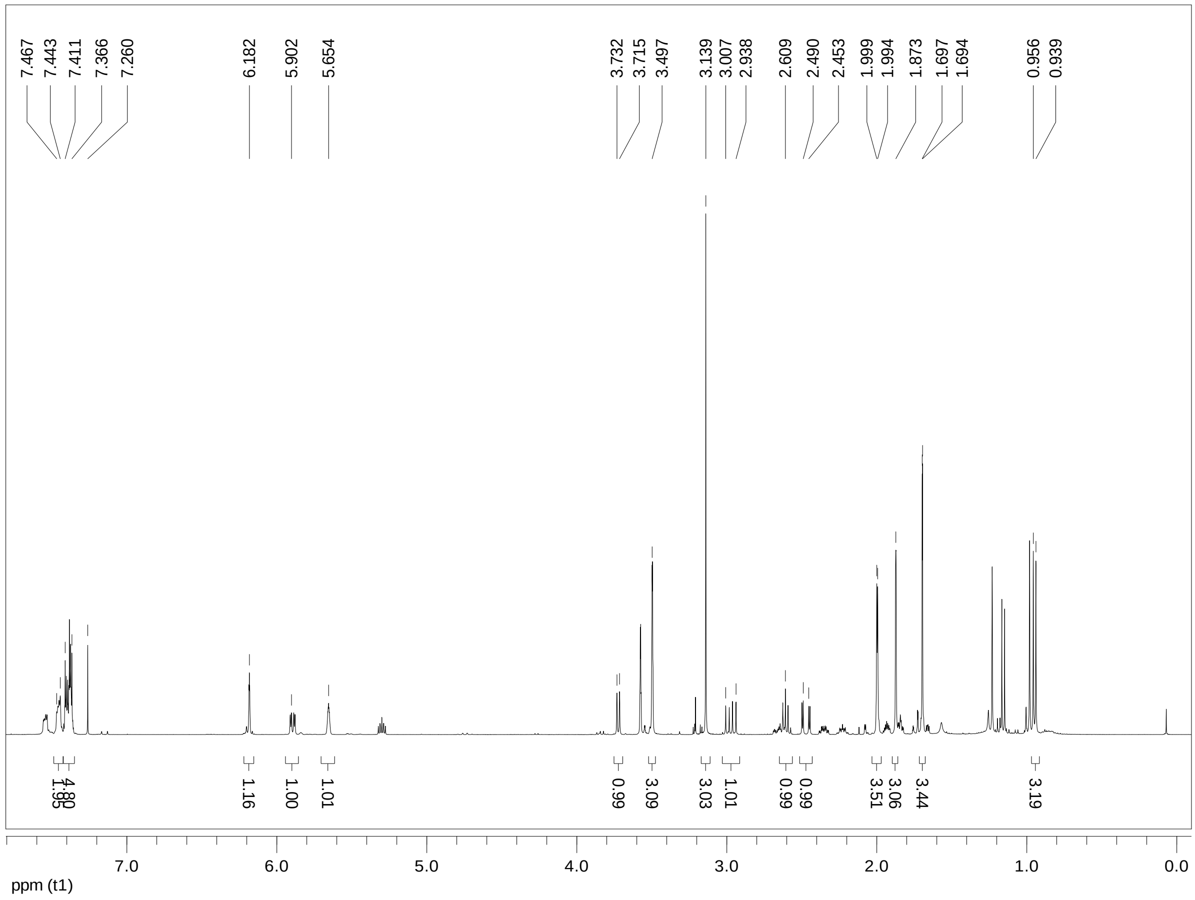
7.467
7.443
7.411
7.366
7.260
6.182
5.902
5.654
3.732
3.715
3.497
3.139
3.007
2.938
2.609
2.490
2.453
1.999
1.994
1.873
1.697
1.694
0.956
0.939
1.95
4.80
1.16
1.00
1.01
0.99
3.09
3.03
1.01
0.99
0.99
3.51
3.06
3.44
3.19
7.0
6.0
5.0
4.0
3.0
2.0
1.0
0.0
ppm (t1)

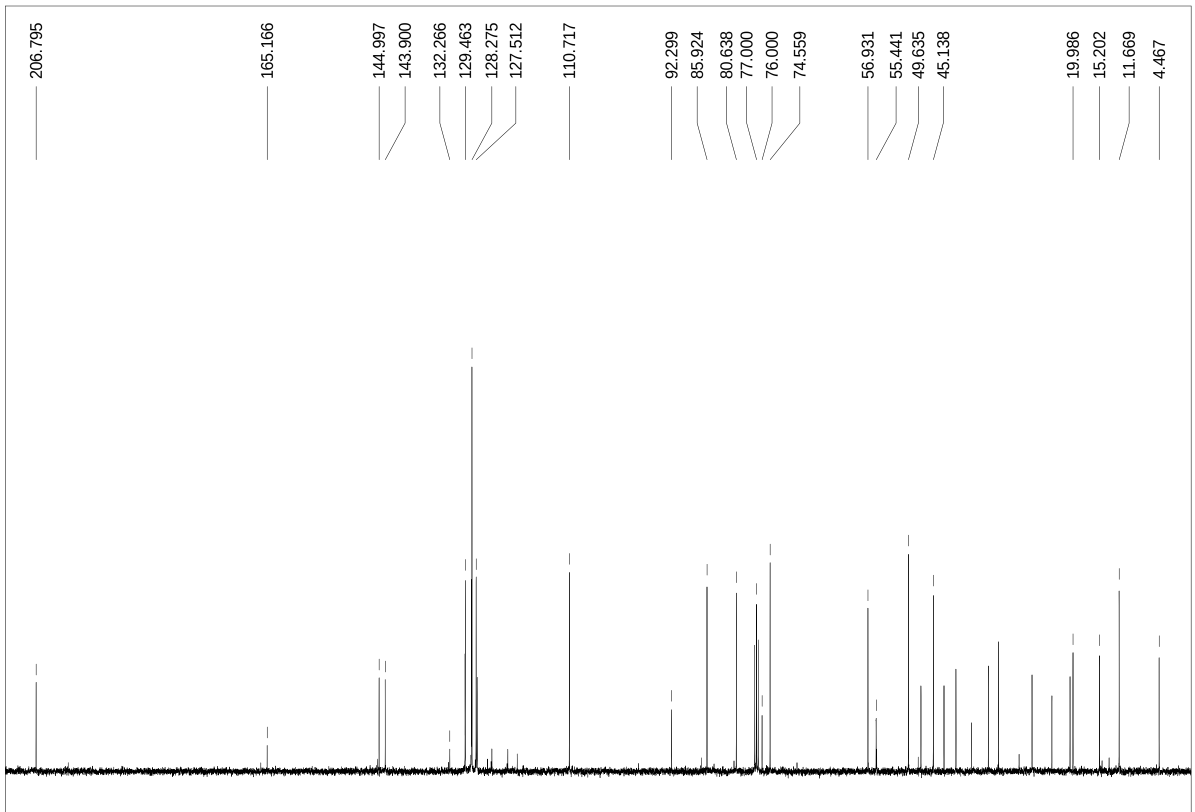
206.795
165.166
144.997
143.900
132.266
129.463
128.275
127.512
110.717
92.299
85.924
80.638
77.000
76.000
74.559
56.931
55.441
49.635
45.138
19.986
15.202
11.669
4.467
200
150
100
50
0
ppm (t1)

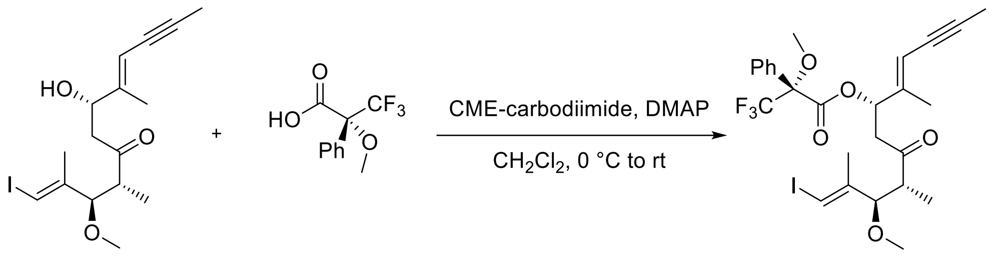

100 **S29**

***S*-Mosher ester S29:**

Purification of the starting material: Isopinocampheol was removed from **100** at a rotavap (40 °C, 10^{-2} mbar). Subsequent column chromatography (hexane/EtOAc 5:1) delivered **100** as a colourless oil.

To a cooled (0 °C) solution of (R)-3,3,3-trifluoro-2-methoxy-2-phenylpropanoic acid (43.2 mg, 0.185 mmol, 1.50 eq) in CH_2Cl_2 (1.6 mL) over molecular sieves (beads, 3 Å) was added CME-carbodiimide (93.8 mg, 0.221 mmol, 1.80 eq). The mixture was stirred at 0 °C for 15 min, then a solution of the alcohol **100** (48.0 mg, 0.123 mmol, 1.00 eq) in CH_2Cl_2 (1.4 mL) was added. The cooling bath was removed and DMAP (1.5 mg, 10 mol%) was added. As the conversion was incomplete after 1.5 h, the mixture was stirred at rt overnight and the suspension was directly applied onto a silica gel column and purified (hexane/EtOAc 10:1), which afford ester **S29** as a colourless oil containing inseparable and unidentified impurities (11.4 mg, 15%). Furthermore some starting material was recovered (27.1 mg, 56%).

TLC (hexane/EtOAc, 5:1): R_f = 0.39
[α]$_D^{24}$ = +54.7 (*c* = 0.57, $CHCl_3$)
^{1}H-NMR (400.1 MHz, $CDCl_3$): δ = 7.51-7.45 (m, 2H), 7.45-7.34 (m, 3H), 6.20 (m_c, 1H), 5.79 (dd, *J* = 2.6 Hz, *J* = 9.8 Hz, 1H), 5.51 (m_c, 1H), 3.81 (d, *J* = 7.0 Hz, 1H), 3.49 (m_c, 3H), 3.16 (s, 3H), 2.99 (dd, *J* = 9.9 Hz, *J* = 18.2 Hz, 1H), 2.68 (qd, *J* = 7.0 Hz, *J* = 7.0 Hz, 1H), 2.49 (dd, *J* = 2.8 Hz, *J* = 18.2 Hz, 1H), 1.99 (d, *J* = 2.2 Hz, 3H), 1.75 (bs, 3H), 1.72 (d, *J* = 1.1 Hz, 3H), 1.04 (d, *J* = 6.9 Hz, 3H).
^{13}C-NMR (100.6 MHz, $CDCl_3$): δ = 207.4, 165.3, 145.0, 144.0, 132.3, 129.5, 128.3 (2C), 127.4 (2C), 110.1, 92.0, 86.0, 80.7, 76.0, 74.6, 57.0, 55.5, 49.7, 45.3, 20.0, 15.1, 11.8, 4.5 (quartetts of *C*CF$_3$ and C*C*F$_3$ not assigned).

IR (neat, ν/cm^{-1}): 2982, 2935, 2920, 2878, 2851, 2828, 1751s, 1717s, 1588, 1452, 1377, 1271s, 1255s, 1169, 1121s, 1086s, 1016s, 991s, 965, 918, 826, 765, 721s, 698, 666, 532, 498, 473, 459, 428, 406.

HRMS Calcd. for $C_{26}H_{30}F_{3}INaO_{5}$ $[M+Na]^{+}$ *m/z* 629.0982. Found: 629.0975.

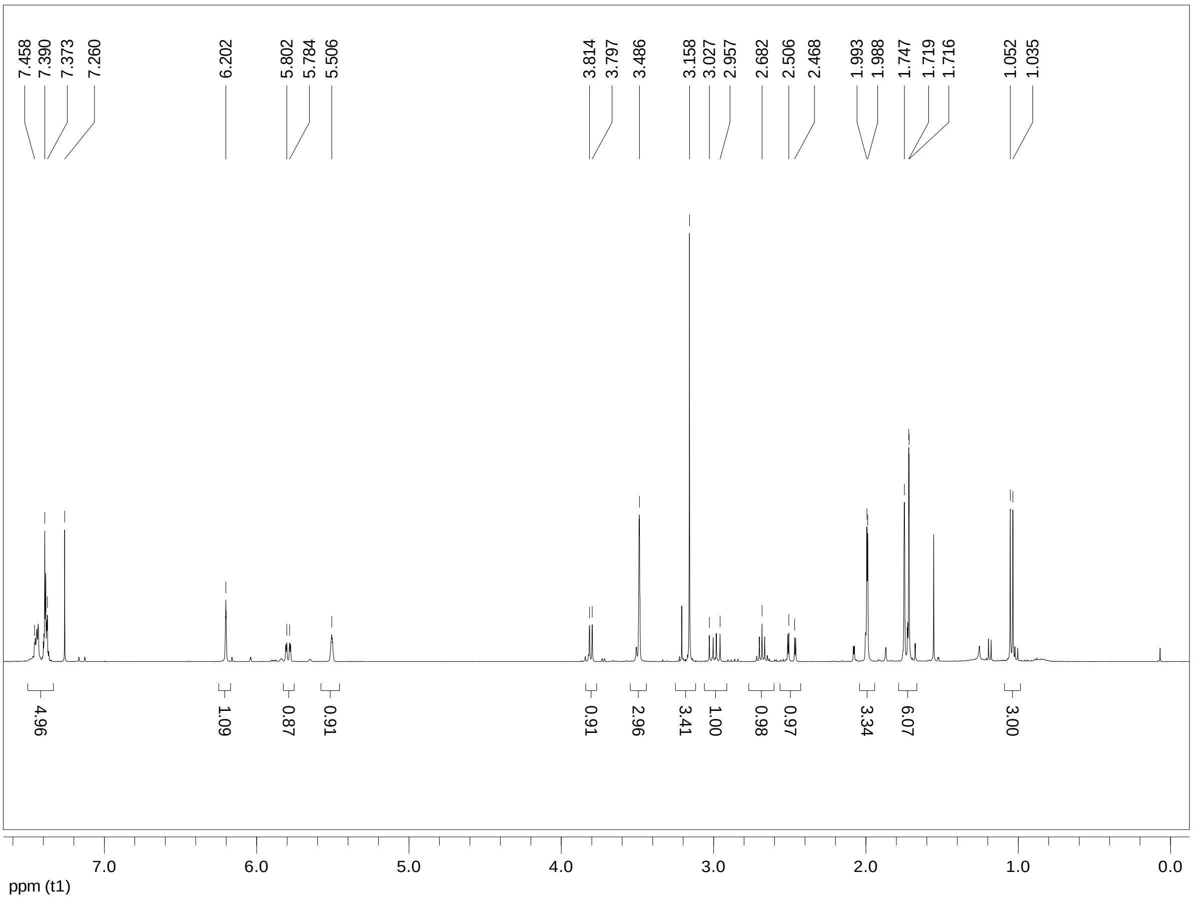

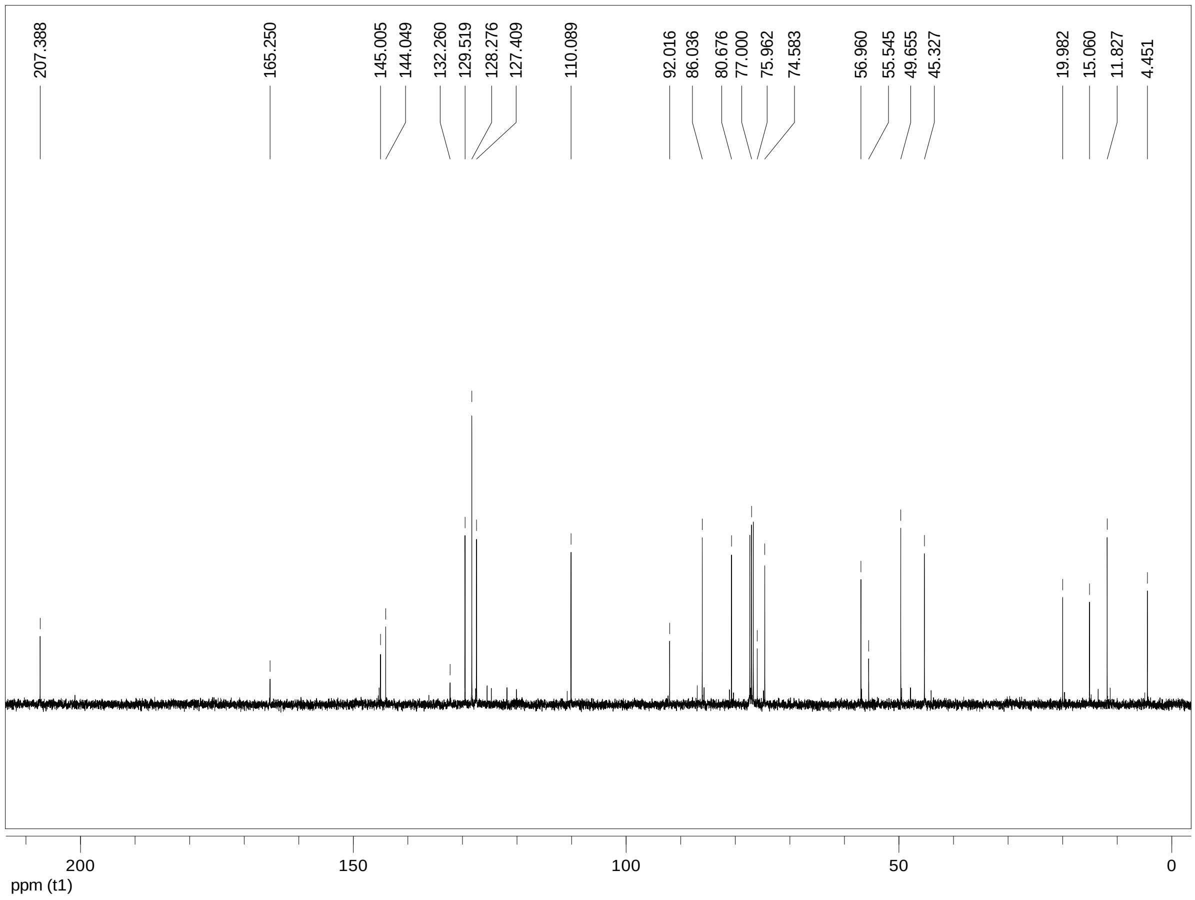
207.388
165.250
145.005
144.049
132.260
129.519
128.276
127.409
110.089
92.016
86.036
80.676
77.000
75.962
74.583
56.960
55.545
49.655
45.327
19.982
15.060
11.827
4.451
200
150
100
50
0
ppm (t1)

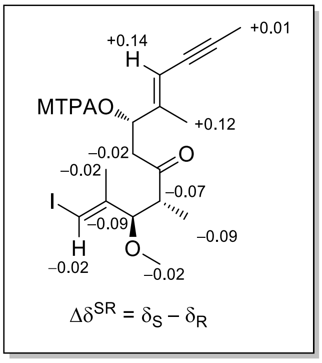
+0.14
H
+0.01
MTPAO
+0.12
−0.02
O
−0.02
−0.07
I
−0.09
H
−0.09
O
−0.02
−0.02
ΔδSR = δS − δR

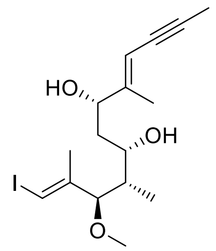

S30

(1*E*,3*R*,4*S*,5*S*,7*S*,8*E*)-1-Iodo-3-methoxy-2,4,8-trimethyldodeca-1,8-dien-10-yne-5,7-diol (*S30*): A solution of $(NMe_4)BH(OAc)_3$ (455 mg, ca. 4.65 eq) in MeCN (1.5 mL) and AcOH (1.5 mL) was cooled to −40 °C (MeCN/dry ice). To the frozen mixture was added a solution of ketone **100** (145 mg, 372 µmol, 1.00 eq) in MeCN (0.5 mL, rinsed with 2×0.4 mL). The resulting thick slurry was stirred for a few min at the same temperature and was then stored in a freezer (−18 °C) for 19.5 h. The suspension was allowed to warm to rt and stirred for another 1 h; *Rochelle* salt (aq. sat.) was then added, leading to the immediate formation of a white suspension. This mixture was stirred at rt for 1 h. Then it was diluted with water, some CH_2Cl_2 was added and the acetic acid was neutralized with $NaHCO_3$ (s). As soon as the gas evolution had stopped, the layers were separated and the aqueous phase was extracted with CH_2Cl_2 (3×10 mL). The combined organic layers were washed with brine, dried over Na_2SO_4 and concentrated under reduced pressure. The residue was purified by column chromatography (hexane/Et_2O 2:1→Hexane/EtOAc 1:1) to afford **S30** (143 mg, 91% wt/wt along with EtOAc, 131 mg, 90%, 1,3-*syn* isomer not detectable by NMR) as a yellow oil.

TLC (hexane/EtOAc, 1:1): R_f = 0.20

[α]$_D^{24}$ = +30.9 (*c* = 1.05, $CHCl_3$)

^{1}H-NMR (400.1 MHz, $CDCl_3$): δ = 6.18 (m_c, 1H), 5.63 (m_c, 1H), 4.40 (m_c, 1H), 3.95 (d, *J* = 3.6 Hz, 1H), 3.81 (m_c, 1H), 3.27 (s, 3H), 3.21 (d, *J* = 5.8 Hz, 1H), 2.88 (d, *J* = 4.7 Hz, 1H), 2.00 (d, *J* = 2.3 Hz, 3H), 1.85 (bs, 3H), 1.80-1.71 (m, 3H), 1.76 (d, *J* = 1.0 Hz, 3H), 0.84 (d, *J* = 7.1 Hz, 3H).

^{13}C-NMR (100.6 MHz, $CDCl_3$): δ = 150.8, 145.1, 105.5, 90.1, 86.6, 78.3, 76.7, 73.3, 71.5, 57.3, 40.7, 38.6, 21.8, 15.8, 11.1, 4.4.

IR (neat, ν/cm^{-1}): 3408br, 2976, 2936, 2914, 2851, 2829, 2365, 2353, 2342, 2331, 171738, 1619, 1441, 1374, 1242s, 1193, 1112, 1088s, 1046s, 1008, 957, 936, 848, 810, 785, 680, 669, 636, 603, 532, 513, 501, 489, 475.

HRMS: *m/z* calcd for $C_{16}H_{25}INaO_3$ [M+Na]$^+$: 415.0741; found: 415.0740.

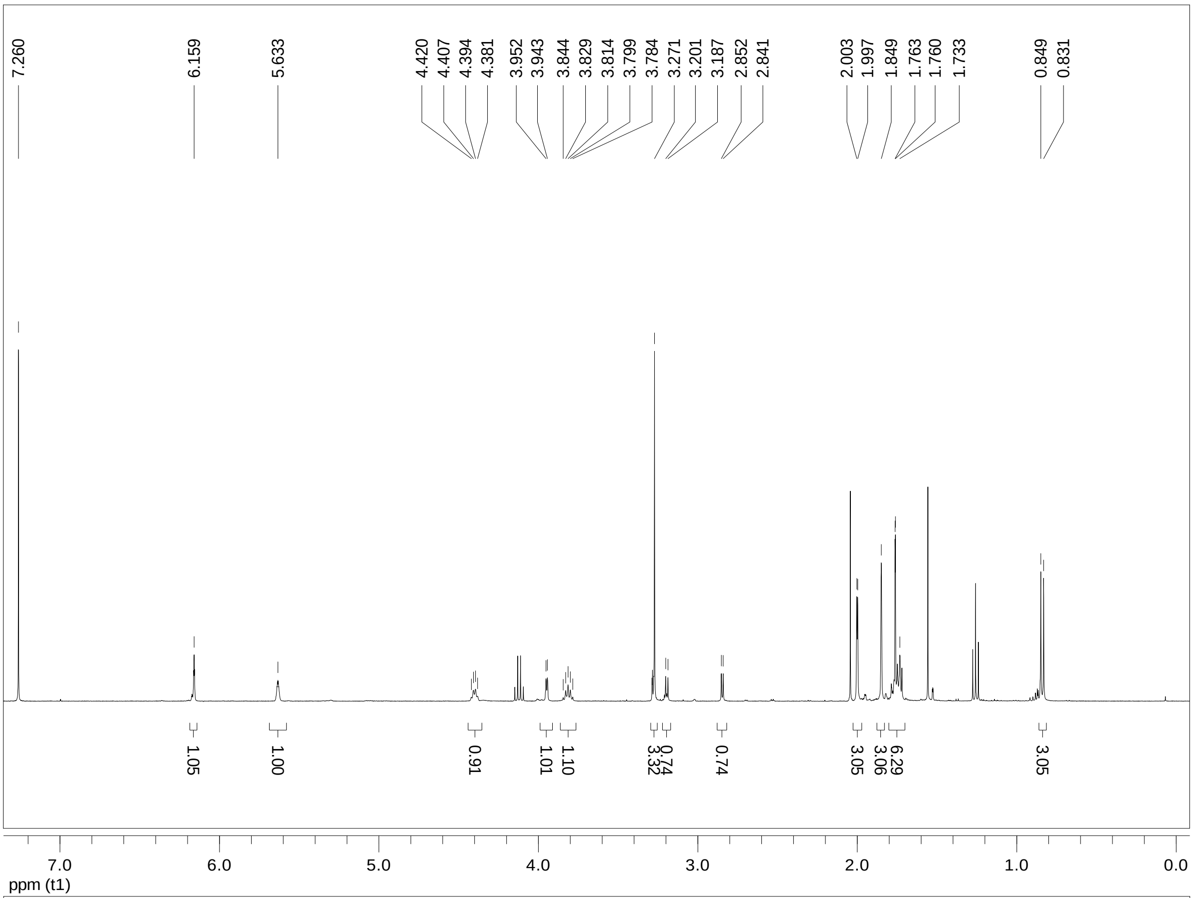

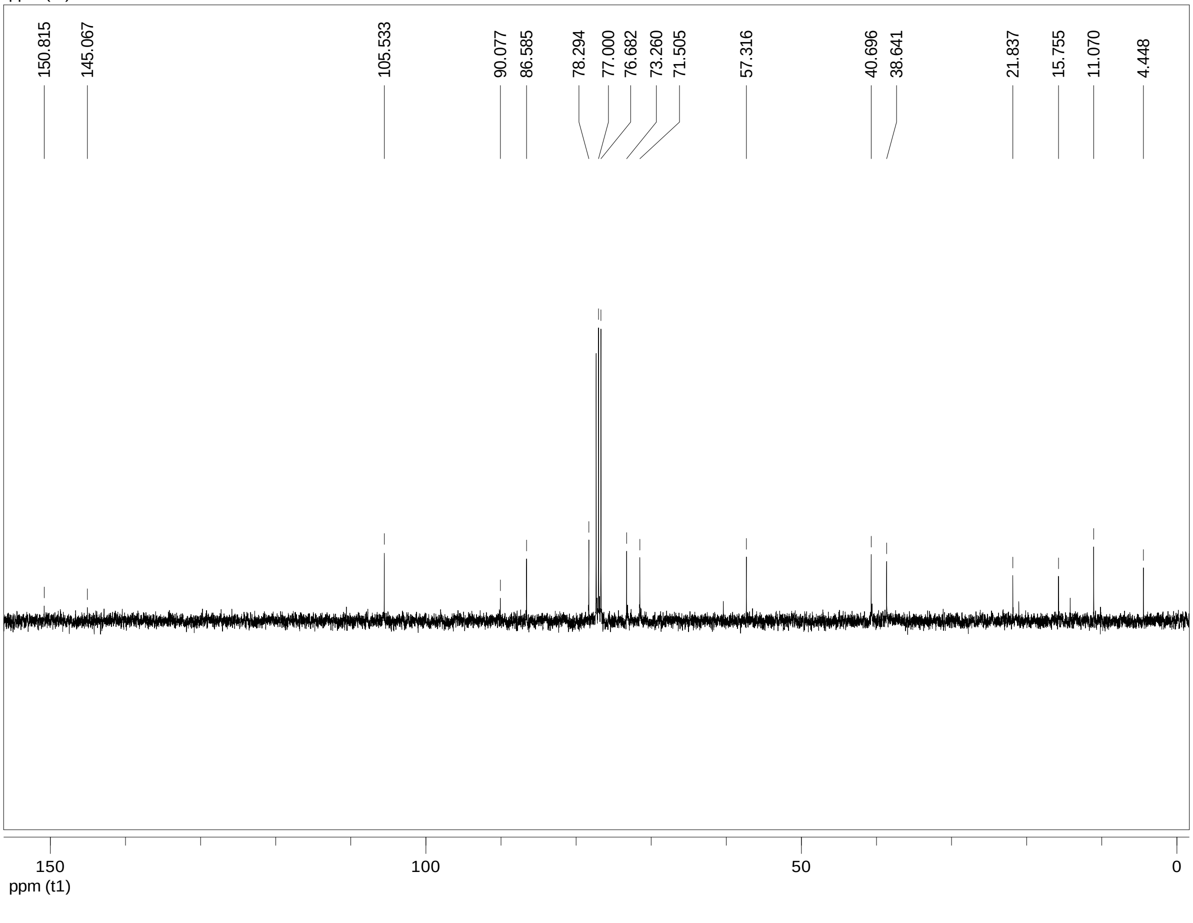

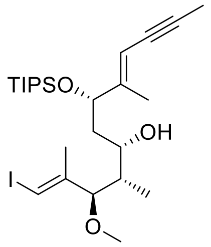

S31

(1*E*,3*R*,4*S*,5*S*,7*S*,8*E*)-1-Iodo-3-methoxy-2,4,8-trimethyl-7-((triisopropylsilyl)-oxy)dodeca-1,8-dien-10-yn-5-ol (S31): To a solution of alcohol **S30** (1.15 g, 2.93 mmol, 1.00 eq) in CH_2Cl_2 (79 mL) over molecular sieves (3 Å beads) was added 2,6-lutidine (1.71 mL, 14.7 mmol, 5.00 eq) at rt. The mixture was stirred at rt for 5 min, then it was cooled to −78 °C and, after another 5 min stirring, TIPSOTf (867 µL, 3.22 mmol, 1.10 eq) was added. After stirring at -78 °C for 1 h the reaction was quenched with $NaHCO_3$ (sat. aq.), the layers were separated and the aqueous phase was extracted with CH_2Cl_2 (3×20 mL). The combined organic extracts were washed with brine, dried over $MgSO_4$ and the solvent was removed under reduced pressure. The crude product was purified by column chromatography (pent/Et_2O 10:1→hexane/Et_2O 7:1) to afford the desired product **S31** as a colourless oil (1.56 g, 97%).

TLC (hexane/EtOAc, 5:1): R_f = 0.53

[α]$_D^{24}$ = +12.95 (*c* = 0.97, $CHCl_3$)

^{1}H-NMR (400.1 MHz, $CDCl_3$): δ = 6.10 (m_c, 1H), 5.65 (m_c, 1H), 4.53 (t, *J* = 4.1 Hz, 1H), 3.77 (d, *J* = 4.2 Hz, 1H), 3.74-3.66 (m, 1H), 3.50 (d, *J* = 2.4 Hz, 1H), 3.23 (s, 3H), 2.01 (d, *J* = 2.3 Hz, 3H), 1.81 (bs, 3H), 1.76-1.67 (m, 3H), 1.74 (d, *J* = 1.0 Hz, 3H), 1.09-1.04 (m, 21H), 0.81 (d, *J* = 7.0 Hz, 3H).

^{13}C-NMR (100.6 MHz, $CDCl_3$): δ = 149.5, 146.1, 106.3, 90.2, 86.1, 77.8, 76.7, 75.6, 69.8, 57.3, 41.3, 38.3, 21.3, 18.0 (3C), 16.3, 12.3 (6C), 9.9, 4.5.

IR (neat, ν/cm^{-1}): 3515br, 2942, 2893, 2866, 2369, 2358, 2344, 2333, 1620, 1463, 1380, 1255, 1193, 1165, 1089s, 1063s, 1012, 997, 950, 920, 882s, 854, 785, 755, 744, 678, 658, 601, 567, 495, 479, 451, 430, 418, 407.

HRMS: *m/z* calcd for $C_{25}H_{45}INaO_3Si$ $[M+Na]^+$: 571.2075; found: 571.2088.

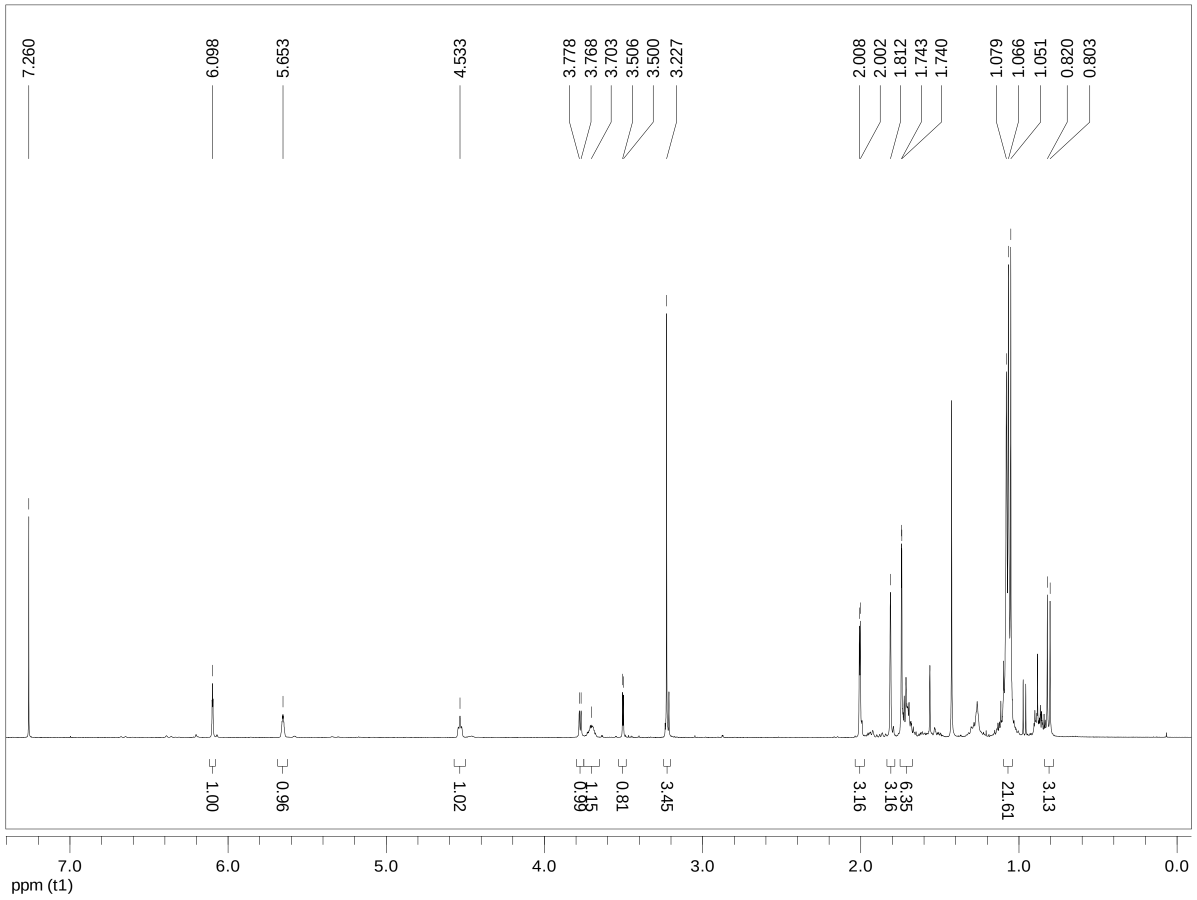
7.260
6.098
5.653
4.533
3.778
3.768
3.703
3.506
3.500
3.227
2.008
2.002
1.812
1.743
1.740
1.079
1.066
1.051
0.820
0.803
1.00
0.96
1.02
0.99
1.15
0.81
3.45
3.16
3.16
6.35
21.61
3.13
7.0
6.0
5.0
4.0
3.0
2.0
1.0
0.0
ppm (t1)

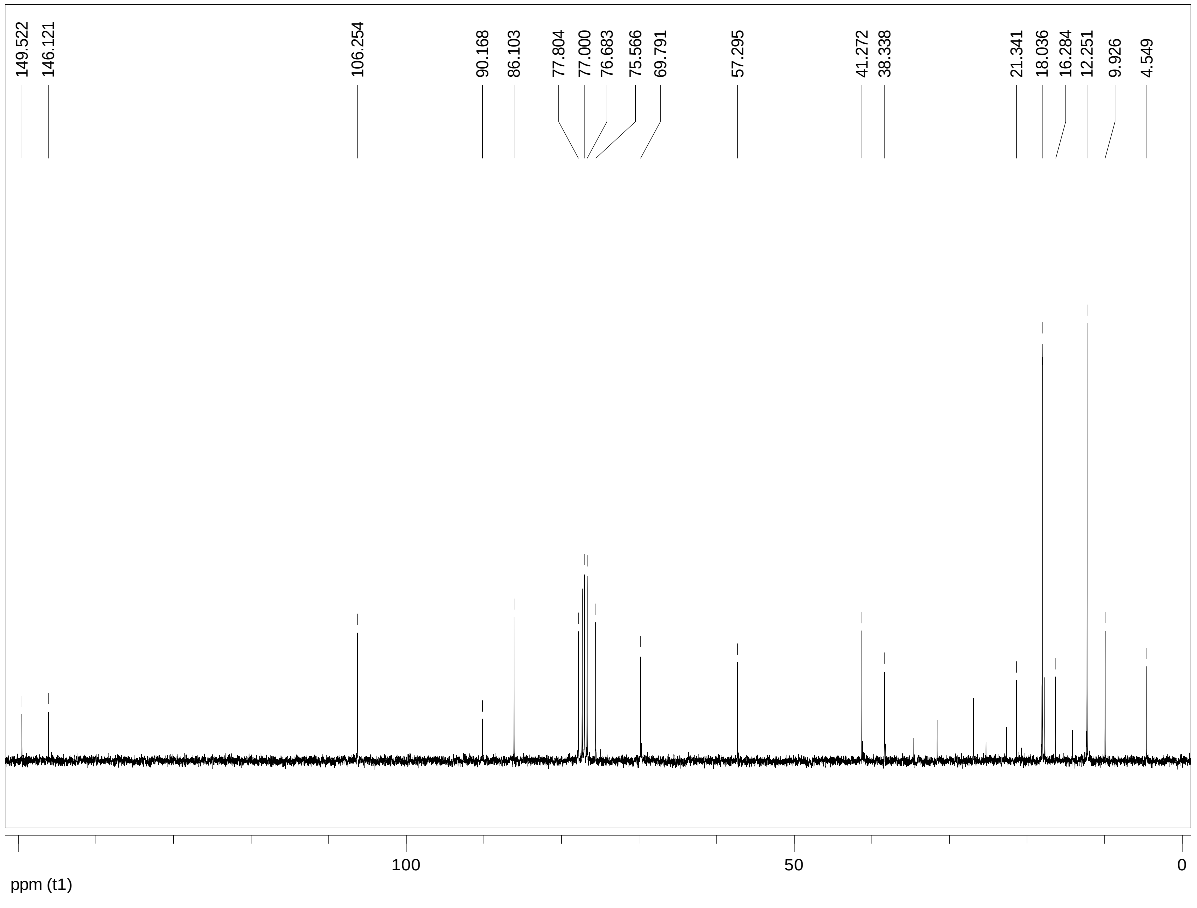
149.522
146.121
106.254
90.168
86.103
77.804
77.000
76.683
75.566
69.791
57.295
41.272
38.338
21.341
18.036
16.284
12.251
9.926
4.549
100
50
0
ppm (t1)

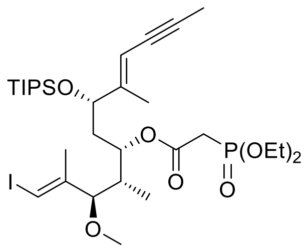

101

(1*E*,3*R*,4*S*,5*S*,7*S*,8*E*)-1-Iodo-3-methoxy-2,4,8-trimethyl-7-((triisopropylsilyl)oxy)-dodeca-1,8-dien-10-yn-5-yl 2-(diethoxyphosphoryl)acetate (101): To a cooled (0 °C) solution of diethylphosphonoacetic acid (**55**) (55 µL, 0.345 mmol, 1.50 eq) in CH_2Cl_2 (3 mL) over molecular sieves (beads, 3 Å) was added CME-carbodiimide (175 mg, 0.413 mmol, 1.80 eq). The mixture was stirred at 0 °C for 15 min, then a solution of alcohol **S31** (126 mg, 0.230 mmol, 1.00 eq) in CH_2Cl_2 (1 mL, rinsed with 0.9 mL, 0.5 mL) was added. The cooling bath was removed and DMAP (2.8 mg, 10 mol%) was added. After 3 h the mixture was concentrated under reduced pressure. The residue was purified by column chromatography (CH_2Cl_2:acetone 30:1→15:1) to afford the ester **101** (137 mg, 82%) as a pale yellow oil.

TLC (CH_2Cl_2/acetone, 10:1): R_f = 0.63

$[\alpha]_D^{24}$ = +28.41 (c = 0.995, $CHCl_3$)

^{1}H-NMR (400.1 MHz, $CDCl_3$): δ = 6.15 (m_c, 1H), 5.32-5.28 (m, 1H), 4.69 (ddd, *J* = 1.2 Hz, *J* = 4.4 Hz, *J* = 10.4 Hz, 1H), 4.20-4.10 (m, 4H), 4.07 (dd, *J* = 5.9 Hz, *J* = 7.8 Hz, 1H), 3.41 (d, *J* = 6.8 Hz, 1H), 3.17 (s, 3H), 2.91 (ddd, *J* = 14.5 Hz, *J* = 21.4 Hz, *J* = 30.0 Hz, 2H), 2.06-1.92 (m, 1H), 1.96 (d, *J* = 2.1 Hz, 3H), 1.91-1.77 (m, 1H), 1.81 (bs, 6H), 1.73-1.63 (m, 1H), 1.33 (m_c, 6H), 1.73-1.63 (m, 21H), 0.93 (d, *J* = 6.9 Hz, 3H).

^{13}C-NMR (100.6 MHz, $CDCl_3$): δ = 165.1 (d, *J* = 6.4 Hz), 150.9, 146.5, 106.5, 89.7, 87.1, 78.9, 76.8, 76.0, 73.6, 62.53, 62.46, 56.7, 39.0, 35.8, 34.3 (d, *J* = 134 Hz), 19.7, 18.13 (2C), 18.05, 16.35 (d, *J* = 5.7 Hz), 16.31 (d, *J* = 5.9 Hz), 13.7, 12.4 (6C), 9.7, 4.4.

IR (neat, ν/cm^{-1}): 2963, 2942, 2893, 2866, 1734, 1463, 1384, 1261, 1163, 1089, 1051 (s), 1024 (s), 966, 883, 856, 811, 784, 734, 682, 654, 620, 572, 501, 491, 477, 461, 446, 428, 419, 408.

HRMS: *m/z* calcd for $C_{31}H_{56}INaO_7PSi$ $[M+Na]^+$: 749.2470; found: 749.2476.

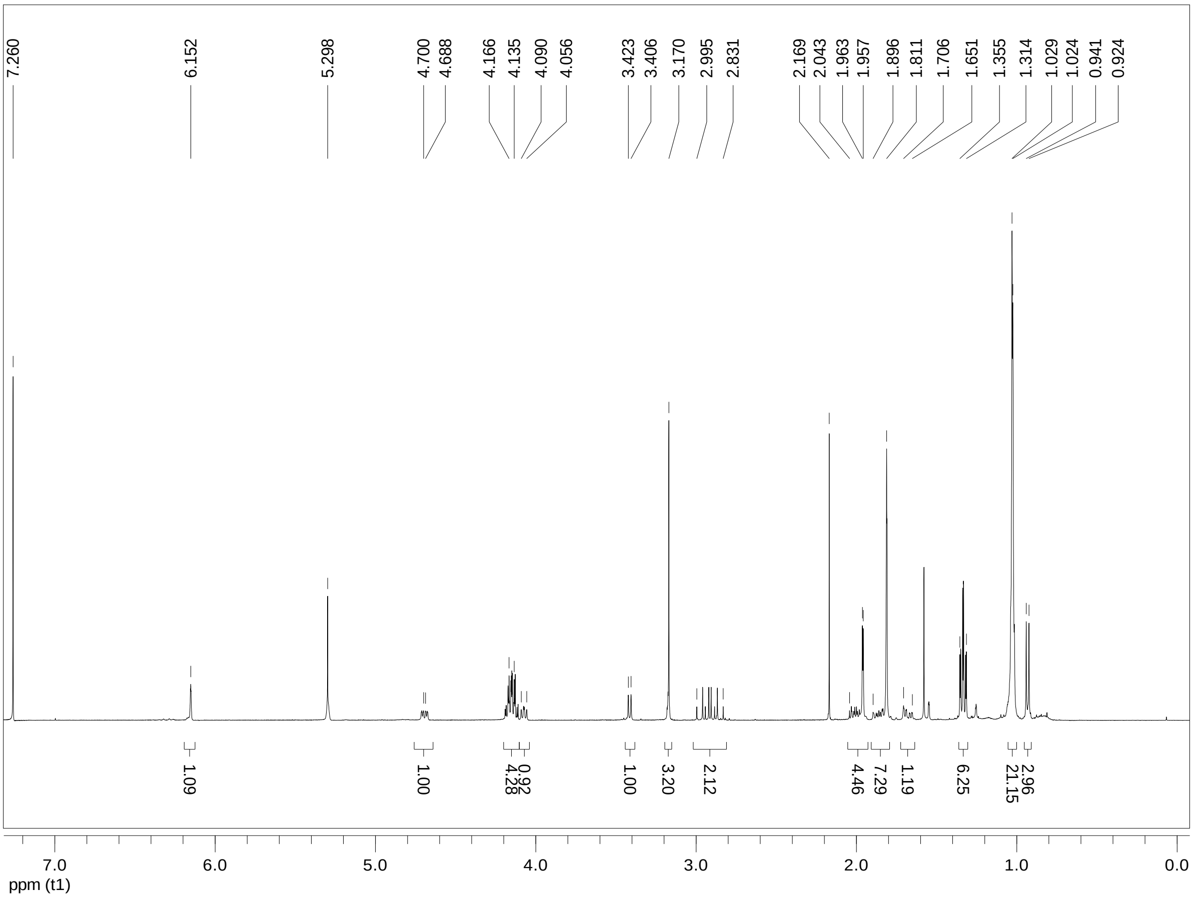
7.260
6.152
5.298
4.700
4.688
4.166
4.135
4.090
4.056
3.423
3.406
3.170
2.995
2.831
2.169
2.043
1.963
1.957
1.896
1.811
1.706
1.651
1.355
1.314
1.029
1.024
0.941
0.924
1.09
1.00
4.28
0.92
1.00
3.20
2.12
4.46
7.29
1.19
6.25
21.15
2.96
7.0
6.0
5.0
4.0
3.0
2.0
1.0
0.0
ppm (t1)

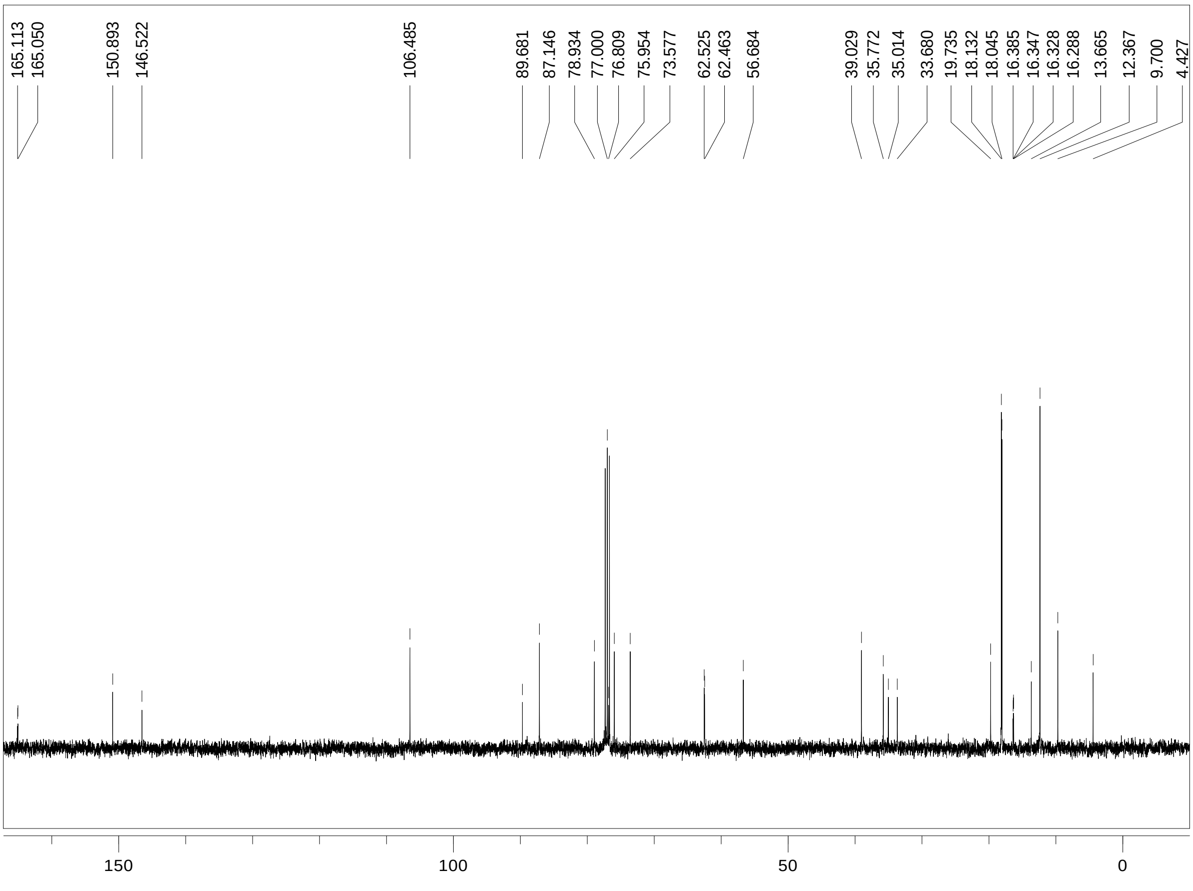
165.113
165.050
150.893
146.522
106.485
89.681
87.146
78.934
77.000
76.809
75.954
73.577
62.525
62.463
56.684
39.029
35.772
35.014
33.680
19.735
18.132
18.045
16.385
16.347
16.328
16.288
13.665
12.367
9.700
4.427
150
100
50
0

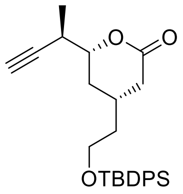

103

A 43.3 mM solution of the *Seyferth-Gilbert* reagent (dimethyl (diazomethyl)phosphonate) in THF was prepared as follows: To a solution of diazo phosphonate **102** (13.0 mg, 0.09 mmol, 1.1 equiv) in THF (2.0 mL) was added solid potassium *tert*-butoxide (11.0 mg, 0.10 mmol, 1.2 equiv) at −78 °C in one portion. After stirring at –78 °C for 30 min, 0.16 mL of the solution (1.1 equiv of *Seyferth-Gilbert* reagent) were added at –78 °C to a solution of crude **66** (2.8 mg, 0.01 mmol, 1.0 equiv) in THF (0.5 mL). After stirring for 2 h at –78 °C, the reaction mixture was quenched with pH7 buffer (2 mL). The reaction mixture was diluted with Et_2O and brine. The layers were separated and the aqueous layer was extracted with Et_2O (2 x 10 mL). The combined organic extracts were washed with brine, dried over $MgSO_4$, filtered and concentrated under reduced pressure. The crude product was purified by column chromatography (hexane/EtOAc, 5:1) to afford **103** (1.2 mg, 43%) as a colorless oil. The analytical data were identical to those reported in literature.[22]

R*f* = 0.32 (hexane/EtOAc, 5:1).
^{1}H-NMR (400.1 MHz, $CDCl_3$): δ = 7.68-7.62 (m, 4H), 7.47-7.36 (m, 6H), 4.11 (ddd, *J* = 2.9, 6.9, 11.8 Hz, 1H), 3.72 (t, *J* = 6.0 Hz, 2H), 2.74 (m_c, 1H), 2.67 (ddd, *J* = 2.2, 5.2, 17.1 Hz, 1H), 2.26-2.13 (m, 2H), 2.11 (d, *J* = 2.5 Hz, 1H), 2.11-2.02 (m, 1H), 1.65-1.51 (m, 2H), 1.40-1.27 (m, 1H), 1.28 (d, *J* = 7.0 Hz, 3H), 1.05 (s, 9H).
^{13}C-NMR (100.6 MHz, $CDCl_3$): δ = 170.7, 135.5, 133.5, 129.8, 127.7, 83.9, 82.2, 71.3, 60.8, 38.6, 36.4, 32.1, 32.0, 28.2, 26.9, 19.2, 17.0.
HRMS (ESI): *m/z* calcd for $C_{27}H_{38}NO_3Si$ [M + NH_4]$^+$: 452.2615, found: 452.2628.

[22] Mitchell, I. S.; Pattenden, G.; Stonehouse, J. *Org. Biomol. Chem.* **2005**, *3*, 4412-4431.

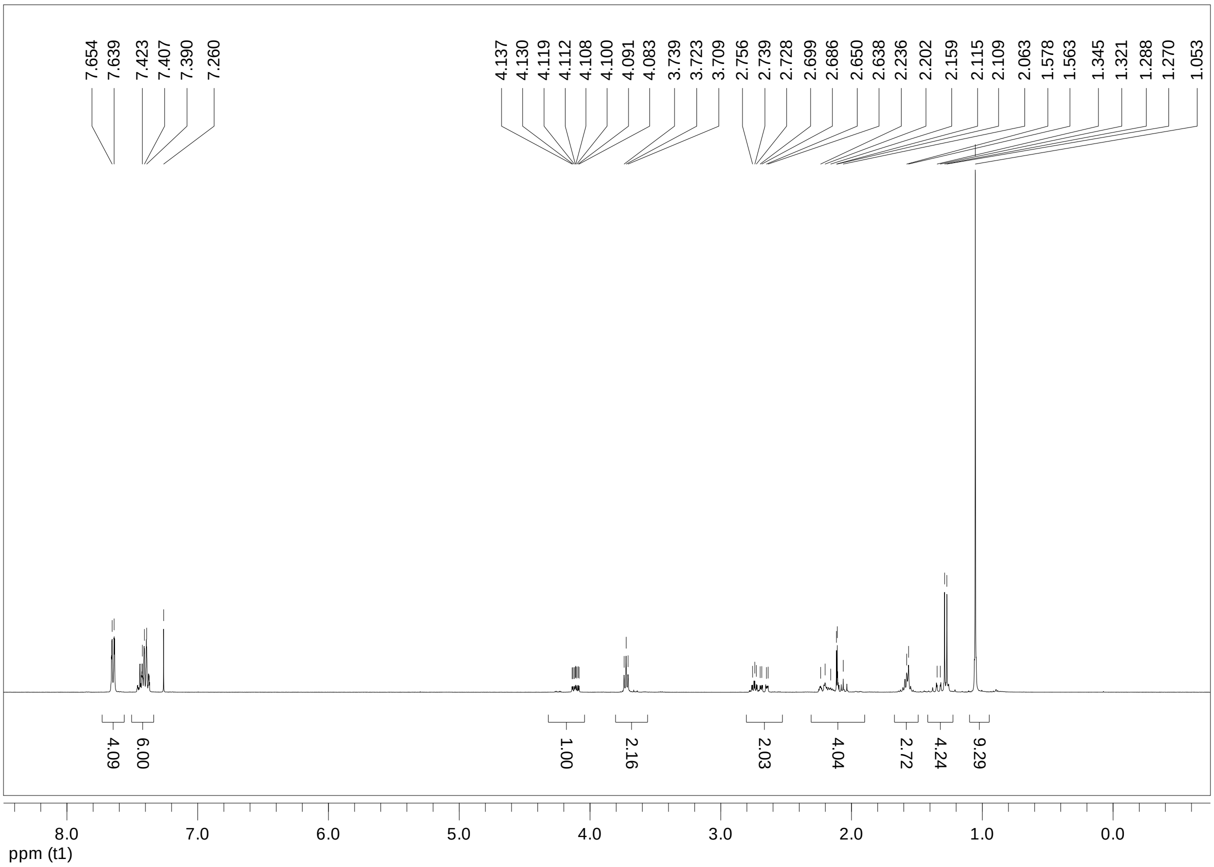
7.654
7.639
7.423
7.407
7.390
7.260
4.137
4.130
4.119
4.112
4.108
4.100
4.091
4.083
3.739
3.723
3.709
2.756
2.739
2.728
2.699
2.686
2.650
2.638
2.236
2.202
2.159
2.115
2.109
2.063
1.578
1.563
1.345
1.321
1.288
1.270
1.053
4.09
6.00
1.00
2.16
2.03
4.04
2.72
4.24
9.29
8.0
7.0
6.0
5.0
4.0
3.0
2.0
1.0
0.0
ppm (t1)

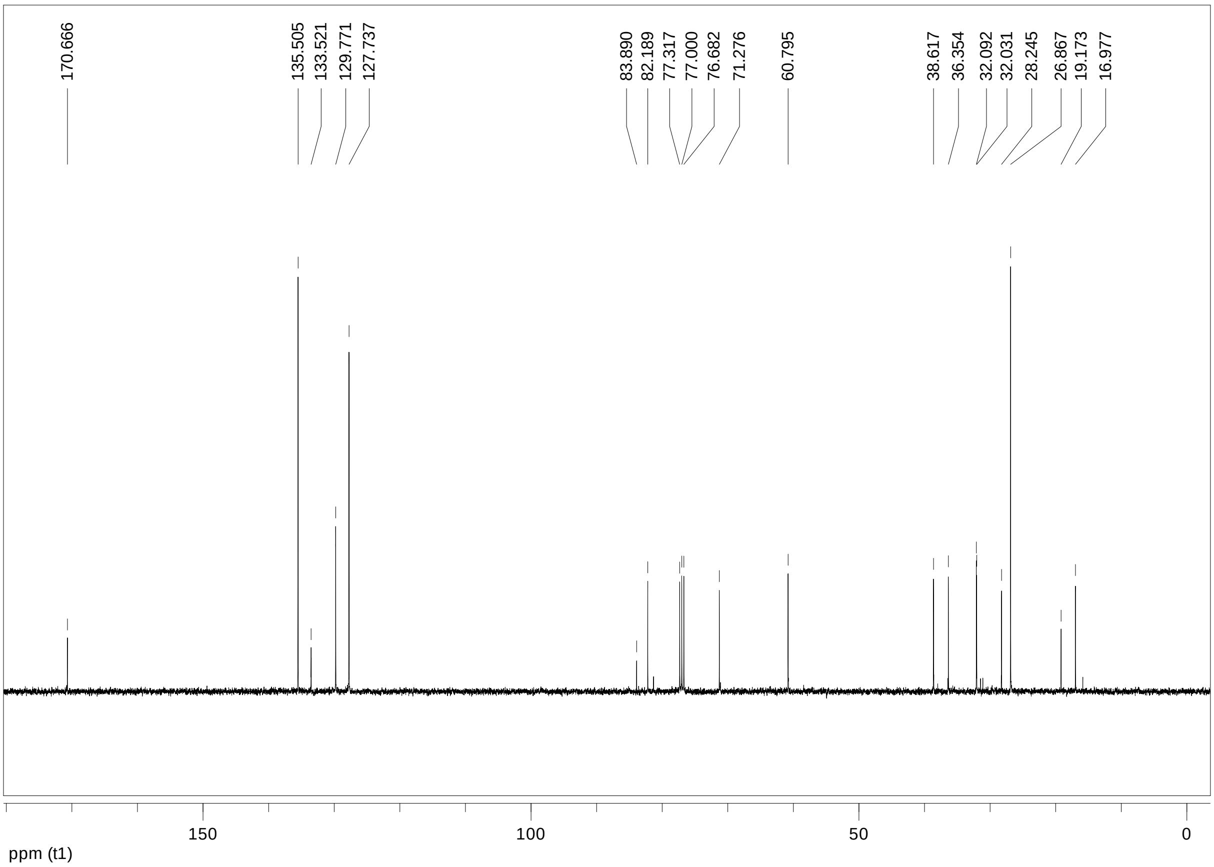
170.666
135.505
133.521
129.771
127.737
83.890
82.189
77.317
77.000
76.682
71.276
60.795
38.617
36.354
32.092
32.031
28.245
26.867
19.173
16.977
150
100
50
0
ppm (t1)

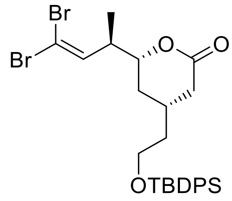

104

(4*R*,6*R*)-4-(2-((*tert*-Butyldiphenylsilyl)oxy)ethyl)-6-((*R*)-4,4-dibromobut-3-en-2-yl)tetrahydro-2H-pyran-2-one (104): To a solution of CBr_4 (408 mg, 1.23 mmol, 1.8 equiv) in CH_2Cl_2 (2.5 mL) was added at 0 °C triphenylphosphine (645 mg, 2.46 mmol, 3.6 equiv). After stirring for a few minutes at 0 °C, the yellow reaction mixture was cooled to –78 °C and a solution of **103** (300 mg, 0.68 mmol, 1.0 equiv, crude) in CH_2Cl_2 (4.4 mL) was added dropwise. After stirring for 45 min at –78 °C, the reaction mixture was diluted with sat. NH_4Cl (30 mL) and CH_2Cl_2 (30 mL). The layers were separated and the aqueous layer was extracted with CH_2Cl_2 (2 x 15 mL). The combined organic extracts were dried over $MgSO_4$, filtered and concentrated under reduced pressure. The residue was purified by column chromatography (hexane/EtOAc, 15:1 → 10:1 → 5:1) to afford **104** (325 mg, 80% over 2 steps) as a colorless oil.

R_f = 0.47 (hexane/EtOAc, 5:1)

$[\alpha]_D^{24}$ = –20.2° [*c* = 0.189, $CHCl_3$]

^{1}H-NMR (400.1 MHz, $CDCl_3$): δ = 7.69-7.60 (m, 4H), 7.49-7.34 (m, 6H), 6.38 (d, *J* = 9.6 Hz, 1H), 4.14 (ddd, *J* = 2.8, 5.5, 11.9 Hz, 1H), 3.71 (t, *J* = 6.0 Hz, 2H), 2.72-2.59 (m, 2H), 2.16 (m_c, 1H), 2.06 (dd, *J* = 10.9, 17.2 Hz, 1H), 1.84 (d, *J* = 13.5 Hz, 1H), 1.56 (m_c, 2H), 1.31-1.12 (m, 1H), 1.10 (d, *J* = 6.8 Hz, 3H), 1.06 (s, 9H).

^{13}C-NMR (100.6 MHz, $CDCl_3$): δ = 170.7, 139.1, 135.5, 133.5, 133.4, 129.8, 127.7, 90.1, 81.6, 60.7, 43.2, 38.6, 36.3, 32.7, 28.4, 26.9, 19.1, 13.7.

IR (neat, ν/cm^{-1}): 3070w, 2930m, 2857w, 1736s, 1471w, 1428m, 1388w, 1227m, 1107s, 1007m, 822m, 787m, 738s, 701s, 613m, 504s.

HRMS (ESI): *m/z* calcd for $C_{27}H_{34}Br_2NaO_3Si$ $[M + Na]^+$: 615.0536, found: 615.0545.

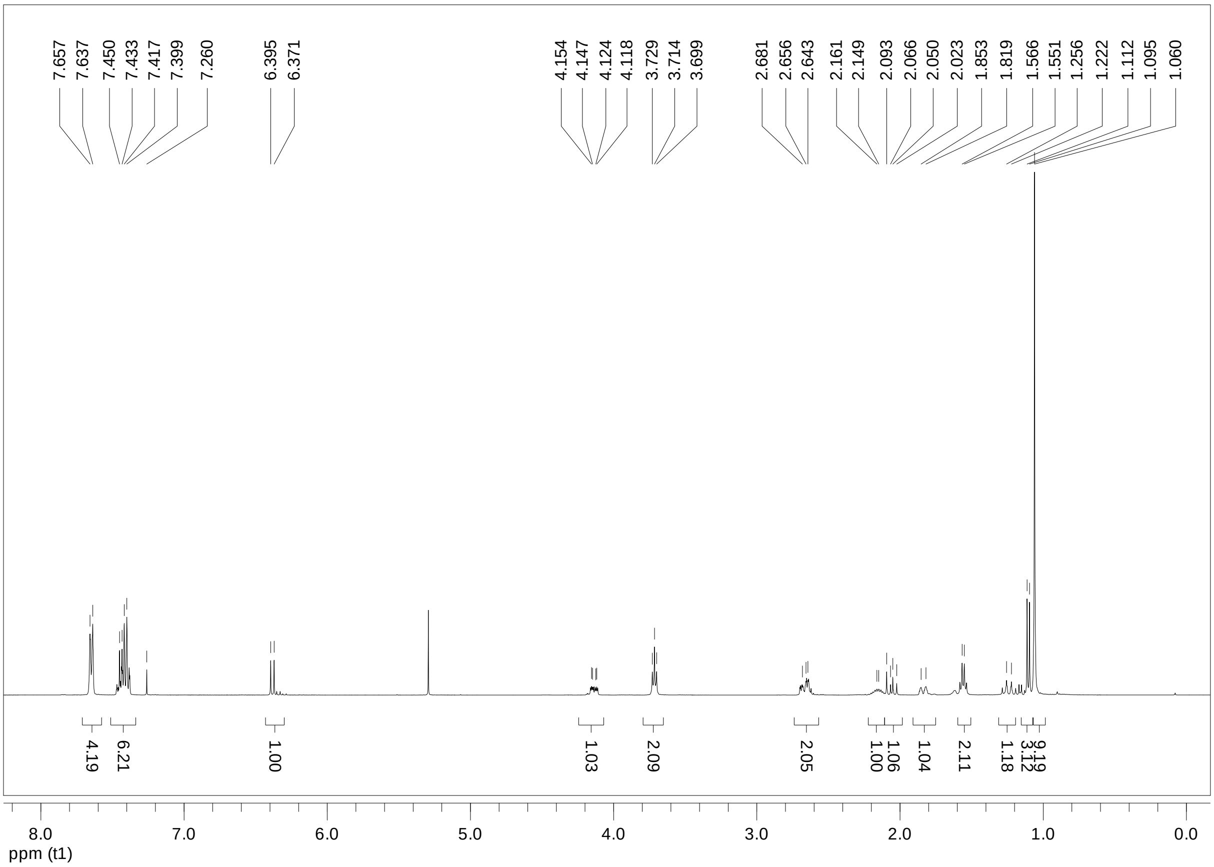
7.657
7.637
7.450
7.433
7.417
7.399
7.260
6.395
6.371
4.154
4.147
4.124
4.118
3.729
3.714
3.699
2.681
2.656
2.643
2.161
2.149
2.093
2.066
2.050
2.023
1.853
1.819
1.566
1.551
1.256
1.222
1.112
1.095
1.060
4.19
6.21
1.00
1.03
2.09
2.05
1.00
1.06
1.04
2.11
1.18
3.12
9.19
8.0
7.0
6.0
5.0
4.0
3.0
2.0
1.0
0.0
ppm (t1)

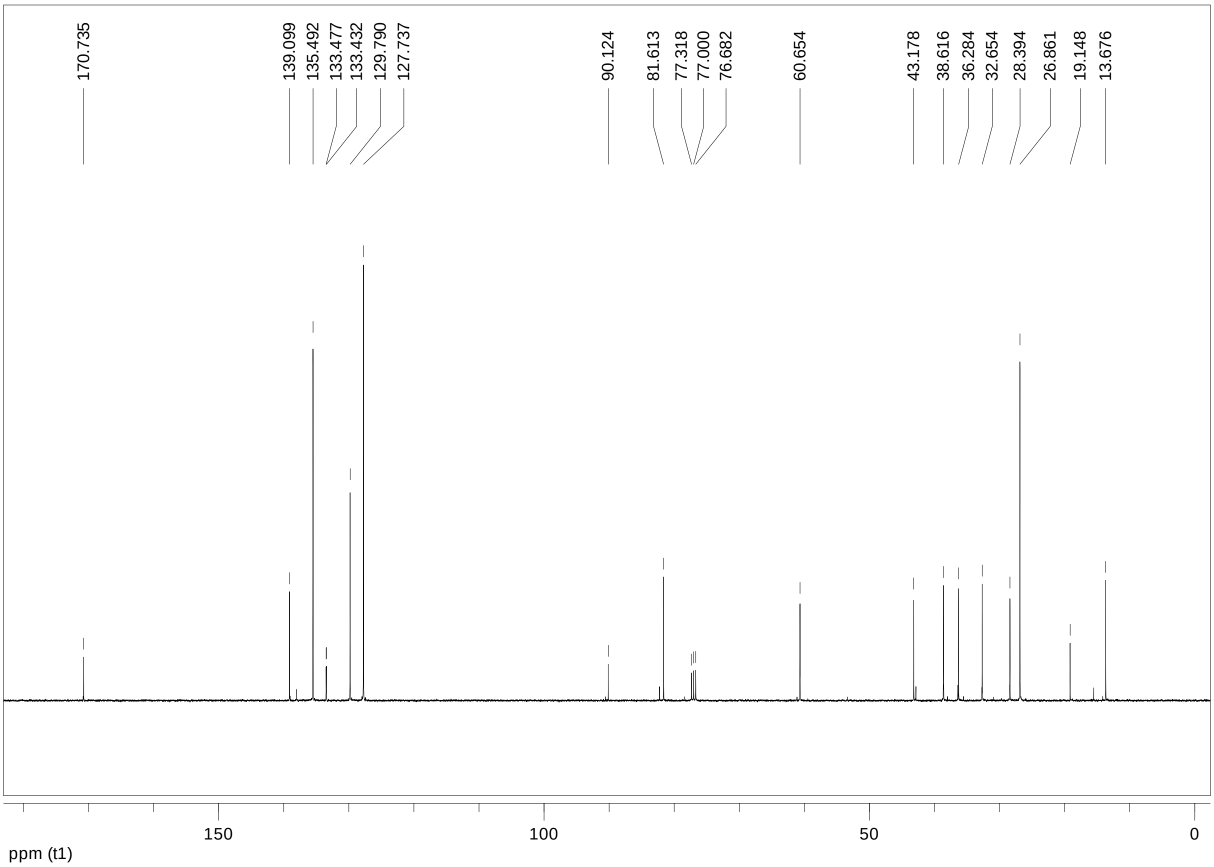
170.735
139.099
135.492
133.477
133.432
129.790
127.737
90.124
81.613
77.318
77.000
76.682
60.654
43.178
38.616
36.284
32.654
28.394
26.861
19.148
13.676
150
100
50
0
ppm (t1)

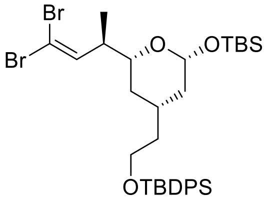

105

***tert*-Butyl(2-((2*R*,4*R*,6*R*)-2-(*tert*-butyldimethylsilyloxy)-6-((*R*)-4,4-dibromobut-3-en-2-yl)tetrahydro-2H-pyran-4-yl)ethoxy)diphenylsilane (105):** To a solution of **104** (331 mg, 0.56 mmol, 1.00 equiv) in CH_2Cl_2 (6 mL) was added dropwise at –78 °C DIBAL (0.56 mL, 0.67 mmol, 1.20 equiv, 1.2 M in toluene). The mixture was stirred at the same temperature for 1 h. Because incomplete conversion was observed according to TLC, more DIBAL (46.4 µL, 0.1 equiv) was added. After stirring for 30 min at –78 °C, the reaction mixture was quenched with sat. *Rochelle* salt (25 mL) and diluted with CH_2Cl_2. After stirring for 15 min at rt, the layers were separated. The aqueous phase was extracted with CH_2Cl_2 (3 x 15 mL) and the combined organic extracts were washed with brine and dried over $MgSO_4$. The solvent was removed under reduced pressure. The crude hemiacetal (353 mg, >100%) was used for the next step without further purification.

R_f = 0.39 (hexane/EtOAc, 5:1)

To a solution of the above hemiacetal (332 mg, 0.56 mmol, 1.00 equiv) in CH_2Cl_2 (4.5 mL) was added at rt imidazole (49.3 mg, 0.72 mmol, 1.30 equiv) followed by TBSCl (101 mg, 0.67 mmol, 1.20 equiv). Upon addition of the TBSCl, a colorless precipitate formed immediately. After stirring for 3 h at rt, the reaction mixture was quenched with sat. NH_4Cl (15 mL). The layers were separated and the aqueous phase was extracted with CH_2Cl_2 (3 x 10 mL). The combined organic extracts were washed with brine, dried over $MgSO_4$ and concentrated under reduced pressure. The crude product was purified by column chromatography (hexane/EtOAc, 40:1) to furnish **105** (345 mg, 87% over two steps) as a colorless oil.

R_f = 0.24 (hexane/EtOAc, 30:1)

$[\alpha]_D^{24}$ = –3.9° [c = 0.565, $CHCl_3$]

^{1}H-NMR (400.1 MHz, $CDCl_3$): δ = 7.69-7.62 (m, 4H), 7.47-7.35 (m, 6H), 6.28 (d, *J* = 9.7 Hz, 1H), 4.63 (dd, *J* = 2.0, 9.3 Hz, 1H), 3.70 (t, *J* = 6.3 Hz, 2H), 3.18 (ddd, *J* = 1.8, 6.7, 11.2 Hz, 1H), 2.56 (m_c, 1H), 1.84-1.64 (m, 2H), 1.56-1.42 (m, 3H), 1.09-0.98 (m, 4H), 1.05 (s, 9H), 0.97-0.85 (m, 1H), 0.91 (s, 9H), 0.13 (s, 3H), 0.11 (s, 3H).

^{13}C-NMR (100.6 MHz, $CDCl_3$): δ = 141.2, 135.6, 133.9, 129.6, 127.6, 97.2, 88.5, 77.2, 61.2, 43.4, 40.4, 39.0, 34.9, 30.8, 26.9, 25.8, 19.2, 18.1, 14.7, –3.9, –5.2.

IR (neat, ν/cm^{-1}): 3072w, 2930s, 2857m, 1472m, 1428m, 1389m, 1249m, 1173m, 1111s, 1077m, 1054w, 836s, 785s, 737m, 702s, 616w, 506m.

HRMS (ESI): *m/z* calcd for $C_{33}H_{50}Br_2KO_3Si_2$ [M + K]$^+$: 747.1297, found: 747.1315.

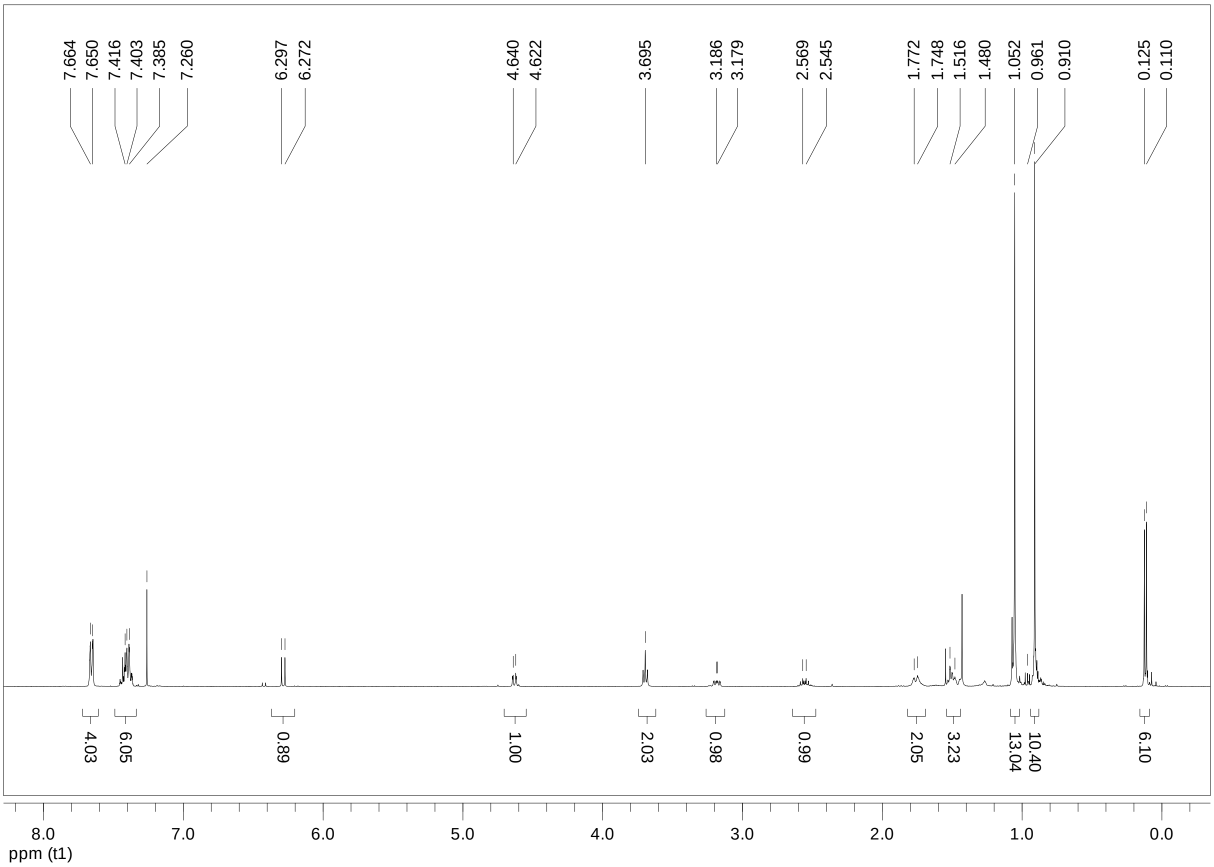

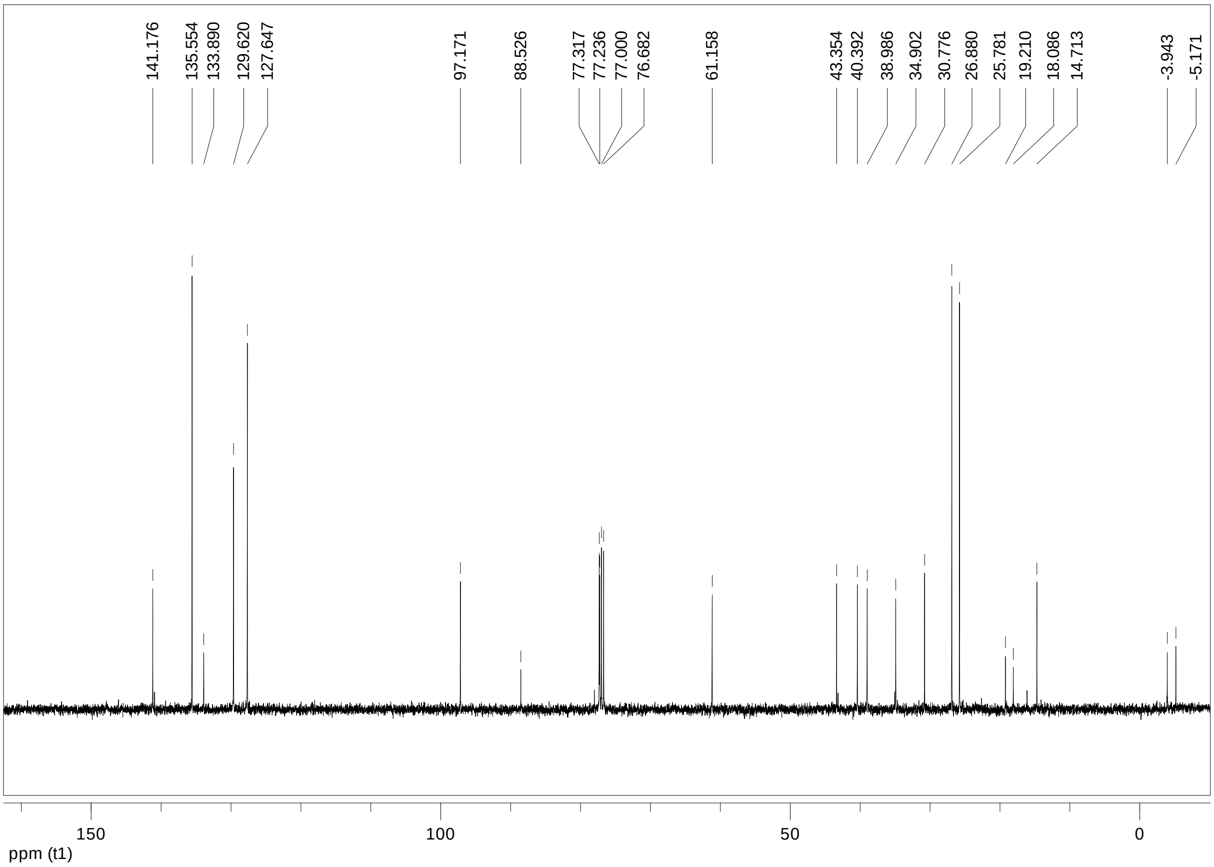

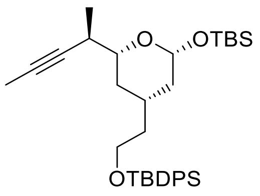

106

***tert*-Butyl(2-((2*R*,4*R*,6*R*)-2-(*tert*-butyldimethylsilyloxy)-6-((*R*)-pent-3-yn-2-yl)tetrahydro-2H-pyran-4-yl)ethoxy)diphenylsilane (106):** To a solution of **105** (294 mg, 0.41 mmol. 1.0 equiv) in THF (1.9 mL) was added at –78 °C *n*-BuLi (0.85 mL, 1.37 mmol, 3.3 equiv, 1.6 M in hexane). The reaction mixture was allowed to warm to rt over 45 min, then MeI (85 µL, 1.37 mmol, 3.3 equiv) was added rt to the yellowish reaction mixture. After stirring for 1.25 h at rt, the bright yellow reaction mixture was quenched with sat. NH_4Cl (20 mL) and diluted with Et_2O (30 mL). The layers were separated and the organic layer was extracted with Et_2O (2 x 30 mL). The combined organic extracts were washed with brine, dried over $MgSO_4$ and concentrated *in vacuo*. The residue was purified by column chromatography (hexane/Et_2O, 70:1 → 30:1) to give **106** (210 mg, 90%) as a colorless oil.

R_f = 0.37 (hexane/ Et_2O, 30:1)

$[\alpha]_D^{24}$ = +10.8° [*c* = 0.409, $CHCl_3$]

^{1}H-NMR (400.1 MHz, $CDCl_3$): δ = 7.71-7.62 (m, 4H), 7.46-7.34 (m, 6H), 4.64 (dd, *J* = 1.9, 9.3 Hz, 1H), 3.72 (dt, *J* = 1.8, 6.3 Hz, 2H), 3.13 (ddd, *J* = 1.8, 8.7, 10.7 Hz, 1H), 2.45 (m_c, 1H), 1.96 (d, *J* = 13.1 Hz, 1H), 1.86-1.69 (m, 2H), 1.77 (d, *J* = 2.4 Hz, 3H), 1.62-1.45 (m, 2H), 1.22 (d, *J* = 6.8 Hz, 3H), 1.09-0.96 (m, 1H), 1.05 (s, 9H), 0.93-0.80 (m, 1H), 0.90 (s, 9H), 0.12 (s, 3H), 0.11 (s, 3H).

^{13}C-NMR (100.6 MHz, $CDCl_3$): δ = 135.5, 134.0, 129.6, 127.6, 97.1, 81.1, 78.8, 77.2, 61.3, 40.4, 39.1, 35.7, 31.9, 30.8, 26.9, 25.8, 19.2, 18.2, 18.1, 3.6, –4.0, –5.3.

IR (neat, ν/cm^{-1}): 2929m, 2857m, 1472w, 1428m, 1389m, 1250w, 1174m, 1158m, 1108s, 1078s, 1007m, 939w, 900w, 835s, 780s, 737m, 701s, 614m, 504s, 490m.

HRMS (ESI): *m/z* calcd for $C_{34}H_{52}NaO_3Si_2$ [M + Na]$^+$: 587.3347, found: 587.3344.

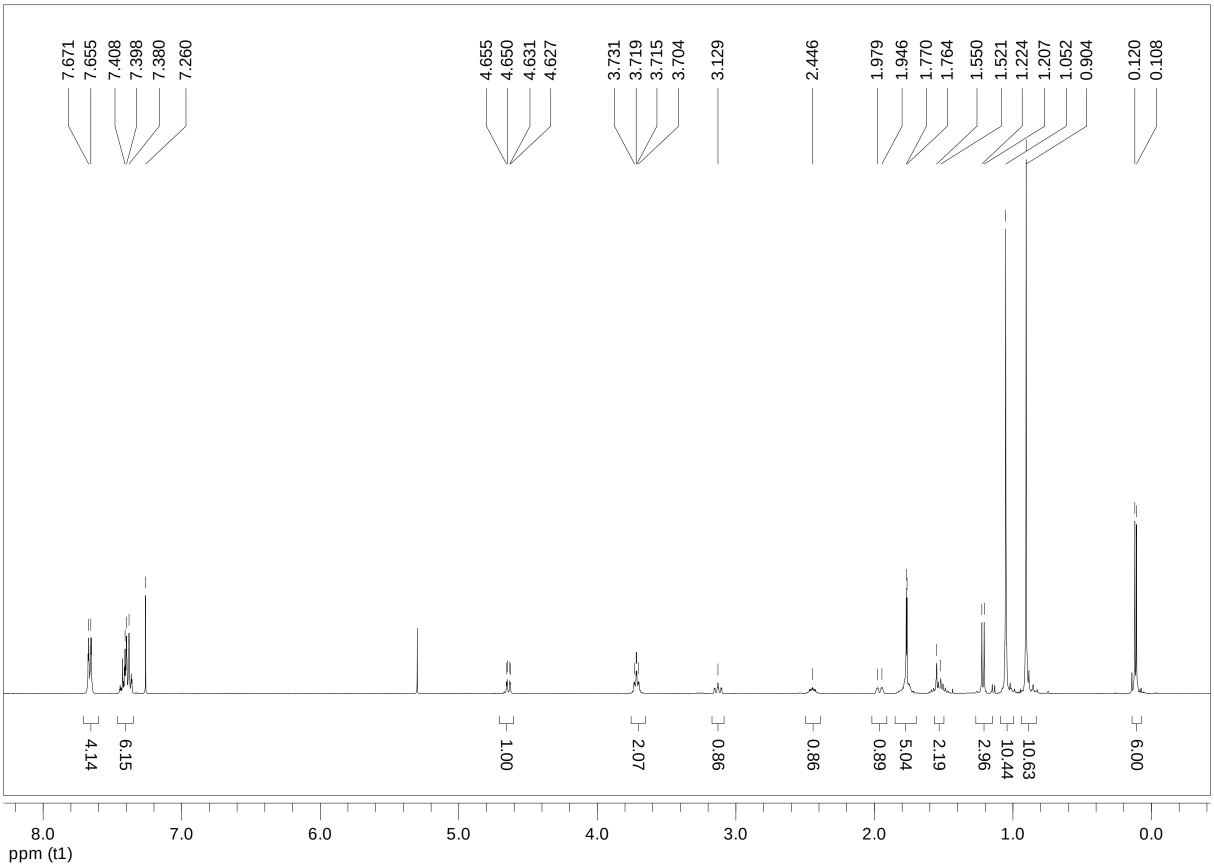
7.671
7.655
7.408
7.398
7.380
7.260
4.655
4.650
4.631
4.627
3.731
3.719
3.715
3.704
3.129
2.446
1.979
1.946
1.770
1.764
1.550
1.521
1.224
1.207
1.052
0.904
0.120
0.108
4.14
6.15
1.00
2.07
0.86
0.86
0.89
5.04
2.19
2.96
10.44
10.63
6.00
8.0
7.0
6.0
5.0
4.0
3.0
2.0
1.0
0.0
ppm (t1)

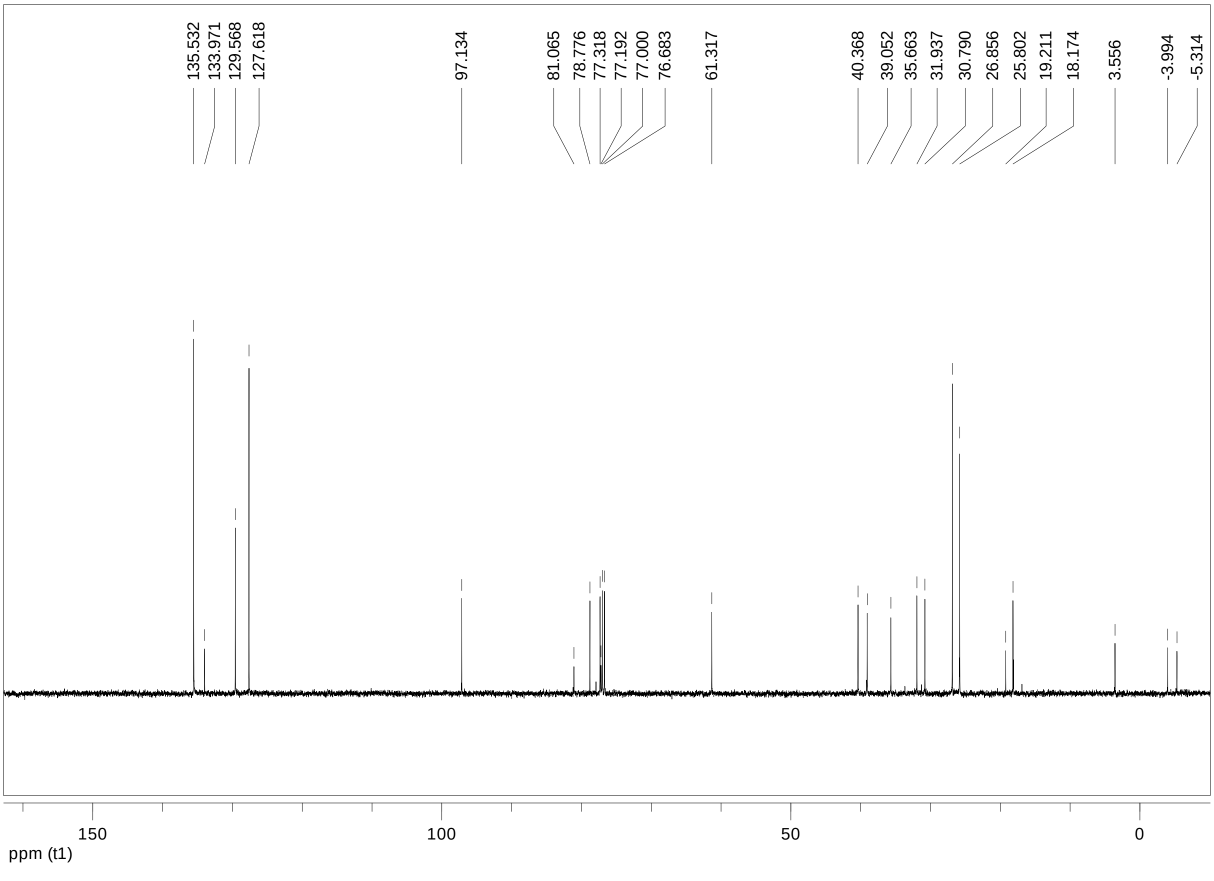
135.532
133.971
129.568
127.618
97.134
81.065
78.776
77.318
77.192
77.000
76.683
61.317
40.368
39.052
35.663
31.937
30.790
26.856
25.802
19.211
18.174
3.556
-3.994
-5.314
150
100
50
0
ppm (t1)

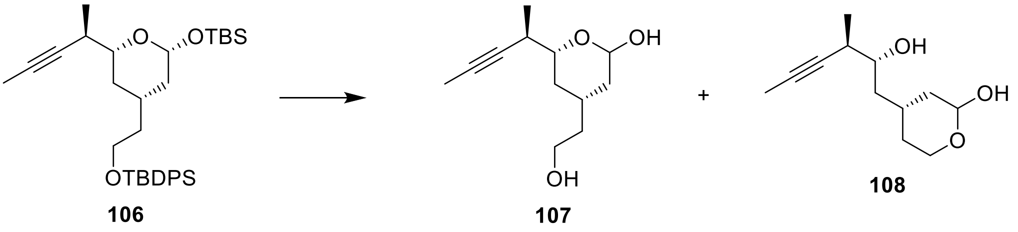

(4*R*,6*R*)-4-(2-Hydroxyethyl)-6-((*R*)-pent-3-yn-2-yl)tetrahydro-2H-pyran-2-ol (107) and (4*R*)-4-((2*R*,3*R*)-2-hydroxy-3-methylhex-4-yn-1-yl)tetrahydro-2H-pyran-2-ol (108): To a solution of **106** (52.4 mg, 92.8 µmol, 1.0 equiv) in THF (0.95 mL) was added at 0 °C TBAF (0.40 mL, 0.40 mmol, 4.3 equiv, 1.0 M in THF) and AcOH (22.7 µL, 0.40 mmol, 4.3 equiv). After stirring for 16 h at rt, the reaction mixture was diluted with EtOAc and quenched at 0 °C with sat. $NaHCO_3$ (15 mL) and EtOAc (15 mL). The layers were separated and the aqueous layer was extracted with EtOAc (2 x 15 mL). The combined organic extracts were washed with brine, dried over $MgSO_4$ and the solvent was removed under reduced pressure. The residue was purified by column chromatography (hexane/EtOAc, 1:1 → 1:2→ 0:1) to give **107/108** (22.7 mg, quant., 2:1) as a colorless oil.

$\mathbf{R}_f$ = 0.47 (EtOAc).

HRMS (ESI): *m/z* calcd for $C_{12}H_{20}NaO_3$ [M + Na]$^+$: 235.1305, found: 235.1306.

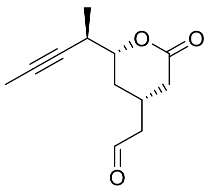

109

2-((4*S*,6*R*)-2-Oxo-6-((*R*)-pent-3-yn-2-yl)tetrahydro-2H-pyran-4-yl)acetaldehyde (109):

BAIB/TEMPO: To a solution of **107/108** (20.4 mg, 96.1 µmol, 1.0 equiv, mixture of acetals) in CH_2Cl_2 (0.65 mL) was added at rt TEMPO (2.9 mg, 19 mol%) and BAIB (67.8 mg, 210 µmol, 2.2 equiv). The yellowish reaction mixture was stirred at rt for 45 min. $Yb(OTf)_3$ (2.3 mg, 3.9 mol%) was added at 0 °C and stirring was continued at rt for 45 min. The reaction mixture was diluted with CH_2Cl_2 (4 mL) and quenched with sat. $Na_2S_2O_3$ (2 mL). Brine (15 mL) was added and the aqueous layer was extracted with CH_2Cl_2 (3 x 10 mL). The combined organic extracts were dried over $MgSO_4$ and the solvent was removed under reduced pressure. The residue was purified by column chromatography (hexane/EtOAc, 1:1 → 1:2) to give aldehyde **109** (12.0 mg, 62% over 2 steps) and hydroxy lactone **110** (2.6 mg, 13%, impure).

R_f = 0.32 (hexane/EtOAc 1:1)
$[\alpha]_D^{24}$ = +33.4° [*c* = 0.760, $CHCl_3$]
^{1}H-NMR (400.1 MHz, $CDCl_3$): δ = 9.77 (s, 1H), 4.11 (ddd, *J* = 3.0, 7.1, 11.5 Hz, 1H), 2.77 (ddd, *J* = 1.9, 5.6, 17.6 Hz, 1H), 2.67 (dtt, *J* = 2.4, 4.7, 9.4 Hz, 1H), 2.61-2.47 (m, 3H), 2.26 (m_c, 1H), 2.12 (dd, *J* = 10.4, 17.5 Hz, 1H), 1.77 (d, *J* = 2.4 Hz, 3H), 1.36 (m_c, 1H), 1.22 (d, *J* = 7.0 Hz, 3H).
^{13}C-NMR (100.6 MHz, $CDCl_3$): δ = 199.6, 170.0, 82.4, 78.9, 78.4, 49.7, 35.8, 32.2, 31.9, 25.7, 17.4, 3.5.
IR (neat, ν/cm^{-1}): 2921m, 2857w, 2729w, 1722s, 1444m, 1384m, 1359, 1236s, 1185m, 1081s, 1029m, 1010m, 968w.
HRMS (ESI): *m/z* calcd for $C_{12}H_{16}NaO_3$ $[M + Na]^+$: 231.0992, found: 231.0991.

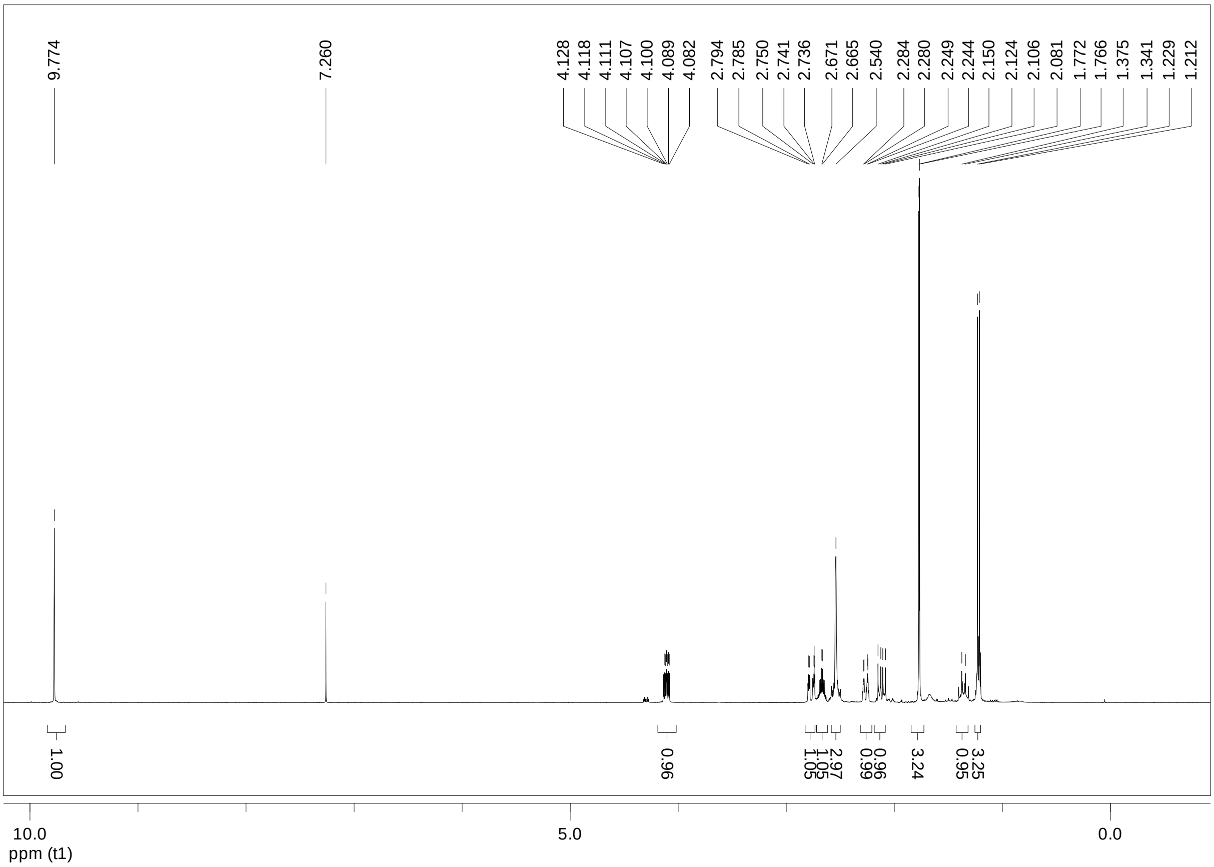
9.774
7.260
4.128
4.118
4.111
4.107
4.100
4.089
4.082
2.794
2.785
2.750
2.741
2.736
2.671
2.665
2.540
2.284
2.280
2.249
2.244
2.150
2.124
2.106
2.081
1.772
1.766
1.375
1.341
1.229
1.212
1.00
0.96
1.05
1.05
2.97
0.99
0.96
3.24
0.95
3.25
10.0
5.0
0.0
ppm (t1)

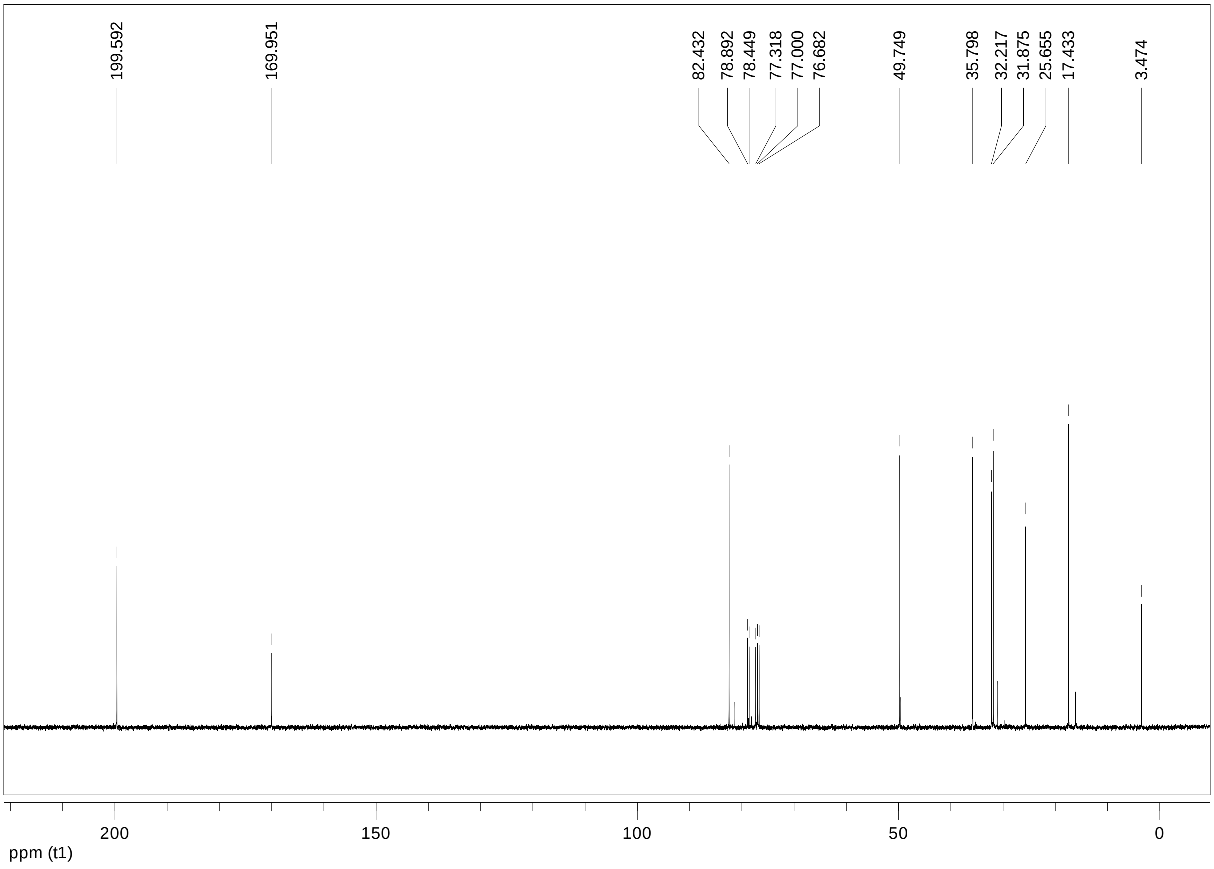
199.592
169.951
82.432
78.892
78.449
77.318
77.000
76.682
49.749
35.798
32.217
31.875
25.655
17.433
3.474
200
150
100
50
0
ppm (t1)

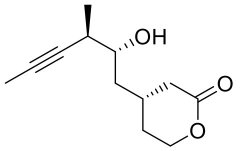

110

R*f* = 0.16 (hexane/EtOAc 1:1)

^{1}H-NMR (400.1 MHz, $CDCl_3$): δ = 4.43 (ddd, *J* = 3.9, 4.9, 11.4 Hz, 1H), 4.28 (ddd, *J* = 3.7, 10.8, 11.4 Hz, 1H), 3.60 (m_c, 1H), 2.76 (m_c, 1H), 2.56 (m_c, 1H), 2.37-2.16 (m, 2H), 2.09-1.98 (m, 1H), 1.81 (d, *J* = 2.4 Hz, 3H), 1.71 (d, *J* = 6.3 Hz, 1H), 1.60-1.44 (m, 3H), 1.13 (d, *J* = 7.0 Hz, 3H).

HRMS (ESI): *m/z* calcd for $C_{12}H_{18}NaO_3$ $[M + Na]^+$: 233.1148, found: 233.1151.

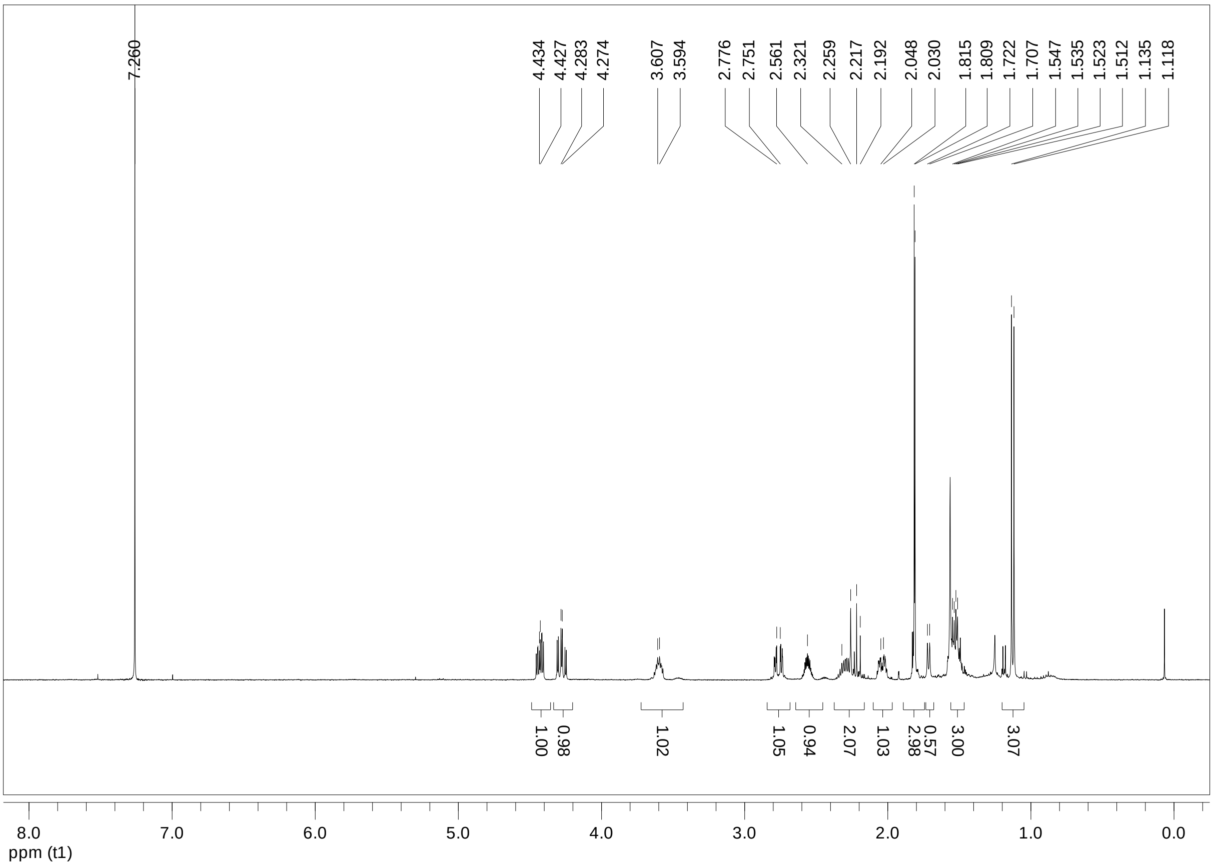

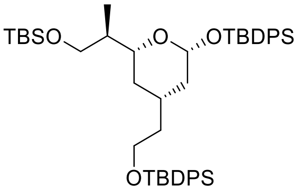

111

tert*-Butyl(((2*R*,4*R*,6*R*)-6-((*R*)-1-(*tert*-butyldimethylsilyloxy)propan-2-yl)-4-(2-((*tert*-butyldiphenylsilyl)oxy)ethyl)tetrahydro-2H-pyran-2-yl)oxy)diphenylsilane (111):** To a mechanically stirring solution of **57** (10.7 g, 33.8 mmol, 1.0 equiv) in 380 mL CH_2Cl_2 was added dropwise at –78 °C DIBAL (61.0 mL, 73.2 mmol, 2.2 equiv, 1.2 M in toluene) over 30 min. After the addition was complete, a thick colorless precipitate was formed. After stirring for 2 h at –78 °C, the reaction mixture was quenched by addition of sat. *Rochelle* salt (400 mL) and stirred over night at rt. The layers were separated and the aqueous layer was extracted with CH_2Cl_2 (2 x 150 mL). The combined organic extracts were dried over $MgSO_4$ and concentrated under reduced pressure. The crude hemiacetal (11.9 g) was used for the next step without further purification. **R*f = 0.59 (EtOAc).

A stirred solution of the above hemiacetal (11.9 g, crude) in dry CH_2Cl_2 (275 mL) was treated at rt with TBDPSCl (21.0 mL, 81.2 mmol, 2.4 equiv) followed by imidazole (5.85 g, 85.9 mmol, 2.5 equiv). After stirring at rt for 2.5 h, the reaction mixture was quenched with brine (200 mL) and diluted with CH_2Cl_2 (50 mL). The layers were separated and the aqueous layer was extracted with CH_2Cl_2 (3 x 100 mL). The combined organic extracts were dried over $MgSO_4$ and concentrated *in vacuo*. The residue was purified by column chromatography (hexane/Et_2O, 60:1 → 30:1) to give **111** (24.7 g, 1.4% wt/wt CH_2Cl_2, 24.3 g, 90% over 2 steps, single isomer) as a colorless oil.

R*f* = 0.45 (hexane/Et_2O, 30:1)

$[\alpha]_D^{24}$ = +3.0° [*c* = 0.415, $CHCl_3$]

^{1}H-NMR (400.1 MHz, $CDCl_3$): δ = 7.84-7.60 (m, 8H), 7.49-7.23 (m, 12H), 4.63 (d, *J* = 9.1 Hz, 1H), 3.69 (t, *J* = 6.2 Hz, 2H), 3.45 (dd, *J* = 5.2, 9.8 Hz, 1H), 3.35 (dd, *J* = 6.9, 9.6 Hz, 1H), 3.15 (dd, *J* = 4.7, 11.1 Hz, 1H), 1.82 (d, *J* = 12.5 Hz, 1H), 1.71-1.56 (m, 2H), 1.52 (dd, *J* = 6.4, 12.9 Hz, 2H), 1.42 (d, *J* = 12.9 Hz, 1H), 1.26-1.13 (m, 1H), 1.10 (s, 9H), 1.06 (s, 9H), 1.00-0.92 (m, 1H), 0.90 (d, *J* = 7.0 Hz, 3H), 0.87 (s, 9H), -0.02 (s, 6H).

^{13}C-NMR (100.6 MHz, $CDCl_3$): δ = 135.8, 135.6, 135.5, 134.0, 133.9, 129.6, 129.5, 129.4, 127.6, 127.5, 127.3, 97.0, 76.1, 65.7, 61.4, 40.8, 40.6, 39.3, 34.7, 31.0, 26.9, 26.8, 25.9, 19.2, 19.1, 18.2, 12.3, -5.4.

IR (neat, ν/cm^{-1}): 3072w, 2955m, 2930m, 2857m, 1472m, 1428m, 1389m, 1362w, 1264m, 1106s, 836m, 823m, 737s, 700s, 613m, 504s, 489s.

HRMS (ESI): *m/z* calcd for $C_{48}H_{74}NO_4Si_3$[M + NH_4]$^+$: 812.4920, found: 812.4921.

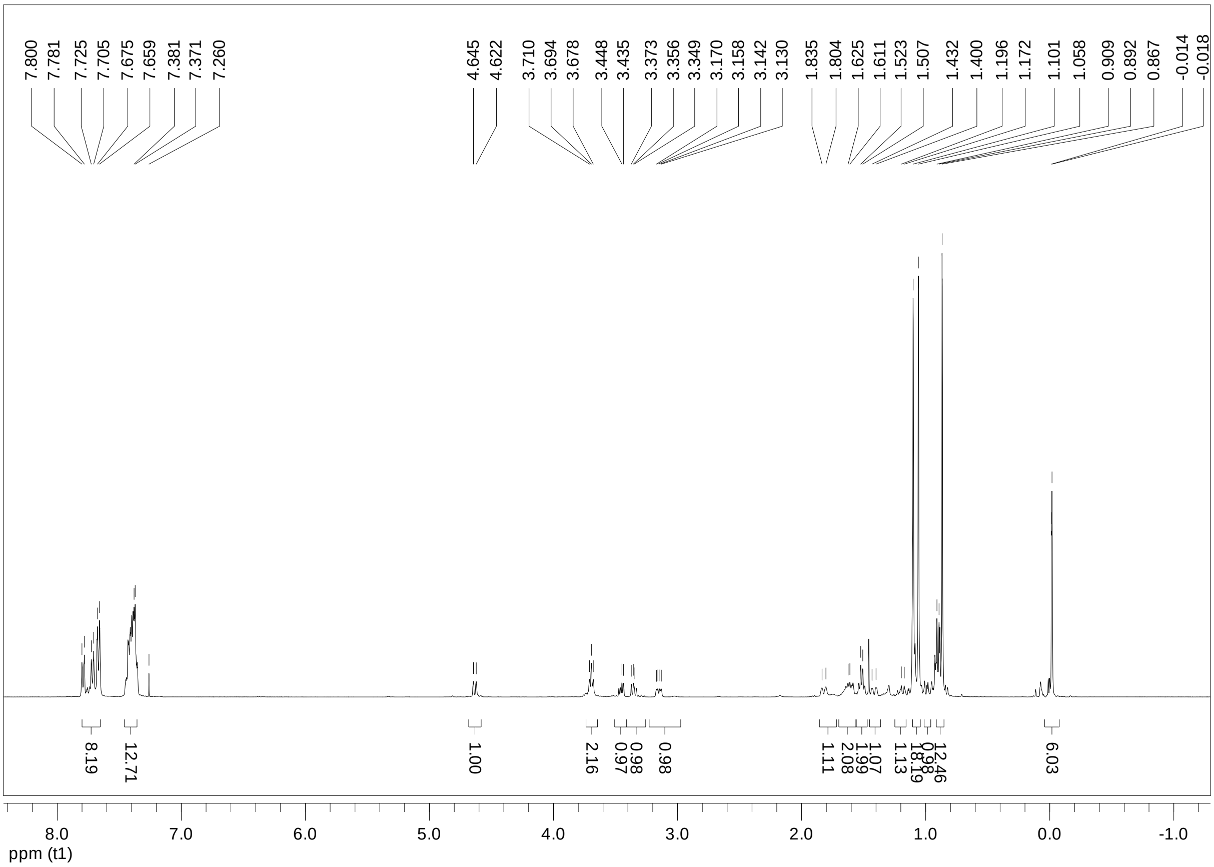
7.800
7.781
7.725
7.705
7.675
7.659
7.381
7.371
7.260
4.645
4.622
3.710
3.694
3.678
3.448
3.435
3.373
3.356
3.349
3.170
3.158
3.142
3.130
1.835
1.804
1.625
1.611
1.523
1.507
1.432
1.400
1.196
1.172
1.101
1.058
0.909
0.892
0.867
-0.014
-0.018
8.19
12.71
1.00
2.16
0.97
0.98
0.98
1.11
2.08
1.99
1.07
1.13
18.19
12.46
6.03
8.0
7.0
6.0
5.0
4.0
3.0
2.0
1.0
0.0
-1.0
ppm (t1)

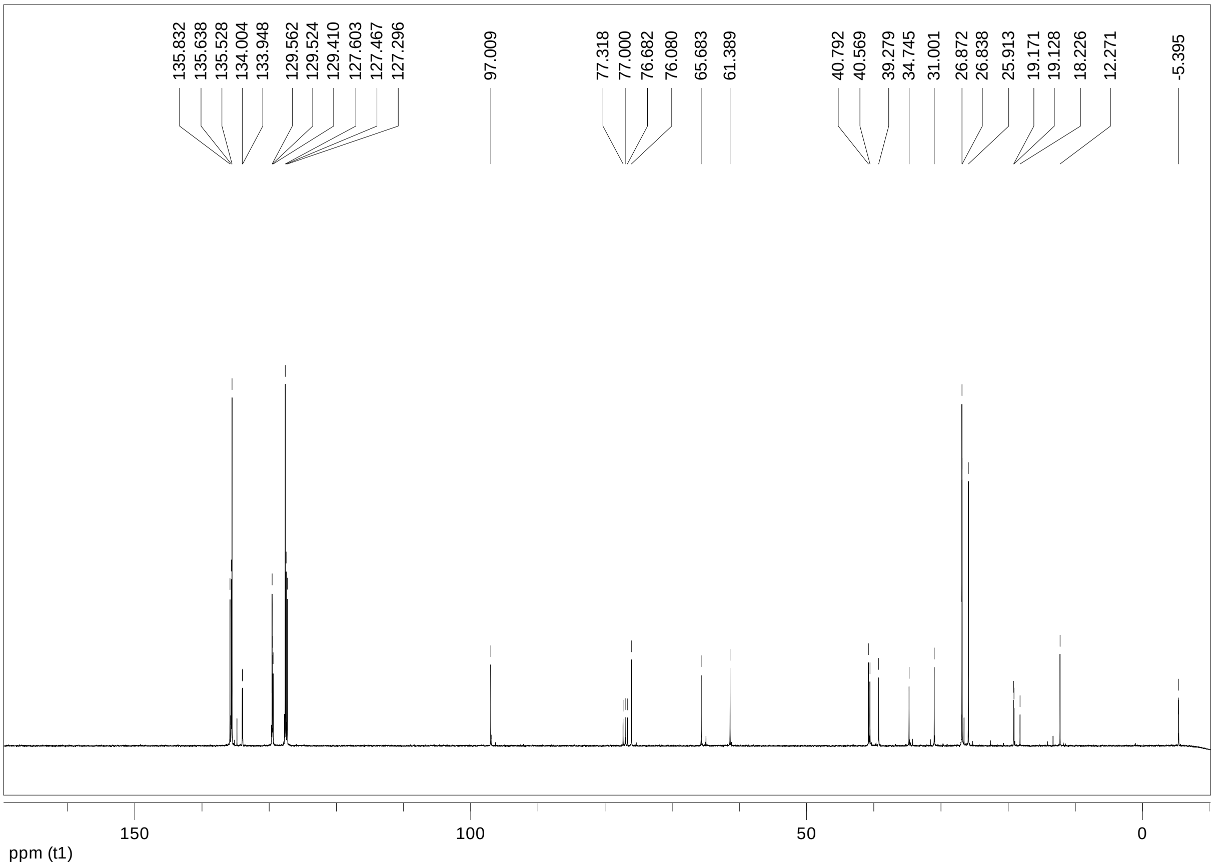
135.832
135.638
135.528
134.004
133.948
129.562
129.524
129.410
127.603
127.467
127.296
97.009
77.318
77.000
76.682
76.080
65.683
61.389
40.792
40.569
39.279
34.745
31.001
26.872
26.838
25.913
19.171
19.128
18.226
12.271
-5.395
150
100
50
0
ppm (t1)

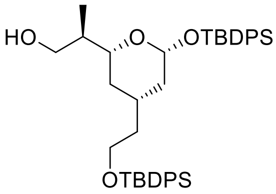

S32

(*R*)-2-((2*R*,4*R*,6*R*)-6-((*tert*-Butyldiphenylsilyl)oxy)-4-(2-((*tert*-butyldiphenyl-silyl)oxy)ethyl)tetrahydro-2H-pyran-2-yl)propan-1-ol (*S*32): To a solution of **111** (1.74 g, 2.19 mmol, 1.0 equiv) in THF/water (4:1, 22 mL) was added at rt sodium periodate (2.81 g, 13.1 mmol, 6.0 equiv). After stirring for 16 h at rt, the reaction mixture was quenched with water (50 mL) and diluted with CH_2Cl_2 (30 mL). The layers were separated and the aqueous layer was extracted with CH_2Cl_2 (2 x 50 mL). The combined organic extracts were dried over $MgSO_4$ and concentrated under reduced pressure. The residue was purified by column chromatography (hexane/EtOAc, 10:1 → 5:1 → 2:1) to give **S32** (1.30 g, 87%, 3% wt/wt CH_2Cl_2/EtOAc) as a colorless oil.

R_f = 0.20 (hexane/EtOAc, 10:1)
$[\alpha]_D^{24}$ = +0.1° [*c* = 0.420, $CHCl_3$].
^{1}H-NMR (400.1 MHz, $CDCl_3$): δ = 7.79-7.60 (m, 8H), 7.47-7.32 (m, 12H), 4.60 (dd, *J* = 2.1, 9.3 Hz, 1H), 3.68 (dd, *J* = 0.9, 6.2 Hz, 2H), 3.39 (dd, *J* = 7.9, 10.7 Hz, 1H), 3.32 (dd, *J* = 4.6, 10.8 Hz, 1H), 3.22 (ddd, *J* = 1.9, 3.6, 11.5 Hz, 1H), 1.91-1.78 (m, 2H), 1.76-1.56 (m, 2H), 1.51 (q, *J* = 6.4 Hz, 2H), 1.36-1.28 (m, 1H), 1.25-1.14 (m, 1H), 1.07 (s, 9H), 1.04 (s, 9H), 1.06-0.94 (m, 1H), 0.78 (d, *J* = 7.1 Hz, 3H).
^{13}C-NMR (100.6 MHz, $CDCl_3$): δ = 135.7, 135.6, 135.5, 133.9, 133.8, 133.7, 129.7, 129.6, 127.6, 127.4, 97.2, 77.0, 65.6, 61.3, 40.4, 39.2, 39.0, 33.0, 30.9, 26.9, 26.8, 19.2, 19.0, 11.5.
IR (neat, ν/cm^{-1}): 3427br, 3071w, 2930m, 2857m, 1472m, 1428m, 1389m, 1110s, 1026m, 938w, 902w, 822m, 804m, 739s, 701s, 613s, 505s.
HRMS (ESI): *m/z* calcd for $C_{42}H_{56}NaO_4Si_2$ [M + Na]$^+$: 703.3609, found: 703.3608.

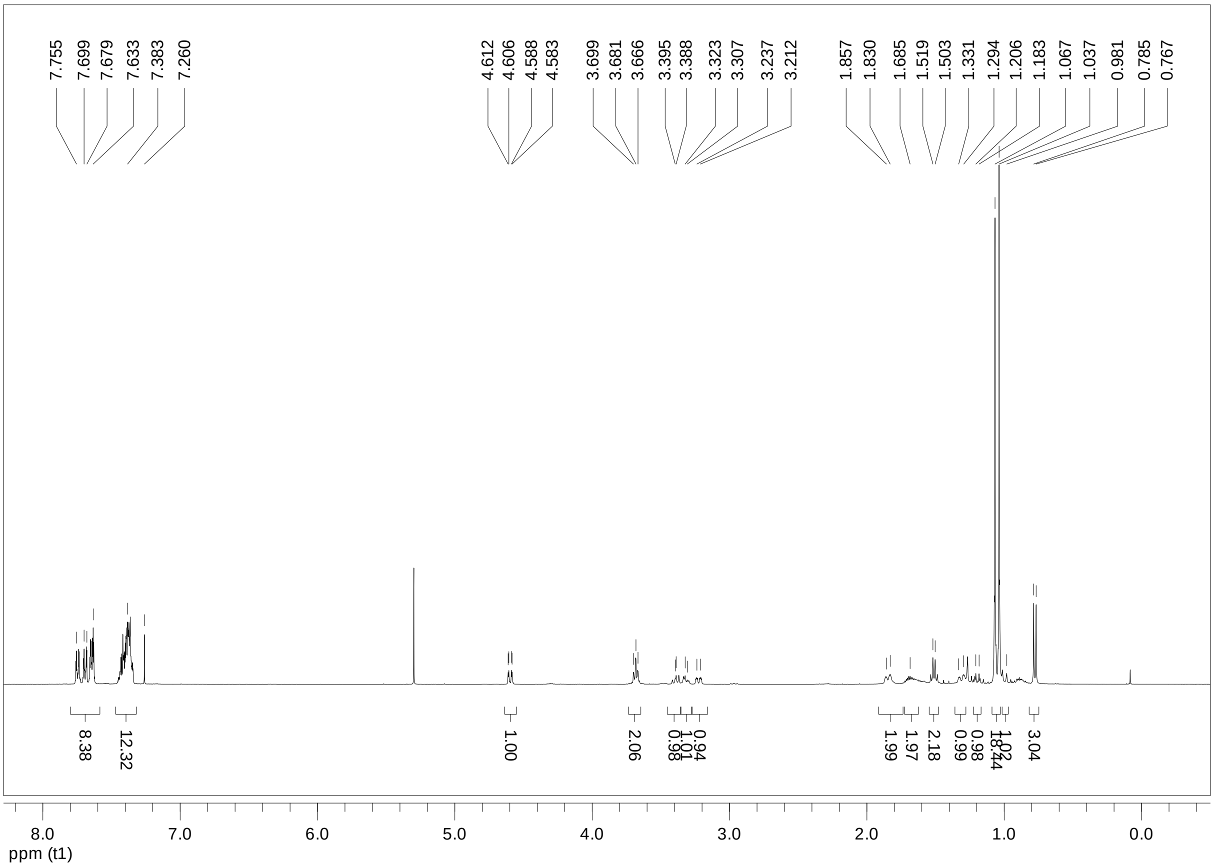

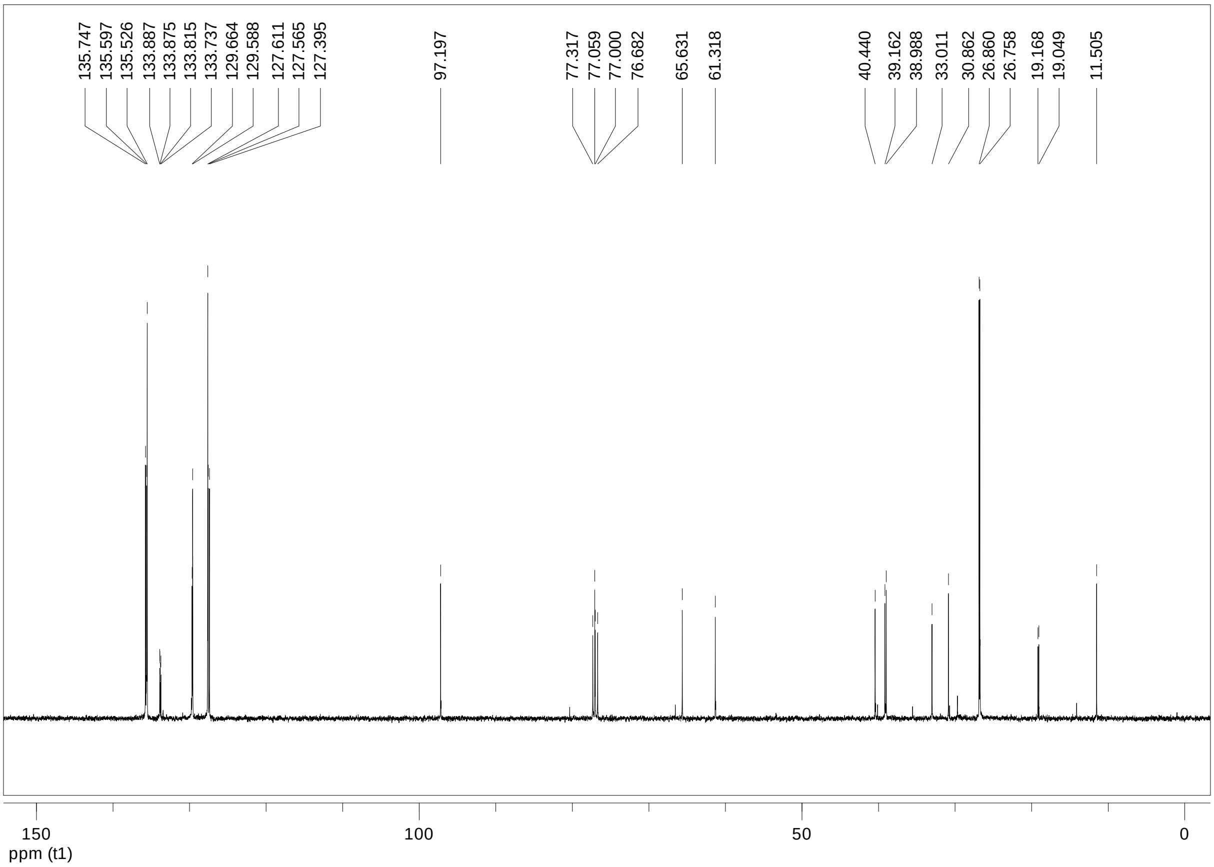

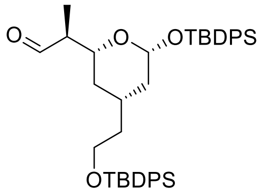

S33

(*S*)-2-((2*R*,4*R*,6*R*)-6-((*tert*-Butyldiphenylsilyl)oxy)-4-(2-((*tert*-butyldiphenylsilyl)oxy)ethyl)tetrahydro-2H-pyran-2-yl)propanal (S33):

***Dess-Martin* oxidation:** To a solution of **32** (90.2 mg, 0.13 mmol, 1.0 equiv) in CH_2Cl_2 (2.6 mL) was added at 0 °C *Dess-Martin* periodinane (67.4 mg, 0.16 mmol, 1.2 equiv) in one portion. After stirring for 1.5 h at 0 °C, sat. $Na_2S_2O_3$ (12 mL), sat. $NaHCO_3$ (12 mL) and Et_2O (25 mL) were added to the reaction mixture. The layers were separated and the aqueous layer was extracted with Et_2O (2 x 15 mL). The combined organic extracts were dried over $MgSO_4$, filtered and concentrated under reduced pressure. The residue was purified by column chromatography (hexane/EtOAc, 10:1) to give **S33** (87.2 mg, 9% wt/wt CH_2Cl_2, 79.3 mg, 88%) as a colorless oil.

***Swern* oxidation:** To a solution of oxalyl chloride (3.35 mL, 39.6 mmol, 1.5 equiv) in CH_2Cl_2 (300 mL) at –78 °C was added dropwise a solution of DMSO (5.63 mL, 79.3 mmol, 3.0 equiv) in CH_2Cl_2 (100 mL). After stirring at –78 °C for 20 min, a solution of **S32** (18.4 g, 2% wt/wt CH_2Cl_2/EtOAc, 18.0 g, 26.4 mmol, 1.0 equiv) in CH_2Cl_2 (180 mL) was added dropwise over 1 h. The resultant cloudy mixture was stirred at –78 °C for 1.5 h, and then TEA (14.7 mL, 106 mmol, 4.0 equiv) was added slowly and the reaction mixture was allowed to warm to room temperature (1.5 h). The reaction was quenched with water (350 mL), and the layers were separated. The aqueous phase was extracted with CH_2Cl_2 (3 x 60 mL) and the combined organic phases were dried over $MgSO_4$, filtered and concentrated *in vacuo*. The crude product was purified by column chromatography (hexane/EtOAc, 10:1) to afford **S33** (17.81 g, 3% wt/wt CH_2Cl_2/EtOAc, 17.26 g. 96%) as a colorless oil.

R*f* = 0.58 (hexane/EtOAc, 10:1)
[α]$_D^{24}$ = +16.4° [*c* = 0.540, $CHCl_3$]

^{1}H-NMR (400.1 MHz, $CDCl_3$): δ = 9.42 (d, *J* = 0.9 Hz, 1H), 7.75-7.58 (m, 8H), 7.47-7.30 (m, 12H), 4.62 (dd, *J* = 2.0, 9.3 Hz, 1H), 3.67 (dt, *J* = 2.6, 6.2 Hz, 2H), 3.46 (ddd, *J* = 1.7, 4.7, 11.4 Hz, 1H), 2.23 (m_c, 1H), 1.85 (d, *J* = 12.7 Hz, 1H), 1.68 (m_c, 1H), 1.49 (q, *J* = 6.4 Hz, 2H), 1.39 (d, *J* = 12.9 Hz, 1H), 1.25-1.13 (m, 1H), 1.06 (s, 9H), 1.03 (s, 9H), 1.00 (d, *J* = 7.1 Hz, 3H), 0.99-0.86 (m, 1H).
^{13}C-NMR (100.6 MHz, $CDCl_3$): δ = 204.2, 135.8, 135.6, 135.5, 133.8, 133.7, 129.6, 127.6, 127.5, 127.3, 97.0, 74.4, 61.1, 50.7, 40.2, 39.0, 34.2, 30.7, 26.9, 26.8, 19.2, 19.1, 8.6.
IR (neat, ν/cm^{-1}): 3071w, 2932m, 2858m, 1727m, 1469w, 1428m, 1389m, 1171w, 1109s, 1002w, 903w, 822m, 739m, 703s, 612m, 506s.
HRMS (ESI): *m/z* calcd for $C_{42}H_{54}NaO_4Si_2$ [M + Na]$^+$: 701.3453, found: 701.3455.

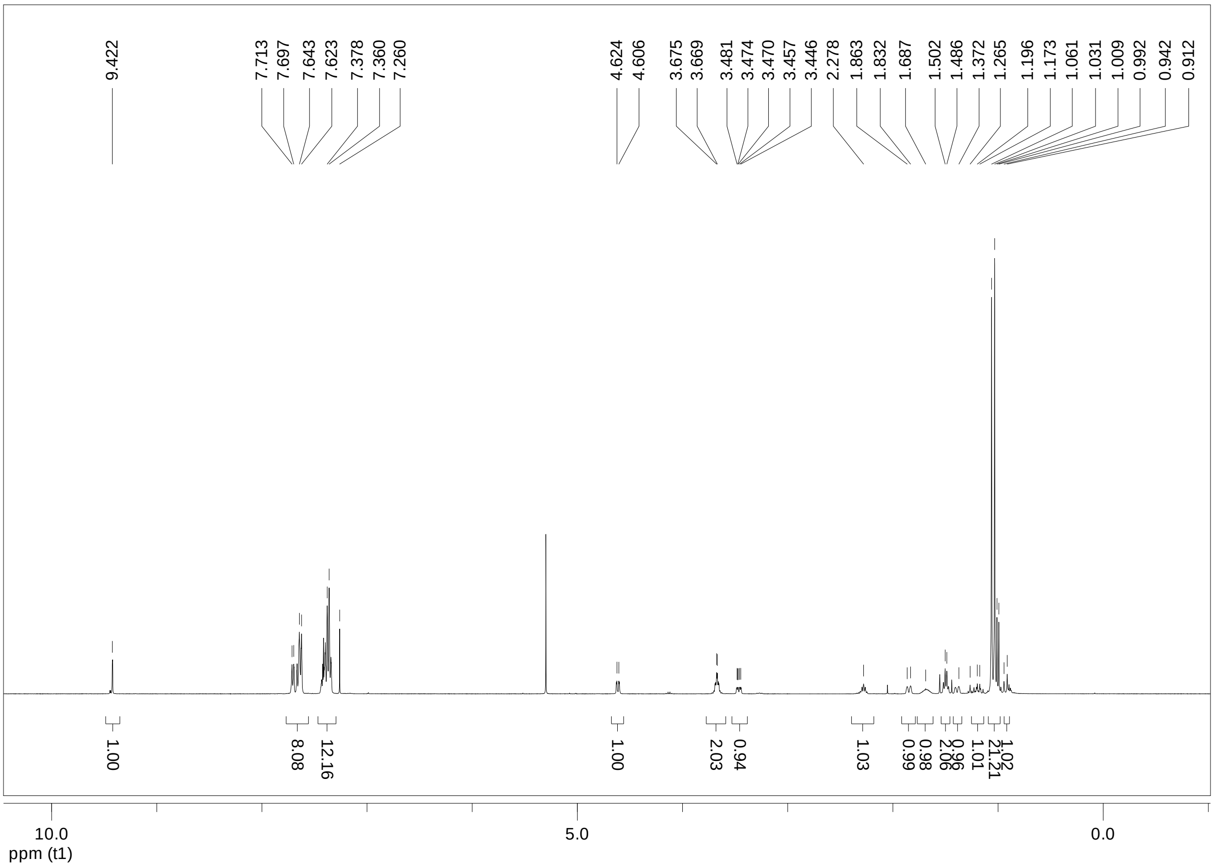
9.422
7.713
7.697
7.643
7.623
7.378
7.360
7.260
4.624
4.606
3.675
3.669
3.481
3.474
3.470
3.457
3.446
2.278
1.863
1.832
1.687
1.502
1.486
1.372
1.265
1.196
1.173
1.061
1.031
1.009
0.992
0.942
0.912
1.00
8.08
12.16
1.00
2.03
0.94
1.03
0.99
0.98
2.06
0.96
1.01
21.21
1.02
10.0
5.0
0.0
ppm (t1)

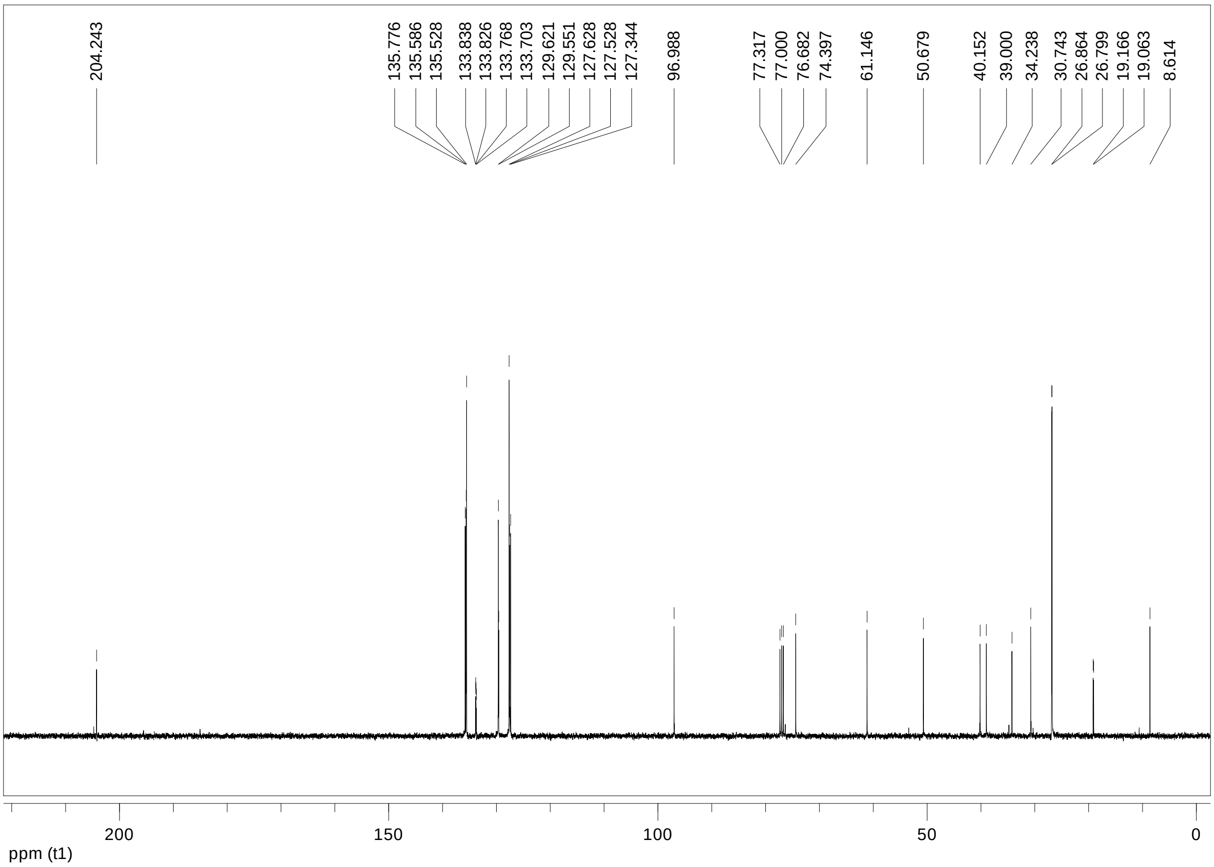
204.243
135.776
135.586
135.528
133.838
133.826
133.768
133.703
129.621
129.551
127.628
127.528
127.344
96.988
77.317
77.000
76.682
74.397
61.146
50.679
40.152
39.000
34.238
30.743
26.864
26.799
19.166
19.063
8.614
200
150
100
50
0
ppm (t1)

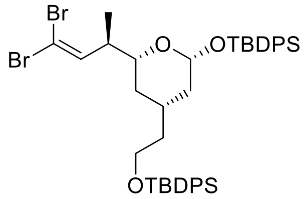

112

***tert*-Butyl(2-((2*R*,4*R*,6*R*)-2-((*tert*-butyldiphenylsilyl)oxy)-6-((*R*)-4,4-dibromobut-3-en-2-yl)tetrahydro-2H-pyran-4-yl)ethoxy)diphenylsilane (112):** To a solution of CBr_4 (69.7 mg, 0.21 mmol, 1.8 equiv) in CH_2Cl_2 (0.5 mL) was added at 0 °C triphenylphosphine (110 mg, 0.42 mmol, 3.6 equiv). After stirring for a few minutes at 0 °C, the yellow reaction mixture was cooled to −78 °C and a solution of **S33** (79.3 mg, 0.12 mmol, 1.0 equiv) in CH_2Cl_2 (3 x 0.5 mL) was added dropwise. After stirring for 1 h at the same temperature, the reaction mixture was diluted with sat. NH_4Cl (10 mL) and CH_2Cl_2 (10 mL). The layers were separated and the aqueous layer was extracted with CH_2Cl_2 (2 x 15 mL). The combined organic extracts were dried over $MgSO_4$, filtered and concentrated under reduced pressure. The crude product was purified by column chromatography (hexane/EtOAc, 30:1 → 20:1) to give **112** (97.5 mg, 1.2% wt/wt CH_2Cl_2, 96.3 mg, 99%) as a colorless oil.

R_f = 0.62 (hexane/EtOAc, 20:1)

$[\alpha]_D^{24}$ = +5.2° [*c* = 0.535, $CHCl_3$]

^{1}H-NMR (400.1 MHz, $CDCl_3$): δ = 7.83-7.60 (m, 8H), 7.50-7.32 (m, 12H), 6.07 (d, *J* = 9.6 Hz, 1H), 4.59 (d, *J* = 8.9 Hz, 1H), 3.68 (t, *J* = 5.9 Hz, 2H), 3.00 (dd, *J* = 6.0, 10.3 Hz, 1H), 2.47 (m_c, 1H), 1.83 (d, *J* = 12.6 Hz, 1H), 1.63 (brs, 1H), 1.57-1.43 (m, 2H), 1.38 (d, *J* = 12.7 Hz, 1H), 1.23-1.14 (m, 1H), 1.11 (s, 9H), 1.05 (s, 9H), 0.99-0.87 (m, 1H), 0.93 (d, *J* = 6.7 Hz, 3H).

^{13}C-NMR (100.6 MHz, $CDCl_3$): δ = 141.3, 135.8, 135.6, 135.5, 133.9, 133.7, 129.7, 129.6, 129.5, 127.6, 127.4, 97.1, 88.1, 76.8, 61.2, 43.2, 40.3, 39.0, 34.8, 30.7, 26.9, 26.8, 19.2, 19.1, 14.1.

IR (neat, ν/cm^{-1}): 3071w, 2932m, 2858m, 1469w, 1428m, 13893, 1110s, 1078s, 1003w, 823m, 739m, 703s, 612m, 505s.

HRMS (ESI): *m/z* calcd for $C_{43}H_{54}Br_2KO_3Si_2$ [M + K]$^+$: 871.1610, found: 871.1612.

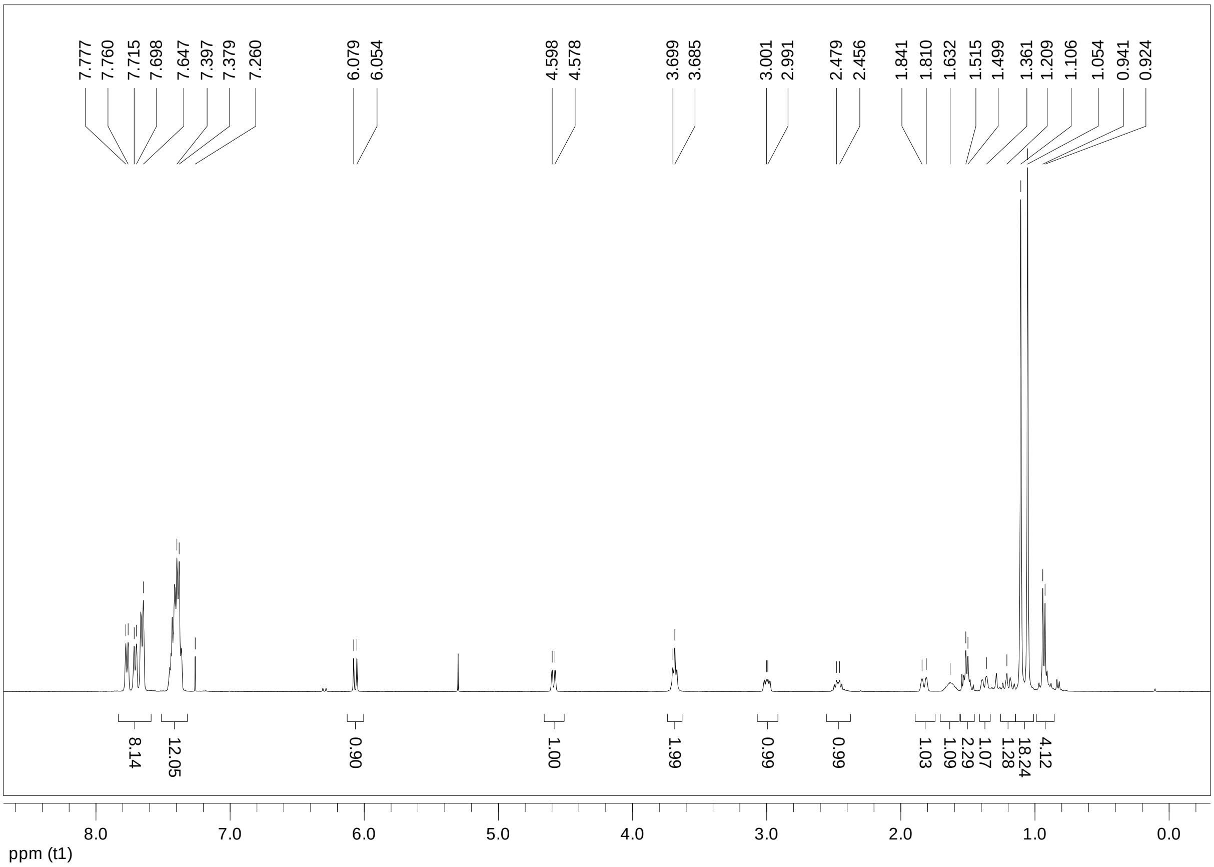

7.777
7.760
7.715
7.698
7.647
7.397
7.379
7.260
6.079
6.054
4.598
4.578
3.699
3.685
3.001
2.991
2.479
2.456
1.841
1.810
1.632
1.515
1.499
1.361
1.209
1.106
1.054
0.941
0.924
8.14
12.05
0.90
1.00
1.99
0.99
0.99
1.03
1.09
2.29
1.07
1.28
18.24
4.12
8.0
7.0
6.0
5.0
4.0
3.0
2.0
1.0
0.0
ppm (t1)

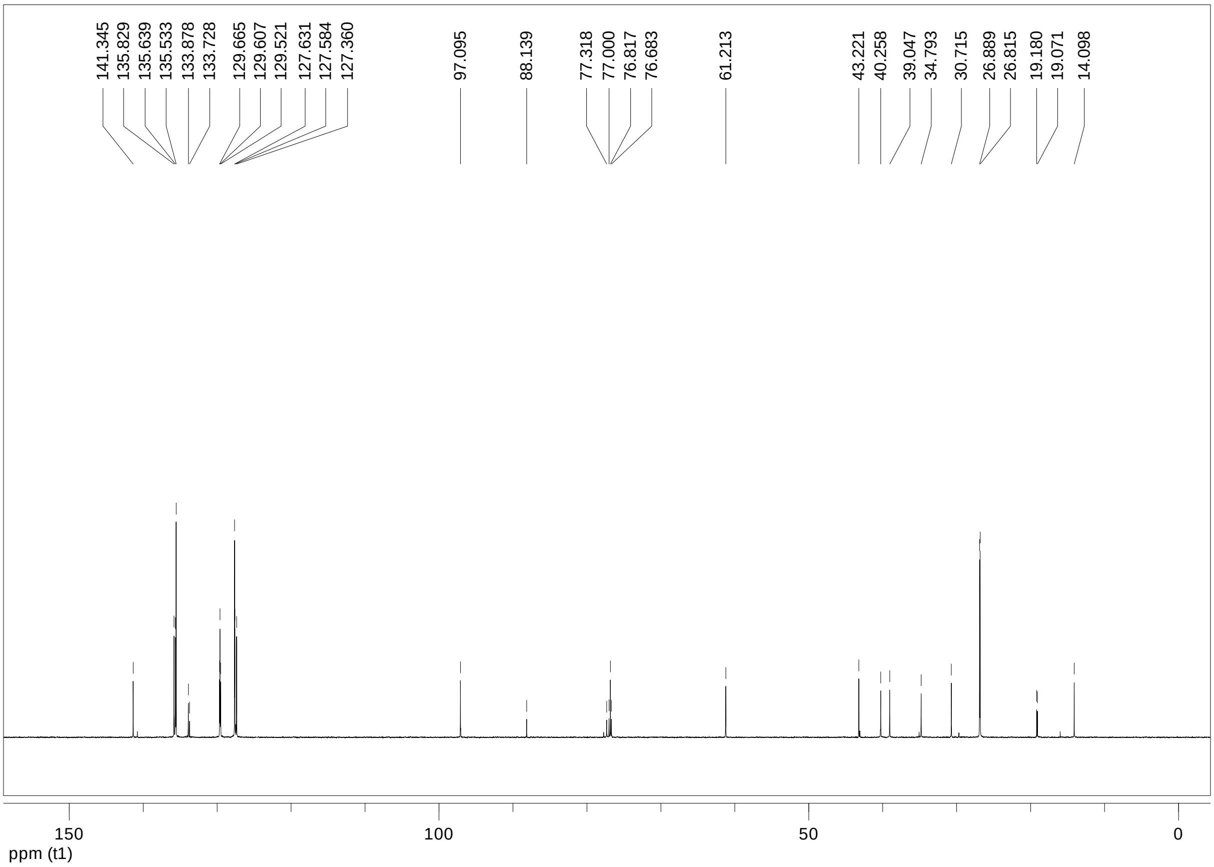

141.345
135.829
135.639
135.533
133.878
133.728
129.665
129.607
129.521
127.631
127.584
127.360
97.095
88.139
77.318
77.000
76.817
76.683
61.213
43.221
40.258
39.047
34.793
30.715
26.889
26.815
19.180
19.071
14.098
150
100
50
0
ppm (t1)

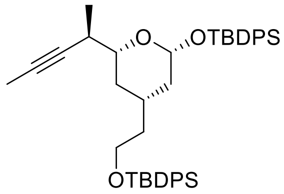

S34

***tert*-Butyl(2-((2*R*,4*R*,6*R*)-2-((*tert*-butyldiphenylsilyl)oxy)-6-((*R*)-pent-3-yn-2-yl)tetrahydro-2H-pyran-4-yl)ethoxy)diphenylsilane (*S*34):** To a solution of **112** (3.55 g, 2% wt/wt CH_2Cl_2/EtOAc, 3.48 g, 1.46 mmol. 1.0 equiv) in THF (24 mL) was added at -78 °C *n*-BuLi (8.8 mL, 14.1mmol, 3.4 equiv, 1.6 M in hexane). The reaction mixture was allowed to warm to rt over night in the cooling bath (over 16 h); then MeI (0.88 mL, 14.1 mmol, 3.4 equiv) was added at rt to the yellow reaction mixture. After stirring for 3.5 h at rt, the reaction mixture was poured into a sat. solution of NH_4Cl (200 mL) and was diluted with Et_2O (100 mL). The layers were separated and the organic layer was extracted with Et_2O (2 x 80 mL). The combined organic extracts were dried over $MgSO_4$ and concentrated *in vacuo*. The residue was purified by column chromatography (hexane/Et_2O, 70:1 → 30:1) to give **S34** (2.75 g, 2.2% wt/wt CH_2Cl_2, 2.69 g, 94%) as a colorless oil.

R_f = 0.44 (hexane/EtOAc, 30:1)

$[\alpha]_D^{24}$ = +10.3° [*c* = 0.580, $CHCl_3$]

^{1}H-NMR (400.1 MHz, $CDCl_3$): δ = 7.86-7.57 (m, 8H), 7.47-7.29 (m, 12H), 4.60 (dd, *J* = 2.0, 9.3 Hz, 1H), 3.68 (t, *J* = 6.3 Hz, 2H), 2.93 (m_c, 1H), 2.40 (m_c, 1H), 1.93 (d, *J* = 13.0 Hz, 1H), 1.80 (d, *J* = 12.7 Hz, 1H), 1.74 (d, *J* = 2.4 Hz, 3H), 1.63 (m_c, 1H), 1.51 (m_c, 2H), 1.27-1.13 (m, 1H), 1.09 (s, 9H), 1.08 (m_c, 3H), 1.04 (s, 9H), 0.93-0.79 (m, 1H).

^{13}C-NMR (100.6 MHz, $CDCl_3$): δ = 135.8, 135.6, 135.5, 134.0, 133.9, 133.7, 129.6, 129.5, 127.6, 127.5, 127.4, 97.1, 81.0, 78.7, 77.1, 61.4, 40.3, 39.1, 35.7, 32.0, 30.7, 26.9, 26.8, 19.2, 19.1, 18.1, 3.5.

IR (neat, ν/cm^{-1}): 3071w, 2931m, 2857m, 1472w, 1428m, 1389w, 1111s, 1078m, 1008w, 823m, 700s, 615m, 507s.

HRMS (ESI): *m/z* calcd for $C_{44}H_{56}NaO_3Si_2$ $[M + Na]^+$: 711.3660, found: 711.3670.

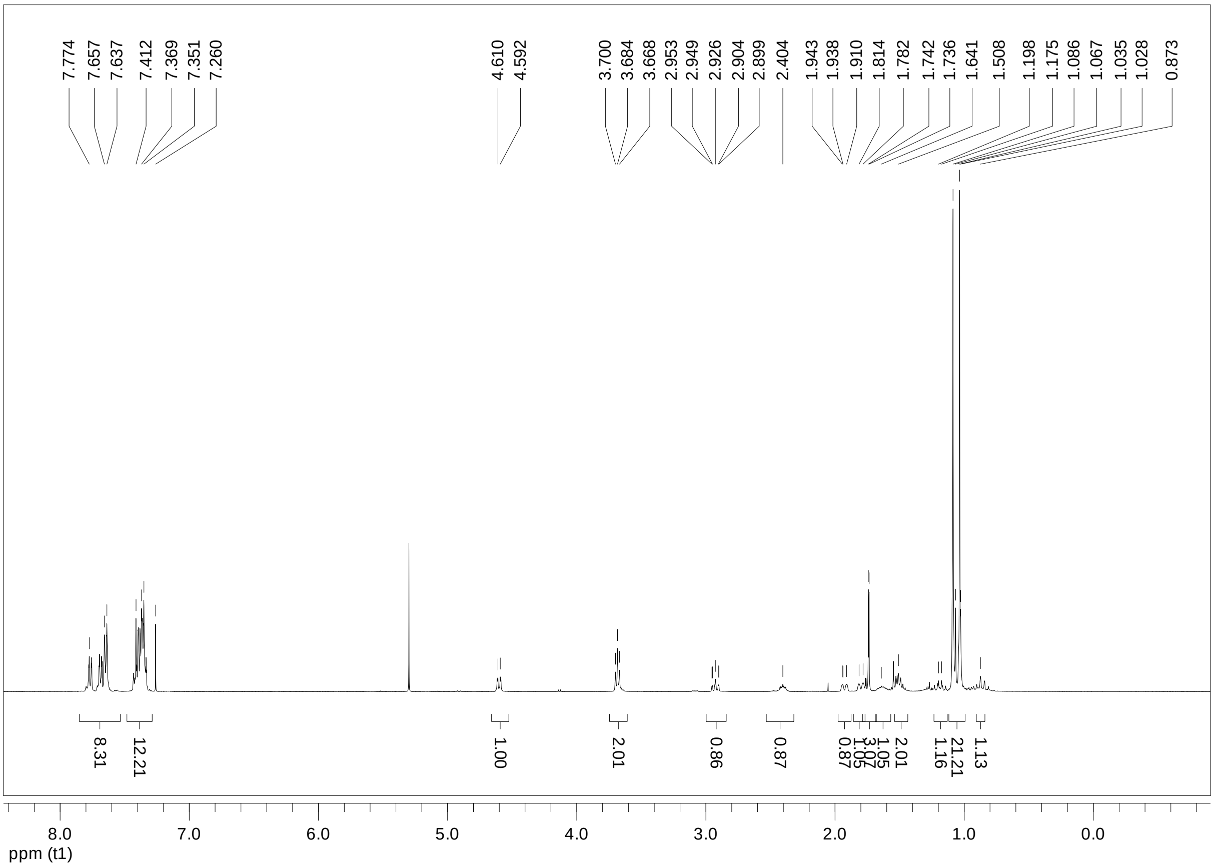
7.774
7.657
7.637
7.412
7.369
7.351
7.260
4.610
4.592
3.700
3.684
3.668
2.953
2.949
2.926
2.904
2.899
2.404
1.943
1.938
1.910
1.814
1.782
1.742
1.736
1.641
1.508
1.198
1.175
1.086
1.067
1.035
1.028
0.873
8.31
12.21
1.00
2.01
0.86
0.87
0.87
3.05
3.07
1.05
2.01
1.16
21.21
1.13
8.0
7.0
6.0
5.0
4.0
3.0
2.0
1.0
0.0
ppm (t1)

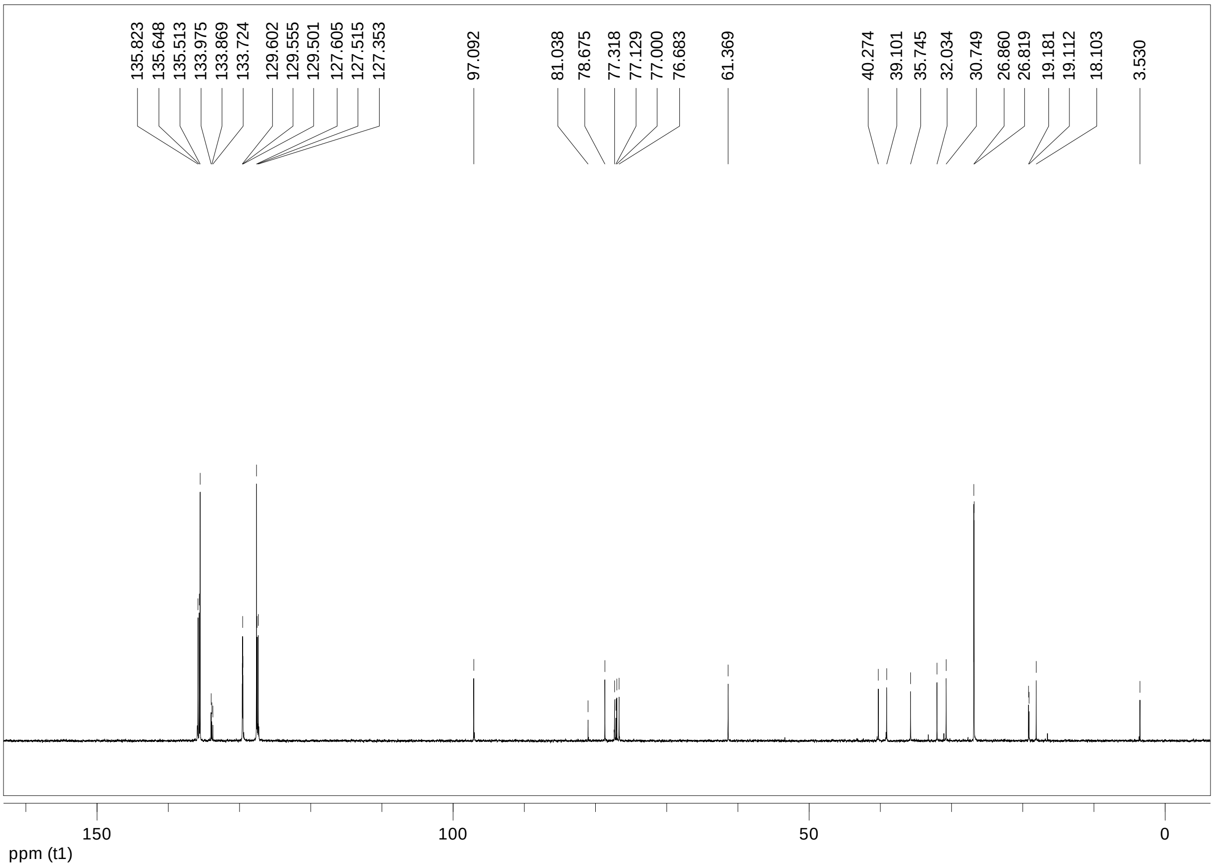
135.823
135.648
135.513
133.975
133.869
133.724
129.602
129.555
129.501
127.605
127.515
127.353
97.092
81.038
78.675
77.318
77.129
77.000
76.683
61.369
40.274
39.101
35.745
32.034
30.749
26.860
26.819
19.181
19.112
18.103
3.530
150
100
50
0
ppm (t1)

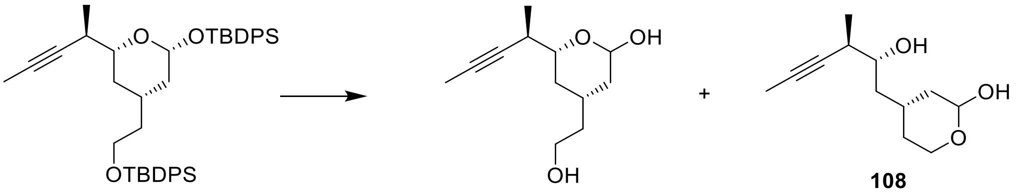

S34 107 108

(4*R*,6*R*)-4-(2-Hydroxyethyl)-6-((*R*)-pent-3-yn-2-yl)tetrahydro-2H-pyran-2-ol (107) and (4*R*)-4-((2*R*,3*R*)-2-hydroxy-3-methylhex-4-yn-1-yl)tetrahydro-2H-pyran-2-ol (108): To a solution of **S34** (5.53 g, 8.03 mmol, 1.0 equiv) in THF (83 mL) was added at 0 °C AcOH (1.61 mL, 28.1 mmol, 3.5 equiv) and TBAF (28.0 mL, 28.0 mmol, 3.5 equiv, 1.0 M in THF). After stirring for 16 h at rt, the reaction mixture was diluted with EtOAc and quenched at 0 °C with sat. $NaHCO_3$ (60 mL). The layers were separated and the aqueous layer was extracted with EtOAc (3 x 40 mL). The combined organic extracts were dried over $MgSO_4$ and the solvent was removed under reduced pressure. The residue was purified by column chromatography (hexane/EtOAc, 1:1 → 0:1) to give **107/108** (1.84 g, including residual solvent) as a colorless oil.

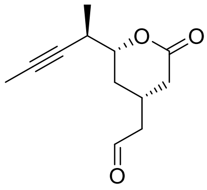

109

2-((4*S*,6*R*)-2-Oxo-6-((*R*)-pent-3-yn-2-yl)tetrahydro-2H-pyran-4-yl)acetaldehyde (109): To a solution of **107/108** (1.42 g, 7.2% wt/wt solvent, 1.32 g, 6.20 mmol, 1.0 equiv) in CH_2Cl_2 (84 mL) over molecular sieves (6.54 g powder, 4 Å) was added at rt NMO (2.01 g, 14.9 mmol, 2.4 equiv). After stirring for 20 min at rt, TPAP (392 mg, 1.12 mmol, 18 mol%) was added at 0 °C in one portion and stirring was continued for 40 min at rt. The reaction mixture was directly purified by column chromatography (hexane/EtOAc, 2:1 → 1:1) to give **109** (652 mg, 99% wt/wt CH_2Cl_2, 646 mg, 50% over 2 steps) as a colorless oil. The analytical data were identical to those reported (*vide supra*).

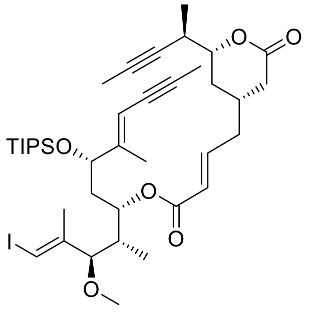

113

(1*E*,3*R*,4*S*,5*S*,7*S*,8*E*)-1-Iodo-3-methoxy-2,4,8-trimethyl-7-((triisopropylsilyl)oxy)-dodeca-1,8-dien-10-yn-5-yl (E)-4-((4R,6R)-2-oxo-6-((R)-pent-3-yn-2-yl)tetra-hydro-2H-pyran-4-yl)but-2-enoate (113): To LiCl (99.0 mg, 2.34 mmol, 1.05 eq) was added a solution of phosphonate **101** (1.62 g, 2.23 mmol, 1.00 eq) in MeCN (15 mL), followed by DBU (349 µL, 2.24 mmol, 1.05 eq). After 20 min stirring at rt, the pale yellow turbid mixture was cooled to 0 °C. After 25 min, a solution of aldehyde **109** (556 mg, 2.67 mmol, 1.20 eq) in THF (17 mL) was added. The cooling bath was removed and the resulting yellow solution was stirred at rt for 3 h. Then the mixture was concentrated *in vacuo* and the residue was purified by column chromatography (hexane/EtOAc 5:1→1:1) to afford diyne **113** (1.44 g, 98% wt/wt along with Et_2O and CH_2Cl_2, 1.41 g, 81%) as a colourless oil. In addition, part of phosphonate **101** was recovered (116 mg, 94% wt/wt along with ether and EtOAc, 110 mg, 7%).

TLC (hexane/EtOAc, 5:1): R_f = 0.24

[α]$_D^{24}$ = +23.6° [*c* = 0.905, $CHCl_3$]

^{1}H-NMR (400.1 MHz, $CDCl_3$): δ = 6.81 (td, *J* = 7.3, 15.6 Hz, 1H), 6.15 (s, 1H), 5.83 (d, *J* = 15.6 Hz, 1H), 5.33 (brs, 1H), 4.77 (ddd, *J* = 1.5, 4.0, 10.1 Hz, 1H), 4.12 (t, *J* = 6.5 Hz, 1H), 4.06 (ddd, *J* = 3.0, 7.2, 11.4 Hz, 1H), 3.37 (d, *J* = 7.0 Hz, 1H), 3.15 (s, 3H), 2.77-2.61 (m, 2H), 2.32-2.18 (m, 3H), 2.17-2.00 (m, 3H), 1.95 (d, *J* = 2.1 Hz, 3H), 1.88-1.76 (m, 1H), 1.82 (d, *J* = 0.8 Hz, 3H), 1.79 (s, 3H), 1.78 (d, *J* = 2.3 Hz, 3H), 1.74-1.64 (m, 1H), 1.42-1.29 (m, 1H), 1.23 (d, *J* = 7.0 Hz, 3H), 1.02 (brs, 21H), 0.90 (d, *J* = 6.9 Hz, 3H).

^{13}C-NMR (100.6 MHz, $CDCl_3$): δ = 170.2, 165.3, 150.9, 146.4, 144.2, 124.2, 106.5, 89.4, 87.5, 82.5, 79.0, 78.9, 78.5, 76.9, 75.2, 72.5, 56.7, 38.8, 38.7, 36.1, 35.9, 32.3, 32.0, 30.7, 19.7, 18.2, 18.1, 17.5, 14.0, 12.4, 9.7, 4.5, 3.5.

IR (neat, ν/cm^{-1}): 2941m, 2922m, 2866m, 1737s, 1718s, 1656w, 1462m, 1381m, 1246m, 1085s, 1059s, 1005m, 884m, 680m.

HRMS (ESI): *m/z* calcd for $C_{39}H_{65}INO_6Si$ [M + NH_4]$^+$: 798.3620; found: 798.3632.

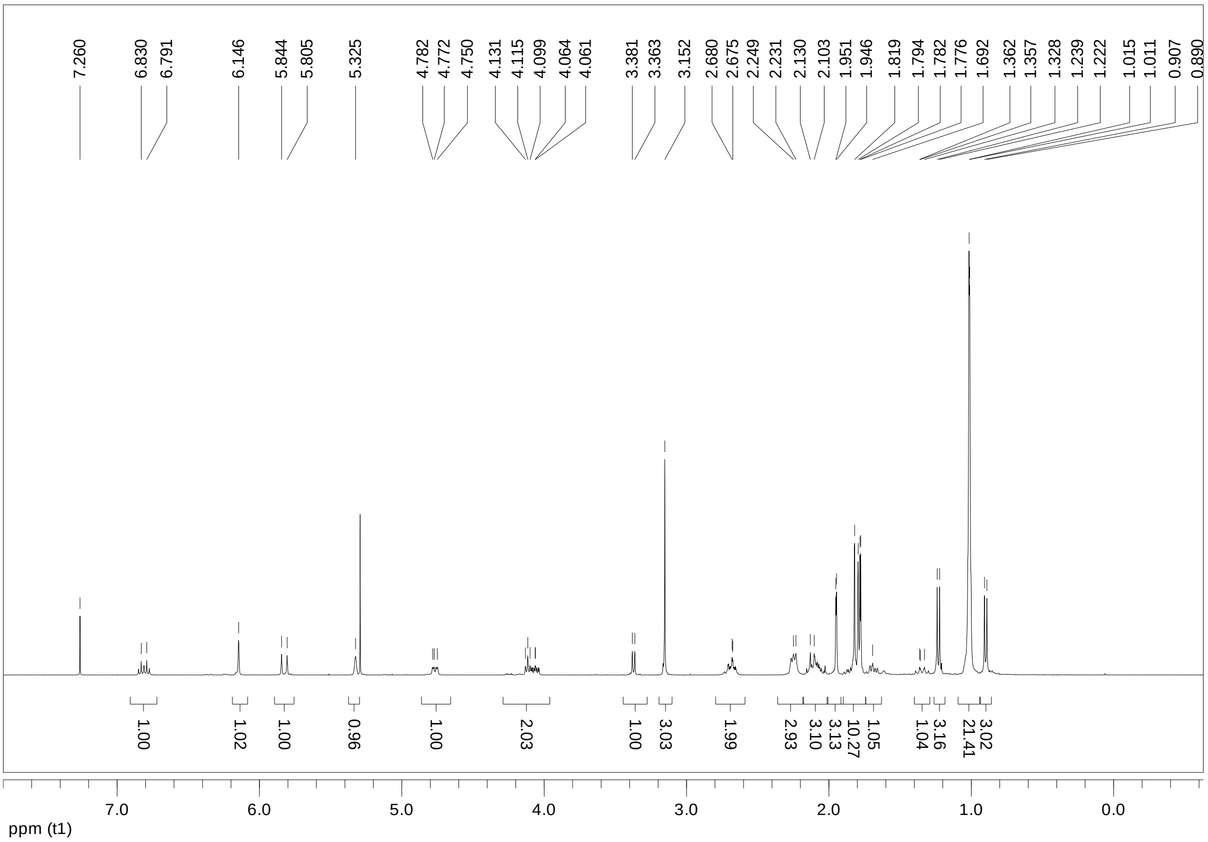

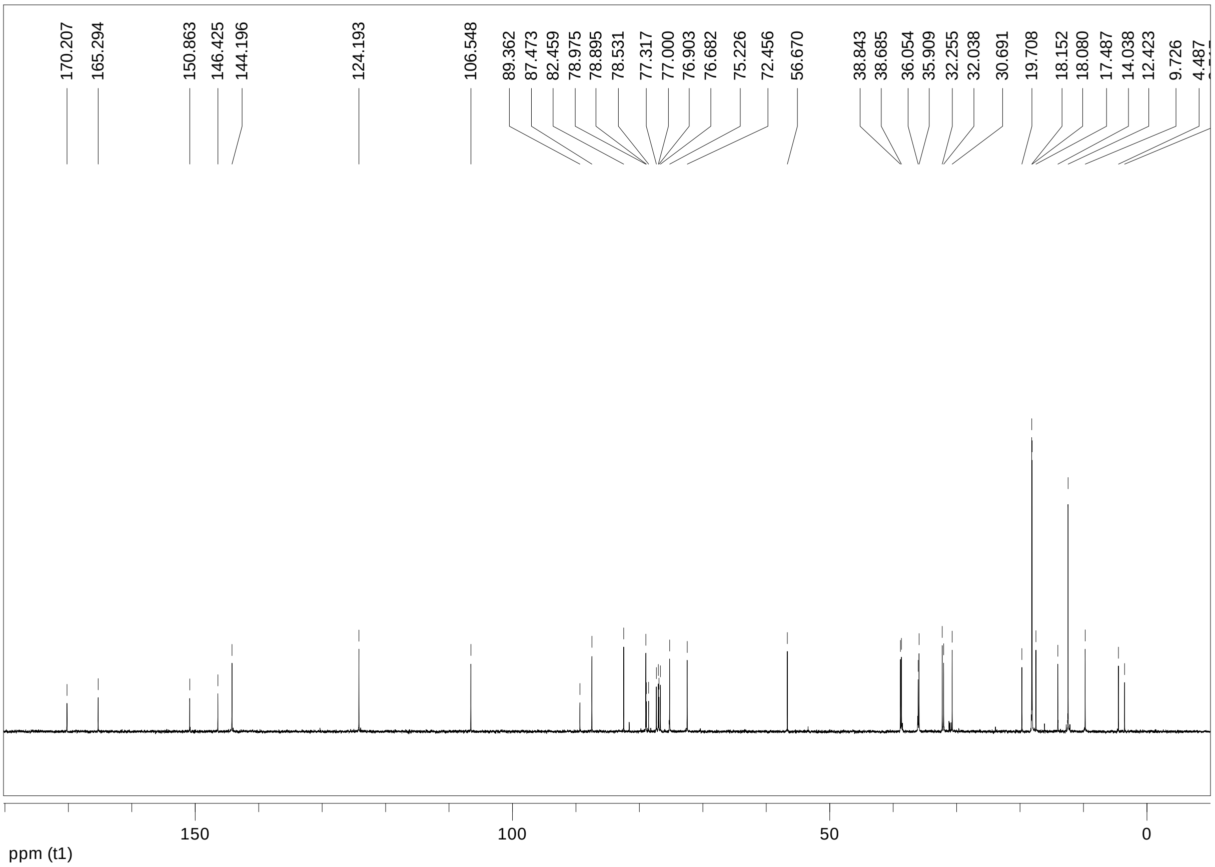

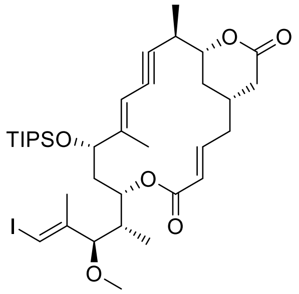

116

(1*R*,3*E*,7*S*,9*S*,12*E*,14*R*,15*R*)-7-((2*S*,3*R*,*E*)-5-Iodo-3-methoxy-4-methylpent-4-en-2-yl)-10,14-dimethyl-9-((triisopropylsilyl)oxy)-6,16-dioxabicyclo[13.3.1]nonadeca-3,12-diene-10-yn-5,17-dione (116):

Using Mo-complex **114**: A suspension of the Mo-complex **114** (37.3 mg, 31.3 μmol, 35 mol%) and $MnCl_2$ (3.94 mg, 31.3 μmol, 35 mol%) in toluene (1.86 mL) was heated to 80 °C for 30 min. Then an aliquot corresponding to 10 mol% catalyst was used.
To a suspension of the diyne **113** (34.9 mg, 44.7 μmol, 1.00 eq) and molecular sieves (255 mg, 5 Å powdered) in toluene (9.5 mL) was added the above catalyst solution (10 mol%) at rt and the mixture was heated to reflux (125 °C oil bath) for 2 h 45 min; at this point reaction monitoring by ESI-MS indicated full conversion. The mixture was then allowed to cool to rt and stored in a freezer (−18 °C) overnight. It was then filtered through a short pad of silica (in a 20 mL syringe), which was rinsed thoroughly with EtOAc. The combined filtrates were concentrated under reduced pressure and the residue was purified by column chromatography (hexane/EtOAc 4:1→3:1) to afford the macrocyclic dienyne **116** (22.5 mg, 69%) as a pale brown foam.

Using Mo-complex **115**: A suspension of the Mo-complex **115** (165 mg, 135 μmol, 15 mol%) and $MnCl_2$ (17.0 mg, 135 μmol, 15 mol%) in toluene (3 mL) was heated to 80 °C for 30 min and then added *via* cannula (rinsed with 2×1 mL) to a suspension of the diyne **113** (705 mg, 903 μmol, 1.00 eq) and molecular sieves (5.2 g, 5 Å powdered) in toluene (150 mL); the latter had been prestirred at rt for 20 min. The resulting mixture was then heated to 125 °C for 27 h with stirring (oil bath). Regular reaction monitoring by ESI-MS indicated the reaction to proceed very slowly and not go to completion. The mixture was filtered through a pad of silica, which was rinsed thoroughly with EtOAc. The combined

filtrates were concentrated under reduced pressure and the crude product was purified by column chromatography (hexane/EtOAc 3:1) to afford the macrocyclic dienyne **116** (365 mg, 56%). In addition, a mixture containing the starting material, side product **S35** and the desired product **116** (94.9 mg, ca. 3.6/2.2/1 **113/S35/116**) was isolated.

In a second experiment conducted on the same scale of **113**, pure **116** was obtained in 31% yield (204 mg) as a bright brown solid. In addition, two mixed fractions of **116/S35** (76.5 mg, containing 48.8 mg of **116**) and of **115/S35** (179 mg) were isolated. The reasons for the discrepancy between the two experiments, which were performed under ostensibly identical conditions, is unclear.

TLC (hexane/EtOAc, 1:1): R_f = 0.55
$[\alpha]_D^{24}$ = −13.9 (*c* = 0.83, $CHCl_3$)
^{1}H-NMR (500.1 MHz, $CDCl_3$): δ = 6.79 (ddd, *J* = 5.6 Hz, *J* = 10.2 Hz, *J* = 15.8 Hz, 1H), 6.14 (d, *J* = 0.7 Hz, 1H), 5.73 (d, *J* = 16.0 Hz, 1H), 5.27 (m_c, 1H), 4.40 (dd, *J* = 3.2 Hz, *J* = 10.0 Hz, 1H), 4.03-3.93 (m, 2H), 3.34 (d, *J* = 8.5 Hz, 1H), 3.17 (s, 3H), 2.80 (m_c, 1H), 2.72 (ddd, *J* = 2.2 Hz, *J* = 5.0 Hz, *J* = 17.6 Hz, 1H), 2.55-2.46 (m, 1H), 2.41 (ddd, *J* = 3.2 Hz, *J* = 5.6 Hz, *J* = 14.0 Hz, 1H), 2.22 (dd, *J* = 11.5 Hz, *J* = 17.6 Hz, 1H), 2.22-2.14 (m, 1H), 2.10-1.86 (m, 3H), 1.89 (d, *J* = 1.0 Hz, 3H), 1.82 (d, *J* = 1.0 Hz, 3H), 1.71 (dd, *J* = 4.1 Hz, *J* = 14.8 Hz, 1H), 1.27 (d, *J* = 6.9 Hz, 3H), 1.24-1.11 (m, 1H), 1.09-0.99 (m, 21H), 0.96 (d, *J* = 6.8 Hz, 3H). **^{13}C-NMR** (125.8 MHz, $CDCl_3$): δ = 170.2, 165.2, 152.4, 147.4, 145.0, 125.2, 106.5, 93.3, 88.4, 83.1, 81.9, 79.0, 77.3, 72.8, 56.5, 38.8, 37.2, 36.3, 35.2, 34.0, 31.3, 29.9, 19.0, 18.1 (2C), 18.0, 17.0, 13.6, 12.3 (6C), 9.7.
IR (neat, ν/cm^{-1}): 2959, 2940, 2891, 2866, 1737 (s), 1716 (s), 1652, 1461, 1384, 1323, 1258, 1219, 1185, 1158, 1122, 1082 (s), 1059 (s), 1011, 997, 984, 961, 884, 849, 800, 785, 756, 684, 602, 533, 517, 476, 461, 444.
HRMS (ESI): *m/z* calcd for $C_{35}H_{55}INaO_6Si$ $[M+Na]^+$: 749.2705; found: 749.2723.

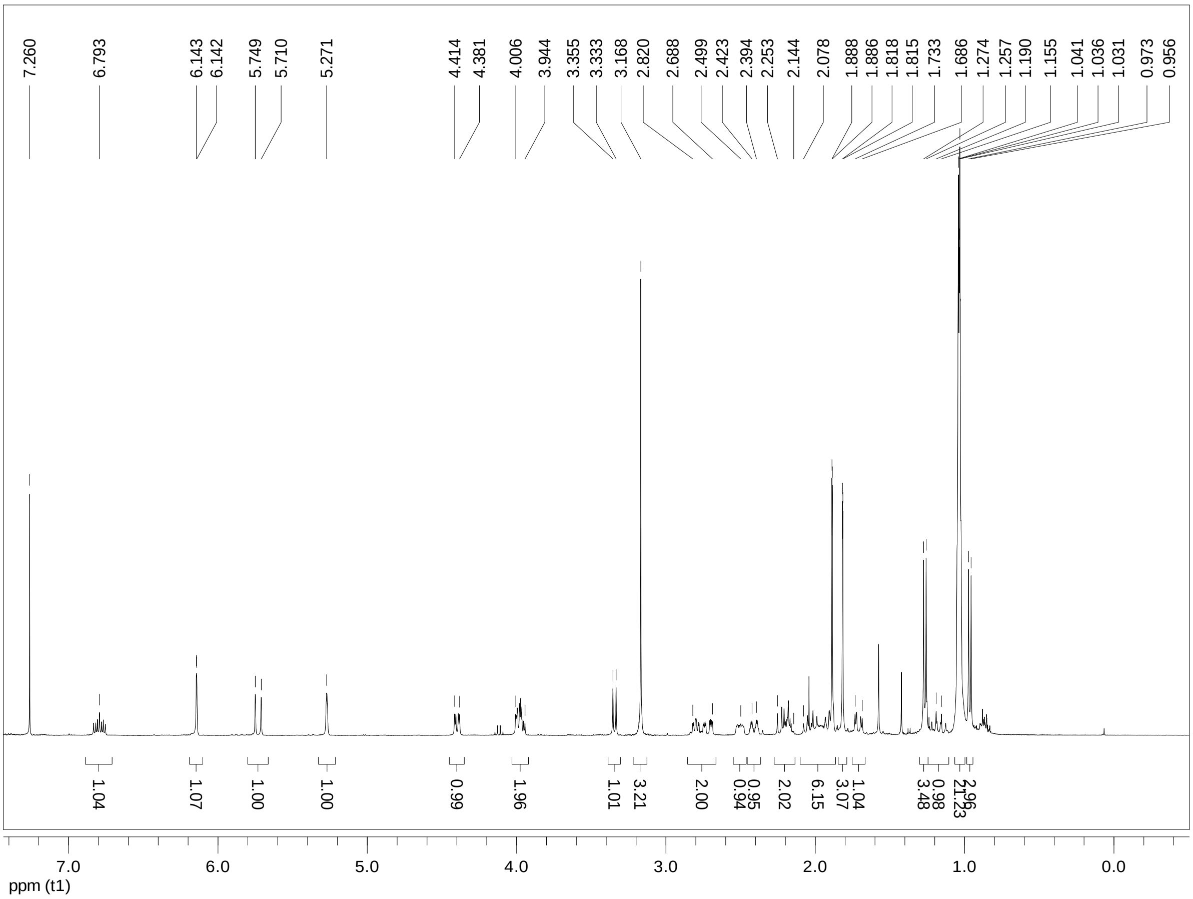
7.260
6.793
6.143
6.142
5.749
5.710
5.271
4.414
4.381
4.006
3.944
3.355
3.333
3.168
2.820
2.688
2.499
2.423
2.394
2.253
2.144
2.078
1.888
1.886
1.818
1.815
1.733
1.686
1.274
1.257
1.190
1.155
1.041
1.036
1.031
0.973
0.956
1.04
1.07
1.00
1.00
0.99
1.96
1.01
3.21
2.00
0.94
0.95
2.02
6.15
3.07
1.04
3.48
0.98
21.23
2.96
7.0
6.0
5.0
4.0
3.0
2.0
1.0
0.0
ppm (t1)

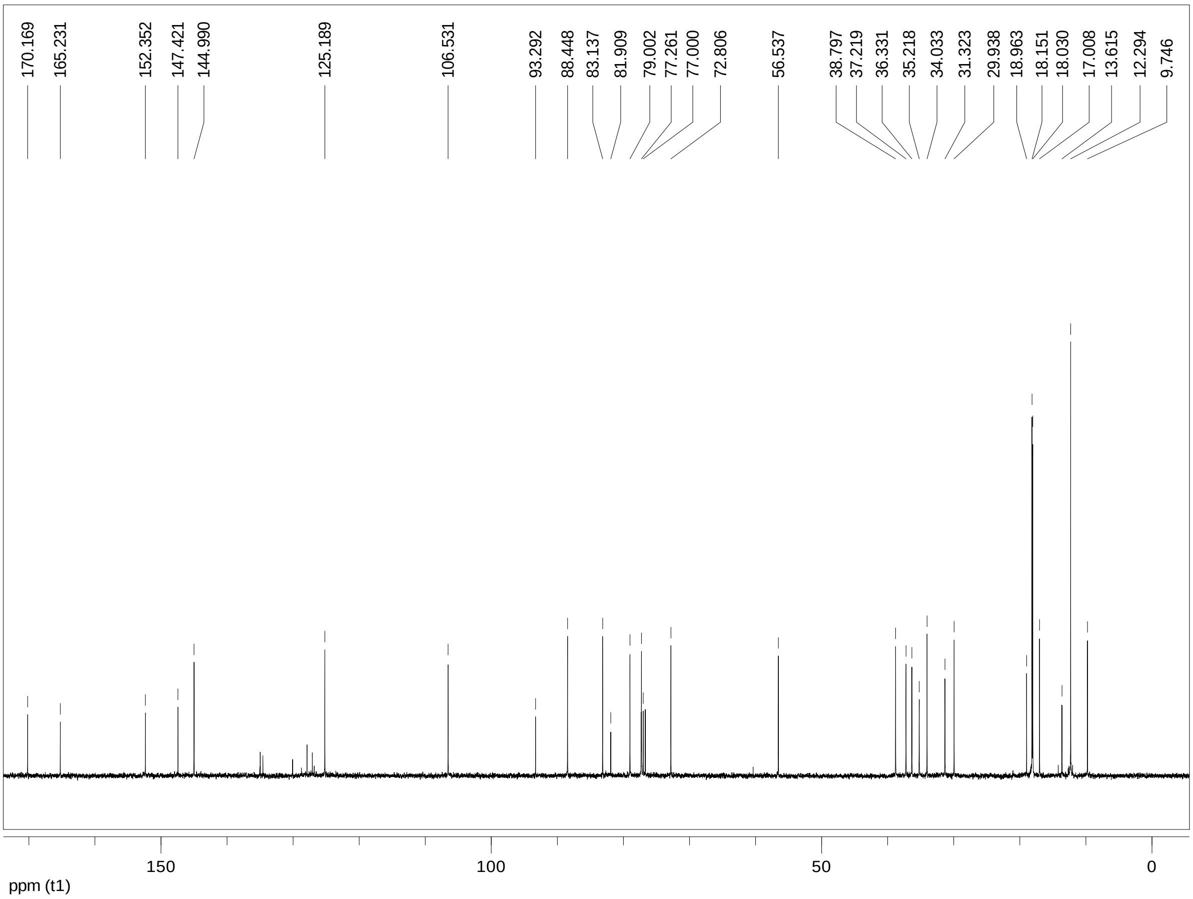
170.169
165.231
152.352
147.421
144.990
125.189
106.531
93.292
88.448
83.137
81.909
79.002
77.261
77.000
72.806
56.537
38.797
37.219
36.331
35.218
34.033
31.323
29.938
18.963
18.151
18.030
17.008
13.615
12.294
9.746
150
100
50
0
ppm (t1)

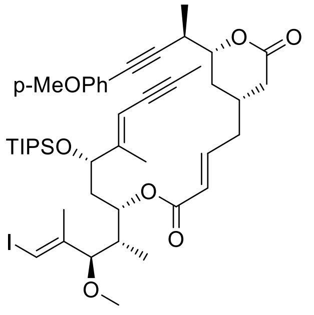

S35

(1*E*,3*R*,4*S*,5*S*,7*S*,8*E*)-1-Iodo-3-methoxy-2,4,8-trimethyl-7-((triisopropylsilyl)-oxy)dodeca-1,8-dien-10-yn-5-yl (*E*)-4-((4R,6R)-2-oxo-6-((*R*)-4-(p-methoxy)-phenylbut-3-yn-2-yl)tetrahydro-2H-pyran-4-yl)but-2-enoate (S35):

R*f* = 0.33 (hexane/EtOAc, 3:1)

[α]$_D^{24}$ = +37.6° [*c* = 0.125, $CHCl_3$]

^{1}H-NMR (500.1 MHz, $CDCl_3$): δ = 7.34-7.28 (m, 2H), 6.86-6.77 (m, 3H), 6.16 (s, 1H), 5.80 (d, *J* = 15.6 Hz, 1H), 5.11 (s, 1H), 4.97 (t, *J* = 6.8 Hz, 1H), 4.79 (m_c, 1H), 4.19 (m_c, 1H), 3.79 (s, 3H), 3.38 (d, *J* = 7.3 Hz, 1H), 3.16 (s, 3H), 2.96 (m_c, 1H), 2.76-2.63 (m, 1H), 2.35-2.19 (m, 3H), 2.19-2.07 (m, 3H), 1.93 (d, *J* = 1.7 Hz, 3H), 1.88-1.79 (m, 1H), 1.84 (s, 3H), 1.79-1.70 (m, 1H), 1.72 (s, 3H), 1.49-1.39 (m, 1H), 1.34 (d, *J* = 7.0 Hz, 3H), 1.10-0.99 (m, 21H), 0.90 (d, *J* = 6.9 Hz, 3H).

^{13}C-NMR (125.8 MHz, $CDCl_3$): δ = 170.2, 165.1, 159.4, 151.0, 146.4, 143.8, 133.0, 124.3, 115.1, 113.9, 106.1, 89.3, 87.7, 87.3, 83.3, 82.2, 79.2, 76.4, 72.5, 70.3, 56.6, 55.2, 38.7, 38.6, 35.9, 35.6, 32.9, 32.0, 30.6, 19.5, 18.2, 18.0, 17.3, 16.7, 12.4, 9.8, 4.7.

IR (neat, ν/cm^{-1}): 2924m, 2865m, 1737m, 1719m, 1655w, 1607w, 1510m, 1462m, 1379w, 1248s, 1173w, 1085s, 1058s, 1030m, 883m, 832m, 804m, 683m.

HRMS (ESI): *m/z* calcd for $C_{45}H_{65}INaO_7Si$ [M + Na]$^+$: 895.3436, found: 895.3424.

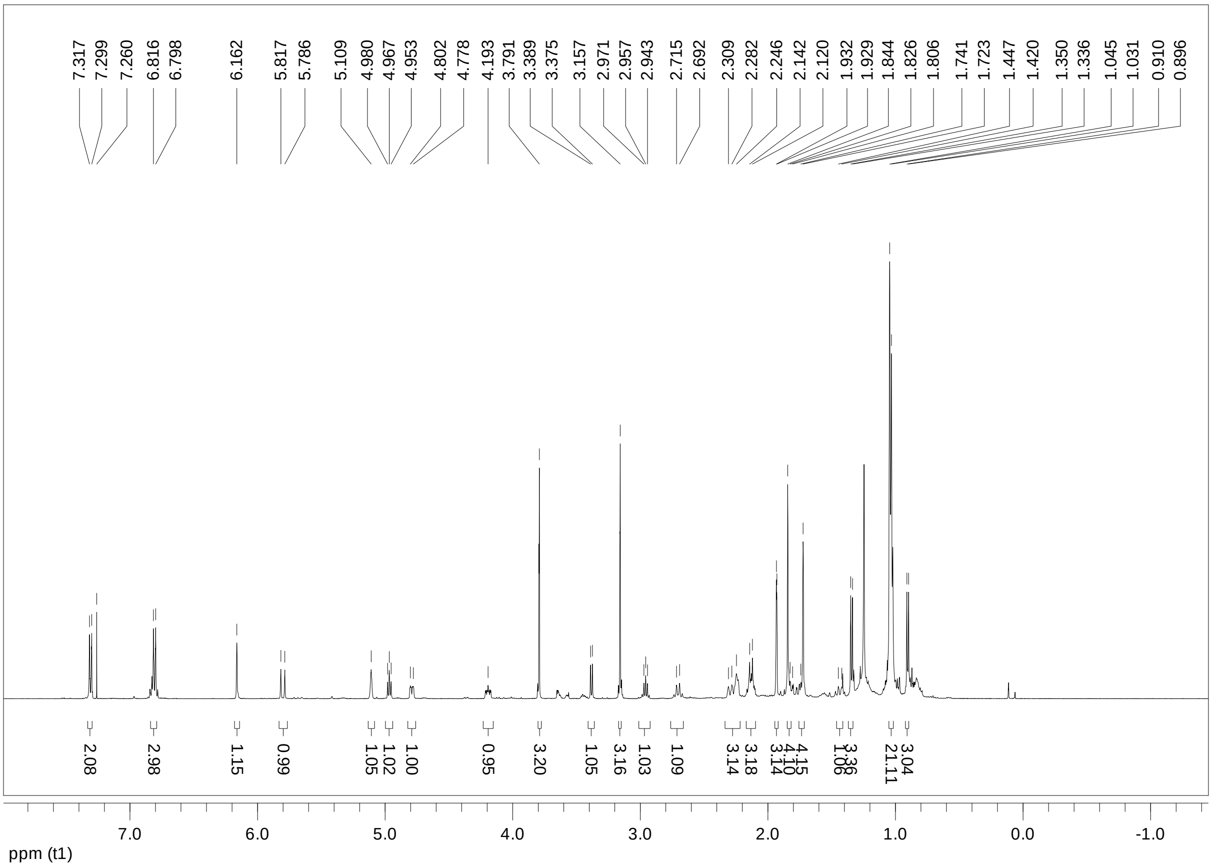

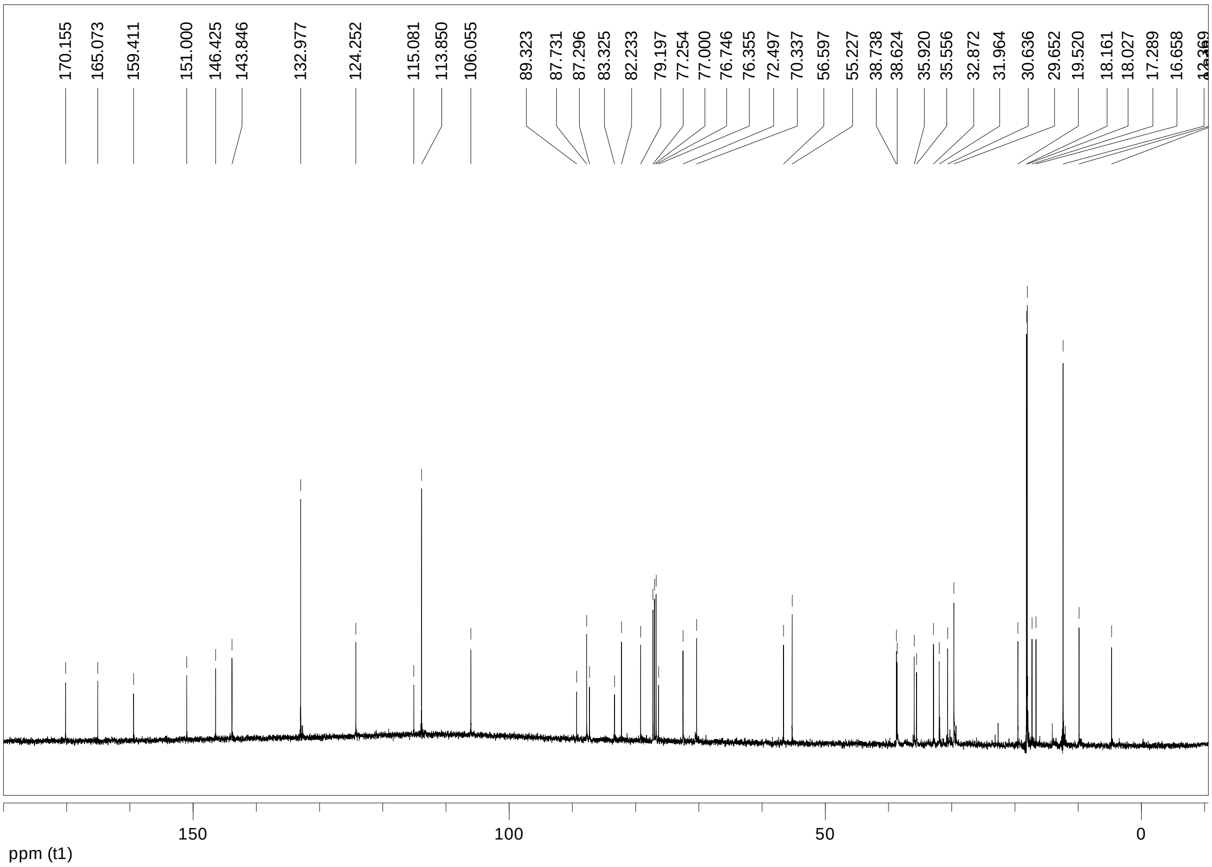

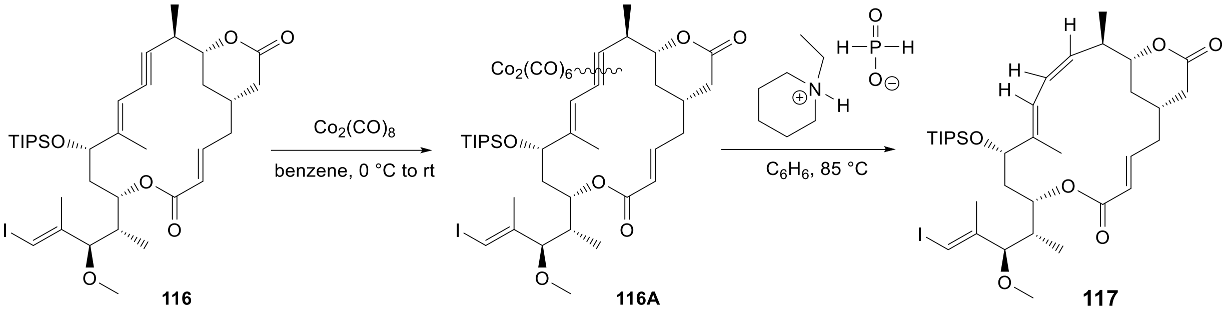

(1*R*,3*E*,7*S*,9*S*,10*E*,12*Z*,14*R*,15*R*)-7-((2*S*,3*R*,*E*)-5-Iodo-3-methoxy-4-methylpent-4-en-2-yl)-10,14-dimethyl-9-((triisopropylsilyl)oxy)-6,16-dioxabicyclo-[13.3.1]nonadeca-3,10,12-triene-5,17-dione (117): To alkyne **116** (323 mg, 444 µmol, 1.00 eq) was added $Co_2(CO)_8$ (304 mg, 889 µmol, 2.00 eq) in a glovebox and the mixture was dissolved in CH_2Cl_2 (16 mL) at rt. After stirring at rt for 4 h, the reaction did not proceed any further. The solvent evaporated and crude **116A** was dried under reduced pressure (affording a dark brown foam), then N-ethylpiperidine hypophosphite was added in a glovebox, benzene (19 mL) was added and the mixture was heated to reflux for 1.5 h. Afterwards it was passed through a plug of silica eluting with hexane/EtOAc 1:1 (100 mL) and the plug was rinsed with EtOAc (2×100 mL). Evaporation of the solvent and drying of the residue under reduced pressure gave a mixture of the parent alkyne **116** and the desired olefin **117** as a pale brown foam (293 mg). ^{1}H-NMR measurement failed, probably due to paramagnetic cobalt being present. The conversion was estimated to be 50% from ESI-MS and from a former experiment, where NMR-analysis was possible.

2nd Cycle: To a solution of the above mixture (estimated to containe 146 mg alkyne **116**, 0.201 mmol, 1.00 eq) in CH_2Cl_2 (8 mL) was added $Co_2(CO)_8$ (137 mg, 2 equiv); the solution was stirred for 3.5 h and then stored in a freezer overnight. The solvent was evaporated and the residue was dried under reduced pressure. To the resulting dark brown foam was added N-ethylpiperidine hypophosphite (360 mg, 10 eq), benzene (9 mL) was added and the mixture was refluxed for 40 min. It was then filtered through a plug of silica (hexane/EtOAc 1:1) to afford a mixture of **116** and **117** (273 mg). The conversion was again estimated to be around 50%.

3rd Cycle: To the mixture obtained above (estimated 68 mg alkyne **153**, 1.00 eq) was added $Co_2(CO)_8$ (64 mg, 2.00 eq) in a glovebox and CH_2Cl_2 (3.5 mL) was added at rt. After stirring for 1 h, the solvent was evaporated and the residue was dried under reduced

pressure. To the dark brown foam was added N-ethylpiperidine hypophosphite (168 mg, 10 eq) in a glovebox followed by benzene (4 mL) and the mixture was refluxed for 40 min. After filtration through a plug of silica (hexane/EtOAc 1:1) the solvent was evaporated and the residue was purified by column chromatography (hexane/EtOAc 3:1) to afford the desired olefin **117** (239 mg, 74%; **117**/**116** 20/1) as a beige foam.

TLC (hexane/EtOAc, 2:1): R_f = 0.38

[α]$_D^{24}$ = –33.3° [*c* = 0.640, $CHCl_3$]

^{1}H-NMR (400.1 MHz, $CDCl_3$): δ = 6.65 (ddd, *J* = 4.7, 10.9, 15.6 Hz, 1H), 6.46 (d, *J* = 12.2 Hz, 1H), 6.25 (dd, *J* = 11.5, 11.5 Hz, 1H), 6.18 (s, 1H), 5.82 (d, *J* = 15.8 Hz, 1H), 5.00 (dd, *J* = 10.6, 10.6 Hz, 1H), 4.42 (m_c, 2H), 3.80 (dt, *J* = 3.6, 10.0 Hz, 1H), 3.30 (d, *J* = 9.0 Hz, 1H), 3.16 (s, 3H), 2.79-2.63 (m, 1H), 2.73 (dd, *J* = 7.5, 17.1 Hz, 1H), 2.55 (m_c, 1H), 2.33 (m_c, 1H), 2.28 (dd, *J* = 6.5, 17.2 Hz, 1H), 2.21-2.07 (m, 2H), 1.95-1.78 (m, 3H), 1.88 (s, 3H), 1.62 (s, 3H), 1.15-1.04 (m, 25H), 0.98 (d, *J* = 6.9 Hz, 3H).

^{13}C-NMR (100.6 MHz, $CDCl_3$): δ = 171.6, 166.0, 147.2, 144.8, 139.7, 130.7, 126.1, 125.4, 119.2, 88.3, 81.6, 79.7, 73.5, 71.8, 56.4, 39.1, 38.8, 38.7, 35.9, 32.8, 32.3, 28.5, 18.8, 18.2, 18.1, 17.2, 15.0, 12.4, 10.0.

IR (neat, ν/cm^{-1}): 2926s, 2866s, 1714s, 1654w, 1461m, 1379m, 1326w, 1255m, 1220m, 1160w, 1118w, 1083m, 1010w, 884w, 753w, 679w.

HRMS (ESI): *m/z* calcd for $C_{35}H_{57}IKO_6Si$ [M + K]$^+$: 767.2601; found: 767.2605.

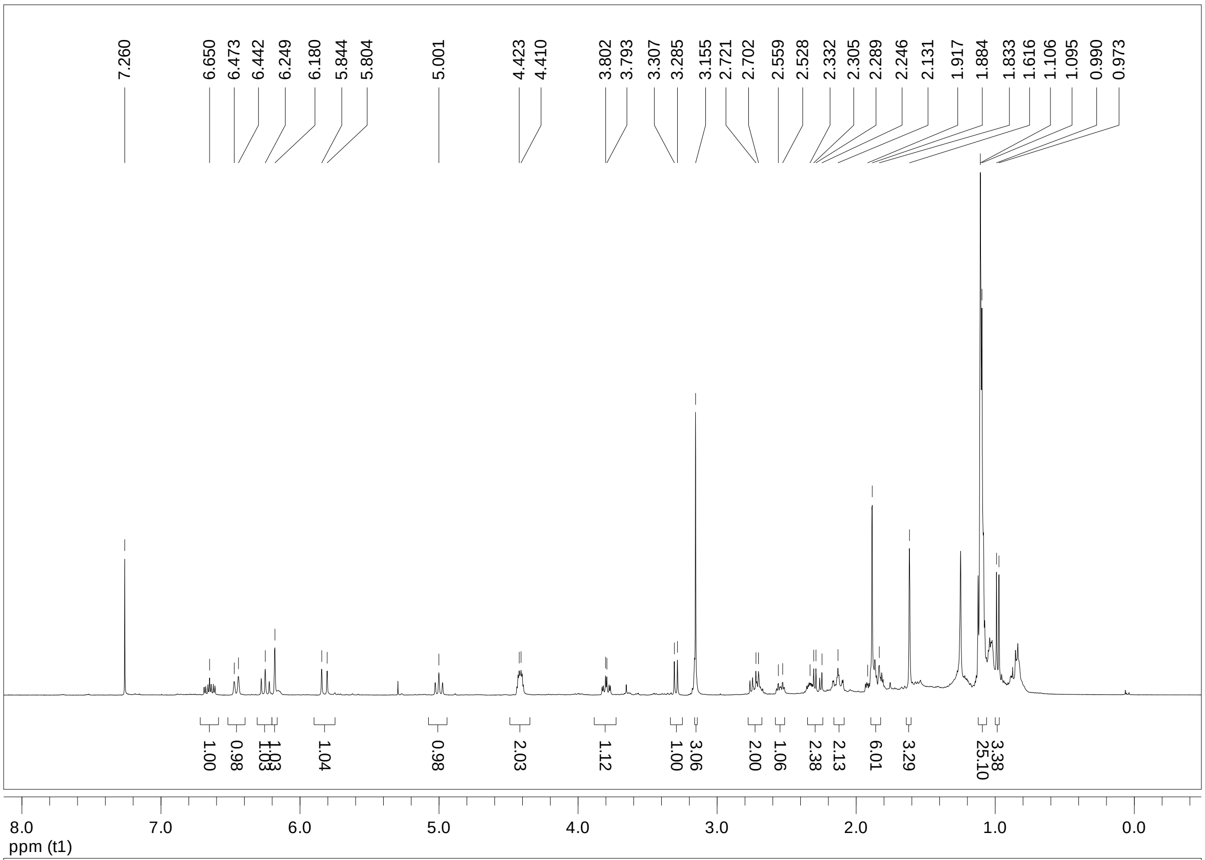
7.260
6.650
6.473
6.442
6.249
6.180
5.844
5.804
5.001
4.423
4.410
3.802
3.793
3.307
3.285
3.155
2.721
2.702
2.559
2.528
2.332
2.305
2.289
2.246
2.131
1.917
1.884
1.833
1.616
1.106
1.095
0.990
0.973
1.00
0.98
1.03
1.03
1.04
0.98
2.03
1.12
1.00
3.06
2.00
1.06
2.38
2.13
6.01
3.29
25.10
3.38
8.0
7.0
6.0
5.0
4.0
3.0
2.0
1.0
0.0
ppm (t1)

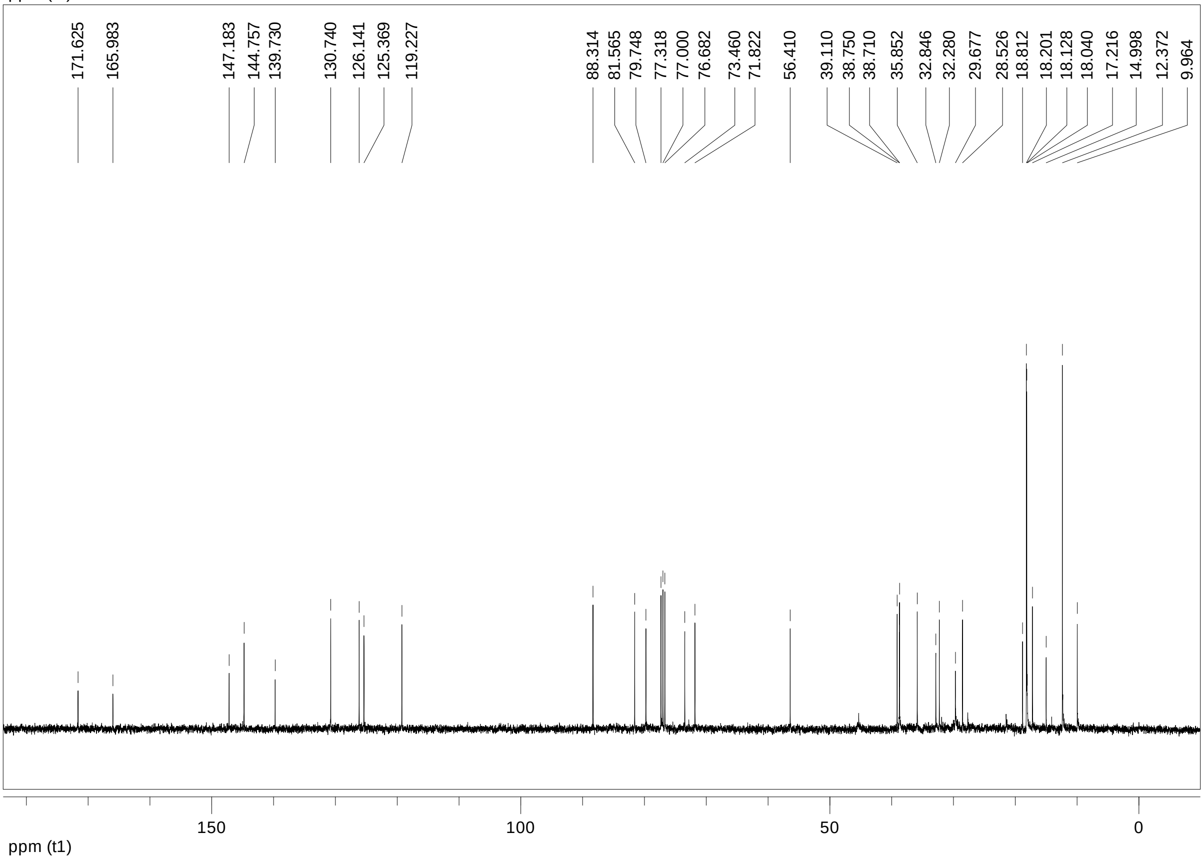
171.625
165.983
147.183
144.757
139.730
130.740
126.141
125.369
119.227
88.314
81.565
79.748
77.318
77.000
76.682
73.460
71.822
56.410
39.110
38.750
38.710
35.852
32.846
32.280
29.677
28.526
18.812
18.201
18.128
18.040
17.216
14.998
12.372
9.964
150
100
50
0
ppm (t1)

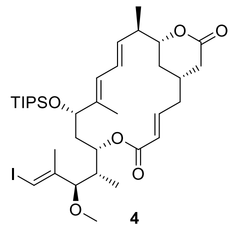

(1*R*,3*E*,7*S*,9*S*,10*E*,12*E*,14*R*,15*R*)-7-((2*S*,3*R*,*E*)-5-Iodo-3-methoxy-4-methylpent-4-en-2-yl)-10,14-dimethyl-9-((triisopropylsilyl)oxy)-6,16-dioxabicyclo-[13.3.1]nonadeca-3,10,12-triene-5,17-dione (4): A stock solution containing thiophenol (10.75 mg, 97.6 μmol) and AIBN (16.0 mg, 97.6 μmol) in benzene (1.8 mL) was prepared. Then an aliquot (0.1 mL, 0.2 equiv AIBN, 0.2 equiv PhSH) was added to a solution of the *cis* diene **117** (18.8 mg, 25.8 μmol, 1.00 eq) in benzene (1.2 mL); the solution was degassed (2×freeze/pump) and then heated to reflux for 2 d. The solvent was evaporated and the residue was purified by column chromatography (hexane/EtOAc 3:1) to afford the desired (*E,E*)-diene **4** (16.9 mg, 97% wt/wt along with EtOAc, 16.5 mg, 88%, *E/Z* 20/1).

TLC (hexane/EtOAc, 2:1): $\mathbf{R}_f$ = 0.38

$[\alpha]_D^{24}$ = +10.6° (*c* = 0.305, $CHCl_3$)

^{1}H-NMR (400.1 MHz, $CDCl_3$): δ = 6.75 (ddd, *J* = 4.8 Hz, *J* = 11.0 Hz, *J* = 15.7 Hz, 1H), 6.22 (dd, *J* = 11.0 Hz, *J* = 15.2 Hz, 1H), 6.15 (d, *J* = 0.8 Hz, 1H), 5.74 (bd, *J* = 10.3 Hz, 1H), 5.60 (dd, *J* = 0.7 Hz, *J* = 15.6 Hz, 1H), 5.14 (dd, *J* = 9.6 Hz, *J* = 15.2 Hz, 1H), 4.45 (dd, *J* = 3.0 Hz, *J* = 10.8 Hz, 1H), 4.01 (dd, *J* = 3.3 Hz, *J* = 10.6 Hz, 1H), 3.68 (ddd, *J* = 2.7 Hz, *J* = 9.8 Hz, *J* = 12.1 Hz, 1H), 3.35 (d, *J* = 8.7 Hz, 1H), 3.16 (s, 3H), 2.76 (ddd, *J* = 2.1 Hz, *J* = 5.2 Hz, *J* = 18.1 Hz, 1H), 2.59-2.46 (m, 1H), 2.32-2.18 (m, 2H), 2.19-2.04 (m, 2H), 2.00 (qd, *J* = 2.4 Hz, *J* = 14.3 Hz, 1H), 1.92-1.67 (m, 2H), 1.90 (d, *J* = 1.0 Hz, 3H), 1.75 (d, *J* = 0.9 Hz, 3H), 1.60 (dd, *J* = 3.2 Hz, *J* = 15.3 Hz, 1H), 1.21 (d, *J* = 6.4 Hz, 3H), 1.14-0.98 (m, 21H), 0.96 (d, *J* = 6.9 Hz, 3H), 0.68 (td, *J* = 12.0 Hz, *J* = 14.1 Hz, 1H).

^{13}C-NMR (100.6 MHz, $CDCl_3$): δ = 170.4, 165.6, 147.2, 146.0, 139.9, 133.5, 130.0, 124.7, 124.6, 88.5, 83.4, 79.3, 78.6, 73.6, 56.5, 45.3, 38.7, 38.2, 36.9, 34.6, 34.3, 29.8, 18.9, 18.2 (2C), 18.1, 16.6, 12.4 (6C), 11.2, 9.8.

IR (neat, ν/cm^{-1}): 2938 (s), 2866 (s), 1732 (s), 1650, 1613, 1460, 1383, 1327, 1254, 1222, 1202, 1167, 1124, 1078 (s), 1054 (s), 1011, 980, 885, 838, 800, 785, 763, 755, 746, 682, 656, 603, 534, 507, 475, 468, 453, 445, 437, 428, 411.

HRMS (ESI): *m/z* calcd. for $C_{35}H_{57}IKO_6Si$ $[M+K]^+$: 767.2601; found: 767.2603.

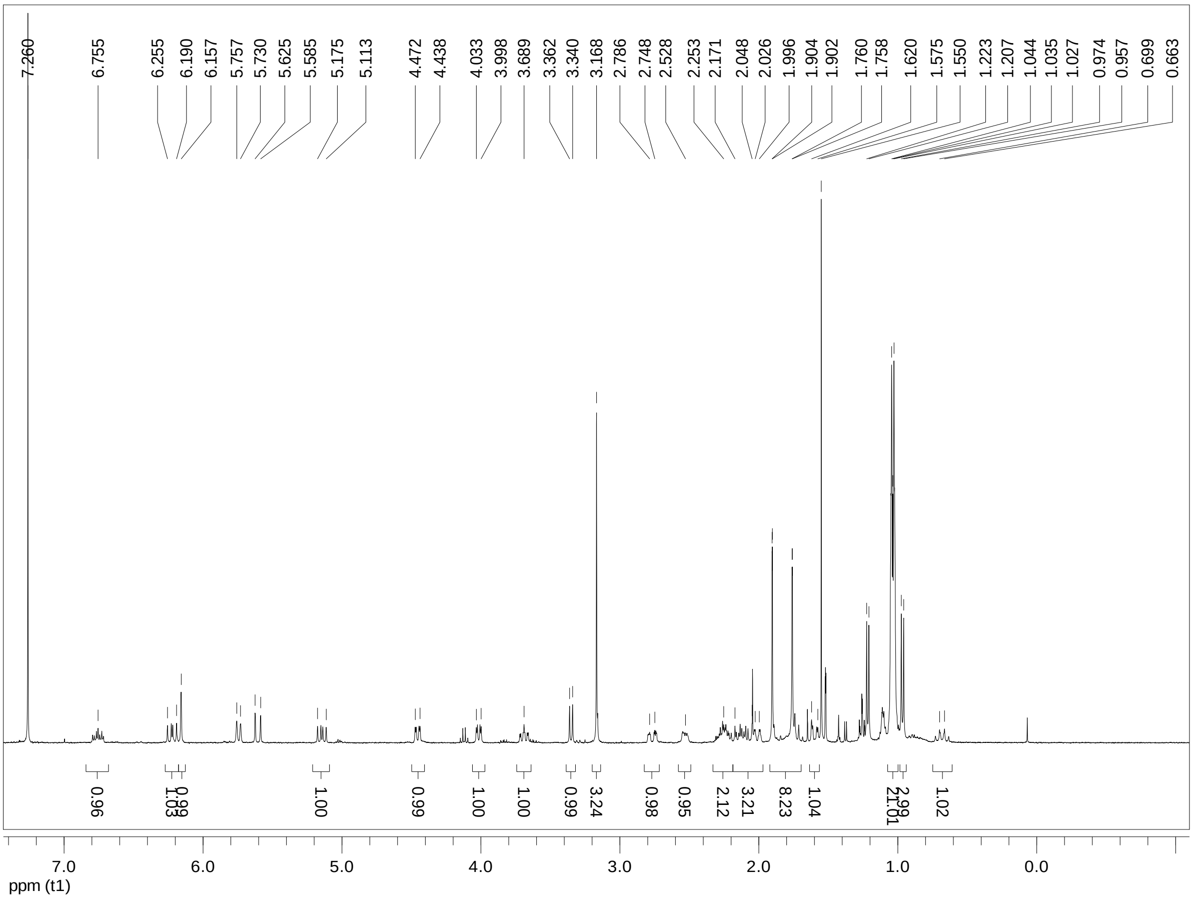
7.260 6.755 6.255 6.190 6.157 5.757 5.730 5.625 5.585 5.175 5.113 4.472 4.438 4.033 3.998 3.689 3.362 3.340 3.168 2.786 2.748 2.528 2.253 2.171 2.048 2.026 1.996 1.904 1.902 1.760 1.758 1.620 1.575 1.550 1.223 1.207 1.044 1.035 1.027 0.974 0.957 0.699 0.663
0.96 1.03 0.99 1.00 0.99 1.00 1.00 0.99 3.24 0.98 0.95 2.12 3.21 8.23 1.04 21.01 2.99 1.02
7.0 6.0 5.0 4.0 3.0 2.0 1.0 0.0
ppm (t1)

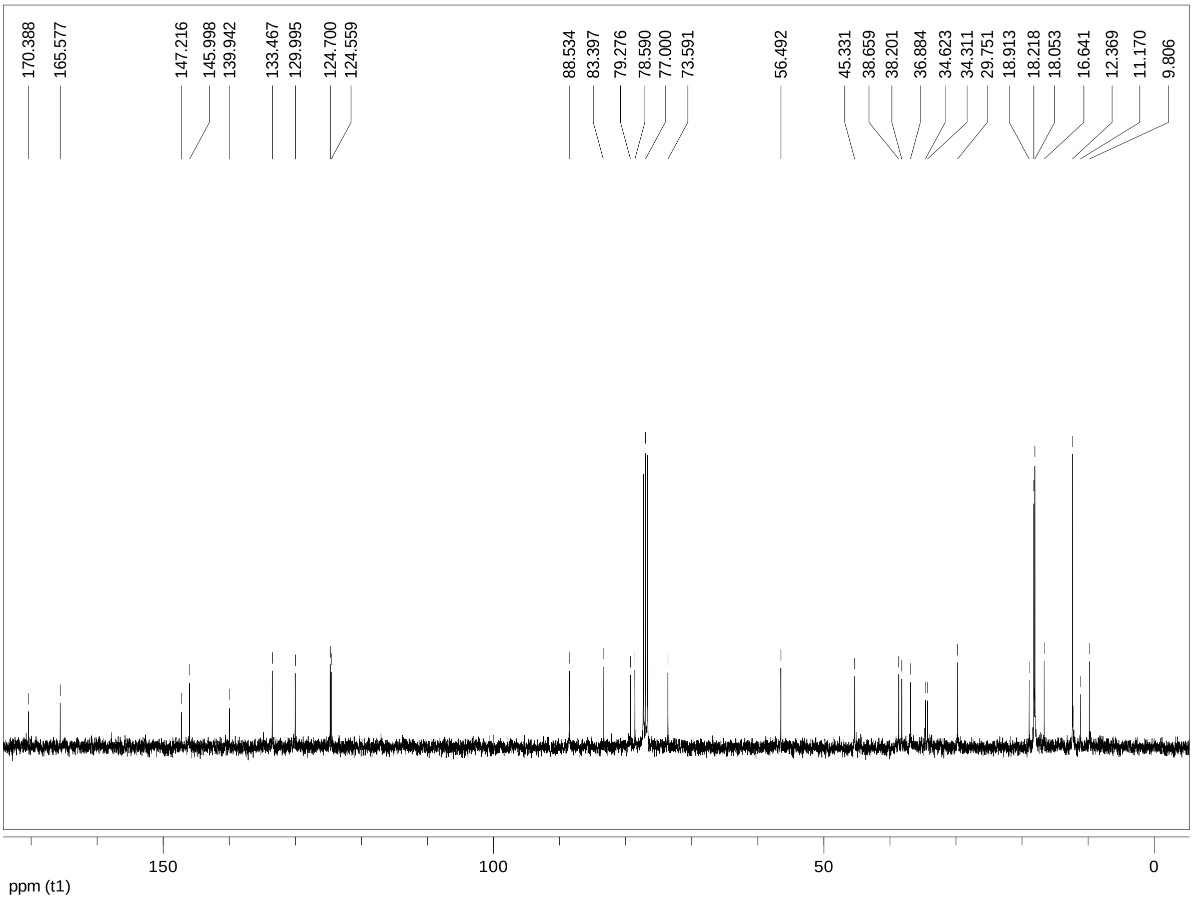
170.388 165.577 147.216 145.998 139.942 133.467 129.995 124.700 124.559 88.534 83.397 79.276 78.590 77.000 73.591 56.492 45.331 38.659 38.201 36.884 34.623 34.311 29.751 18.913 18.218 18.053 16.641 12.369 11.170 9.806
150 100 50 0
ppm (t1)

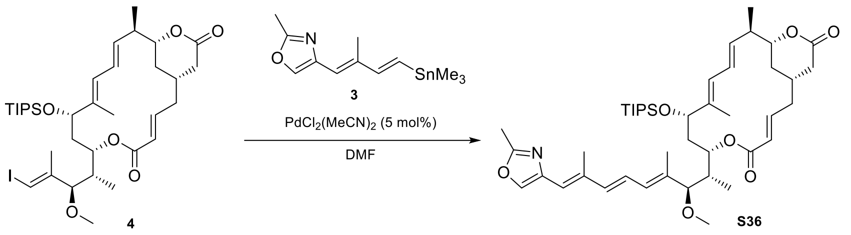

(1*R*,3*E*,7*S*,9*S*,10*E*,12*E*,14*R*,15*R*)-7-((2*S*,3*R*,4*E*,6*E*,8*E*)-3-Methoxy-4,8-dimethyl-9-(2-methyloxazol-4-yl)nona-4,6,8-trien-2-yl)-10,14-dimethyl-9-((triisopropylsilyl)-oxy)-6,16-dioxabicyclo[13.3.1]nonadeca-3,10,12-triene-5,17-dione (S36): To vinyl iodide **4** (16.5 mg, 22.6 µmol, 1.00 eq) was added a solution vinyl stannane **118** (14.1 mg, 45.2 µmol, 2.00 eq) in DMF (1.6 mL) at rt, followed by a solution of $PdCl_2(MeCN)_2$ (290 µg, 1.13 µmol, 5 mol%) in DMF (620 µL). The pale yellow solution was stirred in the dark overnight, whereafter the colour had changed to dark orange. Reaction monitoring by ESI-MS indicated full conversion. Ether (15 mL) and $NaHCO_3$ (sat. aq., 15 mL) were added and the layers were separated. The aqueous phase was extracted with ether (3×10 mL) and the combined organic extracts were washed with brine, dried over $MgSO_4$ and concentrated under reduced pressure. The residue was purified by column chromatography (hexane/EtOAc 2:1→1:1) to afford the desired product **S36** (11.6 mg, 68%) as a yellow oil.

TLC (hexane/EtOAc, 1:1): R_f = 0.54

$[\alpha]_D^{24}$ = +130.9° (*c* = 0.580, $CHCl_3$)

^{1}H-NMR (400.1 MHz, $CDCl_3$): δ = 7.55 (s, 1H), 6.74 (ddd, *J* = 4.7 Hz, *J* = 10.9 Hz, *J* = 15.6 Hz, 1H), 6.61 (dd, *J* = 10.8 Hz, *J* = 15.2 Hz, 1H), 6.33 (d, *J* = 15.3 Hz, 1H), 6.24 (bs, 1H), 6.20 (dd, *J* = 10.9 Hz, *J* = 15.2 Hz, 1H), 6.05 (d, *J* = 10.8 Hz, 1H), 5.69 (d, *J* = 11.2 Hz, 1H), 5.60 (d, *J* = 16.0 Hz, 1H), 5.09 (dd, *J* = 9.6 Hz, *J* = 15.2 Hz, 1H), 4.54 (dd, *J* = 2.7 Hz, *J* = 10.9 Hz, 1H), 3.99 (dd, *J* = 3.3 Hz, *J* = 10.6 Hz, 1H), 3.65 (ddd, *J* = 2.5 Hz, *J* = 9.3 Hz, *J* = 11.9 Hz, 1H), 3.20 (d, *J* = 9.4 Hz, 1H), 3.16 (s, 3H), 2.75 (ddd, *J* = 2.1 Hz, *J* = 5.2 Hz, *J* = 18.1 Hz, 1H), 2.56-2.49 (m), 2.46 (s, 3H), 2.32-2.19 (m, 2H), 2.19-1.95 (m, 3H), 2.13 (s, 3H), 1.90 (s, 3H), 1.86-1.58 (m, 3H), 1.75 (s, 3H), 1.19 (d, *J* = 6.4 Hz, 3H), 1.06-0.89 (m, 24H), 0.67 (m_c, 1H).

^{13}C-NMR (100.6 MHz, $CDCl_3$): δ = 170.4, 165.7, 160.9, 145.8, 140.1, 138.7, 137.5, 137.4, 136.9, 135.9, 133.3, 130.1, 129.3, 124.65, 124.56, 124.1, 120.6, 89.8, 83.4, 78.5, 73.8, 56.1, 45.3, 38.7, 38.2, 36.9, 34.6, 34.2, 29.8, 18.2 (2C), 18.1, 16.6, 14.4, 13.8, 12.4 (6C), 11.5, 11.2, 10.0; **IR** (neat, ν/cm^{-1}): 2961, 2938, 2893, 2866, 1715 (s), 1649, 1582, 1460, 1383, 1319,

1251, 1220, 1168, 1109, 1077 (s), 1052 (s), 978, 969, 919, 884, 795, 754 (s), 682, 663, 638, 426.

HRMS (ESI): *m/z* calcd. for $C_{44}H_{68}NO_7Si$ $[M+H]^+$: 750.4760; found: 750.4768.

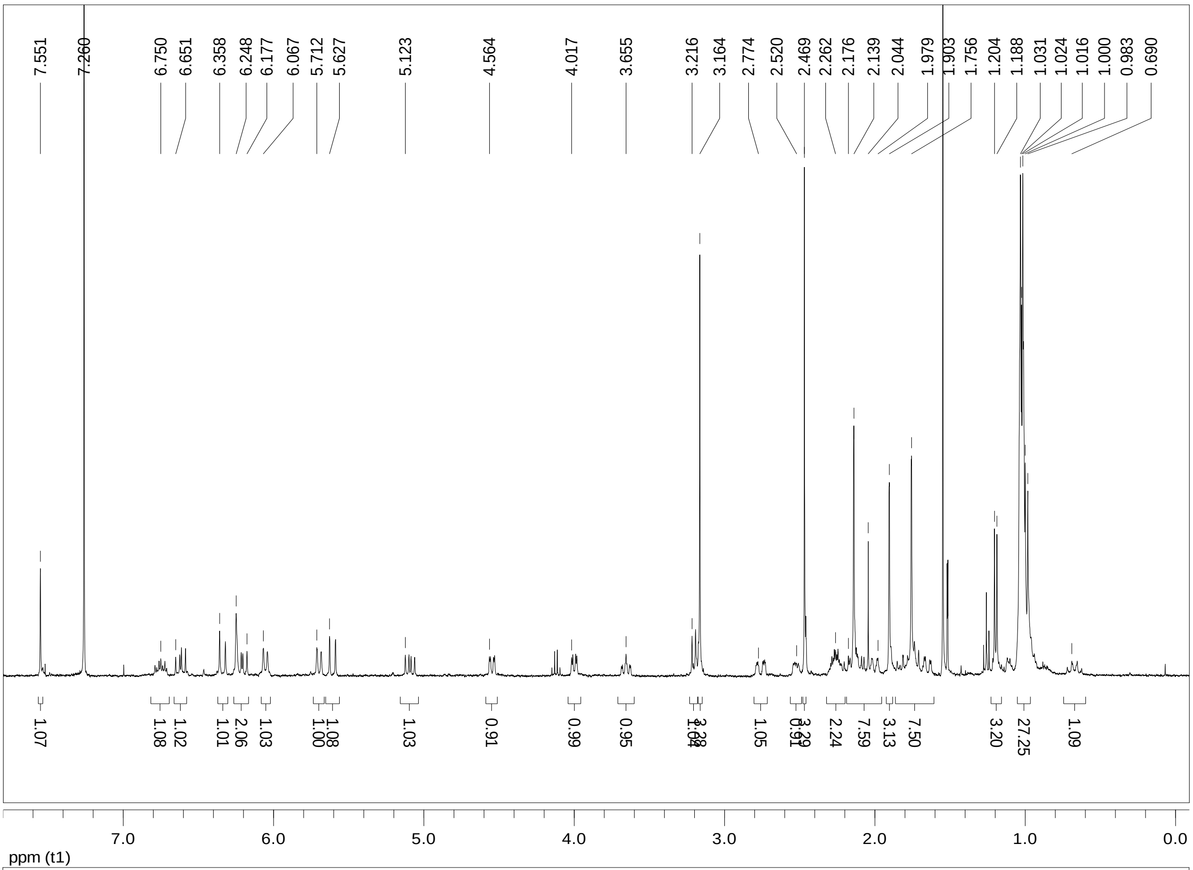
7.551
7.260
6.750
6.651
6.358
6.248
6.177
6.067
5.712
5.627
5.123
4.564
4.017
3.655
3.216
3.164
2.774
2.520
2.469
2.262
2.176
2.139
2.044
1.979
1.903
1.756
1.204
1.188
1.031
1.024
1.016
1.000
0.983
0.690
ppm (t1)

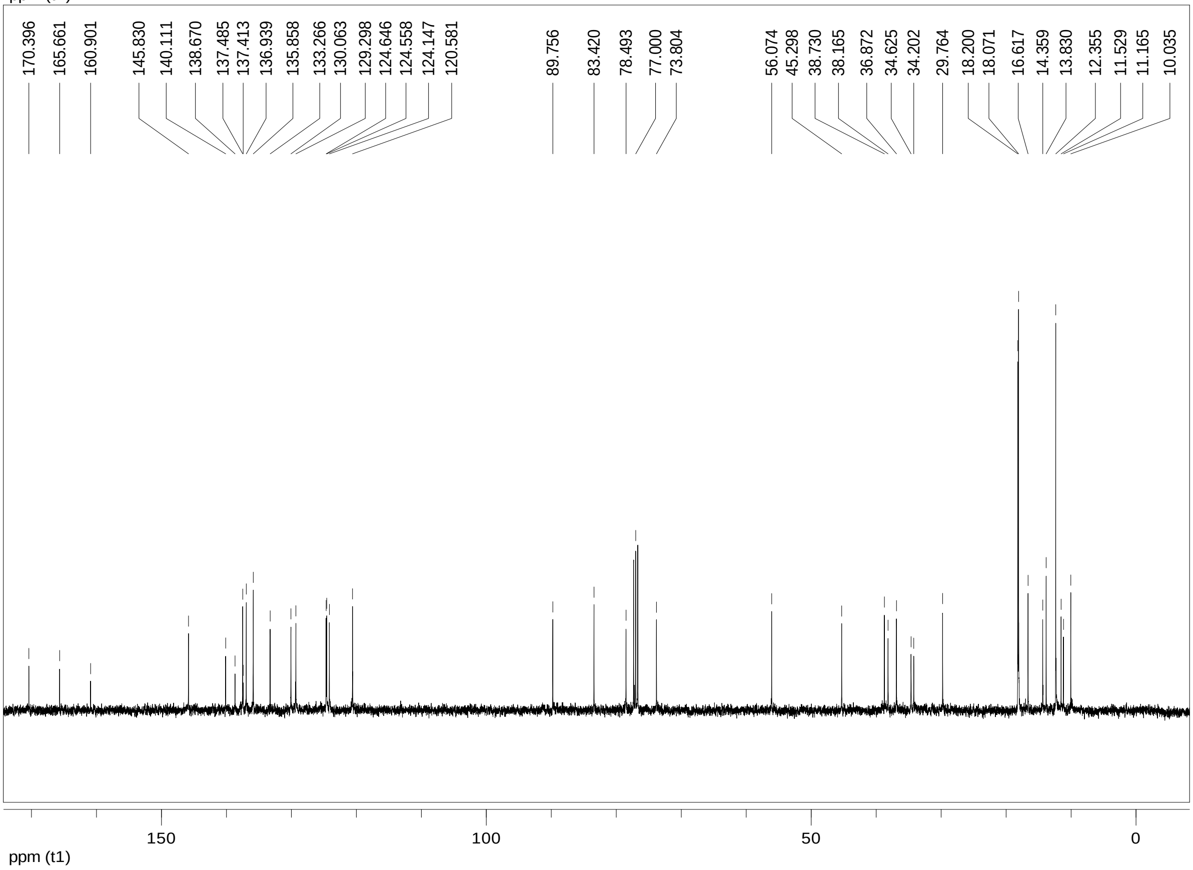
170.396
165.661
160.901
145.830
140.111
138.670
137.485
137.413
136.939
135.858
133.266
130.063
129.298
124.646
124.558
124.147
120.581
89.756
83.420
78.493
77.000
73.804
56.074
45.298
38.730
38.165
36.872
34.625
34.202
29.764
18.200
18.071
16.617
14.359
13.830
12.355
11.529
11.165
10.035
ppm (t1)

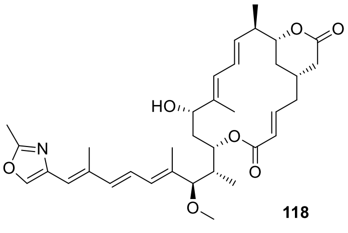
118

(1*R*,3*E*,7*S*,9*S*,10*E*,12*E*,14*R*,15*R*)-9-Hydroxy-7-((2*S*,3*R*,4*E*,6*E*,8*E*)-3-methoxy-4,8-dimethyl-9-(2-methyloxazol-4-yl)nona-4,6,8-trien-2-yl)-10,14-dimethyl-6,16-dioxabicyclo[13.3.1]nonadeca-3,10,12-triene-5,17-dione (rhizoxin D) (3): To a solution of TIPS-protected alcohol **S36** (91.0 mg, 121 µmol, 1.00 eq) in THF (4.6 mL) and pyridine (1.34 mL) was added a solution of HF•Py (2.68 mL 70% as HF, 2.06 g, 103 mmol, 850 eq) carefully at 0 °C. The cooling bath was removed after ca. 10 min and the mixture was stirred at rt overnight. The reaction mixture was then poured into water/ether (90 mL/30 mL) and shaken. The layers were separated and the aqueous phase was extracted with ether (3×30 mL). The combined organic extracts were washed with $NaHCO_3$ (sat. aq.) and $CuSO_4$ (sat. aq.) and the combined aqueous layers were re-extracted with ether (1×10 mL). The combined organic extracts were then dried over $MgSO_4$ and concentrated under reduced pressure. The crude product was purified by column chromatography (hexane/EtOAc 1:1) to afford **3** (44.4 mg, 87% wt along with EtOAc, 38.8 mg, 54%) as a pale yellow oil.

TLC (hexane/EtOAc, 1:1): R_f = 0.17

$[\alpha]_D^{24}$ = +208.2° (*c* = 0.172, $CHCl_3$)

^{1}H-NMR (400.1 MHz, $CDCl_3$): δ = 7.54 (s, 1H), 6.74 (ddd, *J* = 4.7 Hz, *J* = 11.0 Hz, *J* = 15.7 Hz, 1H), 6.62 (dd, *J* = 10.9 Hz, *J* = 15.2 Hz, 1H), 6.39 (d, *J* = 15.2 Hz, 1H), 6.26 (s, 1H), 6.22 (dd, *J* = 11.1 Hz, *J* = 15.3 Hz, 1H), 6.11 (d, *J* = 10.9 Hz, 1H), 5.79 (d, *J* = 11.0 Hz, 1H), 5.60 (dd, *J* = 0.7 Hz, *J* = 15.6 Hz, 1H), 5.14 (dd, *J* = 9.7 Hz, *J* = 15.2 Hz, 1H), 4.57 (dd, *J* = 3.0 Hz, *J* = 10.7 Hz, 1H), 3.90 (dd, *J* = 2.8 Hz, *J* = 10.9 Hz, 1H), 3.65 (ddd, *J* = 2.7 Hz, *J* = 8.1 Hz, *J* = 9.7 Hz, 1H), 3.25 (d, *J* = 9.3 Hz, 1H), 3.16 (s, 3H), 2.75 (ddd, *J* = 2.1 Hz, *J* = 5.2 Hz, *J* = 18.1 Hz, 1H), 2.56-2.48 (m, 1H), 2.46 (s, 3H), 2.30-2.23 (m, 2H), 2.16-2.11 (m, 1H), 2.14 (s, 3H), 2.07 (dd, *J* = 11.5 Hz, *J* = 18.0 Hz, 1H), 1.95 (ddd, *J* = 2.6 Hz, *J* = 5.1 Hz, *J* = 14.2 Hz, 1H), 1.89 (d, *J* = 0.7 Hz, 3H), 1.78 (d, *J* = 0.9 Hz, 3H), 1.75-1.66 (m, 3H), 1.18 (d, *J* = 6.4 Hz, 3H), 0.99 (d, *J* = 6.8 Hz, 3H), 0.67 (m_c, 1H).

^{13}C-NMR (100.6 MHz, $CDCl_3$): δ = 170.3, 165.6, 160.9, 145.9, 139.2, 137.4, 136.9, 136.8, 135.9, 134.4, 129.7, 129.3, 126.0, 124.5, 124.3, 120.6, 89.7, 83.2, 78.3, 74.2, 56.2, 45.3, 38.6, 38.2, 36.8, 34.5, 32.0, 29.8, 29.7, 16.5, 14.3, 13.8, 11.4, 10.9, 10.2.

IR (neat, ν/cm^{-1}): 3442, 2965, 2928, 2877, 2822, 1714 (s), 1649, 1580, 1450, 1382, 1322, 1252, 1222, 1168, 1109, 1078, 1040, 1019, 971, 889, 831, 754 (s), 662, 636, 593, 550, 511, 457, 436, 422.

HRMS (ESI): *m/z* calcd. for $C_{35}H_{47}NNaO_7$ $[M+K]^+$: 616.3245; found: 616.3245.

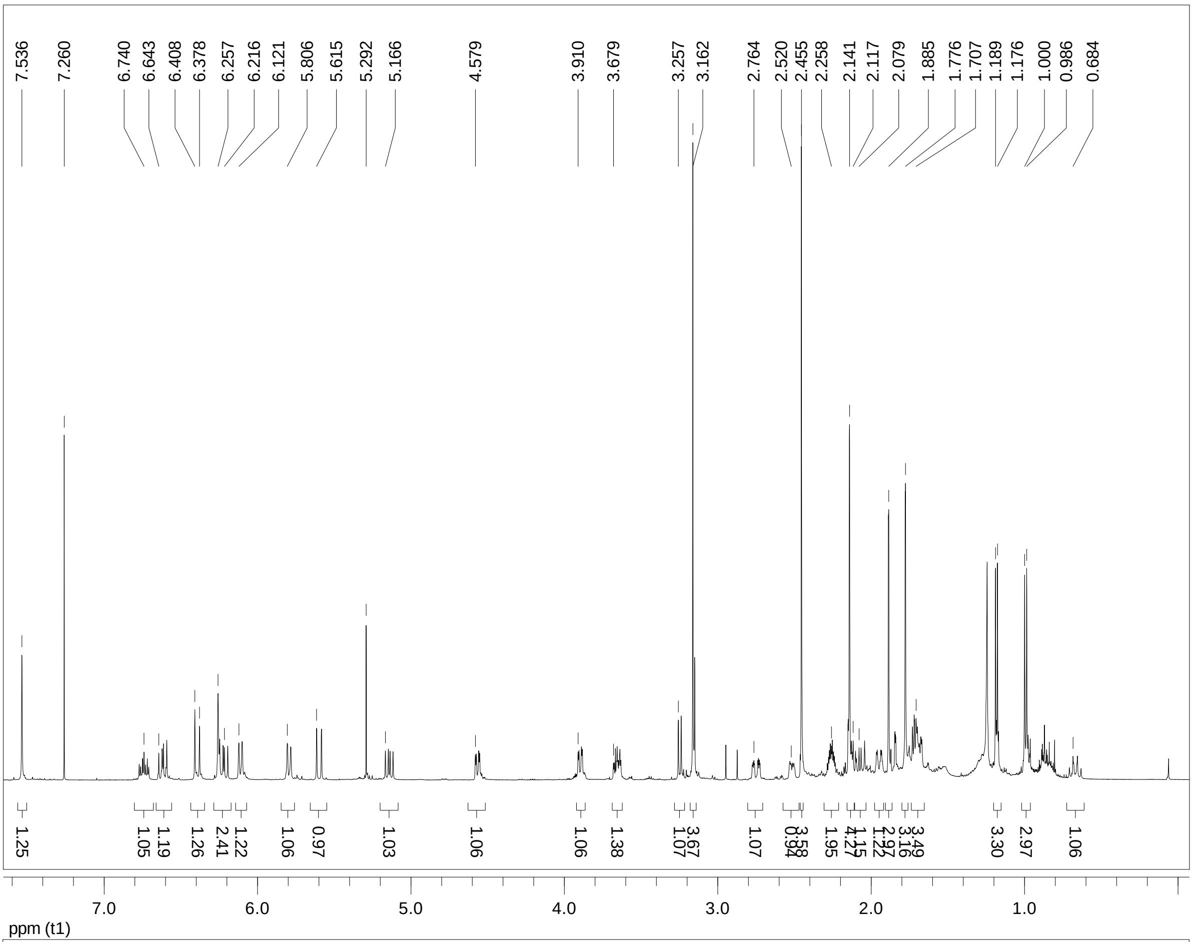
7.536
7.260
6.740
6.643
6.408
6.378
6.257
6.216
6.121
5.806
5.615
5.292
5.166
4.579
3.910
3.679
3.257
3.162
2.764
2.520
2.455
2.258
2.141
2.117
2.079
1.885
1.776
1.707
1.189
1.176
1.000
0.986
0.684
1.25
1.05
1.19
1.26
2.41
1.22
1.06
0.97
1.03
1.06
1.06
1.38
1.07
3.67
1.07
3.58
0.94
1.95
4.27
1.15
1.22
2.97
3.16
3.49
3.30
2.97
1.06
7.0
6.0
5.0
4.0
3.0
2.0
1.0
ppm (t1)

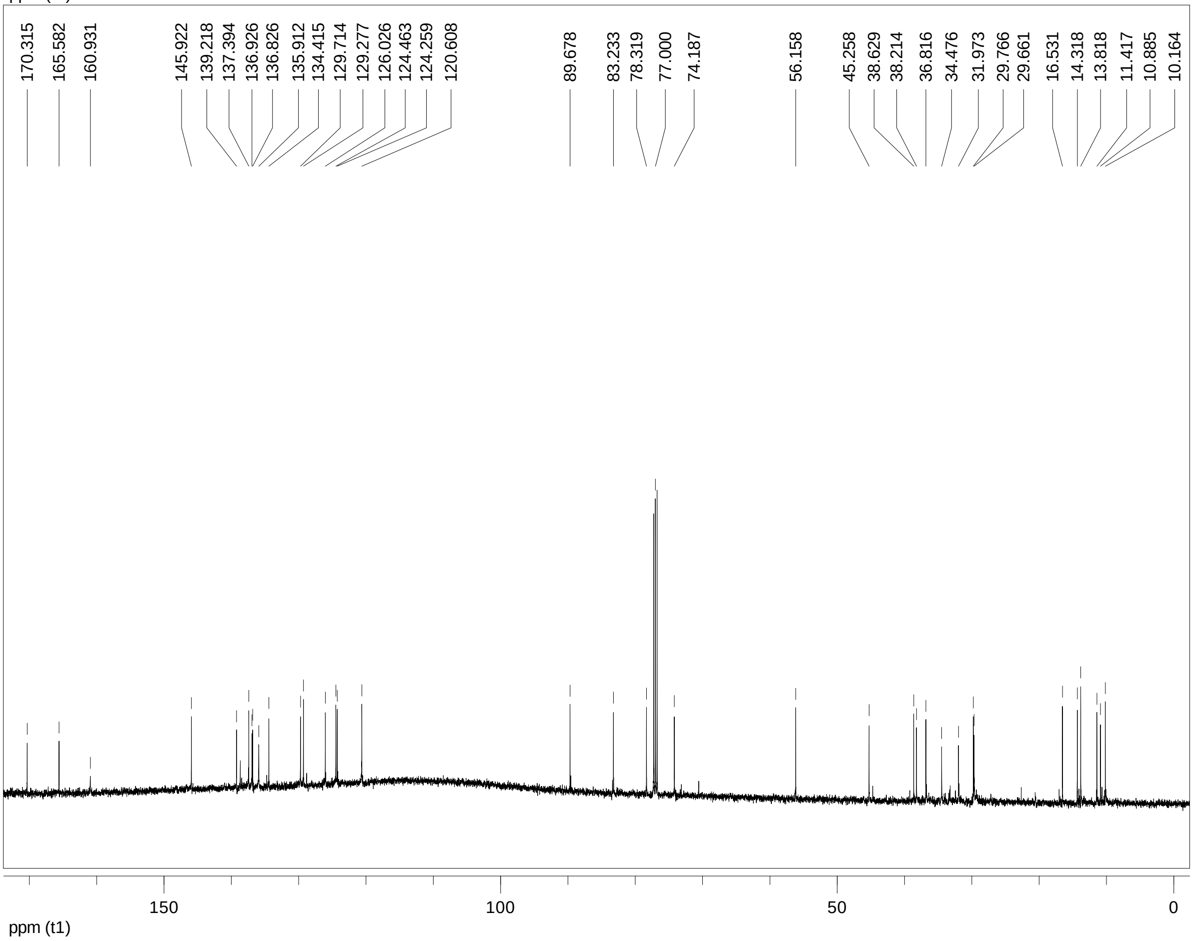
170.315
165.582
160.931
145.922
139.218
137.394
136.926
136.826
135.912
134.415
129.714
129.277
126.026
124.463
124.259
120.608
89.678
83.233
78.319
77.000
74.187
56.158
45.258
38.629
38.214
36.816
34.476
31.973
29.766
29.661
16.531
14.318
13.818
11.417
10.885
10.164
150
100
50
0
ppm (t1)

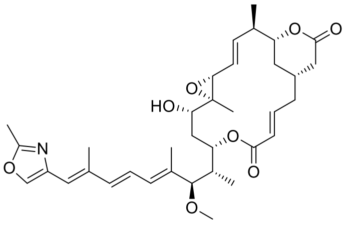

2

(1*R*,3*E*,7*S*,9*S*,10*E*,12*E*,14*R*,15*R*)-9-Hydroxy-7-((2*S*,3*R*,4*E*,6*E*,8*E*)-3-methoxy-4,8-dimethyl-9-(2-methyloxazol-4-yl)nona-4,6,8-trien-2-yl)-10,14-dimethyl-6,16-dioxabicyclo[13.3.1]nonadeca-3,10,12-triene-5,17-dione (rhizoxin F) (2): To a solution of **3** (38.8 mg, 65.3 µmol, 1.00 eq) in benzene (3 mL) was added *t*-BuOOH (14.3 µL 5.5 M in decane, 78.5 µmol, 1.20 eq) at rt. The mixture was then cooled to 0 °C and a solution of $VO(acac)_2$ (0.87 mg, 5 mol%) in benzene (1 mL, flask rinsed with 2×0.2 mL) was added, resulting in a red coloring of the solution. The cooling bath was removed after 5-10 min and stirring was continued for 3 h. The colour of the mixture changed from red to pale yellow during this time. Then the reaction was quenched with sat. $Na_2S_2O_3$, ether was added (10 mL) and the layers were separated. The aqueous phase was extracted with ether (3×10 mL) and the combined organic extracts were washed with brine, dried over $MgSO_4$, filtered and concentrated *in vacuo*. The crude product was purified by column chromatography (hexane/EtOAc 1:1) to afford WF-1360F (**2**) (25.8 mg, 65%) as a pale yellow foam. Preparative HPLC delivered analytically pure **2** as a white foam (11.5 mg, 29%).

$[\alpha]_D^{24}$ = +119.60° (*c* = 0.110, $CHCl_3$)

^{1}H-NMR (400.1 MHz, $CDCl_3$): δ = 7.53 (s, 1H), 6.82 (ddd, *J* = 5.1 Hz, *J* = 10.6 Hz, *J* = 15.7 Hz, 1H), 6.58 (dd, *J* = 10.8 Hz, *J* = 15.2 Hz, 1H), 6.37 (d, *J* = 15.2 Hz, 1H), 6.25 (bs, 1H), 6.09 (bd, *J* = 10.9 Hz, 1H), 5.68 (d, *J* = 15.6 Hz, 1H), 5.55 (dd, *J* = 9.9 Hz, *J* = 15.4 Hz, 1H), 5.34 (dd, *J* = 9.3 Hz, *J* = 15.4 Hz, 1H), 4.61 (dd, *J* = 3.0 Hz, *J* = 10.1 Hz, 1H), 3.74 (ddd, *J* = 2.6 Hz, *J* = 9.7 Hz, *J* = 12.0 Hz, 1H), 3.26 (d, *J* = 4.6 Hz, 1H), 3.24 (d, *J* = 4.7 Hz, 1H), 3.16 (s, 3H), 3.06 (dd, *J* = 2.1 Hz, *J* = 10.6 Hz, 1H), 2.78 (ddd, *J* = 1.6 Hz, *J* = 4.7 Hz, *J* = 6.8 Hz, 1H), 2.56 (dd, *J* = 4.6 Hz, *J* = 8.3 Hz, 1H), 2.46 (s, 3H), 2.43-2.28 (m, 2H), 2.14 (s, 3H), 2.13-2.03 (m, 2H), 1.96 (m_c, 1H), 1.88 (s, 3H), 1.86-1.70 (m, 3H), 1.42 (s, 3H), 1.18 (d, *J* = 6.5 Hz, 3H), 1.00 (d, *J* = 6.8 Hz, 3H), 0.72 (m_c, 1H).

^{13}C-NMR (100.6 MHz, $CDCl_3$): δ = 169.9, 165.3, 160.9, 146.1, 140.2, 138.7, 137.6, 136.7, 136.5, 136.0, 129.4, 126.5, 124.8, 124.0, 120.7, 89.6, 82.3, 76.9, 74.8, 65.7, 64.9, 56.2, 45.0, 38.2, 37.9, 36.8, 33.9, 31.6, 29.6, 16.6, 14.3, 13.8, 12.2, 11.4, 9.9.

IR (neat, ν/cm^{-1}): 3462br, 2974, 2928, 2873, 2857, 1716s, 1650, 1580, 1448, 1383, 1309, 1293, 1252, 1226, 1200, 1170, 1110s, 1077s, 1043s, 981s, 966s, 954, 932, 878, 863, 845, 829, 735s, 702, 635, 547, 484, 446, 427, 406.

HRMS (ESI): *m/z* calcd. for $C_{35}H_{47}NNaO_8$ $[M+Na]^+$: 632.3194; found: 632.3193.

HPLC (MeCN/H_2O, 40/60→90/10 in 15 min, 1 mL/min, 10 µL): t_r = 8.6 min.

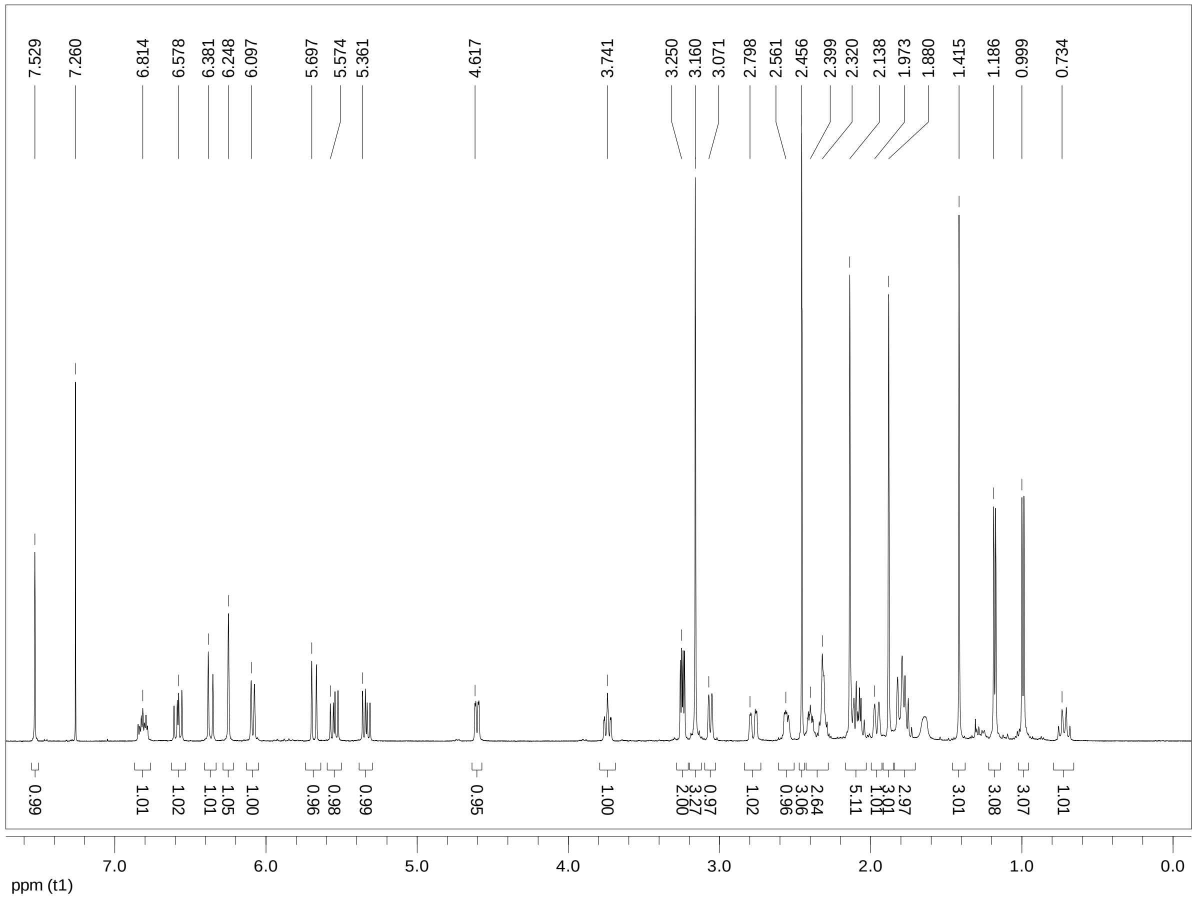
7.529
7.260
6.814
6.578
6.381
6.248
6.097
5.697
5.574
5.361
4.617
3.741
3.250
3.160
3.071
2.798
2.561
2.456
2.399
2.320
2.138
1.973
1.880
1.415
1.186
0.999
0.734
0.99
1.01
1.02
1.01
1.05
1.00
0.96
0.98
0.99
0.95
1.00
2.00
3.27
0.97
1.02
0.96
3.06
2.64
5.11
3.01
1.01
2.97
3.01
3.08
3.07
1.01
7.0
6.0
5.0
4.0
3.0
2.0
1.0
0.0
ppm (t1)

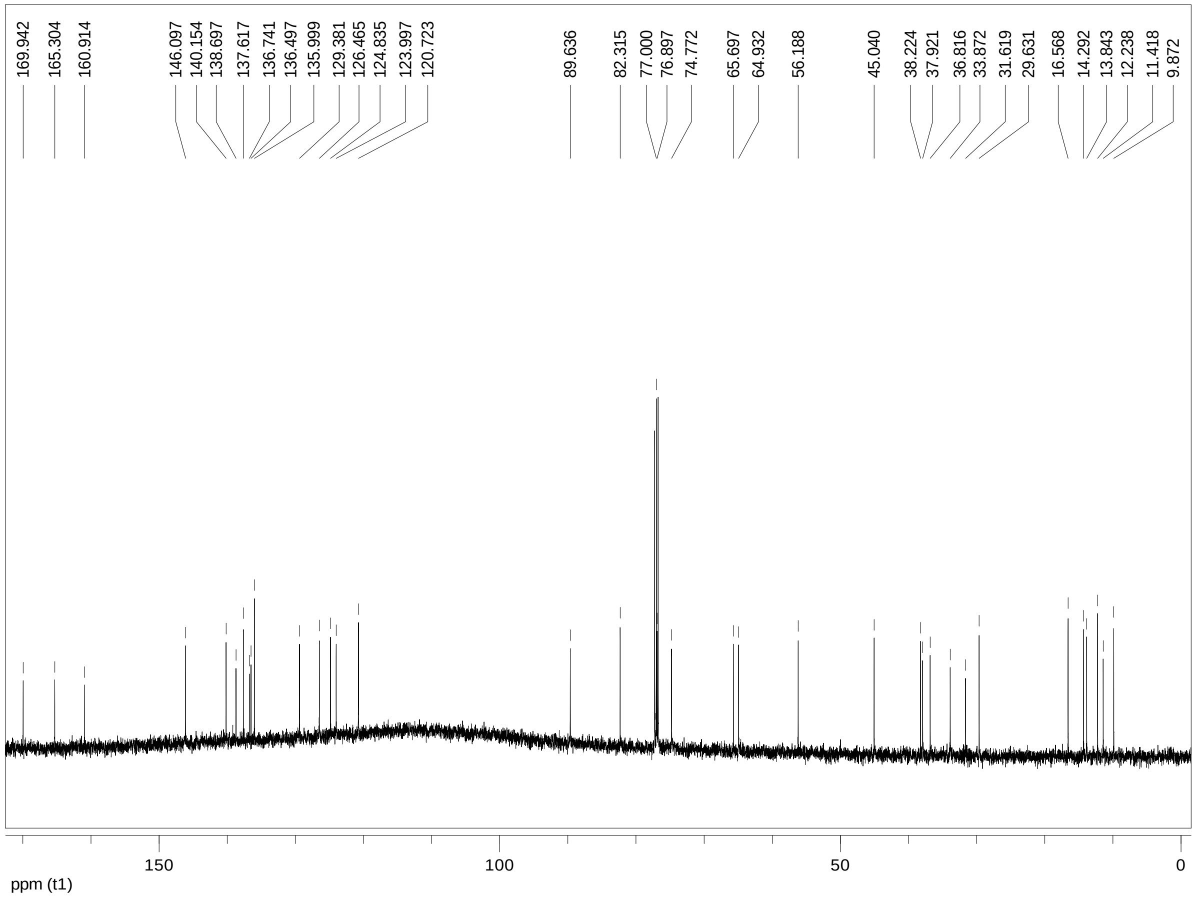
169.942
165.304
160.914
146.097
140.154
138.697
137.617
136.741
136.497
135.999
129.381
126.465
124.835
123.997
120.723
89.636
82.315
77.000
76.897
74.772
65.697
64.932
56.188
45.040
38.224
37.921
36.816
33.872
31.619
29.631
16.568
14.292
13.843
12.238
11.418
9.872
150
100
50
0
ppm (t1)
